# The positive feedback loop between SP1 and MAP2K2 significantly drives resistance to VEGFR inhibitors in clear cell renal cell carcinoma

**DOI:** 10.7150/ijbs.104591

**Published:** 2025-01-01

**Authors:** Zhinan Xia, Zitong Yang, Yu Dong, Xinyu Hao, Keliang Wang, Wenjiao Xia, Liangliang Ren, Tian Li, Min Xu, Guixin Zhu, Cheng Zhang

**Affiliations:** 1 Department of Urology, the Fourth Affiliated Hospital of School of Medicine, and International School of Medicine, International Institutes of Medicine, Zhejiang University, Yiwu, China.; 2 Department of Urology, the Fourth Affiliated Hospital of Harbin Medical University, Harbin, Heilongjiang, China.; 3 Department of Anesthesiology, the First Medical Center of Chinese PLA General Hospital, Beijing, China.; 4 Tianjin Key Laboratory of Acute Abdomen Disease-Associated Organ Injury and ITCWM Repair, Institute of Integrative Medicine of Acute Abdominal Diseases, Tianjin Nankai Hospital, Tianjin Medical University, Tianjin, China.; 5 Department of Urology, Affiliated Jinhua Hospital, Zhejiang University School of Medicine, Jinhua, Zhejiang, China.

**Keywords:** Clear cell renal cell carcinoma, Vascular endothelial growth factor receptor inhibitors, MAP2K2, Drug resistance

## Abstract

Clear cell renal cell carcinoma (ccRCC) is one of the most common and aggressive malignancies of the urinary system. Despite being the first-line treatment for advanced ccRCC, vascular endothelial growth factor receptor inhibitors (VEGFRis) face significant limitations due to both initial and acquired resistance, which impede complete tumor eradication. Using a CRISPR/Cas9 library screening approach, *MAP2K2* was identified as a resistance-associated gene for three prevalent VEGFRis (Sunitinib, Axitinib, and Sorafenib). A strong positive correlation was observed between MAP2K2 expression and resistance to these VEGFRis. Drug-resistant cell lines established through dose-escalation consistently exhibited elevated MAP2K2 expression and activation of the MEK/ERK signaling pathway. Notably, combining MEK inhibitors (MEKis) with VEGFRis significantly enhanced the sensitivity of these resistant cells, leading to pronounced cell death. Additionally, a positive feedback regulatory mechanism was discovered between SP1 and MAP2K2, wherein SP1 and MAP2K2 could enhance mutual expression, thereby maintaining MEK/ERK pathway activation. This study reveals that MEKis can effectively re-sensitize VEGFRi-resistant cells, offering a promising therapeutic strategy for overcoming VEGFRi resistance in ccRCC.

## Introduction

Renal cell carcinoma (RCC) ranks as the third most common malignancy of the urinary system, with clear cell renal cell carcinoma (ccRCC) representing the most prevalent pathological subtype, accounting for approximately 70-75% of all RCC cases 1. The widespread adoption of non-invasive imaging techniques, such as ultrasound and computed tomography (CT), has moderately improved the early detection rates of RCC. However, metastases are still detected in approximately 17% of patients at diagnosis 2. Currently, the first-line treatment for advanced RCC predominantly involves multitarget tyrosine kinase inhibitors (TKIs) and immune checkpoint inhibitors (ICIs), either as monotherapy or in combination 3. Despite these advancements, around 25% of patients exhibit poor responses to TKI therapy, and those who initially respond often develop secondary or acquired resistance after prolonged treatment. The median time to resistance development is approximately 6-12 months 4. Although molecular targeted therapies have demonstrated significant survival benefits for advanced RCC, drug resistance remains a majorobstacles to achieving complete tumor eradication, underscoring the urgent need for innovative cancer treatment strategies.

Tyrosine kinase inhibitors (TKIs) targeting vascular endothelial growth factor receptors (VEGFRs) not only suppress key signaling pathways that drive cancer cell proliferation but also disrupt tumor angiogenesis, reducing the tumor's blood supply and limiting its growth 5. Sorafenib, the first VEGFR inhibitor (VEGFRi) approved by the Food and Drug Administration (FDA) for the treatment of advanced RCC, was introduced in December 2005 6. Sunitinib, another VEGFRi, remains one of the most widely used treatments for RCC. Despite newer therapies demonstrating superior efficacy in both basic and clinical studies, Sunitinib continues to be a cornerstone of first-line treatment for advanced RCC 7. Axitinib, a second-generation VEGFRi, selectively inhibits VEGFR tyrosine kinase activity with 50-450 times greater potency compared to first-generation inhibitors 8. The high selectivity is regarded as a pivotal factor in the clinical benefits observed in patients 9. In addition to the three classic VEGFRis, the National Comprehensive Cancer Network (NCCN) guidelines also included other VEGFRis, such as Cabozantinib, Lenvatinib, Pazopanib, and Tivozanib, further expanding the role of targeted therapies in RCC management 7.

CRISPR/Cas9 library screening enables the mapping of specific phenotypic traits to genetic perturbations, while simultaneously sequencing these perturbations to identify genetic features associated with phenotypic variations 10-12. Understanding the mechanisms underlying VEGFRi resistance and developing strategies to overcome this resistance are critical for improving the treatment outcomes of advanced RCC patients. In this study, *MAP2K2*, was consistently identified as a resistance gene fo three VEGFRis through CRISPR/Cas9 library screening. MAP2K1 and MAP2K2 (collectively known as MEK1/2) are central components of the MEK/ERK signaling pathway. These kinases transmit signals from various upstream regulators, such as SOS, RAS, and RAF, and function as the exclusive activators of downstream ERK1/2, effectively serving as "gatekeepers" of ERK1/2 signaling 13. Notably, all four VEGFRi-resistant cell lines exhibited upregulated MAP2K2 expression and activation of the MEK/ERK pathway. Further experiments revealed that combining MEK inhibitors (MEKis) with VEGFRis significantly enhanced the sensitivity of resistant cells to VEGFRis, leading to effective cell death. Additionally, the transcription factor SP1 was found to regulate MAP2K2 expression, establishing a positive feedback loop between SP1 and MAP2K2. This mutual upregulation perpetuates MEK/ERK pathway activation, contributing to the resistance of ccRCC cells to VEGFRis.

## Materials and Methods

### Clinical samples

Forty tissue samples, comprising both normal kidney tissue and RCC tissue, were collected from 20 randomly selected patients who underwent radical nephrectomy for RCC. This study was approved by the Ethics Committee of the Fourth Affiliated Hospital, Zhejiang University School of Medicine (K2024150), and all procedures were conducted in accordance with the principles of the Declaration of Helsinki.

### Xenotransplanted tumors and gavage administration

Male BALB/c nude mice of SPF grade, approximately 5 weeks old, were used in the experiments and obtained from Model Organisms (Shanghai, China). All animal experimental procedures were approved by the Animal Ethics Committee of Zhejiang University (ZJU20220502) and conducted in accordance with the guidelines for animal experiments of laboratory animals.

First, a 1% carboxymethyl cellulose sodium salt solution was prepared as a solvent. Afterwards, 40mg of sunitinib powder, 20mg of axitinib, and 1mg of trametinib were weighed and resuspended in 10mL of the prepared solvent. Subsequently, 10% DMSO was added as a co-solvent and the mixture was sonicated on ice until a homogeneous solution was obtained. A total of 2×10^7^ cells were injected into the axilla of each nude mouse. Once tumor volumes reached approximately 50 mm³, mice were administered oral treatments. For different groups, medication and DMSO control were administered daily, including Sunitinib (40mg/kg), Axitinib (20mg/kg), or Trametinib (1mg/kg). Tumor size and changes in the body weight of the nude mice were measured weekly. After four weeks of treatment, the mice were euthanized, and tumors were excised, weighed, and photographed.

### Cell lines

The ccRCC cell lines used in this research were obtained from the American Type Culture Collection (ATCC) cell bank (786-O cell line) and the National Collection of Authenticated Cell Cultures (OS-RC-2 cell line) (Shanghai, China). All cell lines were identified by the short tandem repeat (STR) analysis to eliminate cross contamination of cells. The methodology is mainly according to our previously published literature 14 with minor revision.

### CRISPR/Cas9 knockout library screening

In this study, the customized CRISPR knockout library (Megarobo Co., Ltd., Beijing, China) was utilized to identify genes associated with VEGFRi resistance in 786-O ccRCC cell line. The CRISPR/Cas9 knockout library comprised 11,861 sgRNAs, including 10,861 targeting 1,811 genes and 1,000 targeting control genes as positive controls (Supplementary Table S1). Each gene in the library was represented by 5-6 sgRNAs, which were derived from previously published genome-wide CRISPR libraries 15. The sgRNA library was packaged into lentivirus vectors, utilizing a dual-vector system with different antibiotic selection markers 16. The methodology primarily followed established protocols 10 with minor modification. sgRNA sequences were amplified using 2× Taq Plus Master Mix (Vazyme Co., Ltd., Nanjing, China) and subjected to massive parallel amplicon sequencing carried out by Novogene Co., Ltd. (Beijing, China). The sgRNA read counts and hit identification were analyzed using the MAGeCK algorithm 17.

### Construction of drug-resistant cells

All VEGFRis were purchased from Selleck Co., Ltd. (Shanghai, China). When the cell confluence was 30-40%, the original cell culture medium of 786-O or OS-RC2 cells was replaced with a complete culture medium containing either Sunitinib or Axitinib. Initially, the cells were treated with 2 μM Sunitinib or 1.5 μM Axitinib and passaged after 48 hours of incubation. Over the course of a week, the concentrations of Sunitinib were gradually increased based on the cells' responses, with a similar escalation approach applied to Axitinib. The approach continued until final concentrations of 13μM Sunitinib and 5μM Axitinib were reached for 786-O cells, and 9μM Sunitinib and 4μM Axitinib for OS-RC-2 cells. The cells exhibited stable growth at these final concentrations. The drug-resistant cell lines were designated as 786-O Suni-R, 786-O Axi-R, OS-RC-2 Suni-R, and OS-RC-2 Axi-R.

### Lentivirus and small-interfering RNA (siRNA)

The gene interference lentivirus and overexpression lentivirus used in this study were designed, vector-constructed, and virus-packaged by by GeneChem Co., Ltd. (Shanghai, China). The siRNAs were designed and synthesized by GenePharma Co., Ltd. (Shanghai, China). siRNA vector transfection was carried out using jetPRIME (PolyPlus, New York, NY, USA). The sequences are presented in Supplementary Table S2. The methodology is mainly according to our previously published literature 14 with minor revision.

### Cell counting kit-8 (CCK-8) assay

The methodology is mainly according to our previously published literature 14 with minor revision.

### Long-term clonogenic assays

The methodology is mainly according to our previously published literature 14, 18 with minor revision.

### Flow cytometry for apoptosis detection

The methodology is mainly according to our previously published literature 18 with minor revision.

### Quantitative reverse transcription polymerase chain reaction (qRT-PCR)

The sequences of primers are presented in Supplementary Table S3. The methodology is mainly according to our previously published literature 14 with minor revision.

### Chromatin Immunoprecipitation Assay (ChIP)

The methodology is mainly according to our previously published literature 14 with minor revision. The primer sequences for ChIP-qPCR are shown in Supplementary Table S3. Among the three pairs of primers for MAP2K2 promoter, only primer 1 can PCR amplify the corresponding fragment. This proves that the binding site occurs at the position corresponding to primer 1 region.

### Western blotting and immunohistochemistry (IHC)

MEK1 mouse mAb (61B12), phospho-MEK1/2 rabbit mAb (41G9), ERK1/2 rabbit mAb (137F5), phospho-ERK1/2 mouse mAb (D1H6G), HSP90 rabbit mAb (C45G5), and GAPDH rabbit mAb (14C10) antibodies were purchased from Cell Signaling Technology (Danvers, MA, USA). MEK2 rabbit mAb antibody (ab32517) was purchased from Abcam (Cambridge, UK). The SP1 polyclonal antibody (21962-1-AP) was purchased from Proteintech (Wuhan, China). The methodology is mainly according to our previously published literatures 18 with minor revision.

### Statistical analysis

Statistical analysis was performed using GraphPad Prism version 8. Most experiments were conducted at least three times to obtain representative data. Results were expressed as mean ± SEM. Differences between two groups were evaluated using a two-tailed Student's t-test, while one-way or two-way ANOVA was employed for multiple comparisons. A p-value of <0.05 was considered statistically significant for all tests.

## Results

### Constructing a CRISPR/Cas9 knockout library for screening genes resistant to VEGFRis

**Figure 1:**
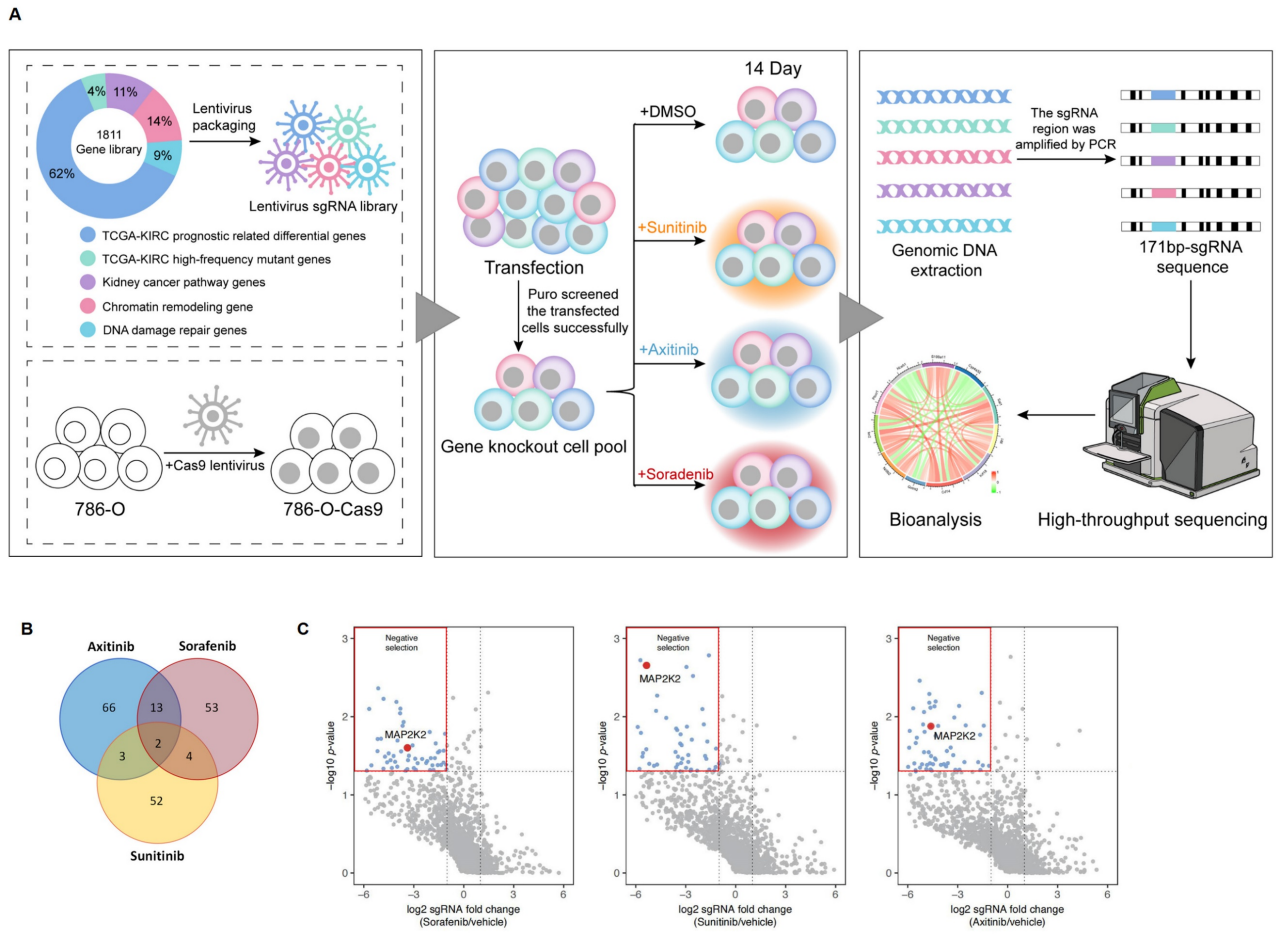
** CRISPR/Cas9 Library Screening Identified *MAP2K2* as a Gene Resistant to VEGFR Inhibitors.** (A) CRISPR/Cas9 library screening workflow. (B) The analysis of significant resistance genes for Sunitinib, Axitinib, and Sorafenib revealed two key genes, particularly *MAP2K2*, that were consistently significant across all three inhibitors. The volcano plot (C) illustrates that in cells treated with these three VEGFRis, sgRNAs targeting *MAP2K2* were notably depleted. *MAP2K2* was thus identified as a significant resistance gene for all three drugs (logFC < -1, P < 0.05).

To identify genes responsible for resistance to VEGFRis, a CRISPR/Cas9 knockout library targeting 1,811 genes was constructed. These genes were divided into five categories: 1,118 differentially expressed genes (DEGs) related to prognosis in ccRCC cells from The Cancer Genome Atlas (TCGA) database, the top 63 genes with the highest mutation rates in ccRCC, based on the TCGA single nucleotide variation (SNP) database, 199 genes related to the renal cancer pathway, 261 genes involved in chromatin remodeling, and 170 genes associated with DNA damage repair. The library screening process is illustrated in Figure 1A.

Negative screening of genes corresponding to differentially expressed sgRNAs resulted in the identification of drug-resistant genes. Analysis of significant resistance genes across the three groups of VEGFRis revealed two genes, *MAP2K2* and *PCSK5*, common to all groups. These genes were found to potentially confer resistance to all three VEGFRis simultaneously (Figure 1B, C). Based on a literature review and preliminary experimental results, *MAP2K2* was selected for further investigation in this study.

### MAP2K2 expression level in ccRCC cells and its association with poor prognosis

**Figure 2:**
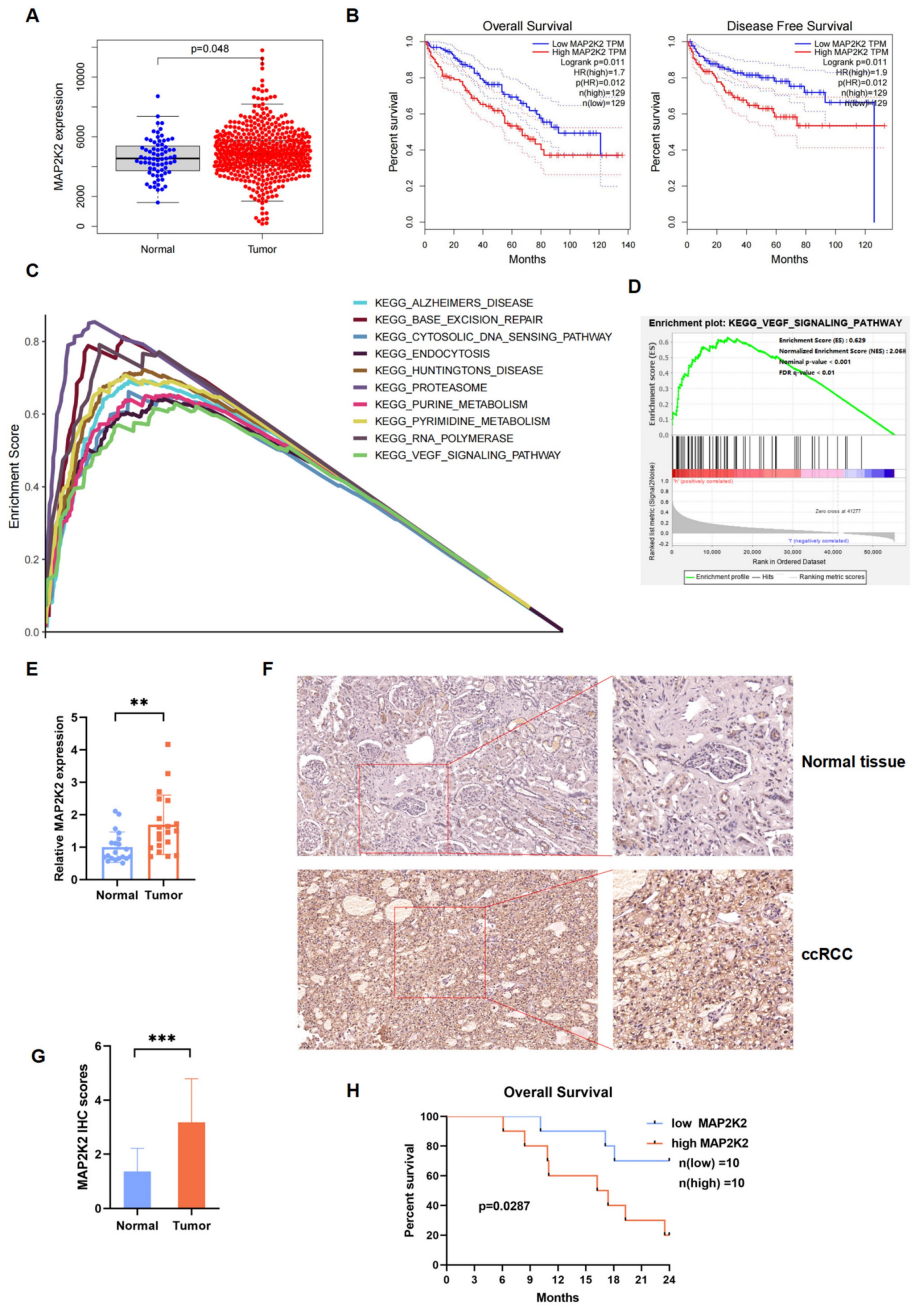
**MAP2K2 was Highly Expressed in ccRCC and Associated with Poor Prognosis.** (A) Differential analysis of MAP2K2 expression in ccRCC and normal kidney samples from TCGA database (P < 0.05). (B) OS and DFS curves for ccRCC patients grouped by high and low MAP2K2 expression levels (P < 0.05). (C) Multiple GSEA plots depicting the top ten KEGG pathways that were positively correlated with MAP2K2 expression level. (D) GSEA indicated a positive correlation between MAP2K2 expression level and the VEGF signaling pathway. (E) The qRT-PCR analysis of MAP2K2 mRNA expression level in normal renal tissue and tumor tissue from ccRCC patients. (F, G) Immunohistochemical analysis of MAP2K2 in clinical patient samples, including representative images and corresponding quantification. (H) Kaplan-Meier OS analysis of ccRCC patients with low and high MAP2K2 expression levels, with the median MAP2K2 expression level used as the cutoff value.

Transcriptomic and clinical data from the TCGA-KIRC database were analyzed, including 611 samples: 539 ccRCC samples and 72 normal samples, were enrolled. Differential expression analysis revealed that MAP2K2 expression level was significantly higher in renal cancer tissues compared to normal tissues (Figure 2A). Survival analysis indicated that ccRCC patients with high MAP2K2 expression level showed shorter OS and disease-free survival (DFS) compared to those with low MAP2K2 expression level (Figure 2B).

The relationship between clinical pathological features and MAP2K2 expression level was examined using the Wilcoxon rank-sum test. The results unveiled that higher MAP2K2 expression was associated with advanced Fuhrman grade, clinical stage, tumor size, distant metastasis, and lymph node metastasis Supplementary Figure 1A-E). Gene set enrichment analysis (GSEA) identified the top 10 pathways positively correlated with MAP2K2 expression, including the VEGF signaling pathway (Figure 2C, D), indirectly suggesting the potential role of MAP2K2 in VEGFRi resistance.

In 40 paired clinical specimens from 20 ccRCC patients, MAP2K2 expression was significantly upregulated at the RNA level in cancerous tissues compared to normal renal tissues (Figure 2E). Immunohistochemical (IHC) analysis of histological sections confirmed these findings (Figure 2F,2G). Consistent with the bioinformatics results, patients with higher MAP2K2 expression level exhibited poorer OS rates (Figure 2H).

### Activation of the MEK/ERK pathway was positively correlated with VEGFRi resistance

**Figure 3:**
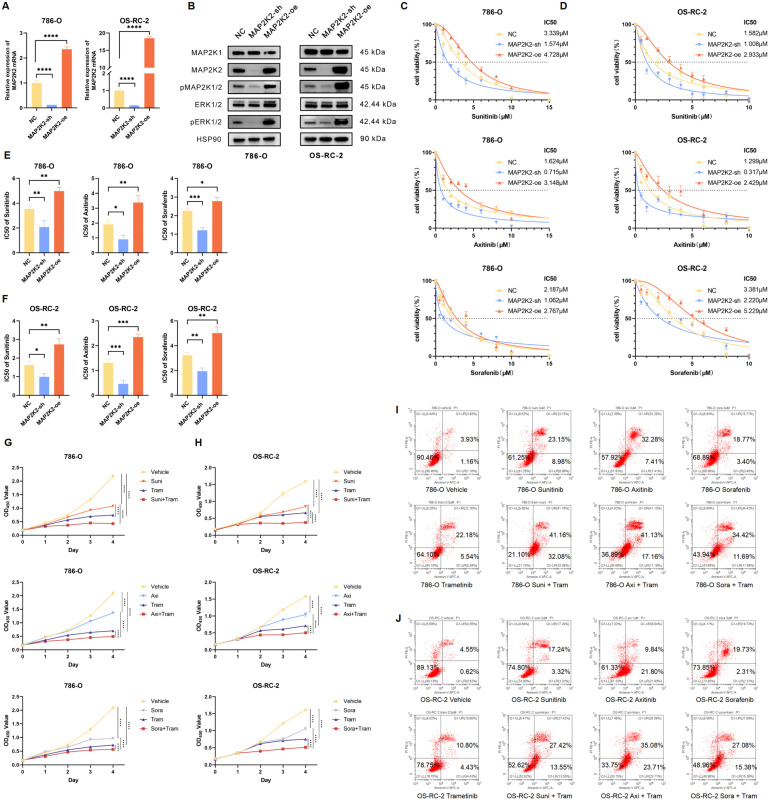
**MEK/ERK Pathway Activation was Correlated Positively with VEGFRi Resistance.** (A) Relative mRNA expression levels in 786-O and OS-RC-2 cell lines following MAP2K2 knockdown and overexpression. (B) Western blot analysis of MEK/ERK pathway-associated proteins in 786-O and OS-RC-2 cell lines after MAP2K2 knockdown and overexpression. (C, D) IC50 curves for Sunitinib, Axitinib, and Sorafenib in the 786-O cell line and the OS-RC-2 cell line. (E, F) IC50 values for Sunitinib, Axitinib, and Sorafenib in the 786-O cell line and the OS-RC-2 cell line from three independent experiments, with statistical differences following MAP2K2 knockdown and overexpression determined using one-way ANOVA. * P <0.05; ** P <0.01; *** P <0.001; **** P <0.0001. (G) Proliferation curves for 786-O cells treated with DMSO (control), Trametinib (0.5μM), Sunitinib (3μM), Axitinib (2μM), and Sorafenib (3μM), both individually and in combination. (H) Proliferation curves for OS-RC-2 cells treated with Trametinib (0.5μM), Sunitinib (2μM), Axitinib (1μM), and Sorafenib (2μM), both individually and in combination. (I, J) Apoptosis of 786-O cells and OS-RC-2 cells treated with the same drug concentrations as previously mentioned, using flow cytometry. Trametinib is abbreviated as Tram, Sunitinib as Suni, Axitinib as Axi, and Sorafenib as Sora.

To validate the accuracy of the library screening, cell experiments were conducted to verify the role of MAP2K2 in VEGFRi resistance. First, stable MAP2K2 knockdown and overexpression cell lines (MAP2K2-sh and MAP2K2-oe) were established in the ccRCC cell lines 786-O and OS-RC-2 using shRNA and overexpression vector lentiviruses, respectively. Lentiviruses carrying empty target sequences served as negative controls (NC) (Figure 3A, B). Notably, knocking down or overexpressing MAP2K2 also inhibited or enhanced the activation level of pERK1/2, thereby modulating the MEK/ERK pathway (Figure 3B).

Next, the CCK-8 assay was employed to determine the half inhibitory concentration (IC50) values of Sunitinib, Axitinib, and Sorafenib in NC, MAP2K2-sh, and MAP2K2-oe cells (Figure 3C, D). Each experiment was performed in triplicate. In each experimental group, MAP2K2 knockdown reduced the IC50 values of the three VEGFRis, whereas MAP2K2 overexpression increased the IC50 values (Supplementary Figure 2A, B). Visual quantification of IC50 results from three independent experiments indicated that MAP2K2 knockdown increased ccRCC cell sensitivity to VEGFRi, while MAP2K2 overexpression enhanced resistance (Figure 3E, F). Moreover, siRNA was utilized to knock down MAP2K2, its homolog MAP2K1, and the downstream kinase ERK in the 786-O cell line. Knockdown of MAP2K1/2 and ERK reduced the IC50 of VEGFRi (Supplementary Figure 2C-E). Collectively, these results suggested that MAP2K2 expression regulated activation of the MEK/ERK pathway, which, in turn, influenced VEGFRi sensitivity in ccRCC cells.

Trametinib, a highly specific MAP2K1/2 inhibitor approved by the FDA for the treatment of tumors such as melanoma and non-small cell lung cancer, was used as the MEKi in this experiment. Compared with the control group, the monotherapy group reduced ccRCC cell proliferation. The combination therapy group exhibited even stronger inhibitory effects (Figure 3G, H). Additionally, flow cytometry was used to assess cell apoptosis 72 hours after drug administration (Figure 3I, J). The proportion of early and late apoptotic cells in the monotherapy group was significantly higher than that in the control group. Apoptosis levels in the combination group of Trametinib and VEGFRi were significantly higher than those in the monotherapy group.

### Construction and evaluation of Sunitinib and axitinib resistant ccRCC cells

**Figure 4:**
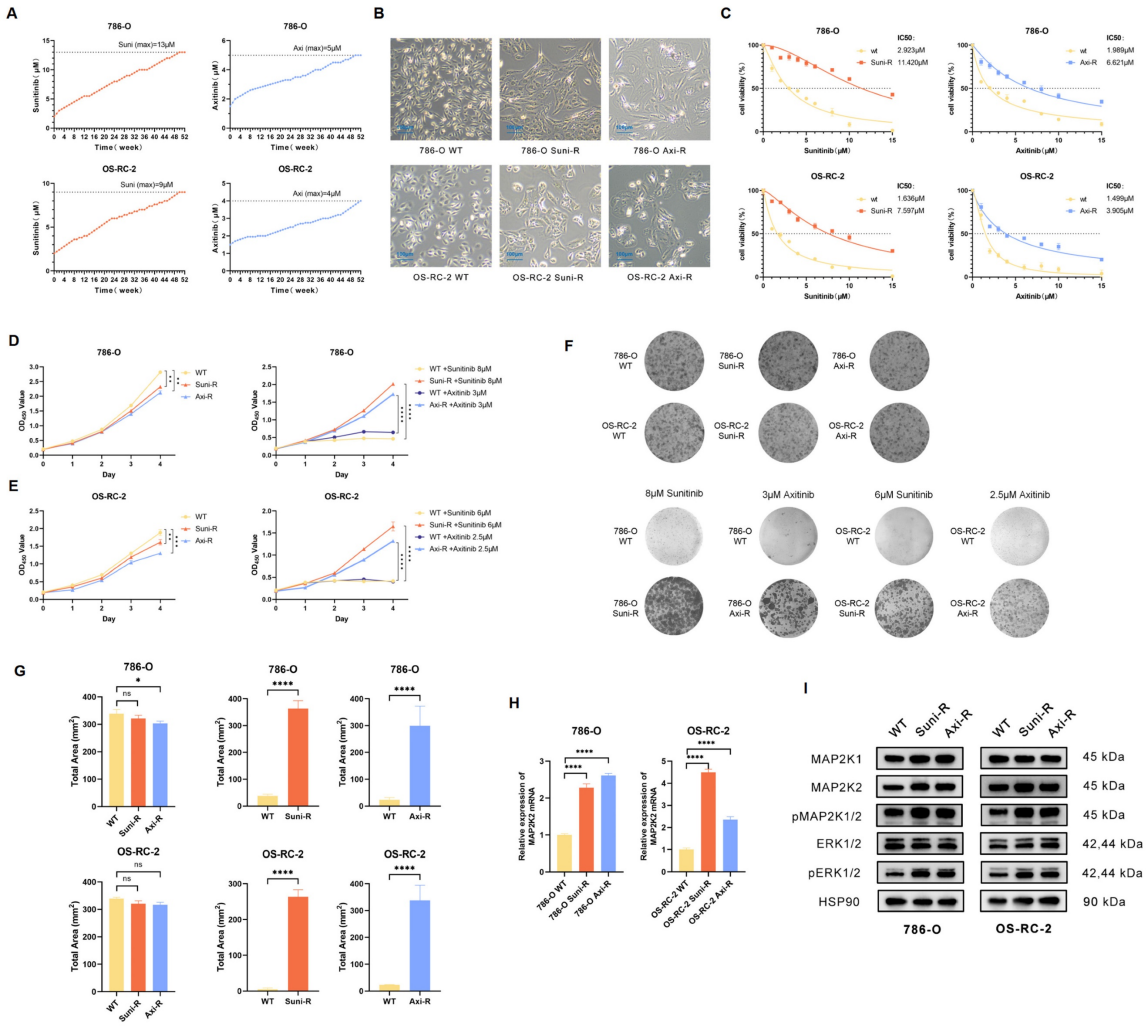
**Construction and Evaluation of Drug-Resistant Cells.** (A) A gradual increase in Sunitinib and Axitinib concentrations in 786-O and OS-RC-2 cells over time. (B) Cellular morphology observed under 40× magnification. (C) Comparison of IC50 values between resistant and wild-type cells. (D) Proliferation curves of resistant and wild-type cells without drug treatment. (E) Proliferation curves of resistant and wild-type cells under high drug concentrations (786-O: Sunitinib+13μM, Axitinib+5μM; OS-RC2: Sunitinib+9μM, Axitinib+4μM). (F) The above shows the colony formation assay of drug-resistant cells and wild-type cells without drug treatment. Below is the colony formation assay under high drug concentrations. (G) Quantification of the total shaded area of cells in each group from the colony formation assay. (H) qRT-PCR results revealed a significant increase in MAP2K2 mRNA expression level in Sunitinib- and Axitinib-resistant strains compared with wild-type cells in both 786-O and OS-RC-2 cell lines. (I) Western blot analysis showed the expression levels of MEK/ERK pathway-associated proteins in drug-resistant and wild-type cells.

To investigate the mechanism of VEGFRi resistance and verify the role of MAP2K2, two of the most common clinical VEGFRis, Sunitinib and Axitinib, were selected to generate resistant strains in 786-O and OS-RC-2 wild-type cells. A sequential drug induction method was employed, where the drugs were progressively introduced from lower to higher concentrations over 52 weeks (Figure 4A). The resulting drug-resistant cells were designated as 786-O Suni-R, 786-O Axi-R, OS-RC-2 Suni-R, and OS-RC-2 Axi-R.

Microscopic examination revealed morphological differences between drug-resistant and wild-type cells (Figure 4B). The IC50 values, determined using the CCK-8 assay, revealed that all four drug-resistant cell lines exhibited significantly higher IC50 values (Figure 4C). In the absence of additional drugs, the proliferation rate of drug-resistant cells was slightly lower than that of wild-type cells (Figure 4D). However, upon exposure to high drug concentrations, the proliferation of drug-resistant cells was significantly enhanced (Figure 4E). The colony formation test further confirmed that drug-resistant cells retained a strong ability to form colonies at high drug concentrations (Figure 4F, G). These findings indicate that the drug-resistant cells have developed significant resistance to VEGFR inhibitors and warrant further experimental research. To validate the CRISPR/Cas9 library findings, MAP2K2 expression and MEK/ERK pathway activation were assessed in drug-resistant cells. The results of qRT-PCR showed upregulation of MAP2K2 mRNA expression in all four drug-resistant cell lines (Figure 4H). Western blot analysis confirmed that drug-resistant cells exhibited elevated MAP2K2 protein expression, along with increased expression of pMEK1/2 and pERK1/2, indicating heightened activation of the MEK/ERK pathway (Figure 4I).

### Knocking down MAP2K2 and using MEKi could effectively reduce drug resistance to VEGFRi

**Figure 5:**
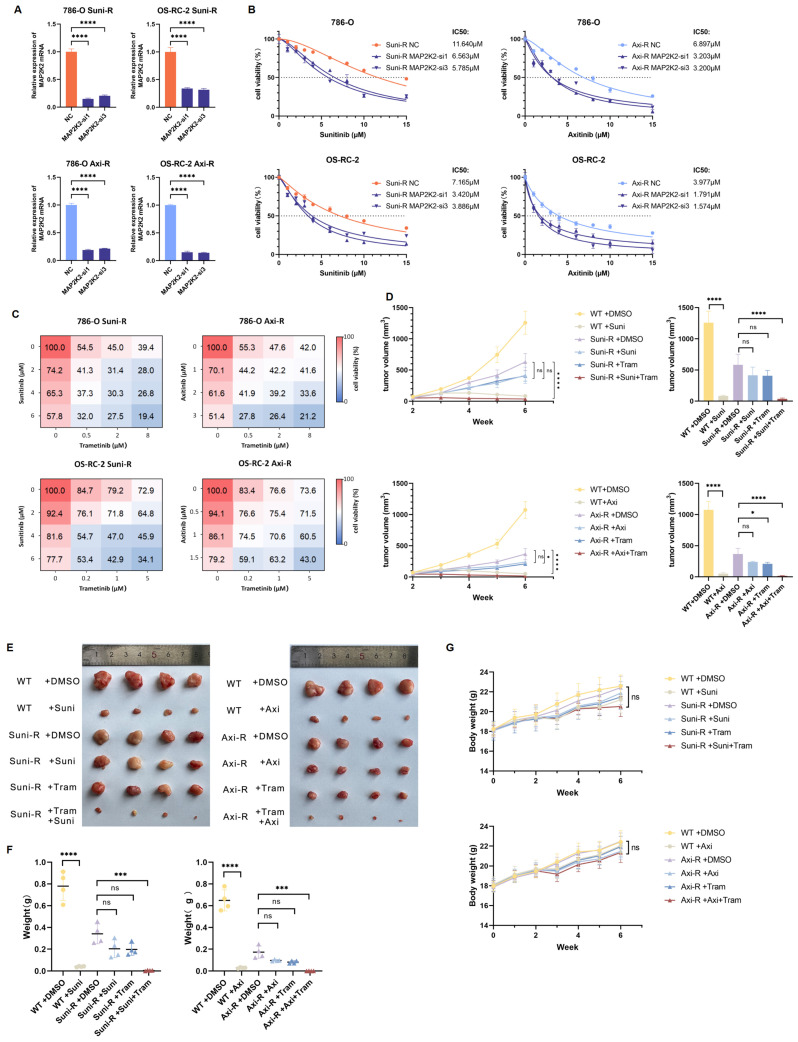
**Knocking down MAP2K2 and MEKi could effectively reduce the resistance of drug-resistant cells to VEGFRi.** (A) Relative mRNA expression level of MAP2K2 after knocking down MAP2K2 in drug-resistant cells. (B) Differences in IC50 values for Sunitinib and Axitinib after MAP2K2 knockdown in drug-resistant cells. (C) Assessment of cell survival in drug-resistant 786-O and OS-RC-2 cells exposed to varying drug concentrations, with a color gradient from red to blue indicating increasing cytotoxicity. (D) Growth curves of tumor size progression over time. (E) Photographs of tumors removed after four weeks of drug administration. (F) Weights of tumors across different experimental groups. (G) Variations in the body weight of nude mice throughout the study period.

In this study, siRNA was employed to knock down MAP2K2 in drug-resistant cells, and the knockdown efficiency was validated (Figure 5A). Results showed that MAP2K2 knockdown significantly reduced the resistance of drug-resistant cells (Figure 5B). This finding corroborates the library screening results, further confirming that elevated MAP2K2 expression contributes to VEGFRi resistance.

Previous experiments demonstrated that combining MEKi and VEGFRi effectively killed wild-type ccRCC cells. The present study explored whether MEKi could sensitize cells resistant to VEGFRi to the treatment. *In vitro* experiments were conducted using gradient concentrations of Trametinib combined with Sunitinib or Axitinib, with drug concentrations determined based on our previous experimental results (Supplementary Figure 3A). Drug-resistant cells were treated in 12-well plates. The results indicated that single-drug treatments were ineffective against resistant cells. However, the combination of MEKi and VEGFRi exhibited significant anti-tumor effects, effectively killing drug-resistant cells (Figure 5C).

Next, wild-type 786-O cells, 786-O Suni-R, and 786-O Axi-R resistant cells were subcutaneously injected into nude mice. Results demonstrated that wild-type 786-O cells remained highly sensitive to Sunitinib or Axitinib, whereas the resistant 786-O Suni-R and 786-O Axi-R cells showed pronounced resistance to these VEGFRis (Figure 5D-F). In resistant cells, monotherapy with VEGFRis showed no significant impact on tumor size compared to untreated controls. However, combination therapy with MEKi and VEGFRi, particularly Trametinib with Sunitinib or Axitinib, significantly enhanced anti-tumor efficacy in resistant cells (Figure 5D-F). Furthermore, mice displayed good tolerance to combination therapy (Figure 5G). These findings suggest that MEKi substantially improves VEGFRi efficacy in drug-resistant cells, offering a promising strategy for treating patients with VEGFRi-resistant ccRCC.

### Positive feedback loop of transcription factors SP1 and MAP2K2 could lead to VEGFRi resistance

**Figure 6:**
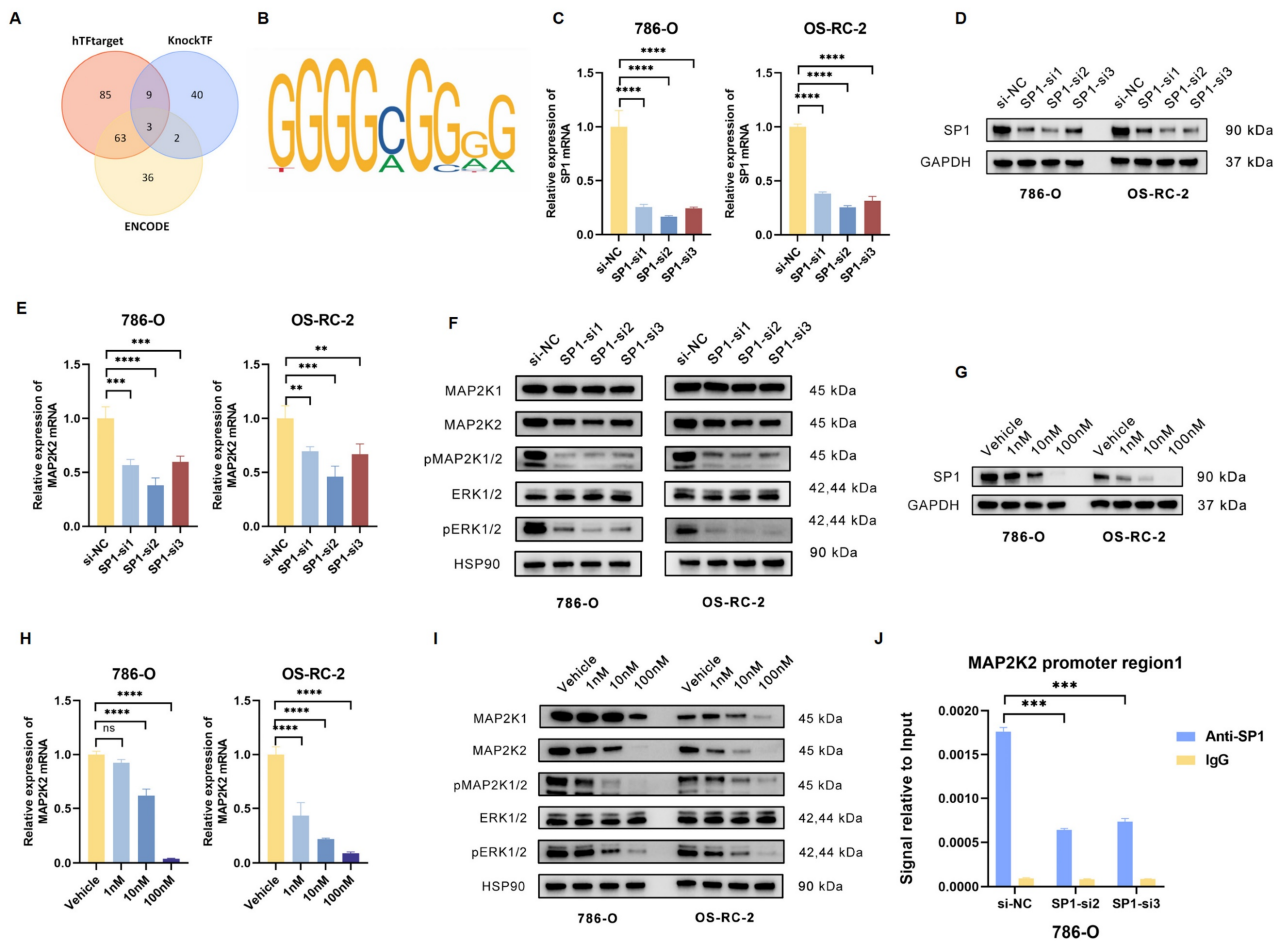
**Transcription factor SP1 could regulate MAP2K2 expression level.** (A) Prediction of MAP2K2 transcription factors using three databases: ENCODE, hTFtarget, and KnockTF. The results revealed three intersecting transcription factors. (B) Predicted binding sites of SP1 on the MAP2K2 promoter from the JASPAR database. (C) Transfection of three independent siRNAs to knockdown SP1 in 786-O and OS-RC-2 cells, showing the relative mRNA expression level of SP1 in each group. (D) Protein expression level of SP1 after siRNA-mediated knockdown. (E) Relative mRNA expression level of MAP2K2 after SP1 knockdown. (F) Expression levels of MEK/ERK pathway-associated proteins after SP1 knockdown. (G) Protein expression level of SP1 after treatment with different concentrations of the SP1 inhibitor Plicamycin in 786-O and OS-RC-2 cells. (H) Relative mRNA expression level of MAP2K2 after treatment with the SP1 inhibitor. (I) Expression levels of MEK/ERK pathway-associated proteins after SP1 inhibitor treatment. (J) ChIP-qPCR analysis in 786-O cells, comparing SP1 antibody with IgG control to evaluate the binding of SP1 to the MAP2K2 promoter.

Due to the significant upregulation of MAP2K2 mRNA in drug-resistant cells, it was hypothesized that the abnormal expression of MAP2K2 may be influenced by transcriptional regulation. To investigate this, three online transcription factor prediction websites (ENCODE, hTFtarget, and KnockTF) were employed to predict transcription factors potentially regulating MAP2K2. By intersecting the predictions of three databases, three intersecting transcription factors were identified (Figure 6A). Subsequently, the JASPAR database was thereafter utilized to predict the binding sites of these three transcription factors within the MAP2K2 promoter region (Figure 6B). A relative prediction score threshold of 0.9 was applied, revealing that among the three candidates, only *SP1* met this criterion, with scores consistently exceeding 0.95.

Subsequently, SP1 expression was knocked down in 786-O and OS-RC-2 by transfecting siRNA (Figure 6C, D). The results demonstrated that SP1 knockdown significantly reduced MAP2K2 expression levels and inhibited activation of the MEK/ERK pathway (Figure 6E, F). Additionally, treatment with the specific SP1 inhibitor Plicamycin showed that increasing concentrations of the inhibitor progressively enhanced its suppression of SP1, as confirmed by Western blotting (Figure 6G). Quantification of mRNA expression levels of MAP2K2 and MEK/ERK pathway-related proteins in Plicamycin-treated cells further confirmed that SP1 inhibition reduced MAP2K2 expression and suppressed MEK/ERK pathway activation (Figure 6H, I).

In order to verify the direct binding of SP1 to the MAP2K2 promoter region, chromatin immunoprecipitation (ChIP) experiments were performed. Firstly, two siRNAs with the most significant knockdown effect on SP1 in 786-O cells were utilized for transfection. Subsequently, immunoprecipitation was performed on the chromatin of each group of cells using SP1 antibody and IgG NC. Compared to the IgG control, a significant enrichment of SP1 binding to the MAP2K2 promoter region was observed. Following SP1 knockdown, the interaction was markedly reduced, confirming the binding of SP1 to the MAP2K2 promoter (Figure 6J). These findings demonstrated that SP1 directly binds to the MAP2K2 promoter region, promoting its transcription and thereby activating the MEK/ERK pathway.

**Figure 7:**
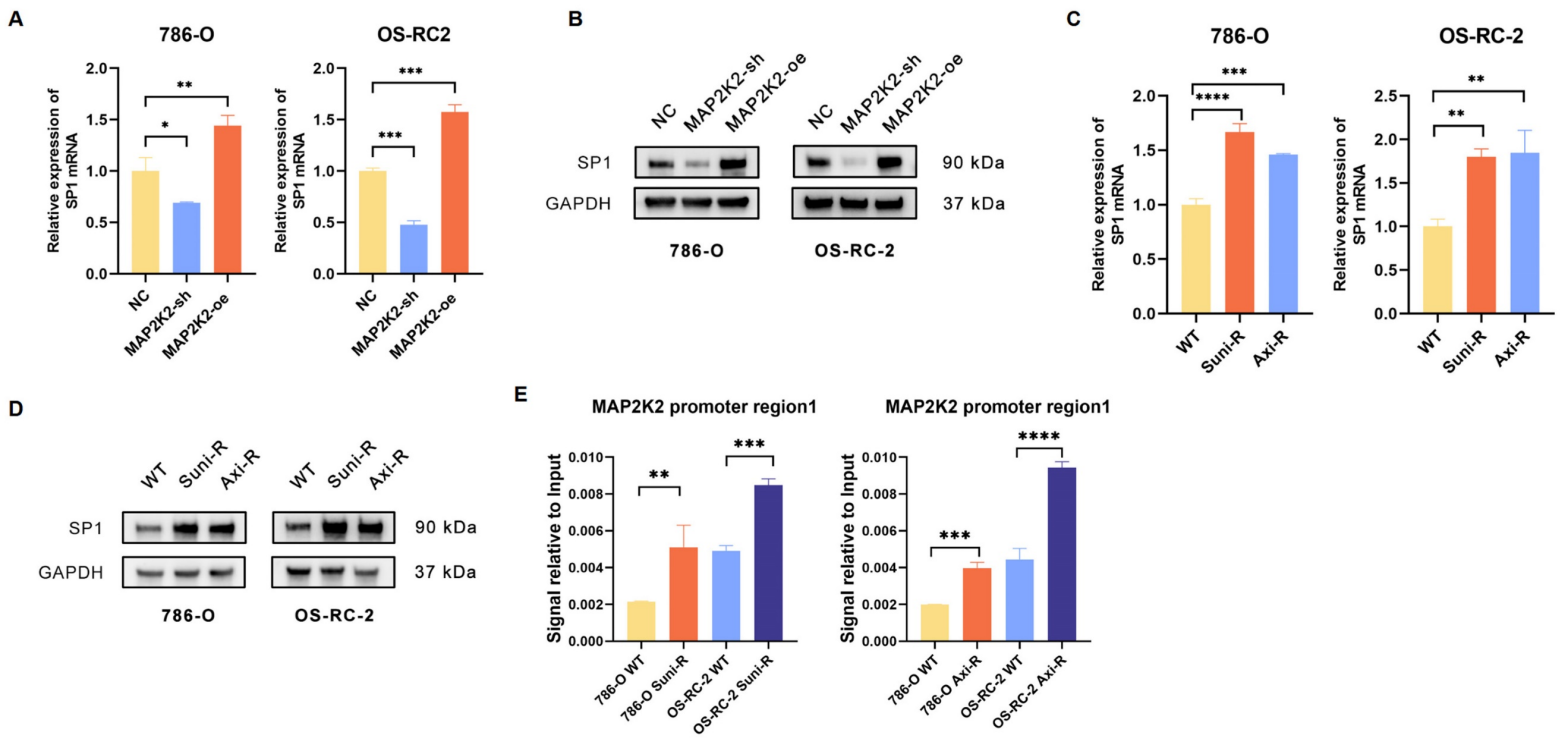
**Positive feedback loop of transcription factors SP1 and MAP2K2.** (A) Relative mRNA expression level of SP1 in 786-O and OS-RC-2 cells upon knockdown or overexpression of MAP2K2. (B) Protein expression level of SP1 upon knockdown or overexpression of MAP2K2 in 786-O and OS-RC-2 cells. (C) Relative mRNA expression level of SP1 in drug-resistant cells. (D) Protein expression level of SP1 in drug-resistant cells. (E) ChIP-qPCR assay was used to detect the binding level of MAP2K2 promoter in drug-resistant cells.

Interestingly, MAP2K2 knockdown was shown to downregulate SP1 expression, while MAP2K2 overexpression upregulated SP1 expression (Figure 7A, B). Analysis of SP1 expression levels in drug-resistant cells revealed that both mRNA and protein levels of SP1 were significantly elevated in these cells compared to wild-type cells (Figure 7C, D). Additionally, we found that, similar to MEKi, SP1 inhibitors could also enhance the sensitivity of VEGFRis (Supplementary Figure 3B). Furthermore, ChIP-qPCR experiments demonstrated increased SP1 binding to the MAP2K2 promoter in drug-resistant cells, which promoted elevated MAP2K2 expression (Figure 7E). These findings confirm a positive feedback loop in which activation of the MEK/ERK pathway upregulates SP1 expression, reinforcing VEGFRi resistance in ccRCC cells.

## Discussion

### Main interpretation

Advanced or metastatic RCC, characterized by tumor invasion beyond the renal fascia and spread to regional lymph nodes or distant sites (19, affects approximately 17% of patients at initial diagnosis. Additionally, over 20% of RCC patients develop distant metastases after radical nephrectomy. RCC remains resistant to traditional radiotherapy and chemotherapy 20. Since 2005, targeted therapies, particularly VEGFR inhibitors (VEGFRis), have largely replaced cytokine therapies 21. While VEGFRis provide survival benefits for advanced RCC patients, resistance to these therapies poses a significant challenge, undermining their efficacy and adversely affecting patient outcomes. The mechanisms underlying VEGFRi resistance have not yet been fully explored, and there is a lack of mechanisms to guide clinical treatment and biomarkers to identify resistance. Addressing drug resistance remains a critical concern for clinicians.

The genes resistant to VEGFRis were screened through high-throughput CRISPR/Cas9 library, and two genes were obtained by intersecting the genes resistant to three commonly used VEGFRis in clinical practice. Based on our preliminary experimental results, which show that knocking down MAP2K2 significantly sensitizes cells to VEGFR inhibitors, and considering that small molecule inhibitors targeting this gene are already available, making it easier for clinical translation, we chose MAP2K2 for further study. Two cell-resistant models of VEGFRis were constructed by simulating the process of acquired VEGFRi resistance in patients. Upregulation of MAP2K2 expression level was also detected in the resistant cell models. The CRISPR/Cas9 library screening was combined with the results of drug-resistant cells, followed by their validation through a series of *in vitro* and *in vivo* experiments to make the research results more clinically relevant, credible, and potential for clinical translation.

After verifying the role of MAP2K2 upregulation in VEGFRi resistance, its mechanism was explored. Due to the significant upregulation of MAP2K2 mRNA level in drug-resistant cells, the underlying mechanisms were investigated at the transcriptional regulatory level. We confirmed the positive feedback loop between transcription factor SP1 and MAP2K2, which could continuously activate the MEK/ERK pathway and contribute to the development of cellular resistance to VEGFRis.

We further evaluated the therapeutic impact of MEKi, VEGFRi, and combination therapy on drug-resistant cells through comprehensive in vitro and in vivo experiments. Our results showed that MEKi significantly enhanced VEGFRi sensitivity, enabling effective eradication of resistant tumor cells. These findings highlight the clinical translational potential of MEKi in overcoming VEGFRi resistance in advanced RCC.

### Limitations

This research has certain limitations. In construction of drug-resistant cells, the sequential induction method reported in the literature 22-24 was used. However, the IC50 value for OS-RC-2 Axi-R resistant cells was only over twice that of wild-type cells, meeting the basic criteria for drug-resistant models. The development of resistance took one year, and despite multiple attempts, further increasing the IC50 value proved unsuccessful. As a second-generation VEGFRi, Axitinib effectively inhibited tumor growth, while this mainly occurred at higher concentrations, leading to significant cell death and resulting in only a modest difference between the initial and final concentrations.

Another challenge was the difficulty in obtaining clinical samples from drug-resistant patients with advanced RCC, as these patients typically received only palliative treatment. As a result, no clinical sample support was available for this study. Previous published studies using patient-derived xenograft tumors have demonstrated that the combined use of VEGFRi and MEKi has better efficacy, which to some extent supports our findings 25, 26. However, no studies have yet explored the underlying mechanisms, which limits the research to a preclinical level. The mechanism we elucidate could potentially promote the clinical application of MEKi in patients with VEGFRi resistance.

Multi-omics sequencing of resistant cells will provide more comprehensive and convincing evidence, which is currently lacking in this study. In future research, we will conduct a comprehensive proteomics study to identify other proteins that may interact with MAP2K2 and SP1 in resistance pathways, further exploring their potential mechanisms. Combined with single-cell sequencing, this approach will help us analyze the heterogeneity of ccRCC cells in response to VEGFR and MEK inhibitor treatments, which will aid in uncovering deeper mechanisms. We will incorporate this aspect into our future research.

## Conclusion

Our study, through CRISPR/Cas9 library screening, identified* MAP2K2* as a common resistance gene to three VEGFRis (Sunitinib, Axitinib, and Sorafenib), with its expression positively correlated with VEGFRi resistance. In the drug-resistant cells we constructed, we observed an upregulation of MAP2K2 expression. Mechanistically, we confirmed a positive feedback regulatory relationship between MAP2K2 and SP1, which continuously activates the MEK/ERK pathway, leading to VEGFRi resistance. Finally, we found that the combined use of MEK inhibitors (MEKi) and VEGFRis can enhance the sensitivity of drug-resistant cells to VEGFRis, with the combination significantly killing the resistant cells. Our findings provide new insights into the mechanism of VEGFRi resistance and suggest that the combination with MEKis may be a novel therapeutic intervention for patients with resistance.

## Supplementary Material

Supplementary figures and table.

## Figures and Tables

**Figure 8:**
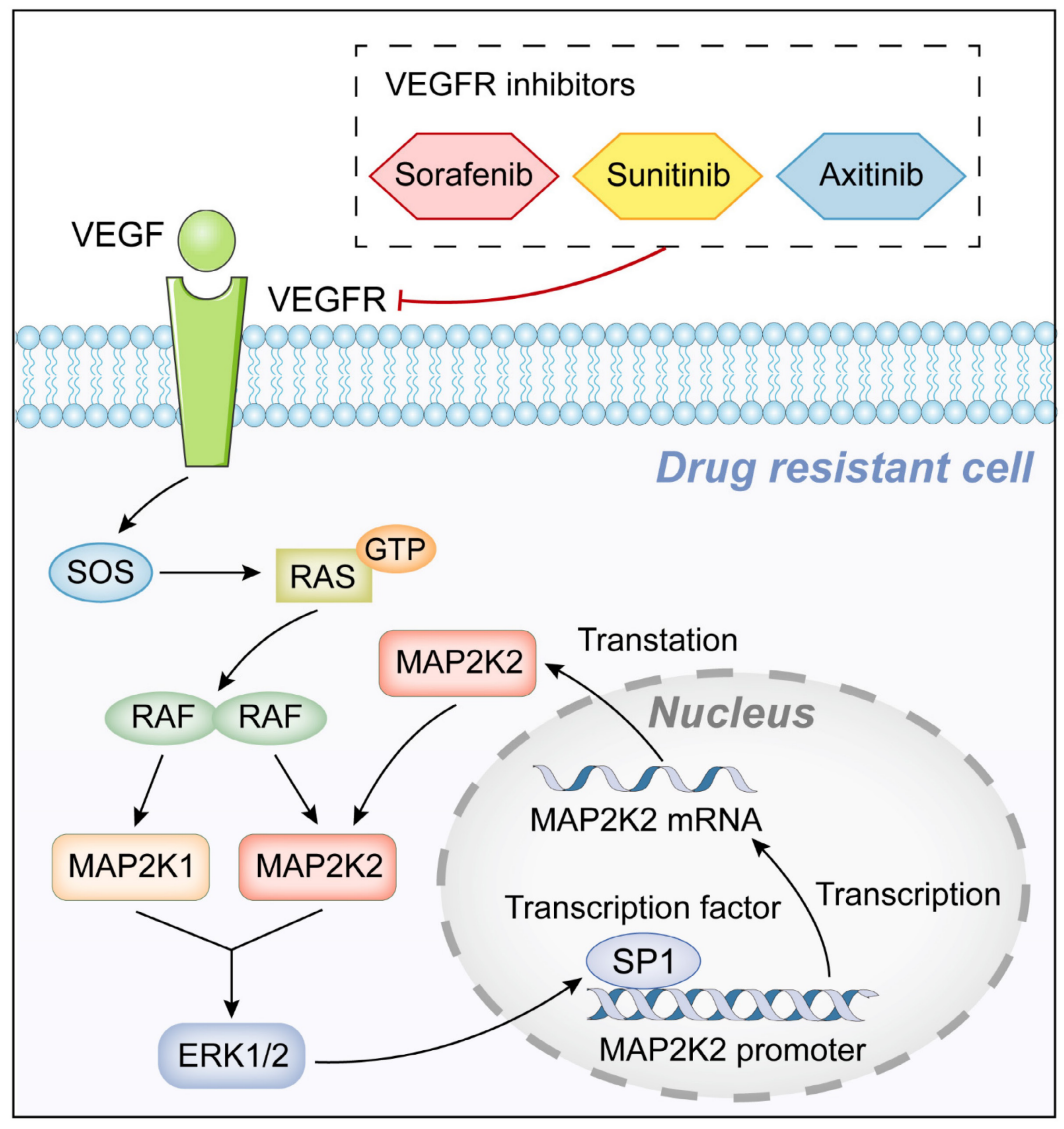
Graphic abstract of this study.

## Abbreviations

ccRCC: Clear Cell Renal Cell Carcinoma
DFS: Disease-Free Survival
ERK: Extracellular Signal-Regulated Kinase
IC50: Half-Maximal Inhibitory Concentration
MAP2K2: Mitogen-Activated Protein Kinase Kinase 2
MOI: Multiplicity of Infection
OD: Optical Density
OS: Overall Survival
RCC: Renal Cell Carcinoma
RTK: Receptor Tyrosine Kinase
SP1: Specificity Protein 1
TCGA: The Cancer Genome Atlas
TKI: Tyrosine Kinase Inhibitor
VEGFR: Vascular Endothelial Growth Factor Receptor
