# Supplementary material for: The positive feedback loop between SP1 and MAP2K2 significantly drives resistance to VEGFR inhibitors in clear cell renal cell carcinoma
Source: Int J Biol Sci. 2025 Jan 1;21(2):860–73. doi: 10.7150/ijbs.104591 (PMC11705632; doi:10.7150/ijbs.104591)
Supplement: Supplementary file 1 — Supplementary figures and table. [file ijbsv21p0860s1.pdf]

Supplementary Figure

Supplementary Figure 1. (A-C) Statistically significant correlations are observed with Fuhrman grade, clinical stage, and distant metastasis ($p < 0.05$). (D, E) No statistically significant differences are found for tumor size and lymph node metastasis.

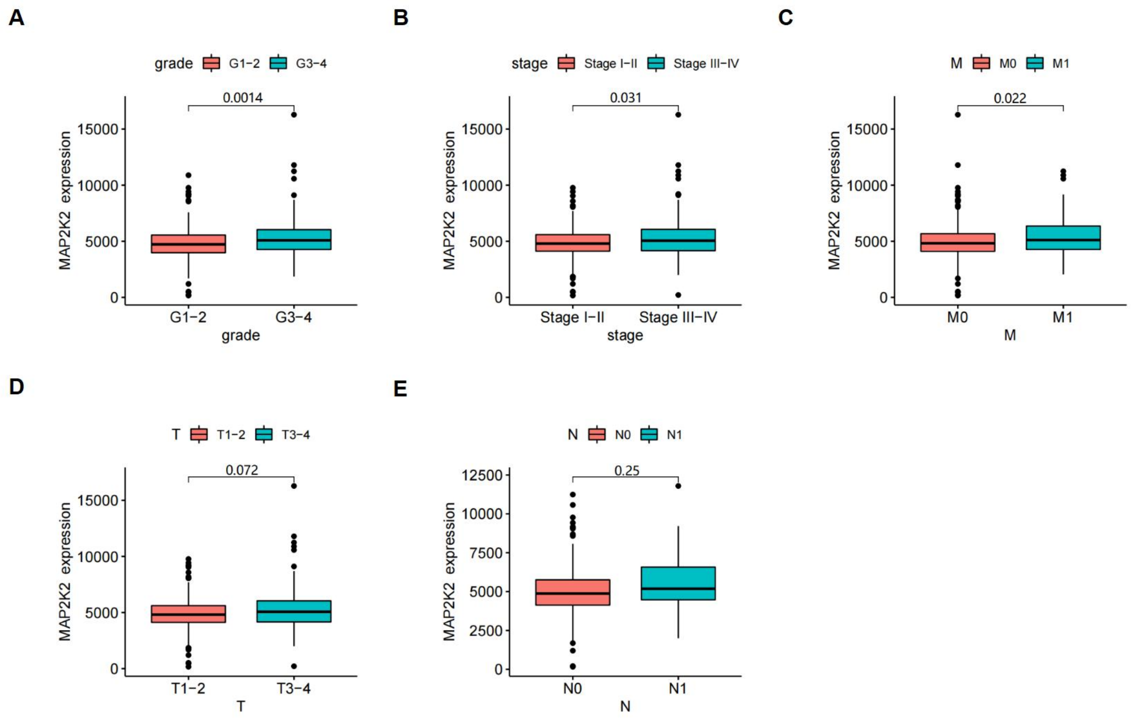

Supplementary Figure 2. (A, B) IC50 curves of Sunitinib, Axitinib, and Sorafenib in 786-O and OS-RC-2 cell lines. Black curves represent NC (negative control) cells, green curves represent cells with MAP2K2 knockdown, and red curves represent cells with MAP2K2 overexpression. Experiments were independently repeated three times, denoted as -1, -2, and -3. (C) Relative mRNA expression levels of MAP2K1, MAP2K2, and ERK in 786-O cells following siRNA-mediated knockdown. Two siRNA sequences were selected for each gene, labeled -si1, -si2, or -si3. (D) Expression levels of MEK/ERK pathway proteins in 786-O cells following siRNA knockdown. (E) IC50 curves of Sunitinib, Axitinib, and Sorafenib in 786-O cells after siRNA-mediated knockdown.

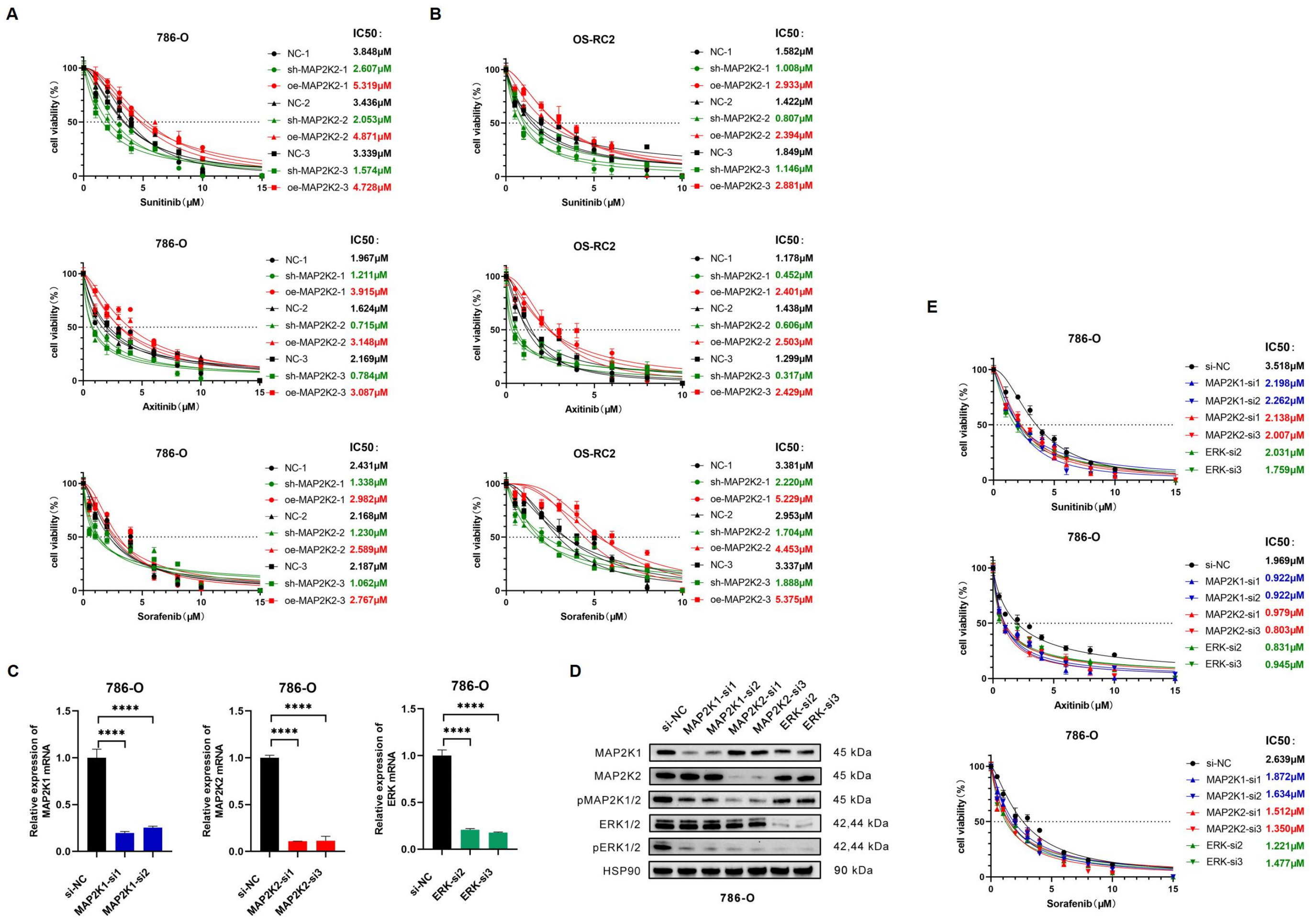

Supplementary Figure 3. (A) The IC50 value of Trametinib of 786-O and OS-RC-2 cells. (B) Assessment of cell survival in drug-resistant 786-O and OS-RC-2 cells exposed to varying drug concentrations, with a color gradient from red to blue indicating increasing cytotoxicity.

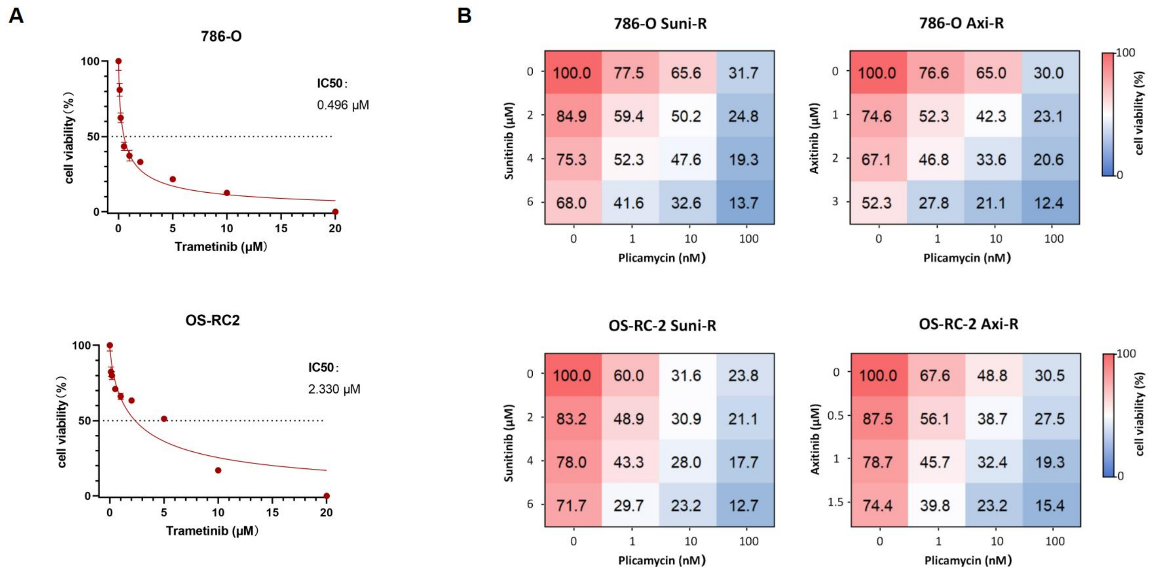

Supplementary Table1 sgRNA sequences of 1811 target genes and control genes in the CRISPR/Cas9 library

UID	sgRNA_seq	Gene
1	AAAS	TCCCTTATCAGGCGTGATGT
2	AAAS	CCCAGACGGCAGCAAAATCC
3	AAAS	GTGGGGTGACCAACCTGCTC
4	AAAS	GCACTCACCAATTTGTGACT
5	AAAS	AATTTGCCCAAGTCACAAAT
6	AAAS	TTTCATTTAGCACCCCAAAA
7	AAGAB	ACCATTCTTCATCACTACAT
8	AAGAB	GCCTTGATAGTGTCTCCTCA
9	AAGAB	AGCAGACATCAATCTATGTG
10	AAGAB	CAGACCAAGATCATCACCTC
11	AAGAB	TGGAACAGAAGATCTTATTG
12	AAGAB	CGCTTTACTCCTGTAGATTC
13	AANAT	CGCGTCCTCGCACATGAGCG
14	AANAT	ACCTTCACGCTCGATCTCAA
15	AANAT	CCTTCATCATCGGCTCGCTC
16	AANAT	GCCTTTGAGATCGAGCGTGA
17	AANAT	GACGCCCAAGACGGAGATGA
18	AANAT	CCGGGCCGCGCTCATGTGCG
19	ABCB1	TTGGACTGTCAGCTGCTGTC
20	ABCB1	GAAGCTAACCCTTGTGATTT
21	ABCB1	CCAAACACCAGCATCATGAG
22	ABCB1	CTAGGTGATATCAATGATAC
23	ABCB1	TGGACTTCCTCTCATGATGC
24	ABCB1	ATCTGGAGGAAGACATGACC
25	ABCB7	TCAGCATGTTTCCCGACATC
26	ABCB7	TTACTAGATCTCCAACAGTA
27	ABCB7	CTGTATTTGGTGCATCACTC
28	ABCB7	CACCTTGTTTACTCTACTCA
29	ABCB7	AACAAAGCAGATAATGATGC
30	ABCB7	GCTGAATTATGAAACTGTGA
31	ABCC12	CGTGCGGTAGTTGATGGCCC
32	ABCC12	CTGGGCCATCAACTACCGCA
33	ABCC12	TGCCGCACTCACCGGCCCTA
34	ABCC12	CACCATCACCGGCGTGAGCC
35	ABCC12	GATGAGTCATATGTCGACAA
36	ABCC12	CCAGAGGACACGCGTGTTGA
37	ABCC4	GGCACTTCGTCTTAGTAACA
38	ABCC4	CTTCCTCTAATCTCCGTTTA
39	ABCC4	TGATGTGAACAAGTTTGATC
40	ABCC4	GAAGACCGCTCACAGCACCT
41	ABCC4	GAGCAATCATAAAGTGTTAC
42	ABCC4	ATGATTGCTCTTGTTAAAGA
43	ABCG2	ACATCTCCAGATAATCCACT
44	ABCG2	GACAGCTTCCAATGACCTGA
45	ABCG2	CGAAGATTTGCCTCCACCTG
46	ABCG2	TATTAGATGTCTTAGCTGCA

47	ABCG2	AGCTGTCGCGGGGAAGCCAT
48	ABCG2	TACAGTGGGATCATGAAACC
49	ABHD6	CCCCTTCCAGGTACTGGCGG
50	ABHD6	GCAAGTTAAGAGGATACACC
51	ABHD6	AACATGTTTGTGATTGCGGG
52	ABHD6	CCTCTTAACTTGCCCATCTA
53	ABHD6	GAGCAGGATGGATCTTGATG
54	ABHD6	CAGAGAATCCGTGGAGCATG
55	ACACA	TGCAATTAGATTCGTTGTCA
56	ACACA	GCAGAATTTGTTACTCGCTT
57	ACACA	TGGCTTGCACCTAGTAAAGC
58	ACACA	GAAGACCTTAAAGCCAATGC
59	ACACA	GATCACTATGTGCCAGTGCC
60	ACACA	CAACAACAACTATGCAAATG
61	ACACB	GATCATTACGTCCCCGTCCC
62	ACACB	GGGTTGGGACCTGTAGCGTC
63	ACACB	CGTTGTCGCCCAGACGCTAC
64	ACACB	TTGCGTGAAAGACGTAGATG
65	ACACB	TACCTGCACGGGGATTCTCT
66	ACACB	CCCTTGTCATAAACATCTTC
67	ACADM	GCCAATCGACAACGTGAACC
68	ACADM	TCCCCCTAATTAGAAGAGCC
69	ACADM	TGCTCGTAAATTTGCCAGAG
70	ACADM	ACTGTAGGAGGTCTTGGACT
71	ACADM	GAAAGCTTACCACAGTTCTC
72	ACADM	GGGGTTCAGACTGCTATTGA
73	ACAT1	TGCTCCATCATTCAGTGTAC
74	ACAT1	AATGCCAGTACACTGAATGA
75	ACAT1	TCCTACAAGGCAGGCAGTAT
76	ACAT1	CCCAATACTGCCTGCCTTGT
77	ACAT1	AAGTGCCGCCAGCACAGCCA
78	ACAT1	TGTATCAAAACCCACTTTGA
79	ACBD5	GAAGAATTGCTGCGTGTCAT
80	ACBD5	TAGGGATGCTTGGAGTTCAC
81	ACBD5	AGGAGTTCTGATATAACCTC
82	ACBD5	TTCTTCAACATATGCAATCA
83	ACBD5	TGAAGTTTACTGTGATTCTA
84	ACBD5	GATCATGTTGAAGATGTTAC
85	ACE2	CAAGTGAACTTTGATAGAAC
86	ACE2	ATGAGCACCATCTACAGTAC
87	ACE2	TGCTGCTCAGTCCACCATTG
88	ACE2	TCAAGTAATAAGCATTCTTG
89	ACE2	CACAGTCAAGCTTCAGCTGC
90	ACE2	TGTGGACTGTTCCTTTAAAA
91	ACER2	TATCGCCGAGTTCTACAACA
92	ACER2	CCGCACCGTGTTGTAGAACT
93	ACER2	CTACTTAATCTGGACTCTTT
94	ACER2	CCATGCAACCCTTAGTTTCT
95	ACER2	GCATCTGACCCAAGAAACTA

96	ACER2	ACGAAACAAGCACATGCAGA
97	ACHE	CCCAGCGACTGATGCGATAC
98	ACHE	CCACAATGTCGTGTGCCCCG
99	ACHE	GGCTCAGCAGTACGTTAGTC
100	ACHE	CCCACAATGGCCCCCGTACA
101	ACHE	GGCCGCAGACACGCTCGACG
102	ACHE	GTTCGACCACTACAGCAAGC
103	ACLY	GACCAGCTGATCAAACGTCG
104	ACLY	GAGAGCAATTCGAGATTACC
105	ACLY	TTCCACGACGTTTGATCAGC
106	ACLY	ACCTACCGATGTGCTCCCGC
107	ACLY	AACTTCCTCCTCAACGCCAG
108	ACLY	TTGTGACTTCGTGCTCCTTC
109	ACOT4	ATAAGGAATGCTCTCGTAGG
110	ACOT4	CCGCGCACGGCAATGCGCAC
111	ACOT4	CGCAGCGTAACCCGCTGCTC
112	ACOT4	TTATATCCACAATGTCCACG
113	ACOT4	TGAAGATCTCCCCAATAACA
114	ACOT4	GGATATGTTGTCCATGTTAT
115	ACOX2	GCCGAGCTATCAACCGGATG
116	ACOX2	TGATAGCTCGGCGCCTGGGT
117	ACOX2	AGGCGCTCACCTTAGCAGCC
118	ACOX2	CTCCATGACGCCTTCCTGTC
119	ACOX2	CTGCCACTCACCGGATCAGG
120	ACOX2	TCTGGTTCCAAGCCTCGTGC
121	ACTB	CAACCGCGAGAAGATGACCC
122	ACTB	TCCTTCCCAGGGCGTGATGG
123	ACTB	AGCCATGTACGTTGCTATCC
124	ACTB	CGTACAGGGATAGCACAGCC
125	ACTB	CACCTTGATCTTCATTGTGC
126	ACTB	CTCTCAGGCATGGAGTCCTG
127	ACTL6A	GTTGAAGGACATAGCCATCG
128	ACTL6A	TGCCAAGACCTCGTAACCTG
129	ACTL6A	TCGTTCTACTGGGCTGATTT
130	ACTL6A	GTACCGAAGCTTGAAAATCC
131	ACTL6A	TCTCAGGAAGCTGTTCGTGA
132	ACTL6A	TTACTCACAGTGTGTTATCC
133	ACTL6B	ACTATGAGCGGGGGCGTCTA
134	ACTL6B	ATGACCTCCGCTCCATCCCG
135	ACTL6B	GTCATGTCGCCCCTCAAGAA
136	ACTL6B	GGCTCCTTCTCAGTCCGCGC
137	ACTL6B	TCCCCGCACAGATGAGGTGG
138	ACTL6B	GCAGGGCATTGGTGTCGATG
139	ACTN4	GCATGAGCTTGAGCCCGTCT
140	ACTN4	GGAGTTGCACCATGCCGTGA
141	ACTN4	TGCACATTGACGTTCTTATA
142	ACTN4	CAACAATGTGAACAAAGCGC
143	ACTN4	ACTCTCATCTTCCCCCGCTC
144	ACTN4	CATCCTTAGGTTCGCCATCC

145	ACTR5	TGAGCACAGCTACATCGCTG
146	ACTR5	AACTGCAAGCGCATCAATCT
147	ACTR5	TTACCTGCACATATAGCAGT
148	ACTR5	CCAAAGAACTCGATGTGCAG
149	ACTR5	TGTCTACCACATCCACCTCG
150	ACTR5	CCACTGCACATCGAGTTCTT
151	ACTR6	GGAGCTTACAACGCCAAAAT
152	ACTR6	TGAGATGACGACCTTAGTGC
153	ACTR6	ACTCTTAACCAATCATCTAA
154	ACTR6	AGATGTATGCTATGTGTCTC
155	ACTR6	TCCCCTTGCAGGATAAATGT
156	ACTR6	TGTTAGGCAGCTACATGTTA
157	ACTR8	GCGGCCCATCGTGCCCGCGC
158	ACTR8	CGCACCTCTTGCAGCGACTC
159	ACTR8	CATCACCCTGAGTATTTAGT
160	ACTR8	TCTTCTCCTACTAAATACTC
161	ACTR8	AAATGGTGGATCAAGCAATA
162	ACTR8	CGAACCTGTTCAGGGGACAC
163	ADA	CAGGCTTGATGGATCCGTCT
164	ADA	GCTCACTTTGGGCTTGTCGA
165	ADA	GAACTGCATGTCCACCTAGA
166	ADA	AGGCATGTAGTAGTCAAACT
167	ADA	AGCCCCTCTGCTGTGTTAGC
168	ADA	TTACCTGCCATAGTATAAGA
169	ADAM8	CGGCTACACAGAGACCTATA
170	ADAM8	CTGGGCGCGATGATGCTGCC
171	ADAM8	CCGACCCATACCTGCCCGCG
172	ADAM8	AGGACGTAGCTCACCCTCTC
173	ADAM8	CCACTCACCTGTTCTTCCGC
174	ADAM8	GACCCGGCCATGCGCGGCCT
175	ADAMTS12	ACCACCCGCACATCGTTTAC
176	ADAMTS12	CTCGGACTGGACCCACCACG
177	ADAMTS12	GTACTACAGAATTTCTCACG
178	ADAMTS12	TGCCTCCTGTAAACGATGTG
179	ADAMTS12	CCCGCCATAGCTCTTGCTTC
180	ADAMTS12	CATTCTACTCGAAGAAGAAG
181	ADAMTS14	ACACTTGTCATCCGCCTTCA
182	ADAMTS14	AAGCCGGACCTTCACCGCCA
183	ADAMTS14	GGCGGTGAAGGTCCGGCTTG
184	ADAMTS14	CCAGCACTCACCAATGCGGT
185	ADAMTS14	ACCTCCTTGTCACAGCCGAC
186	ADAMTS14	ACTTCTCCAGTGCCTCAATC
187	ADCY6	ACGCGTTGCGCTCGCCACCA
188	ADCY6	GCAGTGTTGCCCGAGTGATG
189	ADCY6	CCGCAGCCAGCTTGTCAAAC
190	ADCY6	CCTGACTTACTTGTCTTTGC
191	ADCY6	CCCTGAATGAGCTCTTTGCC
192	ADCY6	GCAACACTGCAGTACCTGAA
193	ADD1	GACCAGAGTGAACTCCGAGC

194	ADD1	CATGGCACCAGACCTTCGCC
195	ADD1	GAATGGTGATTCTCGTGCTG
196	ADD1	CCTCTTGCCAATCTCCCCGG
197	ADD1	GAAAGCGCCTCCGGGGAGAT
198	ADD1	TGAAGTGACTGCATCCAGTT
199	ADH5	CTGATAGATCATTGCCACTG
200	ADH5	GTGCTCATTGAGATGACCGA
201	ADH5	ACTTCAGGTTATCAAGTGCA
202	ADH5	AAGATACTAACCTTATCTTC
203	ADH5	AGTCGGATTGGCAGTTATCA
204	ADH5	ATCGGTGTGGCAAACCGCAG
205	ADH6	TCCATACAGGTTGTGGCCAC
206	ADH6	GCTGGAATCGTTGAGAGTAT
207	ADH6	GATGCAAAGCAGCCATACTC
208	ADH6	TGCACCAGGCTTCCAGAGTA
209	ADH6	GAGGTACATTCTCCACACTG
210	ADH6	ACACTCTTTCTGCCACAGTG
211	ADM5	TCCAGTATATGATCTCCGCC
212	ADM5	CCTACAGCTATGGGCGCCGC
213	ADM5	CCCCTTGGGCGGAGAAGGCG
214	ADM5	CTCCTCGCCTTCTCCGCCCA
215	ADM5	GGTGGGTCCGGCATACGCCC
216	ADM5	CTTCCGCCTCGCGCCACCGG
217	ADRM1	CGCCTAGCGCAGAGAGTTCG
218	ADRM1	CGCTTGAACTCACAGTCGTC
219	ADRM1	ACATCCGCGTTGGCGAGGAT
220	ADRM1	ACCAGCCCTTTCCGCTTATC
221	ADRM1	CCACCGTGACTCCGGATAAG
222	ADRM1	CACGAACTCTCTGCGCTAGG
223	AFF4	GGGCAACTTACAACTTTGTA
224	AFF4	GCAACATGAACCGTGAAGAC
225	AFF4	GAGTCTCAGCAGTCCAATTT
226	AFF4	TTGTTCTCCAGTGCCAAAAT
227	AFF4	TCTTTCAAGGCATCAGCTTC
228	AFF4	CTTTCATACGCAGCACATTC
229	AFG3L2	ATGGGCGGACTCTTCAGTGT
230	AFG3L2	TGAAGAGTCCGCCCATCCCT
231	AFG3L2	AGTTTCCAGATTCCGTTCAA
232	AFG3L2	GTCTACATTGCTGAAAGTGA
233	AFG3L2	TTCTCTCCCATAACTTCTTT
234	AFG3L2	TAGTGAACCTAAAGAAGTTA
235	AFM	AATTTCTTACCTATATCCCG
236	AFM	TAACCCTGCCCACACAACCT
237	AFM	TGCATTTGCTCAGTATGTTC
238	AFM	ATACTGAGCAAATGCAATGA
239	AFM	ATGAAACTACTAAAACTTAC
240	AFM	AGAATACAAAGACAGATGTA
241	AGAP5	TAGCCTTTCTGCCAATCCAG
242	AGAP5	CATCTGTTTGAGAGTTCCTC

243	AGAP5	TAACCCTTCTGCCAATCCAG
244	AGAP5	CAGAGGAACTCTCAAACAGA
245	AGAP5	TTGTCATTGTAAGATAATGC
246	AGAP5	TATTGTGCTTGCCTCTGGAT
247	AGAP9	CATCTGTTTGAGAGTTCCTC
248	AGAP9	TAACCTTTCTGCCAATCCAG
249	AGAP9	TAACTCTTCTGCCAATCCAG
250	AGAP9	TATTGTGCTTGCCTCTGGAT
251	AGAP9	TTGTCATTGTAAGATAATGC
252	AGAP9	CAGAGGAACTCTCAAACAGA
253	AGGF1	TCTATAGTATTTCCACTAGT
254	AGGF1	GACGAAGCCATTACCAGTGA
255	AGGF1	AGCTAATGATGAGCCTTCTA
256	AGGF1	CTCTATTATGATCCTTCCAC
257	AGGF1	ACAGAAGATACCTCCTTAGA
258	AGGF1	AGATTCTTCTGCAACAAATG
259	AGO4	GTTAACATACGTTTGATGAC
260	AGO4	CCAGCACCAATGCTGCAATA
261	AGO4	GTCTAGTCACATTACAAACT
262	AGO4	TTAGGTGAAGAGCAACAGTA
263	AGO4	ACCGGGTCCCAGCGCCTCCA
264	AGO4	CGCCGCCGCCATGGAGGCGC
265	AGPS	TACCTTAGGTACCCTCTTAG
266	AGPS	CTTGGCCATAAAACTATATC
267	AGPS	GATCGAGTATTTAGAGCTCA
268	AGPS	AGGTGTATCACTAGGATTTA
269	AGPS	AAATACCCTTGGAGTAAATG
270	AGPS	CTAGGCAAGAAGTTATGAAA
271	AGTR1	CTACAAGCATTGTGCGTCGA
272	AGTR1	TCTCAGCATTGATCGATACC
273	AGTR1	TCAGGCCCAGCCCTATCGGG
274	AGTR1	GTAGAAACACACTAGCGTAC
275	AGTR1	ACACAGCTATGGAATACCGC
276	AGTR1	TCATTGGGTGAACAATAGCC
277	AGXT2	AGACGGTGCTTGTATGCCAC
278	AGXT2	TATGTTGTTTGAGTGCGCCC
279	AGXT2	AGGATACTCACGGGTGGCAA
280	AGXT2	CCTTGGCTACAACCGTGTCC
281	AGXT2	AAAAGCAGCTCGGCCGCCTG
282	AGXT2	TGGCTCAGAAGCCAATGAGC
283	AHNAK2	CGGATGAGGGCATTCGACCA
284	AHNAK2	GGTCGAATGCCCTCATCCGC
285	AHNAK2	TTTCAGGATGAGTCGTCCAG
286	AHNAK2	GATGTGCGACTGCTTCCACA
287	AHNAK2	TCCTGGTGGAAGCGAGATTC
288	AHNAK2	GAGGGGCGCACCGCTGCCGG
289	AK1	ATTGTCGTCCACACGCCCGC
290	AK1	CTCCACGTTTCAAGAGCCGC
291	AK1	AGTATTGACTTTGGCCACCA

292	AK1	TACCCGCGGGAGGTGCAGCA
293	AK1	ACCCACCACAAAGATGATCT
294	AK1	GAAAACCAAGATCATCTTTG
295	AK3	CATCCAACAGCCAGCTATAC
296	AK3	AGATGATGTCATGACTCGGC
297	AK3	AACAACGCCTTACTGCTCGC
298	AK3	TAGGGCATTGATGACCTGAC
299	AK3	AAACTCACTGGTAATATTCC
300	AK3	TGAATTCAATGTTATAGACT
301	AKAP1	ACGGTCATCTGTGCCGCCCC
302	AKAP1	CTACGAGGAGACCAACGAAG
303	AKAP1	AGAAGGTCGACCTCATCATC
304	AKAP1	GTGTGTGCTGCTGCACGAAC
305	AKAP1	CATCACCGTGGAGGTCATTG
306	AKAP1	CCTCGATCTCCCAGATGATG
307	AKAP2	TTCTATTCACCCCCGCATAA
308	AKAP2	TCAACAAGCCATAGCCGAGC
309	AKAP2	CTTTCTTCGATTAACCCGTG
310	AKAP2	TAATCGAAGAAAGAGCGCAC
311	AKAP2	GCCTCTTCAGGGGCTAAGTC
312	AKAP2	TTCTAAGTTACTGTCTTGCA
313	AKAP6	CCTGAGCTTATCCGAAGTGT
314	AKAP6	CCATTCCTAGTGACGTGACT
315	AKAP6	TTACTCTGTCAACGTGATAG
316	AKAP6	CCAGCACTGAGACTCGAAGC
317	AKAP6	GTGTCTCCAGATACAGTTTA
318	AKAP6	ACTGTATCTGGAGACACACT
319	AKAP8L	ATCTAAGCGCTGGTTAATTC
320	AKAP8L	TGTTGTCCGAGCAGTCCGTG
321	AKAP8L	AATTAACCAGCGCTTAGATA
322	AKAP8L	CGAGTATGTCGACTGCAAAG
323	AKAP8L	CACTTTGCAGTCGACATACT
324	AKAP8L	AGATAGCAAAGCCACCCGCA
325	AKAP9	TCCGACTCATCTAGAGATGA
326	AKAP9	AGAAATAACCAGTCATGAGC
327	AKAP9	ATAATGAGAACTCTACATAG
328	AKAP9	TCCATCATCTCTAGATGAGT
329	AKAP9	TGAACTGCAGTCATCTGCTG
330	AKAP9	AGCAGATGACTGCAGTTCAG
331	AKR1C1	GCCAGCTTCAATTGCCAATT
332	AKR1C1	ACCAAATTGGCAATTGAAGC
333	AKR1C1	CTTATTACCATGGTTCTTCT
334	AKR1C1	GATCCCACCGAGAAGAACCA
335	AKR1C1	CTGCCTACCTTTACAGACAC
336	AKR1C1	CAATTCCCATCGACCAGAGT
337	AKT1	TGTGCCGCAAAAGGTCTTCA
338	AKT1	TCACGTTGGTCCACATCCTG
339	AKT1	GCAGGATGTGGACCAACGTG
340	AKT1	GCCCGTCCTTGTCCAGCATG

341	AKT1	GCCTGGAGTTCTGCAGGACG
342	AKT1	ACCGCGTCCTGCAGAACTCC
343	AKT1S1	CGCGCATGCTCGCCGCGATG
344	AKT1S1	AACGGCGCGCTGCCTCCGCC
345	AKT1S1	TGTGGGCCAGTGCGATGTCG
346	AKT1S1	AAGTCGCTGGTGTTAAGCCG
347	AKT1S1	CGGCGGGGGTCTCCTCGCTC
348	AKT1S1	TCAGACCCCGAGAGTACAGA
349	AKT2	TCTCGTCTGGAGAATCCACG
350	AKT2	CATCGAGAGGACCTTCCACG
351	AKT2	TTAAACAACTTCTCCGTAGC
352	AKT2	TCGCCAACAGCCTCAAGCAG
353	AKT2	CAATGATGACTTCCTTCCGC
354	AKT2	GACCCCATGGACTACAAGTG
355	AKT3	AGAATGGACAGAAGCTATCC
356	AKT3	TAAGGTAAATCCACATCTTG
357	AKT3	TTGCCACTGAAAAGTTGTTG
358	AKT3	GATGTTACCATTGTGAAAGA
359	AKT3	GCAGTCTGTCTGCTACAGCC
360	AKT3	AACCCAACCTTCTTTCACAA
361	ALDH1A3	CGTCCCGGAGCAATCTGAAG
362	ALDH1A3	AGCGTCCGCACACACGATGC
363	ALDH1A3	CATATTTGCTCCCGAGTTGA
364	ALDH1A3	TACTCACGGCCAAGGTGGCG
365	ALDH1A3	CGCTCAGCCCGACGTGGACA
366	ALDH1A3	CTACATGTAACCCTTCAACT
367	ALDH3A2	GTCCGACAGGCGTTCCTGTC
368	ALDH3A2	CGTGCGTACCTTGCACAGGT
369	ALDH3A2	TGGTGCTGATAATCGGAGCT
370	ALDH3A2	TCAACATCGGTAAGTACTGT
371	ALDH3A2	CGATGTTGATCCTAAAACCA
372	ALDH3A2	TGGCTGAATGGTGAGAACGA
373	ALDH7A1	CATTAGGTTATGGATCGCCC
374	ALDH7A1	CCTTTCTAGGATAATAGCCA
375	ALDH7A1	GCAGAATCTTTCGCTGGCTT
376	ALDH7A1	CTGGAGGACAACAAGCTGCC
377	ALDH7A1	CCAAGACCTGTCACAATTGT
378	ALDH7A1	TAGCATCTTTACCAAAGATC
379	ALDOA	TGTGCGGCGCAGCGCTGTGA
380	ALDOA	ACGGAGCTGACTTCGCCAAG
381	ALDOA	CCTTGCCAGGTGCCACGATG
382	ALDOA	CTTCAGCACACAACGCCACT
383	ALDOA	CCAACAGGTAGACAAGGGCG
384	ALDOA	GGTCAGTGCTGGATATTGGT
385	ALDOB	GGTACCTATTGTTGAACCAG
386	ALDOB	TCCATCCAGCCTCGCTATCC
387	ALDOB	TATCCACAGTTAGACCAAGG
388	ALDOB	ATTACCTCTGGTTCAACAAT
389	ALDOB	TAGCGAGGCTGGATGGACAC

390	ALDOB	AATATCCTTACCTTGAATGG
391	ALDOC	GAACCGCCGGCTGTACCGCC
392	ALDOC	CCACGACGATGCCCTTATCC
393	ALDOC	CACGTTGGCGTTCTCCAGAA
394	ALDOC	TCCCATCAAGTATACCCCAG
395	ALDOC	CTCCTCTGGGGTATACTTGA
396	ALDOC	TTTCAGCACACAGCGCCACT
397	ALYREF	GGGACTCACTCTCGCATTAT
398	ALYREF	AGTCTCAGACGCCGATATTC
399	ALYREF	CGTCACAGATTGACGCACAG
400	ALYREF	CAAAACAACTTCCCGACAAG
401	ALYREF	TTCCTTGCTAGCGTAAACAG
402	ALYREF	GACGCCGTTGTACTGCTTCA
403	AMH	TCCGACCGCAACCCGCGCTA
404	AMH	AGACGCTCACGCGCCTGGTG
405	AMH	ACGACCACCGCTGCTTCACA
406	AMH	GTTAGCGGTGGACCGCCCTG
407	AMH	CCACCGCTAACACCAGGTAG
408	AMH	ACTCCCGGCTGAGTACCGCC
409	AMOT	CATATGCCTCCGAGACGCGC
410	AMOT	GACAGAAATCCAGCGCGTCT
411	AMOT	AGATCCTCTCAGACGAGAAC
412	AMOT	AGAGGATCTCAACCATCTGC
413	AMOT	CTTCTCTGCAAGCTGCTTGT
414	AMOT	CAACAAGCAGCTTGCAGAGA
415	ANK2	TGGACTCAACGCTCTCCATC
416	ANK2	CGCTCTTCACATTGCATCTT
417	ANK2	CAATATTAATGCACAGTCTC
418	ANK2	AATGACCACCATGTTGCAAA
419	ANK2	CTTAGTGGCAGAATCCACAG
420	ANK2	CTGAGACTGTGCATTAATAT
421	ANK3	TGGTTTCACGCCATTGTATA
422	ANK3	AGCATTGCACATCGCATCTT
423	ANK3	TCCTGGGCTGCCATATACAA
424	ANK3	TTATTCACGAAGCTCTAGAA
425	ANK3	CTGAGATTGTGCATTGACAT
426	ANK3	GAAGAAACTTGACAACTTCC
427	ANKFN1	ACGCAGCCAAACGCCTGTAC
428	ANKFN1	GTACAGGCGTTTGGCTGCGT
429	ANKFN1	GGTGTCAAGCCCTCGCTGTT
430	ANKFN1	GATTCGCTTGCTTTGCACAG
431	ANKFN1	CCTCAACACACCTAACAGCG
432	ANKFN1	TTACCAGTTTATGCTGTTCG
433	ANKRD13B	TGGAGATTGACCACGACCGC
434	ANKRD13B	CGCACGGCGCAGACGTGGGC
435	ANKRD13B	CCACGTCTGCGCCGTGCGCC
436	ANKRD13B	TGCAGCTGGTGCTTCGGTAC
437	ANKRD13B	TCTGGCAGACACAAGCGCCG
438	ANKRD13B	ACTACCAGCGGGTGGTGAAG

439	ANKRD30A	CACATCTACGAGATTTATAT
440	ANKRD30A	ATAAATCTCGTAGATGTGTA
441	ANKRD30A	GTCTTTGCTGCAGATATATG
442	ANKRD30A	GAAATGTTACTACTTCCTCA
443	ANKRD30A	GAACATTATGCTGTTACTTG
444	ANKRD30A	ATGCAGTTAATAAGTATAAA
445	ANKS4B	CGAAAGATTTAGATCTCGCT
446	ANKS4B	GAATCCATTCTCAATCGTCC
447	ANKS4B	CACATTCGGGTCACTATCTA
448	ANKS4B	CAAGCGAGATCTAAATCTTT
449	ANKS4B	TGTGGTCGATGCCACGCCCC
450	ANKS4B	GATAGTGACCCGAATGTGCC
451	ANKZF1	GAGAAGTTGAGCCGACCCCC
452	ANKZF1	GTCTTCTGATCCCGAGATGC
453	ANKZF1	GGGAACATTATAAGCTTGAC
454	ANKZF1	CATGAGCACCACGCAGTCTC
455	ANKZF1	TAACCTAAAGCAACGTCTCA
456	ANKZF1	CTACAGAACCTGCAAAGTAG
457	ANO4	CATCATCTGAAGTACTAGTC
458	ANO4	AGAGAATCGTCATCTTTGCA
459	ANO4	ATCACCTGGGTGGAAGACTT
460	ANO4	AAAATGGAGGCAAGCTCTTC
461	ANO4	GAAAGAAACATTAGAGCAGA
462	ANO4	CCATTTGATTTGTTTCTTTC
463	ANP32B	ACAGGTTCGAGAACTTGTCT
464	ANP32B	GGACGACGATGAAGTCAGTG
465	ANP32B	CATCTGAGTCAGGTGCTTCC
466	ANP32B	TGGCTATGACCGAGAGGACC
467	ANP32B	GTTACTTACCAAAGGTTCCA
468	ANP32B	CAAGCTGCCTAAATTGAAAA
469	ANP32E	TCTCAGTACAGTAGAAGCTC
470	ANP32E	CTTACCTACCTCAATCTGAG
471	ANP32E	TTGTGATCTCACAGTTAAAC
472	ANP32E	TCAGGAGGATAATGAAGCGC
473	ANP32E	ATTTCTGAGTATGGCTAATG
474	ANP32E	GGAAAATGAAGCTGGTCCAC
475	ANXA8	CCACTCACCGATCCCCTTCA
476	ANXA8	TCTCTTGCAGATTGAACAGG
477	ANXA8	CTTGCCGAACTGAGCCTTGA
478	ANXA8	CCCTGCAGGGACCAACGAGC
479	ANXA8	CATGGCGTCATGCAGCTCCT
480	ANXA8	ACCTTGAAGTCTGAGCTCAG
481	AOX1	CCTCTTCCAGTTCGACTCAC
482	AOX1	TGCACGGTACCTTATCCTCT
483	AOX1	GCTGCCGTCACCACAGTAGA
484	AOX1	CACCTGAACAGGATGAATTC
485	AOX1	ATACGCTTCTTCCTCAAATA
486	AOX1	CAATGCTGTTGCCTTATTTG
487	AP1G2	TCTCAAAGTGTTTGAGCGTG

488	AP1G2	CTGAGGCACAGTCAGCCCGT
489	AP1G2	TTGCATCATGGCTCGCACAT
490	AP1G2	CAGAATCCTCAATCCTAACA
491	AP1G2	CCTGGGGGCCATGCTTCTAT
492	AP1G2	CACCCTCTTGTCTGTGAATC
493	APBA2	CAAAGATGATCCCGTCGATG
494	APBA2	CATCAGAATGATGCAAGCGC
495	APBA2	GGAGGCGCCGGTGTTCCCGT
496	APBA2	GAACGACTGCCATGGCCCAC
497	APBA2	CAGCAGCGCTCTGATCTCAA
498	APBA2	TTTCCAGGTTTGCAATGGTC
499	APC	GGATCTGTATCAAGCCGTTC
500	APC	GATTTATTAGAGCGTCTTAA
501	APC	AAAATGTCCCTCCGTTCTTA
502	APC	GAAGTACTTAAACAACTACA
503	APC	TGATCTTGACAAAGAAGAAA
504	APC	AGAGTGCAGTCCTGTTCCTA
505	APCDD1L	ACCTTCACCGTGTATGCCGC
506	APCDD1L	CGTGCCCCTGGTGTAGCGGC
507	APCDD1L	AGGGGTCCTCGTAGTAGAAC
508	APCDD1L	ATCCTGCCTCCACGCCTTAA
509	APCDD1L	GCGGGTCTGGTTGAGGCGCC
510	APCDD1L	GCTCCCCTACGCTTGCGTCC
511	APOM	ACATGCGGATGGTAGCACGA
512	APOM	TGGATCTACCACCTGACTGA
513	APOM	AAGCCTTCTTATTGACTCCT
514	APOM	AACCCCTTACCTTGATTCCT
515	APOM	CACTGTAGAGGAGAAAGCGC
516	APOM	TCATTCAGCATGATTCCACC
517	APRT	CGCCTGCGATGTAGTCGATG
518	APRT	GGCCGCATCGACTACATCGC
519	APRT	ACGACGACCACCCTCTGTCC
520	APRT	GCTGCGTGCTCATCCGAAAG
521	APRT	AGGACAGAGGGTGGTCGTCG
522	APRT	CTGACAGGCCTAGACTCCCG
523	ARAF	TGTGGTCTACCGACTCATCA
524	ARAF	CGTAGACACTCATGCCATCC
525	ARAF	ACGGAAGACCTTCTTCAGCC
526	ARAF	CTGGGCTCACTGTTGGCGGT
527	ARAF	GGCCAGAGTTTCAGCACTGA
528	ARAF	CACCATCAGTGCTGAAACTC
529	ARHGAP5	CTCTTAGGGTTATGTACCGA
530	ARHGAP5	CTTGCTGCTTCCAATAGTTC
531	ARHGAP5	ACGTGATAACACATCTAAAC
532	ARHGAP5	CTTTCCCAATTTCTACGTGT
533	ARHGAP5	ATCCACCAACACGTAGAAAT
534	ARHGAP5	ATTCAAAAGCAGTTTGATCA
535	ARHGDIG	GTCTGCACCACACCTACCGC
536	ARHGDIG	GCCCCGGAGCCTGTTCCGAC

537	ARHGDIG	ATGAGGCTGTGCCCGAGTAC
538	ARHGDIG	CAAGTACAAGCGGGTGCTGC
539	ARHGDIG	GGCTCCGGGGCCCGTCGTCA
540	ARHGDIG	GTCCACAGGGAGATTGTCAG
541	ARHGEF1	CTGTCTGCATGCCGACATGC
542	ARHGEF1	GAGATGGAAGACTTCGCCCG
543	ARHGEF1	CTGGTTCCCGTCAGCATCAT
544	ARHGEF1	CTCAGCCCCGATGATGCTGA
545	ARHGEF1	GCGGCGCCCAGCCCACCTCA
546	ARHGEF1	GTGGCCCTGCAGTTTGAGCC
547	ARID1A	GCGGTACCCGATGACCATGC
548	ARID1A	ATGGTCATCGGGTACCGCTG
549	ARID1A	CCCCTCAATGACCTCCAGTA
550	ARID1A	TTCAATAGATGACCTCCCCA
551	ARID1A	TGAGCGAGACTGAGCAACAC
552	ARID1A	AATACTCACAGGCAAGCTGG
553	ARID2	TGGCCCTGCACTTGTACAGC
554	ARID2	AGCTGTGGCTTTGGATTGCC
555	ARID2	TACCCGACACACTGTGTTGC
556	ARID2	AGACACATGAAGTTCTGTCC
557	ARID2	AGGTTTCTGAGAAGAATCAG
558	ARID2	CTTTAAAACAGTATTACTTG
559	ARID4A	TCGGTACTTGGCACTGACAT
560	ARID4A	CTCTTAAAGGACCCTTTACT
561	ARID4A	GATGTCAGTGCCAAGTACCG
562	ARID4A	GGTACAAGATGACCAAGTAA
563	ARID4A	GTGTGGTATTATCCTGTTTC
564	ARID4A	TGAGCCTGCCTACCTGACAG
565	ARID5B	GGATGCGTTTCAGCATCGAG
566	ARID5B	CAAAGGTGTCCCGACAGTAC
567	ARID5B	GCTATGCAAATCGGATCCTT
568	ARID5B	CAGAATGTACCCACTTGACC
569	ARID5B	CAGAAATAGCGACCATGGCG
570	ARID5B	AGGCAGTCAACCCTAAACAG
571	ARL6IP1	TCCTCCGATGGGAAAGAGCC
572	ARL6IP1	CTGCTTCTCACCTACCTGAT
573	ARL6IP1	CCACCCAAGCAACCGCAGCA
574	ARL6IP1	CTAGATCCATCTGTTCTGTC
575	ARL6IP1	GAAACCAGGCTCTTTCCCAT
576	ARL6IP1	GCACAAAAACATAACAAAAC
577	ARNT	GTCGCCGCTTAATAGCCCTC
578	ARNT	TGATCAGATGTCTAACGATA
579	ARNT	GACATCAGATGTACCATCAC
580	ARNT	CTCAGCCTATTCACAGAAAC
581	ARNT	TGAATAGGCTGAGCTTTGTG
582	ARNT	GTGGAGGAGCCATTGTCCAG
583	ARNT2	CGCGGCGGTCTTTCATCTGC
584	ARNT2	CCCGGAGGGTTGACCGCCGC
585	ARNT2	CTCTCTCTTAGGCCGGATCT

586	ARNT2	TCCGAGCAAGATGGCAACCC
587	ARNT2	TGGTTATTCTGTTTAGAGGA
588	ARNT2	TAGGTGTGGAAATGCTCCTT
589	ARSF	GGAAGATACCCCATCCGATC
590	ARSF	AGTTTGCGCACCTGATCGGA
591	ARSF	CAAAATCTTGCAGTCCCCGC
592	ARSF	CATCTAGGTATGGTTTCTAG
593	ARSF	ACCATGATTAGGACAATATT
594	ARSF	GCCTAATATTGTCCTAATCA
595	AS3MT	CGCTGGGTCAGACCTACTAC
596	AS3MT	TCGTGACGCTGAGATACAGA
597	AS3MT	CTTCAGCACCTGCCCGTAGT
598	AS3MT	TGAAGCACTTGTTGTTTATC
599	AS3MT	TGATAAACAACAAGTGCTTC
600	AS3MT	TTGAACTGCCAGAAGAAATC
601	ASAH1	TTGCGTCGCCTTAGTCCTCC
602	ASAH1	GGACTAAGGCGACGCAACTC
603	ASAH1	GTGAAAAGCGCGCTGAGACT
604	ASAH1	AGAAAGCTCGCGGGTCCCAC
605	ASAH1	TTTACTCACGTTGGTCCTGA
606	ASAH1	GTTCCTCACTAGAACAGTTC
607	ASB8	CTATGCCGTGCGCCGTAGCC
608	ASB8	CCGGCTATTCTAAAAGTAAC
609	ASB8	CTTCCCACATGATAATGTAG
610	ASB8	GTCAGCATCTGACACCATAC
611	ASB8	CCTTGATGAGTTCCAGTATG
612	ASB8	GCAGATGTGAACTGCACTCA
613	ASCC1	AGAATTAGTTGATCGAGTGC
614	ASCC1	CTCCACCTCGTAGGCATCAC
615	ASCC1	TTGAACACATCAAACGACTG
616	ASCC1	AAAGTAGACCGGAATCCTTG
617	ASCC1	GTAGCATCTCACATGTCTGC
618	ASCC1	CTGGACTAATAGTGAAAGAG
619	ASF1A	GATCACCTTCGAGTGCATCG
620	ASF1A	CTAATTACTTGTACCTATCG
621	ASF1A	AATAAATTCTTGTCCTCGAT
622	ASF1A	GTGGTCACATGCAGTCCATG
623	ASF1A	CAGGTCCTCGATGCACTCGA
624	ASF1A	TCACAAGATTCCACATTAAT
625	ASF1B	TGTTGACGTAGTAGCCCACT
626	ASF1B	GCGGGTCACCCGGGGGTTCG
627	ASF1B	CCAGCTCCAGCGGAACATCT
628	ASF1B	CGAGGCGATGGCCAAGGTGT
629	ASF1B	CTCCTGTCCATGGTAGGTGC
630	ASF1B	CACGTTCAGCACCGACACCT
631	ASIC1	CGTGAGTTCTACGACCGAGC
632	ASIC1	GACGAGACGTCCTTCGAAGC
633	ASIC1	CTTCAGAGACAGCCGCTCGT
634	ASIC1	GGCAGCCACCTCGTCGAGCT

635	ASIC1	GTAGAACTCACGCATGTTGA
636	ASIC1	ACCTCCTTTCATCGACCAGC
637	ASIC5	GGTAACCATCCGCCAAGTGA
638	ASIC5	TCAAACTTCTTTCGCTCAGT
639	ASIC5	ATCGTTACCTTTGGGCTACA
640	ASIC5	TCCCAGCATCAACGAAACCA
641	ASIC5	GTCCATTGAGGTTCAATATG
642	ASIC5	TCACCAAAACTTCAGCATTG
643	ASXL2	TCAGTTCAAGACCCGACATG
644	ASXL2	CACCGCGGGAAAAGCAGCGC
645	ASXL2	GGGGTGAAAGAGCTGTCAGA
646	ASXL2	GACAGCTCTTTCACCCCATC
647	ASXL2	CTTCAAGTTATCCAGAGAGA
648	ASXL2	AAGAATTTCTTTATGACTCA
649	ATAT1	CATGAGTCTGTGCAACGCCA
650	ATAT1	CTCCTATCACTAGTGCATCA
651	ATAT1	ATCCTTGATGCACTAGTGAT
652	ATAT1	TATGACCATTATAGATGAAC
653	ATAT1	TCTCTCTTAGGATGATCGTG
654	ATAT1	CTTGCCCAGTTCATCTATAA
655	ATG4B	TCCTGTCGATGAATGCGTTG
656	ATG4B	TCCTCAACGCATTCATCGAC
657	ATG4B	CTCTGACCTACGACACTCTC
658	ATG4B	CTGAGACCTCAGAGCCCGTT
659	ATG4B	ACTCACCTCGGCCTAGGTGC
660	ATG4B	GGACGAGATCTTGTCTGATG
661	ATM	CCAAGGCTATTCAGTGTGCG
662	ATM	TGATAGAGCTACAGAACGAA
663	ATM	CCTCGCACACTGAATAGCCT
664	ATM	CTCTATCATGTTCTAGTTGA
665	ATM	AAACAATTAAACATCTAGAT
666	ATM	TCTAGATGTTTAATTGTTTC
667	ATP1B1	GTCCTTCCCGTTCAGTGCAC
668	ATP1B1	ATAAAGCTCAACCGAGTTCT
669	ATP1B1	TAATGATCCCAAGAGCTATG
670	ATP1B1	TCATGAACGAGGAGAGCGAA
671	ATP1B1	TGAATTTAAGCCCACATATC
672	ATP1B1	CATATGTACTGAACATAGTT
673	ATP6V1A	TGGAGAGATTATTCGATTGG
674	ATP6V1A	GTAACTTACATTGCTCCACC
675	ATP6V1A	GAGAGTGGGCCACAGCGAAT
676	ATP6V1A	ATGTTACCCCCACGAAACAG
677	ATP6V1A	TTTATCTTCATCGAGTATTT
678	ATP6V1A	GTGCATGGGGTCTCAGGACC
679	ATP6V1B2	CTTCATTAACCGTGATAGTG
680	ATP6V1B2	TACAGAGGGGCAGATCTATG
681	ATP6V1B2	ATGGACAATTTCAGCATACC
682	ATP6V1B2	TACTAGCTGGTTAGATACAT
683	ATP6V1B2	TGGTTCCAAGGCAGTAGTTC

684	ATP6V1B2	GTAATGTAGCCAGTCAAGTC
685	ATP6V1C2	CTGGAAGCATGTCGGAGTTT
686	ATP6V1C2	AACTCGACACCTTTGCTGAA
687	ATP6V1C2	AATTTCTGCCCCTGGCGATA
688	ATP6V1C2	CCTACCTACCTCCGTTTGCC
689	ATP6V1C2	ATCTGTCTAGGTGGGGACCT
690	ATP6V1C2	TCTGAGCCATTCTCCTTATG
691	ATP8B3	CTCCTTGAACTGCCCGTTGT
692	ATP8B3	AGGGGTACTGAGCGAGAACC
693	ATP8B3	TCCTCTTCATCCGTGCCACC
694	ATP8B3	GCCAACAACCGTGCCTACAA
695	ATP8B3	CTGGCCGAGGCTGCCCATGC
696	ATP8B3	GAGAGAAGATCTCCAAGATG
697	ATR	CTTACCAATACAACTGCCTT
698	ATR	CTTCCATGATCCCCGCCCTG
699	ATR	GAGGCCAAAGGCAGTTGTAT
700	ATR	GGATCATGGAAGCCAGCTCC
701	ATR	TTCTGTGTCAATTCATTGAC
702	ATR	ACAACTGTATTATATTCCTC
703	ATRIP	GCGAGACTCACTACATCAGA
704	ATRIP	CAGTTTATTTGCTCGTTCAC
705	ATRIP	TAAAGATAATTTCGAATTAG
706	ATRIP	TTTCCTTGTCACTGAGTGCT
707	ATRIP	GGCATGTCAAAAAATCCTTC
708	ATRIP	CAGTGAACGAGCAAATAAAC
709	ATRX	TCGTGACGATCCTGAAGACT
710	ATRX	ACTATGCAGAGCTTGCCAAA
711	ATRX	ACATACTTTGTTACAATTGA
712	ATRX	ATTCTTCTGATGAGTGTGCA
713	ATRX	CTTGCAATGAATCAAAACAC
714	ATRX	TCAGAAAAATCCAAGTCTTC
715	ATXN1L	CTTACTAAATTTCGCTCTCG
716	ATXN1L	AAGAGTTGTGTCGTCCGCCC
717	ATXN1L	GAAGACGCTACATTAAACGT
718	ATXN1L	GTGCGCAGTGCCGAAGTGAG
719	ATXN1L	CATCTCCCACCTGTAGCCGA
720	ATXN1L	CTAAAGCGCCAACCACCCGC
721	AUH	GCCTGTGATATACGAGTAGC
722	AUH	ATACCAGCACAGAATATCCC
723	AUH	CCATCTATTGCTGCAATTGT
724	AUH	GACTTCACTCCTGATTATTA
725	AUH	TAACTAGGAATTGTGGTGCT
726	AUH	CAGTAAAAATCTTATAAAAA
727	AURKA	TGAGTCACGAGAACACGTTT
728	AURKA	GCTAGTTTACCAGGTGCCGA
729	AURKA	TCTGAGCTGATGCTCCACTC
730	AURKA	CTTTGAAATTGGTCGCCCTC
731	AURKA	CCATATAGAAAATAATCCTG
732	AURKA	TTTGATGCCAGTTCCTCCTC

733	AURKB	GGGCACTTACGTTAAGATGT
734	AURKB	ATTCTAGAGTATGCCCCCCG
735	AURKB	TGACGAGCAGCGAACAGCCA
736	AURKB	GCGCAGAGAGATCGAAATCC
737	AURKB	CGCTCCAATGTCCAGCCCAC
738	AURKB	ACCCATACTGCAGGTGGGCC
739	AVPR1B	GGGCTAAGTGCAGCACGAAC
740	AVPR1B	GTCCAGGTGAGGTAGGCCCG
741	AVPR1B	TCAACAGCCACCTGTTACCG
742	AVPR1B	GTTGAAGCCCATGTAGATCC
743	AVPR1B	GGCCACGGGCCTACCTCACC
744	AVPR1B	GCTGGCGACCGGGGGCAACC
745	AXIN2	CAACCCATCTTCGTTCCGCC
746	AXIN2	TGATCGTCCAGTATCGTCTG
747	AXIN2	CACCAACATAGCGCTACTCA
748	AXIN2	GGGCTATAGCGGCCTACGCC
749	AXIN2	TATCTCACTGTCGTTGGCGC
750	AXIN2	GATATCCAGTGATGCGCTGA
751	B3GAT3	TTCCCTTACCCGAGTGCAGT
752	B3GAT3	CGTTAGATGCGCTGGACCCG
753	B3GAT3	ACGATGACAACACCTACAGC
754	B3GAT3	TCCTGGCACAGGTACTGGTG
755	B3GAT3	GAGCCCATACCTGGCATAGG
756	B3GAT3	CAGCAAAGTAGACGACTCCT
757	B3GNT2	GTTCCTCTACTCCGGCCACC
758	B3GNT2	GGAAGACTCGCACCACCGTT
759	B3GNT2	CCCATTGATGACGTTTATAC
760	B3GNT2	TAACCACCGACGTGACCCTC
761	B3GNT2	TTAATCGCCAGCAACAAGAA
762	B3GNT2	ACTACTGCGAACCTGACCTG
763	B4GALNT4	CCGTACCTCGCCCGTAGCCC
764	B4GALNT4	ACCCCCGTACCGTCCCTCGC
765	B4GALNT4	GCGCACCACCGTGAAGAAGT
766	B4GALNT4	TGCTGCTGAGCTGCGCCGCG
767	B4GALNT4	ACCAGTCCTCAAACACGTGC
768	B4GALNT4	ACCTGCACGTGTTTGAGGAC
769	BAG1	AGGCTTGGTAAAAAAGGTTC
770	BAG1	TGAACCAGTTGTCCAAGACC
771	BAG1	GGTTGCCGGGTCATGTTAAT
772	BAG1	CTATGACCTCTTCAACAACC
773	BAG1	GAAACACCGTTGTCAGCACT
774	BAG1	GGAGAAGATAGCTGACCAGC
775	BAP1	TCAAATGGATCGAAGAGCGC
776	BAP1	CCAAGGTAGAGACCTTTCGC
777	BAP1	TACCGAAATCTTCCACGAGC
778	BAP1	ACCCACCCTGAGTCGCATGA
779	BAP1	TGAAGTCCTTCATGCGACTC
780	BAP1	CGACCTTCAGAGCAAATGTC
781	BAX	TTTCTGACGGCAACTTCAAC

782	BAX	CCATTCGCCCTGCTCGATCC
783	BAX	AGCGAGTGTCTCAAGCGCAT
784	BAX	AGTAGAAAAGGGCGACAACC
785	BAX	TCTGATCAGTTCCGGCACCT
786	BAX	TCCAGCCCAACAGCCGCTCC
787	BAZ1A	TTTCATGTAAGCGCGAACGA
788	BAZ1A	TGTGCTGTGACGGGTAGACC
789	BAZ1A	CTCCCTCTTCGTCTGTTAGC
790	BAZ1A	TTGACTAATATGTATTCGTT
791	BAZ1A	CACTTTATACTTGAATAGTA
792	BAZ1A	TTGTTAAAGCAACACAAATC
793	BAZ1B	AAAGGTACAGTGAGCGCATT
794	BAZ1B	CAGAGAGTATGAAGCCCGCT
795	BAZ1B	GATTGTGAAGATTCATCCTT
796	BAZ1B	GAACTCCAGTCAGATTGCTC
797	BAZ1B	AGTTTCACATCATATTTGTG
798	BAZ1B	CTTCCTACTTCATTAAAAAA
799	BAZ2A	CTCCTTAGAAGTCCCGTTGA
800	BAZ2A	CAGCGTCACTACCATTATTC
801	BAZ2A	GGTGGGACTGGCAAAGATAG
802	BAZ2A	CCATATAGTCCTCCACTCAC
803	BAZ2A	TGAATAGTGGATGACTGATC
804	BAZ2A	GATGTCACTGAAGCCGTCAA
805	BAZ2B	AGTGTCGAGTGCCTTCCCAA
806	BAZ2B	TGGGAAGGCACTCGACACTG
807	BAZ2B	AGCTCTACAATCAACCCATG
808	BAZ2B	AGATGAAGTTGGTGTAGTAG
809	BAZ2B	AGTAATACATCATCTGTAAT
810	BAZ2B	TTGTCAACTCTAGCATCCAA
811	BBOX1	TCAACTTCAATAACGCAACT
812	BBOX1	ATGATGAACACGCATACAAG
813	BBOX1	TGAAGTTGATGCGAACCACT
814	BBOX1	GTTATAAGGTGTACATCACA
815	BBOX1	CACTTACCCCAGGTGGATGA
816	BBOX1	CAAAATCGATGCAAACAATG
817	BBX	GCTGAGCCTGTAAAACGCTG
818	BBX	ACCTCGGCAAACTGAAACAA
819	BBX	TTCTTCAAACACTTCGCAGT
820	BBX	AGGTATCCTCTGGCACATGC
821	BBX	GCTTTGTAGAAGCATCAGAG
822	BBX	TACCTCTGCAAACTGAAACA
823	BCAM	GTTGCGATACCACGTGATCT
824	BCAM	TCGCAACGGGCAGCGCCTGG
825	BCAM	GGGCTACATGACCAGCCGCA
826	BCAM	CGCCCACTACAGCCTGCCCG
827	BCAM	CACAGACAGTGTCCCTTTGT
828	BCAM	TGTGATGGAGGACTCTGCCC
829	BCAP31	ATACTTCCGAATTTCGCGCA
830	BCAP31	CAGATGCCGTGCGCGAAATT

831	BCAP31	CAAGGAGTACGACCGCTTGC
832	BCAP31	CAACAAAGACCTCCGCATAG
833	BCAP31	CACCGATGACCAACAGCACA
834	BCAP31	TTGCGTGCTCCTCCAGCAAG
835	BCAS1	GACAACCCCTGAACCTGCGA
836	BCAS1	GACAACGCGTCTGCTCTGAA
837	BCAS1	CAGTGAGTTCGTGTCCACCG
838	BCAS1	ACGGACTGTGTGTGCCCCGA
839	BCAS1	GGCCTTTAAAGAAGCCCCCA
840	BCAS1	CCTCTAAGTGCTGAACTGTG
841	BCAS3	ACCATCTGTATACTCTTCAC
842	BCAS3	TTCCAGAGAGCAGTCCTACA
843	BCAS3	TTAAAAGCCATGCAGCACAC
844	BCAS3	GCACATTGTGATGAGGACCA
845	BCAS3	AGTCACAACACTTTCCATGT
846	BCAS3	TGAGAAGCCAGTGTGCTGCA
847	BDP1	TTAGCGTGAAGCCGAATGTC
848	BDP1	GATGTGCCCACAGTCGATTT
849	BDP1	ATTCGGTATCTGTCTGAGCA
850	BDP1	CACTGACACTAGACTTAACC
851	BDP1	ATGCTATAAATGAAAGTCAG
852	BDP1	TACGTCATTGGATTATTATC
853	BEND6	AGAAAGGCATTTCCCGAATA
854	BEND6	CAGCCGACTTCGACAGTCTT
855	BEND6	AGCTTTGTCCCTTGAAGTAG
856	BEND6	TATGGCCGAGGCTCTGCTTA
857	BEND6	TCAGTCTGATGCTCTTCCTC
858	BEND6	ACTTCATTCTGATTCATAGC
859	BGLAP	CAATCCGGACTGTGACGAGT
860	BGLAP	CGGGCCGTAGAAGCGCCGAT
861	BGLAP	CTGGCCGCACTTTGCATCGC
862	BGLAP	CTGGCCAGCGATGCAAAGTG
863	BGLAP	GCGAGGTAGTGAAGAGACCC
864	BGLAP	CCTCATACCTGCACCTTTGC
865	BHLHE41	GCTCGCCGCCGAGAACGACA
866	BHLHE41	ATCGCCCATTCAGTCCGACT
867	BHLHE41	CATGAAACGAGACGACACCA
868	BHLHE41	GTCTCGTTTCATGCTCCTTT
869	BHLHE41	ACTGCAGACTCTGGGACATC
870	BHLHE41	ACTGTCAATTTCAGATGTTC
871	BHMT	TTGCGACATCGCCCGACAAG
872	BHMT	CTATGCGAGTGAAGACAAGC
873	BHMT	CTCCAGCATTTAAACGTTCT
874	BHMT	AGTATTTCTGCAACAGTTAG
875	BHMT	CTTGTCTTCACTCGCATAGA
876	BHMT	ATCCTAGAACGTTTAAATGC
877	BIRC6	GTAGTGTATGCCTCGTTTGT
878	BIRC6	ACTCACCAAGGTTCATCAGT
879	BIRC6	GCCCACCTATAGCCTACATG

880	BIRC6	TGTATAGTGAAGCTAACAGA
881	BIRC6	CTTATCAGCATGTTTAGAAA
882	BIRC6	TTCTTACCTTAGCAACTTTG
883	BLOC1S5	CGAGTTTCACCTTGAATAAC
884	BLOC1S5	CCGCATTGTGTCTCTACATT
885	BLOC1S5	GATCACAGACCAGTTATTCA
886	BLOC1S5	ACTCACATCTCTGGAGAACC
887	BLOC1S5	CAGTCTGTAGACTCCAACAG
888	BLOC1S5	TTCGTTCCTGTTCCCTCTGT
889	BOLA2	CATGGCAAGCGCGAAAAGCC
890	BOLA2	TCACCACATGCTCCGCCTCC
891	BOLA2	CTCCTGTAGCTTCCGAGTCC
892	BOLA2	TACCTGTGTCTCTGAAGCAG
893	BOLA2	AAACCCTGACCCCAGACCAG
894	BOLA2	TCTGTCGCTCACGTGCCCAC
895	BOP1	TTCTGGCGTCTCCGACAGCG
896	BOP1	GGACGGAGCCCTTGATGACG
897	BOP1	GCGGCGGTCTGAGCCCGAAC
898	BOP1	GCACGGCGGCGCCGAGCGTG
899	BOP1	AGGCCAGCACTCCTTGCCCG
900	BOP1	CCGCATACTCATCCCCAATC
901	BPHL	CCGTGAAGAGCTTCTTATTG
902	BPHL	TCCATAGCCTCGAGGATCCC
903	BPHL	TTCATTCATAAGCACGTGAA
904	BPHL	CTCGGTAACCTCTGCCAAAG
905	BPHL	CGAAGACAGCATGATATATG
906	BPHL	GTACCCAGACCTAACATCCC
907	BPTF	GTTCCGCAGTACCTCGTAAA
908	BPTF	ACGCTATCTTTCAGATCAGC
909	BPTF	ACCATTAGCTGCCGCCAGAA
910	BPTF	CCAGATGATGACCCTGAGCA
911	BPTF	AGTCAATTAATTCTGCAAGT
912	BPTF	ACACATTATAGAAGAAATTT
913	BRAF	CCCCACCAAATTTGTCCAAT
914	BRAF	GAGGCCCTATTGGACAAATT
915	BRAF	GTTGCTCCGTGCCACATCTG
916	BRAF	GTGCTTTCTTTAGACTGTCT
917	BRAF	TGATACCATAGGTACCTGCA
918	BRAF	TAGCACTGAAAGGCTAGAAG
919	BRCA2	CTGTCTACCTGACCAATCGA
920	BRCA2	ATGTAGCACGCATTCACATA
921	BRCA2	CGATTACCTGTGTACCCTTT
922	BRCA2	GTCTTACCGAAAGGGTACAC
923	BRCA2	ACTTATTAAATGAATTTGAC
924	BRCA2	AAATCAAGAAAAATCCTTAA
925	BRD2	CGACCACTCTCTCTACGCAT
926	BRD2	AGCTGGTTGGTAACTCGTCC
927	BRD2	TCATCGCTTAGCAGAACTAC
928	BRD2	GAGTCAGGCAAGTCTTTGCG

929	BRD2	TGTGGAAACATCAGTTCGCA
930	BRD2	GAAGACGATTTGGCCAAGCC
931	BRD3	CGACGTGACGTTTGCAGTGA
932	BRD3	CAAAGGTCGGAAGCCGGCTG
933	BRD3	CATCACTGCAAACGTCACGT
934	BRD3	CGCAATCAAATTGAACCTGC
935	BRD3	CGCCACGACAGTCGCCCCCG
936	BRD3	ATTATTACCCCCTGCTCCAA
937	BRD4	ACTAGCATGTCTGCGGAGAG
938	BRD4	TCTAGTCCATCCCCCATTAC
939	BRD4	GGGAACAATAAAGAAGCGCT
940	BRD4	GTCGATGCTTGAGTTGTGTT
941	BRD4	ACAGGAGGAGGATTCGGCTG
942	BRD4	TAAGATCATTAAAACGCCTA
943	BRDT	AGCTCCCTGTACCACGTTCA
944	BRDT	TCCTGTTGACGTTAATGCTT
945	BRDT	TTGGGCCGCAGTTTGTGAAC
946	BRDT	CCCAAAGCATTAACGTCAAC
947	BRDT	ATAAGAACAATGTCATCTCC
948	BRDT	GCCACAAGAAGAGCAAGTTG
949	BRF2	CGCGGTTGCCTACTACCAAC
950	BRF2	GAGATTGCCCTCGTCGCTGA
951	BRF2	TACCACTACCTTCAGCGACG
952	BRF2	ACAGTATTTGCGTACCCCTC
953	BRF2	GCTGTCTCGAACAATGCAGT
954	BRF2	AGGGACGTACCTGCTGCAAT
955	BRICD5	TGTGCTGAGCGCCCCAAACC
956	BRICD5	CGTTCCGGGCCACGTCCACC
957	BRICD5	CGAACAGCACCGCCCAGCTG
958	BRICD5	GCACTCACCCCTGTAGGCCC
959	BRICD5	AGGGCTTCTTGGCTCTGCTC
960	BRICD5	GTGAAGACCAAGCCCTCCTG
961	BRSK1	GAAGAACTTTCGGGCCTCCT
962	BRSK1	AGTCCAGCGCAGACACAATC
963	BRSK1	CCTGCCATCCAACGGAGAGC
964	BRSK1	CCCCGACGTCCTAGAGAGCA
965	BRSK1	TTACCACGAGCAGGGCGAAG
966	BRSK1	CATCCTCTTCGCCCTGCTCG
967	BTBD9	TCTTGTGGGACCGAGATAGC
968	BTBD9	ACCTAGCTTAATCTCGATGC
969	BTBD9	GCTCTCAGATCGCACTTTAA
970	BTBD9	TACACACTTACCGAAAATAT
971	BTBD9	GTGCTTACACCAGTTTAATA
972	BTBD9	TCTTCGCCCCTTTACTGCAG
973	BTG2	TTTCCCGAAAAGCCGTCCAA
974	BTG2	CTCCATCTGCGTCTTGTACG
975	BTG2	CCGCGCGCGACATGAGCCAC
976	BTG2	GGTCTTCAGCGGGGCGCTCC
977	BTG2	CTCCTCGTACAAGACGCAGA

978	BTG2	CCCCTCCAAGAACTACGTGA
979	BTG4	GCTACAACTTTCTTCATCGC
980	BTG4	CTGCTATGCACCGGCTGGAC
981	BTG4	CAATCCGAAGAGTATTTATC
982	BTG4	AGAAGCATCCTAAGTGTTAC
983	BTG4	TTCCGAAGGAGATGACCATA
984	BTG4	CACTCTGATTGCCCTTCTAA
985	BUB1	CCCGCCCAGGCAATGTACAG
986	BUB1	AGTTCCTGCAACAACAATAC
987	BUB1	GAAACCCATTTGCCAGCTCA
988	BUB1	AAACAATAACTGATGAATCT
989	BUB1	TTTACTAGAACATTTAATGA
990	BUB1	CATCCCCTCTGTACATTGCC
991	BUB1B	ATGATCCGCCCTTGCCTTAA
992	BUB1B	GCGGATCATGTCCACGCTTC
993	BUB1B	GCGTTTATGCAATGAGCCTT
994	BUB1B	TCCCATTTAGGTATATCAGC
995	BUB1B	CAAGTGAAACACCAATCCCT
996	BUB1B	AGCCAGAGATTGAGAAATCG
997	C10orf90	AATCGTGAACTGCTTCTTGC
998	C10orf90	CCCTCGCCAGCACCCCGCGA
999	C10orf90	TGCAAGGTCAAAGATCTTGC
1000	C10orf90	ACGATTCCCCATCCTCTGAG
1001	C10orf90	TCTCCAGTCTGATGTCACAG
1002	C10orf90	TTGCCTCTGTGACATCAGAC
1003	C11orf87	GCTTCTTCATCCTACGCTTG
1004	C11orf87	AGCGGCAACACGGGTGCCCG
1005	C11orf87	CCAGCCGGGTTTCCTTTGCG
1006	C11orf87	CTTTCCACATCCACAAGCGT
1007	C11orf87	TGCCGCTGCCTACTGCGCCT
1008	C11orf87	TTTGCTTCCCCCAACGCCAG
1009	C19orf73	GAACCATTAGCCTCGTCCGC
1010	C19orf73	TTCGCCCTCAGACGCTCTTA
1011	C19orf73	GGTGCCGCGTGCAGTTCCCG
1012	C19orf73	CCAAACACAGCGCGTCTTTC
1013	C19orf73	CTTCCAAGAGACTAGTCCGA
1014	C19orf73	CAGGGTTCCCCCGGCGGACG
1015	C1orf21	CGCCAAACACATCTCCGTTC
1016	C1orf21	TGAGTATAGGATCAAACCAG
1017	C1orf21	CTGGGTTGATGAACTAATCC
1018	C1orf21	ATGTATTTGACCTCTTCCAC
1019	C1orf21	TTTAAGTCTTACATTTGAGC
1020	C1orf21	TGCCACTGTTCAAAATGAAG
1021	C1QTNF1	GGTGGTGATCTTGTTCGCGC
1022	C1QTNF1	CCGTGTCGAAGATCACCGTC
1023	C1QTNF1	CACGGCGGTCGCCGGGTACA
1024	C1QTNF1	TGAGGAGCTGCCGTCGCCTC
1025	C1QTNF1	GAGTCGTGTGCCCCATGTCC
1026	C1QTNF1	TCAAGATAGTGATGTTGATC

1027	C1R	CTCGCCAATGTTCTTCCCGT
1028	C1R	GGGCTCTTACCTCTATGAGC
1029	C1R	GTGCAGCAGCGAGCTGTACA
1030	C1R	CAGATCCACAGCATTGCTGC
1031	C1R	GAGCTTCACCCTGTATCCCG
1032	C1R	GGATGCTGTAGTTGCAGCGC
1033	C2CD4A	GACCCAAGCGGAGTCGCTCC
1034	C2CD4A	AGTGCTGCATTGCCGAAGCG
1035	C2CD4A	CCCCGGGCCCACACCTACGG
1036	C2CD4A	CCACGAGCCCGCCGTCGTCC
1037	C2CD4A	GGCGCACTTCGTCCTCCGAG
1038	C2CD4A	GCCAGTCTCCGCTCCGCCGA
1039	C2orf40	GTAGTCATCGTAGTTGACGC
1040	C2orf40	CCCGGAGCCCCTACGGCTTT
1041	C2orf40	GCTGCACCTCGGGCCGAGTC
1042	C2orf40	ACTTTAGTCTTAGTTGGAAC
1043	C2orf40	GCCAGGACAGCAGGCCGCGC
1044	C2orf40	CTGATGCTTCAAAAACGAGA
1045	C2orf83	AACCACAGTCATCGTCACGA
1046	C2orf83	ACCCATCGTGACGATGACTG
1047	C2orf83	CCACTTGCCTTAGCGTAAGA
1048	C2orf83	AGTTCATCCATCTTACGCTA
1049	C2orf83	CAAGATCAGGTGGCAGCATC
1050	C2orf83	CAGATGCTGCCACCTGATCT
1051	C3orf35	TGCAATCACCTTGGGTCCAA
1052	C3orf35	GCGGGCTAATGCAGAGCAGA
1053	C3orf35	AGCCTTCCATTGCTCCACAC
1054	C3orf35	AGCAGCACACCTGGTGCGTG
1055	C3orf35	AGGTGTAAACTTTCCAGAAC
1056	C3orf35	CGTGAGAAGAGACACCTGTG
1057	C4orf19	GCCGATGCCGTTGACACCGC
1058	C4orf19	TGATCAACGTTGTCTCCAGC
1059	C4orf19	AAACAATCCAGATGGGGTGC
1060	C4orf19	ACCCATGGCTCCGATGGAGA
1061	C4orf19	CCCCAATGCTGAAGGTAATC
1062	C4orf19	GGGAACAGTATTTGCAGTAC
1063	C6orf141	CACCCAAATACGTGCTTGTG
1064	C6orf141	CACCCGCACAAGCACGTATT
1065	C6orf141	CAGGCGTGTAGCCCCGCCGC
1066	C6orf141	GATCTGCCGTTCTGTCCTCG
1067	C6orf141	ACGAGGACAGAACGGCAGAT
1068	C6orf141	GGGATGCCCCTGTTCGTGCC
1069	C7orf43	ATCGGGTCTGCAGCAATCGC
1070	C7orf43	GGGCGAGCAGAGCGCTTTCA
1071	C7orf43	GAGGATTCGGATATCCCAGA
1072	C7orf43	TATCTACCTGTCATGCCCGA
1073	C7orf43	GAGATGCCGGCAGTTCACTG
1074	C7orf43	AAAACACTTGACCGTGCTCA
1075	C9orf85	CGATAAAAGTGTGCAGACCA

1076	C9orf85	AACGAGCCACGTTGCCTTTC
1077	C9orf85	ATTCTTATCACATAATGTGC
1078	C9orf85	TGCGCAAACTTCAAGTTCAC
1079	C9orf85	ATTAATGCAAAACTTCATGA
1080	C9orf85	CATTATCAAAACCTAAAAAG
1081	CA2	TTCACTAACTTCGATCCTCG
1082	CA2	GTATGAGTGTCGATGTCAAC
1083	CA2	CAATGGTCATGCTTTCAACG
1084	CA2	CTGCTCGCTGCTGACGCTGA
1085	CA2	ACAAGGTTCAGAGCATACTG
1086	CA2	CTTAGCTTCACTTGGTTCAC
1087	CABP5	CACGGTTCATGCGGATTTGC
1088	CABP5	CTCAATCAGTTCCATCTCCG
1089	CABP5	TCTATTCAGTTTGACACGAA
1090	CABP5	TGAAGGCATCCCGCATCTCC
1091	CABP5	GACGGCACAGTTGACTTTGA
1092	CABP5	TGATCGGTGTCCAGGAGATG
1093	CABYR	AAGTTACGGTATTGCTGGGG
1094	CABYR	CCATCAGTTTATGCTGTGCC
1095	CABYR	TCCTTCAGGGAATACTACTA
1096	CABYR	GATGTAGCTAAAAAAAGTTC
1097	CABYR	CAGCTTGCTGCTCAGATGTT
1098	CABYR	AAACAATTTCATCAGATTAA
1099	CACNA1S	GAGCAAGATGATCGTCTCGA
1100	CACNA1S	CAGGACGCTTACCTGCGCAG
1101	CACNA1S	CACCATCTTTGCCAATTGTG
1102	CACNA1S	CTGGTGGAATAAGAAGCCGT
1103	CACNA1S	GTCAGGCAGAACAAGGCCCG
1104	CACNA1S	CTTGGCAGAATCTCAGGAAC
1105	CACNB1	ACCGTCGCTCAAGGGCTACG
1106	CACNB1	ACCTCGTAGCCCTTGAGCGA
1107	CACNB1	GGGCGATTCAAACGGTCAGA
1108	CACNB1	TCTCGAGCTGCGCTAATGCC
1109	CACNB1	GGCATTAGCGCAGCTCGAGA
1110	CACNB1	ATCCAACAGCTTTGTCCGCC
1111	CACNB4	GGACCGGGAAGCAATTCGAC
1112	CACNB4	CCCGTCCGCGGTCCCGTTCT
1113	CACNB4	GTCTGACTCCGATGTCTCTT
1114	CACNB4	GAACAGGCACATCCTCGTCC
1115	CACNB4	GCCGCACTCCCCCACCTCGC
1116	CACNB4	AAGACAAATGTGAGCTACTG
1117	CAGE1	GGTGGGACTTCTCACATGCC
1118	CAGE1	ATAAGCATCTTCACAAAGTG
1119	CAGE1	GACACATTCTAATCTGCTCC
1120	CAGE1	ACACTTTGTGAAGATGCTTA
1121	CAGE1	ACATTTCAAAGAGAGTGAGA
1122	CAGE1	AAAGAAAACTTTAGATAAAG
1123	CAMK1	CATTGTACACCGGGATCTCA
1124	CAMK1	GGAGCTCTTTGACCGTATTG

1125	CAMK1	CAAGTGCCGGATGAAATCTT
1126	CAMK1	GCACCCCAACATTGTAGCCC
1127	CAMK1	CACTCTCATAGATGTCATCC
1128	CAMK1	GAAAAGATTCACCTGTGAGC
1129	CAMSAP3	TGGCCCGCGAGAAATCGTAC
1130	CAMSAP3	GGTACCTGCGCCCCCGAACG
1131	CAMSAP3	TCTGCAGATCCGATACCGCA
1132	CAMSAP3	CTGCCCTCCAGACCGTCCGG
1133	CAMSAP3	GGGCACGTCGCGCCACCACA
1134	CAMSAP3	TCGCTGGGCCGCTTCCTGCT
1135	CANT1	ATTCGATACCGAATCCCAGC
1136	CANT1	AGCCCGCAGGGCGTTGTAGT
1137	CANT1	GGGGTCGTCTACCAGATCGA
1138	CANT1	ATCGGAAGCGTGAAATACGA
1139	CANT1	GTATTTCACGCTTCCGATCT
1140	CANT1	GAAGGACGAGCGTCTGTACG
1141	CAPN8	CGGTGGAATTCAAGACGCTC
1142	CAPN8	GGAACAGACATAAAATTCGA
1143	CAPN8	ATTCAACATCAACACTTGCA
1144	CAPN8	CAAAAGTATCTTGAGTGCAT
1145	CAPN8	ACGTGCCTAGAGCAATGGAA
1146	CARS2	TGCGCATGATCATATACAGT
1147	CARS2	GGACGCACGTGCCTACATGA
1148	CARS2	GTATATGATCATGCGCACCT
1149	CARS2	TTAGATTTGATATCATTCGA
1150	CARS2	TTCCCTCGCCAGTCTTTATG
1151	CARS2	TATGCTGCATCCAAAAACCT
1152	CATSPER2	TGATCGTATTCAAAAAGACC
1153	CATSPER2	AAACCAAGCTGCCACCTCCA
1154	CATSPER2	TTTGAATACGATCATATTGA
1155	CATSPER2	TCAAATTCAAATTATTATTT
1156	CATSPER2	AAATAATAATTTGAATTTGA
1157	CATSPER2	AATCCACAAATACCAAACTA
1158	CBS	GATTTCGTTCTTCAGCCGCC
1159	CBS	GAGTCGAACCTGGCATTGGT
1160	CBS	TGTGCCCTCAGGGATCGGGC
1161	CBS	ATCTTGTTGATTCTGACCAT
1162	CBS	TTCTCGGAGCTCATCTTCTC
1163	CBS	ATGGTCAGAATCAACAAGAT
1164	CBX3	AACACAGTGCTGACAATACT
1165	CBX3	AGACTTGGTGCTGGCGAAAG
1166	CBX3	AAACAGGCTGACAAACCAAG
1167	CBX3	AGAAACGCTTCAATCAATTC
1168	CBX3	AAACAGGAAAGATTCAGATG
1169	CBX3	AGAGCCTGAAGAATTTGTCG
1170	CCDC154	GTCCAGCCATCCGACATCCA
1171	CCDC154	GAAGGGGCCGATGCCCCTGA
1172	CCDC154	AGCGCTGTGAGCGCGCCACG
1173	CCDC154	GACGAGCTGCTGCCCTCGTC

1174	CCDC154	GGCAGCCCAGGCCCCCGAAA
1175	CCDC154	CAAGGCCCAAGCTACAGCCG
1176	CCDC155	TCGAAGCTTGTGACCCTCAG
1177	CCDC155	CCCGGTGGGTGGCCCCACTG
1178	CCDC155	CACCCACCGGGCCCTCGGGC
1179	CCDC155	TTGATACAGGGGATTAGAGC
1180	CCDC155	TCTCACCAGATGGCAGTTGC
1181	CCDC155	TGTCTCCCCAGTGTACCTCC
1182	CCDC25	CAGTTACCTTATGTAATCGA
1183	CCDC25	TGTATACCTTCGATTACATA
1184	CCDC25	GTTACTTACCAGATATCTTC
1185	CCDC25	ATCTGCCTACACTATTTACA
1186	CCDC25	CGCCCACTCACCGCTGCTGC
1187	CCDC25	GATGAAGATCTGATCAAGCA
1188	CCDC28A	AACAGCCAGTGACTCCAATC
1189	CCDC28A	GAAATTAGCTCGCTTGAATT
1190	CCDC28A	TTAAGTTCCTTATTGCACCA
1191	CCDC28A	CACAGCGACCAAAGTTAAAA
1192	CCDC28A	CACTGATGTCTCAGATGTTC
1193	CCDC28A	GAACATCTGAGACATCAGTG
1194	CCDC78	TCCTTATTGAGCGCTAGATC
1195	CCDC78	ACCAGATCTAGCGCTCAATA
1196	CCDC78	TGTCCCACAGATCCTTCGGC
1197	CCDC78	ACCCCGAGAATGAGCAGCAC
1198	CCDC78	TCATGGTAACCTGGAATCTG
1199	CCDC78	CTGAATGTCGACCAGCTCCT
1200	CCL8	GCTGCTTTAACGTGATCAAT
1201	CCL8	ACAGACCTCCTTGCCCCGTT
1202	CCL8	CACCAACATCCAATGTCCCA
1203	CCL8	CAGGGATTCCATGAAGCATC
1204	CCL8	AAGTGGCTGCCATGAGCAGC
1205	CCL8	CCTCAGGGACTTGCTCAGCC
1206	CCNA2	CAGCGCCCGTCCAACAACCG
1207	CCNA2	CCGGGTCCGCGGTTGTTGGA
1208	CCNA2	CAGTATGAGAGCTATCCTCG
1209	CCNA2	TGAAGTACCAGACTACCATG
1210	CCNA2	CTTCAACTAACCAGTCCACG
1211	CCNA2	ATTGACATGTCCATAGTATG
1212	CCND1	CGAAGGTCTGCGCGTGTTTG
1213	CCND1	CTTGCACACCCACCGACGTG
1214	CCND1	GATGTCCACGTCCCGCACGT
1215	CCND1	GTTCGTGGCCTCTAAGATGA
1216	CCND1	TGTTTGTTCTCCTCCGCCTC
1217	CCND1	GAAGCGTGTGAGGCGGTAGT
1218	CCNDBP1	CCCACAAGTCGGCGAAGCCC
1219	CCNDBP1	CATAATCCCATGCCTTGCGC
1220	CCNDBP1	TGGATGCTCTCACCAGCGCA
1221	CCNDBP1	AGTTTAATCGAGAGATGTTC
1222	CCNDBP1	CGCAACCCAGACACTGTTGT

1223	CCNDBP1	CAGATGCCTCAGATACCAAG
1224	CCNG1	TGATCCGAATAAGTCAATAT
1225	CCNG1	TTGTTTCTGGGCGTACTGCA
1226	CCNG1	ACCAGTTAAGGGACCATTTC
1227	CCNG1	TAAACCTATATTGACTTATT
1228	CCNG1	AAGACTAGAAGCTCAACTGA
1229	CCNG1	AAGTGTGTAGAGTTAACAGA
1230	CCNG2	CGGGTTGTTGAACGTCTACC
1231	CCNG2	CGTTCAACAACCCGAGAAGT
1232	CCNG2	AGCCTTGTGCCTTCTCAATT
1233	CCNG2	TGTCCAAAATATTGACAGCC
1234	CCNG2	GCTGAGCTTCTAGTTTATCA
1235	CCNG2	AATGATAACACTTTGTGTCC
1236	CCNL2	ATGACGTCCCGTATGCGTCT
1237	CCNL2	ACTTGGTATAAAAGAACCGC
1238	CCNL2	ACCTCGAGTAGCTCTGCTCA
1239	CCNL2	TCCGCTTCAGCATGTGTCAA
1240	CCNL2	TCCTCTTAGGAGACGCTGAT
1241	CCNL2	CTCCATGGAGTGCTTCACGA
1242	CCNO	GCATTTCACGCACGCTCGCG
1243	CCNO	ATCCCGGTGCACCGCCAATT
1244	CCNO	ACTCGAAGGAGAGGCCGAAT
1245	CCNO	CTACAGACCTTCCGCGACTA
1246	CCNO	CTCGAACAGGTCGCAAATGC
1247	CCNO	AGCTAATGGTGGGCGCACCC
1248	CD300LG	GGGGCCAGTATGCGGACCAT
1249	CD300LG	AGTACTCCCCAGCGTCTTGC
1250	CD300LG	TCGAACCCGCTGATTTCCTC
1251	CD300LG	CTGTCCTTACCAAGCGTGAG
1252	CD300LG	GAAGTTCTGCCTCTCACGCT
1253	CD300LG	TGCAGGCGTGTTGTAGCCAG
1254	CD36	CTCACTCACCTGTACGTATA
1255	CD36	ACCTTTATATGTGTCGATTA
1256	CD36	TAGCAAGTTGTCCTCGAAGA
1257	CD36	GCCATAATCGACACATATAA
1258	CD36	GATGTTTCAGAACCTATTGA
1259	CD36	TCAATAGGTTCTGAAACATC
1260	CD46	CAATTGTGTCGCTGCCATCG
1261	CD46	CGATTTCCAGTAGTCGAAAA
1262	CD46	TTTAAAGGATCCCGTATATA
1263	CD46	AACTCGTAAGTCCCATTTGC
1264	CD46	TCGTTCACCAATCTCATAGT
1265	CD46	TAACAGGCGTCATCTGAGAC
1266	CD72	CCCCTCGACAGCGGTCAAGT
1267	CD72	GGGCACTTGAACATTCTCGT
1268	CD72	ACGGCTCTCCAGGACGCAGT
1269	CD72	GGTATCGCAGGCAGGTTGTG
1270	CD72	TGATGCATTATCCATCCCGA
1271	CD72	GTTCAAGTGCCCGCAGTCCT

1272	CDA	TGAACGGACCGCTATCCAGA
1273	CDA	GTGTACATGACCAAGCCGGA
1274	CDA	GGTCATGTACACGGGCCAGT
1275	CDA	AGAAAATGCCTGCTACCCGC
1276	CDA	AAAGTGACTGTAGGGGCAGT
1277	CDA	CCCCTACAGTCACTTTCCTG
1278	CDADC1	CAGTGGCAGCTGAATCCATC
1279	CDADC1	CTCTCGTAGAGAAGTTGTGA
1280	CDADC1	TCAGCATACTCAGATCCAAC
1281	CDADC1	AGTATCTACTGACAAAAGAC
1282	CDADC1	CCTCTCTTCATTATCTCCTA
1283	CDADC1	AGGTTGACTTTAGAAAGCCT
1284	CDC42	ATTTGAAAACGTGAAAGAAA
1285	CDC42	ACAGTCGGTACATATTCCGA
1286	CDC42	GACAATTAAGTGTGTTGTTG
1287	CDC42	TCAAAAAGTCCAAGAGTATA
1288	CDC42	TATGCAGTCACAGTTATGAT
1289	CDC42	ACTAGAAATACATCTGTTTG
1290	CDC42BPA	AACCTTGCACTATGCTTTCC
1291	CDC42BPA	CAACATAATAATCCATAACC
1292	CDC42BPA	TCATTCACAGTACCTGGTTA
1293	CDC42BPA	TTACTAAGTTATTGTCATCC
1294	CDC42BPA	TGTTTCACTTTAGAAGTAAA
1295	CDC42BPA	AGAAGACTTTGAAATATTAA
1296	CDC73	AGTTTCTTTACCTCAATTCG
1297	CDC73	CCTGATCGAAAAGATCTACT
1298	CDC73	TCGCTGAAGACCTATTTCTA
1299	CDC73	AGAAGCGCTCCCTTAGAAAT
1300	CDC73	AGCAAAGAAACCACGAATTG
1301	CDC73	TTAGACTGAAAATATTCCTG
1302	CDH13	CAAAGTCCCAGTGCCCGTAG
1303	CDH13	TACACGCTGTTGTCGACAAA
1304	CDH13	GCTTACAGTTTAGAATTGAC
1305	CDH13	ACTCACCACTGTCAATTGCC
1306	CDH13	AAGCTGTTCCTGATAAAGTC
1307	CDH13	TCAGCCAGCTGAATTCATTG
1308	CEACAM16	GCACGGTCAGTGTTACGTCC
1309	CEACAM16	CACGTGTCCGTAGCCCACCT
1310	CEACAM16	AACGTCACGCTGGTCGTCCA
1311	CEACAM16	AGAGAGCTTGACCACGACTG
1312	CEACAM16	GGCCGGCGCCTATCAGTGTG
1313	CEACAM16	TCCGCTGGTTCTTCAACGGT
1314	CEBPB	ACCCGCGTTCATGCAACGCC
1315	CEBPB	CGAGTACGGCTACGTGAGCC
1316	CEBPB	TACGCGCTGCGCGCTTACCT
1317	CEBPB	CGATGTTGTTGCGCTCGCGC
1318	CEBPB	TGAAGTCGATGGCGCGCTCG
1319	CEBPB	AGGTAAGCGCGCAGCGCGTA
1320	CECR2	ATCTCCACCCGTGTGCGAAG

1321	CECR2	TACCGCTGGGAGCTCGAAGA
1322	CECR2	CCATTGGGTGAAGACAATTC
1323	CECR2	GGATGCAGACAGTCTCCGTG
1324	CECR2	TCCTGCAGTTTCTTCCGTTT
1325	CECR2	TGCAGGAGGAGATTCTGTTG
1326	CENPF	CTGCAGACTGCGCAGCTTGT
1327	CENPF	GTTTCAGCTTGACAGTCTCG
1328	CENPF	CTGAATTCAGTTGTCCTTCC
1329	CENPF	GGTTGAAAATGAAAAAACCG
1330	CENPF	ACACCAAGTCAATATTATAG
1331	CENPF	ACAGCTTGACAAACTGAAGA
1332	CENPP	GCGCACGTTTAAACATCTCA
1333	CENPP	CTACAGAGACACAGATTATC
1334	CENPP	CAAGTTGAAATGTAACCATG
1335	CENPP	ACTTAATTCTGAGCATTCTG
1336	CENPP	ACCACTCCACAAAAAAATGC
1337	CENPP	AATTAAGTGAATTTGTGTCT
1338	CENPT	CTCTGGCTATCGTCCTTGTT
1339	CENPT	TGGGGGAAGCCGTTTCAAGC
1340	CENPT	ACTGGCTTCACTACCGACTC
1341	CENPT	GAAGTAGAGCCCTTACACGA
1342	CENPT	GGTTGGACCGCCTGCGGTGC
1343	CENPT	CTCTTCAACTCCATCGTGTA
1344	CENPV	CGGGAGCCATGCGGCGATCG
1345	CENPV	AGGTTGGACGCCGAGGACGT
1346	CENPV	TGAGGGCACTGTGCGGAGTA
1347	CENPV	CAAAACGAACTGCTCCACAG
1348	CENPV	AGGAATTCAATGGCAGCGAT
1349	CENPV	AGGAGCTTGAAGCGAGAAGC
1350	CENPW	ACTCGCACAAGCGTTTGTCC
1351	CENPW	GCATGTACTGGCCGCAGCAA
1352	CENPW	ATAAAGCGGAAGGCTCCCCG
1353	CENPW	AAAGAAGCCTCAACTTCGTC
1354	CENPW	GTAATTCTAAAGAAGAGCAG
1355	CENPW	TGTCGACCATAGTCTCCCAG
1356	CEP290	CCTGCCCCGTCAAGAAGAAC
1357	CEP290	AGATGCCACCTAATATAAAC
1358	CEP290	TTAGATGAAAGCTCAAGAAG
1359	CEP290	AATTACTCAGTCACTAATGA
1360	CEP290	GTGACTGAGTAATTCTGAAA
1361	CEP290	TTGGAAGAAGTAGAAAAAGC
1362	CES2	CCTCTTTACTCTTGCCCCGC
1363	CES2	CCATGAAGGCTCTAACCTGC
1364	CES2	CAAAAACAAGCGCACCACCG
1365	CES2	TCTACAGGACCTCACCGCAG
1366	CES2	CAGCATGGAACCATCATACA
1367	CES2	CTGTGACCAAGTTGACTCTG
1368	CETN2	ACACTGATCTTAATAGAGGC
1369	CETN2	GTTCAAAAATCTGAAACGCG

1370	CETN2	ATTGATGAAGCTGATCGAGA
1371	CETN2	CGAAAAGATCAAAAGCTTCC
1372	CETN2	CTTCAGTTCTTTAACATCTA
1373	CETN2	GGGTGAGAACCTGACTGATG
1374	CFDP1	GCCCCCAAGTACACAAGTTA
1375	CFDP1	GACAAGTTCAAGTAAATTGT
1376	CFDP1	AATACTCACCCTGACCCGGC
1377	CFDP1	GCTGAAGGAACATTAGCCTG
1378	CFDP1	GGATGCCAATTCAGAATCTG
1379	CFDP1	TGATGTAAATGAATTAGTGA
1380	CFHR1	CAGAAGCTTTATTTGAGAAC
1381	CFHR1	AGAGAGTACGTTATGAATGT
1382	CFHR1	CTTACCAGTGGACCTGCATT
1383	CFHR1	ACAGAACCACCTCAATGCAA
1384	CFHR1	GGTCCACCCCTCCCAAATGC
1385	CFHR1	CAGCTGAATTTGTGTGTAAA
1386	CFHR4	TGGTACTCGACTCTTGACCA
1387	CFHR4	CGCAAAAAGTGTATCTGCCA
1388	CFHR4	TTACTGAGGCATGGGACCGT
1389	CFHR4	TAAGATTGTCCTGCAGCTGC
1390	CFHR4	AAACCACATGCCATTACTCT
1391	CFHR4	AAATACTTACTATAGCATGT
1392	CGB5	ACCAAGGATGGAGATGTTCC
1393	CGB5	CACCATGGTGGGGCAGTAGC
1394	CGB5	GCTGTTGCTGCTGCTGAGCA
1395	CGB5	TGAGAAGCCTTTATTGTGGG
1396	CGB5	CTTCCAGACCCGCGTGCTGC
1397	CGB5	GGTGTTGACGGTGATGCACA
1398	CGN	AGACCTCCTTCGAGACCAGC
1399	CGN	CATATGAAGGCCACCATCTA
1400	CGN	TTCAGTAACACCTCCTTAGC
1401	CGN	CCAGAACATGAAGCGCCTCT
1402	CGN	ACTAACCTTCCTCCTCACAG
1403	CGN	GGAGAAGATGCAGCCTCTAG
1404	CHAF1A	CTCGGGCCACTCGTCAGCTC
1405	CHAF1A	CGATGACATGTCAGACGATC
1406	CHAF1A	CCGACCTGTGGCGGCTCCCC
1407	CHAF1A	TGGTCTATCTTTGCAATCCA
1408	CHAF1A	AGTGCGGGGCGCCCGGCGCC
1409	CHAF1A	CCAGTTAAGAAGTTAATACA
1410	CHAF1B	TCCTGAAAAGCGATCTGCTC
1411	CHAF1B	TTTGGTATGACGAGCAAGAT
1412	CHAF1B	CAGATGCTGTCATCCTATTG
1413	CHAF1B	ATCATATGGGATGTCAGCAA
1414	CHAF1B	AGAAAAACGCACAACATTGA
1415	CHAF1B	TTTGCTGACATCCCATATGA
1416	CHD1	ACCCAGAATCATCATCCGAC
1417	CHD1	TTCTGATCCGCTATTAGATG
1418	CHD1	CATCAAGCCTCATCTAATAG

1419	CHD1	AGCCAGTCGGATGATGATTC
1420	CHD1	TTCCGATGACTCATCAAGTG
1421	CHD1	GACCTCACTTGATGAGTCAT
1422	CHD1L	GTCGCCTGCATATGTTACAC
1423	CHD1L	TACCGGCCCGATGGAGCGCG
1424	CHD1L	AGCAGGACTTACGGCAGTGG
1425	CHD1L	GAAAACGTGACTCCTGTTTC
1426	CHD1L	CTTACTGCGGCTTCATACTG
1427	CHD1L	AGCCGCGGGGGCCAAGCGCC
1428	CHD3	TCTTACCGCACCTCATGAAC
1429	CHD3	CTTTGTAGTTGGTGAGCGTG
1430	CHD3	GGCACTGAACTCTCTCCATT
1431	CHD3	TCTAAGATGATGACCATCCT
1432	CHD3	TTCTTCACACCCAATGCTGA
1433	CHD3	TCAAAGATAAGGATGACATT
1434	CHD4	TCGAACCCTCACCAACTACA
1435	CHD4	TTGCGGATAGGCACCTCCAC
1436	CHD4	CTCCTCACTGCCCGCCGAGC
1437	CHD4	AATCCTCCTCTGAGAACACG
1438	CHD4	GAAGGGGATGGCGTCGGGCC
1439	CHD4	AGGTGGTGGTGCAACCTCAG
1440	CHD5	CGACGTGGACTACCTGTTCT
1441	CHD5	ACAGGTAGTCCACGTCGTCC
1442	CHD5	CACAGGGAAAAAGTCATCGA
1443	CHD5	CTTCTTCTTAGGAAGGCTCA
1444	CHD5	TGAGCTATCAGAGAATGAAG
1445	CHD5	GAAGAGAAGTCGGAGAGTGA
1446	CHD7	CTCCAATGAAGGATCTACCC
1447	CHD7	AAAGCGCTACACTGAAGACC
1448	CHD7	TTCTCTAGAAAGGGCTCCGA
1449	CHD7	TTAACTATATCAGTCCAGTC
1450	CHD7	GAAGAGACGGTCCAGCAGAC
1451	CHD7	TGTCTCTCAGCATTCCCCGT
1452	CHD8	TGAATCGAAACGCATCACCC
1453	CHD8	GGACATCGGCATGTTGTGCT
1454	CHD8	TAGCACCATCACTCCTGTAG
1455	CHD8	CACATCTGAATTATCAGATG
1456	CHD8	CAGCTGGATAGTTACTACCT
1457	CHD8	ACCAGGCAAGATAGTGTTAC
1458	CHDH	CACCCTGCTCACAAGCGTCT
1459	CHDH	AGGCCGAGACGCTTGTGAGC
1460	CHDH	GACCAATGCAGACCTTCCGC
1461	CHDH	CTTCCAGGCTTATGCCAGCA
1462	CHDH	CCAGGTAGGTGGCACACCAC
1463	CHDH	CCAGAACCTGCAAGACCACC
1464	CHEK1	CTGGGACTTGGTGCAAACCC
1465	CHEK1	CCCTCTTAGGTTTATCTGCA
1466	CHEK1	AGTCATGGCAGTGCCCTTTG
1467	CHEK1	GAGATTCTTCCATCAACTCA

1468	CHEK1	AATCTCTGAGCATCTGGTTC
1469	CHEK1	TAAACCAGAAAATCTTCTGT
1470	CHEK2	AAGAAGCCTTAAGACACCCG
1471	CHEK2	AATGTGTGAATGACAACTAC
1472	CHEK2	ATCATACTTAGACTGCAAAC
1473	CHEK2	TGAATCCACAGCTCTACCCC
1474	CHEK2	AGTGTTTCTTGCTGTATGTT
1475	CHEK2	CAGGAGGATCAGATGACAGC
1476	CHI3L2	GGATATGGGAGCAACCACCA
1477	CHI3L2	CACCTGCCCAGAGAGACTTC
1478	CHI3L2	AGTGGTCTTGCTGCTTCTCC
1479	CHMP3	TGTTTAGGCTGGGATCATAG
1480	CHMP3	AATCTTCACAAGACTTTGCA
1481	CHMP3	CCAAGGAGATGATCAGGTCA
1482	CHMP3	CTCATGGTGGCCTGAATCTC
1483	CHMP3	AGGAAATGAGAGTTGTTGAC
1484	CHMP3	ATTCTCTTTGAAATTACAGC
1485	CHMP5	TTATACCATCCAGTCTTTGA
1486	CHMP5	TGCCCAACAGTCATTCAACA
1487	CHMP5	AAATCATCTTCATCCAGTTC
1488	CHMP5	GATGAAGATGATTTAGAAGC
1489	CHMP5	TCGAGAAATCTTCTTGTCAA
1490	CHMP5	TGACAAGAAGATTTCTCGAT
1491	CHRAC1	CGCTGCCTCTATCCCGCATC
1492	CHRAC1	CATCATGAAGAGCTCCCCCG
1493	CHRAC1	CTTTCCACTGCCGTGTCTGT
1494	CHRAC1	GCCACCTATTCCTACAGACA
1495	CHRAC1	TTTAGGAGTCAGCTTCATCA
1496	CHRAC1	ATACCTGAAAATGCTTAAAG
1497	CHRM2	GGCTGCAATAGCCGTACCAA
1498	CHRM2	CGCTTGACTGGGTAGGTCAG
1499	CHRM2	TACCCCAACTAATACCACCG
1500	CHRM2	GACTAGGATGTTCCCGATAA
1501	CHRM2	GAGGTGGCGGTTGACTTTAA
1502	CHRM2	GTGTGACCTTTGGCTAGCCC
1503	CHRNA5	GTAGTTTGCCGGTGGAGTCC
1504	CHRNA5	TATTTCAAGACTACGAAAGA
1505	CHRNA5	CCAGTACGAAAACAGTCATC
1506	CHRNA5	AGTTAATGACAACAAACGTC
1507	CHRNA5	ACTTGCAATATCTCAATTGG
1508	CHRNA5	GTGTTCCTTCAGACTCTGTC
1509	CILP	TGGTCCGAGCCTCTAGCCGC
1510	CILP	GACTCGCACACGCATTTGCT
1511	CILP	TAGAGGCTCGGACCACTGAC
1512	CILP	AGCAAATGCGTGTGCGAGTC
1513	CILP	TGCCAAGCCTGCCGACACCC
1514	CILP	GCTGGACTCTTCTTACTGAC
1515	CILP2	CACGCGGTTGCTCGCGGTTG
1516	CILP2	TGGAAGCGCGCACCACGGAC

1517	CILP2	CTGTGTCGTCGCTGCGCACC
1518	CILP2	AGGCCCAGTGCAGTCGCCAT
1519	CILP2	TGAGGGCTGGCCGGTGTACA
1520	CILP2	CCTCACCTCGGGCCCCCGCC
1521	CKM	CCCGTGGCGATCCGAGATGA
1522	CKM	TCACCCCTTCATCATGACCG
1523	CKM	GTAGACGATGTCATCCAGAC
1524	CKM	TCTGCAGCCCTACGCAGAAG
1525	CKM	GCAGCTTCTTGTAGAGTTCA
1526	CKM	CAATGACAACAAGAGCTTCC
1527	CLDN10	GGCGTGCGTTACCGACTCCA
1528	CLDN10	ACATGTCCAGGGCGCAGATC
1529	CLDN10	GAACTGCGCAGGTAACGCGT
1530	CLDN10	TGTGATGACCGTGCCGTCGA
1531	CLDN10	TTTATCGGAGCCTCCGACTT
1532	CLDN10	GGTTCCATATTTGCGCTCTT
1533	CLDN11	GGGCTTCGTCACGAGCTTCG
1534	CLDN11	TGGGCACTCTTCGCATGAGT
1535	CLDN11	GGACCGAGGCAGCAATCATC
1536	CLDN11	CGCCCTGATGATTGCTGCCT
1537	CLDN11	GCGCCCTTGTTGCCACCATC
1538	CLDN11	GGTGGGGATGGTGTAGCCGC
1539	CLDND2	ACTGCGGATTCGGTGCGACG
1540	CLDND2	CTGCTGCTGACCGCCTTGAT
1541	CLDND2	CTACTGGACCCGCCAACAAG
1542	CLDND2	TTTACCCGCGAGAATTGAGA
1543	CLDND2	CGTGGTTGCATTCCTGCCAC
1544	CLDND2	CAGTCTCACCGCCGAGGAAG
1545	CLLU1OS	ACTTACCAGTGGCAGTCTTA
1546	CLLU1OS	GAATGCCTTAAGACTGCCAC
1547	CLLU1OS	TCCCAGACTTGTACTTCCTA
1548	CLLU1OS	CGCTCTCACATATGCAGAGA
1549	CLLU1OS	CCTTCTGAAATCTCATAGTT
1550	CLLU1OS	GTACAAGTCTGGGAGATACT
1551	CLP1	CTGTCCGAGACAAGTGTAGC
1552	CLP1	ATATCTCGCCCAGGAGTGAC
1553	CLP1	TCGCTCTAGTTCAAATTTAG
1554	CLP1	GATGCGGAGGCAAGCGGAAA
1555	CLP1	GCAGCACAGTGCGTACAAAG
1556	CLP1	GGGTGTGGTGGAGCGCTCCA
1557	CLUAP1	CTGCCTTACTGCGGAGGTCG
1558	CLUAP1	AATTTCCGTACACCCAATTT
1559	CLUAP1	GATTTCAGACGCAAGCTGCC
1560	CLUAP1	AACAAGTTCAAGTTTGATCT
1561	CLUAP1	GGAGGGCTCTGAAATAGTAG
1562	CLUAP1	ATACCCTCGACATATTTCTA
1563	CMPK2	CGTGGCATCCAGTCCTTCGA
1564	CMPK2	ACCTACCTGTCTACAATCAC
1565	CMPK2	CACAAACTTCACTGCCGAGC

1566	CMPK2	GGTATAAGGTTCCTCCCAGC
1567	CMPK2	CATCAAAGATCTTCCTCCAC
1568	CMPK2	CCACTGGTACACAGGGTGAT
1569	CMTM4	GCGGGCTCGGATGTAGTCAT
1570	CMTM4	CTTTATTGCTTCAATCGTAC
1571	CMTM4	TGACTACATCCGAGCCCGCA
1572	CMTM4	CTGTCAGATTCCAGTTGATC
1573	CMTM4	ACTCCACAAACGTAACAGTA
1574	CMTM4	CTTTAGGATTTGGTCAACAC
1575	CMTM7	CTCCGGCTACCAGTCCGCTC
1576	CMTM7	CGCGCAATGTCGCACGGAGC
1577	CMTM7	CCCCTCCAAGGGGCTCGCGC
1578	CMTM7	GCACGCGGTAGAAGCGGAAG
1579	CMTM7	GATCATTATCAAGTCGCAAA
1580	CMTM7	TCGTGTGTAACCCAGTCCAC
1581	CMTM8	GGTAAATCACTGTATGGTCC
1582	CMTM8	AGGCACTGCCGTTAAAGCAC
1583	CMTM8	GCAGCAGCTTCGCCTACGAC
1584	CMTM8	ACATAACAATGACCTACACC
1585	CMTM8	TCACGATCTCGGCCACGATG
1586	CMTM8	TGTATTTCCAGCGTAGCAGA
1587	CNDP2	GCCTGCTCGCCGACGACTTC
1588	CNDP2	CAGCATGACGTTCTTGCCCG
1589	CNDP2	CCGTCATCCGCTGACCCCAC
1590	CNDP2	CATGTTCGGCACGAGCCTGA
1591	CNDP2	CGGAGAAGAGAGGCGAAATC
1592	CNDP2	AGCGGGATCTCCGAGCCATC
1593	CNIH2	GTATCACTTACTGCCCGCGC
1594	CNIH2	CTTCAAGAACCCCATCGACC
1595	CNIH2	GTCCCAGAATACTCCATCCA
1596	CNIH2	CCTTCTTCTATTACCTGTAC
1597	CNIH2	CCTGTACAGGTAATAGAAGA
1598	CNIH2	TGACACTGACCTTCCTCAGG
1599	CNNM3	CGACACTCCATAGTACGTGA
1600	CNNM3	CTTCACGTACTATGGAGTGT
1601	CNNM3	GGTAGTCTTCAGACTCGTCC
1602	CNNM3	TTCCATCTTAGGAGACACCG
1603	CNNM3	TGAGGTTTGACGAGAGCAAC
1604	CNNM3	GGCTGCGCTGGTACAGGTAG
1605	CNOT1	GAATCTTGACTCGCTCTCGC
1606	CNOT1	TCTTGGTTAAATTGTCCACC
1607	CNOT1	GACAGCGTCGAGATAAAATT
1608	CNOT1	CCAACCTTCTGATAGTGCAA
1609	CNOT1	AGTGGCGATGGTAAAAGCAG
1610	CNOT1	AATAAATGCCTGTCTGCCTC
1611	CNPY3	AGTCTCGTTCCACAGCTCAT
1612	CNPY3	GCCCGTGCCAATCACCTCCT
1613	CNPY3	CGGCAGCAATCGATTTGCCA
1614	CNPY3	CTTTGAGACATTACACAACC

1615	CNPY3	CTGCGCCAACCACGTGCTGA
1616	CNPY3	CTTGGCAAATCGATTGCTGC
1617	CNTD1	GAGGCCGATCGTGGCCTCAT
1618	CNTD1	TGCAGCCGCACCCACGATCT
1619	CNTD1	GCTCCTTACTTTGTTTCGGA
1620	CNTD1	CTACAGGTTTATGGTAAAAC
1621	CNTD1	CCAGACACCATTGTTCAGAC
1622	CNTD1	TTTCTAGGATTTCTACAGCC
1623	COL14A1	ACCAGTACCGCATCTTGCGC
1624	COL14A1	CTTCTTGTGACTCCAACTTC
1625	COL14A1	TACATTATATCTTAACCTTG
1626	COL14A1	TATTTCTGCACCATTGTCCA
1627	COL14A1	CAGTGTTCTGCAGATTCAGC
1628	COL14A1	CTTCTGAATTGGCCTTGAGC
1629	COL15A1	GCGGATCATCCTCTACTACA
1630	COL15A1	TGGCGAAGAGCACGCCACCA
1631	COL15A1	GAGCTCACCGAGGACTCTTC
1632	COL15A1	CGGGGTGCACGGTGAGCTGC
1633	COL15A1	ACTCACCGAGGCTTGAGTGT
1634	COL15A1	CAGGCATCTGGAGAGACCAG
1635	COL20A1	CAAACTCCCTCCGAGCTAGC
1636	COL20A1	CTGTAGCAAGCGGTCTCCTG
1637	COL20A1	CTACCCAGGGGACTCGGAAC
1638	COL20A1	GGGGAGCGGCCTCGGCTACC
1639	COL20A1	TCCGGAGACCCTGCACACCT
1640	COL20A1	CTTGAACTTGCTCTCTTCCC
1641	COL4A4	CAAGGACTTGGTCAATCTGA
1642	COL4A4	CTCTGCACATAGTACTAATG
1643	COL4A4	GAGCAATCTCTTCCTCCACA
1644	COL4A4	TTGCCACTGTGTTCCTGAAA
1645	COL4A4	CTTCAGATTGACCAAGTCCT
1646	COL4A4	GGATACTCACCAGGGACCTG
1647	COL6A3	GATCGCTTTCGACTCCTCCC
1648	COL6A3	CTAGAGCAGAGCCCGTGTAC
1649	COL6A3	GAAGCACTCCATCCTCGATC
1650	COL6A3	TGTACTGAGCCACCGCAATT
1651	COL6A3	TCTTACCTGCTTGCTGCTGC
1652	COL6A3	CATGCCCAGCAGCAGCAAGC
1653	COL7A1	GCGGAAATTGCTGCGGCCAA
1654	COL7A1	TGGCCGCAGCAATTTCCGCG
1655	COL7A1	TACTGCCTAGGATGACGCTG
1656	COL7A1	CATCCGTGAGCTTAGCTACA
1657	COL7A1	CCCCCTTGTAGCTAAGCTCA
1658	COL7A1	CTGGTGGCCGCGCTCTGCGC
1659	COLGALT1	GGTGGCTACGGACCACAACA
1660	COLGALT1	CCATAGGTCCTACCCGGACG
1661	COLGALT1	GCATGTCATGAAGTTGCGCC
1662	COLGALT1	GAAGAGTTTGTACCATTCCG
1663	COLGALT1	TCTTGTTCTCAGCGATGAGC

1664	COLGALT1	CGCAGCCCGGGAATCCAGCA
1665	COMMD10	AGAATCCGAGTGAGCAACCG
1666	COMMD10	CTGAAGCATTTGTCAATACG
1667	COMMD10	GATTTCCACGGTTGCTCACT
1668	COMMD10	AGGGAGCCAGAATTCTCTGC
1669	COMMD10	ATTCCACAAGAACTTTCTCC
1670	COMMD10	ATGATATTGTTTCAAGAACT
1671	COMMD3-BMI1	CTTGAAACATATCGACCCAG
1672	COMMD3-BMI1	GAATTCCCTAGAAATCCTAC
1673	COMMD3-BMI1	AGCTGCAACTTACATACTAG
1674	COMMD3-BMI1	ACAGTACCTACCTTCCCAGT
1675	COMMD3-BMI1	CTGCAACTAAAACTAATCTC
1676	COMMD3-BMI1	GAGCTGACAAGTCAACTCTA
1677	COX15	ACAACTCTTACCTTGTGCGG
1678	COX15	AGGCGGTACTGACTGACCCG
1679	COX15	ATCCTAAGGTTGACAGAGTC
1680	COX15	GAAGGGGAATTCTCCGAGAG
1681	COX15	CTGAGCGGGTGGTGGGCCGA
1682	COX15	AATGCCAATCTACCATCGAG
1683	COX17	CCAAGAAGGCGCGCGATGCG
1684	COX17	CCACGCATCGCGCGCCTTCT
1685	COX17	ACACTGTGGACATCTAATTG
1686	COX17	AAGGAATGCATGAGAGCCCT
1687	COX17	GAAGCCCTGCTGCGCTTGCC
1688	COX17	TTCTTCTCCTGAGACTCAGG
1689	COX7B2	CCATTCTATTCCAATCTGAG
1690	COX7B2	GTGTTTACAGCCACTCAGAT
1691	COX7B2	GAGACTGCTTAGTGCATTTC
1692	COX7B2	AACACAGAAAGCAGTTCCAC
1693	COX7B2	CTGCTTTCTGTGTTGCTACA
1694	COX7B2	TACTGATGTTTCCACTCTTT
1695	CPA4	CGGCAATGTTGTCCATCTCG
1696	CPA4	GCTTCAGATTTACCACGAGA
1697	CPA4	GACATCCACAGGCCGATTGA
1698	CPA4	AGAGTACGCAGTGACAATTG
1699	CPA4	AGCAGTAATAACTTCAACTA
1700	CPA4	CATCATCTTCATTGTCTAAA
1701	CPEB1	ACGGGAAGATTCTTGCGCAG
1702	CPEB1	TCTGCGCAAGAATCTTCCCG
1703	CPEB1	TATAGTTCCGGGGAGGAAGC
1704	CPEB1	TCTTACCAGCGGGAACGCCA
1705	CPEB1	CGGCCCCATGGCGTTCCCGC
1706	CPEB1	AAACACCTTGCAAGAGTAGA
1707	CPNE3	CAAGAAGCTAACTCGACCAC
1708	CPNE3	GTAACTTACAGGTGAGCTTC
1709	CPNE3	AAAACAACTTGAATCCTGTT
1710	CPNE3	TCTCTTAACTCACTGTGTTA
1711	CPNE3	ATGAAAACTGGCAGACCTGC
1712	CPNE3	TGTTATGATTATGACAATGA

1713	CPNE6	AGGTGCACTCCGTAGAGCCG
1714	CPNE6	GTTCCACGTGTTCGATGCCG
1715	CPNE6	TGAGCCCCCAACCATGACGC
1716	CPNE6	GTAGAGCTTGAGCAGCACGC
1717	CPNE6	TGCGGGCAAGTCCACCATCA
1718	CPNE6	CCAGATTGTGTCACAAACCA
1719	CPT1A	TACGTAATTTGTAGCCCACC
1720	CPT1A	GCCAAGATCGACCCCTCGTT
1721	CPT1A	TGCAAAAATCAATCGGACTC
1722	CPT1A	GACATCGCTGCCTCGCCTGC
1723	CPT1A	GCAGGCGAGGCAGCGATGTC
1724	CPT1A	ATAATCCCAGTATCTACAGT
1725	CRABP2	CACAACGTCATCCGCCGTCA
1726	CRABP2	TCACTCTCGGACGTAGACCC
1727	CRABP2	CAACTGGAAAATCATCCGAT
1728	CRABP2	ACCAGAGAACTGACCAACGA
1729	CRABP2	CAGGATCAGTTCCCCATCGT
1730	CRABP2	CGAGGAATTGCTCAAAGTGC
1731	CREBBP	AGCGGCTCTAGTATCAACCC
1732	CREBBP	GAATCACATGACGCATTGTC
1733	CREBBP	CCCGCAAATGACTGGTCACG
1734	CREBBP	TCGACAATGCGGGAGCGAGC
1735	CREBBP	CAGCGAGCCTATGCTGCTCT
1736	CREBBP	ATTTGGGTTACTTAAAGAAG
1737	CRK	TGGTGCTCGAGTCCCGCACC
1738	CRK	TCCAATCAGAGCCGATACTG
1739	CRK	CGAGTCGAAGTTGCCCGCCA
1740	CRK	CGAGCCCTCTTTGACTTTAA
1741	CRK	AGGAGCGGAGTAGCTGGTAC
1742	CRK	CAAACTCTTGATCTCCTATT
1743	CRKL	CCCGATCTTAAAACGGCGGT
1744	CRKL	TATGGGGCCGGTGTCTCGCC
1745	CRKL	AGGTCTTCGGCATCATTCCC
1746	CRKL	GGTTGAGCGTATGCATGAGC
1747	CRKL	CGTTCACTTCGCCTTCCCAC
1748	CRKL	GTGACATCGTGAAAGTCACA
1749	CRX	GCTCGGAGACCCATAGGCGG
1750	CRX	GGGGTCTAGGCCGCTGAAAT
1751	CRX	CCAAGGCGTTGACAGAATAG
1752	CRX	CCACTATTCTGTCAACGCCT
1753	CRX	CCCAGACGTCTATGCCCGTG
1754	CRX	AGATCAATCTGCCTGAGTCC
1755	CRYZ	CTCTGTGATTGAACACTTCA
1756	CRYZ	TGCAGTGCCTGTGTGAAAGC
1757	CRYZ	AGCCATAAGCTCTAGCAATT
1758	CRYZ	ACTCTCTCCAGCTTTCACAC
1759	CRYZ	AGATAATGCATCTGCTTTCA
1760	CRYZ	TGCCAAATTGCTAGAGCTTA
1761	CSMD3	ACACCGTCAATAAAAAGACG

1762	CSMD3	TTAGCGCATCTTCGCTTGCC
1763	CSMD3	AACCTTGTCCTAAAGTTTGT
1764	CSMD3	ATCCTCATCCTACAAACTTT
1765	CSMD3	ACGTAGTGAGAACACAGATT
1766	CSMD3	AGCACTAACTGCAAAATCAC
1767	CSTF3	GTCCGAAGAGTTTATCAACG
1768	CSTF3	GTAACTTGGTAGTTTACCCT
1769	CSTF3	TGATCAACATTGAACAGCTC
1770	CSTF3	TCAAGCATATGACTTTGCAC
1771	CSTF3	TTCATATGTCCGAGAAACCA
1772	CSTF3	AATGGAAATTATGTCCTATC
1773	CT62	TGCAGCACAGCGTCTGCACG
1774	CT62	GGGTGGTGACAGTCTTCTAT
1775	CT62	GAAGACTCCCTGACACTTCA
1776	CT62	TCAAGTAGAGAGTCAGCCCC
1777	CT62	AAGGATTCCCATGAAGTGTC
1778	CT62	CTTGACCTGTAACCCACAAC
1779	CTCF	CGATCCAAATTTGAACGCCG
1780	CTCF	TACACTGGCGTAATCGCACA
1781	CTCF	ATGCCGAACCAATTCTCCAC
1782	CTCF	CGTCACATTCGCTCTCATAC
1783	CTCF	CATAGCCCGAAAAAGTGATT
1784	CTCF	TTTGCAGTTACACGTGTCCA
1785	CTDSPL	GGAACACACCCCAGCGGTCT
1786	CTDSPL	CCCCAGTGTGCTTCCGCCAC
1787	CTDSPL	GTGAAGGACCTGAGTCGCCT
1788	CTDSPL	TCTTTAAGCTGACGTTGCAC
1789	CTDSPL	ATAGTTCCATCGATTTCAAC
1790	CTDSPL	AATCGATGGAACTATACATC
1791	CTNNB1	GAAACAGCTCGTTGTACCGC
1792	CTNNB1	AGAACGCATGATAGCGTGTC
1793	CTNNB1	AATGCAGTTCGCCTTCACTA
1794	CTNNB1	AAAATGGCAGTGCGTTTAGC
1795	CTNNB1	AGTGAAGGCGAACTGCATTC
1796	CTNNB1	CTCCTTCTTGATGTAATAAA
1797	CTR9	GGCAGCGTATTATGTACAAC
1798	CTR9	ACTTGACTTCGATCAGTTAC
1799	CTR9	GGCCACCTTGTTGTATACAA
1800	CTR9	AGCCTGCTTCTGCCTACTTG
1801	CTR9	AATAATATTCCAGCCCTTCT
1802	CTR9	CTGATAACTTCATCTCCCTC
1803	CTRB2	GCCATCCACCCTGTGCTCAG
1804	CTRB2	CACCTGCAGGGACACCTGCC
1805	CTRB2	AAAACCGGCTTCCACTTCTG
1806	CTRB2	GTCCTCACCTGACCCCGCAG
1807	CTRB2	TCTGCCCAGGACCTCCGACG
1808	CTRB2	TGGGTACCTTGGCGATCTTC
1809	CTSE	GGAACCCCTCATCAACTACT
1810	CTSE	TGGGCTAGTGCAGTACACAG

1811	CTSE	GTTCACCGAGTCCTGCTCAA
1812	CTSE	CAGTCACCAAGCAAGCTTAC
1813	CTSE	TCTCCATGTAGTAACCCAGA
1814	CTSE	TTCACTGTCATCTTCGACAC
1815	CTSG	GGTCGTAGGAACCGAAGATG
1816	CTSG	GATCTGAAGATACGCCATGT
1817	CTSG	TCGGTTCCTACGACCCCCGA
1818	CTSG	GTCTCCTATGGAAAGTCGTC
1819	CTSG	GCACAAAGTCTTCTCGCACC
1820	CTSG	TACCTACAGGGGGATTCCGG
1821	CTU2	CCCGTTGTGGTGATACGAGC
1822	CTU2	TCTTACCTGGGCCGCGGCGC
1823	CTU2	CTACGGGGAGCCGGCGCCTG
1824	CTU2	GGGGCCTTCGTCCAGCTCCA
1825	CTU2	CTCAAGAACCTGCCAGACCA
1826	CTU2	TCACCTGCAGAAGGCATCTC
1827	CUBN	TTTGTATCTGTCCCCCACAG
1828	CUBN	GACCTACCTGCTGCAAGCCT
1829	CUBN	AGATGACTCACCTTCCACTG
1830	CUBN	AATCTATCAGCTTAATTCCA
1831	CUBN	GAAGATATTATAGAGTTAAA
1832	CUBN	GATCTTGAGAGAAAATTCCA
1833	CUL2	TTTGACGACAATAAAAGCCG
1834	CUL2	AATACGTCGAAAGAGCAACA
1835	CUL2	CACCACCATAGCCATACTGA
1836	CUL2	TCATGTTCGGCATTTGCATA
1837	CUL2	AGATATCTATGCTTTATGTG
1838	CUL2	AGATATGAATGAACCACTTA
1839	CUL3	ATGTCAGTTCACGTCAAAAC
1840	CUL3	GAGCATCTCAAACACAACGA
1841	CUL3	TTATTTAGTCGTGTGCCAAA
1842	CUL3	CTTACCTGGATATAGTCAAC
1843	CUL3	GACCTAAAATCATTAACATC
1844	CUL3	TCTGCAGACATTTCCAAAAA
1845	CWF19L2	GTGCGAAGAGTATCGAAGAG
1846	CWF19L2	CTTTACGATGGCAACAAGTA
1847	CWF19L2	GTAAAGAACTTAAGCGACTT
1848	CWF19L2	GAGAATTGAACAGTTCTCAC
1849	CWF19L2	AGGTACCTGGCGCAACACCT
1850	CWF19L2	AAGAACAGACCCGGAATGCC
1851	CXXC4	CGCGCATCGGCATGTCCCCC
1852	CXXC4	GGCCGCGTTGTCGCAGTTCC
1853	CXXC4	CCAGCGCTGAAGCATTCCGA
1854	CXXC4	AATGCTTCAGCGCTGGGAAC
1855	CXXC4	GGACATGCCGATGCGCGCGG
1856	CXXC4	GGTGGTGATGCGCGCGATCT
1857	CYP21A2	ATCATCTGTTACCTCACCTT
1858	CYP21A2	GAGGTAACAGATGATGCTGC
1859	CYP21A2	CCCTTACAGGTAAGTGGCTC

1860	CYP21A2	TGGTGGTGCTGAACTCCAAG
1861	CYP21A2	CAGGTCCTCACCCTGAGAAA
1862	CYP21A2	TGCCTATTACAAATGTATCC
1863	CYP4V2	CATACCTTGTAAGAAGTCCT
1864	CYP4V2	ACATGCCGCTGCTGAAGCTC
1865	CYP4V2	CCGACCCAGAGCTTCAGCAG
1866	CYP4V2	CTCACCACAGATGATATCTA
1867	CYP4V2	TGTGCCTTAGATATCATCTG
1868	CYP4V2	TTGGCCTAGGACTTCTTACA
1869	CYP7B1	TTCTGCCGTGTCCCAACTTG
1870	CYP7B1	CCAACCGCTCCAGCGAAAAG
1871	CYP7B1	ACTTACGAAAAGACCCCTTA
1872	CYP7B1	GGAGAAATATTATGTGCACG
1873	CYP7B1	GAGGCGCTTACCTGGTGCGC
1874	CYP7B1	GGTGAGCCTCCATTGATAAA
1875	CYSLTR1	TCTGGGTACATAAGTCACGC
1876	CYSLTR1	CCTTAGAATGCAGAAGTCCG
1877	CYSLTR1	CCACGGACTTCTGCATTCTA
1878	CYSLTR1	ATGGTACGTTGAATATGATA
1879	CYSLTR1	CATACAAAGCATAGGTGCTG
1880	CYSLTR1	GGAAGTCATCAATAGTGTCA
1881	CYYR1	CGACCCGCGTGGGCATCCTC
1882	CYYR1	ATTCCCAATATAAGCGTAGT
1883	CYYR1	CGGCTTGGATGGACGCTCCG
1884	CYYR1	TGCTCCTACTACGCTTATAT
1885	CYYR1	AACTTCGGAAGCAAGACCCC
1886	CYYR1	GATTATTTCCTTGCGTTTCC
1887	DAD1	GCGTGCAGTTATGTCGGCGT
1888	DAD1	GTAACCGAACTGCAGCGCCC
1889	DAD1	GTGGGTTGATCTGTATTCTC
1890	DAD1	CTCGCTCTGGGGAGATGCCT
1891	DAD1	GTTCGGTTACTGTCTCCTCG
1892	DAD1	TGTGGGGAGTTTCATCCTAG
1893	DAND5	GTATGCCTGCTCGCAAGCGT
1894	DAND5	GGTGCCCAACGCTTGCGAGC
1895	DAND5	CACCCACTCACCTGAACGAA
1896	DAND5	AGTGATCCAGGGGATGTGTA
1897	DAND5	TGGATATCTTCACCCGTCGA
1898	DAND5	CAGCTCAGCCTCCCGTCGAC
1899	DAXX	GAGCAGCGCATCCCCTACCG
1900	DAXX	CGAAGCACATCCCCATAGTC
1901	DAXX	GACCTCATCCAGCCGACTCA
1902	DAXX	CCCGGCGCCTTCGGGAAAAC
1903	DAXX	GGTTCTTCTGACAGTAACGA
1904	DAXX	AGGCGTCTCTCCTCACAACT
1905	DAZAP2	TCAAGATCACGACCGAGAGC
1906	DAZAP2	CGTCCGAGCTTTGTGCACCC
1907	DAZAP2	AGACAACGGTGATAACAACT
1908	DAZAP2	TCCAGGAAATGCGGCTGACA

1909	DAZAP2	GGTGGATAGATGGGACCGAC
1910	DAZAP2	TCGGTATAGGGTGGAGCCTG
1911	DBF4	TTATAGGTGTGTTTAAGCAG
1912	DBF4	TTTGATAATATACTATTTGA
1913	DBF4	GTAAAAATTCTTCATATTGA
1914	DBF4	ATTAGTTGAAAAAGCTATCA
1915	DBF4	TATTATCAAATGCCTTGTCA
1916	DCAF10	ATGTTGATTTCAACGTCCTC
1917	DCAF10	ACAAGACTCTTAGTAACATC
1918	DCAF10	ATGACACTACAATAGCACTA
1919	DCAF10	TCACGAGCCTCTCAAAGAGA
1920	DCAF10	ACCACTTCATCAAGTTCATC
1921	DCAF10	CTAAAGACTTAGTTAAGTCA
1922	DCAF11	CCTACCTGGTGCAACATTCG
1923	DCAF11	GGGGATCGATACAACCCACC
1924	DCAF11	GGCTGGCCTGCGTCGGAGTG
1925	DCAF11	CCTCTTCCTCACTCCGACGC
1926	DCAF11	TCACTCGAGACTGTTCTCCA
1927	DCAF11	GAGAACGGGGCCTCTGCCAT
1928	DCC	GACTTCCTCGCCTCGTAACC
1929	DCC	AAATTCCAATGTCCCCCGGT
1930	DCC	GCAGATCAGCCGACTCCAAC
1931	DCC	GCGAGGAAGTCATCCAACTC
1932	DCC	GTCTAAAAAGTATTCTTTAT
1933	DCC	AATGTGACAGATGATGACAG
1934	DCST1	CCTACTAGTACCCAAGATGC
1935	DCST1	GCGCCACAGGAACCAGCTAC
1936	DCST1	CGCCTTGGCTGCCATCTATG
1937	DCST1	GCAGTGACAGGAAACTCGCC
1938	DCST1	GACATTAAACATCATCAGAA
1939	DCST1	GGCTGCCAGTCTCCTGTAGC
1940	DCTN4	TGAGCGAGATCGCAAGAAAC
1941	DCTN4	TGCTCGTGGCCCGAGTAGAG
1942	DCTN4	TCGCTGGACGTCTAGAGATG
1943	DCTN4	ACGTAGAAACTATATGCCTC
1944	DCTN4	TTACTTACCCGTTGTGTGTG
1945	DCTN4	CTGAAAATCCTCACACACAA
1946	DDB1	GGGGCTTCGGCCCGTCAAAG
1947	DDB1	CCTCACCGTGCTATTGATGC
1948	DDB1	GAAAGGGGCCGTGTACTCTA
1949	DDB1	CTCGAGACTTTAATCCCAAC
1950	DDB1	GTGCACCTTCAAAGGTGTGT
1951	DDB1	ATACATGCCCACCTCTTTGA
1952	DDB2	CCGAGATTGTATTACGCCCC
1953	DDB2	TGTGTCTGCTAGTAGCCGAA
1954	DDB2	ATACAATCTCGGAGGTCTTC
1955	DDB2	CACCATGCCGCAGCATCGTC
1956	DDB2	CTTTGCCGTCCATGTTCAGC
1957	DDB2	ACTAGGCTGCAAGACTTTAA

1958	DDC	CCTCTAGATGGTTGCCACCC
1959	DDC	AGCAGCATGTTGTGGTCCCC
1960	DDC	CAGGGCTTATCACTGACTAC
1961	DDC	AATTCACCAATAGCCATTTG
1962	DDC	TCAACTTTAATCCCCACAAA
1963	DDC	GACTTCTAAGAGATTGTCAA
1964	DDIT4L	GCTCCGTTGAGGAAAGCCGC
1965	DDIT4L	AACACAACCTCGCAAGCCGC
1966	DDIT4L	CCGTTGACCATGGTTGCAAC
1967	DDIT4L	CAAACTGCCAGTTGCAACCA
1968	DDIT4L	TCCTGAACCCAACCTCAACG
1969	DDIT4L	AGTAGAGGTCGCTTCTCCTC
1970	DDX56	GTCGCGACCTACGCTGATCC
1971	DDX56	CTCCTAGGCTGTCACCGATC
1972	DDX56	ATTCTTACAGACAGGTCCGG
1973	DDX56	CGTCCACCACCAAAAGCTCC
1974	DDX56	CTCACTGCCTGTTCTACCAC
1975	DDX56	GGAGCTGATATTACATAACC
1976	DDX6	TCTCTAGACCTGGTGATGAC
1977	DDX6	AGTGCCATTATTGATTGTGT
1978	DDX6	TAGGATGTGACCTCCACAAA
1979	DDX6	TAACATACCTGAATAGGAGA
1980	DDX6	GTGAAACCCACTGGTGGCCC
1981	DDX6	CTTGTATATTGTCCTTCTTC
1982	DEPTOR	CAAGTATGAGCGCACCTTCA
1983	DEPTOR	CACGATCTTGATCTCCTTGC
1984	DEPTOR	TGATGCCATGCTCCATAAGC
1985	DEPTOR	TCAAGATCGTGTCTGCAGTG
1986	DEPTOR	GCCTTACCGTAGCTGTTCCC
1987	DEPTOR	GAGTGGCGGGGCGCAGCAAA
1988	DESI1	GGAGCCGCCGAATCTCTATC
1989	DESI1	TACGTGTACGACCTGTCCAA
1990	DESI1	CTCATCCTTGTGCACAACTA
1991	DESI1	GAGTTCTTCTTCGGCAGTGG
1992	DESI1	ACCGGAACAGGGACTCCCCC
1993	DESI1	TCTCTCCACAGGGAAACAAC
1994	DGCR8	GACAGCGACCATCCGTCCGA
1995	DGCR8	CCGGACGAGAAAGACCCACT
1996	DGCR8	ATCTTTGCACACCTCGACTT
1997	DGCR8	GGATGACTTTGACAACGATG
1998	DGCR8	CCTACAGAGCCGCTGCCCGA
1999	DGCR8	ATCATGACATTCCATAACTC
2000	DGKK	TGCACCCAACCGGAAAGACA
2001	DGKK	CTACTTTGCACACCATCCCG
2002	DGKK	CTCGACAAACATTGCAGTGC
2003	DGKK	GGTACTCCAGTTACAGCCAC
2004	DGKK	CCTTATAAATTTCTCCCTGT
2005	DGKK	TGATCTGTCTCAAGCCACTG
2006	DGUOK	AATGTGTAGGACCATCGTGC

2007	DGUOK	GCCAGCCGGATCACATTACA
2008	DGUOK	CATCGAGTGGCATATCTATC
2009	DGUOK	CTCGAAGCCGACTTAGAAAG
2010	DGUOK	TCTGTAGCTACGTGCCATTC
2011	DGUOK	GAGCCGCCTGAAAGTACAGC
2012	DICER1	TACCTTCATAATTTCTCGAT
2013	DICER1	CCCCTATCGAGAAATTATGA
2014	DICER1	GCAGTTAGTCCCAAAATGCG
2015	DICER1	TCCATCATGTCCTCGCATTT
2016	DICER1	AGCCCACTTCTGTCAGTAAA
2017	DICER1	CAATCCACCACAATCTCACA
2018	DIP2C	CCTCTCGCTACCACCGCCGA
2019	DIP2C	CCATCGAGGTCTGCACGACC
2020	DIP2C	GGTCACGAGATGAGCGCTAT
2021	DIP2C	CCTGGTCGTGCAGACCTCGA
2022	DIP2C	CCATGACGTCCGCCAGCCTG
2023	DIP2C	GCTGCCCCCGCTCTGCGTAG
2024	DIS3	CGATTCGAGTAGCAGCAAAA
2025	DIS3	GCCTTGCTGTAGCTTACTTA
2026	DIS3	CCCTTAAGTAAGCTACAGCA
2027	DIS3	AGCAAGACGATCTATGAGTT
2028	DIS3	TTAGACTTACAAGTGAAAGC
2029	DIS3	CTTGCTTGTTTGTCTGAAGA
2030	DISP1	ATGCAGCCCTGATAGCCGAC
2031	DISP1	AACAGCAATAGGCCAGAGAT
2032	DISP1	TGACTTCTCTGATCCATTGC
2033	DISP1	TATTCCATGTGACCAATCTC
2034	DISP1	GACCTTTCAAGTTGCCAAAA
2035	DISP1	TGGCACTAATACTCCAACCA
2036	DLC1	TCACAGGAACGTGCCGCCGC
2037	DLC1	TTAGAACTCACCGTAGTCTT
2038	DLC1	CTGTAATTGCTCAGCAACGA
2039	DLC1	TCACTACCTGACTGCTGTCA
2040	DLC1	TCCACTCCAGTAGCCAATTC
2041	DLC1	GGTGCTGCTGATTATGAAAC
2042	DLD	TGTAGTTGCTGGTCCAATGC
2043	DLD	GCTATTAAAGCTGCCCAGTT
2044	DLD	ATAGCAGCAACATATCCTCC
2045	DLD	TGTGGTAGTATCTATGCCAT
2046	DLD	CATGAGATATTCGATTGAAA
2047	DLD	TGATCTGCGTAAGTTCTCAG
2048	DLG3	CGGGTCACCTCATAGCACTC
2049	DLG3	CAGAGTGCACCTGTACCAAC
2050	DLG3	ATGAGGAAATCGTACTTGAG
2051	DLG3	TTTATTACCAAGATTATCCC
2052	DLG3	CACCTGCGATACTGAAGCCC
2053	DLG3	CCCTAGGTGAATGGCAGTGA
2054	DLST	AGCCTACCAGGTTTGCCAGA
2055	DLST	AGCTAGTGGTGGGGCAACAG

2056	DLST	TCGCCCTCACAGCCTCCTTC
2057	DLST	AAGGTCTGCGTTCAGAACAT
2058	DLST	TACACAACCTTCCTGCTGTT
2059	DLST	TGCAGGGGTCTCCTTATGCC
2060	DMAP1	TACTCCTGCTCCGAGTACAC
2061	DMAP1	AGAACATTGCTCCGTCCTTG
2062	DMAP1	CCTTAGTCCAAGCATCATCG
2063	DMAP1	GTAGCTTACCTTATTGAACC
2064	DMAP1	ACAGGAGCTGCGCAAGATTG
2065	DMAP1	GACTTCCCGGTGCATGCCCT
2066	DMTN	CAGCACCTGATTGTCCATCT
2067	DMTN	CCCCTCACCTCGCGGCTGCG
2068	DMTN	GCTGGTCCGGGGGGTTCCAG
2069	DMTN	GGGGCTCACGCTCCCGGGGG
2070	DMTN	GGTACTCACCACGATGCTGG
2071	DMTN	CCTCCCATCTATAAGCAGAG
2072	DNAAF3	GTCGTCCGCTTCGATGCCGA
2073	DNAAF3	GTACTGGGGGGACATCGCCA
2074	DNAAF3	GATCACTCAACACAACGTGA
2075	DNAAF3	GGGCGCGCGAGAGCCACCGG
2076	DNAAF3	GCTGATCTTCAGCCTAGCCC
2077	DNAAF3	GGCTGAAGATCAGCATGTGT
2078	DNAH2	GGTGCGGGGCCCCTATATCC
2079	DNAH2	CCAGTCCTGTCAGCGCAGCT
2080	DNAH2	CTTTCCCGAGCTGCGCTGAC
2081	DNAH2	ATTCTTCCTAGAGCCACGGT
2082	DNAH2	CTCCACCGAGCAGCCGAAGC
2083	DNAH2	CAAGCCTACCACATCTGCCT
2084	DNAH3	CCATGACGTGCGTTGCATCA
2085	DNAH3	CCTTGATGCAACGCACGTCA
2086	DNAH3	CATACCTGATAGAGTCCAGA
2087	DNAH3	CCTTTCAGCGTTCAAAAGCC
2088	DNAH3	ACATCACATGAGCCACTCCC
2089	DNAH3	ACTGGCCGCCCCTCCCCATC
2090	DNAH9	ATGAGAAGAATCGCCTAAAC
2091	DNAH9	AGCTACTCTGAAGCTTGTCC
2092	DNAH9	GGATTCTCCCCTTGTAAGAG
2093	DNAH9	GGAGAATCCCACCCCTAAGG
2094	DNAH9	CTTGCTCAAGTATAACTGAG
2095	DNAH9	ATGTACAGAGATGTTGTTGC
2096	DNAJA1	CAGCTCGTTGAAGCACTGTG
2097	DNAJA1	AGTTCCCATACCTGGATGAG
2098	DNAJA1	GAGTGCTGTCCCAATTGCCG
2099	DNAJA1	TTAGAAGTTCATATTGACAA
2100	DNAJA1	ATATGACAAAGGAGGAGAAC
2101	DNAJA1	CACCATTATATAAGTCTTCT
2102	DNAJB7	CTTCTTTGAAGACTCGCTTG
2103	DNAJB7	TCCCACTATTATCAAAAGCC
2104	DNAJB7	AGCTTCCTGGACGATTTAAC

2105	DNAJB7	TGTATCTCAATATACTTTCG
2106	DNAJB7	ATGATGAGGGAGGTATATCT
2107	DNAJB7	ACTTCAGACAAAATCGTTAA
2108	DNAJC9	TTGGCGGCTACTCTTTAAAA
2109	DNAJC9	TGCTCACCCAAGACCGAGAC
2110	DNAJC9	TTATCATCAGATATCTTTAG
2111	DNAJC9	TAGCCTGAAGGCAGCCATTC
2112	DNAJC9	TTACCTGAATGGCTGCCTTC
2113	DNAJC9	ATTATAGGATGGGACCTCTC
2114	DNASE1L3	CTGTGACATCATACTCGTGA
2115	DNASE1L3	CCTGGAAGAACATCCGCTTG
2116	DNASE1L3	GCACCAACTGTGCATATGAC
2117	DNASE1L3	GGATCTGCTCCTTCAACGTC
2118	DNASE1L3	GTTGAAGGAGCAGATCCTCA
2119	DNASE1L3	ACAACTATGTGATTAGCTCT
2120	DNHD1	AGGTAGCGAGACGATGACAC
2121	DNHD1	AGGTAAAGGACTTGCGTAAG
2122	DNHD1	TAATACCTCACCTGCTAATA
2123	DNHD1	TAGGCTATCACCTAAAGTAC
2124	DNHD1	AAAAGCCGGTGCTGCTCTTT
2125	DNHD1	CCAAGAGCAGATGATTCTCT
2126	DNM3	GCAGAAACAGATCGCGTGAC
2127	DNM3	GTTACAATGCCCGACCCTCG
2128	DNM3	CCGAAAACGCGTCCTGCAGA
2129	DNM3	GCCGAGTTTCTACATTGCAA
2130	DNM3	GCTGCCGCAGATCGCCGTGG
2131	DNM3	CAGCTTGTTACTTCTAAAGC
2132	DNMBP	GAGCAGCTTACCTCGATGGA
2133	DNMBP	GACCATTCCCAGTCTAAAAG
2134	DNMBP	CTGCCGAAAATCCAGAGCAG
2135	DNMBP	TATTATTGAGGTGCTGGCAG
2136	DNMBP	GGCCACCAGTGATGAAGACT
2137	DNMBP	GCTAGGGCAGAAGTCAAAAA
2138	DNMT3L	AGGGATCTGCGCCCCATGTA
2139	DNMT3L	ACTCACCTTCTATATTTCGC
2140	DNMT3L	CACCAAAATCACGTCCATGC
2141	DNMT3L	CTCTCAAGCTCCGTTTCACC
2142	DNMT3L	CACACAGCACCCTCTGTTTG
2143	DNMT3L	TCTTATTGCATATGAAGTCA
2144	DOCK9	TCTCCCGTAAACAGTCGTTC
2145	DOCK9	TCAAAACCTATAACTCTGAC
2146	DOCK9	TCATGTCTATGAAGTTGACG
2147	DOCK9	CTTACCTGAAAGTCATCGTA
2148	DOCK9	TTATAGCAAAGTGGTCAAGT
2149	DOCK9	AAGATGCCAGTCAGAGTTAT
2150	DPP4	CTTAGAATACAACTACGTGA
2151	DPP4	CTAACCAATTTATGACCCAC
2152	DPP4	ACAACACACAGTGGGTCACA
2153	DPP4	TGAAGTTATACTCCTTAAGA

2154	DPP4	CTTACCTGAAATCCATCTTA
2155	DPP4	AAGAGAATAAACTGCCCATC
2156	DPP6	CTTCAAAATTCATGACCCCG
2157	DPP6	AAAAGTGCATACTCTCTATC
2158	DPP6	ACCACTTATCCACTTAGCCT
2159	DPP6	ACTTCTACTGTCTTAATAGA
2160	DPP6	TTCATCTACAGAGAACAGAA
2161	DPP6	TAGTCTGTCTCAAAAGAAGA
2162	DR1	TCCCGCCTGATTAGATGCAC
2163	DR1	CACCAGCTCTCGAGCATCGT
2164	DR1	GGTACTATGGCTTCCTCGTC
2165	DR1	CTCAGCCAGTGCATCTAATC
2166	DR1	AGTTCTCGTTTGGAAAACCT
2167	DR1	TGTCTCAATAACTCTTCTTC
2168	DRGX	GCTGAAGAGTGAACGTCGTC
2169	DRGX	GGGCGTAGGTGGCCGTGTTC
2170	DRGX	CAGGACCTACCGTTCGCCCC
2171	DRGX	GACGACGTTCACTCTTCAGC
2172	DRGX	TTTCATGGCGAGCTCTTCTC
2173	DRGX	TTACCAGCTGGAAGCTCTCG
2174	DST	GTCACTCCAGCTTATACACC
2175	DST	TTACACCCTAGTCTGACCTC
2176	DST	TCTGTACCAATTGATCTGCC
2177	DST	ATACTACCAGGCAGATCAAT
2178	DST	AGATTGCCAACAGAGTTCAG
2179	DST	ACTGTCCCTCTGAACTCTGT
2180	DSTN	GCTTGTATTGCTGAAAAGTT
2181	DSTN	AATGATCTATGCAAGCTCCA
2182	DSTN	AAACATGAATGTCAAGCAAA
2183	DSTN	GCAATTAAAAAGAAATTTCA
2184	DSTN	AGAAATCAAGAAAAGAAAGA
2185	DSTN	AAGTGCATCATTGTAGAAGA
2186	DTWD2	GTCGTCGCTTCAGTGAAGAA
2187	DTWD2	CTTGTTCCGACATCCCAAAC
2188	DTWD2	GGGAGGCATGCTGCTAGTAG
2189	DTWD2	CTGCACACTCCAGTGTAGAA
2190	DTWD2	ATATTAATGTACCAGACTTC
2191	DTWD2	GATGCTGAATTATGTACAAG
2192	DTX2	CTGGGGAGGTCAATAATGTA
2193	DTX2	GTGGCGGTAGCGGCCTGACA
2194	DTX2	AGCCAGCGTCTGTGACTATC
2195	DTX2	TGTGGCCGTGTGGGAATGGC
2196	DTX2	AAGACACAAACCTCATCTGG
2197	DTX2	GCCAGAGCCAGAGCCAGAGC
2198	DUS1L	GCCGCCTGAACAAACACCTC
2199	DUS1L	GTTTGTTCAGGCGGCTCTCC
2200	DUS1L	TCACTATGGCGCCTTTCTGC
2201	DUS1L	TCCTAGTTGCTGACGGTGCA
2202	DUS1L	CACAGCCTTGATATGCTCCC

2203	DUS1L	GACACCGCAGCTTCAGCTCC
2204	DUSP27	AGGACCTGTACAACCGCGTC
2205	DUSP27	CTATAATACGCCCTGTGTCC
2206	DUSP27	GGAGCAGGTAGTCCCAAGCG
2207	DUSP27	GGTTGTCATGGCGACCAGAA
2208	DUSP27	GCCAAACAGATCATCAATGA
2209	DUSP27	ACCTTCATTGATGATCTGTT
2210	DUT	CCTGTACAGGTCGTAGCCCG
2211	DUT	CCGCGGGCTACGACCTGTAC
2212	DUT	TTTGCAGCCAAGCCTGACCG
2213	DUT	ATTCAGATAGCGCTCCCTTC
2214	DUT	GTCATAGATGAAGATTATAG
2215	DUT	ACTCTTCCATAACACCCAGA
2216	E2F2	CGAGACTGGAAGTGCCCGAC
2217	E2F2	GCAGACAGTGATTGCCGTCA
2218	E2F2	GGTAGACTTCGATGGGCCCT
2219	E2F2	GAGCTTCAAGCACCTGACTG
2220	E2F2	GGAGCCGGACAGTCCTTCCG
2221	E2F2	TTTGAAGACCCCACCAGACC
2222	EDA	GCAACTCCGAGCGCAACTCT
2223	EDA	CTGCTACCTAGAGTTGCGCT
2224	EDA	CTACTTTCTTCTTCAGAGTA
2225	EDA	CAGGGAAGAAGAAATTCAAT
2226	EDA	CCCAGTTAAAAACAAGAAAA
2227	EDA	CGTGGAGCCGAGTCCCGCCT
2228	EDF1	TCGCGGACTATGAGAGCGGA
2229	EDF1	GAGCAAGGGGCTTACGCAGA
2230	EDF1	TCATAGTCCGCGATCACCTG
2231	EDF1	CTTTGTGTTCATTTCGCCCT
2232	EDF1	CATTGGAAAGCCCATCGAGA
2233	EDF1	GTGGGCAAGGTGATCCAGCA
2234	EFCAB14	GTCTGCAGCTGACTTAACAT
2235	EFCAB14	CTCTGAGTCCAGCTCCGAAG
2236	EFCAB14	TGACTGCTTGGGCCTTTCTT
2237	EFCAB14	TAGCCTGCCTACCACTGTAG
2238	EFCAB14	CATCCTTGCTGCCTGTGTTG
2239	EFCAB14	AAGATCTGTTATCCGCTCTG
2240	EFCAB3	GATGTGCACTGTAGCTAAGC
2241	EFCAB3	TACCCATCTCCCACAATAAA
2242	EFCAB3	CTACAGTGCACATCAGTCCA
2243	EFCAB3	GAAGCTAAGTGCTTCACAAA
2244	EFCAB3	CTCAGGGATAGAGACTTACC
2245	EFCAB3	GTCCTTGTAGAAGAAGTTGT
2246	EFHD2	CAACGTGTCCAGCCGCTTCG
2247	EFHD2	CCGCCGCCTTGCGGAAGATC
2248	EFHD2	CACCATGGCCACGGACGAGC
2249	EFHD2	TGAGATCGACGTCTCCAGTG
2250	EFHD2	AGACGCGGCGGCTGGGCGAC
2251	EFHD2	CAGCCCCCGCTACTTAAAGG

2252	EFNA4	CAATGTCTAGGTAATCGTTG
2253	EFNA4	GCCTCCGCCACGTAGTCTAC
2254	EFNA4	AGTTCCAGTAGACTACGTGG
2255	EFNA4	ATTGTCTGCCCCCACTACGA
2256	EFNA4	AAGCACTGGCCAGAACTCTC
2257	EFNA4	GTGCCCACTCCAGAGAGTTC
2258	EFNA5	TTTGTGCCGCGTTCTCGCCG
2259	EFNA5	TGGCTCGGCTGACTCATGTA
2260	EFNA5	TGAGCGCTATGTCCTCTACA
2261	EFNA5	CCGACCGCTACGCTGTCTAC
2262	EFNA5	GCCAGGCGTGATGTTGCACG
2263	EFNA5	GCCTGCGACCACACTTCCAA
2264	EGFR	TGAGCTTGTTACTCGTGCCT
2265	EGFR	GAGTAACAAGCTCACGCAGT
2266	EGFR	ATAGTTAGATAAGACTGCTA
2267	EGFR	GAATTCGCTCCACTGTGTTG
2268	EGFR	TGTGATCCAAGCTGTCCCAA
2269	EGFR	GACAGCTTGGATCACACTTT
2270	EGLN1	TCTTTACCGACCGAATCTGA
2271	EGLN1	TACTACCTTGTAGCATATGC
2272	EGLN1	GGCCATGGTTGCTTGTTATC
2273	EGLN1	GCAAACTGGGCTTTGCCTTC
2274	EGLN1	ACATAACCCGTTCCATTGCC
2275	EGLN1	TTAGGTACGCAATAACTGTT
2276	EGLN2	ACGTACCCGAGCCCGTTGCC
2277	EGLN2	TGATGCAGCGCCCATCGCCG
2278	EGLN2	TCACCTCGTGGGGGTTCCGC
2279	EGLN2	CCAGGTACGCCATCACTGTC
2280	EGLN2	TGGCTCGATGTTGGCTACCA
2281	EGLN2	ACCTAGCTGATACTTGTCTT
2282	EGLN3	CTATTACCTGGTTGCGTAAG
2283	EGLN3	AACAGGTTATGTTCGCCACG
2284	EGLN3	TGCAGCCCTCTTACGCAACC
2285	EGLN3	GATGCAGCGACCATCACCGT
2286	EGLN3	ACAGATATGCTATGACTGTC
2287	EGLN3	AGCACGGTCAGTCTTCAGTG
2288	EHD4	ACGACCTCATCAAGCGAGCG
2289	EHD4	CGGCCTTGTTCAGCACGACA
2290	EHD4	TCCTTCATCGCCGTGATGTA
2291	EHD4	CGAGAATACCAGATTTCTGC
2292	EHD4	TTTAGAAAGCTCAGTCGCTT
2293	EHD4	CGATGACGCTGATGCTCTTC
2294	EHHADH	CGTTACCTGATCGCGTTGAC
2295	EHHADH	TCGGAGGCGGATTAGCGCCA
2296	EHHADH	CAATCCTATAGTGACAGCCC
2297	EHHADH	ATCCAAGGCATGGCTTTCGG
2298	EHHADH	GTAATTAAGTCAAGTGCAGC
2299	EHHADH	TGGCTTACCAGAAGTTACAC
2300	EIF1B	TGAATAACCTCTCCGTATTC

2301	EIF1B	TCTTTCTGCCGTTCCGTTGC
2302	EIF1B	GTGACGACTTACTCCCGGCA
2303	EIF1B	GGATCTTACCGAAAGATTGG
2304	EIF1B	TTTACAGGTTGGCATTGTAA
2305	EIF1B	TTCAATCACAGTACCATTAC
2306	EIF2A	TCTTTAGTCCGAGGATCAGA
2307	EIF2A	ATTGACATAGGTGTCCATCC
2308	EIF2A	AAGAGTTTCATCTTCTGACC
2309	EIF2A	TGTACAGTCCTTCTGATCCT
2310	EIF2A	ACCTTGTTTGCCTGGGGCAA
2311	EIF2A	CTGAAAGAACAAGCAGCAAC
2312	EIF4EBP1	CCGCCCGCCCGCTTATCTTC
2313	EIF4EBP1	GTGAGTTCCGACACTCCATC
2314	EIF4EBP1	GTCGTGCTGTAGTCCCCGGG
2315	EIF4EBP1	GACTACAGCACGACCCCCGG
2316	EIF4EBP1	TGAAGAGTCACAGTTTGAGA
2317	EIF4EBP1	GTACCAGGATCATCTATGAC
2318	EIF5	AATGACCGTTACATTGTCAA
2319	EIF5	ACGTTGCAAAGGCGCTTAAT
2320	EIF5	GCACTTACCAAATCTGTTTC
2321	EIF5	CACACTCCAAACCATGAAGA
2322	EIF5	CAAGACAGTTATAGTCAACA
2323	EIF5	CATCATCAGCTGGTCGGAAA
2324	ELAVL2	TCCAGGACCGCTAGCTCAGC
2325	ELAVL2	TTCTCGTATTCTTGTCGACC
2326	ELAVL2	TCGATTTGACAAGCGAATTG
2327	ELAVL2	TGTGAACTACATTGACCCCA
2328	ELAVL2	GTTGGACAATCTGCTCAATA
2329	ELAVL2	TTTGAAGTCTCAATCCATTC
2330	ELL	GCGGGCAACCCCCATCAACT
2331	ELL	CACCGACGACTCCTACCAGA
2332	ELL	TGTCGCGGCCGATGTTGGAG
2333	ELL	GGCACCGCGTCTGTTGCACC
2334	ELL	CCATGCTCTGCCGCGCCTTC
2335	ELL	GGAGAACGTCCGCGCCTCTG
2336	ELP3	TCCGAGCCACCAATTCAACC
2337	ELP3	GATGCCTGACCTGCCAAACG
2338	ELP3	GAGTTACTCTCCTAGTGACC
2339	ELP3	ATGGGCCACCACTTTAAAAC
2340	ELP3	TTTCCTGTAAAACTGATGTG
2341	ELP3	CTCGAGTCCATGGAGGCACG
2342	EME2	GACTCGCTCGGGTCCACCGC
2343	EME2	GGTCTCGCCAGCACGTTTCC
2344	EME2	CTCCATCAGGACGTCGGCAC
2345	EME2	GGGCGTCGCCACACTGACCC
2346	EME2	GCTGACGGCCACCTCGGCAC
2347	EME2	CATCCAGCCCGATGACAGCC
2348	EMP3	ATCTCTGGTACGACTGCACG
2349	EMP3	TGTAGATGATGCCGCTGACC

2350	EMP3	GATCTATGCCATTCACGCCG
2351	EMP3	CAGCTCTACACCATGCGACG
2352	EMP3	GAAGAGACCTCCTCGTCGCA
2353	EMP3	ATTCTCGCTGACATTACTGC
2354	ENO1	TGACAAACTGATGATCGAGA
2355	ENO1	GCGAGAGTCAAAGATCTCCC
2356	ENO1	TCGCGGGAATCCCACTGTTG
2357	ENO1	TTCAGAGCTGCTGTGCCCAG
2358	ENO1	AGCGAGTCTTATCATTGTCC
2359	ENO1	CTATTCTCAAGATCCATGCC
2360	ENO2	CCCGTGCCGTTCTGAACGTC
2361	ENO2	ACTGCCCGCTCAATACGTTT
2362	ENO2	GGAGACAAACAGCGTTACTT
2363	ENO2	CCAACCCAAAACGTATTGAG
2364	ENO2	GATGCCCGTAGAGGCTCCAC
2365	ENO2	GGGACCCTCACCTCATGAGC
2366	ENO3	AGTCGCCTGATGATCCCGCA
2367	ENO3	CGCCACATCGCAGATCTCGC
2368	ENO3	TCCCAGCGAGATCTGCGATG
2369	ENO3	CAGAGCCTCATAGATACCCG
2370	ENO3	GCATCTGAGTTCTATCGCAA
2371	ENO3	GGCTTCCACGGGTATCTATG
2372	ENPP3	CATGTCACTTGGATTAGGCC
2373	ENPP3	TTAAGAGTGTTCTTCTTAAC
2374	ENPP3	ATCTACGTTGACTTTAGCAA
2375	ENPP3	AAGACCGAGGTGATTGCTGC
2376	ENPP3	ACTCATACTTGATTCCACAC
2377	ENPP3	ATAGGTTCTTCTTGCTTTGC
2378	ENTPD8	CGTAGAGGCGAAAATCGGCC
2379	ENTPD8	ACCCAGATCACGTTCGTGCC
2380	ENTPD8	TGTTCGCCAGCCACTGATAC
2381	ENTPD8	GGGACGCACCTTGATGTCTG
2382	ENTPD8	CGTCCCTCTTCCTGTATCAG
2383	ENTPD8	CGTGAGGCCTGAGACCCCCG
2384	EP300	CTGTCAGAATTGCTGCGATC
2385	EP300	CTTGGCAAGACTTGCCTGAC
2386	EP300	TAGTTCCCCTAACCTCAATA
2387	EP300	ATGCTCACAAGTGCCAGCGC
2388	EP300	ATGTTGGGCATTCCTCCACC
2389	EP300	ATGAACAACCCCAATCCTTA
2390	EPAS1	GCTGATTGCCAGTCGCATGA
2391	EPAS1	CAAGGCCTCCATCATGCGAC
2392	EPAS1	CATGAAGAAGTCCCGCTCTG
2393	EPAS1	TTGGTTCACACATGATGATG
2394	EPAS1	TAGCCACACAGACTATTGTG
2395	EPAS1	TCAACCTCAAGTCAGCCACC
2396	EPM2A	GTGTACGTGCACTGCAACGC
2397	EPM2A	TTACATGTTCCACCTGACGA
2398	EPM2A	TTACCTTCGGTGCTCATATC

2399	EPM2A	GGCTGTCTACATTGACGAAG
2400	EPM2A	TGTACCAGAACGTGTCCACG
2401	EPM2A	CCCGCCATGCGCTTCCGCTT
2402	EPOR	ACGGTGATATCGCGGAGCGC
2403	EPOR	GCGCACCGCACCACGAGCCG
2404	EPOR	CCGAGACGTCCACCTCGTAG
2405	EPOR	GGAGCCAGCGCAACACTACG
2406	EPOR	GTGGGAGAGCAGCGCGAGCA
2407	EPOR	TCGCGCTGCTCTCCCACCGC
2408	EPPIN	CGTCCTCTTAGCGAATGTCC
2409	EPPIN	GCAGCCACCATAGACAAACA
2410	EPPIN	AATAACCAATCAGTCAGACC
2411	EPPIN	ACTTGCTCCATGTTTGTCTA
2412	EPPIN	CACAACACTTCTTGTTGTCC
2413	EPPIN	AAGTGTTGTGTCTTCAGCTG
2414	EPS8	TCATAGCATCTTTCCGATCC
2415	EPS8	GCACTTGACTACCTTTGTCC
2416	EPS8	TCAACTTACTTCATCTGAGA
2417	EPS8	AACAAAGGAAGAATTATGCA
2418	EPS8	TCTAATCATCCCAGTAGTTT
2419	EPS8	TTGAGTCCACACTTTGCCCT
2420	ERBB3	CACTGTACAAGCTCTACGAG
2421	ERBB3	TTCTCTACTGGCGTGGGCGC
2422	ERBB3	GTCCCGTGAGCACAATCTCA
2423	ERBB3	TGGTAGGCGCTATCTCCGCG
2424	ERBB3	ACCTTTATGACCAGATACCG
2425	ERBB3	CCGAGACCCACCACGGTATC
2426	ERC2	AATGCAGCCTATGCTACGTC
2427	ERC2	GCTACAAATCGAGCTGTATA
2428	ERC2	GGACACAAAAATCGCTTCAT
2429	ERC2	AGTGCCTTACCTCAGTCTGA
2430	ERC2	ACGAAACATAAGGGATCTTG
2431	ERC2	CACTTACTGCCAAAGAACAG
2432	ERCC1	AGTTCGTGCGCAATGTGCCC
2433	ERCC1	GCTCTGTGTAGATCGGAATA
2434	ERCC1	CGTAATTCCCGACTATGTGC
2435	ERCC1	ACTGCACATTGATCCTCGCC
2436	ERCC1	CATATTCGGCGTAGGTCTGA
2437	ERCC1	AGCCGCCCATGGATGTAGTC
2438	ERCC2	TTATCGGCAGGCATATCCGC
2439	ERCC2	ACTGGCCCGCAAGGCCGTCG
2440	ERCC2	TTCGACGAGGCCCACAACAT
2441	ERCC2	GGGCGAGAAGCTGCCGTTTC
2442	ERCC2	GCTCAACTTCTATGAGAAGC
2443	ERCC2	CAGCTTACCTTCTCAATCTC
2444	ERCC3	AAACGGGCGTGCACGTTCGG
2445	ERCC3	CCTCAGCCAAGATGTTCCGA
2446	ERCC3	GCACGATGGTGAGCACCCTT
2447	ERCC3	ATCCTCATAGTGCCGCTTCC

2448	ERCC3	ATGATGAAGAGGACGCCCCG
2449	ERCC3	CAGGGTTGACAGAATCATTC
2450	ERCC4	GTGTTATATTTGCGACAAGT
2451	ERCC4	TTAGACAATCCGCCATTATC
2452	ERCC4	AAATCTTCCACTTCAAGCGA
2453	ERCC4	GACAAGACTCGATTATTCTG
2454	ERCC4	CCATAACCCATCGCTTGAAG
2455	ERCC4	CAAGACTAAATCCTTAGTTC
2456	ERCC5	GACTCAATATCTATCGCTTG
2457	ERCC5	AGCGATAGATATTGAGTCTG
2458	ERCC5	GCATATAGAACATGTCCAAA
2459	ERCC5	AGATGAAGGGGGCTTTCTGA
2460	ERCC5	AAATAATGTTCTTCTGCGCT
2461	ERCC5	ATTATTTGAAGCAATGCCAG
2462	ERCC8	GCCAAGATATAGTCATAACG
2463	ERCC8	CAGTGGTATCCTCATGACAC
2464	ERCC8	TCCACGTTATGACTATATCT
2465	ERCC8	ACACTGTATCTGTGAACATC
2466	ERCC8	TGTGTAATAAGATTGTCTGC
2467	ERCC8	TGTAAAGCAGTGTGTTCCAT
2468	ERG	GATAACTCTGCGCTCGTTCG
2469	ERG	CGCAGAGTTATCGTGCCAGC
2470	ERG	CCCTTTCAGATCCTACGCTA
2471	ERG	AAGGATGTCGGCGTTGTAGC
2472	ERG	TTTCCTTACCTTAATAGTGC
2473	ERG	CATTATGGCCAGCACTATTA
2474	ESD	TCCCCCAAAGCACTTGTTGC
2475	ESD	CCTGAGAGCCAATACAGTGC
2476	ESD	AATGTACTCTTATGTCACAG
2477	ESD	TGCTGTCTACTTACCACCAA
2478	ESD	ATTTCCAGCAACAAGTGCTT
2479	ESD	ATAGGTGGCTGCAATATTAA
2480	ESR1	GCCAGACGAGACCAATCATC
2481	ESR1	CTCCGTAAATGCTACGAAGT
2482	ESR1	GGGATACGAAAAGACCGAAG
2483	ESR1	TCATACTCGGAATAGAGTAT
2484	ESR1	AAATTCAGATAATCGACGCC
2485	ESR1	TACCTGTCCAAGAGCAAGTT
2486	ETFDH	GTATAATGGGTAGTAATTCG
2487	ETFDH	TTGTATCCCTACATCGTTAG
2488	ETFDH	CCCTTGGACAGACATACCTA
2489	ETFDH	CTCCTTCAGTCCAATCCCGT
2490	ETFDH	ACAAAAGGATGGTGCACCAA
2491	ETFDH	CCTAGTAGCTCTTGGTCTTG
2492	ETS1	GAGAGTCGGCTTGAGATCGA
2493	ETS1	TGGAAACCACAGTTCATTCG
2494	ETS1	GAAGATCCTCGAATGAACTG
2495	ETS1	GACTCTCACCATCATCAAGA
2496	ETS1	CACTAAAGAACAGCAACGAC

2497	ETS1	ACGAGGCGCTGAGTAAGGGA
2498	EVX1	CGGTGCGGTAACGACGCATC
2499	EVX1	CGGCGGCCCCTTATCTAGTG
2500	EVX1	ACCGGGAGAACTACGTATCC
2501	EVX1	AGCACGCGGAACGTGTCGAG
2502	EVX1	GCGTTCCCGGTGGCGCAGTC
2503	EVX1	CTCAAATTTGTCCGAAGCCG
2504	EXOC5	ACATCACTTACTTGCAATTT
2505	EXOC5	GCCTATCAAAAGGTAACTCT
2506	EXOC5	TGTTTATATAAAGCAGTGCC
2507	EXOC5	TTATATAAACATCAACACAA
2508	EXOC8	GCGAGTTCGACAGCTCACTG
2509	EXOC8	ATTCGTCAGCTTCGCATCGA
2510	EXOC8	TCGAACTCGCTCCTCCACTT
2511	EXOC8	GGGCCGTCTCTATGAACTGC
2512	EXOC8	GGCCCGCGAGATCTCCTACC
2513	EXOC8	TTATAGTCTTCGATGTGAGC
2514	EZH1	ACAGGCTTCATTGACTGAAC
2515	EZH1	AGCTGATCAATAACTATGAT
2516	EZH1	CCTCATCTGAGTACTGATTC
2517	EZH1	AGAAAGCGACATGCTATTGA
2518	EZH1	TACCACTACCTTCTTCACCA
2519	EZH1	GCTTTGCTAGGCTTTGTATG
2520	F8	GCACTGTACAATCTCTATCC
2521	F8	TCTGCTAGGTCCTACCATCC
2522	F8	CTCAGGATCAAAGGATTCGA
2523	F8	CCAACAGCATGAAGACTGAC
2524	F8	TTGGCTTAGCGATGTTGAAA
2525	F8	GACTCTGTTTGTAGAATTCA
2526	FAAH2	CACCGGCCGAGGGGTCTTTG
2527	FAAH2	GACGTGAACCCAATGATCAA
2528	FAAH2	TGAATGCGGGCGGTAAATGA
2529	FAAH2	TAGGTTTGAGGAAGCGATGA
2530	FAAH2	TGGGGTTCCTTTGACAGTCA
2531	FAAH2	GAAATGTATAGATGTTGTTC
2532	FABP5	CAAAGCCTTTGCTGTCCACC
2533	FABP5	CATGGCCACAGTTCAGCAGC
2534	FABP5	GGGAATAGCTTTGCGAAAAA
2535	FABP5	TTTCTTCAAACTTCTCTCCC
2536	FABP5	ACTGTCTGCAACTTTACAGA
2537	FABP5	CATTGGTTCAGCATCAGGAG
2538	FAHD1	GGAACTACGCGGACCACGTC
2539	FAHD1	TGCAGGTTGCGAGTGTACGC
2540	FAHD1	AACACTTCCTGTGAGACTAC
2541	FAHD1	CTTCATTCCTGTAGTCTCAC
2542	FAHD1	CCCTCAGCTTCCCGTTTCTG
2543	FAHD1	ACCTCAGAAACGGGAAGCTG
2544	FAM107A	CCCCCGAGTTTATTAAAGTC
2545	FAM107A	AGCGTGTCCTAGAGCACCGC

2546	FAM107A	TCAGTGTGGCAATTCTCCGC
2547	FAM107A	GAAGCTGCTGAACCCCGTGA
2548	FAM107A	CAATGTCTGCCCGCTCCCTC
2549	FAM107A	TCTAGGACACGCTGCAGCTC
2550	FAM172A	TTCATGGTAGTGGTGTTGTC
2551	FAM172A	GAAGTATGTATATGAGCTCC
2552	FAM172A	ATGGAACCAGAAAAGATACG
2553	FAM172A	ACCGTTTATTAAAAGAGCTG
2554	FAM172A	AAGCATATTCAAATCCTTCC
2555	FAM172A	TCCATCTGTGTAAATCTTCC
2556	FAM174A	GCCAGGGGCCCGCTTAGTTC
2557	FAM174A	GGCAGCGGCGGTAATGTCCG
2558	FAM174A	TTGATGCCAATCATCCTCGA
2559	FAM174A	TACTTACCTTCGAGGATGAT
2560	FAM174A	GTGATCGTCCGTCTCAAGCC
2561	FAM174A	GACATTACCGCCGCTGCCAC
2562	FAM200A	CGTACCATGTCCATACATGC
2563	FAM200A	TATCAGTGCTCTCATCGAGT
2564	FAM200A	TGTTTCATACAGAAGTTCGT
2565	FAM200A	ATGACTTTCCACTGCTAAGT
2566	FAM200A	TGCAAGTTGTCCCACACTCT
2567	FAM200A	CTGTACGATTGCAAAACATT
2568	FAM20A	GGTTAATACCCAGGTGGAAC
2569	FAM20A	GCAGATGAACCTTACCTCCC
2570	FAM20A	ATGAAGTAGAAGAAGTCCAC
2571	FAM20A	TTCCAGACAGCAGCGAGATG
2572	FAM20A	TTACCTCATGGGTTTGAACA
2573	FAM20A	AGGCCATGTTCAAACCCATG
2574	FAM219B	AGGTCCAAGTCCTCGTCATC
2575	FAM219B	TAATGTCTACTGGAGGGTAC
2576	FAM219B	CCGAACCGCCCAGACTCTTC
2577	FAM219B	ACCTGGCCAAGGCCGTTCTG
2578	FAM219B	GCACTCACCTGTCAGAGTCG
2579	FAM219B	CTCCGGGTATTCATCTGCAG
2580	FAM53B	CAATGTCCAACTCGCCGTCC
2581	FAM53B	GGACTCCACCACCGATTTGG
2582	FAM53B	CCTCCATGATGTCCGGCAAC
2583	FAM53B	GTTGCTGACGTGGCGGGCGA
2584	FAM53B	CCTCACCAGTTCACGGCTGA
2585	FAM53B	GGAGCTGACTCCATTGCATG
2586	FAM72A	GCATGACTTACTTGGTAGGA
2587	FAM72A	TACCAACATTTGTAGTTTCA
2588	FAM72A	CAGTTTACATTTGCAGATTT
2589	FAM72A	TCCAGCGCAGTGGACTTCAC
2590	FAM72A	TGTTAATATCATAAACTGCC
2591	FAM72A	CCAGAAGTGTCCGTTGTTGC
2592	FASN	CCTTCAGCTTGCCGGACCGC
2593	FASN	GGATGGTGGCGTACACCCGC
2594	FASN	CGACCCACCTCCGTCCACGA

2595	FASN	CTGCAAGGCCTTCGACACAG
2596	FASN	GGCTGTGTCCAGTGCGATGC
2597	FASN	TACGCCACCATCCTGAACGC
2598	FASTKD2	ATGCACCGATTGCTCTTAAG
2599	FASTKD2	TCACCGATCTCTTATACTCC
2600	FASTKD2	GAATGCAGTAAGGTGGTCCT
2601	FASTKD2	CAAGTTGTGATGAAGTGTAT
2602	FASTKD2	GAATGTTCATGTTCTACGAA
2603	FASTKD2	AGAACATGAACATTCTTGCA
2604	FBF1	GTATGTGAAGCTAGTTTAAC
2605	FBF1	GAACATCTGAGATACACCTG
2606	FBF1	TAGGCACCAAAAACCAAGAA
2607	FBF1	CCCCTTTGCAGGTCCCTCCT
2608	FBF1	TGCTGAAGACATCATCACCC
2609	FBF1	CTCACCTTTACATCCTTTCT
2610	FBN2	TCTAGCGCCCGAGTATCGCG
2611	FBN2	GGCACCCTCCTCGCGATACT
2612	FBN2	CATCACAGGCCCAACGTGTG
2613	FBN2	AGCACTCACGGACAATGCAC
2614	FBN2	CTGGCAGTGGTCATCTGCAC
2615	FBN2	AAGTTCCAATATATCCTTTC
2616	FBP1	ATCGATTGCCTTGTGTCCGT
2617	FBP1	TTCTGACACGAGAACACACG
2618	FBP1	CACCAAAATGAACTCCCCGA
2619	FBP1	CCGTCACTGAGTACATCCAG
2620	FBP1	AGATAAACACGCCATCATAG
2621	FBP1	GGACACAAGGCAATCGATGT
2622	FBXO15	TGCGTACCTGAGGGCCTCTT
2623	FBXO15	AAACCTGTCAACCCTTACAC
2624	FBXO15	ATGCCGGAGGTGGACAGCAC
2625	FBXO15	CTCCCAGTGCTGTCCACCTC
2626	FBXO15	GCTGTGAGCCTTCTGTGTAC
2627	FBXO15	TCACACATCCAGTACACAGA
2628	FBXO3	TGTTCATACCGAATTCACAA
2629	FBXO3	CTTTATAGAGGGTGCTCGAG
2630	FBXO3	TATGTCAAAAATGTTGTATC
2631	FBXO3	GCTATTGACATGTTTATTAT
2632	FBXO3	ATAAACATGTCAATAGCAGC
2633	FBXO3	AAATAAAATACCTGAAAATT
2634	FBXO41	GGACCGCTCTGAGCCCCCGT
2635	FBXO41	GTATTCTGCAGCCCGCCCCG
2636	FBXO41	ACCCCGCCGACACAGCACTG
2637	FBXO41	CAGCCCCTCACGCTCCAATG
2638	FBXO41	CATGGCTCCTCTCCGAGCAC
2639	FBXO41	GGCTTACCCTGTGCTCGGAG
2640	FBXO43	GACTCCGATAAGTAATCTTG
2641	FBXO43	TGACCGCAGAGAGCCTATGC
2642	FBXO43	CTTAGATGTCAAAGTTACGT
2643	FBXO43	ACAACTGAAAACAGATTCTG

2644	FBXO43	AGATGAGAGAATTTCTTGTT
2645	FBXO43	TTTGGAAAGTAAGCAGAAAT
2646	FBXO48	CCCCATGTATCTGCATCCAT
2647	FBXO48	GCAGGGCTTCATTGACATGC
2648	FBXO48	TCACACAGAAGCGAACTCTG
2649	FBXO48	TCTTTCAGGTGATACTGCTG
2650	FBXO48	CGTGTTTCACTTTACTCTTC
2651	FBXO48	AGAGTAAAGTGAAACACGAA
2652	FBXW11	CATCACTTTACGCCGTGTCC
2653	FBXW11	GTGGCTAGGCTGCGCCAACC
2654	FBXW11	CTGATGATCCACTAACAACC
2655	FBXW11	GCCCAGTGTCAGATGTCTCC
2656	FBXW11	GTCAAAGTCTACTACATTGA
2657	FBXW11	GCATTGCCTGTCTCCAGTAC
2658	FCGR1A	CTGGGAGCAGCTCTACACAG
2659	FCGR1A	CACTGTGTAGAGCTGCTCCC
2660	FCGR1A	TCACCCACTTGCCCATCAAC
2661	FCGR1A	TCTCTTGCAGTTCCAGTTGA
2662	FCGR1A	GCTTTCCCATGCCTGAGCAA
2663	FCGR1A	TTCAGGCTGGCTACTACTGC
2664	FCGR1B	TCCAGCAGAGTCTTCATGGA
2665	FCGR1B	TCCTTCCATGAAGACTCTGC
2666	FCGR1B	TCTCTTGCAGTTCCAGTTGA
2667	FCGR1B	TCACCCACTTGCCCATCAAC
2668	FCGR1B	TCTCCAAGTAGACACCACAA
2669	FCGR1B	TCTGTGGATTTCCAGCTGTA
2670	FCGRT	ACCGCCAAGTTCGCCCTGAA
2671	FCGRT	TCATGAACTCCTCGCCGTTC
2672	FCGRT	CAGGCGAGGACACCGCGGTA
2673	FCGRT	CCCGCAGGCTATTGTAGCTC
2674	FCGRT	TCGGGCCTTCAGGCGCATGG
2675	FCGRT	GACGATTCCCACCACGAGCA
2676	FCHO2	AGATACCTATAAACTCTATG
2677	FCHO2	ATTTAACAGAGCTACCATAG
2678	FCHO2	GCAAGCAATTATTCACAACT
2679	FCHO2	GAAAATGACAGAAACAGCTC
2680	FCHO2	CAAAAATTTGCTGAGTCAAA
2681	FCHO2	TGTTATTTATAAATTCTTCA
2682	FCN3	TCGCATAGTGGGCGAAAGTA
2683	FCN3	TGAGGTAGACCACTACCAGC
2684	FCN3	GAGATCCAGTGAACCTGCTC
2685	FCN3	GTCCTCCTGCCCAGTTGTCC
2686	FCN3	CCCCTCTCACCTTGAGGACC
2687	FCN3	CCCATCTTGCCTGGTGGTCC
2688	FEN1	AGCCCGCCGCTCACTGCGTT
2689	FEN1	GCCGTTCTCCATCATGCGAA
2690	FEN1	CTACCGCACCATTCGCATGA
2691	FEN1	AAGCCGCCACAGCTCAAGTC
2692	FEN1	ATGAGGTCCACAGCCCGCTT

2693	FEN1	AGGCTGAGTAAGAGCCGCCA
2694	FGF17	TGCTGTAGAGTTGGTACTCG
2695	FGF17	GGTGGCGGAGATGCGACGCC
2696	FGF17	CAGCCCCTTTGATGCGAACC
2697	FGF17	AGTACATCTGTATGAACAAG
2698	FGF17	GCACACTTACAGAGTGAGGT
2699	FGF17	GATTCTCTGCTGTCAAACTC
2700	FGF5	GAGAAGCGTCTCGCCCCCAA
2701	FGF5	CTGCCGGTCCGGCGCCCCGA
2702	FGF5	GGAAATATTTGCTGTGTCTC
2703	FGF5	CTTGCAGTCATCTGTGAACT
2704	FGF5	AGCCCCTCGGGGCGCCGGAC
2705	FGF5	AGCGGGTCCGGGTTGCCCTT
2706	FGFR3	GGGGACGGAGCAGCGCGTCG
2707	FGFR3	GACGCGCTGCTCCGTCCCCA
2708	FGFR3	CGCGCTGCGTGAGCCGCTGC
2709	FGFR3	CCCGTCTTCGTCATCTCCCG
2710	FGFR3	CCCCACCAGGACACGCTCCG
2711	FGFR3	CCTTGCAGACGCTCCATCCT
2712	FGL1	GTTTATTGTGACATGTCCGA
2713	FGL1	GACCCGGGTCAAACAGCAAC
2714	FGL1	GTAATTCAGAGACGATCTGA
2715	FGL1	GAGAATACTGTCATTGATCT
2716	FGL1	GGATGGAAAGACTATGAAAA
2717	FGL1	CTTCACTTCTTGACCACTCA
2718	FH	CCAGTCTGCCATACCACGAG
2719	FH	AGCGGCCGCTGAAGTAAACC
2720	FH	AAAGTTCATCGTAGATCTCA
2721	FH	GCGCCATAATACTTATCATT
2722	FH	CGATCATGTTAATAAAAGCC
2723	FH	ACCCCAGTTATTAAAGCTTT
2724	FITM2	CCAGGGCAGGTAGCGCCGCA
2725	FITM2	GCCCGTGTAGTGTTCGATGT
2726	FITM2	GAGATCCGGCATGGAGCATC
2727	FITM2	TTTCCACAACTTGTCCCAGA
2728	FITM2	GCAACGGGGACAACTCCTTG
2729	FITM2	GGACGTTGCGCTTGTTGCTG
2730	FKBP11	TCGAAACCGAAAGTCCCGTC
2731	FKBP11	TTCTATAACCAGAGGGTCTC
2732	FKBP11	GTCTTCTCGACATGTGTGTG
2733	FKBP11	TCCTCACCAGGGTCTCCACT
2734	FKBP11	TATAGAACTTGGCCAAAAGC
2735	FKBP11	CCCCAGGGAAGCTTGGTAGA
2736	FKTN	GTGAGTCTATCCCGTCTAGC
2737	FKTN	ACTTACCTGTCAAAAGCTCC
2738	FKTN	GTGTGCTATCAAATCCAATT
2739	FKTN	TTGCACTGCAGTATCACCTA
2740	FKTN	TCTTATTGATCCTTTGATAC
2741	FKTN	TTGGATTTGATAGCACACAG

2742	FLCN	TGCTCCGACCGAGGATACCT
2743	FLCN	CGCCCGTTACCAGGCAAAGG
2744	FLCN	GGAATCATCGATGAGCTCCA
2745	FLCN	CTGGTGGCTGACGTATTTAA
2746	FLCN	TGATGATGCTGTACCAGCGC
2747	FLCN	TCACGCCATTCCTACACCAG
2748	FLG	TGTATCGCGGTGAGAGGATC
2749	FLG	GTCAGACTCTAGTACCGCTA
2750	FLG	CCAAAAGATGTCTACTCTCC
2751	FLG	ATTGAGTAAAAAAGAGCTGA
2752	FLG	TCATCTGCAGTCAGCGATCG
2753	FLG	GTCATTACGAGTTTGTCTGC
2754	FLRT3	GCGTAGTTCTTCTATAGTCC
2755	FLRT3	AAGCAATGAACCCATCTCGA
2756	FLRT3	GACTATAGAAGAACTACGCT
2757	FLRT3	CCACGCACGTTGACCTTCAC
2758	FLRT3	CATCTTGCACCCGCAATACC
2759	FLRT3	ATCTGTGTGTCGCTGCGATG
2760	FLT1	TTGGTCAATTCGTCGCCTTA
2761	FLT1	TTCCCTCGCCGGAAGTTGTA
2762	FLT1	TGGTCCTACCATACAACTTC
2763	FLT1	AGACACTGCATCTCCAATGC
2764	FLT1	TATACTTGTCGTGTAAGGAG
2765	FLT1	ACCCAGCACATCATGCAAGC
2766	FMO2	TCTGAGTGACAACCTTCCAT
2767	FMO2	TGCTGTCTTTGACGCAGTTA
2768	FMO2	AGAGATACGGCTCATGACCC
2769	FMO2	TGCGGAGCATAGAACGAAAC
2770	FMO2	ATTTGAGGGAAAACGCATCC
2771	FMO2	ATTGGCGGCTATGGAAATAT
2772	FN1	GACCTACCTAGGCAATGCGT
2773	FN1	TACAAACCAACGCATTGCCT
2774	FN1	GCTCATAAGTGTCACCCACT
2775	FN1	GAATGGACCTGCAAGCCCAT
2776	FN1	TCACACACCTATGGGCTTGC
2777	FN1	GACTGTACCTGCATCGGGGC
2778	FNDC7	GTGTCCATTATGCGAGCCAA
2779	FNDC7	GAGAGTTCCGGCTTCTAAAC
2780	FNDC7	ATCAGATCCATCAGCGCTGC
2781	FNDC7	TGAAATACCCACTATTGATC
2782	FNDC7	CAGGTTGCTTCAGCAAAATC
2783	FNDC7	ATTCCATGCATAGGCCTTTA
2784	FOXA1	GTTGGACGGCGCGTACGCCA
2785	FOXA1	GTAGCTGCGCTTGAACGTCT
2786	FOXA1	CAGCTACTACGCAGACACGC
2787	FOXA1	GTAGTAGCTGTTCCAGTCGC
2788	FOXA1	CATGTTGCCGCTCGTAGTCA
2789	FOXA1	ACTGCGCCCCCCATAAGCTC
2790	FOXB1	CGGCTTCAACGGCACGCCGT

2791	FOXB1	CCACACGTGTATGGCTCCGC
2792	FOXB1	TCTGGTCGCTGTACGTGTTG
2793	FOXB1	CCGCGCGATGATGTTCTCGA
2794	FOXB1	CCATCGAGAACATCATCGCG
2795	FOXB1	GCAGCGTTTGGCCCGCCGCG
2796	FOXE1	CACGAACCGATCTATCCCAC
2797	FOXE1	GCGCCCGTAGAAGTCCACCG
2798	FOXE1	CTCGAACATGTCCTCCGCGT
2799	FOXE1	GACGCCGCGGGGTAGTAGAC
2800	FOXE1	CGGCCTCGCGCGGGATCTTG
2801	FOXE1	CCCGGCTTACATGCACGACG
2802	FOXH1	TGGCAGAACGGAGGTGCGCG
2803	FOXH1	TGGCCGGCCGTGCAGCACGT
2804	FOXH1	GCGGTTTCAGATCATCCGTC
2805	FOXH1	GGGAGCGGCCTGAATCACCA
2806	FOXH1	CTCCAACCGATGCTTCCGCA
2807	FOXH1	CAAGCCCCCCTACACCTACT
2808	FOXM1	CATGCCCAACACGCAAGTAG
2809	FOXM1	TCTCCCGTTTCTGCTCGCAA
2810	FOXM1	GCGGCCACCCTACTCTTACA
2811	FOXM1	CTTCGAGACCATCAGCGTCC
2812	FOXM1	ATAGCCTATCCAACATCCAG
2813	FOXM1	AACTCATCTTTCGAAGCCAC
2814	FOXN3	GCCTGACATCCGATTAGAAG
2815	FOXN3	CCCTCTTCTAATCGGATGTC
2816	FOXN3	TCACAACCTGCTTGTATCTC
2817	FOXN3	GCACATCAGGTCCACCCATC
2818	FOXN3	GCTTACCTTTGATATGCCTG
2819	FOXN3	ACTCCCCCTGAGATACAAGC
2820	FOXP3	CCCACCCACAGGGATCAACG
2821	FOXP3	TTCGAAGACCTTCTCACATC
2822	FOXP3	AGCTCTGGGGCACAGCCGAA
2823	FOXP3	CCACTTACAGGCACTCCTCC
2824	FOXP3	CCTGGACACCCATTCCAGGC
2825	FOXP3	TGCCCCCCAGCTCTCAACGG
2826	FZD10	CGCGAACTCGTGCAACTGGA
2827	FZD10	GGGCTCGTCCGAGCCGTTGT
2828	FZD10	GCTCAAGTGCTCCCCGATTA
2829	FZD10	GGACGTGTACTGGAGCCGCG
2830	FZD10	AAAGTAGCAGGCGATCACAC
2831	FZD10	CGCCCGTCTTCATCACCCTC
2832	FZD5	TCGCGACACTTGCACACGAA
2833	FZD5	CAGAACCTGAACTCGCTGCG
2834	FZD5	CGCGCGCCAAGCCCGAGTAC
2835	FZD5	CCATGCCGAAGAAGTAGACC
2836	FZD5	AGCAGCACTACCGCGAGAGC
2837	FZD5	AGCGGGGCATTCGCCCCCCG
2838	FZD9	TGCTACCGCAAGATAGCAGC
2839	FZD9	ACAAGGACTTCGCGCTGGTC

2840	FZD9	TGGCACGCACTGCCACTATA
2841	FZD9	GGCGGCGCGGCACTGGAGAT
2842	FZD9	GGGCGCGCTCTACGTGATCC
2843	FZD9	CGTGCCACGTCCGTAGCTCC
2844	GAB1	GGGATACTACACGTTTCCAC
2845	GAB1	TCTACACTCGATGTCCCAGA
2846	GAB1	AGATGGTTCGTGTTACGCAG
2847	GAB1	CTACTTGGTAGCAGACAGCG
2848	GAB1	GATAGATCTAGTTCACTTGA
2849	GAB1	AGTGAACTAGATCTATCACT
2850	GABRQ	CCGTACCGCACCGTTCCATC
2851	GABRQ	TCTCATGCATCCGATAGTCC
2852	GABRQ	GTAGTTGCCCTCCGCGAGCC
2853	GABRQ	GTCCGCCTGAGACCGAATTT
2854	GABRQ	GACGACTTCGGGCACAGCAG
2855	GABRQ	AAATTGTGCAAATGAAGCTG
2856	GAD1	TCGTCCTAGCGTACGATACC
2857	GAD1	TTGGATATTATTGGCCTAGC
2858	GAD1	AGAAGTCAGTCTCTGTGCGC
2859	GAD1	TATCCAATCCAGTGGAGAGC
2860	GAD1	TCCATGGGCCACGCCGCACC
2861	GAD1	GTGCCATAGGCTTCTTGCAA
2862	GALC	TCAACCACCACTCGTATCCT
2863	GALC	GCCCTATCGTTCTCAGATAT
2864	GALC	AATCCCAATATTACACTCAT
2865	GALC	TGTACACACCTTAATATAAT
2866	GALC	CTGGGTAATTTACTAGAAGT
2867	GALC	ATATAATGCCAATTATATTA
2868	GALNTL6	CACTTAGCATGACGAATATC
2869	GALNTL6	ACCATGATGACTCAGCTTAC
2870	GALNTL6	TCTGTACAAGGATAAGCACC
2871	GALNTL6	GAGATGGGCAATTCTATTCA
2872	GALNTL6	AGAGACCAGAGACCAACGTT
2873	GALNTL6	AATAGAATTGCCCATCTCCC
2874	GALR2	GGAGACCAGCGCGTAAACGA
2875	GALR2	GCAGGCCCGCGCAGATCGTG
2876	GALR2	TGGGCAGCCCGAGACGTTCA
2877	GALR2	CAGCGGCACCATGAACGTCT
2878	GALR2	CCGCCTCGCTCATGTGCAAC
2879	GALR2	CTACTACCGCCAGTCGCAGC
2880	GAPDH	TTCCTCACCTGATGATCTTG
2881	GAPDH	TCTGCTGTAGGCTCATTTGC
2882	GAPDH	TTCCACTCACTCCTGGAAGA
2883	GAPDH	ACTCACCCCAGCCTTCTCCA
2884	GAPDH	CACTGGCGTCTTCACCACCA
2885	GAPDH	TTCCAATATGATTCCACCCA
2886	GATA3	GTACTGCGCCGCGTCCATGT
2887	GATA3	TACGTGCCCGAGTACAGCTC
2888	GATA3	AACCACGTCCCGCCCTACTA

2889	GATA3	TCCGGGGAGACGTCCTTCGG
2890	GATA3	CCCTCATTAAGCCCAAGCGA
2891	GATA3	AGAACTTACCAGCCTTCGCT
2892	GATAD1	CTGCACTCTTCTCGCAATAC
2893	GATAD1	GATCCCGCCTCCTATATCAT
2894	GATAD1	CGTGACTTGAAATACTCAGA
2895	GATAD1	GCTACATATGGACTCATGTT
2896	GATAD1	AACTGACTCAGGAGCTTTGA
2897	GATAD1	TGCAGAATCAATCTTCTACA
2898	GATAD2A	TCGGAGAGCACAATCACGTC
2899	GATAD2A	GAACTCGTTGTTGGCGGCGC
2900	GATAD2A	CCCTCGAGCCCGAGAGTGAA
2901	GATAD2A	CAACAACGAGTTCATCTACC
2902	GATAD2A	TTCTGAGTGCCCCGAACAAG
2903	GATAD2A	GGCACTCAGAACATTCCTGC
2904	GATAD2B	ACGATTGGAAGAAGCCCGAC
2905	GATAD2B	TCTTACCGGTTGATAATTGA
2906	GATAD2B	GGAGTTAGCCTTCCTCGCTC
2907	GATAD2B	GCCCCGCTGACCCTGTAGCT
2908	GATAD2B	TTGTCTAGTGAGCCAGAGCG
2909	GATAD2B	GATGCTGCATTCTGTACAAC
2910	GDPD3	AGACCGGCGCATGGTTCGTC
2911	GDPD3	CATCCCTGTTTAGGCCCGAC
2912	GDPD3	CCTCCAGACGAACCATGCGC
2913	GDPD3	TCAGCTGACAGTCGAGCTCC
2914	GDPD3	CTGGAGGTTTACTTCTCTCC
2915	GDPD3	CACAGGATCTGGAGAGCTGC
2916	GEMIN7	TGCGGAGGTAACGCTCCCGA
2917	GEMIN7	ATGGACGCAGAGCCCCCTTG
2918	GEMIN7	GCGTTACCTCCGCAGCCTGC
2919	GEMIN7	GGTGAGCTTCACGTTGCACG
2920	GEMIN7	GCTCACCTGATGACCCACCA
2921	GEMIN7	GTGCGTGTGGCCGCCCACTT
2922	GFOD2	GAGCTGCGGGTAGTAGCGCG
2923	GFOD2	AGTCATCGCTAGTGACGTGC
2924	GFOD2	CAGATATCCGTGAAGGCTCT
2925	GFOD2	TGGCAGCTCCGCCCGAGTTC
2926	GFOD2	GGCAGGGCGACCGCCGCACC
2927	GFOD2	CTGCAGGACGCCTCGTCGCC
2928	GFPT2	GATCTTCGAAACCCTCATCA
2929	GFPT2	ATCACAGGTGTGGCGATCGA
2930	GFPT2	CGAAGATCTCCTTCCTCGTC
2931	GFPT2	CAAAGAAAGACACATTCAGC
2932	GFPT2	ACATGAACTACAGAGTCCCC
2933	GFPT2	CTCTGTACTCCAGCCGCTGC
2934	GGACT	GTACCCGCAGCACCGTGCGC
2935	GGACT	TCCGCAGCCTTTCGGGCGCG
2936	GGACT	ATGGCCCTAGTCTTCGTGTA
2937	GGACT	GGGTTGTAGCGCAGCCCGTG

2938	GGACT	CGAGCGGATGCTGCGCTTTC
2939	GGACT	ACGGGCTGCGCTACAACCCC
2940	GIMAP1	GGGCGCCCATTACTCCAACG
2941	GIMAP1	GTTGGGTCGGTTCACCGCCC
2942	GIMAP1	TCCAGGTAAGCATGGGAGGA
2943	GIMAP1	ACAGATGAAGAAAATGTCTA
2944	GIMAP1	GGGCGGTGAACCGACCCAAC
2945	GIMAP1	TGTTGCTCACGTAATCGTGC
2946	GIMAP4	CTTGTCGTAGTTGACACACC
2947	GIMAP4	GCGACAATGGCAGCCCAATA
2948	GIMAP4	GCCCAATGCTGAAACGTCCA
2949	GIMAP4	TGAAGCTCATACTGCCGTAT
2950	GIMAP4	AATGCAGCGAATAATCTCCT
2951	GIMAP4	TGTTGTTTAACGCACAGTAG
2952	GIMAP6	GCCATCGTCTTATCCGCCCC
2953	GIMAP6	CCCTGGGGCGGATAAGACGA
2954	GIMAP6	ATCCTGGGACAGCTCTTCTG
2955	GIMAP6	AGAATATGAACAAATTCCCC
2956	GIMAP6	CAGATAGCGTCTGCCACCTC
2957	GIMAP6	GTCCCCCCAGGTCTCGCCAG
2958	GIMAP8	CCAAAGACTCAACCTTGCGA
2959	GIMAP8	CCTTCGCAAGGTTGAGTCTT
2960	GIMAP8	GTCCGGGTATCCCCATACAC
2961	GIMAP8	ACGCGATTATGCTGTTCACC
2962	GIMAP8	AGTTTCGATCATTGATGCTC
2963	GIMAP8	CACAGGACCCGAGCAGAATC
2964	GIPR	CCAGCAGACGTACATATCGA
2965	GIPR	CCTTCGATATGTACGTCTGC
2966	GIPR	CACACCTGAAGGCGGTTCCG
2967	GIPR	GCAGCCGCGGAACCGCCTTC
2968	GIPR	GGTTTCGTCCTCCGCCAGTG
2969	GIPR	ACCGCTCCAAGATGAGCCTT
2970	GJB6	ACGCTGCACACTTTCATCGG
2971	GJB6	TTTCCGAGTCATGATCCTCG
2972	GJB6	GATTTGCATGCTGCTTAACG
2973	GJB6	GATCCACACCTTCCCGATGC
2974	GJB6	ACGAAACCACTCGCAAGTTC
2975	GJB6	CGTCTGCAACACACTGCAAC
2976	GLI4	GCGGCCGCTCGCACTTCTTC
2977	GLI4	CAAGCGCTTCCGCGGCTGGT
2978	GLI4	GCTCTCCCAGCCGTCCGACC
2979	GLI4	GAGTACCATGTTGATGCCCA
2980	GLI4	TGCTCACTCACCGGGCCAGA
2981	GLI4	CCTGGTGATGAGAGACTGAC
2982	GLUD2	ACGCTCCATTGTGTATGCCA
2983	GLUD2	CCGGGACGGCAGCAGCGCTT
2984	GLUD2	TGCCGCGATGTACCGCTACC
2985	GLUD2	CAACCACTCGGCCGCGTTGC
2986	GLUD2	CGGCCACCAACTCGCTGTAG

2987	GLUD2	ATGCACCCGATATACTGTCT
2988	GMFB	GGATATCGAGGTTGTCGTTC
2989	GMFB	TGATGTTGCCGAAGATTTAG
2990	GMFB	GCTCCTCATCCAGTACCACC
2991	GMFB	ACGCCTGGTGGTACTGGATG
2992	GMFB	AGTTCATCTTTAAGTTCATC
2993	GML	ATACTTACACTGAGCGCGCA
2994	GML	ATACAGCGCCTAATATGATA
2995	GML	GTCAGCAATATATTGCCATG
2996	GML	CATTTATGACCGCACAGTCA
2997	GML	ATGTCCCTTTCAAGATTAGT
2998	GML	CTTTGCCTTACTCCTAGCCA
2999	GMPR2	GGTGCCCACAGTATCCATAT
3000	GMPR2	AGCACTATAGCCTCGTTCAG
3001	GMPR2	AGTGTTACCTCAAGACAGTC
3002	GMPR2	CCCTACCTTACAGAGAACCT
3003	GMPR2	GATGCTGCTCATGGCCTCAA
3004	GMPR2	TAGGCTCTGTGTGTACTACT
3005	GNA11	CGTTGACCGCATCGCCACCT
3006	GNA11	ACTGACGTACTGATGCTCGA
3007	GNA11	GAAAGGGTACTCGATGATGC
3008	GNA11	TGTTCTGCTCGTACTTGTAG
3009	GNA11	AGGCCAATGCGCTCCTGATC
3010	GNA11	CCCCCAGGCACGGGCGAGAG
3011	GNG12	TCAGGTTTCGAAGGCATCAG
3012	GNG12	AAGCACCAACAATATAGCCC
3013	GNG12	CCTGGCATGTTCCTCACAGT
3014	GNG12	AGCCTCCATTGAAAGAATAA
3015	GNG12	GTTGGTATTCCTATCAGCAA
3016	GNG12	AACTTCAGAAAACCCTTTCA
3017	GOLGA7	ACTCGCAGTCCTCGCTCAAT
3018	GOLGA7	CTGACAGACCCTATTGAGCG
3019	GOLGA7	ACCCGGTTCTCCAGCTCCGC
3020	GOLGA7	AACAACCTTCGAGATATGAC
3021	GOLGA7	CGGCGCCTGCTGCGGCCTCA
3022	GOLGA7	ATATACCATCTTCCTATGCA
3023	GOT1	ACATTCGGTCCTATCGCTAC
3024	GOT1	AGAAGATCGTGCGGATTACT
3025	GOT1	TTCTTAGCGCGTTGGTACAA
3026	GOT1	GGAACAGGTGCACTTCGAAT
3027	GOT1	CTCTGCATCCCAGTAGCGAT
3028	GOT1	CCATTCTCCAGCATATCGCA
3029	GPC4	TTTGCGAGGGACATCTCCGA
3030	GPC4	GCAGCCCGTACTTTCGCTCA
3031	GPC4	TCATCGTTGCAAACGTTGCT
3032	GPC4	GCAACGTTTGCAACGATGAG
3033	GPC4	TCACGTTACAAGAAGTTTGA
3034	GPC4	TCATTTGAAGATCTGTCCCC
3035	GPC6	GTGGGAAGCCCCCGACAGTA

3036	GPC6	CTACTGTCTCAACGTCATGA
3037	GPC6	ACCGTCAAATTTCTTATGCC
3038	GPC6	TCAGCTGAAACATCCGTTCC
3039	GPC6	ACTGACCAGCTCAAGCCATT
3040	GPC6	ACTCGAATTTGAAAACCTTG
3041	GPI	CTCTTGCGCCGCCGCTTTGC
3042	GPI	CAGCAAAGCGGCGGCGCAAG
3043	GPI	CCGGTCAAACACACCCATCC
3044	GPI	TCAGCTCGGAGCGGTGCTCG
3045	GPI	CCGCCTCTTCGATGCCAACA
3046	GPI	TCACATCCTTGCCGTCTACC
3047	GPN3	AGACATCCGGGAACTGATCG
3048	GPN3	CAGCACATTTGCAAATCGCG
3049	GPN3	ACACTTCAACTACTCCGTGA
3050	GPN3	CATCCTTGCGCCACGTGCTT
3051	GPN3	GCAAAATACCAATCCTCCGT
3052	GPN3	GGTTTACAACTTGGACAGAC
3053	GPR173	ACAAGATGGCGTTACCCGCC
3054	GPR173	TGCGGTGACGATACTCGAAG
3055	GPR173	CACCTACAAGTTTATTCGGG
3056	GPR173	CTCCTCCCGAATAAACTTGT
3057	GPR173	GCCAAAGCCGGCGATCCAGT
3058	GPR173	GACGGCAGAGCGTATGCCAT
3059	GPR50	GGTGCGAGGATCGTACTCGA
3060	GPR50	CACGGTCTTGTTCACGAGCT
3061	GPR50	GCCGATTAGGTCTACAACGA
3062	GPR50	CATCTTTATGTTCTGCGCGA
3063	GPR50	CACTGAAGATCCGTTCGTAC
3064	GPR50	AATTGCGAACCTCAGCAAGT
3065	GPR68	TTCCAGACCCCTAACTCGCC
3066	GPR68	CCGCCCGACCCTGGCGAGTT
3067	GPR68	GGTGGTCTATGTTACCGTGC
3068	GPR68	GTCGGCGACGCAGTTGAAGC
3069	GPR68	GTCGATGGTACAGCTCATCG
3070	GPR68	GCATCCTGCGCGCCGTGCGC
3071	GPR78	GGCGCTGCTATCCAACGCAC
3072	GPR78	CGCCTGCGACCGCGCTATGC
3073	GPR78	CACCGCGCCACCAGGAAGAT
3074	GPR78	TCGTCACCGTGAACGCCCAG
3075	GPR78	TCACGTACTCTCTGCTCCGC
3076	GPR78	ACCCACCTGGTCATGACATA
3077	GPRC5A	ACAAAGTCTTCATTGCGACG
3078	GPRC5A	TCAGTCTGACCAAGCTCGTC
3079	GPRC5A	CTCGGCCAAGCGTGGGCCCG
3080	GPRC5A	CCATCCCACGGGCCCACGCT
3081	GPRC5A	TACCTGCAGCTGAAAATGTG
3082	GPRC5A	CAACTCGTGAAGAAGAGCTA
3083	GPX4	CACGCCCGATACGCTGAGTG
3084	GPX4	CGTGTGCATCGTCACCAACG

3085	GPX4	AGAGATCAAAGAGTTCGCCG
3086	GPX4	GCGCCCACCGGTACTTGTCC
3087	GPX4	GCGCACAGCGCCAGTCGTCC
3088	GPX4	GAATTTGACGTTGTAGCCCG
3089	GRB10	CTGTGCACATCCTCGCTGTC
3090	GRB10	GTGGAGCACCACCCGCACCT
3091	GRB10	CGTGTCTGACTGCATGCTGC
3092	GRB10	GAGCCAGGCCGCCGCAAAGC
3093	GRB10	TGGCTCGGAGGTAAAGAACC
3094	GRB10	TCCCTACCTAATCCTAGGTG
3095	GRB2	GCAGCAGCCGACATACGTCC
3096	GRB2	ATTATGTCACCCCCGTGAAC
3097	GRB2	GCACCTGTTCTATGTCCCGC
3098	GRB2	GAGCCGGGAAGTACTTCCTC
3099	GRB2	ACGAAGAATGTGATCAGAAC
3100	GRB2	CAGGAGCGCTCTCACTCTCT
3101	GRIN2D	GCACGCGCAACCCCGACAGC
3102	GRIN2D	ACCTATGCAGACTCTCGCCG
3103	GRIN2D	GTTCTCGGGGCCCGCGTACG
3104	GRIN2D	GGTACTTCATGAACATCACG
3105	GRIN2D	GGTTCTGGGCGCGACAGTCG
3106	GRIN2D	CCGGGATTACTCCTTCAATG
3107	GRWD1	CGCCGTCCGTAGGTGTCCAG
3108	GRWD1	CGTGGACCAGCGGCCATTCG
3109	GRWD1	CTCGGTTGATGCCACCATAG
3110	GRWD1	ATGGTGGCATCAACCGAGTT
3111	GRWD1	CTTACACTTTACTTGTGTGC
3112	GRWD1	CTCGCCCATGTGTCCAGCGA
3113	GSN	TGCAGTATGACCTCCACTAC
3114	GSN	GGAACACCCCGAGTTCCTCA
3115	GSN	ACCCTCCCAGGTATTGCCTA
3116	GSN	TCCTGTACCCACCCACAGGT
3117	GSN	GCAGGGTGCCCAGTCTACCC
3118	GSN	CCTCATCCAGCTGAGCAGTC
3119	GTF2A2	ACAGATTCTGCGATAATGTG
3120	GTF2A2	GAGCCTAGATGAGCTCATAC
3121	GTF2A2	CCACCTGTATGAGCTCATCT
3122	GTF2A2	TTCTATTCATTCTGTAGTAT
3123	GTF2A2	TAAATGCAGCACTGGCTCAG
3124	GTF2A2	CAAACTGAAGTAGAACTTGA
3125	GTF2B	ACAAAAGATCCATCTCGAGT
3126	GTF2B	TGCTCATTGTTCTCCGATTC
3127	GTF2B	GATATGATCTGTCCTGAATG
3128	GTF2B	TCCTCCACTAAAATCGCATC
3129	GTF2B	TGAGAATCTCCAACTCGAGA
3130	GTF2B	GAACAATGAGCAGTTCTGAT
3131	GTF2F1	CTCACTGCCCGCGCCCGATT
3132	GTF2F1	TGCCTGATTTGCCGTTGACC
3133	GTF2F1	CGACGTATTCAGTGACATTC

3134	GTF2F1	ACAAAGTCAACTTTGCTACG
3135	GTF2F1	CGTAGCAAAGTTGACTTTGT
3136	GTF2F1	GTGAGTTCAACCGCAAGCTT
3137	GTF2H1	GACCGTTACAGCCATCAGTT
3138	GTF2H1	TTGCTTCTTACCTCATCTAA
3139	GTF2H1	CCCCAAACTGATGGCTGTAA
3140	GTF2H1	CAGCAAAACTTCTTCAGATG
3141	GTF2H1	ACTACTAGATTTAACAGCTT
3142	GTF2H1	GTAAATCTATCTTTGCCTTC
3143	GTF2H3	TTACTCGCACGTAGTACAAA
3144	GTF2H3	CAAATGCATAGATGCCGTGA
3145	GTF2H3	CGAATTTCCCAGCACCATCA
3146	GTF2H3	TTCAGCCGATTCTTATATCC
3147	GTF2H3	TTAAATTCAGGAGGGTTGCC
3148	GTF2H3	GCAAGCATTAAAGGAATCTC
3149	GTF2H5	AACATGGTCAACGTCTTGAA
3150	GTF2H5	TTAATTCACCCACTCGCTCC
3151	GTF2H5	GAAGCAGTTTCTGCTGTACT
3152	GTF2H5	GTACAGCAGAAACTGCTTCA
3153	GTF2H5	GAATGATGAACTTCTTCCCC
3154	GTF2H5	TAATGTCCTCCAGGAGCGAG
3155	GTF3C5	CGAGCACCACTCGTCCCCGA
3156	GTF3C5	AATTCTCTGCCCCGTCATTG
3157	GTF3C5	GGTGTCCCTGAGCTCGTCGC
3158	GTF3C5	TCTACGCAGACCCCACCAAG
3159	GTF3C5	TGCTTTGACCGGCAAGTCAC
3160	GTF3C5	GGAGCTGTACTTCCGGCCCA
3161	GTPBP8	AGACCTATCTAAAAGAACGA
3162	GTPBP8	TTTACAGTGGTGGACATGCC
3163	GTPBP8	TACCTTACAGGAAACAACTG
3164	GTPBP8	AGAATTTGCATTACCTTATG
3165	GTPBP8	TTCCTCAGTTGTTTCCTGTA
3166	GTPBP8	GAGACTCTGACTTCAACCTC
3167	GTSE1	GTATGAATCTTTCCACGCCC
3168	GTSE1	GGGCGTGGAAAGATTCATAC
3169	GTSE1	GCTGTCAATGTGCCGGCCGC
3170	GTSE1	AGATTCCAGCTAGTCCTTCC
3171	GTSE1	CCATGTTCACGTCCCCTGCC
3172	GTSE1	GCGATGAGCCTTCAGCCTGC
3173	GUK1	GGCATGCTCGATGAAGTCGC
3174	GUK1	CATCGAGCATGCCGAGTTCT
3175	GUK1	TAGATACCACGAGGAACCCG
3176	GUK1	GCCGCCTTCACTGCACGTGC
3177	GUK1	ACATCAAGGCCACCGATCTG
3178	GUK1	ACCTTTGCCGTTCTCCTCGC
3179	HABP2	AGCGTGGGTAAGTGTGCTAC
3180	HABP2	TCCTCAGTGTAGTACCAGTC
3181	HABP2	GCAGTCCCCACCGTGTTCAC
3182	HABP2	TGACACTTATTCCCAGAGAA

3183	HABP2	GTTCTCCCTGATGTCTTTAT
3184	HABP2	CCAGTATGATTACAGCTACG
3185	HADHA	AAAGTCCACAAAGCCTATTG
3186	HADHA	CCATTGATGGCAGCCACAAT
3187	HADHA	AATTAACTCTCCCAATTCAA
3188	HADHA	AAATGAGATACCTTTGAATT
3189	HADHA	CAGAAGACCCTGTAAAATTG
3190	HADHA	ATACCTCAAGTCCTCCTCCC
3191	HBP1	CTAGTGGCTATGATGTGTAC
3192	HBP1	CACACGACTGTGCTTTCATA
3193	HBP1	CAAAGTGATCCTACCCAATC
3194	HBP1	CTTACCCTTTCGTGTCTTAC
3195	HBP1	CCTGTCAGATGATGATGATT
3196	HBP1	ATTCCTGGCCTTCAACTGTC
3197	HCAR2	ACTATGTGAGGCGTTGGGAC
3198	HCAR2	GCCTCACATAGTTGTCCATC
3199	HCAR2	ACAAATTTGCACCGCCATTC
3200	HCAR2	CATGTTGGCTATGAACCGCC
3201	HCAR2	AAGAAGATGCCGATCCAGAA
3202	HCAR2	TTGTGCGGATCCGCATCTTC
3203	HCFC1	GGGTCTGGCCAATGATAGCG
3204	HCFC1	CCCGCCGTAGATCACCAGCT
3205	HCFC1	GAGAAGCTGTGCCCGAGTCG
3206	HCFC1	CCGGGAGTCACATACTGCCG
3207	HCFC1	AGCCACTTACTTATTTCCGA
3208	HCFC1	GTTGCCGAGTGGAGACTGCG
3209	HCFC2	GGCAACTAGCTACGAATCAG
3210	HCFC2	ACACCGAGCGGTGGCCATCC
3211	HCFC2	GAGAAGCTATGTCCAAGCCG
3212	HCFC2	AATCTTCGCTTTCGTTTGCC
3213	HCFC2	GGGATATCTCCTCTAACAGC
3214	HCFC2	TGAGCTGCACGTCTACAACA
3215	HCLS1	AAGACGACGCCCATAGAAGC
3216	HCLS1	CGCTGTCGGCTTCAATGAAA
3217	HCLS1	ATGGGGAGCCAAGACCATCG
3218	HCLS1	TGTGCGTCCAGACCCCTCGA
3219	HCLS1	GGGCCATGAGTATGTTGCCG
3220	HCLS1	GTGGCTGTATATGATTACCA
3221	HDAC1	ACACCATTCGTAACGTTGCC
3222	HDAC1	TCACTCGAGATGCGCTTGTC
3223	HDAC1	AGAATGCTGCCGCACGCACC
3224	HDAC1	CTGGATACGGAGATCCCTAA
3225	HDAC1	TTACGTCAATGATATCGTCT
3226	HDAC1	GCACCATGCAAAGAAGTCCG
3227	HDAC2	TCCGTAATGTTGCTCGATGT
3228	HDAC2	TCCAACATCGAGCAACATTA
3229	HDAC2	TACAACAGATCGTGTAATGA
3230	HDAC2	TTCTGGTTTGTCATGTTTGA
3231	HDAC2	AAATATGGGGAATACTTTCC

3232	HDAC2	CCAGAACACTCCAGAATATA
3233	HDAC4	ACTTACCCGTACCAGTAGCG
3234	HDAC4	TTTCCAGCGACCCTCGCTAC
3235	HDAC4	GGTGCCTCTCCAGCTTCCGC
3236	HDAC4	CGGCCTTACCTGGATGGCTT
3237	HDAC4	ATGAGCTCCCAAAGCCATCC
3238	HDAC4	GCTGCAGCAGCTCAAGAACA
3239	HDAC5	GAAGTCGTCTCGACTGTCGT
3240	HDAC5	CCAGCTCTTACCCGACTCGT
3241	HDAC5	CATGAACTCTCCCAACGAGT
3242	HDAC5	CCCGAAGCTGTCGACACAGC
3243	HDAC5	TCCTGCTGTGTCGACAGCTT
3244	HDAC5	GGGAGCCCACCATGCTTCTT
3245	HDHD2	GGCTCAAGATGTCGGCATGC
3246	HDHD2	CCGATCATCAACTAGCAGCA
3247	HDHD2	TTTCCGCTCTAGTAAACTTC
3248	HDHD2	AATCCCATGACCACAGCATT
3249	HDHD2	AAACTCTCACAAGTTAAGTA
3250	HDHD2	AACAAGTGATCCTAATGCTG
3251	HELLS	TTGGATAGGCTCGCATGTCT
3252	HELLS	TCCAGTTGTTCAACCATTGC
3253	HELLS	ATAGAGAGTCGACAGAAATT
3254	HELLS	GGACACTGCTGTGATTACCC
3255	HELLS	GGAGTCTTTAAAAGTTAAAA
3256	HELLS	ACAGAAGAAGAAAGAAAAAT
3257	HESX1	TCTTCTCTAATATCGATACC
3258	HESX1	GATTTCATTCCCTAGCGTGG
3259	HESX1	TGGCATACCTGATGAGCTGC
3260	HESX1	GGCCTGTGGGGTTTCATTAA
3261	HESX1	TATTTCGAAGCTCTTTCTTC
3262	HESX1	TTTAGAGTAAACTGCTATCC
3263	HGF	TCGATAACTCTCCCCATTGC
3264	HGF	CAGGCAAGATTTGTCAGCGC
3265	HGF	TGTTCTTACCTGTGTTCGTG
3266	HGF	CCCTTCAATAGCATGTCAAG
3267	HGF	ACAGTATCTATCACTAAGAG
3268	HGF	TCCACTTGACATGCTATTGA
3269	HHAT	CTTCTCGGAGTTCATCAAAC
3270	HHAT	GGTGTAGTACAGGCAGCGAA
3271	HHAT	AGGATGCGACCGACTTTGAG
3272	HHAT	CCATTTGAGACACTACCATG
3273	HHAT	TCCACTTTAGATGCAGCAGC
3274	HHAT	GGCTGGGCCGCCTTCTTTGC
3275	HHLA2	ATCCTCGTCCAATTATCACG
3276	HHLA2	ACAGGGCGCTGGACGATGAA
3277	HHLA2	TCAATGAATCCCGATTCTCA
3278	HHLA2	ACTTACCTACATGCACTGTG
3279	HHLA2	TCAGAGATGTTATGAGAATG
3280	HHLA2	ATTCTCATAACATCTCTGAG

3281	HIBADH	GGTGTAGGAGCTGCACGATC
3282	HIBADH	AGCTCCACAGTACACCACGT
3283	HIBADH	GCAGCCGCCGGCTCCAGTAC
3284	HIBADH	GGCAGCTCCGAGGAGCCGTA
3285	HIBADH	AACTGCAGGATCAATAGTGC
3286	HIBADH	TACCACTTACCACCAGAAAC
3287	HIF1A	TTCTTTACTTCGCCGAGATC
3288	HIF1A	CCTCACACGCAAATAGCTGA
3289	HIF1A	TGTTTACAGTTTGAACTAAC
3290	HIF1A	TACTCATCCATGTGACCATG
3291	HIF1A	TTTAAATGAGCTCCCAATGT
3292	HIF1A	GTTATGGTTCTCACAGATGA
3293	HIST1H2AM	TGAGAGCCACCACAAAGCTA
3294	HIST1H2AM	AAAACCCGCTCCTCTAGAGC
3295	HIST1H2AM	AGGCGGTGCACTCGTCCTAC
3296	HIST1H2AM	AAATTGGAGCCCAGCTCTAG
3297	HIST1H2AM	CAGAACACCGCCCTGAGCGA
3298	HIST1H2AM	CTTACTTGCCCTTAGCTTTG
3299	HIST1H2BF	ACGTTAGAATTACTTAGAGC
3300	HIST1H2BF	GTCCGCTCCTGCTCCAAAAA
3301	HIST1H2BF	GGAGCGGACTTAGCAGGTTC
3302	HIST1H2BF	CTCCAAAAAGGCGGTGACCA
3303	HIST1H2BF	GGCTAAGCACGCCGTGTCAG
3304	HIST1H2BF	TAGCACCTTGTACACGTACA
3305	HJURP	GCAGTACAACCAGCCCTTCG
3306	HJURP	CTTACCCTGTGGCGTCTCGT
3307	HJURP	GGTCGATGCCACGTCAGACC
3308	HJURP	TCCCTCGCACCGCACAGTCC
3309	HJURP	GGACTCCTCCATGAAGCCCG
3310	HJURP	CCCAAGCAACTGACTCTTCC
3311	HK1	CAGAGCTTACCGATTCTCGC
3312	HK1	AGATGTTGCCAACATTCGTA
3313	HK1	GCAGATCTGCCAGCGAGAAT
3314	HK1	TTGGATTAAAATCCCGGGAG
3315	HK1	TCAGCAAGTAGATGAAGTTT
3316	HK1	TCATCTACTTGCTGAAAGTG
3317	HK2	TGACCACATTGCCGAATGCC
3318	HK2	TTACCTCGTCTAGTTTAGTC
3319	HK2	GTTGAGCTCCGTGAAGAAGT
3320	HK2	GCTGACTTACCTTCTGCACT
3321	HK2	GATGACCACAACTGTGAGAT
3322	HK2	ATCATAACCACAGGTCATCA
3323	HK3	CCGGATGCTGCCTACATACG
3324	HK3	TCCAGAGCAAGGAGACTTCG
3325	HK3	TGATACCCACCAGTGCCATG
3326	HK3	GATCCCCCAAGAGGTGATGC
3327	HK3	GTCTTCAGGGTTGCGGCAGG
3328	HK3	TGGAAGCTCATGGACTCCAT
3329	HLA-E	CGTTGTCGAAGCGCACGAAC

3330	HLA-E	ACCGGGAGACACGGAGCGCC
3331	HLA-E	GGATTATCTCACCCTGAATG
3332	HLA-E	ACTCCACGCATGTGTCTTCC
3333	HLA-E	GGATGGGGAGGGCCATACCC
3334	HLA-E	CCGAGCCCGTCACCCTGAGA
3335	HMBS	ACATTGAAAGCCTCGTACCC
3336	HMBS	AAAGATGAGAGTGATTCGCG
3337	HMBS	GCAGCTTGCTCGCATACAGA
3338	HMBS	ATCAAGAATCTTGTCCCCTG
3339	HMBS	TCAGTTGCTATGTCCACCAC
3340	HMBS	GTTCAAGCTCCTTGGTAAAC
3341	HMCN1	TGCTACACAAGTATATCGTC
3342	HMCN1	CATGTTAATATTGCTGCGAA
3343	HMCN1	TGCATACGCTTCACATTCCA
3344	HMCN1	AAGGTCCCCTTTATTGATTA
3345	HMCN1	ATAGGAAACATGCTAGATAC
3346	HMCN1	CTCCCCCTCCTACACTGACA
3347	HMGA1	GGGCATCTCGCAGGAGTCCT
3348	HMGA1	TAGAAAACCACCACAACTCC
3349	HMGA1	GGGTCACTGCTCCTCCTCCG
3350	HMGA1	CTCACCGGAGGCTGCTTGCG
3351	HMGA1	CTCGAAGTCCAGCCAGCCCT
3352	HMGA1	TGCCAACACCTAAGAGACCT
3353	HMGB2	CTGCACGAAGAAGGCGTACG
3354	HMGB2	GCGAAATTGACGGAAGAGTC
3355	HMGB2	GTGACAAAGCTCGCTATGAC
3356	HMGB2	GAAGTCGAAGTTTGAAGATA
3357	HMGB2	TATTGCTGCATATCGTGCCA
3358	HMGB2	AAGATCAAAAGTGAACACCC
3359	HMGB3	GTTCTTTGCAGACGATGTCC
3360	HMGB3	AGTCACTTACGGTGGCCTTT
3361	HMGB3	TAAGCGGACATCTTGCCCTT
3362	HMGB3	AAAATTCCGCAAAATTGACA
3363	HMGB3	TTTGCCGCCTTAGTGATGTA
3364	HMGB3	AAGATCAAATCCACAAACCC
3365	HMGCL	AGCCCTCTAGCATGTAGACC
3366	HMGCL	TCCTGATGCCCCCTGTGCGT
3367	HMGCL	CTCACCTGGGGAACCCACTT
3368	HMGCL	GCCTCGAAGCCTTTCAAATT
3369	HMGCL	GTCCTGACCCCAAATTTGAA
3370	HMGCL	ACCAAAGCGGGTGAAAATTG
3371	HMSD	TATGTGCTTAGAACTGCCAA
3372	HMSD	CCACAACACGTGTAAACTCC
3373	HMSD	GAAGATGGAGATATTCATCG
3374	HMSD	CAAGGCTGATGATATGCTCA
3375	HMSD	CATGAGCATATCATCAGCCT
3376	HMSD	CTTTGCCCCCATGAAAACCA
3377	HNF1A	GTACGTCCGCAAGCAGCGAG
3378	HNF1A	GGCTTCTTCTTTGCGCCGGT

3379	HNF1A	AGACACGCACCTCCGTGACG
3380	HNF1A	ACGGTTCCTCCGCCCCTTCT
3381	HNF1A	GGCGGAGGAACCGTTTCAAG
3382	HNF1A	TATCGACCACCTCCCGCTGT
3383	HNF1B	TGTACAGAGCGGCACGCTTC
3384	HNF1B	ACAAGAAGATGCGCCGCAAC
3385	HNF1B	GTTGCGGCGCATCTTCTTGT
3386	HNF1B	AGACTTGCTGTAAAACCGAC
3387	HNF1B	TGCTTACCTGACAGCTTGTT
3388	HNF1B	TCGTTGCTTTCTGACGTACC
3389	HNRNPC	ATACATACACTTTCCAGACT
3390	HNRNPC	GCTCCTTCTTAATGGCCTGA
3391	HNRNPC	AGTGAAAGGAGATGACCTTC
3392	HNRNPC	ACTTCACGAAGGGGCAAAAG
3393	HNRNPC	GCAGAGCCAAAAGTGAACCG
3394	HNRNPC	AGGTGTGAAACGATCTGCAG
3395	HORMAD1	ACACTTACCTGAGGATCTTC
3396	HORMAD1	CCATGGGAGTCCTCTGCAAC
3397	HORMAD1	CACAAACCCAGAAGATCCTC
3398	HORMAD1	TGGCCACTGCCCAGTTGCAG
3399	HOXA2	TGTCGAGTGTGAAAGCGTCG
3400	HOXA2	AGTCACCCTCGCCACGGCGC
3401	HOXA2	GAATCCCTGGAAATCGCCGA
3402	HOXA2	GTCGAGGGAACCTGGCAAAC
3403	HOXA2	TGCAGCCGCCCGAGTACCCC
3404	HOXA2	TACGAATTTGAGCGAGAGAT
3405	HOXB9	GCTGCCGCGAGCTCGCGTAC
3406	HOXB9	GTACGCGAGCTCGCGGCAGC
3407	HOXB9	GTCTGGCCACTTCGTGCCTA
3408	HOXB9	CAGCCAACCCCTCCGCCAAC
3409	HOXB9	GGGCACGCCCGAGTACAGTT
3410	HOXB9	CCGCTGAGCCCGCACGCGTC
3411	HOXD8	CCTAGCCCTCACCGAGAGAC
3412	HOXD8	CAAATTTCCCGTTTCCCGGC
3413	HOXD8	CAACCTTGTGGTCTCATCCA
3414	HOXD8	CTCCTTCTCAAATGTTTCCG
3415	HOXD8	TCGCTTCCAAACTCTAGAGT
3416	HOXD8	GCAGCTCCTGGTAGACGAAG
3417	HP1BP3	GAATTCACAGTTCGACGAAT
3418	HP1BP3	GCCTAAGAAAGCACCTCCTA
3419	HP1BP3	GGTCTTCTTGGCAGGCGTTT
3420	HP1BP3	GTCGAACTGTGAATTCTACC
3421	HP1BP3	AAGAGCCGCCACCTAAGAGA
3422	HP1BP3	TGTCCACACCTTCTCTTAGG
3423	HPRT1	GTTATGGCGACCCGCAGCCC
3424	HPRT1	CTGTCCATAATTAGTCCATG
3425	HPRT1	TCTTGCTCGAGATGTGATGA
3426	HPRT1	AATAAATCAAGGTCATAACC
3427	HPRT1	TAAATTCTTTGCTGACCTGC

3428	HPRT1	GAGCTGCTCACCACGACGCC
3429	HRAS	GGACTCGGATGACGTGCCCA
3430	HRAS	GGAGGATGCCTTCTACACGT
3431	HRAS	TGTAGGGGATGCCGTAGCTT
3432	HRAS	CTCACGCACCAACGTGTAGA
3433	HRAS	CATCCTGGATACCGCCGGCC
3434	HRAS	TTGACACAGGGCAGCCGCTC
3435	HS6ST3	TTCTAACGTGGAGATCAACG
3436	HS6ST3	CACCACTTTCGGCCGGCACC
3437	HS6ST3	AGTTTAGATCCTCAATGCGT
3438	HS6ST3	GCGGGGCTGCTCCCCGAAGT
3439	HS6ST3	TCACACAGTTCAACATCACG
3440	HS6ST3	TCGCTCAGGTAACGTGACAC
3441	HSD11B1	CAGTCAGGACCACGTAACTG
3442	HSD11B1	CATTGTTGTCGTCTCCTCTC
3443	HSD11B1	GCAGGTGGTATCCCACTGCC
3444	HSD11B1	GCCTCAGCACACTACATTGC
3445	HSD11B1	ACCTCGCTGTCACCACCACA
3446	HSD11B1	CTCTCTTCCGATCCCTTTGC
3447	HSD11B2	CCGCGTGCTAGAGTTCACCA
3448	HSD11B2	CCTGGCCGGGGTAATAGCGG
3449	HSD11B2	CCTTGGTGAACTCTAGCACG
3450	HSD11B2	TGGCCAGGCGTAGCGAGTGC
3451	HSD11B2	GGACATGCCATATCCGTGCT
3452	HSD11B2	TGTGTCCATGAGTAGCGCCA
3453	HSD17B4	ATCCGGTGTAGTGATTATTA
3454	HSD17B4	GCTTTGTCACGAGAGTTGTG
3455	HSD17B4	ATCCATAATAATCACTACAC
3456	HSD17B4	AACTCTCGTGACAAAGCCAA
3457	HSD17B4	AACTCACCTGCTCTGGGAAG
3458	HSD17B4	TATAAACCACTTCCCAGAGC
3459	HSPA12B	AGTGCGCTTGCGAGCCTCGA
3460	HSPA12B	CCAGGTCATGGTAGTAATCG
3461	HSPA12B	ACGTGGAGACCGCTCTGCGC
3462	HSPA12B	CCGCGATTACTACCATGACC
3463	HSPA12B	CACTGGGGACCGCTCCGGGC
3464	HSPA12B	GGGCAATGCCGCAGCTTTCC
3465	HUS1	CCAGCTCCACGGAGACCGTG
3466	HUS1	CATTAGCCAGCTTGTCACAA
3467	HUS1	GTTATTGAAGCAAACCTAGA
3468	HUS1	TAAGCTTATCAGGGCTGATG
3469	HUS1	GATTTATTTAGAGCTAACAT
3470	HUS1	ACAGGGTGCTAGGACAGCGC
3471	HYDIN	GATCATCGAACTCTTAGATA
3472	HYDIN	TCTAAGAGTTCGATGATCTG
3473	HYDIN	GCTGGAATAATGCCTGATCC
3474	HYDIN	CGGGCGGCGCATGGAGAGTG
3475	HYDIN	TCATTGTTCCTCAAAATCAG
3476	HYDIN	TACACCCTCAGAGTTCCTGA

3477	IDI1	TAACCACCTCGACAAGCAAC
3478	IDI1	AAGCAATCCAGCCGAGCTTG
3479	IDI1	GTTGAACCTGTTGCTTGTCG
3480	IDI1	GAGCTGGTTACCTCTTCCAA
3481	IDI1	CGTTATCTTAATTTCACCAC
3482	IDI1	CACTACAAAGCTCAGTCTGA
3483	IDUA	AGGTCGTCGCGCAGCAGATC
3484	IDUA	GGTCTACGTCACGCGCTACC
3485	IDUA	CACCCCCATTTACAACGACG
3486	IDUA	CAGGCGCCGCCACTCGCCGT
3487	IDUA	GTAGCTCAGGCCCCGTCCAG
3488	IDUA	GAAGGTCCAGGTACCCGTCC
3489	IFFO1	GGCGTGCGAGAACGACCAGA
3490	IFFO1	TTCTCGCACGCCCGCCGGCT
3491	IFFO1	AGACCGTGTAAATGAGCTCC
3492	IFFO1	CTGAGCTGGTGGTCTTCAAG
3493	IFFO1	CCTCCTGGAGCTCATTTACA
3494	IFFO1	CCAACTGCAGGAAGCCCAGG
3495	IFI30	ACTTCTTCAGGGGCCCCCGC
3496	IFI30	ACACACCTGTGCGTTTCCGT
3497	IFI30	TCATGCACGCCAACGCCCAG
3498	IFI30	CACCACACGAGTATGTGCCC
3499	IFI30	GTCAAGTTCATCCAACACGC
3500	IFI30	CAAACCCCAGGCCTGCGTGT
3501	IFNG	CTGTCGCCAGCAGCTAAAAC
3502	IFNG	CGAAAAGCTGACTAATTATT
3503	IFNG	GTTCATGTATTGCTTTGCGT
3504	IFNG	GACATTCATGTCTTCCTTGA
3505	IFNG	TGCAGGTCATTCAGATGTAG
3506	IFNG	TTCTCTTGGCTGTTACTGCC
3507	IFNLR1	CCACTTCGCGCCACCGTCTA
3508	IFNLR1	ACAAGTTCAAGGGACGCGTG
3509	IFNLR1	ACTTCAGCGTGTACCTGACA
3510	IFNLR1	CACTCAGGATCTCCTCCGTC
3511	IFNLR1	ACCTCATACTTCAGATCCAG
3512	IFNLR1	ACTGAGGCAGTGGTGTTCGC
3513	IGF1R	TCAGTACGCCGTTTACGTCA
3514	IGF1R	GTGGAGAACGACCATATCCG
3515	IGF1R	CCACGACGGCGAGTGCATGC
3516	IGF1R	TGCCGCCACTACTACTATGC
3517	IGF1R	TGTTTCCGAAATTTACCGCA
3518	IGF1R	GGCTCTCTCCCCGTTGTTCC
3519	IGFL2	CTGAGCGAGACCCGCCAATG
3520	IGFL2	TCACAACAAAATCGTTTGTG
3521	IGFL2	CGACTACCCCAGGAGTGTGC
3522	IGFL2	TTACTTACCCAGCACACTCC
3523	IGFL2	AGACAGACTGACACATAAGC
3524	IGFL2	TACTCACCGATGACTTCCCT
3525	IKZF5	GTAACTTGTCCCATCATCGA

3526	IKZF5	ATCTGCTTATGAGCGTCATC
3527	IKZF5	CAGAAATCATGTTCACGTGA
3528	IKZF5	AAATCTTTCACGAAGTCCAA
3529	IKZF5	AACTCAGGAATGTTAGTAGA
3530	IKZF5	TTGATGGGAAGCTTAAGTGT
3531	IL17RD	GTATTACCCGAAATCCTTTC
3532	IL17RD	GCAATCACATGCTTCCCCAC
3533	IL17RD	GTATGCTTGCCATGACCAAG
3534	IL17RD	GTGCCAACAACTGATTCTAA
3535	IL17RD	GTACAGCCCACTGTTTCTGC
3536	IL17RD	TTACTTACTGTCATATTTGA
3537	IL27RA	CAGTGAAGCCAAACGCCCCC
3538	IL27RA	CCTCACCAGAAGGCGGTGTC
3539	IL27RA	GTTCCACTACCGAAGATGTC
3540	IL27RA	CCTGATACCCACACATCTTT
3541	IL27RA	TCCTTCCAGACACCGCCTTC
3542	IL27RA	CTCACCTTCCATAGAAGCAA
3543	IL6	TCTCATTCTGCGCAGCTTTA
3544	IL6	GTACCTCATTGAATCCAGAT
3545	IL6	CACTACTCTCAAATCTGTTC
3546	IL6	TTTGTCAATTCGTTCTGAAG
3547	IL6	ACATTTGCCGAAGAGCCCTC
3548	IL6	GAACAGATTTGAGAGTAGTG
3549	IL6ST	AGATGCCTCAACTTGGAGCC
3550	IL6ST	TTTGAGTTGCATTGTGAACG
3551	IL6ST	ATTCGCTGTATGAAGGAAGA
3552	IL6ST	ATGATAGTATATTGCTCCTT
3553	IL6ST	GTGGATGCTGTGTCTTCAGG
3554	IL6ST	ATTATAACACTCTTAATACT
3555	IMPDH2	AACATCCCGCACGCGATCCT
3556	IMPDH2	AATCACTCTTAAGACCCCAC
3557	IMPDH2	GACTTGGTGGTAGCCCCTGC
3558	IMPDH2	GATACCGCAGAAACCATGCC
3559	IMPDH2	GGGGCTCACCGCCATTGCTA
3560	IMPDH2	CCACGTAGATAATGACAAAG
3561	INHBE	CTGCGACCCCCTTATGTTGC
3562	INHBE	GAGTTAAGGTATGCCAGCCC
3563	INHBE	ACCCCAAGCAGAACGAGCTC
3564	INHBE	GTGGGCACTGGTGCGAGCAC
3565	INHBE	GCACAGTTACTGGACAACCG
3566	INHBE	GTTGTGTCCCTACTGCCCGA
3567	INO80	GGATCCCTTAATACAAGTTA
3568	INO80	AATCCATTATTGCCCCAGTC
3569	INO80	GCTAAGTGATGAATCCAGCG
3570	INO80	ACTCTGAGAATCAGCCTCGC
3571	INO80	TACAGAATCAAAATGTGATA
3572	INO80	AGCTGAAGAAAAGCAGAAAG
3573	INO80B	GCGCTACTGCAGCGGAGCAC
3574	INO80B	AAGGTTGACTCCGCGCCTTC

3575	INO80B	GCGGCTGCTTACTGCTCGAC
3576	INO80B	CCCTTGAGCAGTACCGTGCC
3577	INO80B	TTGAGCCTGGCGGGTGCCCA
3578	INO80B	GCCCGGCGTCTTCTTCCTGA
3579	INO80C	TTAGGATCGTTCAGTTGCCA
3580	INO80C	TGCCAGCTACTGCGCCACCG
3581	INO80C	TCGATGCTTACCACAAAGTT
3582	INO80C	TGAGTTTAGCACAGGACCTG
3583	INO80C	CGCCTTCCACCATGATTGTG
3584	INO80C	AATGGACTCACAGTTCCACG
3585	INO80D	GAGCTGCATGCAGTCGAACC
3586	INO80D	GACGAGATTCTGCCCGCTCC
3587	INO80D	GAACACCAGCCTACCAATGC
3588	INO80D	CTTGAAGATGCCCAACGGAC
3589	INO80D	CCCGGATGGACCATGCAACC
3590	INO80D	ACGCATGTCTTTGATATCTG
3591	INO80E	ACTTCTGCAGTACGAGAACG
3592	INO80E	ACAGGAGCACGAGTGCTTCC
3593	INO80E	ACGTGCCTCACCTCTTGTCC
3594	INO80E	CGTTCTCGTACTGCAGAAGT
3595	INO80E	CAACTCACCGAGCTCAGGTA
3596	INO80E	GCTGTTATCTGATGATGCAG
3597	IPO5	TATGCAGCCTGTAATGCCGT
3598	IPO5	CAGGCTGCATACCTTACTCT
3599	IPO5	CAAATGCCTGAGGATCAAGT
3600	IPO5	ACCATCATTGCTAACATCTG
3601	IPO5	TCCTCAGATGTTAGCAATGA
3602	IPO5	AAGCAACAGAAAACACAATA
3603	IQGAP3	CCTTCGGAATGGAGTGCTGC
3604	IQGAP3	TTGCCTATCAGTACCTGTGC
3605	IQGAP3	CTTGAAGAAGATCTACGATG
3606	IQGAP3	AGATGAACGCCTCACAGCTG
3607	IQGAP3	CACTCACAGGCTGCCCAGCC
3608	IQGAP3	GAACATGGAGAGGAGAGCAG
3609	IQUB	GTACTGCAGATAAGATACTC
3610	IQUB	GCTCCTCAACATGTATTGCA
3611	IQUB	AATCCATCTATTCTTCTGAC
3612	IQUB	ATTACAATTTCCTGGCCCAC
3613	IQUB	AATGAGACTCTAGTACAACA
3614	IQUB	GAGCCATGCAATACATGTTG
3615	IRF6	CTGGGTTAATGATCGAGCCC
3616	IRF6	CTGAGGGTTGCTCACGGTCA
3617	IRF6	CCCACTCACCATTGATGTTC
3618	IRF6	CATGACCGTGAGCAACCCTC
3619	IRF6	AGAGAGCTGATCCACAGTTC
3620	IRF6	GAGCTATAGAAGGGCTGTAT
3621	ISX	CCTCTTTAGGATCGCCTCAA
3622	ISX	TGTGTTTCCCATGGGTGCGC
3623	ISX	TGTACCTGCACCCGAGCTTC

3624	ISX	TCCCACGAGCTGTTGTCCAT
3625	ISX	CGATCCTAAAGAGGCCTGCC
3626	ISX	CGTTCACATCCGCAGCCAGC
3627	ITGA1	GGCCAACCCAAAAACAGAAC
3628	ITGA1	GGTAGATCCAACTTTACACA
3629	ITGA1	CAGCGACCCCTGGGCGGGCG
3630	ITGA1	GGGTCGCTGTCGCCTGCTGC
3631	ITGA1	TATCCAAACATGTCTTCCAC
3632	ITGA1	AAAAATTCAATGACTTTCAG
3633	ITGAE	ACCGGGGAGTGACCGTTGTC
3634	ITGAE	TCTGGTGTTGATCGTGATTC
3635	ITGAE	ACGCTGCCTTTAATGATGAT
3636	ITGAE	CCATCGATGTTCCCTTGTCC
3637	ITGAE	CCTGGACAAGGGAACATCGA
3638	ITGAE	GGCCACATCCACATTGAAAG
3639	ITGB3BP	GCAAAATTTAAGTAGTATAC
3640	ITGB3BP	AGAGAGCTTGAAAATCTCAT
3641	ITGB3BP	TGTTAAAAGATCACTGAAGT
3642	ITGB3BP	AAAATTCTTACTTAGTTCTT
3643	ITGB3BP	GAAATTGTCAGAAGAAATCA
3644	ITIH1	CTGTCGATGGCGTGTTCATC
3645	ITIH1	AACATAGTGGGCGAAGCGAG
3646	ITIH1	GCCATATACCTCGCTGCTCT
3647	ITIH1	TTAGAGCATGGACGGTGCCA
3648	ITIH1	TTTGCAGAAGCGACAGGCTG
3649	ITIH1	ACCTGCCTGTAGCCGAGCCC
3650	ITIH6	AAGCTATTCTATGCGCTCCA
3651	ITIH6	CCGCTGAAGCAGTTCCTCAT
3652	ITIH6	CAAGACCAGTCGTCCATCTC
3653	ITIH6	TGCGCAGACGGCCGGTCCTC
3654	ITIH6	GGTCTCTCCCCTCTCGATCC
3655	ITIH6	GGCATAGCGAGACACCACCG
3656	ITPA	CTGATAAGTGCCGGAGTACC
3657	ITPA	AAATCTCATCCGGCTCCCCC
3658	ITPA	GAACAGGTCGTTCAGATTCT
3659	ITPA	AGTCACTCACATGTAGGGGC
3660	ITPA	ACAAGTGTCCTCAACCAGCA
3661	ITPA	CTCTTGCAGAAAGTGGTTTC
3662	IVD	CTATTAGCTTCGTCAGACCA
3663	IVD	CATTGCTGCGATCGATCTCC
3664	IVD	CATTCCCATTGCGTACAAGC
3665	IVD	CTGTGATGCCCAATACGCCC
3666	IVD	GAGGAGATATCCCGAGCTTC
3667	IVD	GCCATGAGTGAGCCCAATGC
3668	JAM3	CGTTGCTCGAAATGACCGCA
3669	JAM3	ATTCGCAGACAAGTGACCCC
3670	JAM3	CAACGACCTCACAGCGATAA
3671	JAM3	GCCTTCGGCACTCTACAGAC
3672	JAM3	ACCCCCGGCCTCACTACAGC

3673	JAM3	TGAGATTTACAGCCCCTATC
3674	JARID2	CTTCATGCCAGTCGACCCCC
3675	JARID2	TTCAGCTAACCACCCCGCAG
3676	JARID2	TCCTGAGCCGACCGATGCAG
3677	JARID2	AGAACTAACATCGTTGCTAG
3678	JARID2	TTCCTTTCCTGGGGGTCGAC
3679	JARID2	CCTTAGACTGGTCATTACCA
3680	JUN	TCGTTCCTCCCGTCCGAGAG
3681	JUN	GTCATGAACCACGTTAACAG
3682	JUN	CACGCTGCCCAGCGTCACGT
3683	JUN	TCTCCGCCTTGATCCGCTCC
3684	JUN	CAAGCTGGCGTCGCCCGAGC
3685	JUN	GATTATCAGGCGCTCCAGCT
3686	JUP	CATGGCCTCCCGCACCCGTT
3687	JUP	AGTTACGCATGATCTGCACG
3688	JUP	ACGCCTCCTTCTTCGACAGC
3689	JUP	TAGCACCTTGAGCACACGAC
3690	JUP	GCTGCTCGCCATCTTCAAGT
3691	JUP	GTAGTTGATGAGATGCACAA
3692	KAT2A	CCTGAGACCTCTGCCGAAAC
3693	KAT2A	CCTACAAGGTCAATTACACC
3694	KAT2A	TCTCAAATGGAGGGCTGCCC
3695	KAT2A	CCGCAGTTGTGAGCACCCCT
3696	KAT2A	TTTCCAGCCATTACACTTAC
3697	KAT2A	TATCTCACCTGCTCAATATT
3698	KAT2B	CCACATCCAATACTATTCCC
3699	KAT2B	GGCATGGCTACAACTCCGAC
3700	KAT2B	ACCTGTGGTTGAAGGCTCTT
3701	KAT2B	GAGGAGTCTTGTAAATGTAA
3702	KAT2B	TCCAAAGAGCCTTCAACCAC
3703	KAT2B	AGCTGCTCATGTTTCCCACC
3704	KAT6A	TGATAGCCAATCGTAACTGC
3705	KAT6A	TTCACTCGAACCGTTAGTTC
3706	KAT6A	TTCGAGTGAAGGCCTTACGG
3707	KAT6A	TTAGTGTTGAGCCGATAAAG
3708	KAT6A	CATACCACTGTTGCCACAGT
3709	KAT6A	CTCATCTCCTGTGCCGACTG
3710	KAT6B	TGAAAGACGGACCGCAGTAC
3711	KAT6B	GTTGTCTGGGTCCTTATAGG
3712	KAT6B	TTCGATGCACTGCCACCTTA
3713	KAT6B	TGCCACAATCTGCACAAGAG
3714	KAT6B	TCCAATATGTAGCTTCTGTT
3715	KAT6B	TGCAGTGCCTGTAGAGTCCA
3716	KBTBD6	CGTCCAGACTATCCAAGCGC
3717	KBTBD6	GATAGTCTCGAATTACCATT
3718	KBTBD6	CGCGTACAGGCGCTCCACGT
3719	KBTBD6	ATGCCGCGGCCAGCACATTG
3720	KBTBD6	TGGTACCTATATTAATCCAT
3721	KBTBD6	CCTGGGATCGCAGCTTGCGA

3722	KCNIP3	CCCGTTCCCGTCCGCATCAA
3723	KCNIP3	GAACGGGGCCATCCACTTTG
3724	KCNIP3	CGTCGGACGGCAGCCTCCTG
3725	KCNIP3	CTCTGGCTGGTGGCGCACCG
3726	KCNIP3	ATACCCTCCTTCTTGCTAAG
3727	KCNIP3	CACGGTGCGCCACCAGCCAG
3728	KCNJ2	TATCTCCGACTCTCGCCGTA
3729	KCNJ2	GGGCAAAGCTTGTGTGTCCG
3730	KCNJ2	GTGTCCGTACTAGTGCTTTC
3731	KCNJ2	AGACATCTTCACCACGTGTG
3732	KCNJ2	CAACGCAGACTTTGAAATCG
3733	KCNJ2	GCCGTGATTGCCATGAGAGA
3734	KCNMB3	CCCCGTGCAAATCACACTTC
3735	KCNMB3	AGCCTCTTGTGCACATCTAG
3736	KCNMB3	AGAGGACCGAGCCGTGATGC
3737	KCNMB3	CTTACCTCCCCTGGCGCCTC
3738	KCNMB3	GGGGAAGTACCCGTGTCTTC
3739	KCNMB3	TTTGTGAACCTCAGCCATCC
3740	KCNS1	CGGCAAGCGCGTTCTCGCCT
3741	KCNS1	ACAGCTCGTCGAGCACGTGC
3742	KCNS1	GACATCCCGAGAAACCTCTC
3743	KCNS1	CACAGTGAGCATGACCACCG
3744	KCNS1	GTGGTGCTGCCAACACCCCT
3745	KCNS1	CACTTACCTCCTAGGGGTGT
3746	KCNT2	CTACTAGAAAACCCTTCACA
3747	KCNT2	TGAACAGAAGTCTACCTTTG
3748	KCNT2	TACCATTCATTTCCTTGTGA
3749	KCNT2	ATAACCTGTAAGCCCCACAA
3750	KCNT2	CTGTTTGAAACAATATTACT
3751	KCNT2	TTTATAGGTCTCATATCTTT
3752	KDM3B	TCTTGTATGGGCGCCCCGTG
3753	KDM3B	GCCTTGACTGTTACCGGCTC
3754	KDM3B	TCCTGAGCCGGTAACAGTCA
3755	KDM3B	CAAGTGTCGTGAATGCCGCC
3756	KDM3B	GGAAGTTATCGTGCTTCTGC
3757	KDM3B	GGATGGGTCATGCATCAATG
3758	KDM4A	ACTCCGCACAGTTAAAACCA
3759	KDM4A	GCGGAACTCTCGAACAGTCA
3760	KDM4A	TTCCACTCACTTATCGCTAT
3761	KDM4A	GTACGGCAAGCAAGCTGTGC
3762	KDM4A	CATCAGCTCACCTTGTCAAA
3763	KDM4A	TCACAGCTTTGAGCACTTCC
3764	KDM4B	CCCCGCGTACTTCTCGCTGT
3765	KDM4B	GTATGATGACATCGACGACG
3766	KDM4B	TCACCAGGTACTGTACCCCG
3767	KDM4B	CTGATGTCAGCCCCGTAGAT
3768	KDM4B	CATGACGTTTCGCCCAACCA
3769	KDM4B	GGCTCCCTGCGACTCTATGT
3770	KDM4C	CCTTTGCAAGACCCGCACGA

3771	KDM4C	AGTAGGCTTCGTGTGATCAA
3772	KDM4C	GTCTAAAGGAGCCCATCGTG
3773	KDM4C	AAGTAAGAAAAGCATCCCGA
3774	KDM4C	GAAGAAATGCATCACACCCT
3775	KDM4C	CCATATTTCTTCAATACTGA
3776	KDM4D	CCACGCCGATCTGCAGTTAG
3777	KDM4D	CGACCACGTCTGCCATTTGA
3778	KDM4D	TGCATGTTGTGCCCGCCAAG
3779	KDM4D	AACCTGAACGCTATGACCTG
3780	KDM4D	GCTCCAAATCTTCGAAATTC
3781	KDM4D	CAGATTATCCACCCGTCAAA
3782	KDM4E	GTTGCACAATGCGCACAAAG
3783	KDM4E	TGGGGTTCTGGTCGTGGTCG
3784	KDM4E	GGGTATGATATGCAGAGCTT
3785	KDM4E	CAGGCAAGACTTGGCCATTG
3786	KDM4E	GGCCAGCTTGATGTGCGCCT
3787	KDM4E	CTGTTCAGCCTGCATCCAAG
3788	KDM5A	CATGAACCCCAACGTGCTAA
3789	KDM5A	CTGGGATTCAAATAACTCGG
3790	KDM5A	TCTCTGGTATGAAAGTGCCG
3791	KDM5A	ACGACTGACCCAAGTGTCAT
3792	KDM5A	TTTGAATAACATGCCTGTCC
3793	KDM5A	GACCTCCCTTCTCTGCTATA
3794	KDM5B	GTCCGCGAACTCTTCCCAGC
3795	KDM5B	TCGAAGACCGGGCACTCGGG
3796	KDM5B	GGACTTATTTCAGCTTAATA
3797	KDM5B	ACTCCCAGTACTTTGCAATC
3798	KDM5B	CCTCCAATTCATTCAGTCTC
3799	KDM5B	TTATCAACATCACATGCAAA
3800	KDM5C	CTTACCGCCATGACACACTT
3801	KDM5C	GATAAACAATGCGTTCGTAG
3802	KDM5C	GGGCTACCCGAGCCCACCGA
3803	KDM5C	CATCCCCTCGAGAACACATC
3804	KDM5C	AGCTCTGCAGAGTGTATTCC
3805	KDM5C	GAATACACTCTGCAGAGCTT
3806	KDM5D	GATTTACTCCTCGCGTCCAA
3807	KDM5D	AAAGACTTACCGCGGGTGGG
3808	KDM5D	TAAGGCCCGACATGGAACCG
3809	KDM5D	TATCCTTATCCTTAGCCATA
3810	KDM5D	AGGTCCCAAAATGATGGGCT
3811	KDM5D	CCTCCAGTTCATTTAGCCTT
3812	KDM6A	ACTGTAAACTGTAGTACCTC
3813	KDM6A	CAGCATTATCTGCATACCAG
3814	KDM6A	AGACTATGAGTCTAGTTTAA
3815	KDM6A	TTATTGGAAGATTATCCAAA
3816	KDM6A	TTGGATAATCTTCCAATAAG
3817	KDM6A	AAGTCGTAAATGAATTTCCT
3818	KDM6B	TACCACAGCGCCCTTCGATA
3819	KDM6B	ATCCCCCTCCTCGTAGCGCA

3820	KDM6B	CAAAGGCTTCCCGTGCAGCG
3821	KDM6B	CGAGTCAGAGCACGATAGTG
3822	KDM6B	ACTCACCCTGGAGCATAATA
3823	KDM6B	CCAGAACTACTGCCATGTGA
3824	KDR	TTCCCGGTAGAAGCACTTGT
3825	KDR	GAGGTGACTGAGTGCAGCGA
3826	KDR	TGCTGCTGGCCGTCGCCCTG
3827	KDR	GGAGACCCGGGCCGCCTCTG
3828	KDR	GTGTTTCTCTTGATCTGCCC
3829	KDR	CAACTCTTCAAATTACTTGC
3830	KDSR	AACTCTGGTTGCACGAAATG
3831	KDSR	AATGCACTTCCCGATGCCAC
3832	KDSR	GACATGCTGGTAAATTGTGC
3833	KDSR	CAATTTACCAGCATGTCCAC
3834	KDSR	GCACTCTATTAATGACAAAC
3835	KDSR	CTTTCAGGATAAGCTGCTGC
3836	KEAP1	AGCCGCCCGCGGTGTAGATC
3837	KEAP1	AGCGTGCCCCGTAACCGCAT
3838	KEAP1	GCAATGAACACCATCCGAAG
3839	KEAP1	AAGTGCCACTCATCCCGCTC
3840	KEAP1	TCGTAGCCCCCATGAAGCAC
3841	KEAP1	CATTCGGGTCACCTCGCTCC
3842	KHDC3L	TCGCTGGGTCCCCGACTTGT
3843	KHDC3L	TGCATCAGTTGCACGAGCGT
3844	KHDC3L	AAGATCTCAGCCTCGCCGTT
3845	KHDC3L	AGTGACCCAGCGGTTTCGCG
3846	KHDC3L	CTTGATTCACGTGAATCGAT
3847	KHDC3L	TGAGTTCCTGAAGAATCCGA
3848	KIAA0825	ATGGGAATGAAACGCTGCTG
3849	KIAA0825	AATGAAGAGACGTCCTAGAG
3850	KIAA0825	GTTGTTTGCAGCTGCACACC
3851	KIAA0825	CAACAACTGACTGCTTTGAA
3852	KIAA0825	TGCTCAAACTCCAAGTCTCC
3853	KIAA0825	AGATTGAACAAAATGCTGCA
3854	KIAA1024	CAAGCTGCGACAGGTCGAAC
3855	KIAA1024	TTTCATCACGGACACCCGAC
3856	KIAA1024	TGCACGGAATGCGGGCGACA
3857	KIAA1024	AGCCGAATTCAGACGAGCCA
3858	KIAA1024	CCAACCTTCTTGGACCATTG
3859	KIAA1024	TTTAGTAATGAATCGTATCC
3860	KIAA1191	AGGTGCTGATACTTGCGCTC
3861	KIAA1191	AGAGCTTACGTGGAGTGCTT
3862	KIAA1191	CGCACTCTTCACCTAAGCAG
3863	KIAA1191	CAAGTATCAGCACCTCGCCA
3864	KIAA1191	GAAGAGTGCGGAGTGGTGCT
3865	KIAA1191	TCGAAGGTACTTGGTCTCCT
3866	KIAA1614	AATCGCTCGTGTGCCCGTTC
3867	KIAA1614	AGCCGTCCACGAGGGTAGGG
3868	KIAA1614	TAATGGTGGGCACGATGTCA

3869	KIAA1614	AGTTACTCCCGGACGGCCTG
3870	KIAA1614	CTGTTGCAGTACTCACGTCC
3871	KIAA1614	CTGGATCCTAGAGCGACCAG
3872	KIF18B	ACCTGACGTTTGTCTTTGAC
3873	KIF18B	CATCATGTACCTGACCACCG
3874	KIF18B	GGGGCCCCTTGCCATCCGCG
3875	KIF18B	TGGCGAGGCGGCCACCCAAC
3876	KIF18B	TGGTCAGGTACATGATGCCG
3877	KIF18B	TTGCACCACCACCCCCTTGT
3878	KIF1B	TCAGCTTCGACTATTCCTAC
3879	KIF1B	GCTGGTCTCTCGAGAATTGA
3880	KIF1B	AATGCTCTTACACGCCTTTG
3881	KIF1B	TAGTCGAAGCTGAAGGACTT
3882	KIF1B	CAATTCTCGAGAGACCAGCA
3883	KIF1B	AACCGTGTGTACAATGACAT
3884	KIF5A	GACAAATCTGTCCGTGCACG
3885	KIF5A	TGAACGCTTTGTGTCCAGCC
3886	KIF5A	GAACCGGGTGCCATTTGTCA
3887	KIF5A	ATTCTGGATGTGATTGATGA
3888	KIF5A	AAAATTCGTGACCTTCTGGA
3889	KIF5A	TTCTTACTTTGAAATTTACC
3890	KL	ATGTCATCGGGTATACCGCA
3891	KL	CGATGAATGAGCCGTATACA
3892	KL	CCTGCAGTACTATCGCTGCA
3893	KL	TCACATGAAGTTCCGCCAAT
3894	KL	GATCTGCAGCATAACGATAG
3895	KL	TTAACGACCGCACAGCTCCG
3896	KLF13	GGCGAAGTGGTCCACATAGG
3897	KLF13	CTTTCTCGCAGCCCGCGTAG
3898	KLF13	GAATGCTGCAGCGGCGCGGC
3899	KLF13	CCGCGCCGCTGCAGCATTCC
3900	KLF13	AAGCACAAGTGCCACTACGC
3901	KLF13	CCGACCTCGAGTCCCCGCAG
3902	KLF17	CAGTAGCTCATACCACGCTC
3903	KLF17	GCTCTCCTACGGGCATTAGT
3904	KLF17	CTTCATGTACGGCCGACCGC
3905	KLF17	ATGAGCTTAGACGACATATG
3906	KLF17	GTCGTCTAAGCTCATCAGAA
3907	KLF17	CCGGTGGCAGGCGGCGCACC
3908	KLF4	GTGGTGGCGCCCTACAACGG
3909	KLF4	AGCCCGCGTAATCACAAGTG
3910	KLF4	GCGCGGCGGCCCGCCGTTGT
3911	KLF4	TCTTTCTCCACGTTCGCGTC
3912	KLF4	CACCCACACTTGTGATTACG
3913	KLF4	GAGAAGACACTGCGTCAAGC
3914	KLHL32	TACAGGTGGAGTAACTAATA
3915	KLHL32	TACCTGAGGCAGCGTTCAGA
3916	KLHL32	CTCACCTGGATACAAGCCAG
3917	KLHL32	TGCCGTCTGAACGCTGCCTC

3918	KLHL32	CGACATCACCCTGATTGCTG
3919	KLHL32	TCTGCTGATTCAGCGCTGCC
3920	KLK13	CAGGGGTTAGGCGGTTGTTG
3921	KLK13	GTAGCCGCCCTTGCACTAGT
3922	KLK13	CAAGCACGCCCTAGGGCGTG
3923	KLK13	ACTCCTCATCTGAGCGAAGT
3924	KLK13	GTGTCTCAAGATACGTCCTG
3925	KLK13	CATCCAACTTCGCTCAGATG
3926	KLK14	TTGGTGGCCATACGTGCACC
3927	KLK14	AAAGCAGGGCGCCTCCGCAG
3928	KLK14	GCTGACAGCACTTCAAGTCC
3929	KLK14	GGACTTGAAGTGCTGTCAGC
3930	KLK14	ACCCAGAGCCCAAGACCCTC
3931	KLK14	GCTGTGTCTTCATGTCCCTG
3932	KLLN	CCGTGCACGTTTGGGGTTAC
3933	KLLN	ACCCGGCAGGATGGATCGCC
3934	KLLN	CCGCCGATTCTTACAGAACG
3935	KLLN	TCCGTACTTTCCACTCAACC
3936	KLLN	AACCCCAAACGTGCACGGTC
3937	KLLN	CAGTCGGAACTACTTTCCGA
3938	KLRK1	CTGCTTCATCGCTGTAGCCA
3939	KLRK1	TCAAGGGAATTTGAACTTCT
3940	KLRK1	GAAGTTCAAATTCCCTTGAC
3941	KLRK1	TCTGAAAGTATACAGCAAAG
3942	KLRK1	TTTGAGACATACAAGAAGCC
3943	KLRK1	TCATTATTATGGTAACAATA
3944	KMT2C	GGGGAAGCGCCATCTTTGCG
3945	KMT2C	GACACAGATCGCTGAAGAGT
3946	KMT2C	TCTTCAGCGATCTGTGTCTG
3947	KMT2C	AACAGAAACAGAAACGATTG
3948	KMT2C	GAAAACTGCAGTGGAAGATG
3949	KMT2C	TCCTCGGGGGGTGGTGGCGG
3950	KMT2D	CAGAGAGCACAACGCCGCAC
3951	KMT2D	CTCCCGCTGCCCGTGTAGAC
3952	KMT2D	CTCACCATTGGTGTGCTGCA
3953	KMT2D	CCGACCATGCAGCACACCAA
3954	KMT2D	CAGGCTTCAGGAGACTCCTC
3955	KMT2D	ACAGAGACCTCTCCCACATG
3956	KRAS	TCTCGACACAGCAGGTCAAG
3957	KRAS	CAATGAGGGACCAGTACATG
3958	KRAS	TTCTCGAACTAATGTATAGA
3959	KRAS	CAGGACTTAGCAAGAAGTTA
3960	KRAS	GATGTACCTATGGTCCTAGT
3961	KRAS	AGCAAAGAAGAAAAGACTCC
3962	KRT15	AGACCTGAGACGCACGATGC
3963	KRT15	GCTCCTGCATCGTGCGTCTC
3964	KRT15	CCACCACCATCGACAACTCC
3965	KRT15	TGTGGACCTGACCCGTGTGC
3966	KRT15	CCGGCGGTTCTTCTCCGCCA

3967	KRT15	TGATGTCAGCCTCAACGCCC
3968	KRT6A	TCTCTTTCAGGTGCGGTTCC
3969	KRT6A	AGGCAGCTGGACAGCATTGT
3970	KRT6A	TGGGATCGATTTGCAGGTTG
3971	KRT6A	AAATCGATCCCACCATCCAG
3972	KRT6A	TGAATTTGTGACTCTGAAGA
3973	KRT6A	CGCCTGGACTCAGAGCTCAG
3974	KRT75	CCTATACCAGTGAGCTCCGA
3975	KRT75	TGATGCTTTCCAGCTGCCGT
3976	KRT75	CAAGGTCAAATCTCTGCCCG
3977	KRT75	CTCGGGCAGAGATTTGACCT
3978	KRT75	CACCGACCTGGGTCTGCAAC
3979	KRT75	CGAGGTCAAAGCACAATACG
3980	KRTCAP2	ACCGAGTCTGTGTCACCACC
3981	KRTCAP2	CGAAGAGACCCGAACCAAGC
3982	KRTCAP2	GCAGATGTACAGCCGTCAGC
3983	KRTCAP2	AAGATTCTCCAGATTATTGA
3984	KRTCAP2	CAACCAGGCCTTCAATAATC
3985	KRTCAP2	GATGTAGTACAGACCAACCA
3986	KSR1	GTGGCAGACCTGCGACAGCC
3987	KSR1	CTCCAAATATCATGCTCTTC
3988	KSR1	AGAAAACTCACGTGGCAGGA
3989	KSR1	TCTACAGGCAGGGGCTTCTT
3990	KSR1	GCAGGTTCTCCACCAAGTCC
3991	KSR1	TGCCAGAAGAGCATGATATT
3992	KTN1	ACCTCTCTCTTATCTACCAC
3993	KTN1	CTGAAGACCGAAACTGACAA
3994	KTN1	CTCTCATGGTACCATCAAAA
3995	KTN1	TTGTGTTGTAGACTTGCTAA
3996	KTN1	AGAAAACATCATAGTCTTCA
3997	KTN1	TAAAGATGACCAGGATAAAA
3998	LAIR2	AGTTATAATGTGTTTCGACT
3999	LAIR2	TGCCTCTATTATAAGCCCCC
4000	LAIR2	CTGTTTACAGAAAGCTCTGG
4001	LAIR2	CATTCATGGTGCATCAAATC
4002	LAIR2	ATGACTTACCCTCCTGCGTG
4003	LAIR2	TTCTCTTCCAGTGCTCTGCC
4004	LAMA1	GGGTTTCGGACGGGCCGACC
4005	LAMA1	CGCTGTTGCCATCACAGATC
4006	LAMA1	CACCAATCTGGAATAATAGG
4007	LAMA1	GCTGCCAATGCCCCTCGACC
4008	LAMA1	TCACTGGGTCACAATCACTC
4009	LAMA1	CCAGTTATTGGTGCCATCTA
4010	LAMB2	CCAACGTGTGTAGACGAGTC
4011	LAMB2	CCTGACTCGTCTACACACGT
4012	LAMB2	CCAGGTCATCTATCGTGTGC
4013	LAMB2	GAAATATCGGTACACATGCC
4014	LAMB2	ACTACATCATCCCAGTGCCG
4015	LAMB2	ACTCTGAATCCGTGAGCTGT

4016	LARS2	CTGAAAATGCCGCAGTCGAG
4017	LARS2	CCGTGCTATGGTGTCGCTGA
4018	LARS2	CCATCAGCGACACCATAGCA
4019	LARS2	ATGTAGATTCCTCTCGACTG
4020	LARS2	TGGGCCAGATGTCATCAAGT
4021	LARS2	GCATCAACGAATAAAAGAAC
4022	LCE3D	TGGGCCTCTGGCGCCGGCAT
4023	LCE3D	CAAGGCGGGGGCTCTGGCTG
4024	LCE3D	GTGGGGGTTGGCACTGCTGC
4025	LCE3D	TGTCTGCCTCCAGCTTCCTC
4026	LCE3D	AGCAGCAGCCTCCAGAGCCA
4027	LCE3D	GCCCCAAGCTCTGGGGGCTG
4028	LDB2	CTGGTCGCAGTCCACCGTGA
4029	LDB2	TGCTTCTCGGGACTAACTCT
4030	LDB2	GGACTGCGACCAGTGTACCA
4031	LDB2	CATTTAGGCACAAGATCCTC
4032	LDB2	TGCAGGATAGTGACAACCTC
4033	LDB2	ATCTTACCCTGAGGTAGTTG
4034	LDHA	AGCCCGATTCCGTTACCTAA
4035	LDHA	TTGCTTATTGTTTCAAATCC
4036	LDHA	GGCTGGGGCACGTCAGCAAG
4037	LDHA	TACCTTCATTAAGATACTGA
4038	LDHA	GTCCAATATGGCAACTCTAA
4039	LDHA	GGGGAACATGGAGATTCCAG
4040	LDHB	TTACCCAAACACCGCGTGAT
4041	LDHB	GGGGAACATGGCGACTCAAG
4042	LDHB	ACCACAATTATGATGCAATC
4043	LDHB	TCCTGATTGCATCATAATTG
4044	LDHB	TGCATAAGATGGTGGTTGAA
4045	LDHB	GGCTGATGAACTTGCTCTTG
4046	LDHC	CTTCCAATTACACGAGTTAC
4047	LDHC	TTACCTGTAACTCGTGTAAT
4048	LDHC	GGGAGAAACTCGCCTTGCCC
4049	LDHC	TCATTATAGCCACATTACGT
4050	LDHC	TGTCCAATGCAACATCAACA
4051	LDHC	TGCCCTTGTTGATGTTGCAT
4052	LDHD	GGATTCGGTCCATATGCGTC
4053	LDHD	GCAGCACGGGCGCGATGAGT
4054	LDHD	GTTGAGCACGTTGTCCCGCA
4055	LDHD	GCCGGTGCCGAATGGGATGA
4056	LDHD	CATCGCGCCCGTGCTGCTCT
4057	LDHD	CGTGTGCAGCAGCCGCCCGT
4058	LDLRAD2	GCTCGCACGCCGCATCGCGC
4059	LDLRAD2	GTGGCTCCGGACACCGACTG
4060	LDLRAD2	CAAAGCTGCGTCTTGTCTCG
4061	LDLRAD2	GCCTACTTCCGCTGCCAGAA
4062	LDLRAD2	CGCCCTGACTGCAACTGCTT
4063	LDLRAD2	CAAGCCTCGTGTGTGACCCC
4064	LHX2	ACACTTCAACCATGCCGACG

4065	LHX2	GATCTGGCGGCCTACAACGC
4066	LHX2	CGTGGACAAGTCGACAGACG
4067	LHX2	GTTAAGACGGACGTCACAGT
4068	LHX2	CGCGAGCTGCTTCAAGTCCT
4069	LHX2	CGCGGCGCGGTCACTGCTGA
4070	LIAS	ACCTTAAGGTGACGCCTTGT
4071	LIAS	GAAGCTCGATGTCCCAATAT
4072	LIAS	TTAGGTTAAGACTACCTCCA
4073	LIAS	GCCAACAAGGCGTCACCTTA
4074	LIAS	AAGGAGCTTAACGGTCTGAC
4075	LIAS	CCCTACCATGATCGTGGCTG
4076	LIG1	GGGATTCCGAAGCGTCGCAC
4077	LIG1	TTCTCACCTGTGCGACGCTT
4078	LIG1	GGCTCTGAAACGCTTTCCGT
4079	LIG1	GGACCTACCTGGCCTTTAGC
4080	LIG1	CTTCACCGGAGAGTCACTCT
4081	LIG1	CGCAGAAGCAGAGACCCCGA
4082	LIN54	TGATAAGCCGTTGCTGTGGC
4083	LIN54	TCCAGCTCAGTATGTTACTC
4084	LIN54	GCAAGCAACTCTACCTTTAC
4085	LIN54	CATTGAATGGAAGCCGTGCC
4086	LIN54	GGGGTTGTATTAACAACCAC
4087	LIN54	TTGTTAATACAACCCCAGTG
4088	LIN7A	CTCCGTTCACTGATAGCAGC
4089	LIN7A	CTGTCCCGAATTCCGTGCGA
4090	LIN7A	TTTGGCAGTTCAACTACTCG
4091	LIN7A	GCTGAAGCCGAGCGTCACTT
4092	LIN7A	CAATGTCGCCATGTCTGCCG
4093	LIN7A	CCTTTGCTGTTGCCCTCGCA
4094	LIPI	GTGTAATCTAATCTGCTATA
4095	LIPI	ATTCACGGATACAGACCAGT
4096	LIPI	TATGGCTTCAGAACTTCGTA
4097	LIPI	AGCTTAGGGGCTCATATCAG
4098	LIPI	ATATAGCAGATTAGATTACA
4099	LIPI	TTTGTTGGAAAGATATTTCA
4100	LMNA	GCGCCGTCATGAGACCCGAC
4101	LMNA	GCCGAGCCTGAGCAGCTATC
4102	LMNA	CCTGCAGCGCATCCGCCAGC
4103	LMNA	TCTCAGTGAGAAGCGCACGC
4104	LMNA	CCAGAAGAACATCTACAGTG
4105	LMNA	CCTCACTGTAGATGTTCTTC
4106	LMO1	TGTGCCGACGCGACTACCTG
4107	LMO1	CCCACCTCAGGTAGTCGCGT
4108	LMO1	CGAGGTGATACACGTTGTCC
4109	LMO1	AGCGCCCGAGATGATGGTGC
4110	LMO1	CGGGACCATGGTGTTGGACC
4111	LMO1	GCTGATCCCAGCCTTCGAGA
4112	LONRF3	TGTGGGACGGCTTTAAGTGC
4113	LONRF3	GATTCGCCCCCGGGACGCCA

4114	LONRF3	AGCATGTAAGCTCCGCCCGA
4115	LONRF3	GATGGACCCAGCTAAAGTGA
4116	LONRF3	ACCGATTGCTATAAAGCAAG
4117	LONRF3	GCTCCCACATTGTTCTAGTC
4118	LPIN3	TCCAGCTCCGAGTCGCTCTT
4119	LPIN3	AACATCCCCTAAGAGCGACT
4120	LPIN3	GTCTCTCTAGGCATTATAAC
4121	LPIN3	CAGCGCTTTACTTCCCCCAA
4122	LPIN3	GTCATCCAGGTAGATGTCAC
4123	LPIN3	GGCTTGCAGGGAGAGGATCA
4124	LPO	CGTACCTGGTTCGGGAGTCC
4125	LPO	CCAACAGGGCTCTGGCGCGC
4126	LPO	GTTATTGCAGTCTCCCGTAA
4127	LPO	ACGCGCACAGCCATCCGCAA
4128	LPO	CTGACTCACAGTATCGGAGA
4129	LPO	CAGCCCTTACCGCACCATTA
4130	LRP1	CTGCCCAGACGGATCTGACG
4131	LRP1	AAAGTCCTTACCTCGGCAGT
4132	LRP1	GCTGGCGGTGCGACGGTGAG
4133	LRP1	GCCAAACGAGCATAACTGCC
4134	LRP1	CATCGAGTGTGGGGACACAA
4135	LRP1	CATTGTGTCCCCACACTCGA
4136	LRP1B	ACCCAATTCGTATGCAAAAG
4137	LRP1B	AGACACAAGTCACGTGATCG
4138	LRP1B	AGCAAATGGCACTGCGACTC
4139	LRP1B	GCCCATTTCCGCAAGAAAAC
4140	LRP1B	AAAGACTCGTCTGAATCATC
4141	LRP1B	TGGTGACCAGACAGATGAAA
4142	LRP2	CACGTCAGAGACTGCCCCGA
4143	LRP2	CGCGTCATCTGAACAGTCTT
4144	LRP2	CAAAGACTGTTCAGATGACG
4145	LRP2	TGGTCGCACCTGTATTCACT
4146	LRP2	AGCTGTTGTGACCTGCCAGC
4147	LRP2	GGATGCATTGTCCCTCACTC
4148	LRRC45	GACCTGCGCTGGAATAACGT
4149	LRRC45	GGCCAACGTTATTCCAGCGC
4150	LRRC45	CAACACCGTGCTGCGCTTTC
4151	LRRC45	TCTCCTAGGGCAACAACCTT
4152	LRRC45	TAAGTCCAGAAAGCGCAGCA
4153	LRRC45	TCTGAATGGACTTGTTCTGT
4154	LRRC70	AAGATGCTAGCCAGTCTCGA
4155	LRRC70	GCCTTGCCTACGACTGTTTC
4156	LRRC70	ACGTAATGCTCTGCCACGCA
4157	LRRC70	TTCATCAAACGCTTAGATCC
4158	LRRC70	TAACAACCAGAAACAGTCGT
4159	LRRC70	AATTCTTAGGAATACTCGAA
4160	LTB4R2	CGGCTGCTACAGCGTGACGC
4161	LTB4R2	CTTCAGTTCTAGCGTCAACC
4162	LTB4R2	CGGCCGGGACGGCGAGCAAC

4163	LTB4R2	CGAAAGCGGTCAGAGTCTCC
4164	LTB4R2	TCGGTCTGCTACCGTCCCCC
4165	LTB4R2	CCACGCAGTCAACCTTCTGC
4166	LUC7L3	GTATTTGTGAACAATTCCGC
4167	LUC7L3	CCGGGTAGATGACCATTTGA
4168	LUC7L3	CAATAGGCCGCTGGCCCAAC
4169	LUC7L3	AATTGATGTACTTCTGCAAC
4170	LUC7L3	GGCATCTCCTACTATTAAAA
4171	LUC7L3	ACAAATACACGTTCTGATCT
4172	LY6G6C	CAACCAAACCAACCGCAAGC
4173	LY6G6C	TGGTGTGCCACAGCGCAGAT
4174	LY6G6C	TCGCTGTCACTCCTGCTACA
4175	LY6G6C	GAGCAGCATAAGGGCTTTCA
4176	LY6G6C	CAAGGTATGCATGTGTTGTC
4177	LY6G6C	CCCTGTCTGTTCTGCTCTGC
4178	LYSMD3	TTACCGTACAACAGTACTGA
4179	LYSMD3	CAACGTAAAGACCCCTATTA
4180	LYSMD3	TCAATACTCTTCCGAACAAC
4181	LYSMD3	GTGTATGAACTTCGATCCAG
4182	LYSMD3	AGGACAAAGTGTTTCGGTCA
4183	LYSMD3	TCCCCAGTCTGCTCCATAAT
4184	LYST	CAATGTTATGAACACCCGAT
4185	LYST	TAAATCTGAAGCACGTCCTG
4186	LYST	ACTTCAAAGTATACGACTTT
4187	LYST	AGCTACTGAAGCCGCTCCCG
4188	LYST	ACAGGTATCTTCCATACCAG
4189	LYST	GATACCTGTCCAAGAAGAAA
4190	MACF1	CCTTTACAGATGAACGGGAC
4191	MACF1	GGTCCTCTCAGGCATCAAAC
4192	MACF1	GTCTTACCTGTCGCTGCTTT
4193	MACF1	TTGTTTGCAGCCCCGGGAGA
4194	MACF1	TCACCAGTTTGATGCCTGAG
4195	MACF1	TAAGTGCTTGTTGACCCACT
4196	MAGEA6	GCTCACCCAATATTTCGTGC
4197	MAGEA6	AAAGATGTACACGTGGCCGA
4198	MAGEA6	AGCTTCCGATTCCTTGCAGC
4199	MAGEA6	CTCTCAAAGCCCACTCATGC
4200	MAGEA6	GGAGGGAAGACAGTATCTTC
4201	MAGEA6	CCTACCCACTCCTGCATGAG
4202	MAGEB6	ACCCAGACAAGCGCGAGAAG
4203	MAGEB6	ATCCTCTTCTCGCGCTTGTC
4204	MAGEB6	TTCATATTCAAAATCCGATG
4205	MAGEB6	TTCTTTCAATTCAACGCCAA
4206	MAGEB6	GACAGCAGTAACACCAGTCC
4207	MAGEB6	GGTCTCTTGGCGTTTCTCAC
4208	MAGEL2	GTCGAGCCGCCACGCCGCTC
4209	MAGEL2	GGGCCTCTTCTATAGACCGC
4210	MAGEL2	CCCGCATCAGAACCGTAGGG
4211	MAGEL2	GCCTGTCTATAGCCGCCCTA

4212	MAGEL2	TGACTCGAGTCCTCCGGCGG
4213	MAGEL2	AAAATCCAAACGTACACTCG
4214	MAMDC4	ACTAATGTCCCGCCAGCCGC
4215	MAMDC4	TGGCGGGACATTAGTACCTC
4216	MAMDC4	CTCTAGATGACCTAGAGTTC
4217	MAMDC4	CTAGAGCCACAGCGCCCCTG
4218	MAMDC4	AGCTCACTCGGAAGTCACCC
4219	MAMDC4	CAGGAGTTGGCAGTGACCAC
4220	MAML2	AGTTAGCCATTACTTTCGAC
4221	MAML2	CAGCGGCCAGTCGAAAGTAA
4222	MAML2	ACGATAGCACTGTGCACTCT
4223	MAML2	GGCAGACAGCGATCCGAGCC
4224	MAML2	ATGTAACCACTTCCAGTGTT
4225	MAML2	GAGGTGGCCTTGACAAATGT
4226	MAP2K1	CCATACTTACTCCGCAGAGC
4227	MAP2K1	TATGGTGCGTTCTACAGCGA
4228	MAP2K1	CCCGACGGCTCTGCAGTTAA
4229	MAP2K1	TTGGAACAGGACCAACTTGG
4230	MAP2K1	CAATGGCGGTGTGGTGTTCA
4231	MAP2K1	CTTTCTCCAGCTAATTCATC
4232	MAP2K2	TACGGGGCCTTCTACAGTGA
4233	MAP2K2	GATCTCCCCGTCACTGTAGA
4234	MAP2K2	TCTCTGCAGGGCAAACCTGG
4235	MAP2K2	CCTCTAGGACGGCGGCTCCC
4236	MAP2K2	CCTGGGGAAAGTCAGCATCG
4237	MAP2K2	TTGACGAGCAGCAGAAGAAG
4238	MAPK1	GGTGCAGAACGTTAGCTGAA
4239	MAPK1	ACACCAAGTCCATTGATATT
4240	MAPK1	TGCAAACAGATATATAGTAC
4241	MAPK1	GCCTACAGACCAAATATCAA
4242	MAPK1	GTGTGGCCACATATTCTGTC
4243	MAPK1	GCAGTAGGTCTGGTGCTCAA
4244	MAPK10	CAGCTCCCGGTACGCTCTCT
4245	MAPK10	CACATGCCAAGAGAGCGTAC
4246	MAPK10	TGACAACCAGTTCTACAGTG
4247	MAPK10	CCACTTCCACACTGTAGAAC
4248	MAPK10	TCTTACAGAACTTATCTACA
4249	MAPK10	AACTGCTTGTCATATATCTG
4250	MAPK3	GCGTAGCCACATACTCCGTC
4251	MAPK3	CCACGTGCGCAAGACTCGCG
4252	MAPK3	CCCTCTGACCTTAAGGTCGC
4253	MAPK3	CCACGCGAGTCTTGCGCACG
4254	MAPK3	ATACCAAGTCCATCGACATC
4255	MAPK3	CGGAGTATGTGGCTACGCGC
4256	MAPK8IP1	TTGCCCTTACCCGTAAGGAC
4257	MAPK8IP1	ACTCATCAGTGATCTCCGAG
4258	MAPK8IP1	CCTCTGTGCTGCTATGCAAA
4259	MAPK8IP1	CCCTGACTTACCTGTCTTCA
4260	MAPK8IP1	ACGTCATTGCCCTTGTGATA

4261	MAPK8IP1	AAAAGCACAGTTGGCAGGAT
4262	MAPT	CTTGGCGGAAGACGGCGACT
4263	MAPT	GGGGGCCCACCTTCCGCTGT
4264	MAPT	CCGCTGCGATCCCCTGATTT
4265	MAPT	GATCTCCGTGTGGGGCTGCG
4266	MAPT	TGCTAAGAGCACTCCAACAG
4267	MAPT	AGATGTGACAGCACCCTTAG
4268	MAST1	GAACATACTGCCGCCGGGGA
4269	MAST1	GTATGTTCCGCCGCACCAAG
4270	MAST1	GCAGCTGCCGCACCAGTAAT
4271	MAST1	GGAGTGGGGTCTCGGTAGCG
4272	MAST1	TCTCATAGGCAGCAGTCCCC
4273	MAST1	AGGGACTCACCTTCGGGAGG
4274	MAT2B	AGGAAGTTAACATCCCTAAT
4275	MAT2B	GTAACCAGAACCCTCCTATT
4276	MAT2B	CCAGCTCCCCTAAATTTGTA
4277	MAT2B	CTGCACAATGTACTATAACA
4278	MAT2B	TGCTGCCTCTCAACTTAATG
4279	MAT2B	CTCTCTATACACTTTGTTCC
4280	MAX	ATGCACTGGAACGAAAACGT
4281	MAX	GCATCAAACTTTGACGATGA
4282	MAX	CGTACCGCAGATTGAAACCT
4283	MAX	TATTCCAGGAAGAGCAACCG
4284	MAX	GACTCAGTCCCATCACTCCA
4285	MAX	AAGTGTTCATAAGGAAAGAC
4286	MBD2	CGGCGACTCCGCCATAGAGC
4287	MBD2	GGAGCCGGTCCCTTTCCCGT
4288	MBD2	AGTCTTGAAAGCGCATGCCA
4289	MBD2	GCGGAGACTTGCCCTGTGAT
4290	MBD2	TGGTTCTGCCTGGAGTATGC
4291	MBD2	ACAAGCTCTGCGCCAATCAC
4292	MBD3	CGTGGTGTTGAGCCATACGC
4293	MBD3	GAGCACCTTCGACTTCCGCA
4294	MBD3	GCTTGAAGATGGACGCCGTC
4295	MBD3	GTTCCGCAGCAAGCCGCAGC
4296	MBD3	GGCCGAGAGCTGTCCCGTGA
4297	MBD3	TTGAGCTCACCTGGCGCGGC
4298	MBLAC2	CCTCTACCAGTTCGACCGCG
4299	MBLAC2	TCAAGAACGTTTCTACGAGT
4300	MBLAC2	GTAGGGGATGTGATCAACCT
4301	MBLAC2	TAAACTTGCAAGAGATCGCA
4302	MBLAC2	CGTCCGCACCACCTCGCTAT
4303	MBLAC2	CACGTGCACTTCGACCACTC
4304	MC1R	CATCGCCTACTACGACCACG
4305	MC1R	CGTTGCTCCCGCTCACCAGC
4306	MC1R	TGAGGGTGACAGCGCCTTTA
4307	MC1R	CGTGCTGTACGTCCACATGC
4308	MC1R	CTTGAAGATGCAGCCGCACG
4309	MC1R	TGGCCACCATCGCCAAGAAC

4310	MCC	CCCCGTCGCCGTCGCACGTC
4311	MCC	CGCCATACTCGAGCAGCTTC
4312	MCC	AACTCGACTACAGAGCGTGC
4313	MCC	CGCCCGATTAACCCCAGCAC
4314	MCC	CGGGCGCATGATGGCGGCCG
4315	MCC	CGGAAGCTCTTTGCATCAGC
4316	MCF2L	AGGTCCATTTGTCCCGTCGC
4317	MCF2L	CTTCAGCGAGATTCCGGACA
4318	MCF2L	CTTGTCCGGAATCTCGCTGA
4319	MCF2L	TGGCACTTACTGCGATGCGC
4320	MCF2L	AAGCAAAGCGCTTCTTTAGC
4321	MCF2L	AAGCGCTTTGCTTACCTGTC
4322	MCF2L2	ATTTACAGCTATTCGTGTCA
4323	MCF2L2	AGTCAGACCCCTGATGGCGG
4324	MCF2L2	CTATTATCTCCACCGCCATC
4325	MCF2L2	TGAGTTTAAAACGAAAGTGC
4326	MCF2L2	TCGACAGACGAAGAGACAAG
4327	MCF2L2	CAATGTCAGTGAATGTCCTC
4328	MCM2	CTACCAGCGTATCCGAATCC
4329	MCM2	TTGGTGATGCGGTCGTACTT
4330	MCM2	CGCCATTCTCCTCGCAGATC
4331	MCM2	AACCAGCTGATCCGCACCAG
4332	MCM2	AGTCCTCATAGTTCACCACC
4333	MCM2	CAACAAGTGCAATTTCGTCC
4334	MCM3AP	CCACTTCGAACACGCTCAGC
4335	MCM3AP	TCGGGTGAAGAGAACCGATC
4336	MCM3AP	CGTGCCCATGTACTCTGACG
4337	MCM3AP	CCCTACCTCGTCAGAGTACA
4338	MCM3AP	TTTCCAGGCATCTGCAGCCC
4339	MCM3AP	GAAAAGTTTGCATAAAGACA
4340	MCRS1	CCCTTTACAGGTTCATCAAG
4341	MCRS1	GCCTGGCAAAATCTTCTACC
4342	MCRS1	GCTGCTAGATTCATCCCTGA
4343	MCRS1	CACGACATGACACGTGGCAC
4344	MCRS1	TGACCAAGGATCTGGGCCGC
4345	MCRS1	CTGGAGGAGCTTCTCCGTTT
4346	MDGA1	CGAGCGTATTGCACGCACGC
4347	MDGA1	CGAGGCGCTACCTGCCGTCT
4348	MDGA1	CTCGCACATCGCTCACCGTC
4349	MDGA1	CAGACGGTGAGCGATGTGCG
4350	MDGA1	CGTGTGCGGCATCCCAGACA
4351	MDGA1	ACACGTTACGCACAGACACC
4352	MDH1	TCAAACACCGTAACGTCCTT
4353	MDH1	GGTGTTTGATAAGGACGATA
4354	MDH1	TACCTTACCTTGATTACAAC
4355	MDH1	CCAATCAGAGTCCTTGTGAC
4356	MDH1	ATCTCTGATGGCAACTCCTA
4357	MDH1	ACCGTCCAGGACACCCATCA
4358	MDH2	TGGTGTTGAACAGGTCGTCC

4359	MDH2	GATCTGCGTCATTGCCAATC
4360	MDH2	ACAGGGACGTTGACTCGAGC
4361	MDH2	CGAGTCAACGTCCCTGTCAT
4362	MDH2	CAGGCAGCTGTTCAGGTCCG
4363	MDH2	CTACCACATCACAACCTTTC
4364	ME1	TGCCGTAGTCCAATGTAGAG
4365	ME1	CTTAAAGATCCACTCTACAT
4366	ME1	TTGGCTCTATATACAGCTTG
4367	ME1	TATAGAGCCAATTTACCCAC
4368	ME1	ACACCTGATTGTGATGGCCT
4369	ME1	TCATCACTGAGGCTGCCCTA
4370	MED7	TTTCAAGCCGTTGACGTTTC
4371	MED7	TTGCTATCATCAGCATCCAT
4372	MED7	TGATCTTATCATCCGCCCTT
4373	MED7	GATGATAGCAACAATTGTAC
4374	MED7	TCGATGCCCTGACTTTCCAA
4375	MED7	GGATGATAAGATCATCACAT
4376	MEF2B	GAGTCACTTACATACAGCCG
4377	MEF2B	CGGAGGTGTTTAGTCCCCCT
4378	MEF2B	TACGGGGCCTTACCGCCACC
4379	MEF2B	CCCGTATACCACATCTGGGC
4380	MEF2B	ACCCCCACTGTACCTGCCGA
4381	MEF2B	ATCCGGCCTTGCCCCGACCC
4382	MEGF9	AGACCGATTATTGCATTGGC
4383	MEGF9	TGAGTGTCGGCCAGGTTATC
4384	MEGF9	CAGTGAAGCCCCTGATAACC
4385	MEGF9	TGCCCTGCCAATGCAATAAT
4386	MEGF9	GTTGATGCACTCACCACTCT
4387	MEGF9	AAGATTTGTAAGCCCGAGAG
4388	MEMO1	CTTTAGTTTACGGAGAACTG
4389	MEMO1	AAAGACTTACGGGGCAATAA
4390	MEMO1	CAGCTGAATGCACAGCTAGA
4391	MEMO1	CATGCAGGATATACGTACTG
4392	MEMO1	GATCCACTTGTTTATAAGCA
4393	MEMO1	TCGATGTGCACTTTCCAGTG
4394	MET	CACATGGCAGATCGATCCAT
4395	MET	GACCTCACCATAGCTAATCT
4396	MET	GCAGCGCGTTGACTTATTCA
4397	MET	TAGGTTGTGGTTTCTCGATC
4398	MET	GCCAGATTCTGCCGAACCAA
4399	MET	GTATGCTCCACAATCACTTC
4400	MFSD10	CGCTTCTCGGCTGTCGCTCG
4401	MFSD10	TGAGCAGATCAGCCGCATCA
4402	MFSD10	TCGCCTCTGGCCCGCAGTCA
4403	MFSD10	CACGGCCATCGTTGCTGACC
4404	MFSD10	ATCCCCTAGGACCCCCTCTA
4405	MFSD10	CACCGCCATCGGGATGCCAG
4406	MFSD2A	GGGAGACTCACGATAGGCGG
4407	MFSD2A	AGTCGGGCACGAACCAGATG

4408	MFSD2A	GAACATGGTGAGAGCCGAGT
4409	MFSD2A	TTCGTGCCCGACTTCCCACA
4410	MFSD2A	AAGCTACTGTAGAGCTATTG
4411	MFSD2A	GGAAACAAGGCGTGTCTGCT
4412	MFSD6	AATATAGCGAGCCGTATTGC
4413	MFSD6	GACTAACACGCCTCCGATCA
4414	MFSD6	TCCTATAGCAACCATCGACT
4415	MFSD6	CTGTTGCTGTACCAAGTCGA
4416	MFSD6	GAGCAGTAGGATCACCAAGC
4417	MFSD6	GGCGTGTTAGTCAATTATTT
4418	MICAL1	GGGTGCTGCTCGTAGCCACC
4419	MICAL1	TCTTAGCACCGAGTGCCCGC
4420	MICAL1	CACGTTGTGGCGAGAGAACT
4421	MICAL1	CGTTGACACAGAGGCGTTCC
4422	MICAL1	GCCCCAGTACCACAAGATCA
4423	MICAL1	ACCAAGCTGGACAAGCGAGC
4424	MICALL2	CCTTTCGCAGGCCTTCCGCG
4425	MICALL2	ACTCACTGGGGGAGCGGCCG
4426	MICALL2	GATAGTGGAGGCTTCCGGGC
4427	MICALL2	AGGCGTGAAGAGGGCCTCGG
4428	MICALL2	GAGAGCGAATCTGAAGCCCG
4429	MICALL2	AGATTCGCTCTCCATCCTGC
4430	MICU1	ATGGATCGCACAAAATCTTC
4431	MICU1	TATATAATAAAACGCTTTGA
4432	MICU1	ATAAACACTTCTGCTTCACC
4433	MICU1	ATTCAAATCAAACATCTTGA
4434	MICU1	AGATATGGAAGAATTTGAAC
4435	MICU1	CTCACTGATGACTTTCAAGG
4436	MIER1	GGACGTGGTTAAGAACCAAC
4437	MIER1	TGCATTGAACCTCAACCAGT
4438	MIER1	CAGTACTACCATAACCATAA
4439	MIER1	TGATGAACGAACATTAGAAG
4440	MIER1	TGATCCATCAGCTGACATGC
4441	MIER1	CATGAACTTCTCAGCCTTTA
4442	MINPP1	ATCCAGCTGCACCTTGTTTC
4443	MINPP1	TGCTACAGCTCTTTATCACG
4444	MINPP1	ATGTGCTTATTTCAGCTGTG
4445	MINPP1	TTAAAGGTGTTAAATCTCCT
4446	MINPP1	TTTCACCTGTTCATTTGACC
4447	MINPP1	CCTGCATTTAAATCATTTAC
4448	MIS18A	CACCGACGCGTCTTCGCTCA
4449	MIS18A	CTCCATGAGCGAAGACGCGT
4450	MIS18A	CCTTGAGACTTTGTGCTGCG
4451	MIS18A	GAAGCTATCCAAACGTGAAA
4452	MIS18A	CCGCGCAGCACAAAGTCTCA
4453	MIS18A	AAGCATTACAAATGAAGCTG
4454	MKLN1	GTGATATTCTGAACTATAGC
4455	MKLN1	ACTTGATTCTAAAGCTCGAA
4456	MKLN1	CCTTGTCGATTCATTAAAAT

4457	MKLN1	CCAGCTTTAACTTTAGCATC
4458	MKLN1	CCAGATGCTAAAGTTAAAGC
4459	MKLN1	TAGTACAACCTTGTCTCAAC
4460	MLH3	AGAACAAGGTCGCTTCTAAA
4461	MLH3	ACAGACCCACCTCTGGATAA
4462	MLH3	GTCCTCTCTGCTCGAGCTCT
4463	MLH3	TTGTGCCTGTTGCTTCTCGT
4464	MLH3	TATCCCTATCGTTTCACCAA
4465	MLH3	AGTATTTGCCCGTTATCCAG
4466	MLLT1	GGAGCCCCCCTACAAAGTAG
4467	MLLT1	GTCGTGAGTGAACCCCTCCG
4468	MLLT1	CCACTCACCGCGTCTGGGCT
4469	MLLT1	CCCCGACTCCTCTACTTTGT
4470	MLLT1	GGCGCCAGCCATGGACAATC
4471	MLLT1	GCTCCTCACCTGATTGTCCA
4472	MLLT3	GTGGATGGCCTTCAAGATGC
4473	MLLT3	TAATCAAAGCGGACTTTCCT
4474	MLLT3	GCCCCCAGGCATGGCTAGCT
4475	MLLT3	CCTTACAAAGTAGAAGAATC
4476	MLLT3	CCAGATTCTTCTACTTTGTA
4477	MLLT3	GACTTATTCCTGCATCTTGA
4478	MLST8	CGCCGCCCGTGTACATCCAG
4479	MLST8	CAACAAGAACATCGCGTCTG
4480	MLST8	CGATCCCGACGCCAGCTACA
4481	MLST8	ATCGATGTGGGCGGACGTGA
4482	MLST8	CGCAGCATGATTGCTGCTGC
4483	MLST8	GTCCCAGCAGGTGAATGCCT
4484	MMP8	AACGCACTAACTTGACCTAC
4485	MMP8	GCAACCAGTATCAGTCTACA
4486	MMP8	TAACTTACTTGCGGAGGTGT
4487	MMP8	ATTCCATTGGGTCCATCAAA
4488	MMP8	TGCAACACTCCAGAGTTCAA
4489	MMP8	CTATACCCCACAGCTGTCAG
4490	MNX1	CTACGGCTACTCCGCGGCGG
4491	MNX1	GGCGGCGCTCTACGGCCACC
4492	MNX1	CGAAGCGCTTGGGCCGCGAC
4493	MNX1	CGTTACCTTGTTGGGCGCCC
4494	MNX1	ATGGGGGGACTCTCAACAGT
4495	MNX1	AGGACGACTCGCCGCCCCCG
4496	MOCOS	GTAGAGGAGCCGCACGATTA
4497	MOCOS	CCGTAGCCGTAGGCTAGCCG
4498	MOCOS	GGCGCGCGAGTTCAGCCGCC
4499	MOCOS	CTCTGAGCTACCGACTGCCT
4500	MOCOS	CAAGCTCACCCATGACACTG
4501	MOCOS	GCACCTGCTCCACAGTGTCA
4502	MORC2	GCTTGGCCGACTTCCCAAAC
4503	MORC2	ACACCTGAGTCTACTCAGAT
4504	MORC2	GCCACACTCACGTGCCCTCT
4505	MORC2	TCTGCCATCTGGATATCTCT

4506	MORC2	TGCCCAATCTGAGTAGACTC
4507	MORC2	TTTGAGATTATGTCTAGTTC
4508	MPC2	TGATGCTGCCCGAGAAATTG
4509	MPC2	GCATCAGCTCCACTTTATCG
4510	MPC2	AGACTTACCCATTTCATAAT
4511	MPC2	AGCCCAGAAGAAAACTGTTC
4512	MPC2	GTGTAGGGGTTGGTGTGTGC
4513	MPC2	GCCTGCGGGCCACCTACCAC
4514	MPG	CGTAAATGATGTACACGTAC
4515	MPG	TGCAGAGCTCGCGGTCCTTG
4516	MPG	TTGCGTCTTGCTGCGAGCAC
4517	MPG	CCTAATGGCACAGAACTCCG
4518	MPG	CCACCTTACCCGACTGGGGT
4519	MPG	AAGCAGCGACCAGCTAGAGC
4520	MPND	CCCAGCATGCAGTAGCGGAC
4521	MPND	CGCCTCTCACCCGGGTGGGC
4522	MPND	TGCCACATGATCCTTCCGTC
4523	MPND	ACGTACCTCATCAGCAGCCG
4524	MPND	AGTCTGACCTGGCCAAGTCG
4525	MPND	TCTCAGCAGAGGACAAGAGT
4526	MPP5	TTGAGCAAGATCTTGTGCGT
4527	MPP5	TGCTACAGTTCGTAATGAAA
4528	MPP5	ATCATTAGCCGGATAGTAAA
4529	MPP5	GAGAATCTACCAAAGTATGT
4530	MPP5	GCTGAATACTCCACATATTC
4531	MPP5	TTTGAAGCCAGTTCATCATA
4532	MPP7	TCATTAGTTTGTCGCCGATA
4533	MPP7	GCACTGTCACGCCATAGTGC
4534	MPP7	ACGAAGAAGTGACACCGTAT
4535	MPP7	TTCATTCAGCCCTACTCCCA
4536	MPP7	TCTTCCTGGCTATCCACATG
4537	MPP7	TATAGGTCTGTATGAGCTGT
4538	MPPED2	AGATGCAGACAAACCGCGTG
4539	MPPED2	CAAAGGACCCCGTGGTTTAA
4540	MPPED2	GGATGTCCCCATAAGGCATC
4541	MPPED2	AAGCCCCATCCATTAAACCA
4542	MPPED2	CGGCCCAAGCTCCATGTGTT
4543	MPPED2	CATGGCAGACCTTGTTAAAC
4544	MRPL12	CTTCGCCTCGGTCAGGCGGA
4545	MRPL12	TCTGTGCCGTGCGACATATG
4546	MRPL12	ATGTGTCCGTTCTTTCGCTA
4547	MRPL12	GCTGCTCCTCATATGTCGCA
4548	MRPL12	GTCGGGCTTGTGCCGATGGG
4549	MRPL12	GGTGGGCGGCACCGTGGTTC
4550	MRPL40	CTAGCCCTGCGCCCGACTAG
4551	MRPL40	CAGGTTAGGATCCCGCTTGA
4552	MRPL40	TCCGCACTCACCCGCTAGTC
4553	MRPL40	AAGACAACAATGACGCTCGC
4554	MRPL40	CAAGCAGCAAGAGCGTAAGA

4555	MRPL40	GAAAAGGAAGATCCGAAAAC
4556	MRPL49	CGAGAGCGTCGTACAAAGTA
4557	MRPL49	AGCGCCTGTTACCGGCTACC
4558	MRPL49	GACAGGTACCCTACGGATCA
4559	MRPL49	CTTACTTTGTACGACGCTCT
4560	MRPL49	TCCATCCCCGCAGCGTAGCC
4561	MRPL49	GAACATTATCCTACCCCTAG
4562	MRPS15	CCGGACGACATATCCGCGCG
4563	MRPS15	CCGCGCGCGGATATGTCGTC
4564	MRPS15	GGTCATCATCCAGCCTAGAC
4565	MRPS15	GTAGTCTTTGAGCAGCGTAG
4566	MRPS15	CCGACCAACTTCGAGCCTCC
4567	MRPS15	CGTGAAAAGACTCTTGTCTT
4568	MRPS30	GGTGAAGAAATTATTCCTCG
4569	MRPS30	AATATCCAAGCAACTCGCAG
4570	MRPS30	TGGGTGTGACCGTAACAGCA
4571	MRPS30	TCAGTGCCAAAGTATTTAGC
4572	MRPS30	GGAGCACAAGCTATGTATCA
4573	MRPS30	TCATACTGCCGTTTGAATAA
4574	MSC	CTCTGCCGACGAGTTGTCAC
4575	MSC	CCAACGCCCGTGAGCGTGCC
4576	MSC	TCCGCAGCCAACAGACTATG
4577	MSC	GGTGGTTCCACATAGTCTGT
4578	MSC	CTTTGCTCAGCACGCGCATC
4579	MSC	CAACGGGATGTCCACGGGCT
4580	MSH5	TGTTGTTACGAGTGCCAAAC
4581	MSH5	TGAGAATATGACTCGATTTC
4582	MSH5	TCAGTGGCAGTCATGGCGTC
4583	MSH5	AGCAAACAACGCCTCCTTTC
4584	MSH5	CTCTCGTGGTCTGGGGCATC
4585	MSH5	GGAGTCACTAGTATCATAGT
4586	MTA1	CTCTGCCCGCCACGCACATC
4587	MTA1	ATCGGGAGCTGTTCCTCTCC
4588	MTA1	CAGCTGCGGCGCTCATGTGC
4589	MTA1	ATCACCGACTTGTTAAAAGA
4590	MTA1	GATAAAGGAGAGATTCGAGT
4591	MTA1	GCTCTGTGGGCACCTTCGCA
4592	MTA2	TTACCCTCTACTAGGCGATC
4593	MTA2	GATCCCAGATCGCCTAGTAG
4594	MTA2	GCTCCTCAATCCGTCTAACC
4595	MTA2	TGTCCTGTACCGTATGTGGG
4596	MTA2	ACGGATTGAGGAGCTCAACA
4597	MTA2	CATTACCAGCCACCCACATA
4598	MTA3	TCAATCTGTCGATCCGTAAG
4599	MTA3	ACCGTGCTACAACTAAAAAC
4600	MTA3	GGATACCTTCTTCTACTCAT
4601	MTA3	GCAACCCATACCTAATAAGA
4602	MTA3	AAAACACTATTAGCTGACAA
4603	MTA3	GTTCTTCTATCCTTCTTATT

4604	MTF1	CCTTCGTGTGCACTCGCACG
4605	MTF1	GCGGTACCAATGTACCTTTG
4606	MTF1	AAATTTAGGTGCGATCACGA
4607	MTF1	ACTCACGTTCGTACACATAC
4608	MTF1	ACGTTTAACTGTGAATCTGA
4609	MTF1	AAGCACATTCGAACTCATAC
4610	MTFR1	GTTCGTCTCGAAGTATCGTA
4611	MTFR1	ATACTTCGAGACGAACCATA
4612	MTFR1	TCAGCTGAAGACCCCAGCGC
4613	MTFR1	TGCCAAAATTGTGACCCAGC
4614	MTFR1	GACTTAGATTCTACCACATT
4615	MTFR1	GATCAACAGCAGATGTACTT
4616	MTHFD1	CAGTCGATGACTATTGCCCC
4617	MTHFD1	TTTCATTCCTTGTACGCCTA
4618	MTHFD1	AAAGGGGAGTGGATCAAACC
4619	MTHFD1	AGATTGACTAGCATCAATGC
4620	MTHFD1	TCTTGGTAACTTAATGTGAG
4621	MTHFD1	AAAGCTCACCTCAGATTCTG
4622	MTHFR	GCCGTCCTCCGACCCCATCG
4623	MTHFR	CGCCAGGGGCTCATCGTTCC
4624	MTHFR	TAGTTCGAGATGTTCCACCC
4625	MTHFR	GGAACATCTCGAACTATCTT
4626	MTHFR	CACTCACTTTGTGACCATTC
4627	MTHFR	ACTTGCTCTTCAGGTAGAAG
4628	MTMR8	CTGAAGCCTCCACATAGATC
4629	MTMR8	CCCTCCGCTGCAAGAATTTC
4630	MTMR8	ATACGCCCAAAGTCTGATAT
4631	MTMR8	CCACCCGGAAATTCTTGCAG
4632	MTMR8	ATTGACCCAATATCAGACTT
4633	MTMR8	TGCAACCCACCTGATCTATG
4634	MTOR	GAGGAGGTTCCGAAGATAGT
4635	MTOR	GCTGGGTGCTGACCGCAATG
4636	MTOR	AAAGGTGGCATCTTGGCCAT
4637	MTOR	AAGCACCTCTCGGAGTTCCA
4638	MTOR	GCTCCAGCACTATGTCACCA
4639	MTOR	CCAGCTCAGATGCCAATGAG
4640	MUC16	AACACACTCGATGGCGACTC
4641	MUC16	GGTGAACTCGTTACGGGCTC
4642	MUC16	TGTCCCAGAAACCGTTGTGC
4643	MUC16	ACTAATGAGGCTATCGTCTT
4644	MUC16	ACTTCGCCTGCCAGTCCTAA
4645	MUC16	CACCATAAAGATGTCAAGCC
4646	MUC17	TCTGCGGCAAACACCGCCAC
4647	MUC17	GACGGTTCAAGACGTCCCCT
4648	MUC17	AGTCACACTGGACTCAATCG
4649	MUC17	GCCTCCATTCTTGCAGCGAG
4650	MUC17	TAACGTGCTGAGACAGCTGC
4651	MUC17	CACACAACTCCCCATAATAG
4652	MUC2	ACCAAAGTCTACTCCCGCGC

4653	MUC2	AACTTCGCCTCCGACTGCCG
4654	MUC2	GAAGATCAACCAGCCCGATG
4655	MUC2	CGGCCCCGTTAAGCACAGCC
4656	MUC2	GGCCGTTGTAGTCCCCGCAG
4657	MUC2	TCCGGGCCTCACCGTCAGAG
4658	MUC4	CGTTCTTATACCACGTTCCA
4659	MUC4	CACCTTCGGCCGCTACTGCG
4660	MUC4	CTGGAAGACCACGTCGTTCC
4661	MUC4	TGTCGGTTGCCTGGGACGCC
4662	MUC4	AGTGCCGCCCATCCCCAGTC
4663	MUC4	GCGAGTGGCACAACACCTTC
4664	MUM1L1	AGTTACAGCCATCGGTTCCC
4665	MUM1L1	CTCTGACTGTAGTCCTAATG
4666	MUM1L1	GAGTTGTTCAAGGGAATGCG
4667	MUM1L1	GAGACACATCCGTTTGAAAC
4668	MUM1L1	AGAGAAAGATTAATGCGACT
4669	MUM1L1	TTAGTGCCTCTAACCCTGTC
4670	MUT	GGAGCAAAAGTTATTGCTAC
4671	MUT	CCAACATCACCACGAACTCG
4672	MUT	ACGATGTGTCGCCAGATCAA
4673	MUT	GTACTCTAGAACTGGACTCC
4674	MUT	CTGGCCTATACTTTAGCAGA
4675	MUT	TCGTCTTCTTGTAGCAAAAA
4676	MUTYH	CTTGGTCGTACCAGCTTAGC
4677	MUTYH	ACTGTGATCAACTACTATAC
4678	MUTYH	CTGGACAGGCGGGCATATGC
4679	MUTYH	GCTGGTACGACCAAGAGAAA
4680	MUTYH	GCATGCTAAGAACAACAGTC
4681	MUTYH	TGGAGTCACCTGCATCCATC
4682	MX2	CTCGCTCAGGAATCGTAACC
4683	MX2	CATTCCCGGCCATGACGTTC
4684	MX2	GTTGAACTTACCGCTGCCTC
4685	MX2	GACAACCAGCCCCGAGACAT
4686	MX2	TGTGGTGGCACTGTGCCGAA
4687	MX2	GTTCCGGTAGCTGATCCTTC
4688	MXD3	GCAGCGGGCCCGACAGCTCA
4689	MXD3	CTGTGCCCGCATCGCAGTCC
4690	MXD3	ACCGGGCACAGTCGGCCCCC
4691	MXD3	CGCCCGCTGTCCTGCGCGCC
4692	MXD3	GTCTGAGTCTGAGCGCTCAG
4693	MXD3	ACAGGAGGGCCCAGTTGAAG
4694	MYB	CAAGTCTGGAAAGCGTCACT
4695	MYB	CTAACTTACCGGGAGATAAT
4696	MYB	CAGCATATATAGCAGTGACG
4697	MYB	GAAATCGCAAAGCTACTGCC
4698	MYB	GTACTTTCTGCCATCGGTGC
4699	MYB	TGTTATTGCCAAGCACTTAA
4700	MYC	AACGTTGAGGGGCATCGTCG
4701	MYC	GCCGTATTTCTACTGCGACG

4702	MYC	TGCGTAGTTGTGCTGATGTG
4703	MYC	ACAACGTCTTGGAGCGCCAG
4704	MYC	CGCCGTCGTTGTCTCCCCGA
4705	MYC	TCGCTTACCAGAGTCGCTGC
4706	MYO15A	TACACCGTCCCCTATGCCGA
4707	MYO15A	TGGCGCGTCGTACGGCGCAT
4708	MYO15A	TCCGTTCAAATCTAATCTTG
4709	MYO15A	TCCGCAACCTGCCATCCATG
4710	MYO15A	TGTGGAGGACATGACACAGC
4711	MYO15A	TGGACAGCACAGTGGTTTCC
4712	MYO5C	GTGGTACGCACCTCGGTGAT
4713	MYO5C	GGACCCACACATATTTGCCG
4714	MYO5C	CCCGCAATGACAATAGTAGT
4715	MYO5C	GCCCATGTTCTGCCCGCTGT
4716	MYO5C	TGACTCCCCACTTACAATTA
4717	MYO5C	ACCGACTACTATTGTCATTG
4718	MYOG	AGCTCCGTCCGCTCGTAGCC
4719	MYOG	TAAGAGGAAGTCGGTGTCCG
4720	MYOG	GCCTTACTTACCCCCTGGGT
4721	MYOG	GCTGTGAGAGCTGCATTCGC
4722	MYOG	CACTGTGATGCTGTCCACGA
4723	MYOG	CCTTACAGATCATCTGCTCA
4724	MYRIP	CTCCTACCAGAAGCACGAAA
4725	MYRIP	CTGTCTAGGACATAGTGTGA
4726	MYRIP	GCTGCGTCTGCCAGCAAGCG
4727	MYRIP	CATCATCAGTCAAACCAGAC
4728	MYRIP	ACACCTTGGCTGTGGCCCTA
4729	MYRIP	TGAAACAGAGCATGTTCTTC
4730	MYSM1	AGGCTAAATTTGGCCGAAGA
4731	MYSM1	TGTGGATATCGAAGGGGACG
4732	MYSM1	TCCCATCATGGCGGCTGAAG
4733	MYSM1	ATCACTATCTTGATTCATCT
4734	MYSM1	TCTTCACTTGTAAAACAGTG
4735	MYSM1	GCGGCGGCGGGGGCACAGCC
4736	MZT2A	GGCGGAACGCAGCAACCACG
4737	MZT2A	TCCTAGGTGCTGCCCTGCGT
4738	MZT2A	GGCCTCGCGGATGGCGGCGC
4739	MZT2A	GGAGCTGTACGAGCTGGCTC
4740	MZT2A	GGACCTGCTGAAGCTGAACG
4741	MZT2A	CCAGCTCTGACCTCGGGTCT
4742	NAA10	AGGCTGTGAAGCGTTCCCAC
4743	NAA10	CGGTCTGGCTCAGAAACTGA
4744	NAA10	TGCAGTGCTGCATGTTCATT
4745	NAA10	CCAATGAGGTGATATGTCCA
4746	NAA10	ATGAAATACTACTTCTACCA
4747	NAA10	GACCCAGATGATGTGCCCCA
4748	NAA60	AGTCGCGTACATCCTAAGTC
4749	NAA60	TCCGCACAGAGTCTACCGCC
4750	NAA60	GGTGGCCGCCGTTGATGTAG

4751	NAA60	TCACATATCAACCACCGCCC
4752	NAA60	ATATCACGATACCATGAGTC
4753	NAA60	TTGGAAGAGATGCCCGACCA
4754	NABP1	GTCAACCGAATAATATCCCC
4755	NABP1	GATATTATTCGGTTGACCAG
4756	NABP1	TTCTGCTGTCCTCGATAATC
4757	NABP1	TACAGGTACATTTGGACCAG
4758	NABP1	TGTACCCATATTACTATTCA
4759	NABP1	GAACCCAACCCAGATTATCG
4760	NAE1	TCAAGGAGCAGAAGTACGAC
4761	NAE1	ATGGACTAGTTGGTTATATG
4762	NAE1	AGGCGCGGCCATGGCGCAGC
4763	NAE1	TCATCCAGATAATGCATTAG
4764	NAE1	TACGCTTAGCAGATGTCCTC
4765	NAE1	AGATCCTCTAATGCATTATC
4766	NAP1L1	CTATCAGCCTCTATTTGATA
4767	NAP1L1	CACCAACAGGATACATTGAA
4768	NAP1L1	AGCATTACCTTATCAAATAG
4769	NAP1L1	CATTCACATTCTTCTTCCGT
4770	NAP1L1	TACCTTTCAATGTATCCTGT
4771	NAP1L1	TAATGCAATTTATGAACCTA
4772	NAP1L2	CTCAATCGCAGTTCCCCTAT
4773	NAP1L2	ACACGGTACTTAACTTTCAC
4774	NAP1L2	ACTGCGATTGAGTATTCCAC
4775	NAP1L2	AAAAGGTGGCGATACTGATG
4776	NAP1L2	ACATAACCGATAAGTCCTTT
4777	NAP1L2	GCTTAACTTTAATATCTGTC
4778	NAP1L3	TGAAACCGCCTATCATACAG
4779	NAP1L3	CCCGTTGGGAACAAATTTCG
4780	NAP1L3	TCTCGGAACCTGTCGCCCAT
4781	NAP1L3	GCTAGCTCGACTAGTGATTC
4782	NAP1L3	CGAGCTAGCCATCTCCTCTT
4783	NAP1L3	CAGTAGCAGTTGTGCGACTC
4784	NAP1L4	CACTTACGTTTCGATGTAGC
4785	NAP1L4	GATGCAGAATCCTCGAGTTC
4786	NAP1L4	CCGTCACAGTCCACAATCTC
4787	NAP1L4	TGAGTTGGTAAAGTAGTCGT
4788	NAP1L4	GCTAAAAATGCAAGTAACAC
4789	NAP1L4	TGTAAAGCTGCCAGAACTCG
4790	NAP1L5	ACTCGGCATTTCACCGAATT
4791	NAP1L5	GCTCTTGGATCTTGGCGAGT
4792	NAP1L5	CTCCTCTAACATGGCCGACT
4793	NAP1L5	TGCACACCCCTCCATCTCGC
4794	NAP1L5	GGCGTGGGCATCGTCATGTT
4795	NAP1L5	GGTGACCCTGACAGCGCGGC
4796	NASP	ACAAAAGACTAGCTGCTTCC
4797	NASP	AATCACTTCTGGAGTTGGCA
4798	NASP	CTTTGTAGAGGTAAGAAGTA
4799	NASP	AGCAGCTGTCAATGCATTCC

4800	NASP	TGATTCAGTCCTTGAAAACA
4801	NASP	AGAAACTGAAGGCTCAGAAG
4802	NAT9	GTAAGACAGCATCGCGAGAA
4803	NAT9	GTTCTCGCGATGCTGTCTTA
4804	NAT9	CATGACCTCGATCTCCCCCA
4805	NAT9	AGATCTAGAAGACCTCACCT
4806	NAT9	CTGCGAGGAGTGACCACGCT
4807	NAT9	CCTCTGATTTCATCCACTCG
4808	NBEAL1	AAGTGTCCAACCACAGCTTC
4809	NBEAL1	CTTGACAAGCAAAATTGACA
4810	NBEAL1	AGGGTAGATGATATGCCTCC
4811	NBEAL1	ACTTTGAAAAGCTGCCTACC
4812	NBEAL1	TCACAGAAATCTATCAAATG
4813	NBEAL1	CTGAATATAGAGTGTTGTCA
4814	NBN	ACGCCAGTCAAAAGTCTGTA
4815	NBN	CCTCTTGATGAACCATCTAT
4816	NBN	CATCCCCCGACTTCAAAGTT
4817	NBN	CAGTTAACACAGCATGATTT
4818	NBN	AGTAAAAATGTTGATCTGTC
4819	NBN	TTAAAAGATAATTCTAAGTA
4820	NBPF1	TCGCGTAACTTCCCATTCAC
4821	NBPF1	ACCGCGCTCGCTCAGAAGCT
4822	NBPF1	TGATGTAGAAACTTCCCCAT
4823	NBPF1	GCACACAGTGCTTAAGCTCC
4824	NBPF1	GCTCATCAGGGAGATGCCGA
4825	NBPF1	ATCCTCCCATTCAACATGAG
4826	NCAM2	TTGCTTGTCAGTAGCGGGCA
4827	NCAM2	TACTGACAAGCAACCCCAGC
4828	NCAM2	CAAGCTTTCCTCCAAGTCTT
4829	NCAM2	GCAATACAGAACTCACTGTC
4830	NCAM2	TATTGATGTTGAGAATCTGC
4831	NCAM2	AACATCAATAAAAGTGATGA
4832	NCBP2	CAATACGGCCGTGGGCGATC
4833	NCBP2	AGTCGCTGCGCAGCGCCTTC
4834	NCBP2	AGTACCGGGACCAGCACTTC
4835	NCBP2	CCCAGTCTGTGCGAATGATT
4836	NCBP2	TAAAGGTTCGGGATGAGTAT
4837	NCBP2	CAGGACTACGATGCTGGGAG
4838	NCKAP5	TGGCCCCCTCACTTGTTCTT
4839	NCKAP5	GCATCTGCTGACTCAGCTTG
4840	NCKAP5	TGAGAAGCTGATACATGAAC
4841	NCKAP5	TACGTCTTCAAAGCGAGAAG
4842	NCKAP5	AGTTGCCCAAAGAACAAGTG
4843	NCKAP5	AGCAACACAGGAGTCTCTGG
4844	NCOA4	TAGCTGTCCCTTTCAGCGAA
4845	NCOA4	GATTAACAGAAGTCAGCATC
4846	NCOA4	CAGTTGAATCTTCAGGCTTA
4847	NCOA4	CTGTATCTCCATGCCAGAGC
4848	NCOA4	GTGCATCACTACACCTCAAA

4849	NCOA4	GCATGAGCCATCAAGTGCTC
4850	NDRG2	GATCCTTACCTACCACGATG
4851	NDRG2	GGTAAGGATCGCTGGGCGTT
4852	NDRG2	GTTAGCTGCCCGAATCCTCC
4853	NDRG2	CCAGCCACTGTTTCAGTTCG
4854	NDRG2	ATGTCCTCGAACTGAAACAG
4855	NDRG2	CCTCTCACTTTAGGTACTGC
4856	NEB	TTTATGAGACTACGACAACA
4857	NEB	TTACGAAGAGGTGCCGGGAG
4858	NEB	TTTCTGACTGTGTGCAATGT
4859	NEB	ACAGTACTACACAGAAGAAG
4860	NEB	GGCAGATGACGAAGACTATG
4861	NEB	TTGAAGGATCCACTTTCTTC
4862	NEIL3	ATGGAGAGAAGATTCGCGCG
4863	NEIL3	CAGAGCGCTTCCCCGCACGC
4864	NEIL3	TTACAGTGGCGTGGAAACTT
4865	NEIL3	TTATCTTACCGTAAAGCTTT
4866	NEIL3	TTGACTCATCAGTAGAACTC
4867	NEIL3	CTTGAGTATAAATATAAAAA
4868	NEK11	TGGAGTTTCTCGACTTCTAA
4869	NEK11	TTCATTTCAGGGCCGAGATC
4870	NEK11	CCGACTTTGTGTCATAGCCT
4871	NEK11	GAGTTGACTACATGCATGAG
4872	NEK11	CTTGCTCCACAAAACTTGCA
4873	NEK11	TGATCTCAGAAAGGTACTTA
4874	NEK2	ACATCGTTCGTTACTATGAT
4875	NEK2	TTTCAGATCCCGATGCAATA
4876	NEK2	TGGTCATACCGTATTGCATC
4877	NEK2	CTGTCTCTGGCAAGTAATCC
4878	NEK2	ATATTGTGAAGGAGGGGATC
4879	NEK2	CTGGATTACTTGCCAGAGAC
4880	NELFB	GCAGCTCATCGAATACCGAC
4881	NELFB	AGTGCACATTCCGCGTGCGC
4882	NELFB	GCTGTATCGAGCCTGCGCCG
4883	NELFB	AGCCTTCCCCTCCGAAGCGA
4884	NELFB	GAACGTGAAGCTGTACGACA
4885	NELFB	ACTCCTGAAGCAGTACATCC
4886	NELFCD	ATCTGAAAACTACACCGCTG
4887	NELFCD	CCATCTTCAACACTCTGAAG
4888	NELFCD	CTGTGAACCTGCTGGCCGAG
4889	NELFCD	TTCAGGTGTTGAGCCAGTGC
4890	NELFCD	CTGCAAATCTAACTTCATCA
4891	NELFCD	ACAGTTTCCTGAACCTGCAC
4892	NELFE	CAAGCGTTCTCGAACCCTTG
4893	NELFE	AGCAGATCAGGCCGTTGCTG
4894	NELFE	CACAGCGTTTGACACCACCT
4895	NELFE	CTCAGCCTTGATGGCACTGA
4896	NELFE	AGTCATCCAGACGTCCCCAG
4897	NELFE	TCTCATACAGAGATTTCCTC

4898	NF1	TCTTTAGTCGCATTTCTACC
4899	NF1	ACACTGGAAAAATGTCTTGC
4900	NF1	TTTCTTCACACCTGTCGTGA
4901	NF1	AGTCAGTACTGAGCACAACA
4902	NF1	GTTGTGCTCAGTACTGACTT
4903	NF1	TCTCTCTCAGTTGATTATAT
4904	NFE2	TGTCCCGGACTAGCGCTAGC
4905	NFE2	GAGAAGCCGCTCCCGTTCAT
4906	NFE2	ACTCACCTGCAGCTCGGTGA
4907	NFE2	CATGTCCATCACCGAGCTGC
4908	NFE2	CCGACGACGGGGCAAAAACA
4909	NFE2	GATAATCCTGAGTCGGATTC
4910	NFE2L2	TAGTTGTAACTGAGCGAAAA
4911	NFE2L2	TGCATACCGTCTAAATCAAC
4912	NFE2L2	TATTTGACTTCAGTCAGCGA
4913	NFE2L2	TGATTTAGACGGTATGCAAC
4914	NFE2L2	ACAGCTCATCATGATGGACT
4915	NFE2L2	CCCGTTTGTAGATGACAATG
4916	NFKBIZ	GGGCCCGATTCGTTGTCTGA
4917	NFKBIZ	TCTGTGGAGAATACTGGTAC
4918	NFKBIZ	AGGTCCATCAGACAACGAAT
4919	NFKBIZ	CTTTACCCGAACACCTTGAA
4920	NFKBIZ	CCAATGAACACCACACAGTT
4921	NFKBIZ	AGCCCCATATGGGGGTTGGC
4922	NFRKB	AGACGGACACTTTAACCCCG
4923	NFRKB	GAAGGGCGGGGCCACTCCGC
4924	NFRKB	GTCCGCCACTCCCGCTCCTC
4925	NFRKB	GTGACTTGAAGCATAACTGC
4926	NFRKB	ACAAACCTCGGAAAAGCTTC
4927	NFRKB	ATGTTGTCAGCCTCTCAACA
4928	NFX1	CTGTGAATTGGTTCGTGTCA
4929	NFX1	AATTGACCTGCTTGAGATGC
4930	NFX1	GCAAGGTAAAGAATCCTGAG
4931	NFX1	AATGAAATTCCACATAGCTG
4932	NFX1	ATGACAAAAACATGTGAATG
4933	NFX1	TCTTTCAGTCTCTGCCATCC
4934	NHEJ1	TGAGCAGCGTCCTTCAACAA
4935	NHEJ1	AGTGGGGCTACGCTGATTCG
4936	NHEJ1	CCTAGAGATCCAAGACTACC
4937	NHEJ1	CATGCTAGCTAGTCCTTCCC
4938	NHEJ1	ATCCTGCAGATTCATGACAA
4939	NHEJ1	CTTGATGCTTCTGTCCCACT
4940	NHLH1	CTCGCGTGGCGTGGGCCGTG
4941	NHLH1	CACGCGAGAACGCATCCGCG
4942	NHLH1	GAGTTCAGACGTCCAGCACG
4943	NHLH1	ACTTGGCTGTGGCGCGGCGC
4944	NHLH1	CTCCTACCTGAACCACGTGC
4945	NHLH1	CGCCACAGCCAAGTACCGCA
4946	NKTR	GGTTGATCTCGATGTCGAAG

4947	NKTR	CTCGGTCGCGATGGGGGCGC
4948	NKTR	ACTTACCTGAGCACAAGCAA
4949	NKTR	AACTTCCTTTGCTTGTGCTC
4950	NKTR	GGTTAAAAACTTTATGATTC
4951	NKTR	GGGAAGAAGTTATGTTATAA
4952	NKX2-5	CTACGCGCCTGCCTACGGCG
4953	NKX2-5	CGCGCCGCCGTAACCCGGAT
4954	NKX2-5	AAATGCTGAGTGAGACGTGT
4955	NKX2-5	GCTGTAGGCACGTGGATAGA
4956	NKX2-5	AGTCAAAGACATCCTAAACC
4957	NKX2-5	CTCAGCATTTGTAGAAAGTC
4958	NKX2-8	GTTCGCGACGGGCAGCCGTG
4959	NKX2-8	TGCCCGTCGCGAACAAGCAC
4960	NKX2-8	ACGCGCAACACCTGCCGAGG
4961	NKX2-8	CCTTCTAGATTTACCCGAGC
4962	NKX2-8	GCCCGCCAGACTCGTCGCAG
4963	NKX2-8	GTCCGCTAGGCCCGCGTCTC
4964	NLRP5	TGAAATCGAGAATGCCAACG
4965	NLRP5	CATCCCGTGCCTTCTCCGAG
4966	NLRP5	TGGTCAGTCATCGTTGCTTC
4967	NLRP5	GAAGCAACGATGACTGACCA
4968	NLRP5	GCTGCAGAGACAAAAGAACA
4969	NLRP5	TTCTTCTGTCTCTGCTGCTG
4970	NMD3	ATACAGAACGATGGAGTATA
4971	NMD3	GTCGCACTCACATGTGTCCA
4972	NMD3	TTCAGTGTACAGTTCCCTGT
4973	NMD3	TGTAATTGTATGTGTTACTA
4974	NMD3	AAGAAAAGTACCTTGCATAT
4975	NMD3	AAAACAACATGCTCAGAAGA
4976	NME1	ATCTGGTTTGATCGCAATGA
4977	NME1	TTCATTGCGATCAAACCAGA
4978	NME1	CAAAGAATGGACGGTCCTTC
4979	NME1	CCACCCAGGAACATTATACA
4980	NME1	GGCTTCCGAAGATCTTCTCA
4981	NME1	AAGACGGGCCGAGTCATGCT
4982	NME3	CTGCGTGAACGCCCGTTCTA
4983	NME3	GAACGGGCGTTCACGCAGCT
4984	NME3	GAAGGTGCGTTCGTGTGCGC
4985	NME3	CCCGCCGCGAGATCGCTCTC
4986	NME3	CCGAGTCGCTGCCGTGAATC
4987	NME3	TGGACGTGGTGCGCACCTCG
4988	NME4	ACAATGTCGTCCGCGCCTCG
4989	NME4	GAGCCGCCGTTGCACGCCAT
4990	NME4	CATGATTGGACACACCGACT
4991	NME4	CAGAGCTCATGTAGCGGATG
4992	NME4	TGCTCGGCAAGGACGCTCTC
4993	NME4	CATCCACGCCAGCGACTCCG
4994	NME8	AACTTATAGAAAACATGACC
4995	NME8	AAAACGAGAAGTCCAGTTAC

4996	NME8	GCAAAGACAAGTAGTATTAT
4997	NME8	ACAACACTAAAGAGAAAAAC
4998	NME8	AGAAGCACAAGCACTGTGCA
4999	NME8	AGATGAAGACTTCAAAATAC
5000	NOD2	GATCACCCAAGGCTTCAGCC
5001	NOD2	TTCAACAACAAATTGACTGA
5002	NOD2	TGGGTGATCACCAGAGCTTG
5003	NOD2	CAATGAGCTTGCAGATGCCT
5004	NOD2	AGAAGTTCTGCCTGCATGCA
5005	NOD2	GATAACAATATCTCAGACCG
5006	NOL12	GCGGAAGGTCGAGCGAAAGA
5007	NOL12	TTCGCTCGACCTTCCGCTTG
5008	NOL12	CGTGACCACCATCAGTGACC
5009	NOL12	CCAGCATCTTCAAGTATTCC
5010	NOL12	CCGTCTTTGCTGTCACCAAC
5011	NOL12	AGACTCACCGCTGAGAGAGC
5012	NOS1	ACCGAGTCTTCAATGACCTA
5013	NOS1	GACATCATTCTTGCGGTCAA
5014	NOS1	CGTCTCTTCAAGCGCAAAGT
5015	NOS1	CTCGGTCGTTCTCCACGCCG
5016	NOS1	CCTCCACCTTAAGAGCACAT
5017	NOS1	CAAGTGTCCACGCTTCCTCA
5018	NPC1L1	CTTCATCAATGTGACCCGCG
5019	NPC1L1	AGGTCGCCACGTCGTCACCT
5020	NPC1L1	ACATGCCAGCCATCCGACGC
5021	NPC1L1	AAATGCGAGCCTTCCAGCGT
5022	NPC1L1	CGTAGTCAGCCATGCAGCTC
5023	NPC1L1	TAGTGCCCCGCTCACCTTCA
5024	NPEPL1	AGCCACGCAGACCATCGCCT
5025	NPEPL1	GCTGCAGCTTCCCGCGGACG
5026	NPEPL1	CTTCAGAGCCGCAATCAAGC
5027	NPEPL1	CCGCAGTCTCGCTTCATCCC
5028	NPEPL1	CGGCAGGAAGATGGCGAACG
5029	NPEPL1	TCGTCTTCAGTTCCTCATCC
5030	NPFFR2	AAGCTGCGACAGATATTCCC
5031	NPFFR2	GCCAACTAGTAAATCACTTA
5032	NPFFR2	ATAAAACCAGTCCAGTCTAC
5033	NPFFR2	CATTAAGATGCTCCTGATTG
5034	NPFFR2	ATGAAGATTGCTGCCACTTG
5035	NPFFR2	AACACGATGTGCAAGATCAG
5036	NPM1	ATTTACAGGTTGTGAACTAA
5037	NPM1	GTACCGTTCTTAAAGATAAC
5038	NPM1	ACAAGAATCCTTCAAGAAAC
5039	NPM1	GGAACCTTGCTACCACCTCC
5040	NPM1	TGCAGAGTCAGAAGATGAAG
5041	NPM1	TGATGACGATGATGATGAAG
5042	NPM2	ACCCCGCTCAGATTTGCTTG
5043	NPM2	CTGACTTACCATAACGTTCC
5044	NPM2	TAGCCACGCTCGCCTGCTTC

5045	NPM2	CAAAGAGGAGATGCATCGCG
5046	NPM2	TGAGGATGCAGATATATCTC
5047	NPM2	AGAAAGCTGCACTCCTACCA
5048	NPM3	TACCACATTACACTCGTCTT
5049	NPM3	GGCCCGGAACCATGACCATC
5050	NPM3	GGATGATGCGGAGCACGTGC
5051	NPM3	CCGTAGGCCCCGACACCCC
5052	NPM3	GGGCCTACGGGTCCCGGCCC
5053	NPM3	ATCCTCTTCCTCTACCTTAA
5054	NPNT	TTCGATCTCATGTATATTGG
5055	NPNT	GCCATACTGACAGTTTGCCA
5056	NPNT	GTTCATGCACCTGTGCTTAC
5057	NPNT	AGCCCCGGCCCTGTAAGCAC
5058	NPNT	GAGCCTCCTGCCCTAGATTT
5059	NPNT	TATGGCTGTGATGTTGTTAA
5060	NPR2	AGAAGCGCATTGAGCTGACC
5061	NPR2	GTCACTGAATACTGTCCTCG
5062	NPR2	TGTCTTAGGTGTAACTGGGC
5063	NPR2	AACCATCTCACTCGCTTCAT
5064	NPR2	TGAGTTCAAACAGAACCTGC
5065	NPR2	CCTGAAAGTCCCCAGAATCC
5066	NPR3	CATGTAGAGCCAAGACGTAG
5067	NPR3	GTGCGAGCAGTGACACCATC
5068	NPR3	TGCCATCCTCCTCTACGTCT
5069	NPR3	GCATCATGCTGGTGGCGCAC
5070	NPR3	TCCTCAGTAGAGTGACTGTC
5071	NPR3	TCCAGACAGTCACTCTACTG
5072	NPW	GGGACGCTGTCAGAACGGTC
5073	NPW	CACGTGCTTGTACCACGCGC
5074	NPW	GGATCCCCGTCCGTGCGCCC
5075	NPW	TTTGCTGCGGGGTCCACCGC
5076	NPW	TGCGTCGCTCACCCTATCTG
5077	NPW	GACCCCGCAGCAAACCGCCT
5078	NRAS	TACAAAACAAGCCCACGAAC
5079	NRAS	GAATGGAATCCCGTAACTCT
5080	NRAS	TTGTATGGGATTGCCATGTG
5081	NRAS	CCATGAGAGACCAATACATG
5082	NRAS	CTTCGCCTGTCCTCATGTAT
5083	NRAS	ATTCATCTACAAAGTGGTTC
5084	NSA2	TCTCAGCATGACGCTGTTTA
5085	NSA2	GAATGATGAAAAGACACCAC
5086	NSA2	TGAAGTAAATGTGAGCGAAT
5087	NSA2	ATCCCCACTGTATACAACTT
5088	NSA2	AAAGTGTGCTTTGTTGGAGA
5089	NSA2	GAAGAAGGCATGGAAGAGAA
5090	NSUN5	GCAGCTTCTTCTCCGCACGG
5091	NSUN5	CAGCGCCGGCCTCCTCCGTG
5092	NSUN5	GAAATAATCAACTACATCAT
5093	NSUN5	GTTGATTATTTCAAGAGACA

5094	NSUN5	CGATGACTTACGAGCCCTCA
5095	NSUN5	CTACCCTGTCCTGCAGAATG
5096	NT5C	AAGCCGCGGCGTTGCTCCAG
5097	NT5C	GCGAGCAGTACCGCGCCCTG
5098	NT5C	ATAATTCGTTCTACGAACTG
5099	NT5C	GCGGGAGATGAACGACCTAC
5100	NT5C	GCCCGGGGCTTCGTACACAC
5101	NT5C	TTCTACACAGTACCGCTGGG
5102	NT5C3A	TCACACTTACTATGACATGT
5103	NT5C3A	TATCATTGACAACTGTAAGC
5104	NT5C3A	AGGAGGAGCTGCCAAACTTC
5105	NT5C3A	ATAATTTCTTCTACTCTTGT
5106	NT5C3A	TACAGATGAATGTAGAAAAA
5107	NT5C3A	AGAGTAGAAGAAATTATCTG
5108	NUDT11	ACGTTCTGTGCTTGCGATCC
5109	NUDT11	GTGCTTCCGGAGCGAACGCG
5110	NUDT11	CCTCGTCCTCGCGTTCGCTC
5111	NUDT11	CCGCTTCTTCGTACACCTCT
5112	NUDT11	GTACGAAGAAGCGGGAGTCA
5113	NUDT11	AGTGCAAACCCAACCAGACG
5114	NUDT21	CCAGCCGGTCTGCGAGCGAT
5115	NUDT21	CCGCCCAATCGCTCGCAGAC
5116	NUDT21	AAAAGAGCCCCTCTACGAGA
5117	NUDT21	TTCTGATTGTACATGAGCAC
5118	NUDT21	CCTGGTGGTGAACTTAACCC
5119	NUDT21	ACTAAAACGCTTAATGACAG
5120	NUDT5	GAAACAGTTCCGACCACCAA
5121	NUDT5	CCATTCTGAGAAGATTCCGT
5122	NUDT5	AATCAGTGAAACGTACAACC
5123	NUDT5	GCTTGAAAAAACAACGTACA
5124	NUDT5	ATCCGCAGTCTGCTCTTTCC
5125	NUDT5	TCTATGCAGTAGCCCCCCAT
5126	NUDT9	ACCAGTCCAGTCCGTCCTGC
5127	NUDT9	AACGTCATGTCTGGTTCTAA
5128	NUDT9	GTGGCAAGACTATAAGCCTG
5129	NUDT9	TGGTTCTTACCTGGTTATAA
5130	NUDT9	TTCTCCCAAGTTTAACGAAA
5131	NUDT9	AGATTGAAAATGGAAGACCG
5132	NUF2	TAGATCATGTGCAAGACTTC
5133	NUF2	ATTCGCAATAAGATCTTAAC
5134	NUF2	AATCTCAGCTACATTATATC
5135	NUF2	TGAGTAACTAAATTGCTGAA
5136	NUF2	AGAGCCTTACAAATAGTATA
5137	NUF2	AAGAAGCCTTCCATTAAATG
5138	NYX	CGGCACGTCGGTGACACGTC
5139	NYX	CAACCTGTTCCGCCGCGTGC
5140	NYX	ACTGGGTGGATGAAAGGCCG
5141	NYX	ATGTTGGTCCTGCTTCTGCA
5142	NYX	CGGTCTCCATCGACCTGGAC

5143	NYX	CTCGGTGCGCTGCGACCGCG
5144	OAZ2	CACCCACTGTCGAAGATCCC
5145	OAZ2	GATGTTCATGGTTTATCCCC
5146	OAZ2	TACTGGAAGTGGACGATGTG
5147	OAZ2	CTATGACTATTAGTCCTCAT
5148	OAZ2	CATCGTCCACTTCCAGTATG
5149	OAZ2	GGATCTTCGACAGTGGGTGA
5150	OGG1	GTGTACTAGCGGATCAAGTA
5151	OGG1	CGCACGGCCTCCAGCTCGTC
5152	OGG1	GCTACGAGAGTCCTCATATG
5153	OGG1	GGGCACTGGCACTCACGTAA
5154	OGG1	TGAGCCGAGGTCCAAAAGCC
5155	OGG1	GGCCATCAAATGCATTGCCA
5156	OIP5	AGTTACAAATAACGTCGTTT
5157	OIP5	CCCTTCCTAGTTGGCATTGA
5158	OIP5	CATCTCTGATGCATTTACTA
5159	OIP5	CATAGTAAATGCATCAGAGA
5160	OIP5	CTGCCTTTCCAGTGACAAAA
5161	OIP5	GTATTCTACCCATGCTGCCC
5162	OIT3	ACTCACGACCACAGTAGACG
5163	OIT3	GCACAATGCCGTCGCCTTCT
5164	OIT3	AGCGAGTGCACATGCGCTCC
5165	OIT3	TGGAGCGCATGTGCACTCGC
5166	OIT3	AGACTCACCCACGGGTGACA
5167	OIT3	CTCACCTGCCTCTTCATCAC
5168	OPRD1	GCACACGGCCGAGTAGAGCG
5169	OPRD1	CGCCATCACCGCGCTCTACT
5170	OPRD1	GGACTTCCGCACGCCTGCCA
5171	OPRD1	ACCGCCTGCACCCCGTCCGA
5172	OPRD1	GCTGCACCTGTGCATCGCGC
5173	OPRD1	AGGCGTTGGCGTGCCCATCA
5174	OR12D2	TCCATGAAGCTCTCTAACGC
5175	OR12D2	GTCCATGTTGTTCGCCGTGA
5176	OR12D2	CCGTAGAGTAACAGATATCC
5177	OR12D2	GGAACACTGAGCTTAATCAG
5178	OR12D2	TGTCACGGGGACAATTGCCA
5179	OR12D2	ACCAATGACCCAGATTGTGA
5180	OR2H2	CATTTGGGGAACACAACTCG
5181	OR2H2	GGTTAACCAAAGCTCCACAC
5182	OR2H2	TTAACTCTGCAAAAGGGCGG
5183	OR2H2	ACCTGCCCTTCTGCCCCGAT
5184	OR2H2	CACCTCCTACAATGAGATCC
5185	OR2H2	TAATGGCTCCGTAAGAGACA
5186	OR4C6	GTGTGCTGCCTAATGGTAGG
5187	OR4C6	GAGGGTGTCTACAATCACCT
5188	OR4C6	TATCTTAATTGCGTCCTACA
5189	OR4C6	CCCAAGCCCCTCCTACCATT
5190	OR4C6	CTCATCTGTCGTTGCCCCCA
5191	OR4C6	CTCTTAGTTACCCTCAACAG

5192	OR4K2	GACCACAGAATGGTAACGTG
5193	OR4K2	TGCTTCTGTCATTAGTCCCC
5194	OR4K2	AGTTATGCATTCAATGAGTC
5195	OR4K2	CACTTGTTGAGATCATAAAG
5196	OR4K2	AATGATGCTTCACAGTAACC
5197	OR4K2	ATGCATAACTCCCATAATCC
5198	OR8H2	TATAGACTCCTTTGTCAACG
5199	OR8H2	GGTGTTGTATGTATCAGTGC
5200	OR8H2	CTGGGCAAAGCAGCCCGTAA
5201	OR8H2	ACATTCAGCAGTACCCAAGA
5202	OR8H2	CAGAAAGTGTCAGTCCCATA
5203	OR8H2	AACCAACAATAATGAATATC
5204	ORC4	CAATGACAAAATCGCCCTAA
5205	ORC4	AATCACAAGGCAGTTAAATC
5206	ORC4	AATTCATTAAGTGTATCTGC
5207	ORC4	TTCTCAGCAAAAACCTTGTC
5208	ORC4	TGAAAACCTTTCATTTCTTC
5209	ORC4	AAGCTTCCAGAAGAAATGAA
5210	ORC6	CGGCCTCTCCGCACGCACCA
5211	ORC6	TCTCCGTGGTGCGTGCGGAG
5212	ORC6	AACAAAATGGTAGCCACATC
5213	ORC6	TACAGCTAAACTGTACAGCT
5214	ORC6	CAAGATCCACTTGCTGTGTC
5215	ORC6	TGTAAACAACTAGAGAAGAT
5216	ORMDL1	ATACCCGTGTCATGAACAGC
5217	ORMDL1	ATCCGAACACCATGTAGTTG
5218	ORMDL1	AAGCAAGGCTCCTAACTCAT
5219	ORMDL1	ACCTTTCGAAACTCCTGACC
5220	ORMDL1	TTTGTTAAAGTCCAAGCAAC
5221	ORMDL1	GTGTTTAGGATGAAGTGAGT
5222	OSGEP	ATGCAATGCAGTCGATATCC
5223	OSGEP	CGACTGCATTGCATACACCA
5224	OSGEP	TGTAGGTAATGTGAGGCTAC
5225	OSGEP	GTGATTCTGGGGTTACACAG
5226	OSGEP	AGATGTTTCGGGCTGGACAC
5227	OSGEP	TCCATCCCCTTTACAGTGTA
5228	OSM	AAAGAGTACCGCGTGCTCCT
5229	OSM	GCCGCCATGCTCGCCATGCT
5230	OSM	CTTGCGCTGAAAAGCATCCG
5231	OSM	GGCCGAACATCCTCGGGCTC
5232	OSM	TGGGGGTACTGCTCACACAG
5233	OSM	CCACTGAGTGCATGAAGCGA
5234	OTOA	GTACCTTCTGGACGCCAGCC
5235	OTOA	CTGCATCCGACAAAGAACCC
5236	OTOA	TCTTTGTCGGATGCAGTTGT
5237	OTOA	GGTGGCTGCACTTGATCATC
5238	OTOA	GAGGGATTCCAGTTGGCTGT
5239	OTOA	GATGGCTCCGTGCACAGCCT
5240	OTUD7A	CGACTATGAGCAGCTCCGCC

5241	OTUD7A	CTGTGTGCACGAGCTGTAAA
5242	OTUD7A	ATTTCAGGAGCTTCATCGAG
5243	OTUD7A	AGACCTGAGCGTGTACAGCG
5244	OTUD7A	GGGTCGCTCCACCTTGTGCC
5245	OTUD7A	TTTACAGCTCGTGCACACAG
5246	OXLD1	ACTTGCCGAGCCACGGACCG
5247	OXLD1	ATTCGGGACAGACCACGTAG
5248	OXLD1	TGGTGCCGTCCGCACCTGCT
5249	OXLD1	AGCCGCCGCGGTCCGTGGCT
5250	OXLD1	CCCGGAGCGCAAGCCCCTGA
5251	OXLD1	GGCTCCCAAGCAGGTGCGGA
5252	PADI4	TGGTAGCACAGGCGACCAGA
5253	PADI4	GTCACTTACCCACCCCGGTG
5254	PADI4	GCAATATCCACGACCACCCC
5255	PADI4	CTAGCCCGACGATGGCCCAG
5256	PADI4	AAAGCTCTACTCTACCTCAC
5257	PADI4	TGACTCAGCTTGACATCTGC
5258	PAF1	ACCTACCGCATCGACCCCAA
5259	PAF1	ACACACCATTGGGGTCGATG
5260	PAF1	AGTAAACCCCGTCACCCTCT
5261	PAF1	CGGGGTTTACTACAATGAGT
5262	PAF1	ACCCACTCACCTCTTGGAGC
5263	PAF1	TCCAGCTGATGAGAAACTTT
5264	PAK1	TATTCCTGCAGTTACCTCGT
5265	PAK1	GAAAAACCCGCAGGCTGTTC
5266	PAK1	CAGAAATACATGAGCTTTAC
5267	PAK1	AATTGCAGCTGTGTGCCGTG
5268	PAK1	TGCTGTCACAGGGGAGTTTA
5269	PAK1	TTACAATTCTTCTAATGCCT
5270	PAK2	AGTAATCGAGCCCACTGTTC
5271	PAK2	GTTAGGGCATGCCAGAACAG
5272	PAK2	TTTGAGCACACCATCCATGT
5273	PAK2	ATGGTGTGCTCAAAATCAGA
5274	PAK2	CTTACTGCTGGTGTTCCAGA
5275	PAK2	GAGAAAGATGGCTTTCCTTC
5276	PAK3	CTTTGAGCATACGATTCATG
5277	PAK3	AGTAATCGTGCCCATTGCTC
5278	PAK3	ATCGTATGCTCAAAGTCTGA
5279	PAK3	AGAACATCTAGAACAGCTTG
5280	PAK3	ATACATAGCAGCCCATCCTT
5281	PAK3	TTTGCAGATAAAAGTGCACA
5282	PAK4	GGCGATGCACACGATGCCCG
5283	PAK4	GGCCCTTTAACACCTACCCG
5284	PAK4	TGCTCATGGGATACTCGCTG
5285	PAK4	ACCTTTGCTGCCCCGCACGA
5286	PAK4	CTGCACAGGTGGTAATCATG
5287	PAK4	TCTCCACCACATTCTCGTGC
5288	PAK6	CGCCCCCCACGGGCACCAAT
5289	PAK6	CTACCAGCACTTCAACGTGG

5290	PAK6	CTGGTATCCGAAGTGGTCAG
5291	PAK6	TTCGGAATAGCCTGCGCTTC
5292	PAK6	CAGCGGGATCCCCCCGCACC
5293	PAK6	CAGACATCGTCTCCCAAGTC
5294	PAM16	CCACTAGGTGGTCCGCGCAA
5295	PAM16	GAGCCGAGCAGTCACGTATG
5296	PAM16	ACCGGTGTCCAGCGCGTCCT
5297	PAM16	GCACGCCCATCACAATGATC
5298	PAM16	ACCTGCAAACTCCTGCCGCA
5299	PAM16	CGGGCCGCAGCTGATGCCCG
5300	PANK1	ATTGGTGTATTTCGAGCCGA
5301	PANK1	CGGGGCTTTCAAATTCGAAG
5302	PANK1	GGAGGAGACTATGAACGATT
5303	PANK1	GGCCTATGTTGCTTGCTGAC
5304	PANK1	CAGAGCTTTCAGTTGTCCTT
5305	PANK1	GAAGCTGCTGGCATATGCCA
5306	PARD6G	GCTGCAATCGTAGAATCGCA
5307	PARD6G	ACCTGGTCCCCGAGACGCAC
5308	PARD6G	GGGAGTCTAGAGCGTGACCG
5309	PARD6G	AGTTTGGGGCGGAATTCCGA
5310	PARD6G	ATTCTACGATTGCAGCGCAG
5311	PARD6G	TCTCTGGACCGTCATAAGCC
5312	PARP6	ATATGGAACTATCGATGACG
5313	PARP6	AGGACATCAAAGGCCAGTTC
5314	PARP6	CGAGGAAGCTGATGTTGATG
5315	PARP6	AGTCGTCATCATTCCAGAAC
5316	PARP6	GGAGGGAGATAATGAATCAG
5317	PBRM1	TAATACCATCCGAGACTATA
5318	PBRM1	CAAACTCATTTCTTGTTCGA
5319	PBRM1	GAAACCACTTCATAATAGTC
5320	PBRM1	ATAAAGCCGCTTGCAAACTC
5321	PBRM1	CAATGCAAAGTCCTATTATA
5322	PBRM1	CCTGTTCATCCTTATAGTCT
5323	PBX4	CGCATGGTCGGCGCCATTCA
5324	PBX4	CATCGAGCAGCCGCGAACGC
5325	PBX4	GCATTGAGCACTCTGACTAC
5326	PBX4	TATCTAGTGGTAAGCATCCG
5327	PBX4	AGCATGCTCTGAATTGCCAT
5328	PBX4	GGGCGGCCTCACCATCTCCC
5329	PCBP4	GCGAGACTGTAAAGCGAATC
5330	PCBP4	CTCTCGGATCTCCTTGATCT
5331	PCBP4	ATGACAAGGCGCAGGGTCAC
5332	PCBP4	CAGGAAGTGGGCAGCATCAT
5333	PCBP4	AATCAGTGAGCCACACTGAC
5334	PCBP4	CTTTGTGCTGCTCCTGCAAA
5335	PCNA	CGAAGATAACGCGGATACCT
5336	PCNA	TGCTTCAAATACTAGCGCCA
5337	PCNA	ATACGTGCAAATTCACCAGA
5338	PCNA	CATAGTCTGAAACTTTCTCC

5339	PCNA	GACAAGTAATGTCGATAAAG
5340	PCNA	AGTGTCACCGTTGAAGAGAG
5341	PCSK5	GTAGACGCGCGTCCGACACA
5342	PCSK5	GTTGATGAATCCGTACTTGC
5343	PCSK5	AGGTCAGTTATCTCGAGCAG
5344	PCSK5	GCTTACCATATACCACATGC
5345	PCSK5	AGAAGTGGTAGTAGTCCTTC
5346	PCSK5	CCTATTTCAATGATCCCAAG
5347	PDCD5	CAGTATCTTAGCCCAAGTTC
5348	PDCD5	TTCTCCTAGGTATCAGAACA
5349	PDCD5	AAGATGCTTACACCTGGCCC
5350	PDCD5	TTTCCAGGATCCTGGTGATG
5351	PDCD5	ACCTGTGCTTTGCTTCCTGT
5352	PDCD5	CATATCTTGCCATCTGTATA
5353	PDCL	CAAGGGGCCCCAATTCAAGC
5354	PDCL	CACCCCCCTTATAGATCAGC
5355	PDCL	CACCCTTGATGATAAGTTGC
5356	PDCL	GAAGGCATCTCAGTTAACAC
5357	PDCL	CAGTCATTGATCACACCTTT
5358	PDCL	AGAAGCTGTCAATGACTTGC
5359	PDE2A	TACCCCTCGTTCCCTGCCCG
5360	PDE2A	CACCATGGACGTGTCATCCT
5361	PDE2A	CTCCTCCAGGTTCACCGACG
5362	PDE2A	AAAGAACCTCTTCACCCACC
5363	PDE2A	GAAACTCAAGTGTGAGTGCC
5364	PDE2A	ACTCACCCAGGTGGGTGAAG
5365	PDE4B	GAGTTTCTCGACATCGCCTT
5366	PDE4B	CGACTATGACTTGTCACCAA
5367	PDE4B	ACAGTTGTAATAGCAATGCT
5368	PDE4B	TGCTATTACAACTGTAAGCC
5369	PDE4B	CCTCTATAATGAAGAAAAGC
5370	PDE4B	GGCGTGAAGTACCAGCCCAG
5371	PDE6G	ACTCAGCCTTGGGCGGTTCC
5372	PDE6G	AATTTAAGCAGCGACAGACC
5373	PDE6G	AGGTTTGGGGACGACATCCC
5374	PDE6G	CGTGCTAGATGATGCCATAT
5375	PDE6G	ACATCACAGTCATCTGCCCT
5376	PDE6G	GTTCCCAGGCCTTCCATTCC
5377	PDGFB	GAGTCGGCATGAATCGCTGC
5378	PDGFB	CCTCGGCGCTGACCAGACGC
5379	PDGFB	GGTCATGTTCAGGTCCAACT
5380	PDGFB	GCAGGGGGACCCCATTCCCG
5381	PDGFB	GATCTCCAACGCCTGCTGCA
5382	PDGFB	AACATGACCCGCTCCCACTC
5383	PDGFD	CTTTGCGCAACGCCAACCTC
5384	PDGFD	GTCTCTTACCATCTCGCCTG
5385	PDGFD	CAAGTCCGATGACTACTTTG
5386	PDGFD	CGTAGCCGTTTCCTTTCACC
5387	PDGFD	GACATGGCGGCTTCACTCTC

5388	PDGFD	ACACCATCGTCCTCTAATAA
5389	PDGFRA	AAAGCCCTGTCTGCTGTCGT
5390	PDGFRA	TCGGGATCAGTTGTGCGACA
5391	PDGFRA	CTTAAAACCGTGTATAAGTC
5392	PDGFRA	GACCTTCAATGGACTTACCC
5393	PDGFRA	AGCTATGGGGACTTCCCATC
5394	PDGFRA	GTCTTAGGCTGTCTTCTCAC
5395	PDGFRB	GTAGAGCCGTTTCCGCTCAT
5396	PDGFRB	GGTCCCCGAGTCTTCTAACT
5397	PDGFRB	ATGAGGTGGTCAACTTCGAG
5398	PDGFRB	TGGAGACCGATGAGCGGAAA
5399	PDGFRB	CACACTCTCCGTCACATTGC
5400	PDGFRB	ACCCTCATGTGCATTGTGAT
5401	PDGFRL	GTGGAGCTACCCTGCGTATC
5402	PDGFRL	ACTGTAGAGCTTCGATGTAA
5403	PDGFRL	TCTGCTGGTGCACGAAGCGC
5404	PDGFRL	GCAACCTCATTCCGAGCACC
5405	PDGFRL	CGATGTTGTCTACTTGAACC
5406	PDGFRL	GTCAAGCAGAATGAGCGCTA
5407	PDHA1	TGTGCGTCCGAGAGGCAACA
5408	PDHA1	GATGCAGACTGTACGCCGAA
5409	PDHA1	TACCTTCCCAGATCTACAAT
5410	PDHA1	AGGATGGGCTCAAATACTAC
5411	PDHA1	ACTCTCACTCTCTTAAAACC
5412	PDHA1	TGATGCTACATTTGAAATTA
5413	PDHA2	ATGTAATGACGTGATCCGAG
5414	PDHA2	CGTGTACGATAACTGACTCC
5415	PDHA2	CAATGGCATCGTCGGTGCAC
5416	PDHA2	GATGCTGACTGTTCGCCGCA
5417	PDHA2	ACGGACTTAAACTTGATCCA
5418	PDHA2	CCTCAACACGCGGGAGATGA
5419	PDHB	ACCTGGATTGTTATCCCGAA
5420	PDHB	CCTTGTATGCCCCATCATAC
5421	PDHB	AAGGCCACCAGACATGTAGT
5422	PDHB	GACTGACCACCTTTAAGCCT
5423	PDHB	TGGCAGCTGAGTTTATAACC
5424	PDHB	AGTTCGTGATGCTATAAATC
5425	PDIA2	GGGGGATCTGTCGCGCTTCC
5426	PDIA2	GGCTGTTGAACTCCGTGACC
5427	PDIA2	TTGCGGAAGAACTTGAGCGT
5428	PDIA2	ACGCTCAAGTTCTTCCGCAA
5429	PDIA2	GATCGGTGGCCGGGACCTAG
5430	PDIA2	CCGCGGCTCTTTCAGCAGTT
5431	PDIK1L	TAAGATCTCGGTGGATGATC
5432	PDIK1L	TCCCGTATTAGATCGTACTT
5433	PDIK1L	AACTAGCCCTTCGTGAGTTC
5434	PDIK1L	CCAGAAGCGCCTATTATTTG
5435	PDIK1L	ACACAACACCGTAACTACCT
5436	PDIK1L	AGCCAAAGTACGATCTAATA

5437	PDK1	CCGTTCAATTGGTACAAAGC
5438	PDK1	GGTGTCCTGAGAAGATTATC
5439	PDK1	CCATCTCATCGAAAACACAT
5440	PDK1	ACTTCAAGTACATTGCAGTT
5441	PDK1	TAGATGGCTATGAAAATGCT
5442	PDK1	TAGTTCTTCAAGTTCTAGTT
5443	PDLIM7	TGTGACTTCAAGATCGACGC
5444	PDLIM7	GGATGCGGAAGGAACGCGAC
5445	PDLIM7	GGTTCAGAGCAAACCGCAGA
5446	PDLIM7	TCTCACCGCACAGACGAAGC
5447	PDLIM7	CAGACAGCCGCTCCGACCGC
5448	PDLIM7	CAAAGCGGCGCAGGCCGGAG
5449	PECAM1	GCTCTAACTAATGAGTTCCG
5450	PECAM1	CTCACTAAAGCAACGACTGT
5451	PECAM1	TACAGAGTATCTGCTTTCCA
5452	PECAM1	TCAGTCGTGCTGCATAAGTA
5453	PECAM1	TTGTCGCTACAGAGAACGGA
5454	PECAM1	GTATATGACCTACCTTCCTC
5455	PELI3	TCACCCCGTTGGCGTGCGAC
5456	PELI3	TAGCGACACAGACATGTTCC
5457	PELI3	GTGCATCACCAGGACTCCAT
5458	PELI3	TCAGTGCCAGGCGGCTTCGC
5459	PELI3	CCCAGGAGCGAGCGGCCAAA
5460	PELI3	TATACTCCACTATGACCGAG
5461	PEPD	TCCCCAGCGTGGCGTCAACA
5462	PEPD	TCACTTGCTGATGCCGTCAA
5463	PEPD	AGCCACTTACCAAAGTGAGG
5464	PEPD	GCGAACATGGCGGCGGCCAC
5465	PEPD	GCCCTCTGTCCTCCTCACTT
5466	PEPD	CAGCGGCAGTGTCTGCAGGG
5467	PEX12	GCCCACTGTGTCGTAAAACC
5468	PEX12	GCTAGGTCGACTGACAGTTC
5469	PEX12	GAGACCCGCTCTTCAGCATG
5470	PEX12	CTCTGTAAAATCGTTTCCAG
5471	PEX12	ATCAGATGTTGTACTTCTGT
5472	PEX12	CTTGACCACATGCTGAAGAG
5473	PFKL	ACTTACCAGGATCCGGTCGA
5474	PFKL	GACGCGCATGGGCATTTATG
5475	PFKL	GGACGCCCTCTGCCTTCGAC
5476	PFKL	TTGGCCTTACCTCGTAGATG
5477	PFKL	TGTTCTCACCTCCCTCCACG
5478	PFKL	CGTCTCCAACATCATCCAGC
5479	PFKM	TTCAGTCTCGCCAGTTAGTC
5480	PFKM	ATGAAACTCGTCTTTATGCA
5481	PFKM	CTTGAAGAGTCTAGATACTA
5482	PFKM	GAGGATCTTACCCACAGTGG
5483	PFKM	TGGCTTTGGTATGAGCATGC
5484	PFKM	GACATTTGTGTTAGAAGTAA
5485	PFKP	GAACGCTGCCGTCCGTGCCG

5486	PFKP	GTAGATACCCATGCGCACCA
5487	PFKP	TCCCAGGGCGGGACGATCAT
5488	PFKP	CGTCCCCGCCGATCACACAC
5489	PFKP	CGGAGGCTCAAACATCGCAG
5490	PFKP	CCCCTAGGGCTACCAGGGCA
5491	PGF	CGTGTCCGAGTACCCCAGCG
5492	PGF	AGCACGTTCGCTGCGAATGC
5493	PGF	CAGCTCCTAAAGATCCGTTC
5494	PGF	GACGGCCAATGTCACCATGC
5495	PGF	CGAGCCGTTCCCAGCAGACA
5496	PGF	TAGCAGTGGGCCTTGTCTGC
5497	PGK1	GCTCATAAGGACTACCGACT
5498	PGK1	GCACACCATCAGGCCGGCCT
5499	PGK1	ACCTCTGGTTGTTTGTTATC
5500	PGK1	CAGGGATGTTCTGTTCTTGA
5501	PGK1	GACATAGACATCCCCTAGCT
5502	PGK1	CTTCTTCCTCCACATGAAAG
5503	PGK2	ACACCGCGCTCATAGTTCCA
5504	PGK2	CAAGTTTGACGAGAACGCTC
5505	PGK2	AATCTGCCCCATAAAGCATC
5506	PGK2	CATGATATCTTTAACGATCT
5507	PGK2	TGCATCACTGTTATAGGGGG
5508	PGK2	CTCAAGCAAGGCTAATTGTT
5509	PGM1	CTTCTCCTAGATCGGTCGCT
5510	PGM1	ATCCGTATTGATGCTATGCA
5511	PGM1	CCTGACCTGAAAGTAGACCT
5512	PGM1	CTCCATGCATAGCATCAATA
5513	PGM1	CTGACAGCCAGTCACAACCC
5514	PGM1	CTTCAATTGTCTTGCTGATT
5515	PGM2	TCGCCTAGACCGCTGCCTTC
5516	PGM2	GCGGTCTAGGCGAGGACGCC
5517	PGM2	CTCTAGGTCTATTGGGATAA
5518	PGM2	ATCATTTCTCCTCACGATAA
5519	PGM2	GACCATCATCCAGACTACAC
5520	PGM2	GTGAAACGACTAATAGCAGA
5521	PGRMC2	GGTTGCGGGAGCCGTCGTAC
5522	PGRMC2	GAGCAGCTGCGCCAGTACGA
5523	PGRMC2	ATCCTGTTTATTGTGATCCT
5524	PGRMC2	AAATGTGGCCAGTCCTCTGG
5525	PGRMC2	GCTGGTAGGGATGCCTCCAG
5526	PGRMC2	TCTTCATCTGTATATTCTGA
5527	PHACTR2	GGTCAGCTCCAACGCTGACG
5528	PHACTR2	CCCGTCCGAGTCAGAGTCAT
5529	PHACTR2	AAACTCGAACAGACTGTCCC
5530	PHACTR2	TATCTTGTACACCGATGATG
5531	PHACTR2	CTTTGGCAAGTAAAATACGC
5532	PHACTR2	TGATAGCAAGAGTATCCCTC
5533	PHGDH	TGGACGAAGGCGCCCTGCTC
5534	PHGDH	GGCTGCACTGGACGTGTTTA

5535	PHGDH	TGGTGGCAGAGCGAACAATA
5536	PHGDH	GCTTCTGCCAGACCAATCCA
5537	PHGDH	TTGGGAGAGAGGTAGCTACC
5538	PHGDH	GCTCACCCTGTGTTATCACC
5539	PHLDB2	CCTGACAGTCGCTTATCTAC
5540	PHLDB2	CCAGTAGATAAGCGACTGTC
5541	PHLDB2	AGTCATTCTGAACAACCTCG
5542	PHLDB2	TGTTCTTCACTTATACCCTC
5543	PHLDB2	GGATATGGAATGTGCTCTTT
5544	PHLDB2	GAACTGGAAAAGAACATTGT
5545	PHOX2B	AGGTCCTTACCTGCGGCGTA
5546	PHOX2B	GCTTCCAGTATAACCCGATA
5547	PHOX2B	ACCTCCATCCCGGATTCGCT
5548	PHOX2B	CGAAGATAGGACGCTGGCGA
5549	PHOX2B	GCTGCTTGCGCTTCTCGTTG
5550	PHOX2B	CTCCTCCCGAGTGTAGATGT
5551	PI15	TTACGTCAAACTACCTGTAC
5552	PI15	GTGGCGACGTGCAGTTTACT
5553	PI15	CAAAATCTATCTGTACGCAC
5554	PI15	CAGTGCGTACAGATAGATTT
5555	PI15	AATTGCGCATCCTATCCGAT
5556	PI15	CATGTGCACACATTATACGC
5557	PIAS1	ACGCAAGATGGCGGACAGTG
5558	PIAS1	CTTAAAGCTCTAGAATGATC
5559	PIAS1	CCTCTGAAGAAGAACTGTTG
5560	PIAS1	CTGATTTGCTGCACTTGTTG
5561	PIAS1	TGTCAAGGCAAATGCAAAAC
5562	PIAS1	CCTCAACAGTTCTTCTTCAG
5563	PICALM	TGATATACCAGACCTTTCAC
5564	PICALM	TGGTCTATCTCTGACCAAAG
5565	PICALM	GGCACGTTTGAAAGCTTTAA
5566	PICALM	CAGCAAGTTGGAATTGACAG
5567	PICALM	ACCAATTATTCAGAATCAGA
5568	PICALM	TCCATCTGATTCTGAATAAT
5569	PIF1	TCTCGTCAATGACCAACCGC
5570	PIF1	ATATCTGCTAAAGCGAATCC
5571	PIF1	GGTCATTGACGAGATCTCAA
5572	PIF1	GTGCTGAGGGCCGTCCTGAA
5573	PIF1	GGTGGTGCCCCCGATGTGGC
5574	PIF1	GGGATCCAGCTCATCATCTG
5575	PIGO	CCAACACAACCCGCGAAAAT
5576	PIGO	CACCACCATGCAGCGCCTCA
5577	PIGO	GGCCAAGAAACTTAGCCAGA
5578	PIGO	AGGAAGGCGTGTAGTCTTCA
5579	PIGO	ACCAGGGAAAAGGTCTTTCC
5580	PIGO	CGTGCTGATTGCTCACTTCC
5581	PIH1D1	GCTGTTGACAGCTACGTCGT
5582	PIH1D1	TCCTCCCGCCGACGTGACCG
5583	PIH1D1	AGCTCGTGATCACCATCGCC

5584	PIH1D1	TCGATTCTGGTCTGGTTGTC
5585	PIH1D1	CCCAGAACAGCGATTTCTTG
5586	PIH1D1	CTGTCAACAGCGACTTCTAC
5587	PIK3C2A	CCATCACACGTAAAAGTAAC
5588	PIK3C2A	ACCAATCACCGCACAAACCC
5589	PIK3C2A	TTTGAAGAGCCAGTTCTACT
5590	PIK3C2A	TTATCACAACCAAGTAGAAC
5591	PIK3C2A	TGTCCCCCTAAAAGTCACAA
5592	PIK3C2A	GACAGCATTAGAACTGAAGA
5593	PIK3CA	TACACAGACACTCTAGTATC
5594	PIK3CA	GAAGATTTGCTGAACCCTAT
5595	PIK3CA	GCTTCAGCAATTACTTGTTC
5596	PIK3CA	CGAGCAGCACGAGGAAGATC
5597	PIK3CA	TGTTATAGGGCAAATAATAG
5598	PIK3CA	TTACCTCTTTAGCACCCTTT
5599	PIK3CB	CTTCCCGAGGTACCTCCAAC
5600	PIK3CB	AATCCACCGCCCAGATGTCA
5601	PIK3CB	GTGACAAGAAGTTGTGACCC
5602	PIK3CB	CTTGTCACTAATTTGAGAAC
5603	PIK3CB	GATTATGTGTTGCAAGTCAG
5604	PIK3CB	CATACCTGGAACTGAATTAG
5605	PIK3CD	GCGCGAAAGTCGTTCACTTC
5606	PIK3CD	GCCGGAAGACTACACGCTGC
5607	PIK3CD	GTCCACCAAGGACGTGCCGC
5608	PIK3CD	CGGCCGCCTCCTCGCAGAAT
5609	PIK3CD	CAGAGCTACCTTTGCCGATG
5610	PIK3CD	GAGGGGTCAACCCACTGTGC
5611	PIK3CG	TTGGGTAAAGTCGTGCAGCA
5612	PIK3CG	AAGTATGACGTCAGTTCCCA
5613	PIK3CG	GCCAGATTTCTGCTGAAGCG
5614	PIK3CG	TTTAGAGTTCCATATGATCC
5615	PIK3CG	ATATGGAACTCTAAAGCTTT
5616	PIK3CG	ACCACGCTTCAGCAGAAATC
5617	PIK3R1	AGCGTAAGCCAATACTGATG
5618	PIK3R1	AATGATCGATGTGCACGTTT
5619	PIK3R1	CGCTTTCAAACGCTATCTCC
5620	PIK3R1	TCCAGTATCCAAATACCAAC
5621	PIK3R1	CTAGCATACCTCATCAGTAT
5622	PIK3R1	ACTGAGCTAGAGATTCATTC
5623	PIK3R2	GCGCTCTGTACCCGTTCCGC
5624	PIK3R2	CCCACTGATCCACGTCGCTC
5625	PIK3R2	GCTGCCGTGTGCGCTCGTTG
5626	PIK3R2	AGACTGGTCCCTGAGCGACG
5627	PIK3R2	GCAGTTCTCCCCACCTGATG
5628	PIK3R2	GAACTTACCTGTCCTTTCAA
5629	PIK3R3	TATTGAGCGATTTCGCAGAG
5630	PIK3R3	GGCAGATGGCTCAATCACAA
5631	PIK3R3	GGTACTGGGGGGATATTTCA
5632	PIK3R3	GAACTGTCCTTCATTCCATT

5633	PIK3R3	TAATTCCCAGCCAGACATTC
5634	PIK3R3	TGTAGGAAATACAGATGAAG
5635	PIK3R4	ATCGTTAGACTAGCCTATGC
5636	PIK3R4	CACAAATCTGGAGTTCGTCA
5637	PIK3R4	TAACGATAGTAGCATCATCT
5638	PIK3R4	CAAAAACCTTCACAACGACC
5639	PIK3R4	CCAAATGGAAATTATGACAC
5640	PIK3R4	CCTGTGTCATAATTTCCATT
5641	PIK3R5	GGTCGTAGGTGCCCTTCTCC
5642	PIK3R5	GATCCTGCAGAAGACCCGAG
5643	PIK3R5	CTACGACCTGCTCACCCCGC
5644	PIK3R5	CAGGCCAGGTCAGGAACCGG
5645	PIK3R5	CTTGAGTTCAGCATCAATGA
5646	PIK3R5	TCACCTCTCGGGTCTTCTGC
5647	PIP5KL1	GGACCTCCGAGAAATCGTCC
5648	PIP5KL1	AGCCTTCCAACGTCTCCACG
5649	PIP5KL1	GGCCTCCTACCTCGTGAACC
5650	PIP5KL1	CGGAGGAACCAGCTCCGCTG
5651	PIP5KL1	CCGAGACAAGCAGTCTCGCC
5652	PIP5KL1	GCCTGTTTGAGATCAGCCCG
5653	PITX3	ACACGGGTCCCGATAGACGT
5654	PITX3	CCCCACGTTGACCGAGTTGA
5655	PITX3	GCATACCCGCACGCGGGCCT
5656	PITX3	CGTGTGGACCAACCTCACCG
5657	PITX3	GCCCTGTCGCTGTCAGACGC
5658	PITX3	TGCCAGCGTCTGACAGCGAC
5659	PIWIL1	GGCCTAGGCTGAATATAACC
5660	PIWIL1	GCTGCGCTGTCTCCTGACCG
5661	PIWIL1	GGGAATTATTTGGCCGTGGA
5662	PIWIL1	GAGTTATCGTTAGCAGAGAG
5663	PIWIL1	CCTGAAATCCAGCAGATATC
5664	PIWIL1	CGGTCAGGAGACAGCGCAGC
5665	PKHD1	CTGGTCCGGCATGTCACCAC
5666	PKHD1	CCACCATGTTCACGTTCACC
5667	PKHD1	TGATCCACGTTCCCCCTGCA
5668	PKHD1	GAGTATTGAAGTACTACTTT
5669	PKHD1	AAGTGGACAGAATGACTGCC
5670	PKHD1	CAGGTTCAATATGTAAACTC
5671	PKHD1L1	TATCAACTCCATAGTTAAAC
5672	PKHD1L1	CTACATCACAAGTAATTGAC
5673	PKHD1L1	TGTGCCGCGGATCCCAGCAC
5674	PKHD1L1	GTTACCTGTGCTGGGATCCG
5675	PKHD1L1	CCATTTATACTGCCATATTT
5676	PKHD1L1	GCAACAAGGCTGACTATAAG
5677	PKLR	CGGCACGACCCGGACAATAT
5678	PKLR	AGTGGAGAACGGCGGCGTCC
5679	PKLR	CCAGCGACGTGGCTGCCGTC
5680	PKLR	GGCCGCATCTACATTGACGA
5681	PKLR	GAGTCGCGCAATGTTCATCC

5682	PKLR	AGGGCCAGCATCTCGCTCCG
5683	PKM	ATCACTGCCTTCAGCCCGAG
5684	PKM	CAGCCACGTACCAACATTCA
5685	PKM	GACGAGCTGTCTGGGGATTC
5686	PKM	GGCTGTGCGCATGCAGCACC
5687	PKM	CATCAGGTTTGATGAAATCC
5688	PKM	CCTGGAGCACATGTGCCGCC
5689	PKMYT1	CAAGGACCGGGCCCGCAAGT
5690	PKMYT1	AACGCTTTACCGCATAGAGC
5691	PKMYT1	CGCCCCGGAATGACACCCGC
5692	PKMYT1	CACCTTGAAGACCTCTCCGT
5693	PKMYT1	CAACTTGCGGGCCCGGTCCT
5694	PKMYT1	TCCCTTCCAGGTGCGCTCCA
5695	PKP2	GCGAGCAGTGAGTATGCTCG
5696	PKP2	TCCTACCTTTAGCATGTCAT
5697	PKP2	GCTTGAGGATGCCACGAAGC
5698	PKP2	CCCCAGGTTAACCAGCTTCG
5699	PKP2	TCTTCCGAGCTTCAGATTTC
5700	PKP2	TAAAACCTATGACATGCTAA
5701	PKP4	GGGGCAACCACAGACCCGCC
5702	PKP4	CTGTAGACTTGCCAAAAACG
5703	PKP4	CACTATTCTAGCATCCGTGA
5704	PKP4	TCATTCTAGGTGTGTAGGTT
5705	PKP4	CCACTGACCTGACTGAATGA
5706	PKP4	TCTCGCTCCAATGTCGACAC
5707	PLA2G12B	GAATAGGACTCCTCCGTGTC
5708	PLA2G12B	CAAAGCCCAGACTCCGCTTA
5709	PLA2G12B	GCTGTGATGCAAAATTCCGA
5710	PLA2G12B	TGCCCGCTGACTATTCATAA
5711	PLA2G12B	AAATAGGAGCCGCAGCCATT
5712	PLA2G12B	AGCTTCCCCGGAGGTGCCGA
5713	PLA2G2A	TCACCGATCCGTTGCATCCT
5714	PLA2G2A	TGACTGTTGCTACAAACGTC
5715	PLA2G2A	AAGGAAGCCGCACTCAGTTA
5716	PLA2G2A	CATGAAGACCCTCCTACTGT
5717	PLA2G2A	TCAACTGTGTGAGTGTGATA
5718	PLA2G2A	CGTCTTGTTTCTAGCAAAAC
5719	PLA2G2F	GGTACTCCTCTCGGTACGTC
5720	PLA2G2F	CCAAGGCTGTCACCCCTATG
5721	PLA2G2F	CATGAACCAGACGTACCGAG
5722	PLA2G2F	GTAGCAACCGTAGCCCACGA
5723	PLA2G2F	GATGGCGCTCCTCCCTGTGA
5724	PLA2G2F	TATCTCAGTGTTGTTCTCGA
5725	PLA2G4D	GACAGCTGTAGGATCACGTA
5726	PLA2G4D	ACTGGTGTCGGTGAGCGTCT
5727	PLA2G4D	GGAGCTTAGCATCTATGATG
5728	PLA2G4D	TTACTCACGTTTCTTCCATC
5729	PLA2G4D	GACATCTCAGAAGTCCTCCC
5730	PLA2G4D	AGGGGGAGGCCTCTACCTGC

5731	PLA2G4E	GGATGACCCTCACTGTCAAC
5732	PLA2G4E	TCATCCGGATGAAAAATGTC
5733	PLA2G4E	GCACCCACCTGCGGGTTGAG
5734	PLA2G4E	GAAGTTTCCACTCAACCCGC
5735	PLA2G4E	GTCTCACCTTCACTCGGCTC
5736	PLA2G4E	CCAGATCCAGAGCCGAGTGA
5737	PLEKHB2	ACTGCATCAACATCCGCACG
5738	PLEKHB2	CTCCAGATTGTTTGTCGAGA
5739	PLEKHB2	CCTCGATATTCTGCCGAGTC
5740	PLEKHB2	GGAAACCACGGATGTCTCAT
5741	PLEKHB2	CATGACCGATGAGACATCCG
5742	PLEKHB2	TTTCATAGATACTCAGCCCC
5743	PLEKHM3	GATCGTGATCGAGCATGCCC
5744	PLEKHM3	TCCACTGTTGAGCTACAACG
5745	PLEKHM3	ATTCTGCCGCCCCATGTAAC
5746	PLEKHM3	ACAGGCTGCCAGCGATCCAT
5747	PLEKHM3	CAGCACGCATAGTCCACAAC
5748	PLEKHM3	ACCTCATGCCCCACCAGTTC
5749	PLEKHN1	AAAAGTTGTGCATTACGCCA
5750	PLEKHN1	GTCCCCGCTTCGAGCAGCCT
5751	PLEKHN1	TCACCGTGCCCGGGATGTAG
5752	PLEKHN1	TCACCTGTTTCCCTTCAGCG
5753	PLEKHN1	GCCCCTTCTTGAACACACTC
5754	PLEKHN1	GAACAGCCACTGTGTCCCTC
5755	PLEKHS1	AACCTACCATATCCGTCCTG
5756	PLEKHS1	GCAAGATAGTTGATGACATC
5757	PLEKHS1	TTCTCAGCTGTTCTCCTCTG
5758	PLEKHS1	ACAGAGGAGAACAGCTGAGA
5759	PLEKHS1	TATCCCCGATGAAAGCCAAG
5760	PLEKHS1	CTTGCTCTCACAGAAGCCAC
5761	PLG	GATTTCTTACCTTCTTCGGA
5762	PLG	CAGATGCCGATAAAGGCCCC
5763	PLG	GCGGCCCCTTCATTTGATTG
5764	PLG	AAACATACTTCTCCAAGCAG
5765	PLG	AGGTTTGGAATGCACTTCTG
5766	PLG	TGTTCTAAGACTGACTTGCC
5767	PLLP	GTCCGCGTGCTAACTTTCGA
5768	PLLP	TCCCGTCGAAAGTTAGCACG
5769	PLLP	CCGGCCTTATAACCAGCGCG
5770	PLLP	TCACCGAGGCAGCCGCGCGC
5771	PLLP	GAGTGCCTTCTTCAGCTACC
5772	PLLP	CTTTCAGTTCTTTGCGTGTT
5773	PLOD2	CTATGCTGATCAAGATGATC
5774	PLOD2	TGTTTATAGGTCCTTGGTCA
5775	PLOD2	CGTTTCCCAATGTGCACAAC
5776	PLOD2	CTGCTGCAAAGACCACTTTG
5777	PLOD2	ATATTTCAATTATACTGTGA
5778	PLOD2	CACAGTATAATTGAAATATT
5779	PLSCR3	CCAGGATGTAGCGCCGGCTC

5780	PLSCR3	TGGGCCGCATCAGCAAGCAG
5781	PLSCR3	CCGGAGCCGGCGCTACATCC
5782	PLSCR3	CCTACAGTTCCCGCTGGACC
5783	PLSCR3	TTTCCACTCGCTCAGCCTTC
5784	PLSCR3	ACAGGCTACTTGCCCCCCAA
5785	PML	GCGGTACCAGCGCGACTACG
5786	PML	CTGCGCGTGAACCGCGCCAA
5787	PML	CTCACCAGGTCAACGTCAAT
5788	PML	GTCGGTGTACCGGCAGATTG
5789	PML	ACCGCACCCCTACGCTGACC
5790	PML	GGACCCTATTGACGTTGACC
5791	PMS2	CGACTGATGTTTGATCACAA
5792	PMS2	CAAAAGTTTCAACCTGAGTT
5793	PMS2	TTCCAACCTTCGCCGATGCG
5794	PMS2	CTTATTGAAGTTTCAGACAA
5795	PMS2	CTCTAGTACAGAACCTGCTA
5796	PMS2	CCCAAACTTACCAATATTAG
5797	PNP	GGGGGAGCAACGTGAGCTAC
5798	PNP	CCCAACTTTCCCCGAAGTAC
5799	PNP	CGTGACCATATCAACCTACC
5800	PNP	CCATGTCTGATGCCTACGAC
5801	PNP	AGTGGTCAGAACCCTCTCAG
5802	PNP	CTTGCCAGTACCTGTACTTC
5803	PODNL1	CTTGGACCTTCGAGTGTTCC
5804	PODNL1	AATGAGCTGTCCCGCCTCAG
5805	PODNL1	CTGCCCCCGAGTCGACACTG
5806	PODNL1	TGTGGAGGTTGAGGGTTCGC
5807	PODNL1	TTCAATGGGGAGGCCCACCC
5808	PODNL1	GAGGCGGGACAGCTCATTGT
5809	PODXL	TAGATGAGTCCGTAGTAGTC
5810	PODXL	GATGCGTCGAAGTGGGTTGT
5811	PODXL	CAGCTCGTCCTGAACCTCAC
5812	PODXL	GGTGTTCTCAATGCCGTTGC
5813	PODXL	TCGTGGGCTGCACTGTCTCC
5814	PODXL	CACAGAGGGTGTTTCCTGTG
5815	POLA1	TGGTAGGTCGAGGACTTCAC
5816	POLA1	AGGCACGCCAGGATGATGAC
5817	POLA1	GCCCTTGATGCTGATGAGAA
5818	POLA1	TGCAGATGGTATTGGCTATG
5819	POLA1	AGCCCTAGAAAGACTGAAAA
5820	POLA1	CGAGAAAAAAAATCAAAGAA
5821	POLA2	CTCTTACACCACACCTTCAA
5822	POLA2	GTCCTTGCAGGTACTATGCC
5823	POLA2	GCAAAAGATTATCGAAAGCC
5824	POLA2	CAGAAACCCCCCTAACAAAA
5825	POLA2	CACCGTCAAGTTTCTCTCCA
5826	POLA2	TTTACTTACCTTTGAAGGTG
5827	POLB	CACTTCAGAATCAACCAAAC
5828	POLB	ACCTGTAAGCATTGTACTTG

5829	POLB	GAATACATTGCTACAGTCTG
5830	POLB	GAGAACATCCATGTCACCAC
5831	POLB	TCCACAAGTACAATGCTTAC
5832	POLB	TACCCACACAAAATAAAGAG
5833	POLD1	CCGGATCTCAAAGTCGACGT
5834	POLD1	GCCCAGCTTCGCGCCCTACG
5835	POLD1	AGGTTTCGGGCCCGAGCACA
5836	POLD1	CCAAGTTCAGCTCCCGTTGC
5837	POLD1	AGAAGTAGGGAGCGAAGCCG
5838	POLD1	GGCCACTCACCAGGGGGCGC
5839	POLD3	ACTAGGGGTTCATGTTAACC
5840	POLD3	TCATAGTCAGTATTGAACAG
5841	POLD3	GCTGGCAGTCACAGCTAGCT
5842	POLD3	ATTGGACTTACTGTTTGGCC
5843	POLD3	CTCTCACTACTGCAACCTTG
5844	POLD3	AGTAGTGAGAGAAGATAAAT
5845	POLD4	AATCAGTGATGAGCCGCTTC
5846	POLD4	ATTCCTACCCGGTTGTGAAG
5847	POLD4	TGGCATCTCTATCCCCTATG
5848	POLD4	GGATCACACGGCTGCAGCGC
5849	POLD4	GCTCCGCTTCCTCCTCGTCG
5850	POLD4	GGAGCCCCAGCCCCGCGACG
5851	POLE2	TCGCAAATATGGCGCCGGAG
5852	POLE2	AAACATGATTGAACGATCTG
5853	POLE2	CAGCAAGCCCCGCAACTTGA
5854	POLE2	ATTAGAGCTTGAAGATAAAC
5855	POLE2	CAACAGACTGACTGCATTCC
5856	POLE2	TAGACTGAAGAGCTTCTGTG
5857	POLE3	TCGTGCTGTACGCCACATCC
5858	POLE3	GGCGTACAGCACGAAGACGC
5859	POLE3	CGTCCTTACAGCATATAGGC
5860	POLE3	TACCCCATTGAAAGAAGCTC
5861	POLE3	CACCGCCTCCTTGATGATCC
5862	POLE3	TGCCCAATGCCGTGATCACC
5863	POLE4	AGATCCCGACGTGACGCTAG
5864	POLE4	CTGTCCCGCTAGCGTCACGT
5865	POLE4	TGCCTACTGTTGCGCTCAGC
5866	POLE4	TCCCTGCTGAGCGCAACAGT
5867	POLE4	AGATAATGCAATAGAAGCTG
5868	POLE4	AAGGCCTTCACTCGCGCCAG
5869	POLH	CACAAGTTCGTGAGTCCCGT
5870	POLH	TCAGAGGGGATGCGAAAACA
5871	POLH	AGTTATGAAGCTCGTGCATT
5872	POLH	GTTGGGCTTGTTTAGTCCAC
5873	POLH	AAATGAGAGCAGCCATAGAG
5874	POLH	CAAGCCCAACCGCCAAACCC
5875	POLL	GGTTGTGATTGGTCGCCTTC
5876	POLL	CATGTTGTGCGCACTGGCAT
5877	POLL	TGGTTCATACCGACGGGGAA

5878	POLL	GAGCACGTCGACATCACCAC
5879	POLL	GTGCACAGACCCACTTATCC
5880	POLL	ACCACAATGTGAGTGACACC
5881	POLR1C	GTGGTTCACGTACAGTTCGT
5882	POLR1C	GTCCCTCTAGAATTTCCGTG
5883	POLR1C	CAGAATTCGTCGAAAAGCAT
5884	POLR1C	TGAGATTCTTGCTCACCGTC
5885	POLR1C	CCCCACTTACCTTCTCGAAG
5886	POLR1C	GAACTGTACGTGAACCACAA
5887	POLR1D	AAGCACAGCTTCCGCGCTCG
5888	POLR1D	GCGCGGAAGCTGTGCTTGTA
5889	POLR1D	AATTTACGCATTCAGACTCG
5890	POLR1D	TGTGACATTTGTATTGCACG
5891	POLR1D	CCCAGCGATGGAAGAGGATC
5892	POLR1D	GAAAACTAGAGCTGAAACAA
5893	POLR2A	CAATGAGCAGAACGGCGCAG
5894	POLR2A	TGGAGCGGGGCACGCCAACC
5895	POLR2A	TGCCCTTCAACCGCTGCTTC
5896	POLR2A	GCAAAAACATATGCGAGGGT
5897	POLR2A	ACATGTCTACGACCTTTGCA
5898	POLR2A	TGACTCACACGGGGCAAGCC
5899	POLR2C	TCAGGGTGGCCAATTCGATT
5900	POLR2C	CCTTTCTCAACTTGACGATG
5901	POLR2C	ATCGCTGAGGTTCCCATAAT
5902	POLR2C	ATCAATCTGAACCCAGTCAA
5903	POLR2C	ACGATCCAGACAATGCCCTG
5904	POLR2C	CCACAATGTCATCACTAATG
5905	POLR2D	TAGCACTCACCTACGAACAC
5906	POLR2D	AGACCATTGCCAGTGTTCGT
5907	POLR2D	TCCGCGGGCTGGCGACGTAG
5908	POLR2D	AGTCCAAGGCTCTAATCCCA
5909	POLR2D	TCCTCTACGTCGCCAGCCCG
5910	POLR2D	ACTGAAAGCTGCGCTTTGTC
5911	POLR2E	CATCAACATCACGGAGCACG
5912	POLR2E	CATGACACCCTCCGCCAAGC
5913	POLR2E	CATCACACGGGCTCTCATCG
5914	POLR2E	AATCCGCAAGACCATCATGC
5915	POLR2E	ACTTGGGGGCCATGTCGACC
5916	POLR2E	GGACGAGCTTGACCAGACCC
5917	POLR2F	CACGCGGGCTCGCTCGTACT
5918	POLR2F	GGAGGATCTCGACATTCTCC
5919	POLR2F	CAGTCTTCATAGCTCCCATC
5920	POLR2F	ATTCGCCGTTACCTGCCAGA
5921	POLR2F	CAGGATGTGTGCCCCTGTGA
5922	POLR2F	TGGCGACGACTTTGATGATG
5923	POLR2G	CTGCTGCACCCGCGCTACTT
5924	POLR2G	CGAAGTAGCGCGGGTGCAGC
5925	POLR2G	ACAATGGCCTTGTACTTAAC
5926	POLR2G	TGAGATCCGCTTAAAGATTG

5927	POLR2G	ACCAGCACCAATATTGTCAA
5928	POLR2G	GTTGGACTCTTCACAGAAAT
5929	POLR2H	CCTGGAAGGCCTATCATCAG
5930	POLR2H	AGCTCTGCGTACGTGTCCTA
5931	POLR2H	GCTAGTACCTTGTATGAAGA
5932	POLR2H	AGTCCACCTCGAATCCATGC
5933	POLR2H	CAATACTTACCCAAGTCTAC
5934	POLR2H	TCAAATTTACCCTGTAGACT
5935	POLR2I	CAACAAGATCACGCACGAAG
5936	POLR2I	GAACCGCATTCTGCTCTACG
5937	POLR2I	CGGCCCGCGCACTGTGTGAC
5938	POLR2I	CCCTCTCAGGTGCGGCCACA
5939	POLR2I	CCGGAACTGTGATTACCAGC
5940	POLR2I	TCACCGCGTAGAGCAGAATG
5941	POLR2J	GGTGGCCATAAAAGACAAGC
5942	POLR2J	CCCGAAAGCGCTCCTCCAGC
5943	POLR2J	CGAGTCGTTCTTGCTCTTCG
5944	POLR2J	GAAGAGCAAGAACGACTCGA
5945	POLR2J	TCCTAGGATCACCATTAACA
5946	POLR2J	TAATGATGTTTCCCAGTGTG
5947	POLR2K	TTGGAGGTTGAACGTCCTTC
5948	POLR2K	CCAATCAGATGCAGAGAATG
5949	POLR2K	CAACCAATGATATATATCTG
5950	POLR2K	CCACATTCTCTGCATCTGAT
5951	POLR2K	GCTAACAATGGACACCCAGA
5952	POLR2K	ACAGAATAATGTACAAGAAA
5953	POLR3C	GTTAGCAACTGCGCTCACAA
5954	POLR3C	TGCGAATGCTTAGATATCCC
5955	POLR3C	ATCTAAGCATTCGCAATACC
5956	POLR3C	TTGTGAGCGCAGTTGCTAAC
5957	POLR3C	ATCAAACACATTTGTGCGAC
5958	POLR3C	GTTCCTAAACTCAGCTTGAT
5959	POLR3GL	CAGATTGGCGGCGTCTACCC
5960	POLR3GL	CGATTGACAATGCCATCGAT
5961	POLR3GL	CCTACCAGGGTTCCAATCGA
5962	POLR3GL	AGGTACTGGGCGGAACTCCA
5963	POLR3GL	CCGAGTGCGGAAGCTACAGA
5964	POLR3GL	ATATTCCCCTTCCTCGCCTG
5965	POM121	ACTTGCGTAAGCGCCTGTCA
5966	POM121	ACTGAGTGCCCTCAAAGAGA
5967	POM121	TGGGGCGTTACTTGATGGTG
5968	POM121	GAACGCGAAAATCTTCTGTC
5969	POM121	TGTGTAGCCCAGTGACTGTG
5970	POM121	TACCTGTGAGATGCCTCGAG
5971	PON1	ACGACTTAATGCTCTCCGAG
5972	PON1	GTTCTACGGGTTGTACCTCT
5973	PON1	CATCTGGATGGTTCACCACC
5974	PON1	TGTCTATATAGCTGAGTTGC
5975	PON1	AGATAATGCCATGTACCTCC

5976	PON1	CTAATGAAAGCCAGTCCATT
5977	POU2F2	GACTCCCCATCAGAGCACAC
5978	POU2F2	TGCTCAGTTCCTGCTACCGC
5979	POU2F2	CCAGTTGGGGACACGGAGAA
5980	POU2F2	TGCGGTAGCAGGAACTGAGC
5981	POU2F2	CAGGTGCTTACCTTTGTACT
5982	POU2F2	GGAGTCCAGACCTTGCTTCT
5983	PPFIBP1	TAAAGAATCCCTCGTTCTTC
5984	PPFIBP1	AATCACTTGCCTGAAGAACG
5985	PPFIBP1	TATAATAGGAGCTTCTAAGT
5986	PPFIBP1	AAAAAGGCTACAAGAAAAAT
5987	PPFIBP1	ATCCAACTTCTGAGTTTCTA
5988	PPFIBP1	AAAAGAACAACTAGAAGAAA
5989	PPFIBP2	CTAGACGTGCCAAGCGTTCC
5990	PPFIBP2	AACGCTTGGCACGTCTAGAA
5991	PPFIBP2	GCCTCAAGTCCTCGATGAGA
5992	PPFIBP2	AGTGATGGTACTTGTGAGCC
5993	PPFIBP2	CTGCACTGCTAGACCACATT
5994	PPFIBP2	GAAAGCCATCAACACCAAAC
5995	PPP1R18	AGCCCAACCCGACGACGAAG
5996	PPP1R18	CCAATACCCCTCCGAGAGTT
5997	PPP1R18	ATGAATCGGTTCTGGTGCAC
5998	PPP1R18	GAAGCGGTACCCAACTGCCG
5999	PPP1R18	GGAAGATGGTCACCGCCGGC
6000	PPP1R18	CTCCCACAGATGAGTCCTGC
6001	PPP1R21	CGGTCTCACCCGGAAGTACA
6002	PPP1R21	TGAGACCGTCAACCACAGCT
6003	PPP1R21	TCAGCTTCGGGCTCAGAATC
6004	PPP1R21	AGCAAATTCTGCAGCTTTAA
6005	PPP1R21	AGAAGATAGAAGAGAATGAA
6006	PPP1R21	TGATGAGCAGCACAAGCATG
6007	PPP2R5A	TGATCAGAAATTCGTACAAC
6008	PPP2R5A	TTACGGATTCCAGTGTTCTT
6009	PPP2R5A	AACCCACGCTTGAGGCCTCT
6010	PPP2R5A	AGCGTGGGTTCATCCTCTTC
6011	PPP2R5A	TACAAACTGGCAAACATAAT
6012	PPP2R5A	TCAAAATCTGGATTATCACT
6013	PPT1	TTTGGACTCCCTCGATGCCC
6014	PPT1	GGCGTACTCCAAAGTTGTTC
6015	PPT1	GCCTCGTGCAAGCCGAATAC
6016	PPT1	GCCAGTATTCGGCTTGCACG
6017	PPT1	CTGTTGCAATCCCTTAAGCA
6018	PPT1	TGTCTCACCTCCGAATCTAC
6019	PRCC	TTGTAGCTGTAGTCGTCCCC
6020	PRCC	GGGCATTGGCTGCATCGTCC
6021	PRCC	TTTCCAGTTTAAGCGGCTGC
6022	PRCC	ATGACTTAGTCATCCATTGC
6023	PRCC	TTCCGCCGCTGCTGGCCTGT
6024	PRCC	GATGAATAAGATATGTGATC

6025	PRDM6	CGCAGGTTGCGACATGTGCG
6026	PRDM6	GGGAACCTAGTAAGTCGAGC
6027	PRDM6	AACACCGGCAGCTGCGACAC
6028	PRDM6	ATCTAACAGTAGTTCAGTAC
6029	PRDM6	CGTACGGTTGTTGGGGTCGC
6030	PRDM6	GCACTCGCTGCGCCGGCTTG
6031	PRDM9	CTCGCTATAGGAATGTGAAA
6032	PRDM9	TATAATGCACTGATTACTAT
6033	PRDM9	GAGCCCTGAAAAGTCCCAAG
6034	PRDM9	CAGAAGATTCTGATGAAGAA
6035	PRDM9	GTTCTCTCTGTGTCTTCTTC
6036	PRDM9	TCCCTCTTACCTTGCTGCCT
6037	PREPL	GACGTATATCGAGCCACTTT
6038	PREPL	GATATACGTCATGACAGCGA
6039	PREPL	ATTCGCTTCTGGATAAGTAC
6040	PREPL	CTTATCCAGAAGCGAATACA
6041	PREPL	TATTAGCATACTGCCATGTT
6042	PREPL	CCATATACATGTACCAAGAG
6043	PRIM1	TGACAGACTATGACGATGTG
6044	PRIM1	TTCCAACAGACCTGATAAAA
6045	PRIM1	GCGCCTATATCAATCTTGTA
6046	PRIM1	TCAACACAATACAGTGAAGC
6047	PRIM1	CTTCAACAACCAGAGTGATC
6048	PRIM1	CTTCACTGTATTGTGTTGAT
6049	PRKAA1	TACTCAATCGACAGAAGATT
6050	PRKAA1	TCCTGTTACAGATTGTATGC
6051	PRKAA1	GGGCCACAATCAAAGATATC
6052	PRKAA1	GTTGGCAAACATGAATTGAC
6053	PRKAA1	AGTAAAAACAGGCTCCACGA
6054	PRKAA1	TTTCAGAAATCTGATGTACC
6055	PRKAB1	CCCGTCCATCGAAACACCGT
6056	PRKAB1	CTCGGCCAACGGTGTTTCGA
6057	PRKAB1	CCTTGATAGACAGCGCGTAT
6058	PRKAB1	CCTATACGCGCTGTCTATCA
6059	PRKAB1	GTGGACGCACGACCCTTCCG
6060	PRKAB1	CCCATAGTAACCAGCCAGCT
6061	PRKAG1	GTGTACGAGCTGCCCCTTTA
6062	PRKAG1	CCGCTACTATAAATCAGCCT
6063	PRKAG1	CCAAGGCTGATTTATAGTAG
6064	PRKAG1	TGCTTCTTACTATCCCATAA
6065	PRKAG1	TTCAGATAGCTCCCCAGCTG
6066	PRKAG1	GATATTGATGAAATCAGTGA
6067	PRKCE	CGTCAGGCGCAGGGTCCATC
6068	PRKCE	TATGTGATCATCGATCTCTC
6069	PRKCE	GTAGGTGGGCTGCCGAAGAT
6070	PRKCE	AAGCAGGGATACCAGTGTCA
6071	PRKCE	ATCACATACACTCTTCCTTC
6072	PRKCE	AACGCATGCGGCCGAGGAAG
6073	PRKD1	CTTCAATAAGATCGCCTTCC

6074	PRKD1	CGACCAATAAACGACTCTGA
6075	PRKD1	CCTCACACCGCTGCAATTGT
6076	PRKD1	TTGTCATCCACTCCTACACC
6077	PRKD1	GAAAGCGGCCAGTGATATCC
6078	PRKD1	CATACCAGAAGGGGCTCATC
6079	PRLH	TTGCCCTCCTCCGACCGAAG
6080	PRLH	GAGCTGCAAGTCGTACCCAT
6081	PRLH	TACGACTTGCAGCTCCCCGC
6082	PRLH	ACGACATAGCACCGCCTTCC
6083	PRLH	CACAGGCCTGATCCCGCGAC
6084	PRLH	AGCAGGTCAGCCGGGGTCGC
6085	PRRT2	CGCCACGATGCTTAAGAGCT
6086	PRRT2	ACCGCCTAAGTTGATGACGC
6087	PRRT2	GGCCTTCGCTTATGCTGTCA
6088	PRRT2	CACCATGACAGCATAAGCGA
6089	PRRT2	TCTCCCACAGTGTATAAGTG
6090	PRRT2	CGTGGACGGGGCCCAGCGTC
6091	PRRX2	CCGAGAAGTTCTTGCGCGCC
6092	PRRX2	CCAGGCGCGCAAGAACTTCT
6093	PRRX2	GGCGCGGTAGCGCCGCCAAG
6094	PRRX2	CCGGCGCGTCAACCTCAGCG
6095	PRRX2	ACTGGACTTCAGTTCACCGT
6096	PRRX2	GGCCGCCATCGAGCAGCCCG
6097	PRSS23	GCACATGGGTGCGTTTCACC
6098	PRSS23	CTGGCTGGGCATCGCATTGC
6099	PRSS23	GTGGAGTTCCCTTATGACAC
6100	PRSS23	GCTGCCCACTGCATACACGA
6101	PRSS23	GTAGACTGGGGCAAGACGAC
6102	PRSS23	ATAATCCATGCCGATGTCAT
6103	PRSS38	ACCAATGCTGCGACCCGGGG
6104	PRSS38	GGTGGTACATCTCATATGTG
6105	PRSS38	ACCTTACCAGTGCCAATTGC
6106	PRSS38	TGGAGCCGCCGCAGACGTGG
6107	PRSS38	GCGGCGTCCCTGCGCCCGAG
6108	PRSS38	AATCTCCCTAACTGGCAGCG
6109	PSAT1	AGCTGTTCCAGACAACTATA
6110	PSAT1	ACAACACGCCTCCATGTTTC
6111	PSAT1	AGGAAGGTGTGCTGACTATG
6112	PSAT1	CAGAGTTACCTGAAACATGG
6113	PSAT1	CTTTCACAGAAATGAGTCAC
6114	PSAT1	CTCTGTATTGTTAATAATCT
6115	PSME4	AGCATGAATTTGACTCCCGA
6116	PSME4	TGTGTAACACCTTGTCTATC
6117	PSME4	TTCTTAAGGCCTGATAGACA
6118	PSME4	CTTCTGAATTATCAAGTATG
6119	PSME4	ATTCATGCTTGTGAGATCCT
6120	PSME4	GTCATTGTTTCTTATTATAA
6121	PSPH	TCCTAGGACACGGCGAGCCA
6122	PSPH	GTTCCTCTTACCTTATGCCG

6123	PSPH	AATACATACAGCAGGAGGAC
6124	PSPH	TTTCCACCAGATTCAGCTGT
6125	PSPH	AAAGTAGAATTTCAGCCTAT
6126	PSPH	CCTTCTTAGGATGCTTTCAT
6127	PSRC1	CCCGATCGACTCCCCCAGTC
6128	PSRC1	AGATCCTCGATGAGGCCAAC
6129	PSRC1	CTGCGCTCTCCCGATCCTGC
6130	PSRC1	ATTCTCTGTCAACTCGAAAA
6131	PSRC1	GCCCGGCTCTCCCCCGGACT
6132	PSRC1	GCCGAATGCCACTCACCAGC
6133	PTEN	ACCGCCAAATTTAATTGCAG
6134	PTEN	CCTACCTCTGCAATTAAATT
6135	PTEN	TTATCCAAACATTATTGCTA
6136	PTEN	ACAGATTGTATATCTTGTAA
6137	PTEN	ACGCCTTCAAGTCTTTCTGC
6138	PTEN	GTATGCTGATCTTCATCAAA
6139	PTGER1	GTCCGCCTCGTCCATCGCTT
6140	PTGER1	AGCGCGGGCGAAGCGCCCGA
6141	PTGER1	GGTGTACATCCTACTGCGCC
6142	PTGER1	CGCAGTAGGATGTACACCCA
6143	PTGER1	TAGCGGCCCACGCGCGCCAG
6144	PTGER1	GTGCAACACGCTCAGCGGCC
6145	PTH1R	CCAGTGCGAAAAACGGCTCA
6146	PTH1R	TGGACATCTGCGTCCACATC
6147	PTH1R	AGATGACGTCATGACTAAAG
6148	PTH1R	CTTGTCTGATTCCATTATGC
6149	PTH1R	CACAGGGCGCCCCTGTCTGC
6150	PTH1R	TTGAAGTCATAAATGTAGTC
6151	PTPN11	GAGACTTCACACTTTCCGTT
6152	PTPN11	TACAGTACTACAACTCAAGC
6153	PTPN11	GTTACTGACCTTTCAGAGGT
6154	PTPN11	GTGCAGATCCTACCTCTGAA
6155	PTPN11	AAGGAACTGAAATACGACGT
6156	PTPN11	ACGGAAAGTGTGAAGTCTCC
6157	PTPN12	GGCCTGCCGAGAATTTGAGA
6158	PTPN12	CAAGAACAGACTACTTCATC
6159	PTPN12	ATATAGTCTGAATCTTGTGA
6160	PTPN12	CCCTTCCCATCTCAAATTCT
6161	PTPN12	TCTTTAATGTCAATTTAACT
6162	PTPRH	AACAGCCACCAACGTCACCG
6163	PTPRH	TCCAGTGCACTGAACGCAGT
6164	PTPRH	ACCTACTGCGTTCAGTGCAC
6165	PTPRH	GTTCAGTGTACTGGAGACGG
6166	PTPRH	GTTCTGTGGGTCTGGGCCGT
6167	PTPRH	CCCGGGTTCAAGTCCATCCA
6168	PTPRJ	CAGGCTCTAACCCGACAAGC
6169	PTPRJ	CTTCCGTGTCTCGGGACTGC
6170	PTPRJ	AATATCTCGGGCCTGAAGCC
6171	PTPRJ	CGCGCGGGGCATGAAGCCGG

6172	PTPRJ	CAGCCTTCCTCACACCCGTG
6173	PTPRJ	CGGCTGCCTCCGCGCTCGCC
6174	PTPRR	TCTCCCATAAACCGAAAAAC
6175	PTPRR	GGCGATTGATGTGCACTTGC
6176	PTPRR	AGCTGAACATAACCTTGCTT
6177	PTPRR	TGAACGAAGAACTTCCTCAG
6178	PTPRR	CATGCAGTCAATTTCCAAAC
6179	PTPRR	CATCAAAGTTTATCCCAGTT
6180	PUSL1	TGAATTCCGTGGAGCCGGTC
6181	PUSL1	CTCCACGGAATTCAGCCGCT
6182	PUSL1	CTCCGCCATGAGTTCGGCGC
6183	PUSL1	CACAAGATAGCGCGCGCGCA
6184	PUSL1	ACGTGGCTGCGTGACGAGCG
6185	PUSL1	ACACCGGCAGCTCATCACGC
6186	PYCARD	CATGTCGCGCAGCACGTTAG
6187	PYCARD	CGCTAACGTGCTGCGCGACA
6188	PYCARD	CGAGGGTCACAAACGTTGAG
6189	PYCARD	CCCTCGCGATAAGCGCAGCC
6190	PYCARD	GGCTCTGGAGCCGCGCCAGC
6191	PYCARD	CCTGGCTTGGCTGCCGACTG
6192	RAB18	CTGTGCACCTCTATAATAGC
6193	RAB18	TTAACTCCCAGCTATTATAG
6194	RAB18	GACATAGTAAACATGCTAGT
6195	RAB18	TCTTTAAGGTGTTGACTTTA
6196	RAB18	AAGAGATACATTTGTTAAAC
6197	RAB18	AATTTCAGTGGATGGAAATA
6198	RABL3	ATGACCAACGACGCAAGTTT
6199	RABL3	CCTCCCAAAACTTGCGTCGT
6200	RABL3	TTAGTCCCTATTACCAACAG
6201	RABL3	GTATTCTACAACTCCGTAAA
6202	RABL3	CAGTCCTAGTTAAAACTTCA
6203	RABL3	CCTATGCCAAAATCAAGTGC
6204	RAC1	AATCCTTACTGTTTGCGGAT
6205	RAC1	ATTTGAAAATGTCCGTGCAA
6206	RAC1	CAATCGGCTTGTCTTTGCCC
6207	RAC1	TACTAGGTTGGAGAAACGTA
6208	RAC1	ACAGTAGGGATATATTCTCC
6209	RAC1	TACACAACCAATGCATTTCC
6210	RAD1	GAATAGATACCTTACAAGAT
6211	RAD1	GTAATCAGACCCAAGTCAAC
6212	RAD1	TAAGAACTGACCTGTTGACT
6213	RAD1	TTCATAGGCTGGAATATTTC
6214	RAD1	TATCTTGTAAGGTATCTATT
6215	RAD1	TGCTGCAGAAATCAAAGTCC
6216	RAD17	AAATTGAAGAAGTCGAAACC
6217	RAD17	TTCAAGGGGATGTTTAATAC
6218	RAD17	CTGCTGCTTTCTAATGTAGA
6219	RAD17	AGATTTCCAGCGAGAAAAAG
6220	RAD17	TAAACCATAAATCTGTTCTA

6221	RAD17	TCTTCCTTAGAACAGATTTA
6222	RAD51	ATGTTTGGAGAATTCCGAAC
6223	RAD51	TGTTGCCTATGCGCCAAAGA
6224	RAD51	TCTCATAGGTATGGTCTCTC
6225	RAD51	TACGCTAGCTGTCACCTGCC
6226	RAD51	AGGCAACAGCCTCCACAGTA
6227	RAD51	TTATCAAGCATCAGCCATGA
6228	RAD52	AGAATACATAAGTAGCCGCA
6229	RAD52	GGCGGCTCAGTGTTATGCTT
6230	RAD52	ATTGTAACCAAACATCTCAT
6231	RAD52	ACTGAGCCGCCGCCAGCAGC
6232	RAD52	TTTCTAGGTGTGCTACATTG
6233	RAD52	TCTGGCCTCCGCCAGCCATG
6234	RAD54B	ACATTAGATCCACCTCATAC
6235	RAD54B	TGCAGCACCAAGTCAGTTGC
6236	RAD54B	GAATTCCCCTGCAACTGACT
6237	RAD54B	GCAGTCTTACCTGTATGAGG
6238	RAD54B	CAACACTGAAATATTTAACT
6239	RAD54B	TAAAGAAGTAGCAGTGTCCA
6240	RAD9A	GGTAGAGCTCGTCCCCGATG
6241	RAD9A	GCGCTGTAAGATCCTGATGA
6242	RAD9A	AGCGGGAGGAGTTCACCGTC
6243	RAD9A	GGCACTGGCTGGTGACGCTC
6244	RAD9A	AGACACCGACTCGCACTCCC
6245	RAD9A	ACCTCACCCGGAATTCCTTG
6246	RAE1	CGTACCGCATAAATGTCCTG
6247	RAE1	TCGATCGTCAAATCCTATGA
6248	RAE1	TGTTACTACAGGTTCGCTGC
6249	RAE1	AGACAACCAATGCTATCATC
6250	RAE1	TTTAAATGTCATCGATCTAA
6251	RAE1	AAGGTGTTACTGTGCTGACG
6252	RAF1	GTAAAAAAGCACGCTTAGAT
6253	RAF1	CAGCGCCGGGCATCAGATGA
6254	RAF1	GCTTGGAAGACGATCAGCAA
6255	RAF1	ACTGATGCTGCGTCTTTGAT
6256	RAF1	ATCCACACCTAATGTCCACA
6257	RAF1	GTGCTGACCATGTGGACATT
6258	RAI2	GGGAGGCTCGGATTTCCGGT
6259	RAI2	TTCAGCCTTGACTTCGCCGC
6260	RAI2	AGCTGATCACCACCGAGGCC
6261	RAI2	GTCAGTGGAGTTGATGTTCC
6262	RAI2	GCACCAGAGCTCAATCCGAA
6263	RAI2	ACAGGCAAGGGGACGATTAC
6264	RALA	TCTACAGTTCATGTACGATG
6265	RALA	CTGTATCTAAGATATCGATC
6266	RALA	AGAGACAACTACTTCCGAAG
6267	RALA	AACACGAGCTAATGTTGACA
6268	RALA	AGCCAAAGAATTCTGACCCT
6269	RALA	AGAAATTCGAGCGAGAAAGA

6270	RAMP2	GCGACTGGGCCATGATTAGC
6271	RAMP2	CAATCTCGCAGGGTGCTATA
6272	RAMP2	TCAAATGGATCCTATCGAAA
6273	RAMP2	TAGCACCCTGCGAGATTGCC
6274	RAMP2	CCGGCGGCCCGCGTCTCCCT
6275	RAMP2	GCGCTGCCGGCCGCCCGACT
6276	RAP1A	GTCCACGTTTAACGACTTAC
6277	RAP1A	ACAGTGTATGCTCGAAATCC
6278	RAP1A	TTGTATATGAAGAACGGCCA
6279	RAP1A	TTACTGTCCCTGCAGTATCC
6280	RAP1A	CCCACAGGTTCCAATGATTT
6281	RAP1A	CCAGACAGTTCAGTTTGTTC
6282	RAP1B	GATAGAAGATTCTTATAGAA
6283	RAP1B	TAGGTTCCAATGATTCTTGT
6284	RAP1B	CAGACTGTACAATTTGTTCA
6285	RAP1B	ATCAAAAATAAATGTTAATG
6286	RAP1B	ACAGTGTATGCTTGAAATCT
6287	RAP1B	CTCGAAGAATCTGTTCTCTC
6288	RAP2A	CGGTCACGAACTGCACGGTC
6289	RAP2A	GACGATGCGCGAGTACAAAG
6290	RAP2A	AGAGAAGTATCGTCCAGCGA
6291	RAP2A	TTCCGCTAAGAGTAAAACAA
6292	RAP2A	CCCTCGCTCACCGCTTCACG
6293	RAP2A	GAAATACGACCCCACCATCG
6294	RAPGEF1	GGGCGCGACCTCTTCATCCG
6295	RAPGEF1	TTATCAACCACCCGAATGCC
6296	RAPGEF1	GCCTCGCCAACCTCATTCGC
6297	RAPGEF1	TTGGTACACTCGGCTATAGC
6298	RAPGEF1	ATCTTTACGGACACCTCAGC
6299	RAPGEF1	CAAGAGAACGCCATCAAAGA
6300	RAPGEF5	TCTTGTGCGTCTAACATCTG
6301	RAPGEF5	GATCTATGGCCATAGGAGTC
6302	RAPGEF5	CAAGTAGCTACATTCATCAG
6303	RAPGEF5	GACATGGGAATTATGTTATC
6304	RAPGEF5	TATCTTGAAAGTATAGATGC
6305	RAPGEF5	AATTTCATCACTGCTTTCTT
6306	RAPGEFL1	TACGTGTTCCACGGGGAGCG
6307	RAPGEFL1	CTCATAGCTGTCCCGAGTAC
6308	RAPGEFL1	CCGCCGGGAGACGGCCAACT
6309	RAPGEFL1	GGGGGCCGGCCACATCATCA
6310	RAPGEFL1	GCCACCTGTTTGCCTGTACT
6311	RAPGEFL1	CCAGGCCCACCTTGATGATG
6312	RARRES1	TTGGAAGCTCTTACGTGATG
6313	RARRES1	ACAGGTGTCACACTACTACT
6314	RARRES1	CACAGAGCGCTACAACCCAG
6315	RARRES1	CCGTGTACAAGTTACATTGA
6316	RARRES1	AGGTATGCTGACTATTTCCA
6317	RARRES1	GTTCCAGATTAATCCAAAAG
6318	RARRES2	GACCAGTGTGGAGAGCGCCG

6319	RARRES2	GCGACGGCTGCTGATCCCTC
6320	RARRES2	CTATGGGGCAGTGGACCAAC
6321	RARRES2	CCAGTGCTGGCTTAGCTGCG
6322	RARRES2	CCCTTCTTACCCGCAGAACT
6323	RARRES2	ATTGGGCCTGACTTTGCACT
6324	RASAL2	CCTGAGATACTCCGTCTACG
6325	RASAL2	CCTCGTAGACGGAGTATCTC
6326	RASAL2	CCTACACCGAGACAACAACG
6327	RASAL2	CCGCTCCCACGTTGTTGTCT
6328	RASAL2	ATTAACTAGATACCTGTGGA
6329	RASAL2	AACTGTCAGTGTCCCTTCCG
6330	RASEF	TCAATACCTCTTCACCGCAA
6331	RASEF	ATTTGGCCATTGCGGTGAAG
6332	RASEF	CGTCAGTATGAAACTGAAGT
6333	RASEF	CAGTTTCATACTGACGTCTG
6334	RASEF	ACTGCATAGCTGCCTTGTCC
6335	RASEF	GGAAATGGATCAGAGGATTC
6336	RB1	TGTTCGAGGTGAACCATTAA
6337	RB1	ACTCCTGTTCTGACCTCGCC
6338	RB1	ATATGGTTCTTTGAGCAACA
6339	RB1	AACATCTAATGGACTTCCAG
6340	RB1	TTCAGGGGAAGTATTACAAA
6341	RB1	AGTCCAAGAGAATTCATAAA
6342	RBBP4	GACCTTGTCAGCTGATCCTG
6343	RBBP4	CATTTAAGACCATCTGCCTG
6344	RBBP4	CAGTGTGAGCATCAACTGAG
6345	RBBP4	CTACTCCATGAGTCTCTGTT
6346	RBBP4	AGTAAGTGCCCACTGAGATT
6347	RBBP4	AACCCAGACTTGCGTCTCCG
6348	RBBP7	AGATGACGACCTGTACGTAC
6349	RBBP7	CGCGCAGCCCCTGTACGTAC
6350	RBBP7	CTGCTGCACGAGTCATTGTT
6351	RBBP7	ACCTTAAATCCCACACATTC
6352	RBBP7	CTGACCGCCGCCTGAATGTG
6353	RBBP7	TCCCCAGCACTAGCCAATGA
6354	RBM6	TCTCGACCTGCTAACAGAAC
6355	RBM6	AAAGGTCCAGTTCTGTTAGC
6356	RBM6	TGGGTTCTTGCAGAATGAAC
6357	RBM6	TTACAGTGTAAGGCAAACAT
6358	RBM6	CCAGGGTAACTTCTTTGTCC
6359	RBM6	CCAGGACAAAGAAGTTACCC
6360	RBMXL1	ATCTCTACTCAAGTTGTGAC
6361	RBMXL1	CGTAGTGCTCCACTTACACG
6362	RBMXL1	GGCTTCCCCCTTCTGTAGAA
6363	RBMXL1	GGGTACCCCCTTTCTACAGA
6364	RBMXL1	CTGCTGCGAACTAGTCCTGA
6365	RBMXL1	TCCATGTGTCCTCCTCGTGA
6366	RBX1	CAGAAGAGTGTACTGTCGCA
6367	RBX1	AACACGACAGGTGTGTCCAT

6368	RBX1	GTTATCAACCACAATATCCC
6369	RBX1	ACTTCCACTGCATCTCTCGC
6370	RBX1	ACTTACAAAGATCCATAATG
6371	RBX1	GTCTTTCAGGTATGGGCACT
6372	RC3H2	CCCCTTGCTGTCGCAAATCT
6373	RC3H2	TGCCGAGATTTGCGACAGCA
6374	RC3H2	GTACCAGAACTTTCCTTGAG
6375	RC3H2	GTAGCTGCTACATTACTTAC
6376	RC3H2	GTCCCACAGTACCCACAGAC
6377	RC3H2	ACTAGAACCAAGATTTCCTC
6378	RCBTB1	CTGTAGAACCTGATCCCACT
6379	RCBTB1	CCAAGGCATTGCTCCCGTCC
6380	RCBTB1	CATGTTCTTCTCAGCACCGA
6381	RCBTB1	GCCATTGTCCAGAACAGCCA
6382	RCBTB1	CACAATGGATATAGCCAGCT
6383	RCBTB1	TCGGTGCTGAGAAGAACATG
6384	RCHY1	ACTAGATCGCTTTAAAGTGA
6385	RCHY1	CATTGTTATCATGACACAAG
6386	RCHY1	TGCCTAGCTATGAATCTTCA
6387	RCHY1	GTTGCTCATGTCTTGCCATG
6388	RCHY1	CAAGACATGAGCAACAACAC
6389	RCHY1	TTCCTTGAAGATTCATAGCT
6390	RELL2	CGTCCCTGACGGACAAGTGG
6391	RELL2	TGTCCGTCAGGGACGCTCCA
6392	RELL2	CTAACCCCTCGTGCACTTGA
6393	RELL2	GGTTCCCTTCAAGTGCACGA
6394	RELL2	TGAGGACGATGACATGAATG
6395	RELL2	TCTTCCCTCAGCCAATGCTG
6396	RELT	CGATGGCGATGACCGCGTAC
6397	RELT	CTACCACTGCACGGCGCACA
6398	RELT	CTCGAGTTGCCATGCCCACC
6399	RELT	CTGCATAATGTGCCCTGCCC
6400	RELT	TGAAGCCAAGTCTGCTGTGC
6401	RELT	CCAGCTCACCATAAGGAAGC
6402	RERE	AGTACCATTTGACGTTCATG
6403	RERE	ATCTCCTCATGAACGTCAAA
6404	RERE	CAACAATAGTGCCACCGCAG
6405	RERE	AAAAAAAGTCTCGTTATGAA
6406	RERE	ACTGTGTGTATATCGAGAGT
6407	RERE	TGAATGCTACAGATGAAATA
6408	RERGL	TTTCTTACTAAGCGATTCAT
6409	RERGL	AGATCTTTGTCATGTGCGAG
6410	RERGL	TAGTAAGAAACCTCACTGTA
6411	RERGL	CCCTCACAAGTGAGCTTCAC
6412	RERGL	AAGCGCTGATCTACAGAATC
6413	RERGL	CTATAAGAAGCACTTGTGTT
6414	RET	ATCTCCTCGCTGCAGTTGTC
6415	RET	TCCGGTGGCCGGTCTTCAGA
6416	RET	GTCCCGAGATGTTTATGAAG

6417	RET	AGGGTCGGATTCCAGTTAAA
6418	RET	TTTCCTCACAGCTCGTTCAT
6419	RET	CACTTACACATCACTTTGCG
6420	REV3L	GCTCCGAAGACTCGCACCAC
6421	REV3L	TACCTCTATGTGCCATACGA
6422	REV3L	TGCTGTCCATAACCATCGTA
6423	REV3L	GCGAGTCTTCGGAGCGACCC
6424	REV3L	CTATCTTTACAATCCTACAA
6425	REV3L	TTCTCATGATAACCATAAAA
6426	RFC2	CTCGATAACATTCATCAGCC
6427	RFC2	AGACACCGTGAGCAGGCTAG
6428	RFC2	GACTGCACAGCGGGACTGAA
6429	RFC2	TTTCGCCGTAGGTCTTTGCA
6430	RFC2	GACCACAAGCATTCTGTGCT
6431	RFC2	TGAAGCATTGAGTTCCAACA
6432	RFC3	GACTCACCAGGTTCCGCAGC
6433	RFC3	GCCGATACTTGTCCACCCAG
6434	RFC3	ACCACCTTGAAGTTAATCCT
6435	RFC3	CTTACCTAGGATTAACTTCA
6436	RFC3	TGTATTCTACGTGAACTTTA
6437	RFC3	TTTCCTCATCTGTTAGTGTA
6438	RFC4	TACTAAACCCCCGCTGACCA
6439	RFC4	CTCTCCGCTACTTCCCGCAC
6440	RFC4	CGCAAGTCGCCCAAAATGTG
6441	RFC4	GGTCCGTAAAACAAGAGATT
6442	RFC4	TCTAGCTGCTGCCAAAATAG
6443	RFC4	GCAACTTCATCCACACATTT
6444	RFC5	AGCCCTACGCATGTCTCCAC
6445	RFC5	ATTCTGGGCGTCCTGAGTCA
6446	RFC5	TTTATATAGCTGTTTCGCAC
6447	RFC5	GCACTAGTCACTCTTTCCAG
6448	RFC5	GTAGATGTCTTGCCTGTCCC
6449	RFC5	GCCCAGAATGCCTTGAGAAG
6450	RGN	TAGCGACCAATCCAAACCAT
6451	RGN	CCCGCCGGGAGGTACTTTGC
6452	RGN	TGGCAACATAGCCTCCCGAC
6453	RGN	GCATCAATACACATTCCATC
6454	RGN	ATTCGTTTAGATCCTGTGAC
6455	RGN	AAATGTATGTGACCTGCGCC
6456	RHEB	CTACATACCTTTCCATATGC
6457	RHEB	ATTCTGTTACATCAATCAAA
6458	RHEB	AAATAGAATACCTATTATGT
6459	RHEB	CTTCAACTTGTAGACACAGC
6460	RHEB	TTGACGATTCAATTTGTTGA
6461	RHEB	GATCGTAGGAGTCCACAAAT
6462	RHOA	ATCGACAGCCCTGATAGTTT
6463	RHOA	GCTGCTCTGCAAGCTAGACG
6464	RHOA	GGCCACTCACCTAAACTATC
6465	RHOA	TATCGAGGTGGATGGAAAGC

6466	RHOA	GAGCCGGTGAAACCTGAAGA
6467	RHOA	GAGCAAGCATGTCTTTCCAC
6468	RHPN2	GACCGACGCGCTGTTGCCCG
6469	RHPN2	GGCCGCGGGCAACAGCGCGT
6470	RHPN2	TCCTCATCCGCACGGCTTTC
6471	RHPN2	TGTAATCCCCTTGCACAAAC
6472	RHPN2	AGATGAAATTGCAGATCTTA
6473	RHPN2	CATCTTCACTGTAATGTTCC
6474	RIN1	ACTTGCAGACGTTCCTCGTG
6475	RIN1	GGGTGTTAGATTTCCGCACG
6476	RIN1	GCGCTCATGGACCGAAGCAG
6477	RIN1	GCCGCCGACGACCTCTGCTT
6478	RIN1	GGAGCGCCGTAGCTCCTGCA
6479	RIN1	CATTGGGCACGTCATACAGT
6480	RINT1	GAGGCGCCGATCTCGCCGGC
6481	RINT1	CACAGTGTTCATGGCTATCC
6482	RINT1	CGTCAACCATCTCTGAAAAC
6483	RINT1	GGAGGCGAGATGCTACCAGC
6484	RINT1	TGACGAAAGGAAGAACCTCG
6485	RINT1	TTCTAGTGCTGCTCTGAAAG
6486	RMND5A	GTGGAGCACTTCTTTCGACA
6487	RMND5A	TCACCTCATTGAGAAGCCTT
6488	RMND5A	CCGCCCTACCGTGGCTCTGC
6489	RMND5A	CGGCATGGATCAGTGCGTGA
6490	RMND5A	CAACCCGAGAAACACTGCTG
6491	RMND5A	GGCCAAGATGCTGAATTATC
6492	RNASE2	GAGCTTAGATGATTCTATCC
6493	RNASE2	CGTTAGCAAAAGTTGTAAGA
6494	RNASE2	CGCAAAAATTGTCACCACAG
6495	RNASE2	GTTAATGACCTGCATTGCAT
6496	RNASE2	GGGAGGTCATATTGATGTGC
6497	RNASE2	ACTGGAACCACCGGATACTG
6498	RNF139	GTCTACTACGTTCGTTCAAC
6499	RNF139	CGATTCTACACTAACTGTAC
6500	RNF139	ATCCGGGTAGGAGTTGAAGA
6501	RNF139	TTCCCTTACCAAAGAGCCGG
6502	RNF139	CAAAAGACTCGTAGTATGTC
6503	RNF139	GATAAGTGCCATCCACAGCG
6504	RNF151	CAGTGTCGTTCGTAGAGAGC
6505	RNF151	CAGCTCTCTACGAACGACAC
6506	RNF151	AAAAGACCTGTCCGTGCTGT
6507	RNF151	GAGCACACGAAGTTGCTGTC
6508	RNF151	TTCGTGTGCTCCGTCTGCCA
6509	RNF151	CATTGGCCGCCTGGAAGTCA
6510	RNF170	GATTATAACCGGAGATTCTC
6511	RNF170	GAAGTACCCTTACTAGCTCC
6512	RNF170	TCGCCAGTAAGCAATAATGC
6513	RNF170	ATTACCGTTTGTCTACAGAT
6514	RNF170	ACCTTACTCCTAACAGTATT

6515	RNF170	AGAACAGCTTCAAACAGAAC
6516	RNF185	AGCTGGCATCAGCCGAGACA
6517	RNF185	ACACACCTGTCTGTTAGGTC
6518	RNF185	CAGTTGGCCGTGTTTACATC
6519	RNF185	CATCTTACCTGATGTAAACA
6520	RNF185	ACACAGGGATTTCAAGGATT
6521	RNF185	TTCCTCACCCCTCTATTCTC
6522	RNF38	TCAGAACACTACCGTTCAAG
6523	RNF38	CGCACAGCAGCAAGCAATAG
6524	RNF38	CCTCACCACCAATGCGACCA
6525	RNF38	ATTGCTTGCTGCTGTGCGTA
6526	RNF38	AACCTTCTTCTGTTGCGTGC
6527	RNF38	CCCTGCTTGTAGTACACAGC
6528	RNF8	GAGCGCGTCTGGAACCTTTA
6529	RNF8	CCCAGAGTCTAAATGGTGTT
6530	RNF8	TCCCAGGTGACTGTAGGACG
6531	RNF8	ATTTGGTGTCACATACCAAC
6532	RNF8	TTTGCAGCATTGGGCTCTAA
6533	RNF8	AGAGCTAAATCGCAGCAAGA
6534	RNMT	GTACCAATAAAATAGCCCCC
6535	RNMT	ATTAACAAGCTAGTTTGTAC
6536	RNMT	CTCATTTGAGTCTTATGAGC
6537	RNMT	AAAACGTGATATCACTGTTT
6538	RNMT	TATAACTGCTGACAGCTCAA
6539	RNMT	CTGTCAAACAGTGTCAGCAG
6540	ROS1	GGAATTTAATGACCCAGTGC
6541	ROS1	GCTGGAGTCCCAAATAAACC
6542	ROS1	GTTAAAACTTCATGCAGCCA
6543	ROS1	CTGACATTATATGAAGTATA
6544	ROS1	ACTGTTGTTTGCTTCATCTC
6545	ROS1	CATACTATATCCTTGAGATA
6546	RPA2	GCCGCCGGCTCCCCCGTATG
6547	RPA2	GCGGGCGATCCAAAGCCCCC
6548	RPA2	TCTAACCTAGGTCACTATTG
6549	RPA2	ACTTTACCTGAAAAGATCTC
6550	RPA2	GAACACTTCATCAACCAAAG
6551	RPA2	AAGCAGCTGAGATATAGTAC
6552	RPA3	TACGGGTTCCATCAACTCGA
6553	RPA3	GGTTGGAAGAGTAACCGCCA
6554	RPA3	GATGAATTGAGCTAGCATGC
6555	RPA3	ATAAGATGTACACAAGATGG
6556	RPA3	AAATGGAACCATCGAGTTGA
6557	RPA3	GACAAGCCTGTCTGCTTCGT
6558	RPL27A	GGCACTTACCTATGCGGCCG
6559	RPL27A	ACTATTTGTCGAAGTTGATC
6560	RPL27A	CCTAGCCACCCAGGCTACTT
6561	RPL27A	CTAGGGCTACTACAAAGTTC
6562	RPL27A	CTTAGGGGCCACGTGAGCCA
6563	RPL27A	TTCACGATGACAGGCTGCTT

6564	RPS6	TACTACAGTGCCTCGCCGCC
6565	RPS6	TAGACTAACCTTCATTCTCT
6566	RPS6	CCTCCTACCTTCTTTATTTA
6567	RPS6	AAATCTGAGCGTTCTCAACT
6568	RPS6	TACGCCGCCGTTTGTGCTGC
6569	RPS6	TACTTTCTATGAGAAGCGTA
6570	RPS6KA2	AAGGTGAAGGGGTCCGACGC
6571	RPS6KA2	GGACCGAGTGAGATCGAAGA
6572	RPS6KA2	GGCGTAGAGCTGCCCAGCGT
6573	RPS6KA2	GCAGGAAGAAGGCGTCGTGA
6574	RPS6KA2	TCTCCATCTTCGATCTCACT
6575	RPS6KA2	AGACATCAGCCATCATGTGA
6576	RPTOR	CTCCTTGGCGTTGCGACGTA
6577	RPTOR	GTCCTTACGTCGCAACGCCA
6578	RPTOR	GACCACGCCCTGTGCACGCT
6579	RPTOR	CCAGCATTCCAAGCGTGCAC
6580	RPTOR	ATCAAATCCAGTGTGACGCC
6581	RPTOR	CATGCAGAAATGTGTCAGTC
6582	RRM2B	CAATCCAGTACCCTGATATT
6583	RRM2B	TTACTAAGTATTGGAACATC
6584	RRM2B	ATTCCAGGTCGACTTATCAA
6585	RRM2B	TCCTAAGAAAGAGTTCTCGC
6586	RRM2B	GCTTTAAGCTTGTTCCAGTG
6587	RRM2B	TGATGCTGTCAAAATTGAGC
6588	RSBN1	GCAAGAATATCGGGTACCCC
6589	RSBN1	ACGAGACTTATCTGCCGCAT
6590	RSBN1	TAAAAGGTGTTTGTACTCCC
6591	RSBN1	CCTACCGAGCAGGTCCTATG
6592	RSBN1	CTAGCCTCCGACTCCAGTGT
6593	RSBN1	GTGATGATGGGCCTATAATG
6594	RSF1	ACTATGCGTCTCCAGCCAAT
6595	RSF1	CATTGTGATCTTGATCCAAT
6596	RSF1	GATGACATCAAAGAAGCCGA
6597	RSF1	CACCTAAAGAAAAGAACGCT
6598	RSF1	CAAACACATACCTCCTCCAT
6599	RSF1	AATCTGTTACTGCAGACAGA
6600	RSPO4	ACATGCTCGCCCTGAACCGA
6601	RSPO4	CCGCGGATGCCGAAGTACCC
6602	RSPO4	TTCCTGTTCATCCGCCGGGA
6603	RSPO4	GGGCGAGCATGTCCACGGCG
6604	RSPO4	ACTTCTGCATCCGGTGCAAG
6605	RSPO4	CTTTGGCCCACCAGAACACA
6606	RUNDC3B	CAAAGCAAGACCGATCAATC
6607	RUNDC3B	CATGAGTGCTACTCTGATCC
6608	RUNDC3B	GACTCAATGCTATTGATTTC
6609	RUNDC3B	GAAGAAGCAAATATGCTTGC
6610	RUNDC3B	CTGTGAAGACCCTGATTGAT
6611	RUNDC3B	TGAGAGACTTCAAAACAACC
6612	RUVBL1	ACACCTCTCGCGCGTTCTCC

6613	RUVBL1	ACTACTTACCAATGGCCCTG
6614	RUVBL1	ATGAGTACCTTGCCAGTTCC
6615	RUVBL1	TCCCCTTCTGCCCAATGGTG
6616	RUVBL1	GCCTCCCACAGCCACGTGAA
6617	RUVBL1	GGAAGAGCTGTCTTGTTGGC
6618	RUVBL2	AGCAATAAGGACTGCCCGAC
6619	RUVBL2	GGTCGGGCAGTCCTTATTGC
6620	RUVBL2	ACTGGGGCTGGACGATGCCT
6621	RUVBL2	ACTCAATCATCTTGGTGCCC
6622	RUVBL2	CTCTACCTGCCGAGGCTCCA
6623	RUVBL2	GTAGATGGTCTCCATCTCTG
6624	RYBP	CCGTAATTTACACGAATACT
6625	RYBP	GCGAAACCTGCCGCAGACGA
6626	RYBP	ACACGCTGCCTACTAATTGT
6627	RYBP	ACCAGCTGAGAATTGATCCG
6628	RYBP	TGAGGTGTGATTTGTTTCGC
6629	RYBP	ATCCCAAAACCCTTCGTCTG
6630	RYR1	CTGACAGTGATGACCAGCGC
6631	RYR1	GTTCCATAGTGTCTGCATGA
6632	RYR1	CAGCACCTGTCGACCGCCAG
6633	RYR1	CACGTCCTCCGCCTCTTTCA
6634	RYR1	CCGCCCTACCTGATTCTCAG
6635	RYR1	CCCCAGACTTGTCTACTATG
6636	RYR2	GGCGCGGAACCATGGCCGAT
6637	RYR2	CGCTTACAGTTCGCAGGAAC
6638	RYR2	CCAGCTTATCAGTTGAAGAC
6639	RYR2	TCCCACCTTGGAATTGGAAG
6640	RYR2	CCCTTTGCAGGATGATGAAG
6641	RYR2	TGTGCTGCCTGTCCACCTCC
6642	RYR3	GCACAACGTTTGAGAGGTCG
6643	RYR3	GGTGTCATACCTGGTTGGCC
6644	RYR3	GAGAAGCTTTGGTTCAACAG
6645	RYR3	CTGAGAACTATCACAATATC
6646	RYR3	GGCACTGACCTGGAGCTCTC
6647	RYR3	TGGGACACAGGGAATGGTGG
6648	S100A7	CGCCGATGTCTTTGAGAAAA
6649	S100A7	ACCGACTCACACAGGCACTA
6650	S100A7	CTCAATCTTGTCATCACGTC
6651	S100A7	GCTGGGTCACTGGCTGCCCC
6652	S100A7	CTGCTCCATGGCTCTGCTTG
6653	S100A7	TGTGGCTATGTCTCCCAGCA
6654	S1PR2	CATCGCCATTGAGCGCCACG
6655	S1PR2	CGCAGTAGATGCGCACGTAC
6656	S1PR2	GATAAAGACGCCTAGCACGA
6657	S1PR2	CTCCGATCTACTGGCAGGCG
6658	S1PR2	CCACCACAATGGCGCAACAG
6659	S1PR2	TAGGCGTCTTTATCGTCTGC
6660	SAA1	CTGATCAGGCTGCCAATGAA
6661	SAA1	GCCACTCCTGCCCCATTCAT

6662	SAA1	CGTGATCACTTCTGCAGCCC
6663	SAA1	AGCTTCGGCTGCTGACACCC
6664	SAA1	TACCATCAAAAGCCTCGCCA
6665	SAA1	TTCCAGGGGCTCGGGACATG
6666	SAA2-SAA4	GGATATCATTATGTCCCAAT
6667	SAA2-SAA4	AAACGAACGCCAGCTTTCAC
6668	SAA2-SAA4	TCTGGGCTGCTAAACTCATC
6669	SAA2-SAA4	CCTGGGCTGCAGAAGTGATC
6670	SAA2-SAA4	AGCTTCGGCTGCTGACACTC
6671	SAA2-SAA4	CCTGATCACTTCTGCAGCCC
6672	SAA4	CCCGACCGCTTCAGACCTGA
6673	SAA4	TAATCCCTGAAGATAGACCC
6674	SAA4	GGATATCATTATGTCCCAAT
6675	SAA4	AAACGAACGCCAGCTTTCAC
6676	SAA4	TCTGGGCTGCTAAACTCATC
6677	SAA4	GAAGTCCAACGAGAAAGCTG
6678	SAC3D1	TACTCCTTCACCGCGCGCTG
6679	SAC3D1	GGCGGTGCTCCCTTTCGCGC
6680	SAC3D1	CACTAACACCTTGCACGTAC
6681	SAC3D1	CCTCCACGTAGCGACCCCTC
6682	SAC3D1	CGCTGCTGACCGTAGTGGCG
6683	SAC3D1	GCACCGCCTGCACCGCTTGG
6684	SALL1	GCTCGCCGTCGGGACTGAGT
6685	SALL1	AGCCGCCGACCCCGTCGTGC
6686	SALL1	TTTCCAATCCGACCCCGAAG
6687	SALL1	CCCACCAGTCACGTCCCGTC
6688	SALL1	TTTGAGCCAGCATGTCGCGG
6689	SALL1	AATAGCACCCCTGCACGACG
6690	SART3	GGCCGCCTTTACTCGTGCCT
6691	SART3	TAGTTCTGAATTACTGACTC
6692	SART3	CTATGACTACAACTGCCATG
6693	SART3	CTGAAGCTTACCTTCAGTCA
6694	SART3	GTTAGACTCCAGTAAAGAGC
6695	SART3	TAAACCCTATGAAGAAGCAC
6696	SATB1	AACGGGAGCCCGCTAGGAAG
6697	SATB1	GCTTTCAGAAATCCTCCGAA
6698	SATB1	ATCTTGGCTGGTGGACCCTT
6699	SATB1	AGAAATTCTGCATAGCCCGA
6700	SATB1	GCATCTGTCACGTAAGACAG
6701	SATB1	ATCTGAAATAGGGCTAATCC
6702	SATB2	CAGCGAACGCATGCAACACG
6703	SATB2	ACCAACAGATTGCCGTTAGC
6704	SATB2	CGTGTTGCATGCGTTCGCTG
6705	SATB2	AGCTTAGTCCACAACTTGTA
6706	SATB2	TCGGGGCTGTCCCGCAGACA
6707	SATB2	GTCTGCGGGACAGCCCCGAC
6708	SBSN	ACAACCACGCCGTTAGCCTC
6709	SBSN	GTTCCACACTGGGGTCCACC
6710	SBSN	GATGAAAGGCGTGTTGACCG

6711	SBSN	TCATCAACCTTCCCGCCCTG
6712	SBSN	CAGCAGCATGGTTGACCCCT
6713	SBSN	TGGTTGTGGCCCCTCCTTGA
6714	SCAF4	CGACAGTCTCGTCATCAGTT
6715	SCAF4	CGCCGTCAACGCCTTCAACC
6716	SCAF4	AGCTGCTATTAAAGCTATTA
6717	SCAF4	ACCCCTTACCTCCTGGTTGA
6718	SCAF4	GCAGTGTAAACCAGAATACA
6719	SCAF4	TTTAGTGATGAGAATCATCT
6720	SCAMP1	TTCCCGACGATCTAATTCTG
6721	SCAMP1	AAGCCGCAGAATTAGATCGT
6722	SCAMP1	TTAGGCATCTTCACACCGCC
6723	SCAMP1	GTGACAAGAAATGTTCCACC
6724	SCAMP1	TTTGCAATCTGTGTATAAGC
6725	SCAMP1	TTATATTCATCAAGTCCTGG
6726	SCMH1	GGTTTACGCCCCCTCTGCCC
6727	SCMH1	GGTGGGTTCAGCAAAGTCCG
6728	SCMH1	AACAACCAGCACTCCTGAAC
6729	SCMH1	GGGCTTCTCAGTATTCACAT
6730	SCMH1	GAGTCTGCATCCCAATATCA
6731	SCMH1	ACTAGAAGCTGCTGACATCC
6732	SCNN1B	GGGGGTTCCGTTCATCAATA
6733	SCNN1B	TCTTCGCCGCCCTCGTCTGC
6734	SCNN1B	ATGATCCTGGCCTGCCTATT
6735	SCNN1B	CCCCTCACAGATGATGCGCT
6736	SCNN1B	TGGCTCTCACCGGTAGTTGC
6737	SCNN1B	AGCTGCTTGTTAAGCCATTG
6738	SCNN1D	GCTACACGGTCGATGGCGTC
6739	SCNN1D	GACCGTGTAGCAGCTGCCGT
6740	SCNN1D	GCTGCCAGCTGCCCCCGCAA
6741	SCNN1D	GGCCACCATCAGCATCCGAG
6742	SCNN1D	GCTCACCTCTCGGATGCTGA
6743	SCNN1D	GAGGTGAGAGCCCCCCCGTG
6744	SCOC	CAGGCGTGTATTCTCGAGTC
6745	SCOC	TGAACAAGATGGACGGGTCC
6746	SCOC	TTCTCACGGCGCACGTTCTG
6747	SCOC	GCCAACCTTCAAGTGTGTGT
6748	SCOC	AAGACTTATTAATCAAGTGT
6749	SCOC	CCTTGGATACAAAATTCTTG
6750	SDCBP	TCTGCTCCTATCCCTCACGA
6751	SDCBP	ATAAACCTACTTCCATCGTG
6752	SDCBP	AGCCACCATATAGTTTATAC
6753	SDCBP	ACTATACATACCCCCTGAAG
6754	SDCBP	GGTTTCTGGTGCACCACTTC
6755	SDCBP	ACAGAATGTCATTGGATTGA
6756	SDE2	ATCCCGACAAGCTTCTCGAT
6757	SDE2	GGATCTATGCTCCGAGCACT
6758	SDE2	AAAACAACAAGCCGAGCGAG
6759	SDE2	CCAGACGCTCAGCCATCTCA

6760	SDE2	TTCTGCTGAAACCATCTTGC
6761	SDE2	CTGTCTGTTTGAGATTTAGT
6762	SDHA	GCCCATCACCTCGACCACGG
6763	SDHA	CGGCCTTACCCTTCCATATA
6764	SDHA	GCAGGCTTGCGAGCTGCATT
6765	SDHA	GGGCCTGCTCCGTCATGTAG
6766	SDHA	TATTAAACCCTGCCTCAGAA
6767	SDHA	TCGCTATTGCACACCTTATA
6768	SDHAF2	AGTGTTCTCGACTTCGTCGC
6769	SDHAF2	TTAACGAGCCTAGTAATGAC
6770	SDHAF2	CGTTAATCAGGCGGTCATAG
6771	SDHAF2	TCATTCAGACGCTTCTACAG
6772	SDHAF2	CAGCCCAACAGATTCCCAAA
6773	SDHAF2	AATATTTGAAAATGAAGTCA
6774	SDHB	GTCTGGGTCCCATCGATAGA
6775	SDHB	TCGCCCTCTCCTTGAGGCGC
6776	SDHB	GGCCGGCAACCGGCGCCTCA
6777	SDHB	GATTAAAGCATCCAATACCA
6778	SDHB	AGAAATTTGCCATCTATCGA
6779	SDHB	CTTTGTTAGATGTGGCCCCA
6780	SDHC	CTCCCCACATTACTATCTAC
6781	SDHC	CGGTTCTGGAATAAGAATAT
6782	SDHC	TACTTTCTGATACAGAGCTG
6783	SDHC	TTACAGTTGGTCTCTTCCCA
6784	SDHC	CAAGTGCAAACTTAGCTGTG
6785	SDHC	ATGTCGGCCCTGTTACTCCC
6786	SDHD	GAGAACATACAATGGTGGCT
6787	SDHD	AGTGCCGTTTGCGGTGCCCT
6788	SDHD	TCTGTTGCTTCGAACTCCAG
6789	SDHD	TGCCTTGCAGAAAGCTGCCA
6790	SDHD	GCCAGTGACCATGAAGAGTG
6791	SDHD	ACCCCTCACCTCGGCCTCCT
6792	SDS	GTCCATCTTGAGGTAGACGC
6793	SDS	ATTGGATGAAGCCTTCGAGC
6794	SDS	GTTGTTCTTCGCTAGGGCCT
6795	SDS	ATGGGGGTCTTCACGTGCAG
6796	SDS	AAAATGTGCACAGCCTTGCT
6797	SDS	TCTCCACAGTGGGCCAAGCA
6798	SDSL	CGTTTGACCACCCCCTAATA
6799	SDSL	CAACTCTTGCGCCCTCAGAT
6800	SDSL	GTGCCAAGACGGTGGCCGCT
6801	SDSL	GTGCAAGATTCACTCTGAAG
6802	SDSL	TTTCTGCACAGGGGGTAATG
6803	SDSL	GGTGCTCTCGGGGAGCACGA
6804	SEC23B	TGCAATCTTCAGTTCCCGAG
6805	SEC23B	TACAATTGAGTACGTGATAC
6806	SEC23B	TCAGATATGCCTCCATAAGC
6807	SEC23B	TCTCGGGAACTGAAGATTGC
6808	SEC23B	TTGATTATCGAGCAAAACTT

6809	SEC23B	TCTTTGAAAACAGAAATTAC
6810	SEC24B	ACTCTAACGTGGTCATCTCC
6811	SEC24B	GGGAGGCACTCGTATGCAAA
6812	SEC24B	GCCCAACAGTTATGATGCCC
6813	SEC24B	ACTGAGGTCACAGGCTCAAC
6814	SEC24B	GGGAGGTGTTCTTGTTGATG
6815	SEC24B	GCATCATAACTGTTGGGCAT
6816	SEC31B	CGATCTCTTAGCTGCTGAAC
6817	SEC31B	GGCAGTTACCGAGGAGATTT
6818	SEC31B	CTGAGTGACCCTGTAGGTTG
6819	SEC31B	CCGGACCCAGTTCCCCAAGC
6820	SEC31B	GGGTAGCTCCCACAACAATC
6821	SEC31B	AGCAACTGCGGGGTCCTCAT
6822	SEC61A1	GCTGAATGCCTTCCATACGA
6823	SEC61A1	TCATCACATACACGATAGAC
6824	SEC61A1	TGTTGTACTGGCCACGGTAG
6825	SEC61A1	ATCAAGTCGGCCCGCTACCG
6826	SEC61A1	TCTATCGTGTATGTGATGAC
6827	SEC61A1	TCGGCCAGTGTTGACAGTAG
6828	SEC61A2	GTTATTCTGGCTTCCAATAG
6829	SEC61A2	ATCTCCCCAATTGTAACATC
6830	SEC61A2	ATCAAACCAGATGTTACAAT
6831	SEC61A2	ATAACTCTCATCCAGTAGAA
6832	SEC61A2	ACAGATCCAGTTTAGAGAGA
6833	SEC61A2	ACAGAACCTTCTCTCTAAAC
6834	SEC62	CTGTGGTTGACTACTGCAAC
6835	SEC62	ACAGTTGAATCGAAGATACT
6836	SEC62	ACTTGAGCCACATGATGATC
6837	SEC62	AACAAAGTCCACCAATATGA
6838	SEC62	GTAGTCAACCACAGACTCCC
6839	SEC62	AGAAAAACCTGATCATCATG
6840	SEMA5A	CGCTCTGAACTCCCGTAACC
6841	SEMA5A	TCTGGCACTGAGTCGTACCC
6842	SEMA5A	GAACAGCCATGCTATAACAC
6843	SEMA5A	GTTCAGAGCGAAGAATGCTG
6844	SEMA5A	CCTCAAGCTGTAACCTGAAG
6845	SEMA5A	TTTGCAGAAACTACCTCTTC
6846	SEMA6A	GAGCCAATCAGTATTTCGCA
6847	SEMA6A	TTGCCATGCGAAATACTGAT
6848	SEMA6A	CGTTCATGATCATAATCATC
6849	SEMA6A	GGCTTGTGGCCCACAAACAC
6850	SEMA6A	TGATATAGACACATCACACA
6851	SEMA6A	CTATATCAACAGTATAAATA
6852	SESTD1	TAGAGGATTTACCGTGATTG
6853	SESTD1	CATCCATATTTGTCTGTTCG
6854	SESTD1	AACAGTAGTCGTAATGCTAC
6855	SESTD1	ACTACCTACTCAGCATTCCA
6856	SESTD1	CAGAATGGGTAATATTACTG
6857	SESTD1	AAAAAACTAGCCTTCCTTTC

6858	SET	GAGCAGCACCATGTCGGCGC
6859	SET	CCGGCCGCACCATGTGGCGG
6860	SET	CTGATGGGAGCGAGGCATCA
6861	SET	GCTCTCACCTGAGGTCTCGT
6862	SET	TGTGGCTCACCCAGCAAGGT
6863	SET	CAGCTTGAAACCTACCTTGC
6864	SETD2	AGTTCTTCTCGGTGTCCAAA
6865	SETD2	GACTATCAGTTCCAGAGATA
6866	SETD2	AACTTACGAAGGAAGGTCTT
6867	SETD2	AACGTTAACTCTGAGCCTGA
6868	SETD2	ATAATAGATGCCACTCAAAA
6869	SETD2	TCATCTCAGTGGACTGTGAA
6870	SF3A1	CCATGCCTACTACCGCCACA
6871	SF3A1	GGGTAAATAATCCCCACAAC
6872	SF3A1	GTAGGCATGGTAAGGGTCAT
6873	SF3A1	AGTGATGGGTGGAGCCGAGC
6874	SF3A1	CAGCATGGCCCGGACCCAGC
6875	SF3A1	TGCACCTTCTAAGCCAGTTG
6876	SF3A3	CATCTTGCATGGCCCGAGTG
6877	SF3A3	ATGTACAGCGCAAGCAAGCC
6878	SF3A3	ACTTTGCCATCCCAGCCAAG
6879	SF3A3	CCGCTTGGTGCCCTTTGACT
6880	SF3A3	GATCTGTGTGCCAATGTCAG
6881	SF3A3	TCCTCAAATTCCACTGACAT
6882	SFXN2	TTAACATCGATGCCCCCCGT
6883	SFXN2	CCCGTGATGATCATGCCGCC
6884	SFXN2	AAGGTGCGCTGGTCCCAACG
6885	SFXN2	GGCCGAGTCATACAGCTTCT
6886	SFXN2	TGTGTTTAGGGAGCTCATAA
6887	SFXN2	GACAGGAATGAAAATGAGAT
6888	SGK1	GCTACATGCCTCTGATAAGC
6889	SGK1	GCACATTGCAGGTACGAAGG
6890	SGK1	CCGCCACAAAAAGAGGGCCG
6891	SGK1	TTCTTGGATCGGGCTTGGTC
6892	SGK1	CACAAGGAAGACTCGAAGTC
6893	SGK1	GCTTACCCAGTCCGCTCAGC
6894	SGSM1	GCTACGCACCGTCAAGAAGG
6895	SGSM1	CAAGAACTTCCCGCCGGCTG
6896	SGSM1	CTGCAATCTTATTGCTGCGT
6897	SGSM1	CAGAGGCGGAGACCCGACAG
6898	SGSM1	CCTCTTACCCGCGGGGGCCG
6899	SGSM1	AGCCATGGCCTCGGCCCCCG
6900	SH2D1B	AGAGACAGCGAGTCGATACC
6901	SH2D1B	CTTGCTTGGTCAGACGTCCA
6902	SH2D1B	GAACAGTAACAGCGATTATG
6903	SH2D1B	TGAACCACCATCCCCTGATT
6904	SH2D1B	CGTGTTTCTCTCTGAAGATT
6905	SH2D1B	TGCAGAAGGTTCTCCAAAAC
6906	SH2D2A	GGTGCGGTTCAGCGAGAGCG

6907	SH2D2A	TCACCTGTAAGTCAGCACGA
6908	SH2D2A	TGCGGGTCATGTCTGTGATC
6909	SH2D2A	ACCTCCGGGTGATGAAGCCA
6910	SH2D2A	CACTCACCGCAGTGTAGCCC
6911	SH2D2A	GGCTTGGTTCCAGAAGACCC
6912	SH3BGRL2	CCTACCTTTGATGCCAACCG
6913	SH3BGRL2	GATGTACAAAAACGTCCCCC
6914	SH3BGRL2	GCTCACCGCCACGAAGCCCG
6915	SH3BGRL2	AGCCACTTACTCCACAGTAT
6916	SH3BGRL2	GAAACCACGGTTGGCATCAA
6917	SH3BGRL2	CGTGTTCATCGCCTCTTCCT
6918	SH3BGRL3	CCGACGTGCTGTAGACGCGC
6919	SH3BGRL3	CCTGCGCGTCTACAGCACGT
6920	SH3BGRL3	GCGCATCCAATACCAGCTAG
6921	SH3BGRL3	AGATGTCCACTAGCTGGTAT
6922	SH3BGRL3	CCAGGACTATGAGCTCTTCG
6923	SH3BGRL3	GCTTGACTCAAGCCAGCTTC
6924	SH3D19	ACTTACCTCGGTGGCAACCT
6925	SH3D19	TAATCGATCCTCTTCTGACA
6926	SH3D19	TTGCAGGAATCCAACCACCC
6927	SH3D19	CTACCCCCTCGTCCCAAACC
6928	SH3D19	CTCCCTGCTGAAAAACCTAT
6929	SH3D19	GTACTGAAAGTGTTTCCAAT
6930	SHMT1	CACCCGAAGCTGATCATCGC
6931	SHMT1	TCAGGTGGCCCCCATCCGGA
6932	SHMT1	TTTCGCCAGCCGAGCAGTTT
6933	SHMT1	GGAACCCCATGGGCGCATCA
6934	SHMT1	TCACCTGCGATGATCAGCTT
6935	SHMT1	TCGGCTGGCGAAATTCTCCG
6936	SHMT2	AGCACTCACGTTGAGCTTAT
6937	SHMT2	GTTGCTGTGCTGAGCCCGAA
6938	SHMT2	CGAGTACTTGTTGTTCAGAC
6939	SHMT2	CTTCTGCAGCCGAGCTGCGC
6940	SHMT2	TGACGTCAGACATGTAGCCG
6941	SHMT2	TCCCACGGCAGATACTATGG
6942	SHPRH	CTCAACACAGGGATCAATGC
6943	SHPRH	CGAGAGATTGTTACATCTGA
6944	SHPRH	CTCTACTATAATCCATATAC
6945	SHPRH	TCGTCAATGTCTTGTCCCTC
6946	SHPRH	TTACACAATTCTATTATTCC
6947	SHPRH	TCAGCTCATGAAGAAAGTAA
6948	SI	AACCTTGAACCGGAAACGAT
6949	SI	ATCAGACACCCAATCGTTTC
6950	SI	TGATACGTTGTATGATGTGA
6951	SI	ACCTCTGTTGGGAATTGTTC
6952	SI	TTATCAACGAAGAAGCACCA
6953	SI	GTAGTCACACGAGTAGTAGC
6954	SKA3	AGTTCTACCAAACCCTCCAC
6955	SKA3	AGGCCATAGATACAGAATCC

6956	SKA3	TTATAGTTGTTCACTGCCTG
6957	SKA3	CCACGTGTCAAGAAAAATTC
6958	SKA3	AAAAACATTATCATTGAGCC
6959	SKA3	TCTTCTTGATAAAGCAAGAT
6960	SLC10A6	CTTCAGATTGGGGCCGTTGT
6961	SLC10A6	CTTCTGGTGGTCGCAGTTGC
6962	SLC10A6	TATGACAACCTGTTCCACCG
6963	SLC10A6	ACCATTCCTTATCAGAACAT
6964	SLC10A6	GTAATTCACATAGACACCAA
6965	SLC10A6	TGTGTGCCTGACCATTCCTG
6966	SLC11A1	TGCACCCACCGTAGTTATCG
6967	SLC11A1	CCAGACGTGCAGCCAGTCGC
6968	SLC11A1	TTCCTCTTCCTCGATAACTA
6969	SLC11A1	TGGACTCCTTATAACCATTA
6970	SLC11A1	GGGCTTGCTCTGCCAGCGAC
6971	SLC11A1	CTCCGTAGGGCTGCGGAAGC
6972	SLC11A2	TGAGAAGATCTCCATTCCTG
6973	SLC11A2	TACTTACTTAGTTCACAGTG
6974	SLC11A2	CATGGGGAGTCTGCCAGTCT
6975	SLC11A2	CCATCTGACATCTTCTGTTC
6976	SLC11A2	CCTGAACAGAAGATGTCAGA
6977	SLC11A2	CTTCAGCTGCTTCTTCCTCA
6978	SLC12A2	CATAGCAACTGCAACAGCGT
6979	SLC12A2	TACGTTGTATGTTAAACATT
6980	SLC12A2	ATACGTGACCACAGCATCTC
6981	SLC12A2	CCAATTGCACCACCAAATTC
6982	SLC12A2	CGTGAAGTTTGGCTGGATCA
6983	SLC12A2	ATTGTGGGTCAAGCTGGAAT
6984	SLC12A7	CGGCAGGTAGACGCCGATGA
6985	SLC12A7	TCTGTCGCAGGCTCCGCGCA
6986	SLC12A7	GTCCTCGCTGCTCAACAAGC
6987	SLC12A7	GGCCAGCTTGTTGAGCAGCG
6988	SLC12A7	ATGAGTGCGATCGCTACCAA
6989	SLC12A7	AGCGATCGCACTCATGGAAA
6990	SLC16A12	AGCCGTCTCCGTTCTGTTTA
6991	SLC16A12	GCTTATGGTATCGCCATGTC
6992	SLC16A12	GACTCGTGGCAAATGAGCTC
6993	SLC16A12	GGTGGCTTGCTTGCATCTAC
6994	SLC16A12	ACTCTTCCAAGATCATAACT
6995	SLC16A12	CAACAGCCAAGTTATGATCT
6996	SLC18A2	CTATTATGATAACTCGACTA
6997	SLC18A2	TGCAGGAGAGCCGCCGCTCG
6998	SLC18A2	GATGCAGAATCCCGCAAATA
6999	SLC18A2	CATAGTCGAGTTATCATAAT
7000	SLC18A2	ACAGGATGAGCTTCCGCGAG
7001	SLC18A2	CTATCCAATTCCCATATTTG
7002	SLC22A11	CAAAAGCGGCCACGAACCGC
7003	SLC22A11	CCGCAGTAGATGACGAATGT
7004	SLC22A11	CATGGCGTTCTCGAAGCTCT

7005	SLC22A11	TCCTGGTGGGCTCCTTTATC
7006	SLC22A11	CTGGAAGAGGCCCACGCCTC
7007	SLC22A11	AGCCAGTCCATCTTCATGTC
7008	SLC22A2	CCATTTCCCCAACCTATACG
7009	SLC22A2	GGTGCATACCTGAGTCTCCC
7010	SLC22A2	GCGGAGATATCGGAGAACAG
7011	SLC22A2	AAGCCACCCCAGCTAGCACC
7012	SLC22A2	ATCTCTACCCGCCTCCCTTC
7013	SLC22A2	AAAAATTAACATCCACGTAT
7014	SLC25A11	ACCGGCCATCGGCAGTCATG
7015	SLC25A11	CTTATCCGCATGACTGCCGA
7016	SLC25A11	ACGCCCTGATTCGAATCACC
7017	SLC25A11	CTTCCCGGGTGATTCGAATC
7018	SLC25A11	CTTCCCCGCTCAACTGCATC
7019	SLC25A11	GACGACGACGGCCCGAGCCA
7020	SLC25A22	ACTTCAGCGAGGCGTCAACG
7021	SLC25A22	ACATGCTCGTGTACACGCGC
7022	SLC25A22	GATGCCGCCATTGATGAGCT
7023	SLC25A22	TGACGCCTCGCTGAAGTGAC
7024	SLC25A22	GTACATGCCGAAGTAGCCCT
7025	SLC25A22	CATCAAGACCGTCCGCTCCG
7026	SLC25A48	GGCCTCTTACCGTCCCGAAA
7027	SLC25A48	GGGTCTTCAGTAACACGCAG
7028	SLC25A48	CTCTCCATAGACTCGCCTGC
7029	SLC25A48	CGTAGCCAACGCCAGCCTGC
7030	SLC25A48	GGTGCAGCCAGTGTCATCGT
7031	SLC25A48	TGTACCTTGACTGTGTCCAG
7032	SLC28A3	CAACTCTTCGGCACATCATC
7033	SLC28A3	ATCAATTCGATGTTCGTATT
7034	SLC28A3	GCCACGGTGATCACAAAAAG
7035	SLC28A3	CTTGAGAACGAGAACACATC
7036	SLC28A3	ACACAGTATGTGGTTTCTGT
7037	SLC28A3	GATGAGGAGATGCAACAAAA
7038	SLC2A1	CTTCGTGTCCGCCGTGCTCA
7039	SLC2A1	AGGCTTCGTGCCCATGTATG
7040	SLC2A1	CCTCGTTGCGGTTGATGAGC
7041	SLC2A1	GCTGACGGGTCGCCTCATGC
7042	SLC2A1	CTCCTACTTACCGGCCAAAG
7043	SLC2A1	TGCCCGTACCACTCTTGGCC
7044	SLC30A9	CCCTGTAGTCATCCATATAT
7045	SLC30A9	ACCAATATATGGATGACTAC
7046	SLC30A9	CAGAGAGAAAAAATTGCGTA
7047	SLC30A9	CAAGAACCTCTCCAAGTAAG
7048	SLC30A9	TATTTACCTCTTACTTGGAG
7049	SLC30A9	TGCACAGATCTTTGGAAGTT
7050	SLC35A3	CTGAAGAACAACTACTATCC
7051	SLC35A3	CTGGATAGTAGTTGTTCTTC
7052	SLC35A3	TCAGCTGGTTCTCAATTTGT
7053	SLC35A3	GCAAGTTTAAGTGTTTCCAT

7054	SLC35A3	CTTAAACTTGCTATTCCATC
7055	SLC35A3	AGATTCTCAGCTTGATTCTA
7056	SLC35F1	CGGCATCGTTGTCTGCATCC
7057	SLC35F1	CTTCCTGCTGATCCGGTACA
7058	SLC35F1	GCCAGCCCCGCCGAACCATG
7059	SLC35F1	ATCTTCGTCGTAAAATTGCC
7060	SLC35F1	GGATCCGCAAAGTGCTGAAC
7061	SLC35F1	TACTGGTCAGAGTTGTGTAT
7062	SLC35G5	CTCGGTGGCTACGAGTGGTG
7063	SLC35G5	ACTCGTAGCCACCGAGACCC
7064	SLC35G5	ATGTGCCTACAGTGCAGTTC
7065	SLC35G5	CACCACCTGAACTGCACTGT
7066	SLC35G5	CCTAGGACTAATCATCATTC
7067	SLC35G5	CACCAGCGCTGCCAGCCCTC
7068	SLC38A5	ACTCACCCCACAGCATCCGA
7069	SLC38A5	GGAAGAAGATGACCCCCGTG
7070	SLC38A5	GATGAATGGAGCCCTCCCTT
7071	SLC38A5	TTCGAGGGGAAGACATCGTT
7072	SLC38A5	GAAGCGCAATGCACAGCAGC
7073	SLC38A5	GAGCAAGCCGGTCCAGTTCA
7074	SLC39A8	TGCTTGGGCCGATCCTCACA
7075	SLC39A8	GTCAGTGACGATTATTAATC
7076	SLC39A8	GATTAATAATCGTCACTGAC
7077	SLC39A8	CTCAACATAACTGTCGACTT
7078	SLC39A8	AGTCGACAGTTATGTTGAGA
7079	SLC39A8	TTCAATTGCTGTAAGACTGC
7080	SLC40A1	GCCCGTAGACTGCTGTCAAA
7081	SLC40A1	CATTTAAGGGAGATCGGATG
7082	SLC40A1	AACTTACGAGAACCCATCCA
7083	SLC40A1	CTACTGCAATCACAATCCAA
7084	SLC40A1	AACATTCTGTACCACCAGCG
7085	SLC40A1	TGTGATTGCAGTAGCAGTAC
7086	SLC45A4	CATCTTCGAAGGCGACCCCA
7087	SLC45A4	GCCGCTTCTGCATGTCGTAC
7088	SLC45A4	CAAAGAGGACGCCAACGCAG
7089	SLC45A4	GCCGGTTGGGGACATCGCCG
7090	SLC45A4	GCCCATCCGTGCCTATCTGC
7091	SLC45A4	TACCGATGGCAGAGCCGTTA
7092	SLC4A5	GAATGCCCTCCTATCCACAT
7093	SLC4A5	TAACCACAGGAGGAGATTTC
7094	SLC4A5	GGCCTGAAAGATGTCCCTTC
7095	SLC4A5	GCAGCATGACGGAGACCAGA
7096	SLC4A5	GTGACCTCACCTTTCTGATC
7097	SLC4A5	CCCCACCCTCTTTACAGAGA
7098	SLC52A1	TTCACCAGTGCCGTGACCAA
7099	SLC52A1	CACGCCATTGGTCACGGCAC
7100	SLC52A1	CTGAATGGCAGCACCCACGC
7101	SLC52A1	CTCCCTTGCAGGTGCTGTCG
7102	SLC52A1	CCTTTCTGCTTTGAAACACG

7103	SLC52A1	CAGCACCAGACGGCCCAGCG
7104	SLC52A2	GCCACCAACGCGCTGACCAA
7105	SLC52A2	TGGCGTGTTCTCCTACGTGA
7106	SLC52A2	TGGTAGGCCAGACGCCCGTA
7107	SLC52A2	TGGGTCAGCACCGGACGGGC
7108	SLC52A2	AGCACCCACGCCCGCCCGTC
7109	SLC52A2	CACGAGGACCACCCCCGCCG
7110	SLC6A14	AATTTCACTGCCGTTGATGC
7111	SLC6A14	AACTGTAGCAGATCACCAAT
7112	SLC6A14	CTGGGACAATTTGCTAGCTT
7113	SLC6A14	AATGATGAGAATCAGGACCG
7114	SLC6A14	AGCTTCCCAGTGAACAATAT
7115	SLC6A14	CCATATCTGACCTACAGCAA
7116	SLC9A5	AATACTGACCGACTCCCTTC
7117	SLC9A5	GAGATGTTACTTTCCGAGAC
7118	SLC9A5	GCTCAACGATGCTGTCACCG
7119	SLC9A5	CAGGCTATTTCATGCCTAGC
7120	SLC9A5	CGGGGTCCACCGCCGAGATG
7121	SLC9A5	GCAGGCCACTGACTACCTGA
7122	SLCO5A1	ACAGCGAAGTCAGCTCCGTG
7123	SLCO5A1	CTGACTTCGCTGTCCCACGG
7124	SLCO5A1	GGAAGCTGCAACGTTAATTG
7125	SLCO5A1	TGCTCCCATGACATACATGA
7126	SLCO5A1	TACATACTTTAACCCTTGTC
7127	SLCO5A1	CAGAATGACCCTCGTTTCAT
7128	SLITRK2	GTTACAGGAGATCCGAACGG
7129	SLITRK2	TGGCGTCGAGATTCATAGGA
7130	SLITRK2	CTCAGTGTGTTAACCGTGGC
7131	SLITRK2	AAAGCTGATAGATTCGATAC
7132	SLITRK2	TAAAGATGCTGAGCGGCGTT
7133	SLITRK2	CCTGTAACCCGTTGTTACCT
7134	SLITRK6	TTAACCCATCTAGATCTTCG
7135	SLITRK6	CGAACACATTGGCCGAATAT
7136	SLITRK6	CATTAATGTACTCACGTCCA
7137	SLITRK6	TGCAAAGTCCTATCCCCATC
7138	SLITRK6	GCTAACCCAGATTGACCTTG
7139	SLITRK6	CCATTAACTTCAGCTCTTCG
7140	SLPI	ACTTCAAGTCACGCTTGCAC
7141	SLPI	ACTCAGGTTTCTTGTATCTA
7142	SLPI	AAACATTGGCCATAAGTCAC
7143	SLPI	CATTTGATGCCACAAGTGTC
7144	SLPI	AACTCTGGCACCTTGGGCTG
7145	SLPI	GCCTTCACCATGAAGTCCAG
7146	SLX1A	CCTTACCCGAAGGGCGGCCA
7147	SLX1A	GAGATGGTGCTCGTCGTGCA
7148	SLX1A	GGGACCTCACCTGGATGGTC
7149	SLX1A	GCCCGCAGTTTGAGTGGGCT
7150	SLX1A	ACTGCACTCACCAAGGGCAT
7151	SLX1A	GCCTGGGTCAGGATGAAGAG

7152	SLX1B	CCTTACCCGAAGGGCGGCCA
7153	SLX1B	GAGATGGTGCTCGTCGTGCA
7154	SLX1B	GGGACCTCACCTGGATGGTC
7155	SLX1B	GCCCGCAGTTTGAGTGGGCT
7156	SLX1B	ACTGCACTCACCAAGGGCAT
7157	SLX1B	GCCTGGGTCAGGATGAAGAG
7158	SMAD5	TCCAGCAGTAAAGCGATTGT
7159	SMAD5	GGATTGAATTCATTATGACG
7160	SMAD5	TCATCATGAGCTAAAGCCGT
7161	SMAD5	GGTTCCTAAACTGAACCAGA
7162	SMAD5	TACTTACAGAGGCAGATTTC
7163	SMAD5	AAGTGAGTCTCCCGAATAAC
7164	SMARCA1	TTAATTTCAGCTACCGCCAT
7165	SMARCA1	GCTGACAATATGCTCATCAG
7166	SMARCA1	GGTAGTTCTGGATAAACTAT
7167	SMARCA1	CTTCTTGCTCTGTGCGCCTA
7168	SMARCA1	GAAGAGTCTGCCACCTAAAA
7169	SMARCA1	TGAGTAAGATGCAACGAGAA
7170	SMARCA2	CTTGTCATGTATACCATCGA
7171	SMARCA2	CTCCCATCCTATGCCGACGA
7172	SMARCA2	GGTCCAGGCCTTGCGGTTTC
7173	SMARCA2	TCGCATACCAGTGCCCTTCA
7174	SMARCA2	AAGTCTGTGGACCCCATCGT
7175	SMARCA2	CCGCTCTTGCAGAATTTCCA
7176	SMARCA4	CTGGCCGAGGAGTTCCGCCC
7177	SMARCA4	CATCCCGGGGGGCACGCCCG
7178	SMARCA4	CCTGTTGCGGACACCGAGGG
7179	SMARCA4	TTGTCCTGAGGGTACCCTCC
7180	SMARCA4	CGGCACCTCCAAATTACAGC
7181	SMARCA4	GGCGTGCCCCCCGGGATGCC
7182	SMARCA5	AAGCAGAACTTACTATCCGT
7183	SMARCA5	TTCAAATCGAGTGCAAACAT
7184	SMARCA5	CTGAGAGATTATCAGGTCCG
7185	SMARCA5	AATACTCGAATCTATTTGCC
7186	SMARCA5	ACACCGTAGAACAGAGCAAG
7187	SMARCA5	AAATGAGCCAGTTTAATCCT
7188	SMARCAD1	ATCATCATTTCTACGTGCCA
7189	SMARCAD1	TCTGAAGATGAAGAGTCCCA
7190	SMARCAD1	ATGCTGAAGGGGAAGTTAGC
7191	SMARCAD1	AATTTCTCTTAGTGCTGAAG
7192	SMARCAD1	TCATATTTCAAAAATCAAAG
7193	SMARCAD1	TGCTAAACTTCAGACTTTGA
7194	SMARCB1	AACTACCTCCGTATGTTCCG
7195	SMARCB1	TGAGAACGCATCTCAGCCCG
7196	SMARCB1	CATCGATCTCCATGTCCAGC
7197	SMARCB1	TCTTCTTGTCTCGGCCCATG
7198	SMARCB1	CATGCTCCACAACCATCAAC
7199	SMARCB1	CTAGTCGCCTCCAGAGTGAG
7200	SMARCC1	AGATATCATCAAACGACATC

7201	SMARCC1	GGCGCCTTATGTCACATTCT
7202	SMARCC1	GCCAACTTCAGATCAATGTC
7203	SMARCC1	AGCCCCAAGAATGTGACATA
7204	SMARCC1	TGCTCCTACCAATAAAACAC
7205	SMARCC1	TGGGAAGCATGTCACCAACC
7206	SMARCC2	GGCCTCGTAGTACTTCACGT
7207	SMARCC2	TGACCCAGTTCGACAACGTG
7208	SMARCC2	TGCACCGCTCACTAAACTGC
7209	SMARCC2	ATTGTAGCAACTGTACAACC
7210	SMARCC2	GAAATTGAGCCCAAACTACT
7211	SMARCC2	GGACATTATCAAGAGACACC
7212	SMARCD1	TGTTCCTAAGATTCGTGAAC
7213	SMARCD1	TACCTTGATGGGACGTTTCA
7214	SMARCD1	AGCTACTCACTTCTACCAGA
7215	SMARCD1	TTTGTCCAGTTCAATCACCA
7216	SMARCD1	CTGACAAAATTCTACCTCAA
7217	SMARCD1	CAGTGCAAAGAAAAAGAAGA
7218	SMARCD2	CTCCACATCGATGTCGTAAC
7219	SMARCD2	AGCCTGTTACGACATCGATG
7220	SMARCD2	CTCCATCCGCTTGCGAGCAA
7221	SMARCD2	ACCAGACCATTGCTCGCAAG
7222	SMARCD2	TCCTCCCCAGGTTAAAGAGG
7223	SMARCD2	GGCGATCTCCTGCTGATTGG
7224	SMARCD3	GTCCACCCGCTTCCGCATGA
7225	SMARCD3	CCCTCCGGCAACTTCGTCCG
7226	SMARCD3	ATGGCCGCGGACGAAGTTGC
7227	SMARCD3	CAGCGCCCCGGGATGCCGTC
7228	SMARCD3	CCTTCTCCAGATTCGGGAGC
7229	SMARCD3	CGGGGGCGCTGCTCGCTTGC
7230	SMARCE1	TCGACAGAGACAATCTCGCA
7231	SMARCE1	AGACGACGAGAACATTCCGA
7232	SMARCE1	AGTGGGTTACCTGTCTCCAT
7233	SMARCE1	AAACTTACTGTTGCAGGAGC
7234	SMARCE1	TTACCATCTGGATCTTCAGC
7235	SMARCE1	GTGGGAGGTGGGGCATAAGA
7236	SMIM19	ATTATACTGTTCACGAAGCC
7237	SMIM19	GCTTATCGTGTCATAAAAGT
7238	SMIM19	GATTCGTTTAAGACAACAAC
7239	SMIM19	AACTATCAAGTAAACATTGG
7240	SMIM19	TGACAGTGTGCAACTCTCAT
7241	SMIM19	AGAGTTGCACACTGTCAGCT
7242	SMKR1	CGTTGTGGGCGATGTAGTAG
7243	SMKR1	TCTTCCCATGAGACTTGCCC
7244	SMKR1	GCTGACTGCCTGCATCTGCG
7245	SMKR1	GTCTTTGAAAGCCAGCTAAA
7246	SMKR1	GGGCTGAGAATGTCCACTTC
7247	SMKR1	AAAGGCCAGGGCAAGTCTCA
7248	SMTNL2	ATCGGCAGGGGGAGCGTCGC
7249	SMTNL2	TGCGACGCTCCCCCTGCCGA

7250	SMTNL2	AGAAGAATTCCTCTTTCACG
7251	SMTNL2	ACTGCCCCATAGCCAGAGCT
7252	SMTNL2	CAGCCCTACCTGCCCCCGGC
7253	SMTNL2	AATGGGCACCAGCCGGGGGC
7254	SMYD1	GGCTGGCGGCGCGCATCATG
7255	SMYD1	GCAAGTCGTCCACGGACACC
7256	SMYD1	GGGGAAGATGCCTACGCCCA
7257	SMYD1	CTGCGACCGCACCTGCCAGA
7258	SMYD1	ACTTGCACTGCCCACAGCGA
7259	SMYD1	GCCTGGTGAACCATGACTGT
7260	SMYD3	CTTGCACACCGTGTACGCCA
7261	SMYD3	AAAAGTTCGCAACCGCCAAG
7262	SMYD3	CCAGACTCCGTTCGACTTCT
7263	SMYD3	TTGCATTCCCGCTTGTGGTC
7264	SMYD3	ATACTGTAGTGCTAAGTGTC
7265	SMYD3	ACACTTAGCACTACAGTATT
7266	SNAPC4	GGGATCGTCCTCGTCATTGC
7267	SNAPC4	CCAACAGTTACCGAGATCGG
7268	SNAPC4	GGGCGAAGCCAGCAATGACG
7269	SNAPC4	CTTCATGAAGCCGTATTTCA
7270	SNAPC4	AAGTGCCCCATGTATGTGCT
7271	SNAPC4	TCCTGCCGATCCCCCGATCT
7272	SNAPC5	TCACCTTGAGGCGGTTCAGC
7273	SNAPC5	CGACCAGCTGAACCGCCTCA
7274	SNAPC5	CTTACATCATGTGACTGTTC
7275	SNAPC5	TTCTAGAACTGATCATTGAT
7276	SNAPC5	GAGCACAAAGAGTCATGTGA
7277	SNAPC5	ATCAATCAACCAAACAACCC
7278	SNRNP70	TGCCTTCAAGACTCTCTTCG
7279	SNRNP70	CGCCACGAAGAGAGTCTTGA
7280	SNRNP70	AACTCGTGCTGAAACCCGAG
7281	SNRNP70	GTTTCAGCACGAGTTGGAGG
7282	SNRNP70	AGAGACGGGAAAAGATTGAG
7283	SNRNP70	TCACAATGATCCCAATGCTC
7284	SNRPA1	TCAAATCGCTGACTTACCTA
7285	SNRPA1	TCTCACCAATAATAGTCTCG
7286	SNRPA1	CAGTTCCACGAGACTATTAT
7287	SNRPA1	TTGTAGTATCCTAAGAAATC
7288	SNRPA1	TAGTGAACAACAACAGAATA
7289	SNRPA1	ACAGGGTGATCTGGACCCTC
7290	SNX13	TTAGTGTATAATACCAATAC
7291	SNX13	TCTATCAATCTTAATAGTCC
7292	SNX13	AAGGCTTCCCCATCCCCATA
7293	SNX13	TTCAGGCCAGTCTATCCATA
7294	SNX13	CACTCATTCAGTTTGCTACT
7295	SNX13	CTGAAACTCACTTGCTGGAG
7296	SNX7	ACTTTCTTCAGACATCTCGT
7297	SNX7	GCGGATGTCTGGACTCTCAG
7298	SNX7	TTCTTAGGATGCCTCATTGA

7299	SNX7	TCTGTGGTCAGCGTCAGAAG
7300	SNX7	TAGAAACTTTCATTACGTAT
7301	SNX7	GACGATATCAAGATTTCCTT
7302	SORT1	CCGGGACTTCGTCGCCAAGC
7303	SORT1	CTGCGGACGGCCTCTCGCGC
7304	SORT1	GACAGTCCAAGCTATATCGA
7305	SORT1	ATCTCACCTTCGATATAGCT
7306	SORT1	TCAGTATCCTTGTCCTGGGT
7307	SORT1	CATGTGTTTGATGATCTCAG
7308	SOS1	TCTCGAGCATACGATTCATA
7309	SOS1	CCGAAGTGCTTCAGATGTAG
7310	SOS1	CTTAGTCAGTTATCAAAGCC
7311	SOS1	TCATCATTAGACTCGAGAGT
7312	SOS1	CCCAATCAGCTATTGAAAAG
7313	SOS1	TCCCTCATCCAATTGATAAA
7314	SOS2	CAAAATCCATCCTTCGTTGA
7315	SOS2	CTAGTACAGCCACAATATAT
7316	SOS2	CCATGTATCCCTATATATTG
7317	SOS2	AGCAGATTGTGCATCAGCAA
7318	SOS2	TTCAAAACAGCTGCCAGCTA
7319	SOS2	AGCAGTCCTCATCCCTTAGC
7320	SOX6	GCTCCCGTAAACTGATCAGC
7321	SOX6	TTACCCAGCTGATCAGTTTA
7322	SOX6	TTTAATAGATTCCTCCTGCA
7323	SOX6	GTTGGTAGCTCCTCAGAGTG
7324	SOX6	CCAGTCAGCATCTTGTTGAA
7325	SOX6	CTAAATACCAGTGAACTTCT
7326	SOX9	CGTGTTCTCGGTGTCCGAGC
7327	SOX9	CCCTATCGACTTCCGCGACG
7328	SOX9	CATGAAGATGACCGACGAGC
7329	SOX9	CACACGTGGCCCGCGCTCGC
7330	SOX9	CGACTCACCCGAGTGCTCGC
7331	SOX9	TTCACCGACTTCCTCCGCCG
7332	SPAG5	CCTCCTAACCTGCGGGCTGA
7333	SPAG5	AACACCCTCTGCCTAATGAC
7334	SPAG5	AAGGTCCTGTCATTAGGCAG
7335	SPAG5	TTTCACCCGAGTAGCATCAA
7336	SPAG5	CTTCTTTAGACTCTTGTAGC
7337	SPAG5	TCATAATACTCATTTCTGCC
7338	SPAM1	TCTATGTGCGCAATCGAGTT
7339	SPAM1	CCTCAGTATAATGCGAAGTA
7340	SPAM1	AGTGTGACGTTGATTATGTA
7341	SPAM1	AGCCCCCGAATAAACGCCAC
7342	SPAM1	AAAACCGACACTTGAAGACC
7343	SPAM1	CCATACTTCGCATTATACTG
7344	SPANXA1	ATGGACAAACAATCCAGTGC
7345	SPANXA1	TACCATCTCGTTGGCCTCGT
7346	SPANXA1	CAGATGCCGGAGACCCCAAC
7347	SPANXA1	GTAGCTTCTTGCTACTTTGC

7348	SPANXA1	CGTCCCCTGTGATTCCAACG
7349	SPANXA1	GACGCTCCTCTTCACCCCGC
7350	SPANXC	TACCGTCTCATTCACCTCGT
7351	SPANXC	ATGGACAAACAATCCAGTGC
7352	SPANXC	CAGATGCCGGAGACCCCAAC
7353	SPANXC	GTAGCTTCTTGCTACTTTGC
7354	SPANXC	CGTCCCCTGTGAATCCAACG
7355	SPANXC	GACGCTCCTCTTCACCCCGC
7356	SPEN	GTTCATTATAATCCGTGCGT
7357	SPEN	CCGTCACTTCATGCACGAGA
7358	SPEN	TATTGCAGAAACGCATACTG
7359	SPEN	TCTTGGAAATAATCGCCTCA
7360	SPEN	AAAGCCTTGACTGCATCAAA
7361	SPEN	GTAACTTCAGTGCAGATACA
7362	SPG11	GCGGGTTCTACCGATGCTGT
7363	SPG11	GTCGCGAGTGCTGCTTCCGC
7364	SPG11	TGACAATCACTGCAACATCG
7365	SPG11	ATTTAAGTTAAGAGCAACTG
7366	SPG11	CTAATTGCAGCATCTCTTTC
7367	SPG11	ATCATGTCCACTGCCTGTGC
7368	SPINT1	CATCCGTGTCACCCCGCAGT
7369	SPINT1	CTTCAAGTCTATGCCTGCCC
7370	SPINT1	ACCTGGATGGCGCCTTTCCA
7371	SPINT1	GACTAGCTCAGACCACCCAG
7372	SPINT1	TGAGTCCCCACAGTTCCACC
7373	SPINT1	CTCTCTCCCAGGCCCCTCCA
7374	SPOCD1	GCATCGAGCTCATCCGCACC
7375	SPOCD1	AGATGTCACCCCCTACGACC
7376	SPOCD1	GTCCGAGCTGGGGTCGCCAA
7377	SPOCD1	GGCCCTTGGCGACCCCAGCT
7378	SPOCD1	AGCACACAAGCTTCACTCCA
7379	SPOCD1	GAGGAAGTGAAAATACCCCA
7380	SPRYD7	GCCAGCAAACAGTCTTCCGC
7381	SPRYD7	GTCCCTCGTATACCTGATGC
7382	SPRYD7	TCCCTGCGCGATGGCCACCT
7383	SPRYD7	AGTATACATTTAATTCGACA
7384	SPRYD7	TCACCAGACTGTGCATATCT
7385	SPRYD7	GAATTCAAAATCCAGTCCAC
7386	SPSB2	GGAACTCTGGGCTACGCTAT
7387	SPSB2	GCATCCGCTACCTGGGCGAA
7388	SPSB2	CCTGTGTGTGCGCCACAACC
7389	SPSB2	CCAGGTTGTGGCGCACACAC
7390	SPSB2	GGTTCCAACCGTGGCGCCGC
7391	SPSB2	TGGGCAGCAACAGCGAGTCG
7392	SPTBN2	CCGCATGCGGATCCACTGCC
7393	SPTBN2	CTTTGAGAGGTCTCGCATTA
7394	SPTBN2	GGGACCTGTACAGCGACCTC
7395	SPTBN2	CGCTGTACAGGTCCCCCACC
7396	SPTBN2	ACAGTGACATCAACAACCGC

7397	SPTBN2	GACCATCATCCTTCGATTCC
7398	SPTY2D1	TTACTGACAGTCCGTCCAGC
7399	SPTY2D1	ACTCTGCGTGATTGTACTCG
7400	SPTY2D1	TGATTATGCCTTACGTTACA
7401	SPTY2D1	ACTACTTTCCATGTAACGTA
7402	SPTY2D1	AAGCTTGTACAGCTGCTGAT
7403	SPTY2D1	GAAGAGGAGCTGAGACGAAA
7404	SQSTM1	ACGCTACACAAGTCGTAGTC
7405	SQSTM1	CGACTTGTGTAGCGTCTGCG
7406	SQSTM1	TTCACACTCGGATCCTCCGA
7407	SQSTM1	AGGGCTTCTCGCACAGCCGC
7408	SQSTM1	AATGGCCATGTCCTACGTGA
7409	SQSTM1	CACTCTCCCCAACGTTCTTC
7410	SRA1	TGATGACATCAGCCGACGCC
7411	SRA1	GACCCGCCGCAGTTCTCATA
7412	SRA1	CCGAGGATTATGAAGCCTGC
7413	SRA1	ATCACAGCCTCAGACTCGAC
7414	SRA1	TCCGCAGGCAACAAGGAACG
7415	SRA1	CAGGCATTGGAAGACTGCCG
7416	SRC	TAACCGCTCTGACTCCCGTC
7417	SRC	GTCTGACTTCGACAACGCCA
7418	SRC	CTCACGTGTTGTTGACAATC
7419	SRC	GCTCGCCTTTCTTGAAGGAC
7420	SRC	GCACTACAAGATCCGCAAGC
7421	SRC	CTCAATGCAGAGAACCCGAG
7422	SRCAP	ACTTTGCTCAGGAGCGCCGT
7423	SRCAP	AGCAATCCGAGTCTCGATCT
7424	SRCAP	GGAAGCAATTCGACGCAGCT
7425	SRCAP	GCGTCGAATTGCTTCCACCA
7426	SRCAP	ACTTTCAAACCTGTGTCTGT
7427	SRCAP	GCTCCCAGTCCTACAGACAC
7428	SRCIN1	GGCTGGTGCACACGTCCGAG
7429	SRCIN1	CGCATCGTCCGCAGACAGCA
7430	SRCIN1	ATGGGGAACGCTCCGTCCCA
7431	SRCIN1	CGACATGGTCTCCAGCGACT
7432	SRCIN1	CTACGCCTCCGCCGAGTCGC
7433	SRCIN1	CGGAGTACCCGCGGGAGTAC
7434	SRGAP2D	AGCCCAGAATGAGTACTTGC
7435	SRGAP2D	CCGTGGAGTCAGGGGAGCAT
7436	SRGAP2D	TCAGCACCAAGCCAAGTACA
7437	SRGAP2D	CCCATGCTCCCCTGACTCCA
7438	SRGAP2D	GCCTCACCATATCCCCCATG
7439	SRGAP2D	CCACAGCAGTGTTGTGACTT
7440	SRM	GTCAGTGATGATCACGTCGA
7441	SRM	GGTCCAGTGTGAGATCGACG
7442	SRM	TCCGCCCGCAGTAAGACCTA
7443	SRM	ACTCGTCTCTCTCCGTGCAC
7444	SRM	CCCCGCCGAAAGTCTCTTCA
7445	SRM	CTCGAAGCTGACCCTACATG

7446	SRRM5	GCCGGCCGTAACTCTTTCCC
7447	SRRM5	AAGAAGAGTTACCGCCCACC
7448	SRRM5	CGTCGGCTTGACTGTCATCC
7449	SRRM5	GGGGTCGCTCTCCTTGCTAG
7450	SRRM5	GCCAAGTGTCAAACCCCGAC
7451	SRRM5	CTGCTGCTGGGCCGGTTAGA
7452	SRSF6	GTATCTCAAAAAGTCGCTCC
7453	SRSF6	TAGCCGTCGCGATCGCGACG
7454	SRSF6	GGCCCGCGTCGCGATCGCGA
7455	SRSF6	ATAGGTCTCGATCTAGAAGA
7456	SRSF6	TACAGCAGTCGGAGAACATC
7457	SRSF6	AACCTATGCGGATGCCCACA
7458	SRSF8	TCGTCGCGCAGAGCTCGATT
7459	SRSF8	CTGTAGCGCGACCGGCTGTA
7460	SRSF8	GCGACGATCCAAGTCCTCCT
7461	SRSF8	AGTAGCCCCTCGCGTCCAGC
7462	SRSF8	ACGACCTAAGATTACGTTAT
7463	SRSF8	TTCCTCTTGGATACCCGGGG
7464	SSPN	ACGGACTGTACCAGCGTCAC
7465	SSPN	CGTCACATCCCGGTAAACAA
7466	SSPN	CTTCTTCGGCTCCATGTCGT
7467	SSPN	CCTGGGCATCGCCGTGACCG
7468	SSPN	TATGCTTTGTGTCTCATATC
7469	SSPN	ACTGTGTGAGCTGCGAATAG
7470	SSRP1	TATCTGGCGCCGTGTTGCTC
7471	SSRP1	TACAGAATGATGGTCGACTG
7472	SSRP1	CGGCTCACCAAGAACATGTC
7473	SSRP1	CCCTCTATGAGATGGTCAGC
7474	SSRP1	TCACTATCGCCTTGAGCTAA
7475	SSRP1	AGTTCCAGCCCTTCACACAA
7476	SSX3	CGTTTATTACGCATGAAAGA
7477	SSX3	ACGGAGATGACACCTTTGCA
7478	SSX3	GTGGGAAAAGATGAAAGTCT
7479	SSX3	CATCTTTCATGCGTAATAAA
7480	SSX3	AATACCAGAGAAGATACAAA
7481	SSX3	TATTGCCAAATACTTCTCTA
7482	ST20	AAGAAGCTTGAGCCTAAATC
7483	ST20	TCGTAAGAGTCAGAAAAATT
7484	ST20	GGAGTGCAATGGCGCGATCT
7485	ST20	CACCTGAACCTGGGAGACAG
7486	ST20	CACTGCAACCTCTGTCTCCC
7487	ST20	TTAAAAGTAGAAGTATGTGT
7488	ST8SIA4	ACCCGATGAGTTGCGTCTCC
7489	ST8SIA4	CACCAGGAGACGCAACTCAT
7490	ST8SIA4	TTTCACCAATGAAGAATCGC
7491	ST8SIA4	CTCTGATAAAATCATTCGAA
7492	ST8SIA4	ACATAGTGTATGACATCACC
7493	ST8SIA4	TCCAACCTTCTACATTGTGC
7494	STAG1	ACTACTGCCCATTCCGATGC

7495	STAG1	TTCAAGCTCGCTGCCAGCAT
7496	STAG1	ACCACCTCAAATAATGTGAC
7497	STAG1	GCAGGCCGTGGAAGAGCTAA
7498	STAG1	TACCTCGACATCCTGAACAC
7499	STAG1	TTCAATCCAGTCATCCACCA
7500	STAG2	CTTCAGTCGTAGAGATCCAG
7501	STAG2	TGAGCATGCAGCATACCTTG
7502	STAG2	AGTCCCACATGCTATCCACA
7503	STAG2	AGAAGACACAGTTGGATGAT
7504	STAG2	CCTTTAGGTGCTTACAGCAA
7505	STAG2	TGCCAACCTTGTTAAGACAT
7506	STAT3	AGATTGCCCGGATTGTGGCC
7507	STAT3	AGAGAACATTCGACTCTTGC
7508	STAT3	ACAATCCGGGCAATCTCCAT
7509	STAT3	GCATCACCTGCACTCTCTTC
7510	STAT3	ACTGCTGGTCAATCTCTCCC
7511	STAT3	CTGCTGCTTCTCCGTCACCA
7512	STK11	CAGGTGTCGTCCGCCGCGAA
7513	STK11	CAGCCGCCCGAGATTGCCAA
7514	STK11	CCACCGCATCGACTCCACCG
7515	STK11	TCTACCAGCCGCGCCGCAAG
7516	STK11	GAAGGGGAGCTACGCCATCC
7517	STK11	GGGGCCACAGTCGCCCGGGA
7518	STK36	TTCTCTGAGCGCCCCAATTT
7519	STK36	TCAGAACATCCTCCTCGCCA
7520	STK36	GGTGGGAATGCAGATAGTAC
7521	STK36	GGTGGTGACAGACTATGCTG
7522	STK36	CCTGAAGTTCATCCCAAAAT
7523	STK36	AGAAAGCATACCTGGTCTTC
7524	STX3	TGAGATTGCTATCGACAACA
7525	STX3	AACCCGGCCATCTTCACTTC
7526	STX3	CAAGATCTCAGAACATGTAG
7527	STX3	TGGCAAAAAGACAACCGATG
7528	STX3	TCTCAGTCGTGAGCTGCTCT
7529	STX3	CACTCACTTGGCTCTGGAAT
7530	SUCLA2	TGCCAGGGTCCTGTATTAAT
7531	SUCLA2	GATGGAAATATAGGCTGCCT
7532	SUCLA2	GCTGCTGATTCCACAATATT
7533	SUCLA2	GGCAGCCTATATTTCCATCG
7534	SUCLA2	CCATCTGAATCTTCCACCAT
7535	SUCLA2	GAACCTATTGATATTGAAGA
7536	SULT2B1	TGATGCTCTCGAGCGAGTAC
7537	SULT2B1	TGTCGTCGTCCCGCACATCT
7538	SULT2B1	GGATCCGCTCCGTGCCCATC
7539	SULT2B1	GCAGATGATCTCGATCATCC
7540	SULT2B1	TGTCCCACAAGCCCGGGATC
7541	SULT2B1	TTCCGAGATGTCATCTTCAT
7542	SUPT16H	CAACCATCAAAGTGCGAACA
7543	SUPT16H	ATATACCATCGCTGTAAAGG

7544	SUPT16H	TGCCAAATCAACTGCCTTAC
7545	SUPT16H	CAACCTTGTTCGCACTTTGA
7546	SUPT16H	CCCATCCTCCTTTACAGCGA
7547	SUPT16H	CACCTGTAAGGCAGTTGATT
7548	SUPT4H1	GTATATGCGGTGTCAGTCAC
7549	SUPT4H1	CTCTCAGGTAACTTTAAGCC
7550	SUPT4H1	TCCTGCTTCAGGAATCGTGC
7551	SUPT4H1	TGATGCATATCTACAAATGA
7552	SUPT4H1	TGCAGTCATATACCATCTCT
7553	SUPT4H1	GGTCTTTATAGCTGTGTCTC
7554	SUPT5H	GTGTCATCGTGCGACTAGAA
7555	SUPT5H	TCCCCGGCTAGGGCCGAGAA
7556	SUPT5H	GAAGGACAACCGCTTTGCTG
7557	SUPT5H	GCACGATGACACCCACAGTC
7558	SUPT5H	CAAAGCGGTTGTCCTTCTTC
7559	SUPT5H	AGGCGAAGCTTCGGAAGAGA
7560	SUPT6H	ACTCACGCGTCTGTGTATCC
7561	SUPT6H	CCCACCCCTAGCAAAAGTAC
7562	SUPT6H	AGATATTGACGACTTCATTG
7563	SUPT6H	CACCCAAATTCTCCTCAATG
7564	SUPT6H	CAAAAAAATGTCAGATGACG
7565	SUPT6H	TTGGGTGTCAAAGTCAAAAG
7566	SURF1	CATGGTGGACCCTGTCCGGG
7567	SURF1	TGTCACTGTAGGTCCAGCGT
7568	SURF1	TCAGCTTCCACTTCCGACGC
7569	SURF1	GGGCATCATATACAGCTCCT
7570	SURF1	GCAGCCCTCACCTGGGCGCG
7571	SURF1	GACCCTGAGGACGCTCCTCC
7572	SUV39H2	AATCACGTATCTCTTTGATC
7573	SUV39H2	AAGCTCACATGTAAATCGAT
7574	SUV39H2	AGTTTCACTTGATACTCTTC
7575	SUV39H2	TCCCAAGTATTTGTAGAATC
7576	SUV39H2	TCCACAAGAACCATAAATGC
7577	SUV39H2	TTAGTGTGACCCAAATCTTC
7578	SYCE1L	CTAATCAGGTCCTCTATCTG
7579	SYCE1L	AGATAGAGGACCTGATTAGC
7580	SYCE1L	AGGAGTCTAATTCCCTATGC
7581	SYCE1L	GTCTTCAGTCTTCAAAGACT
7582	SYCE1L	TTTGAAGACTGAAGACTTGC
7583	SYCE1L	GGTCTCTCTCAGTTCCTCAC
7584	SYCP3	CAATCTAGTTGACATTAACA
7585	SYCP3	GGGTGAAGTGCAGAATATGC
7586	SYCP3	GAGACTAGAAATGTATACCA
7587	SYCP3	TTCTGACTTTGTTTCAGCAG
7588	SYCP3	GAAACTGCTGAGAATATTCT
7589	SYCP3	ACCAGAAAATTGAACATGTT
7590	SYNE1	AAAAGATCGTCCACCACCAT
7591	SYNE1	GATATCCCGAGGACACCGGG
7592	SYNE1	ATATCGCCAATGTGATGCAG

7593	SYNE1	TTTACAGCGGAAACCTCCAA
7594	SYNE1	AAAAACGAACTTTCACAAAA
7595	SYNE1	TGTTAAACTGCTTGCCCTTC
7596	SYNE2	AGAAGAAAGCCTTCACGTGC
7597	SYNE2	TGAGTTTATCCAGCACGTGA
7598	SYNE2	GCTTCCCACCGAAGATGAAC
7599	SYNE2	CCAGGAACCCTGTTCATCTT
7600	SYNE2	GATCTGCTAGAAGTACTTTC
7601	SYNE2	AAAGTACTTCTAGCAGATCC
7602	SYT9	CATTACTGACCGGGCCGACG
7603	SYT9	ACTATGGCTTCGTTGTAAAC
7604	SYT9	TCAACAGGACAACGTGGATC
7605	SYT9	CATCAGCGAGACTTTCACAT
7606	SYT9	ATGGTCAGCCTGCCAGCCGT
7607	SYT9	CTGTGACTTGGCGCTGCACG
7608	TACC2	GGAGCTGGCGGAGCTTTGAC
7609	TACC2	TCTGGGATGACTTCGGGGGG
7610	TACC2	TAACCAAGAGTCACACTTGC
7611	TACC2	GGTGTTGAAGAGATGTGCGC
7612	TACC2	GTACCTGTCCCGGGTGAAGA
7613	TACC2	AACCAGCATCCTGTCCCACG
7614	TACC3	TCAGGAGGGGGTCGAACTTG
7615	TACC3	ACGAAGAACAGACGATCTTC
7616	TACC3	CTTAGAATCCTGTGCGTCTG
7617	TACC3	TAACTTCTGGTGGCGAAAAC
7618	TACC3	CAGGATTCTAAGTCCTAGCA
7619	TACC3	TCCTGGTAGACCAGTGCCCG
7620	TADA2A	CAATTAGGGAATGGACCGTT
7621	TADA2A	TCTGATAAGCCACCTTGCCG
7622	TADA2A	CATTCAGCACACTTGATATA
7623	TADA2A	TACTGCTAAAGGGACCCAAA
7624	TADA2A	GCTGAACCTGAAACAAGCAG
7625	TADA2A	TCAAATGTGCACCAAGACCA
7626	TADA2B	TTACGAGATCGAGTATGACC
7627	TADA2B	ACGGCGGGCGCTTCACGCTC
7628	TADA2B	CGTAATGCTCCATCACCTCT
7629	TADA2B	GAAGTACTGCGTGTACTGCC
7630	TADA2B	CGAGCTGAAGCGCGCCCACG
7631	TADA2B	GAGCCCGCTGATGAGCGTCT
7632	TAF10	CAACGGAGACGTGAAGCCCG
7633	TAF10	TACGTAAACCCCGTTAGATA
7634	TAF10	CATTGATGCCATACTCGCTG
7635	TAF10	ACCCCTGCCCTCAGCGAGTA
7636	TAF10	CCTCAAAGCCAGCACGGTTC
7637	TAF10	TACTCACATGCGTGGGTCTG
7638	TAF12	GCGATGCCGCGCAAGCTGAC
7639	TAF12	TCGCAAGTCTAGCACCCTGG
7640	TAF12	CAGAGCGCCAGTGGAACATG
7641	TAF12	TTCAGAGAATGGCATTGATC

7642	TAF12	AGTGGATCCTAATGAGCAGT
7643	TAF12	CACCACAGAAGCTCACAAAC
7644	TAF13	ACAGTGCGATGTATGATGTA
7645	TAF13	TATCGTCTTCTTGATTCGAA
7646	TAF13	ATATCCACTGACTCAGTATA
7647	TAF13	AGTAAGCAAGTCTTTAACCC
7648	TAF13	TGCTAGTGGGATGGCAGATG
7649	TAF13	AGGAGGTGCAGAAGGTGGAC
7650	TAF1C	TCCGATACCGGCACCCTCCG
7651	TAF1C	GAAGCGGCCCCGAGTCGTGC
7652	TAF1C	TCACCTGAGGCTGATCCCCG
7653	TAF1C	ACCTCGGTGGACACCAGCCC
7654	TAF1C	CAGTGTGAAGAAGCTGCTCC
7655	TAF1C	CAGGCAATGCAGGTGGAGAA
7656	TAF6	CACTACCTTCCCGAAGTTGA
7657	TAF6	CCACCAGAGGCGAAGCGGAA
7658	TAF6	ATTCCTTTCCGCTTCGCCTC
7659	TAF6	AAGCGGTGATTGTTTGTAGA
7660	TAF6	CTTGAAGCTAAAGAATGTCG
7661	TAF6	CTCGACATTCTTTAGCTTCA
7662	TAF6L	TGACGGTTGAGGACTTCAAC
7663	TAF6L	CCGCTCTTCTCGCTCTGACA
7664	TAF6L	CGTGTGCTATCGTCTGAGAG
7665	TAF6L	AGTCCTCAACCGTCAGCTTC
7666	TAF6L	AAAGTAGAGTTCACCCTCCC
7667	TAF6L	CCTCAGGCTGTGTGTGGTTA
7668	TAF7L	TGACACTTTGAGAACGTGCT
7669	TAF7L	TGTCCCACTAGCTGCTAAGC
7670	TAF7L	AGGAACATGCTTGTACTGTC
7671	TAF7L	GAGGCAGACGCAATATAAAC
7672	TAF7L	GTTTATATTGCGTCTGCCTC
7673	TAF7L	TCAACCAGCTTAGCAGCTAG
7674	TAF9	AGGTAAAAGACTGATCAGCG
7675	TAF9	ACTGAACCAACACTTAACCG
7676	TAF9	TGATGCAGATGATGTGCGAT
7677	TAF9	CTGTAAACCTTTGACCTGTG
7678	TAF9	GGCAACCTAGGACCTGAATA
7679	TAF9	CCATGTCCCTCACAGGTCAA
7680	TANGO6	GTACAGTCTTCGAGTTGCAT
7681	TANGO6	CATAGACGCCGAAGCCAAGC
7682	TANGO6	ATTAGCTGCAAGGCGGATCA
7683	TANGO6	AAGCAGTTCCCGGACTGCTA
7684	TANGO6	TCTATCAGCCCTTAGCAGTC
7685	TANGO6	AATGAGACCTAATGGTGTTC
7686	TAPT1	CTCAGTGCTGAACTAACAAG
7687	TAPT1	TTTGCGAATACCAAGAGAAT
7688	TAPT1	GATAGTGTGTCTAAGAAACA
7689	TAPT1	AACAAGGTCAAAAGCAAGAC
7690	TAPT1	CTCTTTCAAATGTCAAATAG

7691	TAPT1	TTTCAGTTTGTTGAAATTAA
7692	TARBP2	GTCGCCAACGGTGACCCGGA
7693	TARBP2	GCTTTGAGAAGGTCGTACAC
7694	TARBP2	ACAGGGTGTGGGCTCCCGCC
7695	TARBP2	ATCCAGGTAGCTGACGTGAA
7696	TARBP2	GCTGGGTGGACAGTTCCACC
7697	TARBP2	CCAGCATGCTCCCCCCTTTG
7698	TBC1D15	CTAGGATGCCGAAGTAATAG
7699	TBC1D15	TTCCTGAAATCAAGCCGTCT
7700	TBC1D15	TCTAGCATAGAGAATACTAG
7701	TBC1D15	GACCAAGACGGCTTGATTTC
7702	TBC1D15	AGAGATTATAGAAGTCTTAT
7703	TBC1D15	ACTTCAGAATGAAACTGCAG
7704	TBC1D19	AAAAGTACCTTTCGTGCCAG
7705	TBC1D19	CACTGAACTGAGTATCCCAC
7706	TBC1D19	CAGACCTGTTTATGCACCTA
7707	TBC1D19	CCTACCTCAAGAAAATCCTT
7708	TBC1D19	GAAATGGGAACTGATGAACC
7709	TBC1D19	AGTTCTTTCTGTTCTCCAAC
7710	TBC1D26	GCTGCCCCACGTCAGTGCCC
7711	TBC1D26	CCCGTATAACCTGCCTGCCC
7712	TBC1D26	TCTTGCAGGATGGAGATGGA
7713	TBC1D26	GGACTTACTGCACAATCCCG
7714	TBC1D26	GGGTTTCAGGGACACCGAGC
7715	TBC1D26	AGGCTCTTACCTTCACCTCC
7716	TBC1D3B	GGGCTACAGGGACACCGAGC
7717	TBC1D3B	TTGTTGTTGTAGCTTCGAAA
7718	TBC1D3B	AGCTGCCTCCTCTGACTGCG
7719	TBC1D3B	TTCGCCTCCCGCGCAGTCAG
7720	TBC1D3B	TCATTTGACAGCAAATTCGG
7721	TBC1D3B	TATCCACCCACTTGCTCTTT
7722	TBC1D5	ACTAGCATATTACCAACCCG
7723	TBC1D5	GATCAATAATCCTCTTTCAC
7724	TBC1D5	TTGATCGAAGTTCTTTATCT
7725	TBC1D5	TTCCTCAAGACAAAAGTCAA
7726	TBC1D5	GAATTGAAGAATTAAGAGCA
7727	TBC1D5	TATTGATCATCAAATCTTGT
7728	TBX10	CTGAGGGTGCAAGTATGCCG
7729	TBX10	ACACAATCTGGCGCATCCAC
7730	TBX10	GGACAGTGAGCGCTATGCCC
7731	TBX10	TAGCGCTCACTGTCCTTGCG
7732	TBX10	ACCTGTATCTCTTGTCGTCC
7733	TBX10	GCACCCTTGGCTGGCGAGTC
7734	TBX15	TTGCGAATCACATGAACTCG
7735	TBX15	GACTGTGTTCACCACAGTTA
7736	TBX15	TCAGCAGTACTACATAGCAA
7737	TBX15	GCTATGTAGTACTGCTGATG
7738	TBX15	AGCTGCAATGTGCTGACCTC
7739	TBX15	TCATTTCAGTTCCAATATCA

7740	TBX19	CAACACGCACTATGTGAACC
7741	TBX19	AAATCACCTAAGAGACGTAC
7742	TBX19	AGCAGTTTGTTACCATTCGA
7743	TBX19	ACCCAACTCACAGTGAGAAT
7744	TBX19	CCTTTCCTTGGCATCCAAGA
7745	TBX19	CGTGACTGCCTATCAGAATG
7746	TCAIM	AACGTACCGGAAGTACTAAA
7747	TCAIM	ACAGACCAGAGTTCCTCCGA
7748	TCAIM	TTTGCCACCTGAGACCTATG
7749	TCAIM	TTTCCCATTGTTATCTAACC
7750	TCAIM	AATTGCAACTCTCAGATATC
7751	TCAIM	CTTACCTCCTCATAGGTCTC
7752	TCN2	TCGGAGACAACGGATCACCA
7753	TCN2	GCGTAGGCCCACATAGATGC
7754	TCN2	GTGATCCGTTGTCTCCGACC
7755	TCN2	GTCACCGTCATCCTCGCTGA
7756	TCN2	AATACCCTAGGAGGCACTGC
7757	TCN2	CTCATGACTTCCCCCATGCG
7758	TDRD7	TGACCATGTCCACCAACAAC
7759	TDRD7	TTTCAACGGTGGTGTTTGTT
7760	TDRD7	TGAAGCATCACCATCTGTAT
7761	TDRD7	ACCACTCATTTCCTTAGTAG
7762	TDRD7	CCAGCACCGTTAAGAGACAA
7763	TDRD7	ATATCTTAGGTTTAGCCCAA
7764	TEX101	ATAGCATCGAGTTGTACCAT
7765	TEX101	GCAGCCCTTCGTGGCCAAAA
7766	TEX101	ACGAAGGGCTGCATCCCGGA
7767	TEX101	CCTCCATTGTCCAACCTGTG
7768	TEX101	CCTAGAGCTGTATTGTCAAA
7769	TEX101	ATCCAGCCAATATGTTTAAC
7770	TEX9	ATCGGCAAGAAGTACGATCT
7771	TEX9	TGACGTGGTTCAACAAGCTA
7772	TEX9	GCGTTTAAATGCAGAATTGC
7773	TEX9	AGATGAACTATCCCTTCAGA
7774	TEX9	AGAGGTCTGTTACCATCTGA
7775	TEX9	ACGAAGATGATTACAGTTTA
7776	TFAP2C	ATGCTTAAATGCCTCGTTAC
7777	TFAP2C	CCGGTCCTTGCGGGAGAAGT
7778	TFAP2C	CGAATTTCCTAGTAAACCAG
7779	TFAP2C	AAATTCGGCTTCACAGACAT
7780	TFAP2C	TGTATTTAGACGTAGAGCTG
7781	TFAP2C	ACCCCACTGACCTTCTACTA
7782	TFAP2D	TGAGCGCCCGCGCATTGTGC
7783	TFAP2D	TCCTTCCAGATACGTCACGA
7784	TFAP2D	CTTGGCTTAAACTTACCAGC
7785	TFAP2D	GCGCCTCTTTACCTCAGCAA
7786	TFAP2D	CAGTGTGGGCTTGTTCTCAA
7787	TFAP2D	AAAAGTCATACCTTCAACCA
7788	TFE3	GTCATACATACCTTGAGCGA

7789	TFE3	TGCTGCAGGTGGTAGCGCGT
7790	TFE3	AACCCTTACCCCTTCGCTCA
7791	TFE3	TTCACAGGTGCAGACCCATC
7792	TFE3	GAGCATTTCATCATTGTAAC
7793	TFE3	CATTGATGAGATCATCAGCC
7794	TFPT	GCGCTGCCGGGAGATCGAGC
7795	TFPT	GGCCCGGTAGTCATCCCCGT
7796	TFPT	TTCTGCGATTTAATTCCCGC
7797	TFPT	ACTCCTACGGGGATGACTAC
7798	TFPT	GAGTTATCCTCTGCACCTGA
7799	TFPT	CCACCCCATAGGTGAACGAG
7800	TGFA	CGTACGTACCCAGAGCGAAC
7801	TGFA	CGCCCGTAAAATGGTCCCCT
7802	TGFA	GTGCACCAACGTACCCAGAA
7803	TGFA	AACAGCACGTCCCCGCTGAG
7804	TGFA	CTTGGTGGTGGTCTCCATCG
7805	TGFA	GCTGCACTCACCACTCAGCG
7806	TGFB1	CGGGTTCAGGTACCGCTTCT
7807	TGFB1	ATACCTCAGCAACCGGCTGC
7808	TGFB1	TTGATGTCACCGGAGTTGTG
7809	TGFB1	GGCTCACCTGAAGCAATAGT
7810	TGFB1	ACCAAAGCAGGGTTCACTAC
7811	TGFB1	CACTCCTACAGGGGAAATTG
7812	TGFB2	CTTTGCTGTCGATGTAGCGC
7813	TGFB2	TCGATTTGACGTCTCAGCAA
7814	TGFB2	TATAGCTCAATCCGTTGTTC
7815	TGFB2	CCAGTTGTTACAACACCCTC
7816	TGFB2	AACAGCATCAGTTACATCGA
7817	TGFB2	AGATGGTACAAAAGTGCAGC
7818	TGFB3	GCCACCGATATAGCGCTGTT
7819	TGFB3	CCTCTACATTGACTTCCGAC
7820	TGFB3	ATTGCCAAACAGCGCTATAT
7821	TGFB3	ACCTATTCCGAGCAGAATTC
7822	TGFB3	GAAGTGGGTCCATGAACCTA
7823	TGFB3	GCTGATTTCTAGACCTAAGT
7824	TGM5	TACCGACCCACGGCCGCCGT
7825	TGM5	CCACGAAGATGATGTTGTCC
7826	TGM5	CCTGCTTGTTCGCCGGGGCC
7827	TGM5	CCCCGTCTACGTCAGCAGAG
7828	TGM5	CACCACTCTGCTGACGTAGA
7829	TGM5	CCTAGGACCGCTGCCAGACC
7830	THAP9	TTCTCTGCACCTATCGTCAA
7831	THAP9	GATTTCGCCTATGATCTGAC
7832	THAP9	TTATCTCCGTAAAGAACTAC
7833	THAP9	ATTCACTTACGTTCTGAGGA
7834	THAP9	CCAGTGCTACTAACTCCACG
7835	THAP9	CACAGTATAGCACCTGGTCC
7836	THEG	CCTCCAGTTGTCAAAGCCGA
7837	THEG	GTTCGGGGTCTGTGACCCGC

7838	THEG	TCGGGGGAAGCTCCTCTTC
7839	THEG	CGGTTATGGTCTGACACATG
7840	THEG	CGTGTCCTCACCTGTCTTTC
7841	THEG	TGGCAGAGCCCAAGATAAAC
7842	THEGL	TACCTGACCGTGATGCCCAT
7843	THEGL	CGATGGCTTACAGGACGGAT
7844	THEGL	AGACCCTCTAACTTGATCCT
7845	THEGL	ACTTCGCTAGAGCTATTGTT
7846	THEGL	CCATTTCCTTACAAAGTCAC
7847	THEGL	CTGTTTCCTGTGACTTTGTA
7848	THEM6	GGACGACCGCGCGTTCTACC
7849	THEM6	CTGCACGACGCGCTCGGGTG
7850	THEM6	CAGCAGCCAGCTGCTCCGCA
7851	THEM6	TGCCCGCTGATCTGCAGCAC
7852	THEM6	CGCACACGAAGCCGTCCCGC
7853	THEM6	GCGCCTCCAGGTAGAACGCG
7854	THNSL1	CTGGGGCCGACTACTTCCGC
7855	THNSL1	TCATATCAGCTGCACAGACC
7856	THNSL1	AAACTACCTGCGGAAGTAGT
7857	THNSL1	GTTTGCACCTGCTATCATGC
7858	THNSL1	GCTGTAGATGAGATAATCAC
7859	THNSL1	TCAAGTGTAAATGTCGTTCT
7860	TIMP1	TCATCTTGATCTCATAACGC
7861	TIMP1	GTAGACGAACCGGATGTCAG
7862	TIMP1	AGTCATCAGGGCCAAGTTCG
7863	TIMP1	CAGGTCCCACAACCGCAGCG
7864	TIMP1	TTCACCAAGACCTACACTGT
7865	TIMP1	GCCTGAGCTTAGCTCAGCGC
7866	TIMP4	GTTAGGGGCCGAGATGGTAC
7867	TIMP4	GCTCGATGTAGTTGCACAGA
7868	TIMP4	CTGTTGGAACGAAAGCTCTA
7869	TIMP4	TGTGCAACTACATCGAGCCC
7870	TIMP4	CAGAAGCAGTATCTCTTGAC
7871	TIMP4	CAGTCAAGAGATACTGCTTC
7872	TIRAP	CGATTCATGTACTACGTCGA
7873	TIRAP	CATCGACGTAGTACATGAAT
7874	TIRAP	TCTCGGCCTAAGAAGCCTCT
7875	TIRAP	TCACCAGCCATCTTGCCTAG
7876	TIRAP	TCGGACACTATAGCGCCGCC
7877	TIRAP	AGGCTGTGAAGCATCGCTGG
7878	TK1	TGCCTATGACAGCCACGCCC
7879	TK1	CGCCTGCCTGCTCCGAGACG
7880	TK1	GGCACTTGTACTGAGCAATC
7881	TK1	GCTGCTGTAGCGAGTGTCTT
7882	TK1	CAAGACCCGGGGGCAGATCC
7883	TK1	AACCTGCCCACTGTGCTGCC
7884	TK2	TGTACCACGATGCCTCTCGC
7885	TK2	AACTACAGTACCTTCTATAC
7886	TK2	ACGAGTATGCCTGTCCAGCA

7887	TK2	TCCTTTAGGTGTCATCTGTA
7888	TK2	TAGCGTAAGACCCCAGCGAG
7889	TK2	TTGTAGAAAACCTGTATAGA
7890	TLR2	GTTAACGTTTCCACTTTACC
7891	TLR2	TTCCCGCTGAGCCTCGTCCA
7892	TLR2	TATCTAATTTATCGTCTTCC
7893	TLR2	GGTTCAGGATGTCCGCCTCT
7894	TLR2	TGTGACATTCCGACACCGAG
7895	TLR2	GGAACTGCGAGATACTGATT
7896	TMBIM1	AACCTGTCAGCGCCTTTGTG
7897	TMBIM1	GTGGACTTCACCTCGTGCAC
7898	TMBIM1	TAAAAGTGTGTCGCACTTTC
7899	TMBIM1	TAGTGACAATCCCAGTCACC
7900	TMBIM1	AGCCACATTTCTCCTCACAA
7901	TMBIM1	TCTCCTCCCCATCATAGCCA
7902	TMC2	GCAGGAGAAAGCGCGACGAG
7903	TMC2	ACAGCACGAGGCGGAGTGAA
7904	TMC2	TCTCCCCGGAGGAAGCAAAC
7905	TMC2	AAGAGCGGCTCTCCACACAC
7906	TMC2	GCTGCTGACCATGAGCCACC
7907	TMC2	TGGACTCACCTTCCTCTTTC
7908	TMED2	CGGCTTCCTGGACATCGACG
7909	TMED2	AACGAAATAGCCCGAGACCG
7910	TMED2	GACAGCTGTAAAGCACGAAC
7911	TMED2	AATGATCAATGAGCTAGCAG
7912	TMED2	GGCTCCAAAAGGACAAGATA
7913	TMED2	CGACAACACAAACAGCAGAG
7914	TMED5	TAAAGGTGAAGTCGCTATCG
7915	TMED5	TTGGTACTCGATCTCCAGCG
7916	TMED5	CCTGTTCTCCCATATTATCC
7917	TMED5	TACAGTGTAGAGACTGAAGT
7918	TMED5	GTGCAAATAAGACTGCATCT
7919	TMED5	TTCCATCTTGCCTCTCCAGA
7920	TMED6	TAATCTGGCCCCGAACACCC
7921	TMED6	CAAACTATAACTACGTGAAC
7922	TMED6	AGTCATATCGATCAGCTCCA
7923	TMED6	ACTACAACTTTGCCCGGATG
7924	TMED6	TGAATCTAGTGACGTCTGCC
7925	TMED6	TTGGCAGGTTCAGCGGACAG
7926	TMEM127	CGGAGGCACCTGTTCGCGCC
7927	TMEM127	GTGCACATAGCCCAACACGT
7928	TMEM127	CGACGAGTGATCTTCAGAGC
7929	TMEM127	CTGCAGTGTGCCACCGTCAT
7930	TMEM127	CGGCGAAGGTGACATAGACC
7931	TMEM127	TGCCTTCGCCCATATCCTAA
7932	TMEM159	CTTCGTATCACTCGCCATGT
7933	TMEM159	CTTGGACAGCCATCCGTTTC
7934	TMEM159	GAGTATCTCAAGGGACTTGC
7935	TMEM159	CTGTCCAAGTACTGACCCAC

7936	TMEM159	TGCTATCATCATCCCCGACA
7937	TMEM159	CATGAAGTCTGCAGAATTCG
7938	TMEM165	GGACCCCTATGGTGTAGCCG
7939	TMEM165	TTACTGCCAATCCCGTGCAC
7940	TMEM165	GTTATAGCGCATTGCCATGA
7941	TMEM165	CTATAACCGCCTGACCGTGC
7942	TMEM165	GTACCCGTTTCAACATCTCC
7943	TMEM165	AGTTCCTCTTGACCCTCATC
7944	TMEM170A	TTGTGCCGCGGGTGGGCAAC
7945	TMEM170A	TGGCGACGGGATGGAGCGCG
7946	TMEM170A	AGTAGCTAAAATCCGTAAAA
7947	TMEM170A	ACCATTTGAAGCCCTCACAC
7948	TMEM170A	ATCGTGGGACCAATTACTGC
7949	TMEM170A	AAGAGGGCCAGTAATCCAGC
7950	TMEM175	CGCGTGGAAGCCTTCAGCGA
7951	TMEM175	GGCGTAGACTCCGTCGCTGA
7952	TMEM175	GTTCTGGGGAGATCTCCGTG
7953	TMEM175	AGTTCGACAGAAGTGTACAG
7954	TMEM175	GACCTTTCTCATCGTGACAG
7955	TMEM175	AAATACTCACCAGGTTGAGC
7956	TMEM232	TCAAGCACGTTATTCAACTG
7957	TMEM232	GATATTCTACCGTATAAATA
7958	TMEM232	TTTCAGGTTGGATTCAGTAC
7959	TMEM232	GGAGGCTGCCAAATTAAACA
7960	TMEM232	AACTTTCTAGATGACCATGC
7961	TMEM232	ATGAAATTAACCACCTGCTC
7962	TMEM25	GGTGGTGGCCACCAATGACG
7963	TMEM25	TGGATGCGCAGAACTACCCC
7964	TMEM25	AACGCCACGCCTTCACCTGC
7965	TMEM25	TCCCACGCTCAGCAGTCTTG
7966	TMEM25	GGCACCCATCTCTGATATCA
7967	TMEM25	AGTTACCTTGATATCAGAGA
7968	TMEM38A	GGTGCGAGTCCGCAAGATCG
7969	TMEM38A	TGGTTCGTCATGATTGCAAC
7970	TMEM38A	CGCACAGCCAGGACGCGATG
7971	TMEM38A	TTGCTGAAGTAATCGATCAG
7972	TMEM38A	AGCTGAGCGCCAGTTCGCCC
7973	TMEM38A	GCTGCTCAAAGTTGGACATG
7974	TMEM40	GTTGGCATCTACTTCGGACT
7975	TMEM40	GTGTGTTATCACTATTACGC
7976	TMEM40	TAGTCCGAAGTAGATGCCAA
7977	TMEM40	AGCTCATCCTTCAAAACGTC
7978	TMEM40	ATAGTGATAACACACCAGCA
7979	TMEM40	AAGGATGAGCTTCAACTCTA
7980	TMEM44	CCCGACGGTGTCACACAGAC
7981	TMEM44	ACGCAGCACACAGTGCCGAC
7982	TMEM44	CAGATCGGGAAGCCCGAGAG
7983	TMEM44	TCACTAAGTCAATAGCTGCT
7984	TMEM44	TCTGTGGGTTGCTGTCCCGA

7985	TMEM44	ACCTGAATTAGACTTGAATT
7986	TMEM86B	CCATATCCGGCTCGAGGTGC
7987	TMEM86B	GAGGCAGAAGTACACGCAGC
7988	TMEM86B	CAAAGCGCCCAGATGTCTGC
7989	TMEM86B	CCGCCCACCTCCTCTACGTC
7990	TMEM86B	GGGCTGAGCAGTGAGTCTTC
7991	TMEM86B	GAAGACTCACTGCTCAGCCC
7992	TMPRSS6	CTCGGGGTCCACTTCGTACT
7993	TMPRSS6	CTTCGGCGGGGGTGCTACTC
7994	TMPRSS6	TCCAAATCCCCGAGCACCGC
7995	TMPRSS6	CAGGCCTTGAACATCCCCTC
7996	TMPRSS6	GCTCATCACCAGCACCCGCC
7997	TMPRSS6	TCCAGCTCCGTCTATTCCTT
7998	TMTC2	ACAACAATGTTCTCCGTAGA
7999	TMTC2	TAGCACCCACTGTAATCAGT
8000	TMTC2	TTTGTACTACCGCAGCAACA
8001	TMTC2	TTGGCAGCATGGGGTAACCT
8002	TMTC2	CATTGGGAGCAGGCCTACCC
8003	TMTC2	TATTACTTACAAGCCAGGGT
8004	TNNI3	CGTGGCATAAGCGCGGTAGT
8005	TNNI3	CAACTACCGCGCTTATGCCA
8006	TNNI3	TGAGTCTCAGCATGGCGGAT
8007	TNNI3	TCCTTCCAGGAGCAGCGATG
8008	TNNI3	CCCCGTCCCACCTTGGCGTG
8009	TNNI3	GAGCGGCGTCTGATTGGGGC
8010	TNP1	CAAGAGCCGATCTCCTCACA
8011	TNP1	AGCCGCAAATTAAAGAGTCA
8012	TNP1	TGGGGGCTCACAAGTGGGAG
8013	TNP1	CAGCAAAAGAAAATACCGTA
8014	TNP1	TGCCATGACTCTTTAATTTG
8015	TNP1	GCAACCTGAAAAGTAGGAAA
8016	TNP2	GGTACCTGAGCTCCGCGTCT
8017	TNP2	GTACACCTGCTGGATCCTCT
8018	TNP2	CTTTGGTGGTGGACTAGTGT
8019	TNP2	AGGTGTACAAAACCAAGACG
8020	TNP2	AGTTGCAGACAGAGCCATCG
8021	TNP2	TCCAGTTGGGTTGCGGTGGC
8022	TNS1	GCATGGAGCTTCGTGATGTC
8023	TNS1	AGAACCGCTTCATTGCAAAC
8024	TNS1	TGTAGAACAACGACATTGTG
8025	TNS1	TGCAATGAAGCGGTTCTATG
8026	TNS1	CATCACGAAGCTCCATGCCA
8027	TNS1	TGTCGTTGTTCTACACAACA
8028	TOLLIP	CGTGGGCCGACTGAACATCA
8029	TOLLIP	ATGGCGCCAAGAATCCCCGC
8030	TOLLIP	TCTCGTACACCGCGTAGCCC
8031	TOLLIP	TCACCGCGTAGGACATGACG
8032	TOLLIP	GTACATCGGTGAGCTCCCGC
8033	TOLLIP	AATGCGGTCGTCCATGGAGA

8034	TOP1	GAAGCGGATTTCCGATTGAA
8035	TOP1	CCCTTACCTGTTGCTATGCT
8036	TOP1	AAAAGTCCAAGCATAGCAAC
8037	TOP1	TGAGCTTCCATCTTTGTGTT
8038	TOP1	TGAAAAGAAACACAAAGAGA
8039	TOP1	AACACAAAGATCGAGAACAC
8040	TOX2	GGACGTCCGCCTGTACCCCT
8041	TOX2	GGGGTACAGGCGGACGTCCA
8042	TOX2	AGTCTTCGTTGTTCTCGCTC
8043	TOX2	GCATTGCCGGGCGACCCAGC
8044	TOX2	GTCCCAGCTCATCTCGCAGA
8045	TOX2	AGCTACCACTCGCTGTGCCA
8046	TOX3	GGACGATGGACCGCATGATC
8047	TOX3	TGCGTTCTTCGCTGCCAGTG
8048	TOX3	ATGACCGATGCTGCGCGTTC
8049	TOX3	AAGTCTCTCCTCAATGCGCC
8050	TOX3	CTTTCAGATAGGTAGTTATC
8051	TOX3	CAAAACCCCAATGCAACCTT
8052	TP53	CCGGTTCATGCCGCCCATGC
8053	TP53	CGCTATCTGAGCAGCGCTCA
8054	TP53	CCCCGGACGATATTGAACAA
8055	TP53	GAGCGCTGCTCAGATAGCGA
8056	TP53	CCCCTTGCCGTCCCAAGCAA
8057	TP53	ACTTCCTGAAAACAACGTTC
8058	TP63	GCCCTGACCCTTACATCCAG
8059	TP63	CAAACTCACCGCTGGATGTA
8060	TP63	GCCCATTGACTTGAACTTTG
8061	TP63	TTTGTCGCACCATCTTCTGA
8062	TP63	CCAGAAAATCCCAGATATGC
8063	TP63	AAACCCCAGCTCATTTCTCT
8064	TPI1	GAGGGCTTACCGGTGTCGGC
8065	TPI1	GAACGGGCGGAAGCAGAGTC
8066	TPI1	TGTCTTTGGGGAGTCAGATG
8067	TPI1	ACAGCTGATTGGGCAGAAAG
8068	TPI1	GCCTTCTCTCTGAGTGCCCC
8069	TPI1	ATCTAGCTTCTCCCCAATGC
8070	TPMT	ACTTACCATTTGCGATCACC
8071	TPMT	TTTCATTACAGAGTTCTTCG
8072	TPMT	TCAGTCCACTCTTGCCTTTA
8073	TPMT	GCATTAGTTGCCATCAATCC
8074	TPMT	CATTTAGATACTTTCCTTAA
8075	TPMT	CACCAACTACACTGTGTCCC
8076	TPSD1	GCCGCCGCCATACCCGCTGA
8077	TPSD1	CAACAATGCCCGTTTGCTGC
8078	TPSD1	CAGCAGGGCGATGTCCGCCC
8079	TPSD1	TGTGATATTCCGCGTTGCAA
8080	TPSD1	CAGTTCTACATCATCCAGAC
8081	TPSD1	CTGCAGCAAACGGGCATTGT
8082	TPTE	CATTATTGTGATTCTTCTGC

8083	TPTE	CAGCAGAAGAATCACAATAA
8084	TPTE	ATTGTATCATCCTTTGCATT
8085	TPTE	TCTCACATACCCAAATGCAA
8086	TPTE	AACATCCATGAGAAAAAATA
8087	TPTE	CAGCAAACTTTATATTCCTT
8088	TRABD2A	TAATGCCACGCTACCACCTC
8089	TRABD2A	ATGTCCCGTACACCCGAGTT
8090	TRABD2A	TTGTGGATGGGTCGTCCAGC
8091	TRABD2A	CAGCGATCTGTGGGTCCGCC
8092	TRABD2A	CTTCTGGTGCCGGTACTTCC
8093	TRABD2A	TCCGCTTGTAGATCAGCTCC
8094	TRAF5	GTACCGGCCCAGAATAACCT
8095	TRAF5	GGTTTCATCCGCCAGAATTC
8096	TRAF5	CAGTATAGAGTACCAGTTTG
8097	TRAF5	GTATATTGCAGCAATGCTCC
8098	TRAF5	AATCTGCCCTGTAGATAAAG
8099	TRAF5	TTGATGACCTCTTTATCTAC
8100	TRAK2	GAATATGGCTCTTCGATCCA
8101	TRAK2	GAGTCCTTTAGCTTATCTCA
8102	TRAK2	CATCAGATGCATGCTGCCGC
8103	TRAK2	TATTAGTTCTAGGCACAGAC
8104	TRAK2	GTCGATGTCATTGTAAGTTT
8105	TRAK2	AAGAACAACTACCACAGTAT
8106	TREH	ACTTCCTGGGCCCGACGTAA
8107	TREH	GCTCCTAAACCAAGTTCAAA
8108	TREH	TTTACGTCGGGCCCAGGAAG
8109	TREH	CCACAAACTGCTTGTCATCC
8110	TREH	CCCCAGTATGACGTCAGCAA
8111	TREH	AGACCTTCACTGAGCTGTCC
8112	TRIAP1	ACGACCAGTGCTTCAATCGC
8113	TRIAP1	TTCGCCGAGAAATTTCTCAA
8114	TRIAP1	AGAGATTCCTATTGAAGGAC
8115	TRIAP1	ATGAACTCCAGTCCTTCAAT
8116	TRIAP1	CGAACCAGCGATTGAAGCAC
8117	TRIAP1	CGCTTGAAGAGGTCGGTGCA
8118	TRIB3	ACTTCGAGCTCGTTTCTGGA
8119	TRIB3	GTCCAGAAACGAGCTCGAAG
8120	TRIB3	GGCGGATCTTGCCGAAGAGC
8121	TRIB3	TCCGAGTGAAAAAGGCGTAG
8122	TRIB3	TTGTCTTCGCTGACCGTGAG
8123	TRIB3	ATCTGGCCCAGTCAGCACGC
8124	TRIM73	GCCGGTCCTCCAGCTCCAGC
8125	TRIM73	CCCTGGGGACCCAGAGCCCA
8126	TRIM73	CACTCACGACGATTCGGGTC
8127	TRIM73	CCTCTTCTCTGAGCTGAAGC
8128	TRIM73	CACTCACCCAGATGAACTCA
8129	TRIM73	ATGAGTCGGATGTCTTCAGC
8130	TRPM3	AGATTACCTATGGGGGTCTT
8131	TRPM3	AGCGTGGATATTCACTGGAG

8132	TRPM3	AAGCTCCAACTGCCATTCCT
8133	TRPM3	CCAGCACCAAAGACCCCCAT
8134	TRPM3	CCTCTTACACCTGATGACCA
8135	TRPM3	AAGCTTCTCATCTCTGTCCA
8136	TRPM8	GTCTGTGCTCCGAGACGCGC
8137	TRPM8	GAATTTCCTACCGACGCCTT
8138	TRPM8	CCCTGTACTCCAGCGCGTCT
8139	TRPM8	TTACGTGGCCTTGGAATCTT
8140	TRPM8	GGATATTCAGTTTGAGACAC
8141	TRPM8	CTTCTTTACCAAAGATTCCA
8142	TSG101	TATTTGGTGGCCCCGTTGCC
8143	TSG101	GGCGGATAGGATGCCGAAAT
8144	TSG101	TCCAGTAGCCATAGGCATAT
8145	TSG101	TGACAGTTTCACGTACAGTT
8146	TSG101	ACAATCCCTGTGCCTTATAG
8147	TSG101	TCACCATATGAATCCAAAAC
8148	TSPY1	CTTCATCTTCGTCAGTGATC
8149	TSPY1	TGAAGACATGCTGAGCTACA
8150	TSPY1	GATGTTCACCAGATATTCCT
8151	TSPY1	GCAAGATCATGTTGTTCTTT
8152	TSPY1	TGTTAGATCCTGCGAAGTTG
8153	TSPY1	CTCATTCCACTCCAATTGAG
8154	TSPY2	ACAACATTGGCCCAGAAGCC
8155	TSPY2	AGCCCGGCGCATGCGCCCTG
8156	TSPY2	CTTCATCTTCGTCAGTGATC
8157	TSPY2	TGAAGACATGCTGAGCTACA
8158	TSPY2	GATGTTCACCAGATATTCCT
8159	TSPY2	GCAAGATCATGTTGTTCTTT
8160	TSPY3	TACGCCTCCACTTCATAATC
8161	TSPY3	CTTCATCTTCGTCAGTGATC
8162	TSPY3	TGAAGACATGCTGAGCTACA
8163	TSPY3	GATGTTCACCAGATATTCCT
8164	TSPY3	GCAAGATCATGTTGTTCTTT
8165	TSPY3	TGTTAGATCCTGCGAAGTTG
8166	TSPY4	CTTCATCTTCGTCAGTGATC
8167	TSPY4	TGAAGACATGCTGAGCTACA
8168	TSPY4	GATGTTCACCAGATATTCCT
8169	TSPY4	GCAAGATCATGTTGTTCTTT
8170	TSPY4	TGTTAGATCCTGCGAAGTTG
8171	TSPY4	CTCATTCCACTCCAATTGAG
8172	TSPY8	TAATCCAGATACCACTCAAT
8173	TSPY8	CTTCATCTTCGTCAGTGATC
8174	TSPY8	TGAAGACATGCTGAGCTACA
8175	TSPY8	GATGTTCACCAGATATTCCT
8176	TSPY8	GCAAGATCATGTTGTTCTTT
8177	TSPY8	TGTTAGATCCTGCGAAGTTG
8178	TSPYL1	GCGGATGCGTCGACACTACC
8179	TSPYL1	GAGCCCGACGTCGCCCGCTA
8180	TSPYL1	CTCCCTTAGCGGGCGACGTC

8181	TSPYL1	GGTAAGATCCTCCGGCCGAG
8182	TSPYL1	CCCTAATCATGGCGGACAAC
8183	TSPYL1	ACTCCCCAGATCCGAGTTGT
8184	TSPYL2	GAGTTCCAGCGCAACCGCTC
8185	TSPYL2	ACTTACCTGAGCGGTTGCGC
8186	TSPYL2	CATCCTGGTTCCCGTGACGA
8187	TSPYL2	TCTCTCAGGTAGTAGCGTAG
8188	TSPYL2	GAACCCCAGGCCCGTCGTCA
8189	TSPYL2	CCGCTACTTGACCAATCTGC
8190	TSPYL4	GCTTCCAGACCTTTCGTAGA
8191	TSPYL4	GACTAAAAAACGCGCCATCT
8192	TSPYL4	TGAAACTTCTGCGCTGCATG
8193	TSPYL4	CTTCAGCTTGAGCGCAAGTT
8194	TSPYL4	CAACCCCTACTTCCGAAATG
8195	TSPYL4	AAGGATCTGGCGCTCTCCAC
8196	TSPYL5	CGCATCTCTCTCGGAGCGCC
8197	TSPYL5	AGTCCTCCCGCGCCAAAAAC
8198	TSPYL5	CCGAAAAATGCCCCCCGCGT
8199	TSPYL5	AGGACTTTCGACCCCGACTT
8200	TSPYL5	ACATGAACGCCCAGGCGGAC
8201	TSPYL5	TCCATGCTGCCTTCCGTCGC
8202	TSPYL6	GACCCGCATCGACCAAGATG
8203	TSPYL6	CCATAGAGCTAGACGTCGCC
8204	TSPYL6	CCAGGCGACGTCTAGCTCTA
8205	TSPYL6	CAATCGTGTTCCCACCGCCC
8206	TSPYL6	GGTCAGATCCTTCGGCCAAG
8207	TSPYL6	CGCTACTCTCGACTATGCTC
8208	TSTA3	GAACGACAACGTCCTGCACT
8209	TSTA3	GACCCGCGCCCTGTTTGAGA
8210	TSTA3	GAGGCAGTTCATATACTCGC
8211	TSTA3	GAGTATATGAACTGCCTCCG
8212	TSTA3	CCTGCGGGAGTACAATGAAG
8213	TSTA3	CTACCCGATAGATGAGACCA
8214	TTC19	GTATCTATGCTGCGCAGAAC
8215	TTC19	GTTCTGCGCAGCATAGATAC
8216	TTC19	AGCCGACGGGGCCGCTGCCG
8217	TTC19	CAGCGCTCGCCTGGTTCTCG
8218	TTC19	GCAACAATGAGTTACCTCCT
8219	TTC19	TTAGCAGAAGACATTATGTC
8220	TTC33	CTCTTGGGCAAGAACGCTCC
8221	TTC33	GGCTTCATGCCATTAAACGT
8222	TTC33	GATGGTGCTACACCACCAGA
8223	TTC33	CCTATACGAGATGAAATCAC
8224	TTC33	ATGTAGTTGACAACGATGAA
8225	TTC33	CCTGTGATTTCATCTCGTAT
8226	TTC39B	TGTCCTGACCTTCGAGCAAC
8227	TTC39B	CCAGGGGCGAAGCAATTCTA
8228	TTC39B	CTGGATGTCCTGTTGCTCGA
8229	TTC39B	CCTTAGAATTGCTTCGCCCC

8230	TTC39B	TTACATTGAGATGGTTTCCA
8231	TTC39B	GGTTTGTAAAGCGTCCTTCA
8232	TTC39C	CTGAAGCCGATCAACCATAG
8233	TTC39C	CCGAAAATCCGCCCCCTCTA
8234	TTC39C	GAATGCCATGATGACATTTG
8235	TTC39C	TACCAAAAAACTGACAAAGC
8236	TTC39C	TTCTTCCTCAAATGTCATCA
8237	TTC39C	GATAATCATAGCTGACTGCC
8238	TTI1	GCAGCGTTCCATCACGTCCA
8239	TTI1	ACCAGTTTACTCGCACACGC
8240	TTI1	AAATCTGCAAATCCGCCTGA
8241	TTI1	GTGGTGACTTTCTTCGCAGC
8242	TTI1	ACAGCCCGTGAAATTACAAG
8243	TTI1	TGCCCAAGATTACCTGTGTC
8244	TTLL11	ACAGTTCAACTACCTGCGCC
8245	TTLL11	CAGGCTTTCGTACCTTCATA
8246	TTLL11	CTCATGTTCGATTCTCATAC
8247	TTLL11	TGGAAACTTGTTCACTTGAC
8248	TTLL11	GCTTGCCTTGTGACATCTAC
8249	TTLL11	GCATTTACTTCAAGTAGTAT
8250	TTN	GCAGCCGTTACAAAGCGTTG
8251	TTN	CGGGGATCGTCAGTTTAGCG
8252	TTN	GTAAATGCCACCAATAGCGT
8253	TTN	AGTAGCTCTTCCAACGCTAT
8254	TTN	AGTACCACAACGCTTTGTAA
8255	TTN	GTGCAGATCTCCTTTAGCGA
8256	TTYH1	AGGTTCAGAACATAAGGGTC
8257	TTYH1	GCCACTTGCTCTGCTTCGCC
8258	TTYH1	TTATGTTCTGAACCTGACCC
8259	TTYH1	CCCAGCTCAGGACGAGAACC
8260	TTYH1	GCCTGGCGAAGCAGAGCAAG
8261	TTYH1	CCTGGAGGCAGCCACGGCCG
8262	TUBB3	CGAGATGTACGAAGACGACG
8263	TUBB3	GCCTCACCTTGGCCCCGATC
8264	TUBB3	GTCGCCCACGTAGTTGCCGC
8265	TUBB3	GATGGACAGCGTGGCGTTGT
8266	TUBB3	ACCATGGACAGTGTCCGCTC
8267	TUBB3	ACTCGGACTTGCAGCTGGAG
8268	TYMS	GAAGAATCATCATGTGCGCT
8269	TYMS	TGACAACCAAACGTGTGTTC
8270	TYMS	ACCCTTCCAGAACACACGTT
8271	TYMS	TTCTACAGATTATTCAGGAC
8272	TYMS	AGGTGACTTTATACACACTT
8273	TYMS	CATCGAGCCACTGAAAATTC
8274	UBE2QL1	TGGACGAGAGCCTGTTCGAC
8275	UBE2QL1	GGCTGCGAACTGGCGCATGA
8276	UBE2QL1	GTCACCCCGCCCGTGTCCGA
8277	UBE2QL1	CAGACATCAGCCGTCGGACA
8278	UBE2QL1	TAGCGACCGCTTCATCTCCG

8279	UBE2QL1	GCTTCACGTTCCAGTCGAAC
8280	UBE4B	TCATGTTGTAGATTCGACGG
8281	UBE4B	GGCTTACCTGATGCACCTAT
8282	UBE4B	CACCATCGATATCCATGCTC
8283	UBE4B	GAGACTGAGATTGCGTTTCA
8284	UBE4B	GGAGGAGCTGAGCGCTGATG
8285	UBE4B	TCCTGGGGCTGATGCCGCTA
8286	UBR1	ACGCTGTCCGTTGAATGAAG
8287	UBR1	TATCAGATGCATACTTCTAC
8288	UBR1	TCATAAAAATCATCGTTACA
8289	UBR1	TCCAACATGTGTACTCTGTA
8290	UBR1	ATGTCGTAGAAATGACTATA
8291	UBR1	TTTATAGTACCTGCTCTTCC
8292	UBR7	GCTTATGCTGCACAATTGGC
8293	UBR7	AAAAGAACAACGTTTCATGC
8294	UBR7	CAAGCTAAACAAATTCCTGC
8295	UBR7	GCTTGCAGTTATGAATGTCA
8296	UBR7	AAACAGCAAGTTTAAAAATT
8297	UBR7	GGTGCCATTCCCCCTGAGAG
8298	UCHL5	CTGATAATGTCTCGCCTAAA
8299	UCHL5	AAGTACACAACAGTTTCGCC
8300	UCHL5	GTTACTGAACTGTACCCACC
8301	UCHL5	AACTCCATATTTCTTCTACT
8302	UCHL5	GAGCCCAAGTAGAAGAAATA
8303	UCHL5	GTTCAGTAACACACTCACTA
8304	UCN3	TCCTCCCAATTTGCGCCATC
8305	UCN3	GCCCACCTGATGGCGCAAAT
8306	UCN3	GTATCTGTACCGGGTGCCTC
8307	UCN3	CGTCTCTGCTGCGCAGGTAG
8308	UCN3	GCGCAGCAGAGACGCCTCTT
8309	UCN3	TGATGTTGGTGGGGACGTCG
8310	UEVLD	ATTCCGATCACTCGATTTGC
8311	UEVLD	CACTTACCCTGATACATCAC
8312	UEVLD	ATTCTTACCATAGGTGTCCA
8313	UEVLD	GAAGATTTCAAGGTCCATCG
8314	UEVLD	TTTCCTGCAAATCGAGTGAT
8315	UEVLD	CTGTACAGGGTATTGCAGAC
8316	UMPS	TGGAGCTCATTTCCGCAATA
8317	UMPS	CTGAGAAGCTTCGATGCAAC
8318	UMPS	TTTGTGGCAGCGAATCATAA
8319	UMPS	AGATCTAGTAAATGCTCACG
8320	UMPS	GAACAGATAACTGTAGCCAA
8321	UMPS	GCCTTATACAGCTTTGCCAT
8322	UNC13B	GATCCCGCACGTATTCATTG
8323	UNC13B	TTGAGCAGTTCGTGCTACAA
8324	UNC13B	GGCTCCACAATGAATACGTG
8325	UNC13B	AACCGTCTTCTCACACACAG
8326	UNC13B	CATCCTTCTTATCTCGTTCC
8327	UNC13B	AGCGTGTACTGAAGGAGCTC

8328	UPF3B	ATCATCGATAGTCCCGACTT
8329	UPF3B	TTGTTCAGGGATCGCTTTGA
8330	UPF3B	ATTACCTCCCACTTTGACCA
8331	UPF3B	TACTCCAGAGACACTGCTAG
8332	UPF3B	AGGACTCACCTTTATTGTCA
8333	UPF3B	ATTTCCTCTAGCAGTGTCTC
8334	USH2A	GTACACGCGTCGCCCTCCGT
8335	USH2A	GCGACGCGTGTACAGCGGCT
8336	USH2A	GGCTTGGTCCTAACAACTCC
8337	USH2A	CGTCAGTCGTGCAGATGACC
8338	USH2A	CATAGTGTTAATGGCGATGC
8339	USH2A	ATCCAATATGATACCTCTAC
8340	USP11	CCATTATACCTTGCGTTCAA
8341	USP11	TTCAGCCATACCGATTCTAT
8342	USP11	CTGGGTACACTTCGACCTTC
8343	USP11	CCAGCCACCCATTGAACGCA
8344	USP11	GCAGTGGGAGGCATACGTGC
8345	USP11	GGTTCCTTGTGGAGAAGCAC
8346	USP17L11	GCTGTGTTTGTTCTTCCCTT
8347	USP17L11	GGAGGACGACTCACTCTACT
8348	USP17L11	CACCAAGTGCTCGTCCAACT
8349	USP17L11	CGATGCAAGCTCACATCACA
8350	USP17L11	TGTCTGTGTCTTCTGCGCCA
8351	USP17L11	CCACGTCATCCAGCCCTCAC
8352	USP26	AGCTCACTTAATGACGTATC
8353	USP26	TACGATGATATGCGGGTGTT
8354	USP26	GACAACACTAATGAGCCGGT
8355	USP26	ATGGCTGCCCTATTCCTACG
8356	USP26	TCATGCATCATGAACGCCAC
8357	USP26	CATTCAAGCTATAGCGTTTG
8358	VAMP2	CCAGTATTTGCGCTTGAGCT
8359	VAMP2	CCAAGCTCAAGCGCAAATAC
8360	VAMP2	CAGGTCTGCTACCGCTGCCA
8361	VAMP2	CCCCCAGATGATGATCATCT
8362	VAMP2	CTGCTGCAGTCTCCTGTTAC
8363	VAMP2	ACTCACCTATGATGATGATG
8364	VEGFA	AGCAGCCCCCGCATCGCATC
8365	VEGFA	CTGCCATCCAATCGAGACCC
8366	VEGFA	GGAGGGCAGAATCATCACGA
8367	VEGFA	ATGCGGATCAAACCTCACCA
8368	VEGFA	AGCTCATCTCTCCTATGTGC
8369	VEGFA	GTGGTCCCAGGCTGCACCCA
8370	VEGFB	CCAGTACCTGCATCCGGACT
8371	VEGFB	GCGCTGCACAGTCACGCAGC
8372	VEGFB	GAGCACAGATCCTCATGATC
8373	VEGFB	GCTGGCACCTGCATTCACAC
8374	VEGFB	AAGTATTACCTTTCCTCTGG
8375	VEGFB	GTCTCCCAGCCTGATGCCCC
8376	VEGFC	ACATCTATACACACCTCCCG

8377	VEGFC	CAATACCCACCGTCTTGCTG
8378	VEGFC	CTGCCGATGCATGTCTAAAC
8379	VEGFC	CCTGCCCCACCAATTACATG
8380	VEGFC	AGTCATGAGTTCATCTACAC
8381	VEGFC	CTTACCATCTCCAGCATCCG
8382	VGF	CGCCATACGCGCTCTGGCCC
8383	VGF	CGGCCGGGATAGTGGCGCGA
8384	VGF	CACGCTGACCCGAGTGAATC
8385	VGF	CAGGCGCGTATGCCCGACAG
8386	VGF	CGCTCGGAATTCCGAGCCGC
8387	VGF	ATGCTGACCACGTCGTCCGC
8388	VHL	CGCGCGTCGTGCTGCCCGTA
8389	VHL	GCCGTCGAAGTTGAGCCATA
8390	VHL	CAGGTCGCTCTACGAAGATC
8391	VHL	TGACTAGGCTCCGGACAACC
8392	VHL	TGTCCGTCAACATTGAGAGA
8393	VHL	GTGCCATCTCTCAATGTTGA
8394	VIL1	TTGAGGAAATTGGCCCGCGC
8395	VIL1	CGGTCTCCAAGTTGTTAGTT
8396	VIL1	TGGTGAGGGAAGTCGCCACA
8397	VIL1	CACCTGAGGCTTGACATGCA
8398	VIL1	CACTCTAGCCAAAGTGGAAC
8399	VIL1	CTCTTACCTCGTGACTGAGC
8400	VPS13D	GGCGGTAACTGAATACCAAT
8401	VPS13D	GGCGATAAAAGGGAATCTGA
8402	VPS13D	TTACCGCCTCCGTAGTTACA
8403	VPS13D	AGATCACCCAAGGGTCCACA
8404	VPS13D	CTCTCAGTTGCACTTCTCAA
8405	VPS13D	TGTTGGAAGGCCTTGTAGCC
8406	VPS26A	TTCATACCTCAAGCGGACAT
8407	VPS26A	AACCTAGCCTTTAAGCAACC
8408	VPS26A	ACAATATCGATCTCACAAAT
8409	VPS26A	AAGCCATATGAATCTTACAT
8410	VPS26A	CCCCAATAGGTATCATTTAA
8411	VPS26A	ATCGATATTGTTCTTAATGA
8412	VPS28	TGTCCTCAGGTACGACAACA
8413	VPS28	AGGCACTCACGCGGAACTTG
8414	VPS28	TGAAGTTGTACAAGAACGCC
8415	VPS28	GGTGAAGACAATGCAAGCCC
8416	VPS28	TGCAGCCCCTGGGAACAAGC
8417	VPS28	CCACTCACCTCATACAGCTC
8418	VPS37B	GGGGTGGCTAAGCGTCCCGC
8419	VPS37B	CTTAGCCACCCCGTTTACTG
8420	VPS37B	CCGGTTCGCCGGGCTGTCGC
8421	VPS37B	GTGCTTTCAACGTGTCCAGC
8422	VPS37B	ACGCTTGACCCAGAAATACC
8423	VPS37B	TCTGCACCATCTCCGTCAGC
8424	VPS37D	CCAAGCTGGACCGGATCGTG
8425	VPS37D	CACCGTGGATCCTACCGGTG

8426	VPS37D	CGGGCGGCATGTACCGGGCC
8427	VPS37D	CAAATACCAGGAGCTTCGTG
8428	VPS37D	TCGGGAGCGTTCTGCCCAGC
8429	VPS37D	CAGCTTGTCCGCGCAGTTCT
8430	VPS72	ATACACGACAAACGTTCCTT
8431	VPS72	CGAAAGGTCAACACCCCGGC
8432	VPS72	CATAAGAAGCGGAAGTGCCC
8433	VPS72	CCGTCATCTTGTAGTTCTAA
8434	VPS72	CCTGCCTCTCCTGTACCCGA
8435	VPS72	TCCGGAGATGATGAGTATCA
8436	VRK3	AACCAGGGCATTCTCTATGA
8437	VRK3	TGACATCCAGGGCCGACTGA
8438	VRK3	CTCTCTGACAGCACATGCTT
8439	VRK3	CTTCAAGTGAGGTGGTCACT
8440	VRK3	GCAAAAGTTCTCACTCAAAC
8441	VRK3	TCCACCCTCACCTGTGACTC
8442	VWA1	CCCGGACACGGACTACGACG
8443	VWA1	GGACGCCGGCTTACGGCTCA
8444	VWA1	CAATGAACACGGTGACGCCC
8445	VWA1	CCTCGCGCGCGATGCTCCCC
8446	VWA1	GCCAGGCCGAGCGCCGTCCA
8447	VWA1	ACCGTGTTCATTGTCAGCAC
8448	VWA3A	CTTGGGCAAAATTCGGACAA
8449	VWA3A	TACACATGTTAACCAGACAC
8450	VWA3A	CAAACTAGTCATGTCTTCCA
8451	VWA3A	ATATGTTTCTGGAAAACCAT
8452	VWA3A	TCTATCAAGAGAGAATTCAG
8453	VWA3A	TTACCAGTAAGTCCTGTGTC
8454	VWF	AACTCGCGGCAGGTCATCCA
8455	VWF	AAACGCTCCTTCTCGATTAT
8456	VWF	GTACTACAAGCTGTCCGGTG
8457	VWF	GTTGCCGCTGCCATCGATCC
8458	VWF	AAGCAGCACCCCGGCAAATC
8459	VWF	CCGTGACACAGGGGGACCAA
8460	WDFY3	AGTCCTAAGGCGTTGTCTTG
8461	WDFY3	AGCCCACAAGACAACGCCTT
8462	WDFY3	AACACAAGTCTCACGACTAA
8463	WDFY3	TAGAAGCATCCAGCCTCTAC
8464	WDFY3	ATTAATTTGTTAGCTTCCTC
8465	WDFY3	AGAGCATCAAACAAATCCAC
8466	WDR17	GTTTGCATATTGTGCGACCC
8467	WDR17	TCCCATTCCAAGGCCGTAAC
8468	WDR17	TGTACAATCACTCCACTATC
8469	WDR17	TTTGCATTATGGAATTCGCC
8470	WDR17	GCTAAACTCGACAGTACAAA
8471	WDR17	GAGATGGTCCAAATGAACAG
8472	WDR20	GTTCTTAGCGTGCGTGAGCC
8473	WDR20	TTCCTAGTAGCCCACTCGAG
8474	WDR20	GGAGGCTCTAGTAACTGTCC

8475	WDR20	TGGTTGAAGTCATGACAAGT
8476	WDR20	AGAACTCTTGTCAGCACTTG
8477	WDR20	AGAAAGTGTCTCTCTCCTAG
8478	WDR61	GCTCAGCGTGCCAGCCAAAT
8479	WDR61	TTGATGCTCATATTCGTCTT
8480	WDR61	CCTGTTGAAGGCGTGATGAG
8481	WDR61	GAATGCAACGTTCAGCACCC
8482	WDR61	ACCATTTCCAGACCTTCACC
8483	WDR61	CCCCCAAGCAACTGACCAAA
8484	WDR62	CTAACCTGTGACCCCGGCAC
8485	WDR62	AGCACATCTTTAACACCGCC
8486	WDR62	ATCTGCTTACCCTGCCAGGT
8487	WDR62	GTCACTATGTACTTCCCATC
8488	WDR62	GATGGGAAGTACATAGTGAC
8489	WDR62	GTGCTGCTGCTTGTTCTCCT
8490	WDR78	ATCAACAATACCCGCCAACT
8491	WDR78	AGTCAATCTTTCCCGTTTAT
8492	WDR78	AGACCAAAGTCGTTCCAAGT
8493	WDR78	GAAAGATTGACTAGTTTCAC
8494	WDR78	GCATCATCCTTTCAACATAT
8495	WDR78	TGCCTAATCTGTTTCTACAA
8496	WFDC11	TGGTCAGCCTCATGAAGCTC
8497	WFDC11	CCTATTGTGGAAACATCTGC
8498	WFDC11	CCAGCAGATGTTTCCACAAT
8499	WFDC11	GCTCATGACATTCTTCTGTA
8500	WFDC11	AATTGTTACTTGAAGAATGC
8501	WFDC11	TTGGTACATTCTTTGACATT
8502	WFDC13	AATCTTCCTGCATTGTACAC
8503	WFDC13	AAACATTTCAAAAGCGCAAC
8504	WFDC13	CCTACTCAGAACACGCTGCT
8505	WFDC13	GGAAATATGCCTCAGTTGGC
8506	WFDC13	GTTCTGCCTAGCACTGCAGC
8507	WFDC13	GTCATCATGCCTGCCAACTG
8508	WFDC5	GATTGCCGGGATCCTGCCAG
8509	WFDC5	ACTGGCTGTCTTCCACGCAC
8510	WFDC5	TGATGGGCCCTGCCTCCTAT
8511	WFDC5	AATCCCGCCCGCAGGCGCTG
8512	WFDC5	AGTCAGCTGCCTGCTGTCTT
8513	WFDC5	TTCTTCCTGCCAAAGACAGC
8514	WNT16	TGCTCCGATGATGTCCAGTA
8515	WNT16	GGCGCCCATCGGGGCGGTAG
8516	WNT16	TGGCCCGCTTGTGCGCGCTG
8517	WNT16	CCGAGGTTACAACACCCATG
8518	WNT16	TGCTGAGCCGCCGTTCTGCA
8519	WNT16	TGCTCAGCTCGTAGCCAAAG
8520	WNT3	CGTAGATGCGAATACACTCC
8521	WNT3	GATCGCAGCCATCGATGCCG
8522	WNT3	CTTCGTTCACGCCATCGCCT
8523	WNT3	TCCTCGCTGGCTACCCAATT

8524	WNT3	CGGCTGTGACTCGCATCATA
8525	WNT3	TTTGGGCCCGTCCTCGACAA
8526	WNT5A	TGCGCTCACCGCGTATGTGA
8527	WNT5A	GAGGATGCGAGCACTCTCGT
8528	WNT5A	AGTATCAATTCCGACATCGA
8529	WNT5A	TGCAGTTCCACCTTCGATGT
8530	WNT5A	ATGAACTTACCACCAAGAAT
8531	WNT5A	GTCTTCCAAGTTCTTCCTAG
8532	WSB1	GCACAGTAAAGCTTGTTCCG
8533	WSB1	CAGTCTATAATATGTTCACG
8534	WSB1	TTAGTGAGATTACGTACTAT
8535	WSB1	CAGTATGATCTACCAAGTTA
8536	WSB1	GATCAGCTACTTCTTGCTAC
8537	WSB1	TCTTCTATGCTGTGTTCAGT
8538	WWOX	TGCTGCCGTCGTATCTTTGC
8539	WWOX	TCCACAGTAAACGCCAGTCT
8540	WWOX	GTTTCTTGTTCCCATCCGTA
8541	WWOX	CCAAGATCACATGTGCACCA
8542	WWOX	CACATGTGATCTTGGCCTGC
8543	WWOX	ACACCGAGGAGAAGACTCAG
8544	XDH	TCAAATCGCAGCTGCTTCCG
8545	XDH	CGTGTTCCCCACGACCAGCT
8546	XDH	TCCAGGGGCGTGAACTCCTC
8547	XDH	GCATCACCTACCAGCAACTC
8548	XDH	TTACTGAGTGGTCTTTCTTC
8549	XDH	CTCTAGGATGGTGGATGCTG
8550	XIRP2	GGAGCGGATGGCGAGGTACC
8551	XIRP2	GGAGGCAATTCATAGCAGCC
8552	XIRP2	TGCTTCTTGAAGTGCCAACC
8553	XIRP2	TCAGAAATGTGCGCAGTGCC
8554	XIRP2	ATCTGTTCTGATGTTGATAT
8555	XIRP2	TTACATTAGCAGAGAAGCTG
8556	XPC	TTACTCTGCTCGAGATGATG
8557	XPC	CAAGTCGGGCTTTCCGAGCA
8558	XPC	TGCCCTTACCTTCCCGTCCA
8559	XPC	TCCTTTCCAATGTAGTCTGC
8560	XPC	TCAGATGGTGTGCCTTCTTG
8561	XPC	TGAGGTCATCCCCATCGCTG
8562	YEATS2	ATAGTAGTTTGCTACAATGC
8563	YEATS2	CAGAGACCAAGCCGAAATAC
8564	YEATS2	AGCGACTGATTGAAGCAAGA
8565	YEATS2	TATATACCCTTCCAGTATTT
8566	YEATS2	TGAAATTGAAGTCATTGACC
8567	YEATS2	TGCTCTTGAAATGAAGAATA
8568	YEATS4	GCAACATTACCGTAAACTAT
8569	YEATS4	GAATTTGGGCCTGACTCCGG
8570	YEATS4	ACTGACCTTTACTCTCCCGC
8571	YEATS4	ACATCTCGTCAGCTAACATT
8572	YEATS4	ATCGTTAAACCAATAGTTTA

8573	YEATS4	ACAGCTTTAGCAAATGATAC
8574	YLPM1	TCCCATACCGGATACATTCC
8575	YLPM1	GGCTGACCATCTACCACCTC
8576	YLPM1	GTCTCAAAAGTTGATCTTTC
8577	YLPM1	AAAGTTGAACAGATACCTTA
8578	YLPM1	CAGATTGTTGTTATAATGAG
8579	YLPM1	ACATGTGTCTTTCCACTGCC
8580	YPEL1	CACGTATTTCCACCCGAGCG
8581	YPEL1	GACAGTTCGGCAGATACGCT
8582	YPEL1	CCGCATGCAGCCCGGTGAGA
8583	YPEL1	GTACTCACACGGAATTGAAG
8584	YPEL1	AGTGGATACAGCTGTACGTT
8585	YPEL1	GCGTCCCTGGCTCCCCTGAA
8586	YY1	GATGTAGAGGGTGTCGCCCG
8587	YY1	GTCGGGTCGTCGGTGACCAG
8588	YY1	TCTCTCCAGTATGAACCAGT
8589	YY1	CCTCTCAGATCCCAAACAAC
8590	YY1	AAAACTAAAACGACACCAAC
8591	YY1	TGAGGGCAAGCTATTGTTCT
8592	ZBP1	GCATGGACGATTTACCGCCC
8593	ZBP1	GCGGTAAATCGTCCATGCTT
8594	ZBP1	TTGGGCGGGACTGATCCTGA
8595	ZBP1	ATCAGTCCCGCCCAAGCACC
8596	ZBP1	CTCGAAGCTCACCCCCAAGC
8597	ZBP1	AGTCACTTTCCTCGAGACAT
8598	ZBTB1	CGTCTTGACACACGTTCTTT
8599	ZBTB1	GAACGTGTGTCAAGACGCTT
8600	ZBTB1	CAGATCTCTCACTTCTCCAC
8601	ZBTB1	CTGTGTGGCAGTGGTGAAAT
8602	ZBTB1	CAGGAACATGCTCAACGATG
8603	ZBTB1	CCACAATTAGGACATCGAAA
8604	ZBTB21	ATGTCCGAGGGGTCCGACAG
8605	ZBTB21	CGTTCCGGCACAGCCGCTCG
8606	ZBTB21	TGAAGGTGCGCATGCACTCG
8607	ZBTB21	TCTTGTGACCGACGAAGATG
8608	ZBTB21	CCGGGAAGTATGGCTTACAT
8609	ZBTB21	CCGATGTAAGCCATACTTCC
8610	ZBTB7A	TTGAATGTAGCGGGCGCCGG
8611	ZBTB7A	ATTCAACCGGCCCAAGCCGT
8612	ZBTB7A	GTAGTGGCCGTTCTGCGTGG
8613	ZBTB7A	GATGGGGCCGTCCACGCCGC
8614	ZBTB7A	AACGGCGTCCCCTCGCGCCG
8615	ZBTB7A	CGAGTTCCCCACGCACCGCT
8616	ZC3H13	CTTAGACACAGTGCCGTAAC
8617	ZC3H13	CTGCTATAACCTTTACCACG
8618	ZC3H13	TGAAGCGTCAGAAGATACAG
8619	ZC3H13	TTATGTTCATGAATTGTCAT
8620	ZC3H13	GGAAGACATAAAAATCACTA
8621	ZC3H13	GTGAAGAAGAAAATGTAGAA

8622	ZCCHC12	GGCTCCCCACCGATAGTGAT
8623	ZCCHC12	TACCCTCGACGACTCCGATG
8624	ZCCHC12	GAGAAGTCTCGGTCCTATAA
8625	ZCCHC12	TACTCAGCTCACCGCCTAAA
8626	ZCCHC12	TCAGCACTGCCGATCACTAT
8627	ZCCHC12	GGACCTCCGACTCAGACTTA
8628	ZDHHC19	CGGATACGCGATGGCCTTGT
8629	ZDHHC19	ACGGGGCCTTCCGCCTGCAA
8630	ZDHHC19	CCACACCACGTGCACCGTCA
8631	ZDHHC19	GCAGGTGGGTTGTGCGCACC
8632	ZDHHC19	TCACCTTGATGTAAGATGCC
8633	ZDHHC19	TGGGCCTTTCCTGTTATCAC
8634	ZDHHC4	GTAGAGCACCTCACGTTCTT
8635	ZDHHC4	GACCCATCAGCACCGAAGCC
8636	ZDHHC4	TCGAAAACCCATAGCTTGAA
8637	ZDHHC4	TGGTTTATACTGAGTACACC
8638	ZDHHC4	GTTTACACCTAGCAGCAGAT
8639	ZDHHC4	TGTCTTCAGAGAGCCGTGCA
8640	ZEB1	CAGAGACATGTGACGCAGTC
8641	ZEB1	TGAAGACAAACTGCATATTG
8642	ZEB1	ACAGATGCAGCTGACTGTGA
8643	ZEB1	AAATTCAAATGCACTGAGTG
8644	ZEB1	GCTTCATCTGCCTGAGCTTC
8645	ZEB1	GAAGCTCAGGCAGATGAAGC
8646	ZER1	TGCTGCGGGGGTCCGAAAAG
8647	ZER1	CTCGTGTGGCTCGAAGTTAC
8648	ZER1	CAGGGACCCAATCAATCATG
8649	ZER1	GCTGCGGCCGCTTAACTCCC
8650	ZER1	AGAGCAGCATCATACCTTTC
8651	ZER1	GAGAGTTCTCAAAGAGCCCG
8652	ZFAT	GAGAGGAGTTCCGACTGATT
8653	ZFAT	ACTGCACACGAAGCAATTCC
8654	ZFAT	CACAACCTTACCAGGATGGT
8655	ZFAT	CTTCGCCCCAGAAAGCTGTC
8656	ZFAT	CCATGTGCTTCTCTGAAACG
8657	ZFAT	CATATCAAGACCAACCATCC
8658	ZFHX4	ATCCAACCTTCCGCGGTTTA
8659	ZFHX4	TTCGATGGTTGTGTTAGCGA
8660	ZFHX4	GGTTACTCACCAAGCTCATT
8661	ZFHX4	TCTGGTACAACATGTCCGTT
8662	ZFHX4	AGCTATTAAGCCTACAGCAG
8663	ZFHX4	AGTGAGAGAGACAATAGTGA
8664	ZFP1	AATTGCCTCGCCTGCTCGTC
8665	ZFP1	AGATCGCCTCTTTCCTCAAT
8666	ZFP1	AACAACTGGACCCCTCTCAG
8667	ZFP1	CCAGCATCACATCCATGTAT
8668	ZFP1	GCAGAAAATGAACAAATCCC
8669	ZFP1	GGAGAAACCTTTCGAGTGTC
8670	ZFP37	TTTACTTGGACAACCTTGAC

8671	ZFP37	CTACTGCAACCAAGCCTCAA
8672	ZFP37	GATGGCTGTGTCCGAGCCCG
8673	ZFP37	AAGTGCGGAAACGACCAAAG
8674	ZFP37	TACACTTATCAGATGAGCTA
8675	ZFP37	CATGCTTAGTAGATGAGCTA
8676	ZNF136	TTCATGTGTTCGAAATCTAC
8677	ZNF136	AAAGATCACTACAAACACCG
8678	ZNF136	ATCATGTGTCTTCGCATACT
8679	ZNF136	ATGAGGAATCTGGCCTCTAT
8680	ZNF136	ATTACCTTAGATTTCTCCCT
8681	ZNF136	TATAGAGGCCAGATTCCTCA
8682	ZNF189	AAAGGCCTTTAGCCGGAACT
8683	ZNF189	TGCTTACTTACCTTCGACTC
8684	ZNF189	ACCCATGAATGTGACGCTTG
8685	ZNF189	GGCTTCCCCGAGCCCCCCGC
8686	ZNF189	GATGTCATGATGGAGAATTA
8687	ZNF189	GATTCTTACCCAGTGAGACC
8688	ZNF24	TTTCTCTCCGTCGACGAAAA
8689	ZNF24	GATTAATAAGCCCCGAATTC
8690	ZNF24	ACACCCATATTGAGAGTGCC
8691	ZNF24	CAAACTGCCTGAATCGCTGT
8692	ZNF24	AACCCAAGTCTGTAGCTCTT
8693	ZNF24	CCCAGGATGCCCACTTGAGC
8694	ZNF28	CAGTATGAAGCGCCTTGTGA
8695	ZNF28	CAACCCTTGCACGCCATCGT
8696	ZNF28	CTGTAAAGAGTCCTCTGAGC
8697	ZNF28	TCTTTACAGGGATGTGATGC
8698	ZNF28	GTCAGGGATGGCTCTTCCTC
8699	ZNF28	TTTCAGTCGCAAATCACACA
8700	ZNF350	GTTTGCGTATATGTCGTGTC
8701	ZNF350	TTGCAGTATGAAGTCGTTCA
8702	ZNF350	ATTACAGGAATCCATAACAC
8703	ZNF350	CAACTGTGGACAATTGAAGA
8704	ZNF350	ACTCCTGGGCGCTGCTCAGA
8705	ZNF350	CACCTGAACAGGCTCCACTG
8706	ZNF407	TGAGTAATGGCACGTGGGCC
8707	ZNF407	AAAAGCCGTTTAAATGTACC
8708	ZNF407	GAGTTGGCACCCCAAAAGAA
8709	ZNF407	CACTTAAATACTCATCTACT
8710	ZNF407	CAAGCTGAGCTGTTGCAAAA
8711	ZNF407	ACTTGTATTCCCTCATCCCA
8712	ZNF425	GTTACACTCGGGACACGAAA
8713	ZNF425	GCTCCCCGCGGTGTAGACAC
8714	ZNF425	CCTTCTTACCGAAGCCGGCT
8715	ZNF425	TTACGAGACCCTTGATTCCC
8716	ZNF425	AATGCTATTAATCAGCGAAC
8717	ZNF425	CCAAGCCAGATTTGATCACA
8718	ZNF470	CATCCCTAGCTCATCATCGA
8719	ZNF470	TCCCTAATGTTGAACATTCA

8720	ZNF470	TCAGAGAAGTTTGTACAAGA
8721	ZNF470	CTACAGGAACCTAGTTTCAG
8722	ZNF470	AAAGACCCTTGGGTGATAAA
8723	ZNF470	TCTTCTCCAAATGAAGAGCC
8724	ZNF474	CATGCCACACAAGACGCATT
8725	ZNF474	GTTCATGAATGGCAATTGAC
8726	ZNF474	GGCCTGCGGGCCGGGATCAC
8727	ZNF474	TTCACCTAATGCGTCTTGTG
8728	ZNF474	CTTTACCTCAGACCGCCTCC
8729	ZNF474	GTGGAACCCCAGCCCGACCA
8730	ZNF483	GTCTCAAAAAGCTCCCGACT
8731	ZNF483	GCTATATAAGGAAGTGCTAC
8732	ZNF483	CAATTCAACCCACTTTAGCT
8733	ZNF483	ACTTACACAGGACTCAGTAT
8734	ZNF483	ACAGTAGAATCTTGAGAGAC
8735	ZNF483	CTAGGAATCTATAACATTGA
8736	ZNF569	GTCCTCGTATATGTATAGTA
8737	ZNF569	TCCACATTTACTACAGTCGA
8738	ZNF569	TATAACAACTTAATCACAGT
8739	ZNF569	AAGAAGTATTAAGGAGACAC
8740	ZNF569	CTGCTCAGAGAAAACTGTAC
8741	ZNF569	AGAAGAAGAACCATGGGTGA
8742	ZNF580	GGACCCCATTGGCGTCGATG
8743	ZNF580	CACCTCCTCATCGACGCCAA
8744	ZNF580	CTCCTGGGGGCGCCTCGCGC
8745	ZNF580	GAGCCACCGCGTGTCGCACT
8746	ZNF580	GCGTGGCGCGATGCCGCGAC
8747	ZNF580	GCAAAGACACGGGCGCACTC
8748	ZNF583	GTTGCGCCAAGTTTGCACTC
8749	ZNF583	AAGTGATGAACTATTGCTAA
8750	ZNF583	GCCAGATGTGATCTCATTAT
8751	ZNF583	CTACAGGAGCTTGGTATCAT
8752	ZNF583	GGAACAAGAGGCCCATGCCC
8753	ZNF583	GGCTGTAAATTTCTCTCAAG
8754	ZNF595	GCCCTAAACCAAGCCCTCGC
8755	ZNF595	ACAAATTGTCCAATACCCTC
8756	ZNF595	CCAAGACCTTTCACCAGTGC
8757	ZNF595	ACCCTACAATGTGAAAAATG
8758	ZNF595	TGTGATCTCTAACCCAGACC
8759	ZNF595	CTCATGTATCTTCAAATTGC
8760	ZNF615	GAGCCGACACTTCTTGATGA
8761	ZNF615	GTTCGCTGATGTCTGATGAG
8762	ZNF615	ATTACAGGAATCCCTAACAC
8763	ZNF615	ATCTACTCTCGAATCTGTTC
8764	ZNF615	TTAGATTCAGGAGGTGCATC
8765	ZNF615	CAGCCACATCCTCCAGTGTT
8766	ZNF619	CCTAATGGAGACCCAACTGC
8767	ZNF619	ACCAGATCTGATATTCCAGC
8768	ZNF619	CCAGCCTGCACCCTACGCAG

8769	ZNF619	TTCTTGTGCAGGGCTTGGGC
8770	ZNF619	CAGCCATGCTCCAGACAGTG
8771	ZNF619	GGGACTCACACATAGGCAAT
8772	ZNF670	CACTAGTAATGGGCCTTATA
8773	ZNF670	AGCCCACTCCTCCTGAGTAA
8774	ZNF670	TCATACACTGGACCCTTATA
8775	ZNF670	CAAGATGACTTCAAAAATCC
8776	ZNF670	ATGTGATGCAAGAAATCTTC
8777	ZNF670	TGAAGTCATCTTGGATATTC
8778	ZNF677	ACCAGTGTTCGAACCTCACT
8779	ZNF677	AAGCAAGGGATTATCACCAA
8780	ZNF677	GAACCTGCTTTCTCTCGATG
8781	ZNF677	TATCCTCATCGAGAGAAAGC
8782	ZNF677	GGAGGGAATGGCTCTTTCTC
8783	ZNF677	TGCCTAAGTTTGACAGCCTG
8784	ZNF682	GCCAGAACTGATTAGCCGTC
8785	ZNF682	TTCACATTTATAGCGTTTGA
8786	ZNF682	GTAAAAATTCTTACGTAGTA
8787	ZNF682	GAGACCATAGCCAAACCCCC
8788	ZNF682	TATTCTCACCCAGAGAGACC
8789	ZNF682	GCTAGAGAACTACAGAAACC
8790	ZNF683	CCATAAGATCTAGTGCCCCC
8791	ZNF683	CACATATGTTGCACTCGTAC
8792	ZNF683	CTGTACGAGTGCAACATATG
8793	ZNF683	CCAGGGGGCACTAGATCTTA
8794	ZNF683	TGCACTGCTGGCCTGCCTAC
8795	ZNF683	AGGATCTCACCGAGCACTTG
8796	ZNF684	TATGCATAAAGCCCAACTCG
8797	ZNF684	TCACCTGGAGAGCTCTCGTG
8798	ZNF684	TACCCACTTGTTGATGAACC
8799	ZNF684	AATCCACACGAGAGCTCTCC
8800	ZNF684	GTGCTGAGAGAACCCTGTAT
8801	ZNF684	GGCTGTGGATTTCACTGCAG
8802	ZNF707	ACTCGTAGAACCTCTTCGTC
8803	ZNF707	TGGTTCCCGTGTGCACCGTC
8804	ZNF707	ACTGTTCCAGGCGAGAGACG
8805	ZNF707	CTCTCCCGGTCTTCAACCCA
8806	ZNF707	ACGTGGCCATCTACTTCTCA
8807	ZNF707	CTCCTCCCTTGAGAAGTAGA
8808	ZNF778	CACTTCGGCAGGATAGATCT
8809	ZNF778	GGGTTCTTATGTGCACGATA
8810	ZNF778	CGCGGTGACCTTTGACGACG
8811	ZNF778	CAGTAGATAAAACCCACCGC
8812	ZNF778	GATCTATCCTGCCGAAGTGC
8813	ZNF778	GTCCACAGCCACGTCGTCAA
8814	ZNF821	GTTGATTGCTCACGTGTACC
8815	ZNF821	GCTAGAAGAGCAAGCCCGCC
8816	ZNF821	AGTCCTGATCTCTTAGTTGT
8817	ZNF821	GAACGAAGTGGTAGAGCACG

8818	ZNF821	ATCAGGGAAACATGTCCCGT
8819	ZNF821	ACCACTTCGTTCCCACCAAC
8820	ZNF827	ACCGAGGCAAAGTCGTGCTT
8821	ZNF827	GATTGTCCTTGCGAGCACAC
8822	ZNF827	CCCTAAGCACGACTTTGCCT
8823	ZNF827	TGATATTCTTCCGTGCAGCA
8824	ZNF827	CCTTTCCAGTGTCCAATATG
8825	ZNF827	CCCAGTGACTCCGTGCTGCA
8826	ZNF829	TTCAGGGGCCGGTGATGTTC
8827	ZNF829	AATTCATCTGATCAGCGTCC
8828	ZNF829	TACCCACCACACATGAGGAA
8829	ZNF829	ACTTCTACAAGCAGTATCCA
8830	ZNF829	TGATCTCCATTCCTCATGTG
8831	ZNF829	CTCCAGGTGTCACCCAGAAG
8832	ZNF836	AATCAACACTAGCACGTCAT
8833	ZNF836	CTTTAGAGTGCGTTCAATTC
8834	ZNF836	GTCAGGGATGGCTCTTACAC
8835	ZNF836	CAGTACAAAGCTTTCTGCAC
8836	ZNF836	TTCAGGGACCTTTGACATTC
8837	ZNF836	ATTCCACTTCGAACCTTGCA
8838	ZNHIT1	CGAGCTGAGGGAGTCCCGCG
8839	ZNHIT1	GCCTCAGTTTGATGACGATG
8840	ZNHIT1	TCGCTTCCGAAAAAACTTTC
8841	ZNHIT1	CCGCGATGGGGGTCCCGCAC
8842	ZNHIT1	CCTGTGCGGGACCCCCATCG
8843	ZNHIT1	CTGTCTGCAGGTGTCTGAAG
8844	ZNRF3	TCCCCGCTGTACTGCTCGCT
8845	ZNRF3	GCTCTGAAGACCCGCTCAAG
8846	ZNRF3	TTTCCTGGCTTTCTTCGTCG
8847	ZNRF3	AGGCCAAGCGAGCAGTACAG
8848	ZNRF3	ACCCACTGAATACTTTGACA
8849	ZNRF3	GCACCCTTCACATACACCAC
8850	ZSCAN2	TCCGCTGGTGCACTACGAGC
8851	ZSCAN2	ATCCAGCCTTAACACGCATC
8852	ZSCAN2	GAAACAGCTTCCTGCGTACA
8853	ZSCAN2	TCCGAGAGCTCTGCCGGCGC
8854	ZSCAN2	CACAGGGCAGCCATGTACGC
8855	ZSCAN2	AGGCGGCCGAGTGCACCCTG
8856	ZWINT	GGAAATCCGCTACCTGAAGC
8857	ZWINT	TGCTAAGGGTCTCGACCCCT
8858	ZWINT	GGAGTTGTGTCCGTTTCCTC
8859	ZWINT	GGCCTCTACGTGCTCCCTGT
8860	ZWINT	ATACCACCACAAACTCAACC
8861	ZWINT	CTACCTGTTGTAGCTGCCAC
8862	ZYG11B	AGGTGGCTGATCGACTGCTT
8863	ZYG11B	CACTGAAGCGTTACAGTGAA
8864	ZYG11B	ACACAATGTTCCATCTTGTC
8865	ZYG11B	AGGCTGCATGCCCGAGGACC
8866	ZYG11B	TTCTGCAATCTGAGTTTCAT

8867	ZYG11B	CTCCCTCACCATGGCTGCGC
8868	ABRAXAS2	TTTCCCATAGGTATAATCAG
8869	ABRAXAS2	TGGAGTGATGAAAGACATCA
8870	ABRAXAS2	TAAGAAGACAAATCACTCAG
8871	ABRAXAS2	GATTCTTAAAGATCGGAGAA
8872	ABRAXAS2	AGCTGAAGAGAAGAAAGACG
8873	ABRAXAS2	AACAGCAACGCGGACCACGT
8874	ACTN1-AS1	TTATTAGAGATCCCGCACGA
8875	ACTN1-AS1	CTCTGGGGACCGCTCCACAG
8876	ACTN1-AS1	CGACCAAAACTACCTTCGCG
8877	ACTN1-AS1	CACGCGCGCTCCAAACCCCG
8878	ACTN1-AS1	ATGTGCACAGCCATCCTGTG
8879	ACTN1-AS1	AGATTTACCTAGATCACTCG
8880	ADAM5	TATGATGAAATTAATACCAG
8881	ADAM5	GTGTGTCTACATACCTACCC
8882	ADAM5	CTCATATGGAATTGAACCGA
8883	ADAM5	CCTTACTGCCAACCAAAGCA
8884	ADAM5	ATATGCATTTCAATATATCG
8885	ADAM5	AAATGCCAACCTACACACAC
8886	ADAMTS9-AS2	TGAGTTGGTATATTACAGGT
8887	ADAMTS9-AS2	TATACTCAAACAGATCACAT
8888	ADAMTS9-AS2	GTGAATTGGCCAAAAATCTG
8889	ADAMTS9-AS2	GCAGGTCCAGGTGTTACTTG
8890	ADAMTS9-AS2	CTGATATGTATTGCAACCAG
8891	ADAMTS9-AS2	AGGCATAACCACCCCCAGAG
8892	ADGRB2	TGAGCAAGACCTCGACAAAG
8893	ADGRB2	CCAGGCTCATCTGCAGACCT
8894	ADGRB2	CAGCCACTGATAGCAAGTGG
8895	ADGRB2	CACTGCACAGCCGATCACCA
8896	ADGRB2	ATCATCAGCCGAGGGCACGT
8897	ADGRB2	AGCACCGATCACAAAGTAGG
8898	ADGRL3	TTACAACACTGACCAAAGCA
8899	ADGRL3	TGTGTTATATGATTGACCCA
8900	ADGRL3	CACAGTATGAAGCAACATGG
8901	ADGRL3	CAAAGTTGTGGTCCGAAGGG
8902	ADGRL3	AGGCATCTGGCAGATAACAT
8903	ADGRL3	ACAGGTTATTGGTCAACACA
8904	ADGRL4	TGAATTTGTATCAAACTCTG
8905	ADGRL4	TGAAGCCTGCTATTGCAACA
8906	ADGRL4	CTTACAAGGATCACTCAACT
8907	ADGRL4	CTGCATATGATTCAAATGGT
8908	ADGRL4	ATTTACATTAAGTCATCGAA
8909	ADGRL4	AATGATGGAACCGTCTGTAT
8910	ADGRV1	TTATTGGTCATCCGTGCACA
8911	ADGRV1	TCACCCGTATCACCACAGAA
8912	ADGRV1	TATGGTCCACTCAAGTCGGA
8913	ADGRV1	ATATCCTTGAGCATCAAGCA
8914	ADGRV1	AGGATAAGTATCGTTCACCT
8915	ADGRV1	AAGATTGGATGGGATACCAG

8916	AFAP1-AS1	TCTGAGGAATGGTTATCCTG
8917	AFAP1-AS1	TAAAAACACTGGACTCAGAG
8918	AFAP1-AS1	GGGATGTATACGCTTCCTTG
8919	AFAP1-AS1	CCTGGTTCAGAAGAGACACG
8920	AFAP1-AS1	CCAGAATCTACTGCGTCCTG
8921	AFAP1-AS1	CAGAAACGCCGTTACTCCCG
8922	AGAP2-AS1	GTCAAGGTAAGAGTTTGAGG
8923	AGAP2-AS1	GGTCTGCGGAAGGAGCGTAG
8924	AGAP2-AS1	GACCCAGAGCAATCCGAGTG
8925	AGAP2-AS1	CACGGCCGAAGTCGTGTGAG
8926	AGAP2-AS1	AGGAAGCCCGAGCACATGCA
8927	AGAP2-AS1	AAGGGACAGAGTTCAAGCAG
8928	AGGF1P2	ATTACAGACTCTCATACCGA
8929	AGGF1P2	ATGATTCATCTTTCTTGTCA
8930	AGGF1P2	AGGAAGATGAGGAAAAAGTG
8931	AGGF1P2	ACTGGGACAAAGCTCAGGAG
8932	AGGF1P2	ACTCTTCAAATCCCTGAAGT
8933	AGGF1P2	AATCCTGCATGTGTTTGCCA
8934	AKR1D1P1	TGTTAAAACTGGATACTCCG
8935	AKR1D1P1	TGGTGATTCGATGATCAATG
8936	AKR1D1P1	GGACCACTAGGAATCCAACC
8937	AKR1D1P1	GCCTAAATTGACCCCTAAGG
8938	AKR1D1P1	GAGCCTAGAGAGGATGCTCA
8939	AKR1D1P1	CCCTAAGGTCCTCCACACGT
8940	ALOX12P2	TGTGCTGAGACAATTCCAGG
8941	ALOX12P2	GGGAAGGCACCAAACCATTG
8942	ALOX12P2	GAGGAGGACGGAATACTCAA
8943	ALOX12P2	CTCTCTGAAGAATGTCCACG
8944	ALOX12P2	CACCTGGAGATCTTAAACCG
8945	ALOX12P2	AGGCAGGAATAGGCTACCTG
8946	ANAPC1P1	GTGCAGCATGCTGAACATAG
8947	ANAPC1P1	GGACAACGAAGATTGCAGCC
8948	ANAPC1P1	GCTAGTGGCCACATTCCGTG
8949	ANAPC1P1	CTTTGTGGGATCCCTTCAGG
8950	ANAPC1P1	CACAGGAAATTTGCCCACAT
8951	ANAPC1P1	ATTGTAAATGGATACTAACA
8952	ANKRD31	TACATCACCAGAAAGCACCC
8953	ANKRD31	GCACTTCATGAAGCTAGTGT
8954	ANKRD31	GAATTAACTAATAACATCAG
8955	ANKRD31	CATTGACCCACTAGACATAG
8956	ANKRD31	AGGTTGGACACCACTTCATG
8957	ANKRD31	AGATTGTACACAAATAGCAG
8958	ANP32BP3	TGACAAAGATGAAGTCAGCG
8959	ANP32BP3	TACCAAGAGGGACTAAATGG
8960	ANP32BP3	GTGAGACTCTTGCTGTAGCG
8961	ANP32BP3	CTTGGATACCTATGACCCAG
8962	ANP32BP3	AAGGACTAGGAGAATGAGGA
8963	ANP32BP3	AACCTACATTTATTAAACGG
8964	ASB16-AS1	GTGGAGACAGAGTAGACTGT

8965	ASB16-AS1	GTCCTGAAGGAATAGGCCTG
8966	ASB16-AS1	GTAACGAATCCAATCAGCCG
8967	ASB16-AS1	CAATTCTGTTGTCTGTAGCG
8968	ASB16-AS1	CAATCCTAGCCTAATCTGGG
8969	ASB16-AS1	AAAATGTTAGGAGATAAGGC
8970	ASNSP1	TTGTAATAGAGCACGTGTGG
8971	ASNSP1	TGGGCATTTATATAATAAGG
8972	ASNSP1	TCAGTGGCATCGATCCACTT
8973	ASNSP1	GTGCTATGTAGATTGCACAC
8974	ASNSP1	GGGCCAAATGCCACACGTGG
8975	ASNSP1	GGAACAATGAAAGTCAACTC
8976	ATP5F1A	TTATTGGTGACCGACAGACT
8977	ATP5F1A	GGCCGTAGCCGACACCACAA
8978	ATP5F1A	CATTCCTCAGCCCATGTACG
8979	ATP5F1A	AGGATAGGCCTCACGACCAG
8980	ATP5F1A	ACCAACTGGAACGTCCACAA
8981	ATP5F1A	AAATTCGAGGAATGATACCG
8982	ATP6V0E1P2	TAGAATAAGACATTTAAATG
8983	ATP6V0E1P2	GGACCCTTAGGGATAAACCA
8984	ATP6V0E1P2	CCATGAGGCCATAATATGCA
8985	ATP6V0E1P2	AATTTGGGAAATGAAGCGGT
8986	ATP6V0E1P2	AATGGGCAAGTCAAGAAAGG
8987	ATP6V0E1P2	AACATGCTCATCACAGCGAA
8988	ATXN2-AS	TGTGTCCCCTGAATCTGGCA
8989	ATXN2-AS	TGGAGTGGAGCAACCCTCCG
8990	ATXN2-AS	TCAGCATCTTCACTTCTGCG
8991	ATXN2-AS	GGGGACGGCAGGAAAACGGC
8992	ATXN2-AS	AGGAAAAAGAAGTTCAAGTG
8993	ATXN2-AS	AAGACACATTCTCCGCCCCA
8994	B4GAT1	GGCGATTACCGCGTCTACAG
8995	B4GAT1	GCTGGATCAGAGCAACCAGT
8996	B4GAT1	GACCTGGTCCACGGACCGTG
8997	B4GAT1	CTATGCCCTGGTGATCGATG
8998	B4GAT1	CGTGTGCCCCTCGCGTTACG
8999	B4GAT1	CCCAGCGCATAATTAATCCC
9000	C1GALT1P2	GTAAAACAGGGCTGTATGAG
9001	C1GALT1P2	GATAATGGACAGAATCATCT
9002	C1GALT1P2	CTGCCGGAGACTGATCTGGG
9003	C1GALT1P2	CAGAAATACACTTTCCGAGA
9004	C1GALT1P2	AGAGGGCGACGACATGGGAC
9005	C1GALT1P2	AGAGCACTGGAATCCCAGTG
9006	C1orf143	TTAGAAAACTGTACCCCAGA
9007	C1orf143	TGTGGAAAACATCTATCGGC
9008	C1orf143	GGAACCTTTATCAGATAGCA
9009	C1orf143	GAAGGAAGAGCAATTGGTTG
9010	C1orf143	AGCTTCCCATATAGCCCTGG
9011	C1orf143	AAATAGTGTAATCTACCTGA
9012	C1orf220	GGGCCACGAAGGATGGACGA
9013	C1orf220	GCTTCCAAAATGGTGCCTTG

9014	C1orf220	GAACTCTACACTAAATGTGG
9015	C1orf220	CTTGTTTCTTGGCACCACAT
9016	C1orf220	CTCTGCAAATAGCTGCACCT
9017	C1orf220	AGGACTAAGATACCATTCCA
9018	C1QBPP2	TTAGTGCGGAAAGTTGCTGG
9019	C1QBPP2	TGGATTTCCTTGCGGACCAA
9020	C1QBPP2	GGGGAGTTGATATCAATTCA
9021	C1QBPP2	GAGAGGACTGCGAACATGTG
9022	C1QBPP2	CTTCCATTCAAACTCGCTGG
9023	C1QBPP2	ATAATGACAGTCCAGCACAA
9024	C20orf203	GTGGACTCGCTGAGCCTTTG
9025	C20orf203	GAGTCCAGCCAAGACTGATG
9026	C20orf203	CTGTGATCCAGCATGTTGGG
9027	C20orf203	CCCCACCGCCAAGGTCCCAG
9028	C20orf203	CAAGGTGGGTCGGAGAGATA
9029	C20orf203	ACCCAGGCCCTTATCAGGAA
9030	C3P1	GTCAAGTTGGTACCTCGATG
9031	C3P1	GAACATGGTACCCGACACGG
9032	C3P1	CTCTGCCAAAGAGTAAATCG
9033	C3P1	CAGGTTCCAGAACAATTGAG
9034	C3P1	ATGTGAATGACAGATGCATA
9035	C3P1	ACAGCAATGCCTACCAACAG
9036	C8orf49	TGTCTTCCGTCCTACCGCTG
9037	C8orf49	TGCTCGTGATCGTAAGCTCC
9038	C8orf49	CTTCTCTGGCTAAAACCCGG
9039	C8orf49	CACTCGTGGACCAAGTGCAT
9040	C8orf49	AATACTATGGACAATACTAG
9041	C8orf49	AAGTATGCGTCATGCCTGCG
9042	CASC20	TATAATTTACAATATGATGA
9043	CASC20	ATTTCCAGACACAAATTGTG
9044	CASC20	ATAGGCATAGATACTAAGAT
9045	CASC20	AGGGTTACCTGATTGAGGAG
9046	CASC20	AGGATTGATACGTAAAAACG
9047	CASC20	AGGAAATGCATGGTCTCACA
9048	CBX3P4	GAGGTGGAAAGATTCAGGTG
9049	CBX3P4	GAAAATGGATGTTTCTCATG
9050	CBX3P4	ATTGGTGCCACAGATGGCAG
9051	CBX3P4	AGAAAAGCTTCAATTAACTC
9052	CBX3P4	AAAGCTGGTAAAGAAAAAGA
9053	CBX3P4	AAACCAAGAGGATTTGCCAC
9054	CCDC197	GGGACAACACATACTTAGCC
9055	CCDC197	GGACCACAGGGCTCTCATGT
9056	CCDC197	GGACACGCAGAAGCGCCTCG
9057	CCDC197	GGAAGCTGTGTACATACCCT
9058	CCDC197	CTTCCCGTAGTGCTTCACCG
9059	CCDC197	CCCAAGCAATCCTGGTGACA
9060	CCDC198	GATTGAATGCAAAGTCCACA
9061	CCDC198	GATGTCAAAATACATGTCTC
9062	CCDC198	CTTCCTGAAGTAATTACTCG

9063	CCDC198	CAGGACAATGCACAAAGCAA
9064	CCDC198	AATTAATAAGCAAAGTCCAA
9065	CCDC198	AATGATCCGTAAAAGACAAG
9066	CCDC57	TTCCATCCAGTGAGATCCAG
9067	CCDC57	TGGAGTTTGAATCGAAAATG
9068	CCDC57	GGTGAGCGAGCTCAAGATAG
9069	CCDC57	CTGGGCTGAGACTCAGCATG
9070	CCDC57	CCCTTTCAATGTCATCACAG
9071	CCDC57	ATCAACTGTAAAATCTGCCT
9072	CCDC9B	TGGACAAGGCCAAGTCCACG
9073	CCDC9B	GCAAGCCAGCAGACCCTCGG
9074	CCDC9B	CTTACAATCCGTTTGCCCTG
9075	CCDC9B	CCCAGGGTGAGGCTTACACC
9076	CCDC9B	CAGCTCAGATTCTGCACGGA
9077	CCDC9B	AGGAGATCCAGGAGGACCGT
9078	CCNYL2	TTTGGAGTGATATGGCTGTG
9079	CCNYL2	TGACATGGGAACATTGCTTG
9080	CCNYL2	TATATTATCATATCAAGCAA
9081	CCNYL2	TAGCAGTTTAGCATGTTTGG
9082	CCNYL2	CAGGGCTTGGAACACGTCCA
9083	CCNYL2	ATTAAGCAAATATCTGTACG
9084	CCRL1P1	TGTGCTACTTTATCACGGCA
9085	CCRL1P1	GTGCAATGCTTTCTGTGACT
9086	CCRL1P1	GGGGGTGTGCTACTTTATCA
9087	CCRL1P1	GCCATGGCTCCAATCTCAGA
9088	CCRL1P1	CAATGACGAAAGCTATTGTG
9089	CCRL1P1	ACTAATGTCCCCAGCCAATC
9090	CDC37P1	GGGAGTTAGCTGGGTCGCAG
9091	CDC37P1	GGAGGTGGAGTGGCTACACG
9092	CDC37P1	GCGGAAGAGGTTAGCCCGGT
9093	CDC37P1	CTGGGAGTACAAGTGCAAGA
9094	CDC37P1	ATGTTGGACTACAGCGTGTG
9095	CDC37P1	AAAGAGCATGACCTGGAACG
9096	CDC37P2	GGGTCGCAGAGGCAAAATGT
9097	CDC37P2	GCTACACGAGGAGCTGAGCT
9098	CDC37P2	CTCTTCATCATCGCTCAGTG
9099	CDC37P2	CCAAGAAAAAGAGCATGACC
9100	CDC37P2	AGCGGAAGAGGTTAGCCCGG
9101	CDC37P2	AAGGAGCTGGAGGTGTCAGA
9102	CES5AP1	TGGTTGTGATCGTCCAGTAC
9103	CES5AP1	GGGAGTTTGGGGGATCTCGG
9104	CES5AP1	GGAGTCAATAACCCCGAGTG
9105	CES5AP1	GGAAGTATTCACCAGCCACA
9106	CES5AP1	ATTCTGTCTCCCATGGCCGA
9107	CES5AP1	ATCACAGCTCAGTATCATAG
9108	CFAP300	TCTGAAAGTAACTTCAAATT
9109	CFAP300	GGAACTCTTCTCTATCTGGT
9110	CFAP300	GACATTGAAAGCTGTGTGCA
9111	CFAP300	GAAGATATTGTACGAGACAG

9112	CFAP300	CCTCGGTTCCTCACCACTGG
9113	CFAP300	ATCCTTCCGATAGGACTGAA
9114	CFAP54	TATGCTAAAATGCATGAATG
9115	CFAP54	GAGTGACAGGAGCGGGACCA
9116	CFAP54	CTGTAGTCTTTGAAACCCGG
9117	CFAP54	ATCATCCAAAGGATGCTCAA
9118	CFAP54	AATGCAAATTAGGAATTCAG
9119	CFAP54	AACAACGGAAAGCTTGTCGG
9120	CFAP73	GGCAGCCCTGAAACAGCGTT
9121	CFAP73	GCAGCGCCTCCACCTCCCGA
9122	CFAP73	CCTGCAAAAACTTGTCAAAG
9123	CFAP73	CCCCCGACTCTAGTTCCAAG
9124	CFAP73	AGCTGGAGGCGGCGCGAGCG
9125	CFAP73	AGCTACGGCGGGAACACGCG
9126	CHKB-DT	TGTGATTCGTGATCCCATGT
9127	CHKB-DT	TCCTTGTCGGAGCGCCCCAG
9128	CHKB-DT	CTTGATCCTGATAAACGGTG
9129	CHKB-DT	AGGTAGGAACGCTTCCACGA
9130	CHKB-DT	AGGCCAATCAGTTGGCGGAG
9131	CHKB-DT	AGCGTACGGGAAGACCGGAG
9132	CIPC	TGCTGAGATCACTTCAAAGG
9133	CIPC	GTGAGAAAAAGTCCGACTCC
9134	CIPC	GGCTCACTGGAAGACAAGCT
9135	CIPC	GCCGGTATCCAGCAGTCACG
9136	CIPC	ACCCAGCGCCAAACTTGCCG
9137	CIPC	ACATTCTTCATGACGACCAT
9138	CLLU1	TGTTACCTAACTGTGATCCT
9139	CLLU1	TGGGCCTTCAGACTTAAACC
9140	CLLU1	TCAGGGAAGAAATGGATCGT
9141	CLLU1	GTGGGCCTGTCATGCACACA
9142	CLLU1	AACAGGCATAAACTTCCGTT
9143	CLLU1	AAATATATTCAGTCATAGTG
9144	CLTRN	TACATTGCAAAGTAGGACAT
9145	CLTRN	TAATCCAGATGGGCACAGAT
9146	CLTRN	CTTTCTAAATGACCAAACTC
9147	CLTRN	CTTAGTATCAGAACAGCTCT
9148	CLTRN	CATGCTGAACTCTGTCAACC
9149	CLTRN	AAGCTACCATCGCTTTGAAG
9150	COL26A1	TGAGCGACTGACCACACTGG
9151	COL26A1	TCGGTCACCTTTAGACCCGG
9152	COL26A1	GGTCGGCGGAGTGCTCTCGG
9153	COL26A1	CGTGCGGTAGGACACTCTGT
9154	COL26A1	CCATCACACAGTGACACGGA
9155	COL26A1	AGGGAGTAGAGGGCGCCCTG
9156	COX6CP2	TGAAGTTTACACACCTATCA
9157	COX6CP2	GTAGAAAGCTGCAACCACCA
9158	COX6CP2	CGTCCATTTCTCCAACAGCT
9159	COX6CP2	ATCACAGACGCTTAACCAGA
9160	COX6CP2	AGCTTTCTACAAGATTGCTA

9161	COX6CP2	AGCTAAGAAATAGTTTCTCT
9162	CPEB1-AS1	TGTCAACCATAAGAAAATGA
9163	CPEB1-AS1	GGCAGACAGTGACTCGGCAA
9164	CPEB1-AS1	GCGCGGACCGTTAGCTACTG
9165	CPEB1-AS1	GAGAGCAAAGACTCAAACCA
9166	CPEB1-AS1	ATGATGCGACCTGATACCGC
9167	CPEB1-AS1	AGGGGCTACAGTTCCGACCA
9168	CRYBG2	GTGTCACTCGGTATTCCTCG
9169	CRYBG2	GCCTGTCCGGACAGAGACCA
9170	CRYBG2	GCCTCGAACTACCCTTATGG
9171	CRYBG2	CTTGTCTCTGATCCCACCGA
9172	CRYBG2	CTTGTCCCCCAAGTCAACTG
9173	CRYBG2	CTTCACAACCTCTTTCCAGG
9174	CTBP2P2	TGTTTGCTCACTATACCAGG
9175	CTBP2P2	GCTGTAGGTCACATGATGGG
9176	CTBP2P2	GATTGAACAGAATTTGTGAA
9177	CTBP2P2	ATCCGCGAGGTCAACTCGGG
9178	CTBP2P2	ACTTTACTATAAAGTAGATG
9179	CTBP2P2	AAGCTTAAGAAACTGTGTGA
9180	CYP21A1P	TGTCCGCAGGACGCACCTCA
9181	CYP21A1P	GCATGGCAAGACGCCCGTCA
9182	CYP21A1P	GATTAAGCCTCAATCCTCTG
9183	CYP21A1P	CCAGAGCAGAGACCAACGAC
9184	CYP21A1P	AGGATTGGGGAAGCCCCCGA
9185	CYP21A1P	AGAGAAGAGGGACCACAACG
9186	CYP2D7	GGTCGGCGAAGGCGGCACAA
9187	CYP2D7	GCGCGACAGGATCACCCCTG
9188	CYP2D7	GCACAGCACAAAGCTCGTAG
9189	CYP2D7	GAGAACCTGCGCATAGTGGT
9190	CYP2D7	CAGGTGCCAGAACATTCCCT
9191	CYP2D7	ATGGCCACTATCATGGCCAG
9192	CYP4F24P	TGAACCCCTGACATGTGTGA
9193	CYP4F24P	GGGGTTGTGGAAAGTACTGA
9194	CYP4F24P	GGCCCTATGATCAGCCAAAG
9195	CYP4F24P	GACACATATGTCATGGAACG
9196	CYP4F24P	GAAATCACAATACCCTGAGT
9197	CYP4F24P	AAAAGCGATCAACTTGAAGG
9198	DDX11L2	TGCCCAGTTTCACTAGAAGT
9199	DDX11L2	GGTGGAGAACCTCTGCATGA
9200	DDX11L2	GGGAGATAGACATCCAGGCA
9201	DDX11L2	GGGAATCCCAAAGAAATGGT
9202	DDX11L2	GCTCTGGAGACCTGATGCTA
9203	DDX11L2	ACAGCCGGAGAGCATCGCGA
9204	DGLUCY	TCTATGATCGGCATAGACCC
9205	DGLUCY	GGCTCATGATTGAAATGTGT
9206	DGLUCY	GCCTCATCGTGACCACCAGA
9207	DGLUCY	CTCTGAGATAGCACCTTGAG
9208	DGLUCY	CACAGTTATGACTGACCTGA
9209	DGLUCY	ACACACCATGGCATCCCCGT

9210	DHRS4-AS1	TGAAATGGACACCATTACAC
9211	DHRS4-AS1	GCAGAACATATGTAACCAGA
9212	DHRS4-AS1	GACCGCTCACAGCGGTACCG
9213	DHRS4-AS1	CAAGACCTCACTAACACACA
9214	DHRS4-AS1	ATCTCTGTAGTGAAAGTGGG
9215	DHRS4-AS1	AAAGAGTACTATTGATCCAG
9216	DIAPH2-AS1	TTTGTCCATTTCACAATGTG
9217	DIAPH2-AS1	ATGACTTGATGTCCTCCTTG
9218	DIAPH2-AS1	ATGACCCAAATTCCTCCCAT
9219	DIAPH2-AS1	ATATATATATATGCTGGACA
9220	DIAPH2-AS1	ACTATATCTTGGAAGACGGA
9221	DIAPH2-AS1	ACAGTGTATGCAATGGTATG
9222	DKFZP434A062	GCCCTCAGGTGACAGAACCG
9223	DKFZP434A062	GAAGCCACGTCACACCACAT
9224	DKFZP434A062	CAGGTGACAGGCCTGACATG
9225	DKFZP434A062	ATAAACACGAGGTTAAAAAG
9226	DKFZP434A062	AGTTACGTGATAGCGCAAGG
9227	DKFZP434A062	AGGCTGAGCAGTTAGAACCG
9228	DOC2GP	GAGTGCAAGACCCTTAGGTG
9229	DOC2GP	GACCCATGGTACAGCGACAG
9230	DOC2GP	CTTCGGACACACACCGTTCG
9231	DOC2GP	CACGAGGGTACCTGTCTGGG
9232	DOC2GP	CAAAGCCAATCTGCTGCCAG
9233	DOC2GP	AGCTGTAGCACAGTGACAGT
9234	DOCK9-DT	TCCTCCGGTCTTCGGACGTG
9235	DOCK9-DT	GTGGCACCGATCGGAACCCG
9236	DOCK9-DT	GGTTACAGGCGACATAACCG
9237	DOCK9-DT	GGAGTCAGAAGCCTAAAGGG
9238	DOCK9-DT	CGGGGAGCCTGACTGAACGG
9239	DOCK9-DT	CACAGGGTGGCTGTCGCTAG
9240	DPH6-DT	TAAACAAACAAATTGCACAG
9241	DPH6-DT	GCTTCCGAGAACAATAGCAA
9242	DPH6-DT	CTTTCAGCGCCACTTCATGG
9243	DPH6-DT	ATCATGGTTAAGGTTTACCC
9244	DPH6-DT	AGGCGGCGGAGCGAACACCC
9245	DPH6-DT	ACGGCCTAACCACCTAAGTG
9246	DPY19L1P1	TTAATGTATTAATAACAAGG
9247	DPY19L1P1	TATGAGAAGCTTTCGCGGAG
9248	DPY19L1P1	ATATGTCGGACGGTTACCAG
9249	DPY19L1P1	AGGTTGTGTCCACATCACAC
9250	DPY19L1P1	AACGTGTCAGGGACTATGTG
9251	DPY19L1P1	AAAGAACTGTGGACAGCACA
9252	DRAIC	TCACCCACACGAGCCCAATG
9253	DRAIC	GGGCCCACCCGTAGTATGGT
9254	DRAIC	GCAGGCTGAAAAGTTAAGTG
9255	DRAIC	CATGATGGCCCCTTTATGAG
9256	DRAIC	AGCTTCACAAGTCGAAACCC
9257	DRAIC	AAGGGGTGGTCAATCCTGAG
9258	DUXAP8	TTGGAGTAAGGAATCCTCAC

9259	DUXAP8	TGGCCTTCTATTCCACAGCA
9260	DUXAP8	TAATTGAAAGGTGCTATAGG
9261	DUXAP8	GGTACAGAAGATACACAAAG
9262	DUXAP8	GAGTAGGAGGCAGGTCTCCG
9263	DUXAP8	ATGCAGAAAGAGAGTTCTGG
9264	ECPAS	TAGTGGTATCAACAGTGTCG
9265	ECPAS	TAGTGATACACGCCACAGTG
9266	ECPAS	GGCACAGCGAACTCTCATGG
9267	ECPAS	GAACGAGCAATCCAAACACT
9268	ECPAS	CATGGCAGTAAGAAGCGTAG
9269	ECPAS	AACTGGTAACGATAAAGTCG
9270	EEF1A1P10	TCTGGTAAAAGGCTAGGAGA
9271	EEF1A1P10	TCTAACGGTGACTGGAGCAA
9272	EEF1A1P10	TCCATCACTGATCAAGAGTG
9273	EEF1A1P10	TCATTGGACATGTAGATTCG
9274	EEF1A1P10	CATGATTACAGGGACATCAC
9275	EEF1A1P10	ATCCAGAACCTCAAACAGCG
9276	EEF1A1P11	TTTGAATGAAGCTCTTCCTG
9277	EEF1A1P11	TACTGTTCTTGTTGGCCGAG
9278	EEF1A1P11	GCCAGGAACCATATCAGCAA
9279	EEF1A1P11	ATTACAGGGACATCTCAGGC
9280	EEF1A1P11	ATGAGACAGACACTTGCGGT
9281	EEF1A1P11	ACCCAGTGTATATGCCAGAA
9282	EEF1A1P12	TGCATGCAATATGAGCCGTG
9283	EEF1A1P12	GTGTCCAATGACGACAATGT
9284	EEF1A1P12	CTTCCATCACTGATTAAGAG
9285	EEF1A1P12	CATGATTACAGAGACATCTC
9286	EEF1A1P12	ATGAGACAGATAGTTACAGT
9287	EEF1A1P12	AAGGAAAGGAGAATGTTCTG
9288	EEF1A1P19	TGCATGCAATGTGAGCCGCG
9289	EEF1A1P19	TCTGGTGGCAAACCCATTGT
9290	EEF1A1P19	GGCACTGCCATCCTTACGGG
9291	EEF1A1P19	GCCACCCAACTCTTAATCAG
9292	EEF1A1P19	GATGAAGTCTCTGAGTCCTG
9293	EEF1A1P19	ACAGCAAAATGACCCAAAGG
9294	EEF1A1P22	TCTGTCAAGGATCTTTGTCG
9295	EEF1A1P22	TCACCATTCATATCTCCTTG
9296	EEF1A1P22	TACAGGAACCATGTCAACGA
9297	EEF1A1P22	CTACAGCCAGTAGAGATACG
9298	EEF1A1P22	CCAGTACAGGGACGTAGCCA
9299	EEF1A1P22	ATGAGACAGACAGTTGTCAT
9300	EEF1A1P27	TGTAACGTCGACTGGAGCAA
9301	EEF1A1P27	GCGCACATGCTACTGTGTCA
9302	EEF1A1P27	GACACCTCAGTCTTTACCAG
9303	EEF1A1P27	CATCCAGACCAAATCAATGG
9304	EEF1A1P27	CAACTGACAAGACCTTAAGT
9305	EEF1A1P27	ATGAAACAGACAATTGCTGT
9306	EEF1A1P7	TCATCACAAACATCTTTACA
9307	EEF1A1P7	GGTGTGAAATAACTAATCAC

9308	EEF1A1P7	GCCACCCTGGTATTAAGCAG
9309	EEF1A1P7	CCCTGTACTAGATTGCCACA
9310	EEF1A1P7	CAAGCAGCAAAGCAACCCAG
9311	EEF1A1P7	ACAGGTTTGCAACAGAACAC
9312	EFCAB10	TTTGAGATGATGGACTCCTC
9313	EFCAB10	TTTCCAAATACTCCGCGGCT
9314	EFCAB10	TATGGATAACTCTAACATTG
9315	EFCAB10	TACCCGGCCGAAAGAAAAGG
9316	EFCAB10	GGAAAAGCATCAGATCAAGG
9317	EFCAB10	AATTGCAAAAGTAACAGGCG
9318	EIF4BP6	TCAAGCACTGGATAAAGACA
9319	EIF4BP6	TAACGATGACGATGTGTACA
9320	EIF4BP6	GAGGCCGCCATGTTGGCAGA
9321	EIF4BP6	CTCCCAACTAACCCCCTTGG
9322	EIF4BP6	ATGGGCCAAAACTAGAACGA
9323	EIF4BP6	ATGAAGACCAATATGACAGA
9324	ELDR	TCTTGCATTGTCACACGGAG
9325	ELDR	GTGTGTAAAAATCGCTCCAG
9326	ELDR	GGTTTGCTGAGATTTAACAG
9327	ELDR	GGCGCTCGCAAACGTCCGGG
9328	ELDR	CGCCACGATGCACTCTAACG
9329	ELDR	CAATTACAGTGTTTAGACGG
9330	ELL2P1	TTAACAAACACAAGTCTCGG
9331	ELL2P1	TCTATCGAATTGGATTTGCA
9332	ELL2P1	CCGACATCTTAAACTCCCAG
9333	ELL2P1	AGTGTGTGCAACAAACGACT
9334	ELL2P1	ACTTTAATAGAACTGAACCA
9335	ELL2P1	AATCCTTTGAAGTATACGAG
9336	ELOA	TGGTCGACGTACATCTGATG
9337	ELOA	TAGGATTCAAAAGACATGGT
9338	ELOA	GCTATAGGATCGGCTCCCCG
9339	ELOA	GAGGATATCAGCTCGAGGGA
9340	ELOA	CTCAGTTCAGAAATTACCCA
9341	ELOA	CCACAAGGACAAACGCCCCG
9342	ELOB	TCGTCGAGGGCATCCTCAAG
9343	ELOB	GCTTCACCAGTCAAACAGCA
9344	ELOB	CTTGGCGTCCGTGAAGATGG
9345	ELOB	CTTCAGTTCGAACACCGTGC
9346	ELOB	AGGACGTGTTCCTCATGATC
9347	ELOB	AGCGGCCTCCTGACGAGCAG
9348	ELOC	TTACTTTACTTTAGATGGAG
9349	ELOC	TGGGAATTCAGGAATCTCGG
9350	ELOC	AGGTCCTTCACAGCCACCAT
9351	ELOC	ACTTTCGATAGCACATGTGA
9352	ELOC	AATTGGGAATTCAGGAATCT
9353	ELOC	AATTGCACCTGAAATTGCAC
9354	EXTL3-AS1	GTCCTTTGCTCCATGCTCTG
9355	EXTL3-AS1	GGCTCTCCTCCAAAGGTACG
9356	EXTL3-AS1	GCAGACTCACAAGATCAAAG

9357	EXTL3-AS1	CATCTCCACACTGGCGAGAG
9358	EXTL3-AS1	AGGAGACACCACAGAAGACA
9359	EXTL3-AS1	ACCGCGTCCGAAGACCGCCG
9360	FAM225A	GGGAACACAGTATTAGACCT
9361	FAM225A	CTGGGCTTGGACTCAAACCG
9362	FAM225A	CATCCTACATCACAAAACAG
9363	FAM225A	CAATAATATAAACATGTAGG
9364	FAM225A	ATGTGCGTACACAAACAGAG
9365	FAM225A	AGCTGTGAAATAGATCATGG
9366	FAM225B	GTCCGGGTATCCTAAGCATG
9367	FAM225B	GGTCCACTAACAAAACTGAG
9368	FAM225B	GCAACGCATGAGTTTGTGAG
9369	FAM225B	CGGACACTCAGGATGCACTT
9370	FAM225B	ATCTGCATAATATTTCACAA
9371	FAM225B	AGAGAGGCTAGACCTGAGGG
9372	FAM83A-AS1	TGCCTCACCATCCATTAGCG
9373	FAM83A-AS1	GGAGCTAAGGAAGTGCTCTG
9374	FAM83A-AS1	CCTCCTAATTGACAGAAGTG
9375	FAM83A-AS1	CCGAGATTGTAAATGACATG
9376	FAM83A-AS1	AGCACCTGATACATTATACA
9377	FAM83A-AS1	AGAGCTGGTGACCCTAGTCA
9378	FER1L4	TGTTCGAATTTCATGACACG
9379	FER1L4	TGCCTGGTGATACCTCAACA
9380	FER1L4	TGAGGTCGTCAGAACCCACG
9381	FER1L4	CGAGGCTTGATGTCAACTGG
9382	FER1L4	CAGCGAAACAGATTGCACCT
9383	FER1L4	CACCATGATCGACCCCACCG
9384	FEZF1-AS1	TTTCGCGGAAAGTTAGAGTG
9385	FEZF1-AS1	GGTTTCCGGGAAGACGCCTA
9386	FEZF1-AS1	CTTGGCGCACCAATGACTCG
9387	FEZF1-AS1	AGACACCGGCAACCGGAGTG
9388	FEZF1-AS1	ACAAGCGTAAAACGAACTGG
9389	FEZF1-AS1	AAACAACCATATTCAAGACA
9390	FLJ46906	TGGGTGACCCATTCCACCAG
9391	FLJ46906	TGAGTCTCAGATACCATATG
9392	FLJ46906	GTCCCACCCTCACCCCACAA
9393	FLJ46906	CTAGCCAGTGCCCAACACAC
9394	FLJ46906	AGACTGGGCCCGACTCCCAT
9395	FLJ46906	AAAAAGTCTCACTGAAGGGT
9396	FLNC-AS1	GGGCAGATGGAACCCCACTT
9397	FLNC-AS1	GCGGTCTGGAAAATTCCCTA
9398	FLNC-AS1	GACGCAGTTACTCATCTCAA
9399	FLNC-AS1	AGTTAATCATGAAACCAGCG
9400	FLNC-AS1	AGAGGGAACAGTCAGTGACT
9401	FLNC-AS1	ACTAACAAAAATGGCTCAGT
9402	FUNDC2P4	TTGGCTGTAACAGTTGTGGG
9403	FUNDC2P4	GAGCAATCAGATACCTACCA
9404	FUNDC2P4	CAGTCTCTACACTCCATCAT
9405	FUNDC2P4	CACTCGGATTTGCTACAGCA

9406	FUNDC2P4	ACGCAGAGGATCCGCGCTGT
9407	FUNDC2P4	AATTTGCTAAGAAACAGCCA
9408	GACAT3	TCACAAATAGCATCTATGCA
9409	GACAT3	GTGATCTCCAACGTTCATTG
9410	GACAT3	GCTGCGAGTTCCATGTGAGA
9411	GACAT3	GCGCATAGGACAGTCTCAAG
9412	GACAT3	AGACCAGTGACCTATTGAAG
9413	GACAT3	AATGCAAATTAAGCACAAAG
9414	GAPDHP14	TTTATTGATGGTACACAAGG
9415	GAPDHP14	TGCCAGTGCTCATTATGTTG
9416	GAPDHP14	TCCTCCGGGAACACGGTGAT
9417	GAPDHP14	GGGAGATTCTCACTGTCATG
9418	GAPDHP14	CTTGAAAAAGTGGTTGTTGG
9419	GAPDHP14	CATGGGGTCATCAACAGACA
9420	GAPDHP45	GCTTGACAAAGTGGTCGTCG
9421	GAPDHP45	GAGGAGGGGAGATTCTCAGT
9422	GAPDHP45	CCTGAAGAGGGAGACACCAT
9423	GAPDHP45	CAGGCCCACAACTGACACGT
9424	GAPDHP45	AGCATCGAAGGTGAAAGAGT
9425	GAPDHP45	AGAGCACGAGAGGACGAGAG
9426	GBA3	TGGATCTACGTGGTACCATG
9427	GBA3	TCTTGATGACACTCAACGCT
9428	GBA3	TAGAAAAATTATGGATCACA
9429	GBA3	GCCTCCATGAGTCAAAAGCA
9430	GBA3	CCAAAGTGAGGGATACCCGG
9431	GBA3	ACCCCGAGTCCCTTACACAT
9432	GGT8P	TCAGAATGTGTCCCCACATG
9433	GGT8P	GTTGCTTTGAGAGAGGCATG
9434	GGT8P	GACAGTAAGTCCTGGCCCCG
9435	GGT8P	CCTTGCCTACCTCCTCACAG
9436	GGT8P	ATGGCTCTGCTGCTCCCATG
9437	GGT8P	AGCAGTGCTCGGAGATTGGG
9438	GLUD1P3	TGCACACTTGTATGTCATCA
9439	GLUD1P3	GTGTGGTGCAAAAAGCCAGA
9440	GLUD1P3	GGCGCCTGGACGACTTTCAG
9441	GLUD1P3	CTTGGGGAGGACTAAGGCAA
9442	GLUD1P3	CGGGAGGGATACGGACCCGG
9443	GLUD1P3	CCGGAGTCACCCAAACCAAA
9444	GSDME	TTCTATGAGTACATCGCCAA
9445	GSDME	TCTTCTGTGTCAAAACGCAC
9446	GSDME	GTATAACTCAATGACACCGT
9447	GSDME	GGAGAAGTGTGGTGGCATCG
9448	GSDME	CAAACTCTCGAAAGACCAGG
9449	GSDME	ATGAAGACTGGCTCTCTACG
9450	GTF2IP20	TTTCTGATGCACTTGAGCAG
9451	GTF2IP20	TCTGGTGCTCAGGTGTGTTG
9452	GTF2IP20	TAAACAAATTAGAAGCCAAG
9453	GTF2IP20	GGTGTTCGGCTGCGTCACTG
9454	GTF2IP20	GGATTGTAGAGGCCGACGGG

9455	GTF2IP20	ATAGAAAACTATGTCTGGCG
9456	GUSBP8	TGCTGGGTACCAACCTCTGG
9457	GUSBP8	GTGGCTGGTGCAGAAGGTGT
9458	GUSBP8	CCCTGTGGGGCTCCGCACTG
9459	GUSBP8	CCACGGCGTCAACAAGCATG
9460	GUSBP8	CAGCCACTATCCCTACACAG
9461	GUSBP8	AGAGCCAGTTCCTCATCAGT
9462	H1-0	GTATATCAAGAGCCACTACA
9463	H1-0	GGTGTCCTCAAGCAGACCAA
9464	H1-0	GAAGGCATCCAAGCCCAAGA
9465	H1-0	CTTCTGAATGGACTGGCGCG
9466	H1-0	CGACGAACCCAAGAAGTCAG
9467	H1-0	CAAGTATTCAGACATGATCG
9468	H1-1	GAAGGCAAAGAAACCTGCTA
9469	H1-1	CTTCTCCACGTCGTAGCCTG
9470	H1-1	CGATCAGCTCTGACACGGAA
9471	H1-1	CCTGGTAAGCAAGGGAACGT
9472	H1-1	ACCACCACGCTCCTTAGAGG
9473	H1-1	AAAGGTGGCTACAAAAACTA
9474	H1-10	TGGTGGTGGAGACCATCCGT
9475	H1-10	GTTGAGCTTGAAGGAACCGT
9476	H1-10	GCTGGCCAAGATCTACACCG
9477	H1-10	GCGCCTTGATCGAGTACTTG
9478	H1-10	CCTGCCAGTGACGACCGCCG
9479	H1-10	ATTCTTCCTCTTCTTAGATG
9480	H1-2	TCCGCTACGCTCTTTAGAGG
9481	H1-2	GGGGGACCAGACGCCTTACG
9482	H1-2	GGCTGCTCTGAAAAAAGCGT
9483	H1-2	GGCACTCTGGTGCAAACGAA
9484	H1-2	CAAACCTAAGAAGCCAGTTG
9485	H1-2	AACAACAGCCGTATCAAACT
9486	H1-3	GTGATAAGCTCAGATACTGG
9487	H1-3	GCAGCTTCTAAGGAGCGCAG
9488	H1-3	CTGGTGCAGACCAAAGGTAC
9489	H1-3	CGCGCTTAAGAAAGCGCTTG
9490	H1-3	AGGCTGGCGCAGCCAAGCCT
9491	H1-3	AACAACAGCCGTATCAAGCT
9492	H1-4	TTTGAAGGAACCCGACGCGC
9493	H1-4	TAAGGCTAAAAAGGCAGGCG
9494	H1-4	GTAATGAGCTCGGACACCGG
9495	H1-4	GGCCGCTCTCAAGAAAGCGC
9496	H1-4	AAGAAGGCCCGCAAGTCTGC
9497	H1-4	AACAACAGCCGCATCAAGCT
9498	H1-5	TAAAGCTAAGAAGCCCGCGG
9499	H1-5	GCTGCTTCTAAGGAGCGCAA
9500	H1-5	CTTTAAACTCAACAAGAAGG
9501	H1-5	CTGCTAAGCGCAAAGCGACG
9502	H1-5	AGCGCCGGTGGAGAAATCCC
9503	H1-5	AAGAAGGCCTTAGCGGCCGG

9504	H1-6	TTCCAACCAAGAAGCGAGGG
9505	H1-6	TCCTAAATCTACCAGAAGCA
9506	H1-6	TCAGTGTCACAGGAACGAGT
9507	H1-6	GGTTGCGCTCAAGAAGGCAT
9508	H1-6	CTCAAGAGCTTAGTGAACAA
9509	H1-6	AAGGGCCTCGGTGATCAACT
9510	H1-8	GCTTACTTTGAAGCTGCCAG
9511	H1-8	GCACCATGCGTAGCACCGGG
9512	H1-8	GAGAAGGCTCGCAAGCAAGG
9513	H1-8	CTCTCCTCAGGCCCGAGCCA
9514	H1-8	CCTGCACAAGTACCCAACAG
9515	H1-8	AAGGAGGACCCTCCCAACGT
9516	H1-9P	GGATCAAGGACGGTCACCCA
9517	H1-9P	GCTTATCAAGGGGGTTCGAA
9518	H1-9P	GAGGCTACCAGAATTCTGTG
9519	H1-9P	CCACTGCACTGAGCCAATGG
9520	H1-9P	AAGACACTTCACCTACCCCA
9521	H1-9P	AACTCAAATACTGCCATGGG
9522	H2AB1	TATTGAGTACCTGACGGCCA
9523	H2AB1	GTGGAGCGCAGTCTACGGGA
9524	H2AB1	GGTACTCAATAACCGCAGCG
9525	H2AB1	CTGCTCTCGCACCGTCCGAG
9526	H2AB1	CGCTCAGCGCCTGAGTCGCA
9527	H2AB1	CGCCAGCACCGGAGGACCCT
9528	H2AB2	GGCGCCGGTCTACCTCGCTG
9529	H2AB2	GCCCGCCAGCTCCAGGACCT
9530	H2AB2	GACTCAGGCGCTGAGCGTAG
9531	H2AB2	CTGAAAACGAAAGCTCCGCT
9532	H2AB2	CGCCGAGGGTCCTCCGGTGC
9533	H2AB2	CCCGTAGACTGCGCTCCACC
9534	H2AB3	TGCGGTTATTGAGTACCTGA
9535	H2AB3	GGTGGAGCGCAGTCTACGGG
9536	H2AB3	GGCCAAGGTCCTGGAGCTGG
9537	H2AB3	GCGCCTGAGTCGCACGGCGC
9538	H2AB3	CGAGGGTCCTCCGGTGCTGG
9539	H2AB3	AAACGAAAGCTCCGCTCGGA
9540	H2AFZP1	TTTGAATCACAGTGCGCCGA
9541	H2AFZP1	TTCATCGACACCTGAAATGT
9542	H2AFZP1	TGCATCAAAAGACTTAAAGG
9543	H2AFZP1	CTGGGAAGAAGGGACAACAG
9544	H2AFZP1	CCTTTATTGAGCATATCCAC
9545	H2AFZP1	ACCATATTTAAGGGTCACTG
9546	H2AW	TGCGGAGCAACCGGTGCACG
9547	H2AW	TCCGGTCGTGGTAAGCAGGG
9548	H2AW	TAAGTCGCGCTCGTCGCGCG
9549	H2AW	GGCAACTATTCGGAGCGCGT
9550	H2AW	CCTGGAGCTTGCCGGCAACG
9551	H2AW	CACCGCGGCCAGATAGACCG
9552	H2AX	TTCGCGTCTTCTTGTTGTCG

9553	H2AX	TCCCAGTGGGCCGTGTACAC
9554	H2AX	GCTCCAGGATCTCAGCGGTG
9555	H2AX	CGGCGCGCCAGTGTACCTGG
9556	H2AX	CGCCAACGCGCTCGGCGTAG
9557	H2AX	CCGCGGCAAGACTGGCGGCA
9558	H2BC1	TGTCGTTAAGACCCAGAAAA
9559	H2BC1	CTTTGAGCGTATAGCGAGCG
9560	H2BC1	CTCAAAGATATCAGTGACGA
9561	H2BC1	CATCTACAAAGTGCTAAAGC
9562	H2BC1	AGGTGCTACCATTTCCAAGA
9563	H2BC1	AAAGCGCAAGAGGACCCGTA
9564	H2BC10	GGTGACCAAGGCACAGAAGA
9565	H2BC10	GCTTGTTATAATGCGCCAGG
9566	H2BC10	GCATCTCGTCCAAGGCTATG
9567	H2BC10	CTCGAAAATGTCGTTGACGA
9568	H2BC10	CTATTCCGTGTACGTGTACA
9569	H2BC10	ACACGGAATAGCTCTCCTTG
9570	H2BC11	GAAAAAGGGCTCCAAGAAGG
9571	H2BC11	CGCAGGTGAGGCTTCCCGCC
9572	H2BC11	CAGAACCTTGTACACATAGA
9573	H2BC11	ACTAAGGCGCAGAAGAAAGA
9574	H2BC11	ACGAAATGCCGGTGTCAGGG
9575	H2BC11	AAACGAATTCATGATGCCCA
9576	H2BC12	GTCCGCTCCCGCGCCCAAGA
9577	H2BC12	CTTCTTCTGCGCCTTAGTCA
9578	H2BC12	CTCGAAGAAAGCCGTGACTA
9579	H2BC12	CGTGACTAAGGCGCAGAAGA
9580	H2BC12	CGACATCTTCGAACGCATCG
9581	H2BC12	CATGATTCCCATGGCCTTAG
9582	H2BC13	TAGAAGAGATGCCGGTGTCG
9583	H2BC13	GTCTGCTCCCGCCCCGAAGA
9584	H2BC13	GGTGACCAAGGCCCAGAAGA
9585	H2BC13	GGCATCTCTTCTAAGGCCAT
9586	H2BC13	CTTCGAGCGCATCGCAAGCG
9587	H2BC13	CTACTCCGTGTACGTGTACA
9588	H2BC14	GTTCATGATTCCCATAGCCT
9589	H2BC14	CTCAAAGATGTCGTTGACGA
9590	H2BC14	CTACTCTGTGTATGTGTACA
9591	H2BC14	CGCCGGAGAAGCGTCACGCC
9592	H2BC14	CCCTAAAAAAGGCTCCAAGA
9593	H2BC14	ACACAGAGTAGCTCTCCTTG
9594	H2BC15	GCCTTTCTTCGGGGCAGGAG
9595	H2BC15	CTCGAAGATGTCATTGACGA
9596	H2BC15	CGCCGGCGAGGCTTCCCGCC
9597	H2BC15	CGACACCGGTATCTCGTCCA
9598	H2BC15	CCCGAAGAAAGGCTCCAAGA
9599	H2BC15	ACAAAGGCCCAGAAGAAGGA
9600	H2BC17	TTACTCTATCTACGTGTACA
9601	H2BC17	TCTGCTCCTGCCCCCAAAAA

9602	H2BC17	CTCCAAGAAAGCCGTAACCA
9603	H2BC17	CTCAAAGATGTCATTGACGA
9604	H2BC17	CGACACCGGCATCTCATCGA
9605	H2BC17	AGATAGAGTAACTCTCTTTG
9606	H2BC18	TGTTACGAAAGTGCAGAAGA
9607	H2BC18	GATCTCGCGGGATGTGATGG
9608	H2BC18	CTACTCCGTTTACGTGTACA
9609	H2BC18	CAGCACCTTGTACACGTAAA
9610	H2BC18	CACATCCCGCGAGATCCAGA
9611	H2BC18	ACGAAAGTGCAGAAGAAGGA
9612	H2BC19P	TTCGCGCCGGCTCCCAAGAA
9613	H2BC19P	TCACGTCCCGGAGATCCAGA
9614	H2BC19P	GGCATCTGGTGCAAGGCCAT
9615	H2BC19P	GCGAGCGCCAGGTCCCGGCA
9616	H2BC19P	AGCAGCCGGCGACCCGCGGA
9617	H2BC19P	ACGTGTACAAGGTGCTGAAG
9618	H2BC21	TGAACCGGCAAAATCCGCTC
9619	H2BC21	CTACTCCATCTACGTGTACA
9620	H2BC21	CGCGGGAGAGGCTTCCCGCC
9621	H2BC21	CAGCACCTTGTACACGTAGA
9622	H2BC21	AGATGGAGTAGCTCTCTTTG
9623	H2BC21	ACCAAAGCCCAGAAGAAAGA
9624	H2BC3	TTCTAAGAAGGCTATCACTA
9625	H2BC3	TCTGCTCCAGCCCCTAAAAA
9626	H2BC3	GCATCTCATCCAAGGCCATG
9627	H2BC3	CTATTCTATCTATGTGTACA
9628	H2BC3	AGCGCTTATTGTAGTGAGCC
9629	H2BC3	AGATAGAATAGCTCTCCTTG
9630	H2BC4	TTACTCTGTGTACGTGTACA
9631	H2BC4	GAAAGAATTCATGATGCCCA
9632	H2BC4	CTTGGAAGAGATGCCAGTGT
9633	H2BC4	CGACATATTTGAGCGCATCG
9634	H2BC4	ACCAAAGCGCAGAAGAAAGA
9635	H2BC4	ACACAGAGTAACTCTCCTTG
9636	H2BC5	GGTGACTAAGGCTCAGAAGA
9637	H2BC5	GCATCTCTTCCAAGGCAATG
9638	H2BC5	CTTCTTGGAGCCCTTCTTTG
9639	H2BC5	CTATTCAGTGTATGTGTACA
9640	H2BC5	ACACTGAATAGCTCTCCTTG
9641	H2BC5	AAGCAGGTCCATCCCGACAC
9642	H2BC6	GCATCTCCTCTAAAGCCATG
9643	H2BC6	CTCCAAGAAGGCCGTGACCA
9644	H2BC6	CGTGTACAAGGTGCTGAAAC
9645	H2BC6	CGCCAGGCGGGAAGCCTCGC
9646	H2BC6	CATGGCTTTAGAGGAGATGC
9647	H2BC6	ATCCGCTCCCGCCCCGAAGA
9648	H2BC7	GAAGCGCAAGCGTAGCCGCA
9649	H2BC7	CTTCGAGCGCATCGCTGGCG
9650	H2BC7	CTCGAAGATATCGTTGACGA

9651	H2BC7	CGTGTACAAGGTGCTAAAGC
9652	H2BC7	CGACACCGGCATCTCATCCA
9653	H2BC7	AAAAAAGGGCTCCAAAAAGG
9654	H2BC8	TTCGAAGATGTCGTTAACGA
9655	H2BC8	TGGATGAGATGCCAGTATCG
9656	H2BC8	TCCGAAGAAGGGTTCCAAGA
9657	H2BC8	TAGCACCTTGTACACATACA
9658	H2BC8	GAAGCGCAAGCGCAGTCGTA
9659	H2BC8	CTTCGAACGCATCGCAGGCG
9660	H2BC9	GGTGACCAAGGCGCAGAAGA
9661	H2BC9	GCTTGTTGTAATGAGCCAGG
9662	H2BC9	GCATCTCCTCCAAAGCCATG
9663	H2BC9	GAAGCGTAAACGCAGCCGCA
9664	H2BC9	CTACTCCGTATACGTTTACA
9665	H2BC9	AAGCAAGTCCACCCCGACAC
9666	H2BE1	TGGCAGAGATGCCAATGTCA
9667	H2BE1	TCCCAGAACTGCGACCCCCA
9668	H2BE1	GTTTGAGCAGCTGGCGTGTG
9669	H2BE1	GCCCGGCTGGCCCAGTACTC
9670	H2BE1	CAGCACCTTGTAGATATACA
9671	H2BE1	AAAAGTCCAAAAAGCGCTGT
9672	H2BS1	TCCGCTCCCGCGCCCAAGAA
9673	H2BS1	GCTTGTTGTAATGCGGCAGG
9674	H2BS1	CTTGTTGTAATGCGGCAGGC
9675	H2BS1	CTTCGAACGCATCGCAGGTG
9676	H2BS1	AGGCGCAGAAGAAGGACGGC
9677	H2BS1	AGCGCTTGTTGTAATGCGGC
9678	H2BU1	TTCTAAAAAGGCTGTCACCA
9679	H2BU1	GCTTGTTGTAGTGTGCCAGG
9680	H2BU1	GACGGCAAGAAGCGCAAGCG
9681	H2BU1	CTATTCTATCTACGTGTACA
9682	H2BU1	ATCGGCTCCTGCGCCCAAGA
9683	H2BU1	AAGCAGGTGCACCCCGACAC
9684	H2BW1	TTCTTTGGTTCATGACATAT
9685	H2BW1	GGTGATCAGCTGTTCCTCAG
9686	H2BW1	GGGCCTCAGCCTTTCCCGGG
9687	H2BW1	GCTCTGCTTCTGGGACGTAG
9688	H2BW1	CAGTTGGAGTGGCACCTGCG
9689	H2BW1	CAGCACCCGGCGGAAATAGG
9690	H2BW2	TTCCTCTGAGACAACCTCGG
9691	H2BW2	GCGCCACGCCAACCGCCGTG
9692	H2BW2	CTTCACCCCCTATTTCCCCC
9693	H2BW2	CGATGCGGTCCAATATGTCA
9694	H2BW2	CAAAGAGGCCAACTCCACGA
9695	H2BW2	ATGGGCCAGCTGACCAGCCT
9696	H3-3A	CGACCGGTGGTAAAGCACCC
9697	H3-3A	CAGCTAGCACGCCGCATACG
9698	H3-3A	AGTCTGCTTTGTACGAGCCA
9699	H3-3A	AGAGGGCGCACTCTTGCGAG

9700	H3-3A	ACTGCCCGCAAATCGACCGG
9701	H3-3A	AAGCACGTTCTCCACGTATG
9702	H3-3B	GAAGCCTCATCGCTACAGGT
9703	H3-3B	CAGCTTCCGGATGAGCAGCT
9704	H3-3B	AGGATTTCAAAACCGACCTG
9705	H3-3B	AGCTGGCCACGAAAGCCGCC
9706	H3-3B	ACTGCTCGTAAGTCCACCGG
9707	H3-3B	AAGCGCTCCCTCTACCGGCG
9708	H3-4	TCCGCAAGTTGCCCTTCCAG
9709	H3-4	GCTGATGCGCGAGATCGCTC
9710	H3-4	GCACCGCTACCGGCCCGGCA
9711	H3-4	CTTGCGGATTAGCAGCTCAG
9712	H3-4	ACTGCGCGCAAGTCAACGGG
9713	H3-4	AAGAGCGCACCTGCCACTGG
9714	H3C1	CTCGCACTAAGCAAACTGCT
9715	H3C1	CCTGCGCAATCTCGCGCACC
9716	H3C1	CCGCTACCGGCCGGGCACCG
9717	H3C1	CAGTGGACTTCTGATAACGG
9718	H3C1	CAGCGGAGCTCTGGAAACGC
9719	H3C1	CAAGGCGCCACGCAAACAGT
9720	H3C10	TCTGGTAGCGGCGAATCTCG
9721	H3C10	GGGCTTCTTGACACCGCCGG
9722	H3C10	GGAGCTCTGGAAGCGCAAGT
9723	H3C10	CTGTACCAGGCCGATAGCGA
9724	H3C10	CAGCTTTCTGATCAGCAGCT
9725	H3C10	ACTGCTCGCAAGTCCACCGG
9726	H3C11	TCTGCGCTTCCAGAGCTCGG
9727	H3C11	GGGCTTCTTGACGCCACCGG
9728	H3C11	CTTGGTACGGGAGATCGCAC
9729	H3C11	CCGCTACCGCCCCGGCACCG
9730	H3C11	CAAACAGCTCGCAAGTCCAC
9731	H3C11	AGCGCTGAAAAGGTAGCTTC
9732	H3C12	GTGCCTGGCCTGTAGCGGTG
9733	H3C12	GGCGCTGAAATGGCAGTTTG
9734	H3C12	CTACCGGCGGCAAGGCACCG
9735	H3C12	CGAGCTCTGGAAACGAAGGT
9736	H3C12	CAGTCGACTTCTGATAACGG
9737	H3C12	AAAAGCGCTCCAGCGACTGG
9738	H3C13	GGTAGCCAGCTGCTTCCTCG
9739	H3C13	GATCGCGCAGGACTTTAAGA
9740	H3C13	CGCGCTCTTGCGGGCCGCTT
9741	H3C13	CCTTGCCGCCGGTCGACTTG
9742	H3C13	CCGTAGACTTCTGGTAGCGC
9743	H3C13	CCGCTACCGGCCCGGCACCG
9744	H3C14	GTAGCGCCGGATCTCCCGCA
9745	H3C14	GGTGGCCAGCTGCTTCCTCG
9746	H3C14	GGCCCGGCACCGTAGCCCTG
9747	H3C14	GAGGAAGCAGCTGGCCACCA
9748	H3C14	CCGGCGCTACCAGAAGTCCA

9749	H3C14	ACTGCTCGCAAGTCGACCGG
9750	H3C15	GCTCTTGCGGGCCGCCTTGG
9751	H3C15	GCCCGGCACCGTAGCCCTGC
9752	H3C15	GCAGGGCTACGGTGCCGGGC
9753	H3C15	CTTGCGGATCAGCAGCTCCG
9754	H3C15	CCGTGGACTTCTGGTAGCGC
9755	H3C15	CAGACTGCTCGCAAGTCGAC
9756	H3C2	TTGGTAGCCAGCTGCTTGCG
9757	H3C2	CTCGTACTAAACAGACAGCT
9758	H3C2	CCGTTACCGCCCGGGCACTG
9759	H3C2	CAGCTTCCGAATCAGCAACT
9760	H3C2	AGAGCTCTGGAAGCGAAGAT
9761	H3C2	AAGAGCGCGCCGGCTACCGG
9762	H3C3	TTAGTAGCAAGCTGCTTGCG
9763	H3C3	GTAGCGACGGATTTCGCGCA
9764	H3C3	GAAACCTCATCGCTACCGCC
9765	H3C3	CCTGGTGCGAGAAATCGCCC
9766	H3C3	CCTGCGTTTCCAGAGCTCTG
9767	H3C3	AGGTTTCTTCACGCCACCGG
9768	H3C4	GCTCTTTCGAGCAGCCTTGG
9769	H3C4	CTGCTCGCAAGTCCACGGGT
9770	H3C4	CGATCTCACGGACTAGACGC
9771	H3C4	CCACCGTTACCGGCCCGGCA
9772	H3C4	CAGTTTGCGAATCAGCAGCT
9773	H3C4	AAGAGCGCTCCAGCCACCGG
9774	H3C6	GCGCAAACAGCTGGCCACTA
9775	H3C6	GCAGAGCCACGGTGCCAGGG
9776	H3C6	CCTGGTGCGAGAAATAGCTC
9777	H3C6	CCTGCGCTTCCAGAGTTCCG
9778	H3C6	CAGCTTCCGGATTAGAAGCT
9779	H3C6	ACGGCTCGTAAATCCACAGG
9780	H3C7	GGGCTTCTTCACGCCACCGG
9781	H3C7	GATAGCGGCGGATTTCACGG
9782	H3C7	CGATCTCACGTACCAGACGC
9783	H3C7	CGAGCTCTGGAAGCGCAGGT
9784	H3C7	CGACAGTACCAGGCCTGTAG
9785	H3C7	ACAGCTCGTAAGTCCACTGG
9786	H3C8	TCTGCGCTTTCAGAGTTCCG
9787	H3C8	TCGCTACCGTCCCGGCACCG
9788	H3C8	GGCGTTGGAAAGGCAACTTG
9789	H3C8	GCTCAGTCGACTTCTGATAG
9790	H3C8	CCTGAGCGATTTCTCGCACC
9791	H3C8	ACTGCACGCAAGTCCACCGG
9792	H4-16	TGTCCCGCAGCACCTTCCGG
9793	H4-16	TGCGCTTGACGCCCCCACGT
9794	H4-16	GTGCTGCGGGACAATATCCA
9795	H4-16	GGTGGCAAGGGGCTGGGTAA
9796	H4-16	GAAGACTTTGAGGACTCCCC
9797	H4-16	ATGTCTGGGCGAGGTAAAGG

9798	H4C1	TGTTGTCACGCAACACCTTG
9799	H4C1	TGTCTGGACGTGGTAAGGGC
9800	H4C1	GGTGTTGCGTGACAACATCC
9801	H4C1	GGAGACTCGCGGGGTGCTCA
9802	H4C1	GATCTCTGGTCTGATCTACG
9803	H4C1	CCCGGCGTGGCGGTGTGAAG
9804	H4C11	TGTCTGGCCGCGGCAAAGGC
9805	H4C11	TGTCGCGCAGTACTTTACGG
9806	H4C11	GTACTGCGCGACAATATCCA
9807	H4C11	GGGAAGGGTCTTGGCAAAGG
9808	H4C11	CATCTCCGGCCTCATCTACG
9809	H4C11	AGATGCGCTTCACGCCGCCG
9810	H4C12	GGCGGGAAGGGTCTTGGCAA
9811	H4C12	GCGGCGAGCAAGGCGCCGGA
9812	H4C12	CATCTACGAGGAGACTCGCG
9813	H4C12	ATGTCTGGCCGCGGCAAAGG
9814	H4C12	AGTCTCCTCGTAGATGAGGC
9815	H4C12	AGTACTGCGCGACAATATCC
9816	H4C13	TGTCTGGGCGCGGCAAAGGC
9817	H4C13	TGTCGCGCAGAACTTTGCGG
9818	H4C13	GTTCTGCGCGACAACATTCA
9819	H4C13	GGCGGGAAGGGTCTGGGCAA
9820	H4C13	CATCTCAGGCCTTATATACG
9821	H4C13	CAAGCCCGCCATCCGACGCC
9822	H4C14	TGTCTCTCAAGACCTTGCGG
9823	H4C14	GTCTTGAGAGACAACATTCA
9824	H4C14	GCATCACCAAGCCTGCCATT
9825	H4C14	GAACACCTTCAGCACACCGC
9826	H4C14	ATTCGGCGTCTAGCTCGGCG
9827	H4C14	ATGTCCGGCAGAGGAAAGGG
9828	H4C15	TGTTGTCTCTCAAGACCTTG
9829	H4C15	GGAAAAGGCTTAGGCAAAGG
9830	H4C15	CTCGGCGTGGCGGCGTTAAG
9831	H4C15	CTCATTTACGAGGAGACCCG
9832	H4C15	CGTCCCGAATCACATTCTCC
9833	H4C15	ACGCCGAGCTAGACGCCGAA
9834	H4C2	GGTAAAGGTTTGGGTAAGGG
9835	H4C2	GGAGACTCGTGGCGTTCTCA
9836	H4C2	ATTCGGCGCCTTGCTAGGCG
9837	H4C2	AGCGTCACCGAAAAGTGCTG
9838	H4C2	AATTTCCGGTTTGATTTATG
9839	H4C2	AAGGCGCCGAATGGCCGGTT
9840	H4C3	TATCCCGGAGCACCTTACGA
9841	H4C3	GTGCTCCGGGATAACATCCA
9842	H4C3	GGAGACTCGAGGTGTGCTTA
9843	H4C3	GCAAAGGCGGAAAAGGCTTG
9844	H4C3	ATTCGCCGTTTGGCTCGGCG
9845	H4C3	AGTCTCCTCATAGATAAGAC
9846	H4C4	TGGCGCCAAGCGTCACCGTA

9847	H4C4	GTATTGCGTGACAATATCCA
9848	H4C4	GGCGGAAAGGGTCTAGGTAA
9849	H4C4	CTCATTTATGAGGAAACTCG
9850	H4C4	ATGTCTGGCCGCGGTAAGGG
9851	H4C4	AAATACGCTTGACGCCGCCG
9852	H4C5	TGGTCGCGGCAAAGGCGGAA
9853	H4C5	GGTCCTGCGAGATAACATCC
9854	H4C5	GGAAAGGGACTGGGTAAAGG
9855	H4C5	CCGGCGCCTTGCTCGTCGCG
9856	H4C5	CATTTCTGGTCTCATCTACG
9857	H4C5	AGGCGCTAAGCGTCACCGTA
9858	H4C6	TGTCACGCAGCACTTTGCGA
9859	H4C6	GTGCTGCGTGACAACATACA
9860	H4C6	GGTAAAGGTTTAGGAAAGGG
9861	H4C6	GAACACCTTAAGAACACCGC
9862	H4C6	CGTCCCGTATCACATTCTCC
9863	H4C6	CGCCGCGTCGGGCCAAGCGA
9864	H4C7	TATCGCTCAGTACCTTGCGA
9865	H4C7	GTACTGAGCGATAATATTCA
9866	H4C7	GGAACACCTTGAACACCCGG
9867	H4C7	CGGCGCTTGGCCCGGCATGG
9868	H4C7	CAAGCGCCGGATAGTGCACT
9869	H4C7	ATGTCTGTTCGGGGCAAGGC
9870	H4C8	GGAGACTCGTGGTGTTCTGA
9871	H4C8	CGTCACGAATCACGTTCTCC
9872	H4C8	ATGTCTGGCCGTGGTAAAGG
9873	H4C8	AGGTGGAAAAGGTTTGGGTA
9874	H4C8	AGGAGCTAAGCGTCATCGCA
9875	H4C8	AATTTCTGGCCTTATCTATG
9876	H4C9	TGTTGTCGCGCAGCACCTTG
9877	H4C9	GTGCTGCGCGACAACATCCA
9878	H4C9	GTATCACCAAGCCAGCCATT
9879	H4C9	GGAACACCTTCAACACTCCG
9880	H4C9	CGTCCCGGATCACGTTCTCC
9881	H4C9	ATGTCAGGACGCGGCAAAGG
9882	HACD1	GTTCAAGAATCTTTATGGTG
9883	HACD1	CGGCGAGCGGAGGCGCCTGG
9884	HACD1	CCAGTCACAATCACAGAAGT
9885	HACD1	ATGGCGTCCAGCGACGAGGA
9886	HACD1	ATGGAAAAAGGAACACACAG
9887	HACD1	ACGACATCGCCATGACCGCG
9888	HAR1B	TCATCCCTGAATGTCCACAC
9889	HAR1B	TCAACACTTGAACAAGCAAG
9890	HAR1B	ATGTGTAATAATCACAACAG
9891	HAR1B	AGAAACTCACACATGATTTG
9892	HAR1B	AATTGTCATGAGTACATACA
9893	HAR1B	AAAAGCAGAAGCTACGTCCG
9894	HGH1	GTAGGAAGGCCCGCGCCGCA
9895	HGH1	GCGACTGAGGTTGGCTAGCG

9896	HGH1	CCTGCACGAGACATTGCTGG
9897	HGH1	CCGGCCGCGCTGTTGGCG
9898	HGH1	CCCTGCGTACAGAGGAGTCG
9899	HGH1	ATCCCTCAGGACATCACGAG
9900	HID1-AS1	GAGGAGAATGCCCATCTGAG
9901	HID1-AS1	CTGCACAGTCTGAATGACAG
9902	HID1-AS1	CCAGGAACTCCCAAGTGACA
9903	HID1-AS1	AGTGATGGGCAAGACCAAAG
9904	HID1-AS1	AAAGCATCGGGATTACAAGC
9905	HID1-AS1	AAAATAATAAAGGCAAGTGA
9906	HIKESHI	TGTTAATTTCTTGCAGGAGA
9907	HIKESHI	GGAACAATCCCATTTCCTGA
9908	HIKESHI	GCAAACAGCTGCACAGCAAG
9909	HIKESHI	CCAGCATAAAAACCACAACA
9910	HIKESHI	ATGGCTCAGCAGACTCCTGT
9911	HIKESHI	ATGCCAGTATGGCAACTCCT
9912	HIST2H2BC	TGCGCTCGAAGATGTCGTTG
9913	HIST2H2BC	TGAAGCCAAAAACATCAACT
9914	HIST2H2BC	CGACACCGGCATCTGGTGCA
9915	HIST2H2BC	CAAGAAAGATTCTTTAACTG
9916	HIST2H2BC	AGAAAATAGTTATCTGTGCT
9917	HIST2H2BC	ACAAAGCAGCCGGCGACCCG
9918	HNRNPA1P21	TTTACCAAACCATGAAACCA
9919	HNRNPA1P21	GGAACGCTCACGAACTGCAT
9920	HNRNPA1P21	CGTGCTTCAACCAGCCAAAG
9921	HNRNPA1P21	CAGAAATACTGTACTGTGAG
9922	HNRNPA1P21	CAAGGTGGATGGAAGAACTG
9923	HNRNPA1P21	AACTCAACAAAGCATAGTGG
9924	HSD17B7P2	TGTACCCCAGAATGTACAGA
9925	HSD17B7P2	TGAGTGCGCGAAGATGCGAA
9926	HSD17B7P2	GGCGTGCAGGAATATGAGCA
9927	HSD17B7P2	GAAATCTAATTTCAGCCTCG
9928	HSD17B7P2	AGGCCCGGAAGAATGACTGC
9929	HSD17B7P2	AAATTACATTATGACCCAGA
9930	HSPA7	TGTAGCGCTCAAGCCCACCA
9931	HSPA7	TCATGAAGCCGAGCAGTACG
9932	HSPA7	GGTGCTGCCGATCATCAATG
9933	HSPA7	GGCTTTCACCTCAAAGACAC
9934	HSPA7	CTGGCGGCAGAGAAACCGCA
9935	HSPA7	CAAACGTCACCTCTATCTGG
9936	HSPA8P1	TTTATAAACACTATTGTACA
9937	HSPA8P1	TGCAATGAACCCCACCAACG
9938	HSPA8P1	GGCACCACATACTCTTGTGT
9939	HSPA8P1	ATGAGTAGAACCACCAACCA
9940	HSPA8P1	AGGTGAGCATACCATGACCA
9941	HSPA8P1	AAGCTGAAGATCAGAAGCAG
9942	HSPC324	GGAGAACACAGGACGTCCAC
9943	HSPC324	GAACAGTAAGTGTGTCCTGG
9944	HSPC324	CTGCGAAGACCAGCACCTTG

9945	HSPC324	CGCTCTGACATCTTCCGTAG
9946	HSPC324	CAGCACGGCGCGCACACACA
9947	HSPC324	AGAACCCTCATCAACCGAGG
9948	IBA57-DT	TAGTAGGTGACACATCTACA
9949	IBA57-DT	GGGAAAGTGGCCACTGTACA
9950	IBA57-DT	GATGCGTAGTAACATCAGTG
9951	IBA57-DT	CTCCTCGAGAAAGCACGGTG
9952	IBA57-DT	CATCATGTCACACTGATTAG
9953	IBA57-DT	AATGACACAACAGCCACACG
9954	IGHG3	TCAGCACCTGAACTCCTGGG
9955	IGHG3	GTACAACAGCACGTTCCGTG
9956	IGHG3	GGTCCAGTTCAAGTGGTACG
9957	IGHG3	CTACTTCCCAGAACCGGTGA
9958	IGHG3	ACACACCTCCCCCATGCCCA
9959	IGHG3	ACACAACTCACACATGCCCA
9960	IGHG4	TCAGCACCTGAGTTCCTGGG
9961	IGHG4	GTTCAACAGCACGTACCGTG
9962	IGHG4	CTTCGTGCCCAAGCTGCTGG
9963	IGHG4	CGTGAGCCAGGAAGACCCCG
9964	IGHG4	CAGGGCGGCTGTGCTCTCGG
9965	IGHG4	ACCAAGGTGGACAAGAGAGT
9966	IGKV1-33	TGGGACCCCTGTTTCCAAAT
9967	IGKV1-33	TGCTAATGTCCTGACTCGCC
9968	IGKV1-33	GCTGCTCTGGCTCTCAGGTA
9969	IGKV1-33	CATCGTAGATCAGGAGCTTA
9970	IGKV1-33	CATCACTTGCCAGGCGAGTC
9971	IGKV1-33	ATCGTAGATCAGGAGCTTAG
9972	INTS6P1	GTTGTAAACAATCATATCGG
9973	INTS6P1	GGCATAGACAACTATGGGCA
9974	INTS6P1	CCTTATTCAGCAAAGCAACG
9975	INTS6P1	CCAGCCTTGATAGCACAGGG
9976	INTS6P1	AGCCGTCTGGTGTGATTGCA
9977	INTS6P1	AAGGGCCCGAGATAGTAGGG
9978	ISX-AS1	TGTTCACAGATCCTAGCACC
9979	ISX-AS1	TCAACACAGAAACATAGGCA
9980	ISX-AS1	TAGAGACACGTTGTTAGGGA
9981	ISX-AS1	GGTTAGCTTCACTTTACCAG
9982	ISX-AS1	CCAGGCTGAGGTCATGACTG
9983	ISX-AS1	AGGTCCCAACCTTAAACCCG
9984	ITGA9-AS1	TTAGACTCTAGTATCAAACA
9985	ITGA9-AS1	TGAGCCAGACAGCTTATGGG
9986	ITGA9-AS1	GCGAAACCGTGGATAAGCAG
9987	ITGA9-AS1	GCCCTCCACTCACCCTGTGT
9988	ITGA9-AS1	CCACCAGGCGAGCAGGCGAG
9989	ITGA9-AS1	ATTAATTTACAAACTGACAG
9990	ITIH4-AS1	GGAGCATCCAGAATAACGTG
9991	ITIH4-AS1	CTCCAGGACGTGCTGCACAG
9992	ITIH4-AS1	CACCGATGGCGACCCCACTG
9993	ITIH4-AS1	AGGAACACGCACTCTCAAGG

9994	ITIH4-AS1	AACCTGACAACCATCAAAAG
9995	ITIH4-AS1	AAAGGGAAGTATAATAACAA
9996	ITPR1-DT	TCACTATTTACCAGTTACAC
9997	ITPR1-DT	GGTCAAGGATTAAAGGCGCG
9998	ITPR1-DT	GGATTATCCATACATACAGG
9999	ITPR1-DT	GAGACCACAAATTTCACTGA
10000	ITPR1-DT	ATAGTGATTACATGTTGAAG
10001	ITPR1-DT	AGCCGTTCAAAGTCACCCCT
10002	JADE1	TGCGGGTCACCATGTAAGTG
10003	JADE1	GATGTCCACATAGCCCAACT
10004	JADE1	CTTCATCATATTCGATCCCC
10005	JADE1	CTCATCCGGATTCAGCTGGT
10006	JADE1	CCACCCGTAGCGGAACCAAG
10007	JADE1	CAGCTCACATAGGAAACCCG
10008	KDF1	GTCTACTGAGGACTCCACTG
10009	KDF1	GCGGCCAACAGAGCTATGTC
10010	KDF1	CCCTCGGGTAGGGATACACA
10011	KDF1	ATGGAAAGAATAGTACTCCT
10012	KDF1	ATCAATTTCTCGGCTCGACA
10013	KDF1	AGGTAATGCTCTCTGGCCCA
10014	KHDC4	GCTGGAGACAACTGAGCGAT
10015	KHDC4	CTGCAGTATCAACTCGAGGG
10016	KHDC4	CCTAACTAGCAATAAAAGCA
10017	KHDC4	CAGGTGCCAAAGTCTTCCTG
10018	KHDC4	AGGGACACTACTTATAGCAG
10019	KHDC4	ACTATGCTTGGTACTCCGTA
10020	KIF1BP	GAATGCCCAACTCTCCATGC
10021	KIF1BP	CGCTCAGTAGGATCAAGAGG
10022	KIF1BP	CCGTCGCCCAGCGAGCGGTG
10023	KIF1BP	CAAGTCCAAATACAGCGCCC
10024	KIF1BP	ATTGATGTAAAACTGTGACA
10025	KIF1BP	AGAATAACCTGGGTATCTTG
10026	KIF9-AS1	TGTAGAAAACTGTTACACGC
10027	KIF9-AS1	GTCAGTCGCCAGCTGTGACA
10028	KIF9-AS1	GAGCCATACTCGTTGCAAGG
10029	KIF9-AS1	CGCGTTACCTCAGAGCCCAG
10030	KIF9-AS1	CAAGCACTGTCACTTCAGCA
10031	KIF9-AS1	ACAGGGTTGTGATTTAGCGA
10032	KRT17P5	TGGCTGTGCCATGACCCTCG
10033	KRT17P5	TCCTTCTGGACGTGAAGACG
10034	KRT17P5	GGGCTCCTAGAAGACCAGAG
10035	KRT17P5	GAGGAATTCAACCTGCTCTG
10036	KRT17P5	CCAGGGCTGGTTCTTCAGCG
10037	KRT17P5	AGAGGGGCTGAACTGCAAGG
10038	KRT87P	GTTCGCGGCCTTCATGGACA
10039	KRT87P	GTCCATCTTGACGACCACTG
10040	KRT87P	GGCTGCCGAAGCCTCCGGTG
10041	KRT87P	GAGTGCATGGAAGCCAACAG
10042	KRT87P	CCTGCGCAAATCAGACCTAG

10043	KRT87P	ACACGGTGGCGATGCATGGG
10044	KRT8P36	TGCCTTCATCGAGAAAGATG
10045	KRT8P36	TCTGCATCTCAGACTCCAGC
10046	KRT8P36	GGTCACCCTGATGAACATGG
10047	KRT8P36	CCAGATCTCGGACACGCCTG
10048	KRT8P36	CAGTAAACTCCCCCGCAGGT
10049	KRT8P36	AGAAGATAGAGACTTGCGAT
10050	LAMC1-AS1	TTTCTGGCAGCAGTGAACGA
10051	LAMC1-AS1	TTGCCCTAAGTAGAACACTG
10052	LAMC1-AS1	TGAAGATATATTGCCAGCTA
10053	LAMC1-AS1	TAATGACATACACGAACTTG
10054	LAMC1-AS1	GATGAAAATGACCCACACTG
10055	LAMC1-AS1	CCGTGATTGCTACACCCAAC
10056	LRRC69	TTGCTAGCCAGAAACAACAT
10057	LRRC69	TAATATTTCCAGCTATGTCT
10058	LRRC69	GTTTCAGGATCTGAAGCTGA
10059	LRRC69	GATTGTTGTTCAGATTAAGC
10060	LRRC69	AGAGTTATGCAACTTGACCC
10061	LRRC69	ACAAAGTTCTCTAGGAATGC
10062	LRRC74B	TGGGCTTGGAGAACTGGTCA
10063	LRRC74B	GGCCTGTGGGCGTCTTTCAG
10064	LRRC74B	GAGCTGAACCTCCGGCACCG
10065	LRRC74B	CAATCCATATGTCAAGCGGC
10066	LRRC74B	ATTCCAGCTCACGTTAAGCT
10067	LRRC74B	AAAGCAGCAGCATCCATGGT
10068	LUARIS	TTGTATCATGACCCTCCATG
10069	LUARIS	TGAAGAAAATCTCATCACCG
10070	LUARIS	GCACTGTCAAGCCCTCATCA
10071	LUARIS	GAGTGAGTGAGTACACATCG
10072	LUARIS	GAGCTTAACGAGCCACTCAG
10073	LUARIS	ACGCTAAGATCAGTAGACAT
10074	MACROH2A1	TCCTTGTCTAGGAAACACGC
10075	MACROH2A1	GCAGCGAGAGACAACAAGAA
10076	MACROH2A1	GAGTTGCTAGCGAAGAAGCG
10077	MACROH2A1	CAGGAGTCATCTTTCCCGTG
10078	MACROH2A1	AGGACGGCGGCCATGTACAC
10079	MACROH2A1	AGCGCCGACAGCACAACCGA
10080	MACROH2A2	TGCCAGCAAGATGTGTCTCG
10081	MACROH2A2	TATTGGCTCCATGAGAGTGG
10082	MACROH2A2	GTTCAAGTACCGGATCAGCG
10083	MACROH2A2	GCCAAAAAGCGAGGGACCAA
10084	MACROH2A2	CCACGGACGTCCAAAAAGGT
10085	MACROH2A2	AAAGGAGTGACCATCGCCAG
10086	MAFA-AS1	TTCCACGGTTGATTTCCTAG
10087	MAFA-AS1	GTTCTGTGAAGCAGTCACCA
10088	MAFA-AS1	CGAGAGAAACAAGCTTGGAG
10089	MAFA-AS1	CCTCCTGACCACCAGAACAC
10090	MAFA-AS1	CCAGCACAGGAATCCGACGG
10091	MAFA-AS1	ATATTGATGGAGCACCTCCT

10092	MAFG-DT	TGCACAAGTACCATGGACAG
10093	MAFG-DT	TCCACGACCGGCTTTAAGTG
10094	MAFG-DT	TCCAACCAAGAAACTGCGGC
10095	MAFG-DT	GTGCCAGCCCTCACTGACCA
10096	MAFG-DT	GCCCCGCGATTCCAACTCGG
10097	MAFG-DT	ACTCGGGAGGAAGATAAACG
10098	MAP3K15	TGATCGGCCAAATCACACGT
10099	MAP3K15	GGCAGATGGGAATTACCATG
10100	MAP3K15	GCTGAGGGTTTACCACTCAA
10101	MAP3K15	GCACCTGAGATAATTGACCA
10102	MAP3K15	ATGGTGAGAGAGTTGTCTTG
10103	MAP3K15	AGTCCGAGAAAGCTTTGACA
10104	MAP3K20	TGTATGGTTATGGAACCGAG
10105	MAP3K20	TGCATGGACGGAAGACGATG
10106	MAP3K20	TCCTACACAACAAGGCGGAG
10107	MAP3K20	GTGACAATGCCATAGTTGGG
10108	MAP3K20	GCTTGTAGTGGAAAAAAACG
10109	MAP3K20	CAACACTAACCTACCTGATG
10110	MAPT-IT1	TGACACCCCAGAATCAACCG
10111	MAPT-IT1	TATTAATAAAAATACACCCA
10112	MAPT-IT1	GTGCGTACCAGGTTCATCAC
10113	MAPT-IT1	GGAGCGAAATCGCAGAAGCG
10114	MAPT-IT1	GGACGCTGGCTACCCATCCG
10115	MAPT-IT1	ACAAATAAAAACAGCCGTAG
10116	MATN1-AS1	TCTGCAGCTCTGCTAATGAG
10117	MATN1-AS1	TCCTTGTCCCGGTAAGACCG
10118	MATN1-AS1	CACTGAACCACACTGCCCAG
10119	MATN1-AS1	CACAAATGCAACATCAGCCG
10120	MATN1-AS1	ACCACCTGCGACACGCGGGG
10121	MATN1-AS1	AACCACCCAGGGTCACACAA
10122	MGAT4EP	TATGCACTTCTATGTTCACA
10123	MGAT4EP	GTGTAGTTTCCCAGTCACGG
10124	MGAT4EP	GGTCACCCTTGAATGAATGT
10125	MGAT4EP	GGCCAGTTATAACAGCCGGA
10126	MGAT4EP	CTCCCAGGGGAAATGCACAT
10127	MGAT4EP	AGTCTATATGAGTGACATAG
10128	MIGA1	TTCAGCCTGTCTATTGCGAA
10129	MIGA1	GTTTGTAACTATGCTAATGG
10130	MIGA1	GGAGTTTGAAGCTACCCTTG
10131	MIGA1	CATCACTTTAAAAGAAAACG
10132	MIGA1	AGGATATTCCTCGCTGAGAG
10133	MIGA1	ACTGAAATGTTGGAGTGCCT
10134	MINDY2	TGTGATACACAGATTGTCCC
10135	MINDY2	TAGCTGCGAGTTCAATAGTG
10136	MINDY2	GAGGGTCTACTAACCACCCA
10137	MINDY2	CAAGCAGAACTGACCGCCGC
10138	MINDY2	AGGCCAGGAGCAAAACATTG
10139	MINDY2	ACCTTGTACTGCCCTCTCAG
10140	MIR1281	TCCTCCCGGTGCCCCCTCCC

10141	MIR1281	GGGCGAAAAGGGGGGAGAGG
10142	MIR1281	GGAGGCGGGGGCGAAAAGGG
10143	MIR1281	GCCCGGGAGGGGGCACCGGG
10144	MIR1281	CGGGGGCGAAAAGGGGGGAG
10145	MIR1281	AAGAGACACTCACCTCCTCC
10146	MIR6778	TCACCTGTGGAATGTCAGGG
10147	MIR6778	GTGGGTGGAGCCAGTTCAAG
10148	MIR6778	GTGGAGCCAGTTCAAGTGGG
10149	MIR6778	CTGTGGAATGTCAGGGAGGC
10150	MIR6778	CAAGTGGGAGGACAGGAGGC
10151	MIR6778	AGTTCAAGTGGGAGGACAGG
10152	MIRLET7BHG	TTGGTGCCGATGGGACTCCG
10153	MIRLET7BHG	GAGACATCAGGAGCCCATCG
10154	MIRLET7BHG	CTGGGACAGAGTGTAGCATG
10155	MIRLET7BHG	CCTGTGACCTCCAAACGTCT
10156	MIRLET7BHG	AGTGACACGACCCCACGGGA
10157	MIRLET7BHG	ACGTTATAGAGCCCGACCTG
10158	MKRN9P	TGAGAGCGACTTGTTTCATG
10159	MKRN9P	TAGCAAAGGGGAACTAGACG
10160	MKRN9P	GGAGATTCGTGTGACATGTG
10161	MKRN9P	GACTATCCATGAGAGACTTG
10162	MKRN9P	CGTTCCAAGTGAGTACTGTG
10163	MKRN9P	AGTCACCATTCTGTCCCCAG
10164	MSNP1	TGAGGGAGGTTCTAAGCTAG
10165	MSNP1	TAAATAACTATAAACGGGAT
10166	MSNP1	GTTAGAAGTTTGTCAAACAG
10167	MSNP1	GAGACCTAAAACTCCTGGCA
10168	MSNP1	ATGCTGTCCAGTCTAAGTAT
10169	MSNP1	AAGGGAGGAGCGGTCCCTAG
10170	MT1DP	GTTATTTACAACTGGACGCA
10171	MT1DP	GGTGATGAAGGCTCTTGGCG
10172	MT1DP	GCACATTTGGCACAGCCCAT
10173	MT1DP	GACTGAGGCTGAAAACTGCC
10174	MT1DP	CCTCTCGCCTCAGAAATCGG
10175	MT1DP	CCACAGAATAGAACATCCCT
10176	MT1HL1	GGAGGCTCCTACGCCTGCGC
10177	MT1HL1	GCAGGCGTAGGAGCCTCCAG
10178	MT1HL1	GCAGGAGCCGGCGCAGGCGT
10179	MT1HL1	GCACACTTGGCACAGCCCAG
10180	MT1HL1	CAGCGGCGCAGGAGCAGTTG
10181	MT1HL1	AACTGCTCCTGCGCCGCTGG
10182	MT1XP1	TTGGGGTCCATTTAGAGGCA
10183	MT1XP1	TCTATTTACATCTGAGAGCA
10184	MT1XP1	GAAAAAAAGATGTAGCAAAT
10185	MT1XP1	CTGCATCTGCAAAGGGACGT
10186	MT1XP1	ATAGAGCAACCTTATAAACC
10187	MT1XP1	AGCAGGAGTTCCTGATCAAG
10188	MYCBP2	TGGTCCTTAGCTAGAGACAG
10189	MYCBP2	GGTTGACTGAGATATCCTTG

10190	MYCBP2	GCTTCAGCCAGATTCATATG
10191	MYCBP2	AGTGAAAGGAACGTACACAA
10192	MYCBP2	AGAGCTGTGGTGTTACAATG
10193	MYCBP2	AATGTGCTAATAGACACACC
10194	MYOSLID	GGGCACATCCTAGAGCAAGG
10195	MYOSLID	CTGGGGAACAATACCTGTTG
10196	MYOSLID	CATTTGCCTGCATCTGAATG
10197	MYOSLID	CAAGTGATTAGTCCATAGTG
10198	MYOSLID	AGAATAGACACAGAGATGCA
10199	MYOSLID	ACAAACGACATAAAAGACAA
10200	NANOGNBP3	GTGGGGTAGAACTAAATGAG
10201	NANOGNBP3	GTGGGAAGAAGATCCCCCGA
10202	NANOGNBP3	GAAAAAACATAAGAGATGTG
10203	NANOGNBP3	ATTCTCTGAAAGGATAAACA
10204	NANOGNBP3	ATAAACTCTCTTGTATTGTG
10205	NANOGNBP3	AACGTCTTAGCCAGGCACAA
10206	NAP1L6P	GGGGAGTATGAAGGAATCGA
10207	NAP1L6P	GCTGATTAAGAAATATGACG
10208	NAP1L6P	CAACGACTACTTCACAAATG
10209	NAP1L6P	ACTGTATGAGCCCACGAAAG
10210	NAP1L6P	ACTCGGTACTTAACCACCAG
10211	NAP1L6P	AAAGCCAGTTGTTAACCTGG
10212	NDUFAF4P2	TTTACCTGCACGGAAGACAC
10213	NDUFAF4P2	GTAGAGGGGTAACCGGGAGT
10214	NDUFAF4P2	GAGGAATTCAGATTGCCCAA
10215	NDUFAF4P2	CTTGCGCGGCGTGAATCTGA
10216	NDUFAF4P2	CAAAAGTAACAAAATAAGAG
10217	NDUFAF4P2	AGAATTCTCAGTTTCGCCGG
10218	NDUFAF8	GCTCATGTCGGCTAACGGAG
10219	NDUFAF8	GCGCGCAGAAGTCCTTACTC
10220	NDUFAF8	CTTACTCAGGCGGCCGCCCG
10221	NDUFAF8	CGCGGAGGCGGCTTCGCACG
10222	NDUFAF8	CCTCACCTCGGCCCCGCAGG
10223	NDUFAF8	CACGCACCTGCCGTACGCCG
10224	NFE2L3P1	TTCAAAAAGAGCAAATACTG
10225	NFE2L3P1	TGTTAACTATTGGGGTAGCG
10226	NFE2L3P1	CTTTAGCACTCCATTCATCA
10227	NFE2L3P1	CATGATGGGAACTAGAGTCA
10228	NFE2L3P1	AGAAATGGTTCTTGTGACTG
10229	NFE2L3P1	AAATGAGAGACATCTGAATG
10230	NFE4	TGTAGAGTCTTAAATGACCT
10231	NFE4	GCTCTGCATCTGCTATACAA
10232	NFE4	AGAAGACACCTACTCTGACT
10233	NFE4	ACAGTCAGATCAGAATCAGT
10234	NFE4	ACAAACACCATGATACAAGA
10235	NFE4	AAGTGGGAGTACTGTCACTG
10236	NOS2P2	TGCCTAGCTGAACTTGAATG
10237	NOS2P2	GGTGTGACGGCACTTCCTGG
10238	NOS2P2	GCTGCGGCACTCGAACACCG

10239	NOS2P2	GCCAGCACAGAACTTAACGA
10240	NOS2P2	GAGGTGAGAAATAAGAAGTG
10241	NOS2P2	CAGCCCTGCAAACATAGAGG
10242	NPIPB3	GTCTTGAGATCATCCGCTGA
10243	NPIPB3	GGGGCCGCTTCCACCCTGAG
10244	NPIPB3	GGAGTGAGTAGACACTCGGG
10245	NPIPB3	GAAGGGCATGATATGACGCT
10246	NPIPB3	AGGGGAGTGAGTAGACACTC
10247	NPIPB3	AGATTATCATCCGCTCAGGG
10248	NPIPB9	TGGTCATGCGACAGGAACTG
10249	NPIPB9	TGACCAGAGCCAGTTCACCA
10250	NPIPB9	GTCATGCGACAGGAACTGTG
10251	NPIPB9	GCTGTGAGGTAGAGCCAGTA
10252	NPIPB9	GACACTCAGGAGGTGTCTTG
10253	NPIPB9	AGCTGTGAGGTAGAGCCAGT
10254	NPY6R	TGGTATCCCCAAATATCCAG
10255	NPY6R	TCAAATACAGACACAGATCA
10256	NPY6R	GAGTTGGAATCATTGCACAG
10257	NPY6R	CCCACAGAGTTGCTTACACA
10258	NPY6R	CATCTTTGACTGGTATCATG
10259	NPY6R	AATCAGTGTGATGCCCCAGT
10260	NT5DC4	TGAGTATGTAGAAACACTGC
10261	NT5DC4	GTGTAGCGCAGGATCTCATG
10262	NT5DC4	GATAGCCGGTGTCACAACTG
10263	NT5DC4	GACATGGACTACACTCTGGC
10264	NT5DC4	CCTTTGTCCCTTACTCCGAG
10265	NT5DC4	CATGAATAACATCCACCAGT
10266	NTF6G	GTAGGATGGGTGCAACAACG
10267	NTF6G	GGAAAAGGGGACAATGTCAA
10268	NTF6G	GCCGATAACTCTGAAGAAGG
10269	NTF6G	GAGCACAACCAAGTCCACAG
10270	NTF6G	CTGGGGAGGAAGCAAATGAG
10271	NTF6G	CCCTAGACAGGACCACTTGG
10272	NUTF2P7	TGGAGCATCACCCAGCCTCG
10273	NUTF2P7	TGCATCATCAGCATGGCTGT
10274	NUTF2P7	TCTGGTGGAAACCCATGATG
10275	NUTF2P7	GCTAGACAAGTTCTCCACAA
10276	NUTF2P7	CTAGACTAGTTGCTTCTGAC
10277	NUTF2P7	AGGTCAACCGAAGTTGTGCA
10278	NXF4	GATGCACAAGTCTACCACAT
10279	NXF4	GACCGAAGGCAAGATCTCAG
10280	NXF4	CTGTGTCCCTAACGCCTGAG
10281	NXF4	CAGCCAAGGAATCCGAAAGG
10282	NXF4	ATGGTACTGAGAACATCCCG
10283	NXF4	AGAGACCTGACACATGTTAG
10284	OCIAD1-AS1	GTCAAGGACTCTGCTAGACG
10285	OCIAD1-AS1	GGAAGAAGATGAATAACACT
10286	OCIAD1-AS1	GCAGAGTCCTTGACACCTAC
10287	OCIAD1-AS1	GAGTTTAGGTTGATGATTGA

10288	OCIAD1-AS1	CAGGTGATATCCAGCAGAAA
10289	OCIAD1-AS1	AATAAAGTTTATACATACGT
10290	OLA1P3	TGCAAATTATATCATGTTTG
10291	OLA1P3	TCTACGTATGTACTTCATCT
10292	OLA1P3	CAAAGTAATGACCAATACAC
10293	OLA1P3	ATCTGTATGAATCTGTTCCA
10294	OLA1P3	AGATTAATCAAGTACACCAC
10295	OLA1P3	ACAAAGCAGAAATTATACTG
10296	OR6W1P	GTAATCCATTCCTCGCCCCA
10297	OR6W1P	GAGAGGGTGAGCCCTTGACA
10298	OR6W1P	GAGAACTAATGGACACACAT
10299	OR6W1P	CTCCACATACCTCAACAAGG
10300	OR6W1P	CGCTACCCAACCATCACGAA
10301	OR6W1P	CATCCTATGTACCACAGTGG
10302	OSTN-AS1	TTCACCACTAACAGTAGAGG
10303	OSTN-AS1	GAAGCTCACATAAGAAAGGT
10304	OSTN-AS1	CCATGATTGTAAGTTTAACG
10305	OSTN-AS1	CATTTAAATAACTTATGGGC
10306	OSTN-AS1	CACAACCTTGAATGGCATGT
10307	OSTN-AS1	AAGAAGAAAAGGAGTTTCTC
10308	PAK5	TTGGAATGAATAATCCAGAG
10309	PAK5	GGCCAACTTTATCAAAATCG
10310	PAK5	GGATGGGTGTGATGCATGAA
10311	PAK5	GGAGGGGTGATGGTACAAGG
10312	PAK5	GCATTTAAAACCCATCCCCA
10313	PAK5	CGTGTAGTCAGCAGTAGTAT
10314	PAXX	TGGGCCTGGCAAAACGCGTG
10315	PAXX	GCAGGTGCTCCAAAGCTCCG
10316	PAXX	CGGCTGAGGACATCACCCCC
10317	PAXX	CAGTAGCACACGAAGCGGGG
10318	PAXX	AGGTCAAAGGCCAGTGCCGA
10319	PAXX	ACTAGAGGTTGAAGCCGCCG
10320	PCNPP3	TCTGATAACCAGAAACTATG
10321	PCNPP3	GCCTCCAAATACAATACTGT
10322	PCNPP3	GAGAGGAGTATTGTATTGGG
10323	PCNPP3	CAGCGAGCTGGAGCTACCGG
10324	PCNPP3	CAAATCTAACAAGGTATATG
10325	PCNPP3	ACTTGACACAAATCATAAGG
10326	PDXDC2P-NPIPB14P	TTTATCAAGAAGGGAAACAG
10327	PDXDC2P-NPIPB14P	GGAATAAGAAGACATTCGGG
10328	PDXDC2P-NPIPB14P	CTGTACTTTATGGAAACAGG
10329	PDXDC2P-NPIPB14P	CCAGCGTGGAAAGAGCTGCG
10330	PDXDC2P-NPIPB14P	ACCTTGAGAGAAACTGAATG
10331	PDXDC2P-NPIPB14P	ACAATAATGATAATTATCCA
10332	PGBD5	GCTGAGGACGGACTCGCAGT
10333	PGBD5	GAACCCCTTCTACAGCACCT
10334	PGBD5	CCTCAAGTACTTCCACGTCG
10335	PGBD5	CCGCGCTTCGAGGATACCGG
10336	PGBD5	ACAGGATCCTCATCGATCAG

10337	PGBD5	AAGAACATTGTCTGACCCAG
10338	PIEZO2	GTTTCCATAAACCACCATGA
10339	PIEZO2	CTTGCATAGTAGTCATTGGG
10340	PIEZO2	CTCCTATCGATCCTACAGAG
10341	PIEZO2	AGTGGCAACCACTTACTGAC
10342	PIEZO2	AAGTACTGGATCTACGTCTG
10343	PIEZO2	AAGATGAAGAACCTCCACGA
10344	PIMREG	TTGAGGTTGACGGCTCTCAG
10345	PIMREG	TGTCAGTATTACCTGGGACA
10346	PIMREG	CTGGAGAGATCTCCGGCGCA
10347	PIMREG	AGCCATCAGGAGACCTCTGT
10348	PIMREG	ACCCCAGAGCCAGGTCAGCA
10349	PIMREG	AAGGCCAGGAGGCGGAAGAG
10350	PKD1P1	GGAAGGAGCACCCAGAAGCA
10351	PKD1P1	GCGCGTGACGATGGGCGTGA
10352	PKD1P1	CCAGGGGCAGCTGGGCACGA
10353	PKD1P1	CATGGCTTGGGGGCATGAAG
10354	PKD1P1	ATGCGAGTGAAACAGCTCCG
10355	PKD1P1	AGGCAGCAAGAGCCCCCCAG
10356	PKD1P6-NPIPP1	GGGACATCCAGAAGAGAAAG
10357	PKD1P6-NPIPP1	CTGGTGGCTGAGCTGCAGTG
10358	PKD1P6-NPIPP1	CACGTCACTGAGGTTAGCCG
10359	PKD1P6-NPIPP1	CAACGGAGTTGGCAGAGCTG
10360	PKD1P6-NPIPP1	ATGGAGACAGGTGGAAACCA
10361	PKD1P6-NPIPP1	ATCCGAGTGTGGCACGACAA
10362	PLPBP	TTGCTGACCGCCACTAGCCG
10363	PLPBP	TTCAGAGACCATAGCCATCG
10364	PLPBP	TGGGAGTCGGGTGCGCATTG
10365	PLPBP	TGATCGAGGCCTATGGACAT
10366	PLPBP	GGACACAAAGACAGAATCTG
10367	PLPBP	ATGGTCCAGATTAACACCAG
10368	PLPP3	GTCCCGGAGAGCAAGAACGG
10369	PLPP3	GATGGCAATGACGATCCCCA
10370	PLPP3	GACGATTCAGAACCCCTACG
10371	PLPP3	CAGGCGCCCTATGGACACTT
10372	PLPP3	ACCATCAAGCCTTACCACCG
10373	PLPP3	ACAGCAAAGTCCAGGAAGCC
10374	POLR1H	TTTCTGTTCAGATTGCGGCT
10375	POLR1H	GGCTTCAACATCAACGTTCG
10376	POLR1H	AGGGCAGCGCCTGTCAACCT
10377	POLR1H	AGCTGGAGCAAGTATTGGCG
10378	POLR1H	AGCCATGCCTATGTCGGTGG
10379	POLR1H	ACGGTCACCTGTATTCGCTG
10380	PRAG1	TGGAGTTTGAATGGTCCGTG
10381	PRAG1	TGCTCAGGTAGATCGTCCGA
10382	PRAG1	GCAATGTTCCTCTCGCCGCG
10383	PRAG1	GATTTGGAAAAAGTGAGCCA
10384	PRAG1	CAGCCCAAGGCATCTGCTAG
10385	PRAG1	AGCTAAAGTGAGATTCGTCG

10386	PRMT5-AS1	TGTGGGGGTACTGATTTGAA
10387	PRMT5-AS1	GACTTCAAACTGTAACCCTG
10388	PRMT5-AS1	CCAAGCACCGAGGTCAGGAG
10389	PRMT5-AS1	ATTTGCTGAAGGAGTCACGT
10390	PRMT5-AS1	ATGTAGCTGGTTATAACATG
10391	PRMT5-AS1	AACATGCCTGGGTCTAAGTG
10392	PROX1-AS1	TTTGGGCCTCAGATTTCCAA
10393	PROX1-AS1	TTAGATAAGACACCTTCACT
10394	PROX1-AS1	GTGGTCTCAATCCATGCCTG
10395	PROX1-AS1	GCAACTGGTGGATATGCCGG
10396	PROX1-AS1	ATGGTTAGGAGACTGTTCTG
10397	PROX1-AS1	AGAAACTGTCAGACCAACAG
10398	PRXL2C	GTCATCCTACCATCATATTG
10399	PRXL2C	GACATGTTATGTCTTACCTG
10400	PRXL2C	CGCGGGCAGCGGGTACCGTT
10401	PRXL2C	CATCTGCAAGGAATACGTAG
10402	PRXL2C	CACCCGCACGAACACCACCA
10403	PRXL2C	AGGGTGACATTTGCTTCCTT
10404	PSME2P6	TAAATGATCTATATGTTGTG
10405	PSME2P6	GAGAAAACTGTCCACCCAAA
10406	PSME2P6	GAATGCCATCAAGACCAAAG
10407	PSME2P6	CTCGCTCATGCACCAAGACC
10408	PSME2P6	CAAGTATTTCTCAGAACGTG
10409	PSME2P6	AGCAATCCAGGATAAGGTGT
10410	PTBP1P	TCTTCCTCGGATACTGAGGG
10411	PTBP1P	TCCCATCGACGTCATCGAAG
10412	PTBP1P	GCTGATCCAAATGCGCTCCG
10413	PTBP1P	GATACGGATGAGATGCGGAT
10414	PTBP1P	CATGGTGAATTACTACACCT
10415	PTBP1P	CACACACAGCAGACCCGTGG
10416	PTPRVP	TGGAGCTGAAGGTCCTAGCG
10417	PTPRVP	TCACTCGTCACATTTACCAG
10418	PTPRVP	GGTCTGCATTAGGCGAGGAG
10419	PTPRVP	CTCTAGTAATAAGCAGACGA
10420	PTPRVP	CAGTCCATGGACAGTTGACG
10421	PTPRVP	AAGCAGTACCACGCGCTGAG
10422	RASAL2-AS1	TGTATACCTATGTAACATGG
10423	RASAL2-AS1	TCGTTACTCGAGTGCACACG
10424	RASAL2-AS1	TAAAAAAGCAGGATTACTGG
10425	RASAL2-AS1	GGGATTCTGGGATATGCACA
10426	RASAL2-AS1	GATCTACTATGTCCAAGGCA
10427	RASAL2-AS1	CCAGTCTCTTTGTGCAACGT
10428	RBAKDN	TTTGGGTGAGGCGTTAACAG
10429	RBAKDN	GTCCGGATCTAACAATCCCT
10430	RBAKDN	GGTTCCGTGAGAGGCGTCCA
10431	RBAKDN	GCCACGCGACCAGAAAGCCG
10432	RBAKDN	CGTCCCCTGCCAATGCGTTG
10433	RBAKDN	ATCAATACAGCCAGAGCCCG
10434	REC114	TTGAGCCTCGGGCTTACCGG

10435	REC114	GTTGAAGATTGTAAGACGCG
10436	REC114	GAAATGACCTGATATAACTA
10437	REC114	CCCAGGGCAACTGAAAGTCA
10438	REC114	CAAGGACAGACACTACTGGT
10439	REC114	CAAACACCAGAGACCCACCT
10440	RETREG2	GCCAGGTATCTGCACAACTC
10441	RETREG2	GAGGCTTCTATCTTGGAGAG
10442	RETREG2	CTTGTCGTGTTTGTGATGCA
10443	RETREG2	CAGCTCATGATAAACCACCA
10444	RETREG2	AGAGACAGAGAGTGAAAGCG
10445	RETREG2	AACACTCACCTGAAACGTCA
10446	RPL22P19	TCAAAATGAACGGAAAAGCT
10447	RPL22P19	GGGCAGCTTGGATGTCACGC
10448	RPL22P19	GGCGCCCATGAAAAAGCTTG
10449	RPL22P19	GAGGGGTAGTGACCATCGAA
10450	RPL22P19	CCAAATTAACCAGGACGAAG
10451	RPL22P19	AGGGCTAAAAAAAAGGAAGC
10452	RPL22P3	TGGTCGCCATCCCTCCACCA
10453	RPL22P3	TGCACTCACCCCGTAGAAGA
10454	RPL22P3	TGAAGAATAATTTACGTGAT
10455	RPL22P3	GGCTTCTGTGAAAAAGCCTG
10456	RPL22P3	CCAGATTAACCAGGACAAAG
10457	RPL22P3	CAAGATCAACGTGACATCCG
10458	RPL23P2	TTTATTCCGAAGATAATGCA
10459	RPL23P2	TTAGACAACCAAAGTCATAC
10460	RPL23P2	TGATCAACTGCTGACAACAC
10461	RPL23P2	GCGTTCAAGATGTCAAAGCG
10462	RPL23P2	CGAGCATTGGACACAATCCA
10463	RPL23P2	AAAAATGGTACATCCAGCAG
10464	RSRP1	TTCGCTTAAGTGAAAAAGGT
10465	RSRP1	GGTTCACCAGGAGATACTAC
10466	RSRP1	GGAAGTACAGGCGCTACTCG
10467	RSRP1	AGGGCGTACGCGATCGCGCG
10468	RSRP1	AGGATTCGCCCTCGACCTCG
10469	RSRP1	ACCAATGCAGCGAAAGCTCT
10470	RTL4	TGGTACAGGCATCACTGTGG
10471	RTL4	TCAAATGCAGCATCCAACCA
10472	RTL4	TCAAACTTAGCATTCATCAG
10473	RTL4	GGCACTAGCTGATCCCAACC
10474	RTL4	GGAACTCTGAGCAATTGGCA
10475	RTL4	AAAGTTGTGGGATCATATCT
10476	SACS-AS1	TGCCACAATCGACTGCCCGT
10477	SACS-AS1	GGTGACAAAGTGAATTGCAC
10478	SACS-AS1	GCAGCACACTATGCCAACAC
10479	SACS-AS1	GAGGAGATAGAGTATTCGTG
10480	SACS-AS1	GAGCCAGCTAGTGGCACCCA
10481	SACS-AS1	AAGGCCAAGAGGTCACTGCG
10482	SCARNA8	GTAAAGTCTTCATAATTGGG
10483	SCARNA8	GGGAGGCTGATACACAAATT

10484	SCARNA8	CGATTTCACTCAATTCGTCC
10485	SCARNA8	CGAATTGAGTGAAATCGTAA
10486	SCARNA8	AATCGTAACGGACAGATACG
10487	SCARNA8	AAAGGCAGTTTATTCTAGGG
10488	SCHLAP1	TCTACTCTACTCTAGCCAAG
10489	SCHLAP1	TAGCAGATTTCAGCTCACAC
10490	SCHLAP1	GGTCACAAAGTCCTTCATAG
10491	SCHLAP1	GCACTGGGACCAGTTAGCAG
10492	SCHLAP1	CAGCTCTTAAGGCGGCGCGT
10493	SCHLAP1	ATGATGAGAATGGAATGACT
10494	SELENOKP3	TGTTATCAAAGATAAACTCC
10495	SELENOKP3	TGGGGAACAGCACTGCATGA
10496	SELENOKP3	TCTGATTCCAGATATGATCA
10497	SELENOKP3	CTGCAAGTTGCTCCGAAGTG
10498	SELENOKP3	ATCTAAACAAGGATTGCTCT
10499	SELENOKP3	ACACAAGTTCGAATCATCTT
10500	SEMA5A-AS1	TTATTTACATCCTATCCCAA
10501	SEMA5A-AS1	GAGAAGAAAGTAAACACTCA
10502	SEMA5A-AS1	CAGCGTCTTCTGGGACACAG
10503	SEMA5A-AS1	ACTGTCGTGTCTCTTCACAC
10504	SEMA5A-AS1	AACACAGAAAACTAACATAG
10505	SEMA5A-AS1	AAACATGTCCAGTCCGGAGT
10506	SEPT7P2	GCCGCCGCACATAAACTGCG
10507	SEPT7P2	GCAGGAGACCGCCACTCACG
10508	SEPT7P2	GCAGATAGCCCGTCACAATG
10509	SEPT7P2	CATATAACAACCATCATGAG
10510	SEPT7P2	AGCAGGCTAGAGCCGCAGGG
10511	SEPT7P2	AGCAGACACACTCATGCCAG
10512	SERBP1P5	TTGAACAGAAGGGTTCACGA
10513	SERBP1P5	TCAACCACGCCAACGCTGCG
10514	SERBP1P5	GTGACTGACGAAACACCTGA
10515	SERBP1P5	GAGTGAAGAGGCTTATGCTG
10516	SERBP1P5	CGGCCCGCTGCAATCCGTAG
10517	SERBP1P5	ATATTCACACATGATCAACT
10518	SETSIP	TCTGGGACTGGAGGAGACAT
10519	SETSIP	GCAGTGAAGACACTTGTGGA
10520	SETSIP	CAAAGTCCACCAAAATCAAA
10521	SETSIP	AGTGTCTTCACTGCTTGGGG
10522	SETSIP	ACTTAATGAACAAGACAGTG
10523	SETSIP	AAACGCAGAATAAAGCCAGC
10524	SLC22A20P	TGGTGGACGGGAAAACACTG
10525	SLC22A20P	TGCAGGTAGGACCAGACCAG
10526	SLC22A20P	GGTGCTGTATTCCCCAAGTG
10527	SLC22A20P	GGGCATCCACTCCACAACTG
10528	SLC22A20P	GCGGCCCACGTAAATCATGG
10529	SLC22A20P	GCCTCTCTACATAAAGCCGG
10530	SLC39A12-AS1	TGGAAGCCATCCCCTATCTG
10531	SLC39A12-AS1	TCACCCTCACCTCCAACCGT
10532	SLC39A12-AS1	TAAAGATTGTATCTACAGAG

10533	SLC39A12-AS1	GATGTTTCAAGCCCCGTTCA
10534	SLC39A12-AS1	ATAGTGCGTTACTTTAGCAA
10535	SLC39A12-AS1	AGAGGGCAAGCGTTTCCTAG
10536	SMIM10L2A	GTCTGCAGCGGCGGCTGCGG
10537	SMIM10L2A	GTCGGCGGCTCTGTCTGCAG
10538	SMIM10L2A	GCTGCGGCCCGAGGCTCGTA
10539	SMIM10L2A	GCGGCTGTCGCGCTCAGCTG
10540	SMIM10L2A	CGTACGGCGCCTTCTGCAAG
10541	SMIM10L2A	CCGCACCGCCAGGCCAGACA
10542	SMIM10L2B	GGTTCGGCTGTCGCGCTCGG
10543	SMIM10L2B	GGCGTCGGCGGCTCTGTCTG
10544	SMIM10L2B	GCTGCTCACCTTCTTCGACC
10545	SMIM10L2B	CTCATACGGCGCCTTCTGCA
10546	SMIM10L2B	CCGAACCGCCAAGCCAGACA
10547	SMIM10L2B	AGAAGGCGCCGTATGAGCCT
10548	SMIM24	GTTTCTGCTCCTCTCCCCGG
10549	SMIM24	GGAGGAGACCACGTTCAGAA
10550	SMIM24	CGGAGCATCGCCTGAAGCCG
10551	SMIM24	CCAACCTATACCAGGACCAG
10552	SMIM24	AGACCAAATAGACGATGAAC
10553	SMIM24	ACCTGGCCTTGGAACACCAG
10554	SMTNL1	TGGATTAGATGAGGTCAAAG
10555	SMTNL1	TGAACCTAAGGCAACAGTTG
10556	SMTNL1	GTGGCCTGAGAGCCCCACTG
10557	SMTNL1	GGCCAAGGCTGAATCGCAGA
10558	SMTNL1	CTGGAAAGGCCATCAATGAG
10559	SMTNL1	CATCTCCTGAGAACCCACAG
10560	SNHG1	TAAGGTGTATAAAAAATGGT
10561	SNHG1	TAAGACATTAGGTCAACTCA
10562	SNHG1	GGAGACCTAAAGGCTCATGA
10563	SNHG1	GAAGGACAAAATGGCGATGG
10564	SNHG1	CTTCAAAAAAGGTTCCCTTG
10565	SNHG1	AGGTCAGTAGTTTACGAAAG
10566	SNHG10	TGGACTACGCCGAGACAACT
10567	SNHG10	GTTGGGCCTAGGATCAGGAA
10568	SNHG10	GAGAAATAATCGCGCGCCGG
10569	SNHG10	CTAGGACGATGCTTGGAACG
10570	SNHG10	CGGCCGCATGCAGTTCGTCG
10571	SNHG10	ACTGCCTTACTATTGGTCGT
10572	SNHG12	TTGTCCACAGAGCGTACACA
10573	SNHG12	GTTTCTACCTAAAATGACCG
10574	SNHG12	GCAAAACATTGCGCTATGTG
10575	SNHG12	CATCCCAGAGACAACTCCAG
10576	SNHG12	AGGGGGTCATACTCCCCGAA
10577	SNHG12	AAGACTCTTAAGATGACAGA
10578	SNHG15	TCCGTACTTCGTAGTCGCAG
10579	SNHG15	GGGACTACGCGGTGACGTCG
10580	SNHG15	GGAATGGTCAGGCAACACGT
10581	SNHG15	GCTGTAGAAACACTGACGGA

10582	SNHG15	CTTGAACATGAGACTCAAGG
10583	SNHG15	CTCTGCAACGTGATTTACCG
10584	SNHG28	GACCTTGGGCTAACACACTG
10585	SNHG28	CTGAGGGAAGTCTACACCAG
10586	SNHG28	ATGGTCCTCATATCTCCCAT
10587	SNHG28	AGTTTATGGACAAGGTGTGG
10588	SNHG28	AGGAAAATAAAGTGAAAACG
10589	SNHG28	ACGGTGCCCATCTCAACCAT
10590	SNHG4	CTACCCTTAAGAGATGGGAT
10591	SNHG4	CCGAGGGGAACCCCGCCCCG
10592	SNHG4	AGAGAATCTGTGTGTCCTAG
10593	SNHG4	AATTACTATAGCTCAGATGG
10594	SNHG4	AAAGGCGCCACGCTCGTGAG
10595	SNHG4	AAAAAAAAATGCGAGTGCGG
10596	SNHG8	TGCACGGAACCTTTCCCCGA
10597	SNHG8	TCAGGATATTGGTAGGTCGG
10598	SNHG8	GCTCAAACTGACGGTTCTCG
10599	SNHG8	CTTTCACATTCGGGAAGCGT
10600	SNHG8	CTTAAGTTTACAAGCATGCG
10601	SNHG8	CTGAGCTGAACACATTACGA
10602	SNORA11	TTGGGGGACGTTTGTTCATA
10603	SNORA11	TGATGAGGGCGTCAAACTTG
10604	SNORA11	TAAACAGAGGAGCCATTCTT
10605	SNORA11	GAAGCCACATAGAGGTGTTG
10606	SNORA11	ACACACCCAACAGGAATCTG
10607	SNORA11	AATCTGGGGTCATTGTGATG
10608	SNORA69	CAGATTGACATGGACAATTC
10609	SNORA69	AGATTGACATGGACAATTCT
10610	SNORA69	AAAATCATGACAGATTGACA
10611	SNORA69	AAAATCAAAAATGATTAACA
10612	SNORA70J	TTGCACACTGGGCCCCCATG
10613	SNORA70J	TTATTGTACGGGTTGCTCTA
10614	SNORA70J	TGTTTGCAGCGATTGCACAC
10615	SNORA70J	GAGCAACCCGTACAATAAAC
10616	SNORA70J	CGGGTTGCTCTACGGGACCT
10617	SNORA70J	ACAGGCTGCATATACTACCA
10618	SNORA71C	GGAGGTGTGGGAAAGCTCCA
10619	SNORA71C	CCCTGGTCATTGGTAGTGCA
10620	SNORA71C	CCCTGCACTACCAATGACCA
10621	SNORA71C	CAAACACGGGGAAGTGCTCT
10622	SNORA71C	ATGATCACTTTCGAATGCAG
10623	SNORA71C	AGCACTTCCCCGTGTTTGGA
10624	SNORA73B	TCCAACGTGGATACCCTGGG
10625	SNORA73B	TCATCTTCAACGTTGTGGAA
10626	SNORA73B	GCTCTGTCCAAGTGGCATAG
10627	SNORA73B	CACTAATGAGCATTCCAACG
10628	SNORA73B	AGTCCTGTGGACAATGACTG
10629	SNORA73B	ACAACGTTGAAGATGAAGCT
10630	SNORD116-2	TCCCCAAAAAAAACATTCCT

10631	SNORD116-2	CTCAGTTCCGATGAGAATGA
10632	SNORD116-2	CGTCATTCTCATCGGAACTG
10633	SNORD116-2	ACTCATACCGTCATTCTCAT
10634	SNORD12B	TCAACATAGTCGATCTGTCG
10635	SNORD12B	GATCAACATAGTCGATCTGT
10636	SNORD12B	GACAAAGGAGCTGCTCAAGC
10637	SNORD12B	CTGGCATATCAGACAGAAAC
10638	SNORD12B	ATCAACATAGTCGATCTGTC
10639	SNORD12B	AAGTCATCATATATGCCAGC
10640	SNORD14E	TTGGGTCACTCAGACATCCA
10641	SNORD14E	TGTAGGGTCACAATGATGAA
10642	SNORD14E	TCCACCAGAATTCAAGGTGT
10643	SNORD14E	GTCACTCAGACATCCAAGGA
10644	SNORD14E	GGTGGAAACCGCGAATGTTT
10645	SNORD14E	GCCAACACCTTGAATTCTGG
10646	SNORD3B-1	TGAGCGTGAAGCCGGCTTTC
10647	SNORD3B-1	GTAGAGCACCGAAAACCCCG
10648	SNORD3B-1	CCGAAAACCCCGAGGAAGAG
10649	SNORD3B-1	AGAAAGCCGGCTTCACGCTC
10650	SNORD3B-1	AAACGCTACCTCTCTTCCTC
10651	SNORD3B-1	AAAACGCTACCTCTCTTCCT
10652	SNORD7	TTCACTGTTGGCTTTGACCA
10653	SNORD7	TATTGCCATTGCTTCACTGT
10654	SNORD7	GAGTGAAGTAGAGCCTGACC
10655	SNORD7	CAAAGCCAACAGTGAAGCAA
10656	SNORD7	AGTGAAGCAATGGCAATACC
10657	SNORD7	AAGATTAAGAGATCATACCC
10658	SNORD99	TGGATGAGAAATGCGGATAT
10659	SNORD99	GTATTTAGAATGACTGGTCC
10660	SNORD99	CTAATTTGAGTGGACATCCA
10661	SNORD99	AGGATGAAACCTAATTTGAG
10662	SNORD99	ACTCAAATTAGGTTTCATCC
10663	SNORD99	ACATCCATGGATGAGAAATG
10664	SPATA31E3P	TGGTTTGTACATATCACCCA
10665	SPATA31E3P	TACAAATTCAGAATCCCGGG
10666	SPATA31E3P	GCTCCACAGACCCTAATCTG
10667	SPATA31E3P	GACAAACTGGGGATTCAGCA
10668	SPATA31E3P	AGGAGCGATGCACAGACACA
10669	SPATA31E3P	AGACACTGAGGGGTTACTAG
10670	SSX8P	TTGATAATGACCGTAACCGC
10671	SSX8P	TGGTGACAAAGTGTGGCTGG
10672	SSX8P	GTGGGAAAAGATGAAATACT
10673	SSX8P	GGAAAAGCCAAGCTGATGTG
10674	SSX8P	CCATGATGAGAAGCAGAACA
10675	SSX8P	AAGTCTGAAATTAAACACTC
10676	SUSD6	TGACGTGTAAGAATGGCGAG
10677	SUSD6	GGTTGCACTACCATCATACG
10678	SUSD6	GCAGCACAAACAGCACCACG
10679	SUSD6	GAAGCCACTATAGACAGCGT

10680	SUSD6	AGGGTGCAAAGGATCACTAG
10681	SUSD6	ACAGCACAATCTGTACTCTG
10682	TBC1D27P	TGCCCCTCAGTAGAAGAACG
10683	TBC1D27P	TGCATCATTCAAAATACACA
10684	TBC1D27P	GGGGACTCATCTCAGCAGTG
10685	TBC1D27P	AGATTACAAAGCATTAGGGG
10686	TBC1D27P	ACTCGAGAGAGGGTCCTCGA
10687	TBC1D27P	AAATTGTAGTAAGGAAACCA
10688	TEX49	TACTCTAATTTCAGACCAGA
10689	TEX49	CCCCCATTATCTCAAAAGAT
10690	TEX49	CAGCTTCGTGGTATCTTGTG
10691	TEX49	CACCCATTCAGTTCCCAATC
10692	TEX49	ATACCACGAAGCTGTCCGGA
10693	TEX49	AGTTCTGAGCATCAGTCAAG
10694	TOGARAM1	TTTGCTATCTACAAGAGCTG
10695	TOGARAM1	CTGAGAACGCTTATACAACA
10696	TOGARAM1	CCCAACTATCTGTACCCGAA
10697	TOGARAM1	CAAAAATCGTCTGATCCTAC
10698	TOGARAM1	AATGAAGCTCGACTTGACGA
10699	TOGARAM1	AAGCCAACAAGACTAGAATG
10700	TOGARAM2	GCACCGGGTCTCAACTCGTG
10701	TOGARAM2	GAGGCGTGGCGGGCAAAGCG
10702	TOGARAM2	CTCCCAGAGAGCCTTCATGA
10703	TOGARAM2	CGGAGAAGGGAGGAGCACCA
10704	TOGARAM2	CACATACTAGACACCCGCCG
10705	TOGARAM2	AAGGAGAAGGCTGTCAGCGT
10706	TPSB2	GCTTCAGAGGAAATGGCGGT
10707	TPSB2	GCTGACCGCAGCGCACTGCG
10708	TPSB2	CTACACCGCCCAGATCGGAG
10709	TPSB2	CCTCAGAGACCTTCCCCCCG
10710	TPSB2	CAGCGAGTGGGCATCGTTGG
10711	TPSB2	AGTGCATCCAGTATCGGTCG
10712	TRBV23-1	GGACATTTGGTCAAAGGAAA
10713	TRBV23-1	GCCAAAGTCACACAGACTCC
10714	TRBV23-1	GACACAGGGCTGCACAGCCG
10715	TRBV23-1	ATGGATTGTACCCCCGAAAA
10716	TRBV23-1	ATCTGCTGCCAGGAGACACA
10717	TRBV23-1	AAGAACTTGTTCATTCTGAA
10718	TSPEAR-AS1	TCAGAGCTCAGATCCGACTG
10719	TSPEAR-AS1	CGTGGTTACCGAAAAACACG
10720	TSPEAR-AS1	CCTACAGCGAGTGTGACCCA
10721	TSPEAR-AS1	CCATCCCACACCCTACACCG
10722	TSPEAR-AS1	ATACCTTTAGACCCTTCGAG
10723	TSPEAR-AS1	ACCTGGCCACAAACACACGG
10724	TSPEAR-AS2	GCACGGCTCCCACATGTCAG
10725	TSPEAR-AS2	CTGTGAGTACACGTGACACA
10726	TSPEAR-AS2	CTGATGACACATGACTGGTG
10727	TSPEAR-AS2	CAGACAAGGAGCACACACGG
10728	TSPEAR-AS2	ATTTGAAACGTTATCACACA

10729	TSPEAR-AS2	AAGTTACCTTCAAAGCACAC
10730	TSPY10	GTGGTATCCGGATTATGAAG
10731	TSPY10	GTATCCGGATTATGAAGTGG
10732	TSPY10	GGATGGAGGCTGTACAGGAG
10733	TSPY10	CTGTACAGCCTCCATCCTGA
10734	TSPY10	AGGATGGAGGCTGTACAGGA
10735	TSPY10	AAGCCCCACCTAGACCGCAG
10736	TSPY26P	TATGTAGGCCGCACATCCCA
10737	TSPY26P	GGTCCACAATTAGGGCACCA
10738	TSPY26P	GGCCGAGGACGCGAACAAGG
10739	TSPY26P	GCTCGGAGTAGTCGCCGTGG
10740	TSPY26P	ACTCGCATCCGATGGCACTG
10741	TSPY26P	ACAGGTTGTACCTCGCCCGT
10742	TTC3-AS1	TCTGCAAAAAGGATCATGGA
10743	TTC3-AS1	CTCAGCGGGAGATTCCCAGT
10744	TTC3-AS1	CAAGGGCATCAAAAGCTTCG
10745	TTC3-AS1	AGGAATACGGATGCTGGTGT
10746	TTC3-AS1	AGCCGGTATTGCAGTTTCGT
10747	TTC3-AS1	ACACGAGTGTGCATTACCTA
10748	TUBBP6	GGCCAAATGCCACTACACAG
10749	TUBBP6	GGCATTGTAGGGCTCAACCA
10750	TUBBP6	GCTGACCCACTCACTTGACT
10751	TUBBP6	CCACGGCCAATACCTCACCA
10752	TUBBP6	AGATCCACCAGGATCGCATG
10753	TUBBP6	AAGGATAGAATGTGCCCCAG
10754	UBE2F-SCLY	TTAAGGTAAAAGCTGAGTCA
10755	UBE2F-SCLY	GGAGGACGGTTCTATGTGGG
10756	UBE2F-SCLY	GATTGCCAGTGGGCGGAACA
10757	UBE2F-SCLY	GACTCGGCCGCTCTCGCCCG
10758	UBE2F-SCLY	CCACGGGCTTCCCAGAAGCG
10759	UBE2F-SCLY	CCAAATGCTATCATGAACGT
10760	UBR5-AS1	TCCAGTAAAATGGATAACTG
10761	UBR5-AS1	TATGGGAGGGATGTTGTTTG
10762	UBR5-AS1	GAGCTATCCCAGAGTCCGCG
10763	UBR5-AS1	CGAGCTCGGGAATCTCGAGC
10764	UBR5-AS1	CACCAAGTGGCTAGAGCCCG
10765	UBR5-AS1	CAAGTTGAGATAATCCCTTG
10766	UGDH-AS1	TGCCAGGCTTTAGGGCTGCG
10767	UGDH-AS1	GAGGAATCTGACTAAACCCT
10768	UGDH-AS1	CGCACAGACATCCCAAATCC
10769	UGDH-AS1	CATCTCATCTTAAAACCCAG
10770	UGDH-AS1	ATCTCATCTTAAAACCCAGT
10771	UGDH-AS1	AATGAAAACAACCTGGAGAG
10772	UMAD1	GTACCAGAGACAGAAGCAGA
10773	UMAD1	GGGTGAACGGCACATCGCTC
10774	UMAD1	GACAGCAAGAGGCAAAACTT
10775	UMAD1	CCTGAGATGGAAAATAAGGC
10776	UMAD1	AGGGTCTGATACAGTCACAC
10777	UMAD1	AGAGGCCAACCAACCTTTGG

10778	USF3	TCAGATACAATGCAAACCAC
10779	USF3	GTCACAAAGTTGTTACTAGG
10780	USF3	GGTATTTGGATCTTCGCTGG
10781	USF3	CTGTAGCCACATATCTACTG
10782	USF3	AGCAGTCCATCATTACAGGG
10783	USF3	ACAAAAGCAGACCGCCAATG
10784	VEGFD	TAATTGAGTATGGATGGCGG
10785	VEGFD	CTGTTCAGATCGTTCCAATG
10786	VEGFD	CAGCCCTAGAGAAACGTGCG
10787	VEGFD	ATCGGAACACGTTCACACAA
10788	VEGFD	ACTTCGAATTACTCACTCTG
10789	VEGFD	ACTCTCGCTCAGCATCCCAT
10790	VNN3	TGCTGCAGTATATGAGCATG
10791	VNN3	GTTACCTGTCATGTGCATGC
10792	VNN3	CTCCCACTTAGAATGATCTG
10793	VNN3	CTATGGTTGGATCTTCACCA
10794	VNN3	CCAGATCCCTGCCCTAAGCG
10795	VNN3	AAAATGCCAAACTTCCCAAA
10796	VPS33B-DT	TTTGCAAACATCTGGTGAAG
10797	VPS33B-DT	GTGGTCTTGATTGTGTATGT
10798	VPS33B-DT	GAGACATGAACCATTACTCC
10799	VPS33B-DT	CAATACCTGGCCCAACGTTG
10800	VPS33B-DT	ATCATAAATGTGGAACGATG
10801	VPS33B-DT	AAGTCACCCCAACGGCAGTG
10802	VWFP1	TGGCAACAGGACCAACACTG
10803	VWFP1	TCAGGACTCATGGCACAATG
10804	VWFP1	GGTGTCTTCCACTTTAACAG
10805	VWFP1	CAGTAGAAATCGTGCAACAG
10806	VWFP1	ACAAGGTGCCAGCATACCAG
10807	VWFP1	AACAAGGCCTTCGTGCTGAG
10808	WHRN	TGGCATTTACATCACTGGCG
10809	WHRN	TACACCTGGGTGGACCCGCA
10810	WHRN	GCTGCTCTTCGACCAATACA
10811	WHRN	GATGTCAGATCCACCTCCCA
10812	WHRN	GAAGCGTTCTAGGTCTTGCG
10813	WHRN	ATCCTGACAGTGAAGGACGT
10814	YWHAEP5	TGAGTGATACGATGAAATGG
10815	YWHAEP5	GTGGCATATAGGCCTGGACG
10816	YWHAEP5	GTAATGCAAGCATCTAAGAG
10817	YWHAEP5	GACAATGGGGAGTTTCCAAG
10818	YWHAEP5	CAACCTGCAAGCACGGTCAG
10819	YWHAEP5	AGATGGTTACAGGTCGTTTG
10820	ZNF204P	TAGATGGGAAGCTTGAACGT
10821	ZNF204P	GTGGCGTTTCTGTGGTCATG
10822	ZNF204P	GACTACTTAGAATGATCACA
10823	ZNF204P	ACCCTATGAATATCATGACT
10824	ZNF204P	AAGTGCTAACCAATCACTGA
10825	ZNF204P	AAGACTTACATATATATGCC
10826	ZNF252P	TTATAAGCAAAAGTTTGTCA

10827	ZNF252P	GTTTACTTAACTAAGAACCT
10828	ZNF252P	CCTCAAAGGAAACCACGATG
10829	ZNF252P	ATTTCAGAGAAATTACCATG
10830	ZNF252P	ATGCTGTATCATGTTGCACA
10831	ZNF252P	ATGCTGCAGAACAAACATGG
10832	ZNF32-AS1	TTAGAGGCAGTACACTGCTA
10833	ZNF32-AS1	GATAGGTTGCTTCATCTCAG
10834	ZNF32-AS1	GAGCAAAGGGAGCCTGTCAA
10835	ZNF32-AS1	CATTATCTGTATCACTCTCA
10836	ZNF32-AS1	CAGTTTGCCCTACCCAATGA
10837	ZNF32-AS1	AAGCAACCTATCCAGTTCTA
10838	ZNF33BP1	TTGCTAGAAGACAAACTCTG
10839	ZNF33BP1	TAATTATGCTTATATATACA
10840	ZNF33BP1	GAGACTTGATTTGTGAGTGT
10841	ZNF33BP1	ATTTAGTAAGGACTGAACTG
10842	ZNF33BP1	ACATCAGAGAAAACACACGG
10843	ZNF33BP1	ACATCACAGGCTACACACAG
10844	ZNF436-AS1	TGTTGAGCCTAGTAGAGCAT
10845	ZNF436-AS1	GCAGATAGATCCACCCACTG
10846	ZNF436-AS1	CCTCATCTAAAGTCGATCCG
10847	ZNF436-AS1	CACTGCAGTCCAGAACAATG
10848	ZNF436-AS1	ATCCCCAGTGGAAAGAGCGT
10849	ZNF436-AS1	AGGTTTAAGGATATACAAGG
10850	SEPT10	CCAGATATACCAGTTCCCAA
10851	SEPT10	ATGGATGCGAGAATCATGGT
10852	SEPT10	AAATCCCACTGTATTCACAA
10853	SEPT10	GCAAACTCTCAAAACCAACA
10854	SEPT10	GAAGAACCTTGACAGCAAGG
10855	SEPT10	TTACCTTGTACAACACCCCA
10856	SEPT12	GGACCAAGCATCTCGCAGGG
10857	SEPT12	GCTGTGCCGGACTGTGAATG
10858	SEPT12	CGCTGTTCAAGTCCAAAGTG
10859	SEPT12	GATCAACAATGACAACTGGT
10860	SEPT12	CCCCCAGATGTGCTTTGACG
10861	SEPT12	TGTACTTTGTACCACCCACT
10862	Control	ACGGAGGCTAAGCGTCGCAA
10863	Control	CGCTTCCGCGGCCCGTTCAA
10864	Control	ATCGTTTCCGCTTAACGGCG
10865	Control	GTAGGCGCGCCGCTCTCTAC
10866	Control	CCATATCGGGGCGAGACATG
10867	Control	TACTAACGCCGCTCCTACAG
10868	Control	TGAGGATCATGTCGAGCGCC
10869	Control	GGGCCCGCATAGGATATCGC
10870	Control	TAGACAACCGCGGAGAATGC
10871	Control	ACGGGCGGCTATCGCTGACT
10872	Control	CGCGGAAATTTTACCGACGA
10873	Control	CTTACAATCGTCGGTCCAAT
10874	Control	GCGTGCGTCCCGGGTTACCC
10875	Control	CGGAGTAACAAGCGGACGGA

10876	Control	CGAGTGTTATACGCACCGTT
10877	Control	CGACTAACCGGAAACTTTTT
10878	Control	CAACGGGTTCTCCCGGCTAC
10879	Control	CAGGAGTCGCCGATACGCGT
10880	Control	TTCACGTCGTCTCGCGACCA
10881	Control	GTGTCGGATTCCGCCGCTTA
10882	Control	CACGAACTCACACCGCGCGA
10883	Control	CGCTAGTACGCTCCTCTATA
10884	Control	TCGCGCTTGGGTTATACGCT
10885	Control	CTATCTCGAGTGGTAATGCG
10886	Control	AATCGACTCGAACTTCGTGT
10887	Control	CCCGATGGACTATACCGAAC
10888	Control	ACGTTCGAGTACGACCAGCT
10889	Control	CGCGACGACTCAACCTAGTC
10890	Control	GGTCACCGATCGAGAGCTAG
10891	Control	CTCAACCGACCGTATGGTCA
10892	Control	CGTATTCGACTCTCAACGCG
10893	Control	CTAGCCGCCCAGATCGAGCC
10894	Control	GAATCGACCGACACTAATGT
10895	Control	ACTTCAGTTCGGCGTAGTCA
10896	Control	GTGCGATGTCGCTTCAACGT
10897	Control	CGCCTAATTTCCGGATCAAT
10898	Control	CGTGGCCGGAACCGTCATAG
10899	Control	ACCCTCCGAATCGTAACGGA
10900	Control	AAACGGTACGACAGCGTGTG
10901	Control	ACATAGTCGACGGCTCGATT
10902	Control	GATGGCGCTTCAGTCGTCGG
10903	Control	ATAATCCGGAAACGCTCGAC
10904	Control	CGCCGGGCTGACAATTAACG
10905	Control	CGTCGCCATATGCCGGTGGC
10906	Control	CGGGCCTATAACACCATCGA
10907	Control	CGCCGTTCCGAGATACTTGA
10908	Control	CGGGACGTCGCGAAAATGTA
10909	Control	TCGGCATACGGGACACACGC
10910	Control	AGCTCCATCGCCGCGATAAT
10911	Control	ATCGTATCATCAGCTAGCGC
10912	Control	TCGATCGAGGTTGCATTCGG
10913	Control	CTCGACAGTTCGTCCCGAGC
10914	Control	CGGTAGTATTAATCGCTGAC
10915	Control	TGAACGCGTGTTTCCTTGCA
10916	Control	CGACGCTAGGTAACGTAGAG
10917	Control	CATTGTTGAGCGGGCGCGCT
10918	Control	CCGCTATTGAAACCGCCCAC
10919	Control	AGACACGTCACCGGTCAAAA
10920	Control	TTTACGATCTAGCGGCGTAG
10921	Control	TTCGCACGATTGCACCTTGG
10922	Control	GGTTAGAGACTAGGCGCGCG
10923	Control	CCTCCGTGCTAACGCGGACG
10924	Control	TTATCGCGTAGTGCTGACGT

10925	Control	TACGCTTGCGTTTAGCGTCC
10926	Control	CGCGGCCCACGCGTCATCGC
10927	Control	AGCTCGCCATGTCGGTTCTC
10928	Control	AACTAGCCCGAGCAGCTTCG
10929	Control	CGCAAGGTGTCGGTAACCCT
10930	Control	CTTCGACGCCATCGTGCTCA
10931	Control	TCCTGGATACCGCGTGGTTA
10932	Control	ATAGCCGCCGCTCATTACTT
10933	Control	GTCGTCCGGGATTACAAAAT
10934	Control	TAATGCTGCACACGCCGAAT
10935	Control	TATCGCTTCCGATTAGTCCG
10936	Control	GTACCATACCGCGTACCCTT
10937	Control	TAAGATCCGCGGGTGGCAAC
10938	Control	GTAGACGTCGTGAGCTTCAC
10939	Control	TCGCGGACATAGGGCTCTAA
10940	Control	AGCGCAGATAGCGCGTATCA
10941	Control	GTTCGCTTCGTAACGAGGAA
10942	Control	GACCCCCGATAACTTTTGAC
10943	Control	ACGTCCATACTGTCGGCTAC
10944	Control	GTACCATTGCCGGCTCCCTA
10945	Control	TGGTTCCGTAGGTCGGTATA
10946	Control	TCTGGCTTGACACGACCGTT
10947	Control	CGCTAGGTCCGGTAAGTGCG
10948	Control	AGCACGTAATGTCCGTGGAT
10949	Control	AAGGCGCGCGAATGTGGCAG
10950	Control	ACTGCGGAGCGCCCAATATC
10951	Control	CGTCGAGTGCTCGAACTCCA
10952	Control	TCGCAGCGGCGTGGGATCGG
10953	Control	ATCTGTCCTAATTCGGATCG
10954	Control	TGCGGCGTAATGCTTGAAAG
10955	Control	CGAACTTAATCCCGTGGCAA
10956	Control	GCCGTGTTGCTGGATACGCC
10957	Control	TACCCTCCGGATACGGACTG
10958	Control	CCGTTGGACTATGGCGGGTC
10959	Control	GTACGGGGCGATCATCCACA
10960	Control	AAGAGTAGTAGACGCCCGGG
10961	Control	AAGAGCGAATCGATTTCGTG
10962	Control	CGGCTCGTTCTACGCACTGA
10963	Control	TCCAGCGCGAGCTTACTCGT
10964	Control	GGAGTGGTCCCGTACGCAAC
10965	Control	CAAGACCTTATCGTGCAGCG
10966	Control	ACGTCGTTTAGCACCCGGCT
10967	Control	TTTCAGGCTACGGGCGCGGG
10968	Control	CAATCGGCGACGTTTTAAAT
10969	Control	GTACCCCTATGGCCGTTCTA
10970	Control	TACCCACGCGTATTCCATCT
10971	Control	CTTGTTGCGTATACGAGACT
10972	Control	GAACGCCGCTCAAGTTGATA
10973	Control	CGCAGTACGTATAGACTTAA

10974	Control	GCGAACCCCGTAGCCAGGCT
10975	Control	CCTCGGGCGTAAATACTCAT
10976	Control	AGGGCACCCGGTTCATACGC
10977	Control	CACGGCCTAACTTGCTGATA
10978	Control	CCGGGAGATTAACGTTAATT
10979	Control	ATCTCGGGTCGACTGCGGAT
10980	Control	CGCCGGGACCGTTAGGGAAT
10981	Control	GCAAACCCGAGTGACACGTC
10982	Control	GTGCGTGAGTATTAACGCTC
10983	Control	GCTTAACCTATCCGCCAGTA
10984	Control	TGGCCACGAATTCCGCCGCC
10985	Control	GTAAGGCCCGCGTACGAGCT
10986	Control	CTCCTTACGTCGGGCATTAA
10987	Control	ATTCCTTCGGCGCTCTGCGT
10988	Control	ATGCGCTTTAATCGCCGTTC
10989	Control	TTAGCCCTCGATTGGTTGCG
10990	Control	AACGCTGTCGTACGTGTATA
10991	Control	TAACGCGCATATCTGAACAC
10992	Control	CGCTAGGTTATTTCGTGGCC
10993	Control	AATATTTGGCTCGGCTGCGC
10994	Control	CAATATCGGGTGCTACAGGA
10995	Control	CGGCCGCATCCTGTTATATT
10996	Control	CTGGATCGCCCGCAGAAATA
10997	Control	ATTAGCACGGCGACCTTCTA
10998	Control	TGGAAATCGACTGTGCGCTT
10999	Control	ATTAGCCGTTGCCATATCAA
11000	Control	ACCCGATAATAGCTACTGGT
11001	Control	ATTCAGCGCGCTCGCCCTGG
11002	Control	CCCGCCGAAGACCCTGCTTG
11003	Control	TGCCGTGAAAAGACGCTGCG
11004	Control	CTTACGCGCCTGGTCAAAAG
11005	Control	CGCATAAGTCGATAGACACA
11006	Control	GTCATCAGCGATTTGACGAG
11007	Control	CGAATCGGAACTTTGTACCG
11008	Control	AGGTCAAGCCGACCTCGAAC
11009	Control	TGCGCCTTACTCGTTAACTG
11010	Control	ATCTGAGCGTTTTCGGCCGC
11011	Control	ACGGTACATGCGCATGAGTC
11012	Control	TGACGCGATAGAGTTGGCTT
11013	Control	TTCACCGTCCACGTGCGCAT
11014	Control	TTCTTCGGCCTACACCCGGT
11015	Control	GGAATTACGACTAACCGATT
11016	Control	TGCGGGGAGCTCTCTTAATG
11017	Control	TAGAGATATCCGATCGTGGT
11018	Control	GGGTGCCCACTAATAGCCGC
11019	Control	TGCAGTCGCGCTGAGCGTCA
11020	Control	GGATTGAATGGCTAACGCGG
11021	Control	GCGGTTCCGGGAGCACATTT
11022	Control	GAAGACGTGCTGGCGTCACC

11023	Control	GGCGCATTAAAGTCGAGAGC
11024	Control	GACGTAGCCTTCCGAAATAT
11025	Control	GGTTCGAGACCTACTTAAGT
11026	Control	TGAAGTGGGGCGTCGGACAC
11027	Control	CTATATTGTCGCGCAGTGGA
11028	Control	CGGCTTTGTTGCCCGTAAGC
11029	Control	TCCCGGTTGGTGAACGATAC
11030	Control	TTAACTCGAACGCTCGAAAG
11031	Control	GGACTATCCACCGTTTACTC
11032	Control	CAGTAGCGATCGAATGTCAA
11033	Control	TAGGCGCCCGTAGCATTGGA
11034	Control	GCGGCGTCTGGGAATCGTTC
11035	Control	CAGTGTCCGAGCGATATTTT
11036	Control	TTCAATCACCTCACGGTAAG
11037	Control	CGGTTTACATCTGCCCATCG
11038	Control	GGGCGTGTATGTTCGTATTG
11039	Control	GCGGCGTCATCCACGCGTGC
11040	Control	GGGTATAGACGCGATCCTCA
11041	Control	ACAGCGCTCTCGTGTACTAT
11042	Control	ACTAGCCTGTTCGCGAGTAG
11043	Control	GACCGCGTGAGATAACGTCA
11044	Control	CGCGTGCATCTGCCGAAGGC
11045	Control	AAAACATCGACCGAAAGCGT
11046	Control	ACACCGAAGCACCTGTACGT
11047	Control	GGGTCGAGGACTAATAGATT
11048	Control	ACGCCTCCTCAAATTAGCTC
11049	Control	CCTACGCGGTAGGGAACTTT
11050	Control	AAGCACTAGTCCGTATGATG
11051	Control	AGGCGCCAACATTGACCGTA
11052	Control	TAATCATGCACATTCGGGAC
11053	Control	CGTCGGGTAGCTATTTCTTT
11054	Control	GTGCGACGAATTGTCCTGAG
11055	Control	CTTCCGTTATTCGGAAGTGA
11056	Control	TACTGGAGTTTGCGACTCGG
11057	Control	ATCCAGATCACCACGCGTCA
11058	Control	CGTTAAGACTAGCTCGGTTT
11059	Control	ATACGGCCGAAGCCCCTTCA
11060	Control	AACATCTCGTTAGGGGTATC
11061	Control	GTCAGGTAATAGTCGGACTC
11062	Control	TTCGAGGTCCGGACAGGTCG
11063	Control	TGAGCATTCGTAGCCCAGCA
11064	Control	TAAGGCGACCTGCGCTTGTG
11065	Control	AGCTGCGCGCTACTGGATCA
11066	Control	GCAAAAACCCAACGCTATTC
11067	Control	GCCGCCGATTTCATAAGTAA
11068	Control	GTTCCGTGAGGGTTACTTCA
11069	Control	CGGGGAATTGCACGGCGGAA
11070	Control	TGTCTTTAAACACGCCATCG
11071	Control	ACAAAATGCCGTGCGTCAAT

11072	Control	ACGCTCAGCACCCGCTATGC
11073	Control	CGAAACCCTCTTAAGTTAAC
11074	Control	TAAACCCTTGGCGCCCTCAC
11075	Control	CCATTCTCAACCGGTCCAAT
11076	Control	GTTATTGACCCGTCGGGAGT
11077	Control	GGTTTCACTTCGAGACCGGC
11078	Control	CTTTGATAACGTGGGCGGTA
11079	Control	CCCCAACTTTCGCGACTCCG
11080	Control	CAAACAGTCTAAGGCGACGA
11081	Control	CGGCACACCAATGCGTTCGT
11082	Control	ATCGATATACCGCCATAAAA
11083	Control	GGGACGCGAAAGAAACCAGT
11084	Control	AACGCGAGTCCGGTGGCTAG
11085	Control	TGTCATTAGCGTAACGATAT
11086	Control	AGGGCGAGCAGCAGAGTACG
11087	Control	CCGTCTCCGCATCGTCTTTT
11088	Control	CGTGCGTGAAATCGCTAGCT
11089	Control	ATCAGGCCTTGTCCGTGATT
11090	Control	CGTCCAGAAGAACGGCCCCT
11091	Control	AGAGCGTCTTCGTGACAAAC
11092	Control	GATGGCGCGCAGTTGAGTCA
11093	Control	ACGCTGACGAGTAAAAGCGG
11094	Control	TATCAATCGTCCGGGTCACT
11095	Control	TCAGATTCCGCAAGGGTCCA
11096	Control	GCGATCGGAGTGCCACGATA
11097	Control	ATATAAACTGTCGCGGTAAA
11098	Control	GTTACCTGCTACGAAAACGA
11099	Control	GAAGCGATCCGAGAGTGTAT
11100	Control	ATGACATTGCGCGTCTACGG
11101	Control	ATACCAGATGCGTCCGCTTG
11102	Control	AACGTGCTGACGATGCGGGC
11103	Control	CGGCACCTGTTAGGCTATTC
11104	Control	AGGATCGTGTACCGGGGACG
11105	Control	CGACAACGTGCAGGTGTATC
11106	Control	ACTCGAAATCACCTATGGTA
11107	Control	TCTCGTAGCCTAATGCGCCA
11108	Control	TTATGTGAGCACGCCATTAC
11109	Control	CGACGGTAATGCACCTACTA
11110	Control	ATCTGTGTGACTGCGGTCGG
11111	Control	TGGAAAGCGAGCACACCGTC
11112	Control	CAGCGCCGAAACTCTTTCCG
11113	Control	TGCCGTTAGCATGCGATCCC
11114	Control	TCGTAAACACACGACCAAGT
11115	Control	ACTACTCCGGCAAATACTCG
11116	Control	GCCCTTCAATGCGTTCCGTA
11117	Control	CAACACCCCGCGTTATGCTA
11118	Control	GCTGATATATACGACAAGCC
11119	Control	GGTATTCCGAAGTCCTCCGC
11120	Control	GGTCGCCTGTGCGACATGCT

11121	Control	CTAATCACGACCTCACCCTA
11122	Control	ATCGGTACCTCTTCACATAT
11123	Control	TTGCGTCAGCGCTGCACATC
11124	Control	CGGTGTGCCCCCAAATATTG
11125	Control	CGTGCGGTAAATACGAAATA
11126	Control	ATACGAGGCGCTTTTCTTTG
11127	Control	TGTGCACAAGTCGCAACGAA
11128	Control	CTCGGCCCACTTGCGTTATA
11129	Control	TATACTGCGGATCAATCTGA
11130	Control	AAAGCGACGTAGGCATACTT
11131	Control	ACGATCGGTAATGGTCTGTT
11132	Control	GGGCCTACGATCAGAGGTGT
11133	Control	CGCAGTCATTCGATAGGAAT
11134	Control	GGCGATTCGGGTGGAGGTCG
11135	Control	AGTTGAATGGACCTCGACTA
11136	Control	GAGTAATTTCGAACGTATTG
11137	Control	TTCCACGGTAAAATCGGTCA
11138	Control	ACTGTTGCCGCGCCAACTGC
11139	Control	CCGGCAAGAAACTATACTTG
11140	Control	TAGTGGGAATGGTCGCGTAG
11141	Control	CCGCTGTCTCACTAATCTCA
11142	Control	TGCTACCTTCGGGACCACCA
11143	Control	CTTAGCTGACCGACAAGGTG
11144	Control	CTTAAGCTTATCAACATCCG
11145	Control	ACGGAAAGACCTCGCTATTC
11146	Control	CCCTTCTGGCGGGCCAAACA
11147	Control	TCTGACGATTAATGCTTCTA
11148	Control	GCGGAATGAACACGAGGTAG
11149	Control	CAGACGGTTGGTAAGGACGC
11150	Control	GGGACTGATATATGGCGAAC
11151	Control	AGAAGACCGAGGCGCTTCAA
11152	Control	CAGGTTTGCACGCATAGCTA
11153	Control	GGCCGTCGTATTCCCCCAAG
11154	Control	CTCCCATTGATCTACGATGG
11155	Control	TTTCGTGCCGATGTAACACA
11156	Control	ATCACGTGATCGGATGGTTC
11157	Control	GCCTATCGGCATTCCCACTG
11158	Control	CAACGACGGGCCTAGTCTCA
11159	Control	ATATTTCGGCAGTTGCAGCA
11160	Control	GATATCCCGCGAAAAAATCT
11161	Control	TCACTTGTCTATACGGGGTA
11162	Control	ATGTTATTACAGACGCGTGT
11163	Control	TGGGAATTCCTCGGCCGATT
11164	Control	CGCCTCTCACGTGTAGGCTT
11165	Control	GGGCGCTAAGATATATGCCC
11166	Control	CGTCATATACACAAACGCCC
11167	Control	CGTTGGGCATAGCGAACACT
11168	Control	GCGGGGCGGTGACTTTCAAG
11169	Control	AAGGGCGTGCCCTGCGTTGT

11170	Control	GCGTTCCCCCACTGACGGGG
11171	Control	GATCCAGGAGTGATCGAGTA
11172	Control	AGCATTTGCGCGGCAACTGT
11173	Control	TTGTCCCTGAGAAAACGCGG
11174	Control	GTCCTCATCCGGTCAGGCTG
11175	Control	TATAGCTGTTTCGAAGGCGC
11176	Control	CGTGGTAGGGGTTTCCTCAA
11177	Control	CCATCACCGATCGTGAGCCT
11178	Control	TGAATCGTAACCTCGCCATT
11179	Control	AAGAAGGGCCGTACCCGAAA
11180	Control	CGTGTTTGGAATTTGCCGCG
11181	Control	CAAGTGAGCATAAGCGATGT
11182	Control	AGGACTAGTGTCGCACTCAG
11183	Control	GGCACTCCGAAAGACCTTAT
11184	Control	CCCAGGTGTATAATGAAACG
11185	Control	GACCGCAAAGTGGTCCGAAG
11186	Control	GTTGCGAGTTACTATTGGTT
11187	Control	TCTAAAGCCGTCCTGATGTT
11188	Control	GCCGTGGTATCAAGTCGGTA
11189	Control	CGCAATCCCTTAGGATAGCC
11190	Control	ATAGGCACCTTAAGGGTCTC
11191	Control	TTTGGGTTTATCCGCCCCCA
11192	Control	CTAGAGGGGTATAGCAACAA
11193	Control	CTCGGGACTAACGTGAGAGC
11194	Control	CCCCGTAGCTCATTAGTCTG
11195	Control	GAAAACACGATGACGTCTCT
11196	Control	GACGCCCTAATGCCCATCGT
11197	Control	GGATATTGAGTAAACCCGAT
11198	Control	TGACTCGGGCAATATCGGTT
11199	Control	GATCTAGTCCTCTAATCGAT
11200	Control	CATAAGGTTAACTGCGTGGA
11201	Control	GGTACCTGAACAACGGCACC
11202	Control	TGGCGGCCCAAACTTAACAC
11203	Control	GGGCGGTCAGGTCGCTCCGA
11204	Control	TCCGGAGGCTCAAACCAAGT
11205	Control	GCTTGCTATATGGGTGCGAG
11206	Control	TCGGCTACGGCGTGGAGAAG
11207	Control	CCCGTGGCGTGCGCACCTGT
11208	Control	GGCTGGTTGACGACTCCTGA
11209	Control	GGTGCGGTCTGCTTGTAGAT
11210	Control	TGCCTATGACCTAGGTACCA
11211	Control	GACAGATCACGTATCAGGGC
11212	Control	GAGGTATGTCATCGCCATGA
11213	Control	GCCATTCTAGTCCCGGCATA
11214	Control	TGAATCGAATACAAACGATG
11215	Control	GTTACGTACCCTCCAACTTC
11216	Control	CCAATGATAAGCCCGAACGG
11217	Control	AGCGATTCACGTATTAGATG
11218	Control	GCCAACAAGAATCGGATCCC

11219	Control	ACTTACGGCACTCGCATGCC
11220	Control	GATATGGCTCGAGTAATCTT
11221	Control	GTATCCCATATCGGCACAGG
11222	Control	ATGCTGCAGCTTTACGATCA
11223	Control	GAGTGATGCTTAGACTCCGT
11224	Control	ACATACCCCCCTGGTTCAGA
11225	Control	GCCCCTTATGATTGTTATAG
11226	Control	AGGTCGGCCGAACATACGGT
11227	Control	ATTGCGCAATCCTTAGGATA
11228	Control	GAAGCGGGACCGTGTCTCAC
11229	Control	GTGTATGATGCTTCGACTTA
11230	Control	CCTACTCCCGTGTGTTATCC
11231	Control	ACAGCCCTCACGAGCCCGAA
11232	Control	GCTGTTGTAACGGTAGATAT
11233	Control	TACGAAACATCATGGGTTCC
11234	Control	CGCACGACCATTGCTGCTGC
11235	Control	CATTGCACGCCACAGCATTG
11236	Control	CGATGCCCGTCTATGGCCCG
11237	Control	CCAGCAATACCCCGGTATGG
11238	Control	TCGAGATGCGCAGCAGATGA
11239	Control	ACGGGGTGAAACCATGTCGT
11240	Control	CCTTGGCGAGACGGAGGTAC
11241	Control	CCATAGCTCTAGCGATAAAC
11242	Control	AGCTAGCGATGGCTCTAAGT
11243	Control	CGGGTCTCCAAAGATCGCTT
11244	Control	GGTCCGCGCACAAGAGCAGG
11245	Control	CAACCGGCGGGCCCCTACAA
11246	Control	TCCTCGATAGCTGGAATCCA
11247	Control	TACGGATCACCAAATCTTAG
11248	Control	CTCGCACCATTGAGGGTAGT
11249	Control	TCGGGGACCACCCACGATCC
11250	Control	CCTGCGGTGCACGGCTAGCC
11251	Control	ACCGCTCATATAGGTAAAAA
11252	Control	AGTATTGTGGTGTCGTCAAC
11253	Control	TGACGCTCCACGTCCGGACC
11254	Control	GCTCGCAAGTATTTAAGGAC
11255	Control	GCCAGGGTTCTTGGTCCCGA
11256	Control	GTCGCTGCGCCAGTGAGAAC
11257	Control	CCCGCTAACAGCCTAGATCA
11258	Control	TTGTACGGTACTGGCTTTGT
11259	Control	CAGGCTGCGCTTCGCAAGCT
11260	Control	GCCTAGTCTCGGTAAGAGTG
11261	Control	GGACGCACCATTCCGGGTGA
11262	Control	GATTGTGGTCGCTCAAAACC
11263	Control	CTATAACCGAATCAAAGGGT
11264	Control	AAATTGGCTTTCGTTCGTGC
11265	Control	TCACCTCCGAACGAACACCT
11266	Control	GGAGATGCGGCCTTCTCAAA
11267	Control	CTTAGGATTCCGAGGTATCT

11268	Control	GAACTGGCAAACAGGCGTGG
11269	Control	CCTGTCGCTATCGTTAGAGA
11270	Control	ATAGCAGGACGAGGTTCCTT
11271	Control	GGCGTCAAAATTAGAAGCCG
11272	Control	TCGGCTACAATCTTTGGCAT
11273	Control	GCACGCTGTACAGACGACAA
11274	Control	GAGAGCGTTAGCGTGGGATG
11275	Control	TTCAATTCACCGAGGGCGCA
11276	Control	TTCGTGGTAGGTATAACTAT
11277	Control	TGAGCGGCCTCTAATTAATC
11278	Control	CGCTAAATTGTCACACGTTT
11279	Control	GGTTAACATCGCCACTCTGA
11280	Control	GTTTCTCTTGAACCCCGCGA
11281	Control	ATGTCTAGACCTAATCGTTT
11282	Control	ATTCATGCGCCGCCTCCTCT
11283	Control	ACCCATTGAGAGTCGCCTGA
11284	Control	CACCCGGAACCACTACTGAG
11285	Control	GCTGAACGCCGACAGGACGG
11286	Control	GCCCAGACGCCCTAGAATAG
11287	Control	GGGATGCGTCTTGCTAAACC
11288	Control	ATCGTTGCTGACAGGATCTA
11289	Control	TAGTCTCACCTGATGGCGTG
11290	Control	CCCTACCTATCCGGTGCTTT
11291	Control	GTTATCCTGTCGAAGTAAAG
11292	Control	TACTTTTTGGAATCCGTCTA
11293	Control	CAGCGGTGCTATTTGGTCTT
11294	Control	CTGCGTGTCTTGCTCGCATG
11295	Control	CCTAGTTCATAAGCTACGCC
11296	Control	TGTCTTCGGATAGGCGGCTT
11297	Control	CGCACATCTAAAGTTACTAC
11298	Control	GTAGGGTACAGCGTCAGCTT
11299	Control	GAAATGCTATGCTTCGGTTC
11300	Control	TCAACCCCAGCGCACCGTTG
11301	Control	AATGCGAGTGTATCCGCAGT
11302	Control	TTTATGCATTTAATACGCCG
11303	Control	CATTGCACTCGTTGCCAACG
11304	Control	GCCCCGTAAATCTCATTACA
11305	Control	TCCGTCTGCTTCATGAGCGG
11306	Control	CTCGGAATACTCCTATTACA
11307	Control	CTAACGGACTGCAGAACGGA
11308	Control	ACAAAGATCGCATGGCATAC
11309	Control	ATCTTCTCGACGAAAATGCG
11310	Control	CATGGCCTACGGTGTCTTTG
11311	Control	CTGGCCGAATCTCACTATGT
11312	Control	GGGGCTTACGTGAAGGGCGG
11313	Control	ACACCCATTCTCATAACGGA
11314	Control	GGCCACGAAGGGCGAAAAGG
11315	Control	TAACCGATACTCCCCACATT
11316	Control	GCACATCGTTATATACCAGA

11317	Control	CGAGCAAAGATTGTTGGATA
11318	Control	GCCACACGAATCATAAAGAG
11319	Control	GAGAGTGCGCCTTGATAGTA
11320	Control	GGATTTGTCGCTTGCCACAC
11321	Control	GGCTTACGTGGGGGGCAAAA
11322	Control	CGTTGTAGGGTCTTTCTTGA
11323	Control	ATTGCTCTGTCGCATCAATC
11324	Control	CTCAGTGGATACGATTTGCT
11325	Control	ACTACTGGCTATCCGCGCCA
11326	Control	ACCCAATGTGGCGGAGCCGA
11327	Control	ACCTATAATCGACCACATTT
11328	Control	TAGGAGCTGTATCTAGTGGC
11329	Control	CCAATCTTGAACGTCATGTT
11330	Control	ACCCATATATGCTGCCGCAC
11331	Control	GCCAGGGTATGGGCATCTCG
11332	Control	CATAGGTCCCTAGCAACTCC
11333	Control	ATACGCATGATTGCAAGAGG
11334	Control	TTCGTAGGAACTAAACTGTA
11335	Control	TTTGGTCAACGCATAGCTTG
11336	Control	CGGTGCTGTGAAAGCCGAGC
11337	Control	GGCCCTCTAGAAAAGTCTCG
11338	Control	ACGGTTATGGTCTCATGGGG
11339	Control	AACTAGAATAGGCGGGCTTG
11340	Control	TAATCACATTGCTTAACCGG
11341	Control	ACTGCTCCCGGTCGCCCCTC
11342	Control	CGCCCGTTTATGTGGCTACC
11343	Control	GAGTACAGCGATTCCTCATG
11344	Control	TTTCTAGTTACTACTGGACG
11345	Control	CACGCACAATCCTTCACGCA
11346	Control	TGCCGCTATACTAAAACCTT
11347	Control	GTTTACTCATATCCAGTCAC
11348	Control	TCGGCTCCTGAAGCCAGTAT
11349	Control	TCGATGTAGCCCCGCCCAAG
11350	Control	AGACCCCGTAGGCAGGACGT
11351	Control	TCCCAAGGGTTTAAGTCGGG
11352	Control	TATACCAGACCACAGCGCCG
11353	Control	CGTGCCTTTACATTCACTTT
11354	Control	TTTTGACTCTAATCACCGGT
11355	Control	GCTGTTCCGAAGTTGAGAAT
11356	Control	GCTTAAGTCACGGCTTTCCA
11357	Control	ACTAGAGTCATGATCAGCGA
11358	Control	CTGCCCCAGGCGTAATCCTC
11359	Control	GTCCCGTGATTTTAGCCAGG
11360	Control	GCTTCGCTGGCAGGACCTCA
11361	Control	CGTAATTTTGTAATCGCTTC
11362	Control	AGTCTTGGCCAATGTCACGG
11363	Control	CCCGGACGTCAAAGCTGGAT
11364	Control	GGTCTCACCTGCACCCCGAA
11365	Control	GGTAGACGGGGCATCTCAGC

11366	Control	ATGTAACGAGTTGTAAGTCA
11367	Control	TAGTCAACATTCGCAAGAGG
11368	Control	TTTACGAAGTATACCAGGTC
11369	Control	GTAGCTGCTGTAAATCGCAT
11370	Control	CGAAACCTCCTAACTGAGAG
11371	Control	ATAAGCCACACTACCCGCCT
11372	Control	TACGTAAGTGACGACAGGAA
11373	Control	CTTTATCTGGCGTGGGGTAT
11374	Control	TTAATTTGGGTGGGCCCTGC
11375	Control	CCACTGGTAAAGGCTAGACT
11376	Control	CCCCTATGCAGACTACAATT
11377	Control	CTGGTGACCGACAATTACAC
11378	Control	CTGTCCACCTACAGCGATGT
11379	Control	ACGTGGGGACATATACGTGT
11380	Control	TCTGGCTTGCACCGTGTCAT
11381	Control	GTCCAATAAAAAGTGCCACT
11382	Control	GTTCCCCGGGAAGTCTATGC
11383	Control	CTGACGTGTCTGAAATGAGT
11384	Control	ATTTCCCTACGGAGATATCC
11385	Control	ATCAAGTCAGGTTATGCGGG
11386	Control	GGATACCTGGGCCGACTTTC
11387	Control	CTCCGTTATGTGGCATGAGA
11388	Control	CGCAGGCTAGATGACACCAG
11389	Control	TTCGGAACTTACTCAGGGTA
11390	Control	GCGGGCAGAACGACCCTGAC
11391	Control	ATAGGTCATCCACTGGGCGG
11392	Control	AAGCGGGCACACATGACAAG
11393	Control	CCGTGTTCCACAACTACCCT
11394	Control	GTAAAGAAGCGGAAAGGTCC
11395	Control	GCCTGCCCTAAACCCCGGAA
11396	Control	TACGTCATTAAGAGTTCAAC
11397	Control	CGATGGATCCCTAGTTCCTG
11398	Control	GCTGCGGCGAGATCACATAA
11399	Control	CGCGCACCACGGGCGCGCAC
11400	Control	CATTGTATGAACGCAATAGC
11401	Control	CAGAGCCTTGCGCAATTTTG
11402	Control	TGCAGGTCTAGGTCCCAAAG
11403	Control	CCGCGCATTTCAGAGCACAA
11404	Control	AAAACAGGACGATGTGCGGC
11405	Control	ACCTATTGTCCCTTCAAGCT
11406	Control	AAAAAGCTTCCGCCTGATGG
11407	Control	TTGCAAAGCTGATCGGCTGT
11408	Control	AAAATTATCGGAAACGGTAG
11409	Control	CTGTATTAACGAACCTTGTG
11410	Control	AGTCATAACTGAGTGAATCG
11411	Control	TAGTTACAGACTCAGCGGGT
11412	Control	CCAGGCTGAAGTTCGTACCT
11413	Control	TATTTATACACTATGCGGCA
11414	Control	CACTTACACATGAGGCGGTA

11415	Control	TTTCGCCCAAGAGGCTTGGG
11416	Control	CGCGTGTAGCTGGAGACAAG
11417	Control	CGGAGTTAACCTGGAACCTT
11418	Control	AGCGCTCTGGTTGCATCCCT
11419	Control	ATAGAAGTGTGACCGCTGGG
11420	Control	GGTTGCTGTGACGAACGGGG
11421	Control	GTATTAAGATGCGTCTTAGA
11422	Control	GGCCACAAAAACTCGCTAAG
11423	Control	TCTCTCGGAGTGGAGCAACA
11424	Control	GAACCGACTTGAAGGGGGCT
11425	Control	ACTGAGTGGGTAACACGCAT
11426	Control	CCTAAGGGGTACCACCATGG
11427	Control	TCCCCGAGACCATCTTAGGG
11428	Control	CCTAAACTCAGACGCACTAC
11429	Control	TACCCTGGATTGTCCTTGCG
11430	Control	ACGCCATATTTCTGGCTCTA
11431	Control	CATCTGTAGGGTTGCAAGCC
11432	Control	TAGCTCGAGTCATTTCTCTA
11433	Control	TTTAACTGTCCCGGTGTGCA
11434	Control	CCTCGTCCAGATTCCGGCGG
11435	Control	AATGGGTTGCCGGGAGTAAA
11436	Control	TGGATCGGCAGTGGTACTGG
11437	Control	AAATACAAGCTATAGCGATA
11438	Control	CATGAGCGCATTGAATAATA
11439	Control	GACTTTGGTTGAGCTTCAAT
11440	Control	GTTGGCATATTGGCCCAGAC
11441	Control	GGAACCCTCCCTGCGATAGA
11442	Control	CTCACGGGGACATACAGGGC
11443	Control	GATCATAATCGCTTCAAGCA
11444	Control	CGACCCGGAGGATGAGATGT
11445	Control	TATTTTGACTTGACGCAGGC
11446	Control	CGGGATGGTCCCTGCCGAGA
11447	Control	TAGATTGGCCCCACAAAGCG
11448	Control	CCACACCTGTCTAGCATGAC
11449	Control	GAACCCAACCTTTTACCGCA
11450	Control	GTACACACTTATGCCATCAC
11451	Control	GAGTCGAAGATGGTCTAGGA
11452	Control	TTCCTGCCCGAACTGCAGAA
11453	Control	CGGCTGAGGCACCTGGTTTA
11454	Control	AGGTTGAATACCCCTTACTA
11455	Control	CCTGCGCGTAGAACAGTGGT
11456	Control	CACGCTTCTGCCATCACCGG
11457	Control	AATCGCAGGTATCCCAGAGC
11458	Control	TCGGGCAGTGAGTACAATAC
11459	Control	GAGACCACTTTCGTGCAAGC
11460	Control	TGTCCCTTGCAACCTCCGTT
11461	Control	ACAAACGACCTTGAGCAGGG
11462	Control	GTACATTCCAGTATTCACGC
11463	Control	GGAGAGGGCCCGCGAACTCA

11464	Control	GGCTGGTTGACCTTCCCGCT
11465	Control	GATGTGATCTATGGTTGCGA
11466	Control	ACGTCAACTGCTGGAGTGGG
11467	Control	ATTTAAACCGTTACACAGTC
11468	Control	CACGCCAACTAAAACTGCAG
11469	Control	CCTAGAGGTCCCAAGGCGTG
11470	Control	CCGTTGATCCCCAGGCGTGC
11471	Control	CCTCGATGGTCACCTGTAGC
11472	Control	GTGCGCATGGGCTGATGTTA
11473	Control	AGACTCGTATTGTCATATTA
11474	Control	GGATCTAGCTACCTCAAAAG
11475	Control	AGAACCCAGACGCCAGCGGT
11476	Control	CGAACTTCTGGCTGCAGTTT
11477	Control	ATATGACAGGGCAAACGCAT
11478	Control	GGGACATCCTTGCCGTCTCA
11479	Control	AGCATTCTCACCAAGACCGA
11480	Control	ATAGTAACGTCAGGGAGTAA
11481	Control	CGAATATTATTTCTATCGGG
11482	Control	GAGTGTAAGCTAACACTCTG
11483	Control	ATACAATACTTTGGCGCATA
11484	Control	CTCCCTGCCGGCCGGGTTAG
11485	Control	GAACCTCCCCGAATATCTGG
11486	Control	ATCTTCAGGGTAACTACGAA
11487	Control	TTCTAAGCCACGTGTGGTAC
11488	Control	AGAAACTGAACTATCCTACT
11489	Control	CAGACCTAGCTCCGCTCCCA
11490	Control	AAATGCACAGATCGCTGATC
11491	Control	TCAATTCTCACTCACGACCA
11492	Control	ACGGTGGGGATGGACCTACT
11493	Control	TGTGTTAGCCGAGATCTCTG
11494	Control	GCTAGTGTCGTGGGGGATGA
11495	Control	CGAAGTCTTTCTTAGATGGT
11496	Control	TATTTCTCCTAAGTCTAGCG
11497	Control	ATGCGAAACGACATTTATTA
11498	Control	CATGATAGATCAGTCTTCCC
11499	Control	AGTGGGGCGCTAAGTGGGGG
11500	Control	CCCAATGGCTTCTGCGTGAC
11501	Control	AGATTCATTCACGAGTTGGG
11502	Control	AGCTTTCGAAATTGAGTGTC
11503	Control	CTAAGTTTGTTAATGGGCCA
11504	Control	CTTTTTTTATTTATCGATCG
11505	Control	TGTAGCTAAGTGAGTATGCC
11506	Control	AGTAGACGGACGGTGAGCTG
11507	Control	TTGTCCGTGACCCTGATTAA
11508	Control	TCTACGTGTAGTTGTACATA
11509	Control	CAATGGCGTGGACATTTGAT
11510	Control	TTCAGCGAGTGTGACTAAGC
11511	Control	CACATGGGGTACAGCACACC
11512	Control	CAAAAGTCTCGCTTGGTCCT

11513	Control	GGTTTTATAAGGGTGGGCCT
11514	Control	TCGGAAGCAAACTTCTGGAG
11515	Control	ACTATCATGGCACCCAATTG
11516	Control	TTAGCCAGTAGTGCATATGA
11517	Control	GGGACTGTAGGAACATCCGC
11518	Control	AAGAATTAGGCACGGTTACT
11519	Control	TTTTTCTCACCCGATGAATC
11520	Control	AAACCCTATGCCCAAATGAG
11521	Control	AGGCTTAAATGGCGAGATTG
11522	Control	CATTAGTCTGATACCTGTGC
11523	Control	GGTGCTTAGCTCTGCGCACA
11524	Control	AGGCCACAAATTGTATACAG
11525	Control	ATGCCTTAGACTTAACCTCG
11526	Control	CCAGTGCCCTTTTGTCGCAA
11527	Control	AGCGATCTGGACACTCTCCA
11528	Control	AGTCTTAAAGACCCTAAGCT
11529	Control	AGGTAAGCCCCTTAGAACTG
11530	Control	TTGGTCCGAGTCTGGAGAAA
11531	Control	CAAAAAGGCGTTTGAGCGTG
11532	Control	GTGTAAATCTGTCCAAGTAG
11533	Control	AATCTTACTCGTCCTCCTTG
11534	Control	GACCTATGCCAGAAAGTTCG
11535	Control	ATGCGCAGCTCCAGAATTTT
11536	Control	GGTCCCTCAGGGTGCAACTT
11537	Control	GCCCCAAGCTAGAACTCAGC
11538	Control	CCATTCCGTAAGGGCTTGGA
11539	Control	AAACCTAGCCCCAATACTTA
11540	Control	TACGGGTGTCTCCATCTTGC
11541	Control	GGTCTGCTCCAATGGGAACC
11542	Control	CTTACCCCTATTATAATGAA
11543	Control	GAGCAATCCAAAGTTAACGG
11544	Control	TCACTTTACACTTGTAGTGT
11545	Control	TTCTTAGAAGTTGCTCCACG
11546	Control	ATCTCTATACTGTCACTCGC
11547	Control	GAACGTAGAAATTCCCATTT
11548	Control	CGAGTGGGAAACGGGAATCA
11549	Control	ATTGAGAATTCGTTTCAAGG
11550	Control	CATCATAAATGTACAACGGG
11551	Control	TCCCTCCTAGTCAAGAAGAG
11552	Control	CGACTGACCCCTGGGTGAAG
11553	Control	GAACGTCCAAGCAAGGGAGC
11554	Control	GGGTGGTCATTCTCTACTTG
11555	Control	AGTGAGTGACAACCAGATCG
11556	Control	GGAAACTGGGTTTCATCGTC
11557	Control	TATGACCCTGTTACATTGCC
11558	Control	TGAGCATGTCGGGAGTAACT
11559	Control	CGGTGAGCCACACGAAGGAA
11560	Control	TGGGGACGTTTATCAATATA
11561	Control	GTTCATTTCCAAGTCCGCTG

11562	Control	CGTCCCTTCGTCTCTGCTTA
11563	Control	TGCATGCCGAGCATTTTCAA
11564	Control	GTTTTTGGTTAATTGCCTAC
11565	Control	CGGGTTATGTGATGCCATCT
11566	Control	CATTAGCAGCCCAGCGCCCA
11567	Control	ATCAGCCCATTTCTGCGCAC
11568	Control	GTGAAACAGAGGGTCCATCA
11569	Control	CGTAGTAAATATCTAGCTAA
11570	Control	ATTAAACGACACCTTATTCT
11571	Control	CCCTCAGGAGCTACTAAGGT
11572	Control	GAGGGGGCTTCAAACATGTG
11573	Control	TCGCAAGGAAGCCAGCTAAG
11574	Control	CGGAGCTTAGCGTGGGGGCG
11575	Control	GCTCCCATCCATAGTAAAAA
11576	Control	TGACTAGCTCTTACATATTC
11577	Control	CCTTATGGAATCAGACCGTT
11578	Control	CGTCAAGTATTAAGCTGCTT
11579	Control	ATAGCGGATGTCCTTGGAAA
11580	Control	ACGCATGCTTCCCAAAGCGT
11581	Control	AGTATTAGGTACCTGCCCTA
11582	Control	TAGAGTGCATAAGAGAACCA
11583	Control	AGTGTATCTTCCACCTGTCT
11584	Control	AGTATGAGACTCATAGGGTG
11585	Control	GAGTTTCTAGTAGTGGTAGG
11586	Control	GAAACGAGAAGTTTGTACTA
11587	Control	AGCCCGCCACAGAATAGCTG
11588	Control	GTTGATCGAAAATGGGAGAA
11589	Control	TAGGGGATTAGCTGACAGTC
11590	Control	GCTTGCATAATTCTGGCCAG
11591	Control	GCTAAGGTCATGTTTGCAAT
11592	Control	GACACTATCCAACCCAAGAG
11593	Control	GAGTTATTTATTCTCTCGAG
11594	Control	CAGTCGTTTCTATGGGATCT
11595	Control	AAAATCGATGGGCTGAATCT
11596	Control	GTTCTGCCACCGACTTGGCC
11597	Control	GACGCCTTGCCCGGCTCACA
11598	Control	ATTTAGTAATGCACACCCAG
11599	Control	TAGTTCTAATCGTTCCTTGA
11600	Control	CTTTTCAGCTGAGACGTACG
11601	Control	CACCCTTATATTCAGTAACT
11602	Control	TGCCCACTTAGCAACACTCT
11603	Control	CACTGCAGTATTCGTGGCCT
11604	Control	TGCCTCTCCCTTACCCGGAC
11605	Control	AGAGCATGATGACCCGTGAC
11606	Control	GGTGTCACCACCGCTTACCA
11607	Control	CAGCCTATTTTGCTACCTAC
11608	Control	GCCAGCGGGGATATGGTGAA
11609	Control	ACGCTCTCCTGGCAACAAGT
11610	Control	TGGAGAAACGAGGTGTAATA

11611	Control	TATAGACCTCCCAAATACAT
11612	Control	GGCGTTAATTAAACTGTTTT
11613	Control	CAGGGTTGCGCAGAGGACTC
11614	Control	AAGTGACGGTGTCATGCGGG
11615	Control	TGTCAGTAGTCAGGACCCCG
11616	Control	GGAATCCGGAGCTCATGAGG
11617	Control	CATTAAACCTTGCCCCACAA
11618	Control	CGGCACTAGAAGTTTTTGAA
11619	Control	CCAGTTATAATTAGGGGTTT
11620	Control	CCTCTCACCGACTCTGTGTC
11621	Control	TAACCCAGAAGCCCATTCAG
11622	Control	GCAGTACTACTGAGTTTTTC
11623	Control	CGACCCATGGATGTGAACCC
11624	Control	AGATGGTCAGATCTAGTAGT
11625	Control	CCACGATGCCACCTCATCCC
11626	Control	TAGTCCTTAGGGTGGGCTGA
11627	Control	CGTGTGTGGGTAAACGGAAA
11628	Control	CTCTGTGAGATGTCCCGAAA
11629	Control	TTGGGGATTAACCCAGAGCC
11630	Control	TTCCTAGAGTTGGCAGACGC
11631	Control	GACAGTGAAATTAGCTCCCA
11632	Control	TGTTCTACTTTCGAAGTTAA
11633	Control	GGGAGTTGATTGTTTCGAGA
11634	Control	TAGAATTTGACCAAAGGCAC
11635	Control	CTTCTAGCTGGTTCATTGCT
11636	Control	CCCTGTGAAGGAGGCGTAAG
11637	Control	CAAGCATTTAGACACCTGTC
11638	Control	GTGTATGAATGTTAATTCCG
11639	Control	CGGCCAAAGAATTAGAAGTT
11640	Control	GGACTTTAGTTCCAGTGGTT
11641	Control	TGAACGGTGAAGAGATAGGG
11642	Control	AGCCGGCTTGTGACAGTGAA
11643	Control	CCAAAAAGATGAATATCTCG
11644	Control	GATTCATACTAAACACTCTA
11645	Control	ATTTATAAACACAGGGTCGC
11646	Control	AGGGGCAGGGCTATCTTATG
11647	Control	GTAAACTTTGTCTGGAGTAT
11648	Control	GAATAGATTTGTCAGTTAGG
11649	Control	AGTTCTGTTCGATAGATGCC
11650	Control	GTGATAATGATGTATTCTCG
11651	Control	TCCTCACCTAAAGTGCAATA
11652	Control	GTTTTCAGTTGCCCAACAGC
11653	Control	CGCGCAGAAGGCAAGCAGGG
11654	Control	ATTTTCGAAAGCTTAGGCCA
11655	Control	GTTTCGAAACTTGAAGTAAG
11656	Control	CTAACATGAGTACATAGATA
11657	Control	ACCACTAATGAGATTCTTGT
11658	Control	AGGATGCTGAACAAGTACGT
11659	Control	TTCTAAGCGCCCTGGGGACA

11660	Control	ATCCTAGGTACAAAAGGACG
11661	Control	GTATTACTGATATTGGTGGG
11662	Control	CTCATGAGTCGTTTCTTTCA
11663	Control	CTTAAGGCGAGAAAAATTAG
11664	Control	TTAACGCCTTATTTTTATGT
11665	Control	GGATGTTTCTGTGCGCACAT
11666	Control	TCAGTATCGGCTGCTGGTAA
11667	Control	GTCAAGAGATTATGAGATTC
11668	Control	CAGACCCAGTAAAACCACCA
11669	Control	CACCATAGAACCTGAAATAC
11670	Control	AGCTGAAAATATACGTATTC
11671	Control	TCTATTTTGTCTGCGCAGAA
11672	Control	GCTTTTTCCAGCGAGAGCAA
11673	Control	GGATTAATTCGCTAAATGAT
11674	Control	ATAAGCTACTCTGAGTTCCT
11675	Control	GTGAACTGCAATCTTATTAT
11676	Control	GATTTTAGCTTAGGTCTTAC
11677	Control	ATGCAAGACAGCCTCCCAGC
11678	Control	TGTAGTCTGGGGTAGACTCC
11679	Control	GACGAGGGCGGCAGAGCAGT
11680	Control	GGACCAACTCCCCTGCCCAC
11681	Control	GGGGCAGGGACCGAGTATCC
11682	Control	CTGCCCTCTTGAAATAGCCA
11683	Control	AGGGATCGTTAGGAAGGGAA
11684	Control	TGAGTCTTACTAGGTCCTGT
11685	Control	CACATAACATGAGGTATCAG
11686	Control	CTTCCTGCGTGGCTTTAAAC
11687	Control	ATAGCTAAAGTTGATGTGTA
11688	Control	CTTAACACTTTAGTGCGTCA
11689	Control	CTTATTACTCCAGTATAAGA
11690	Control	AGGGAAACCTCTATGGGTAA
11691	Control	AAGCCATTGTATAACTCCAG
11692	Control	TGCAAGGACCTGGTATGAAC
11693	Control	CCAGAGCCTTGGTTTATATC
11694	Control	GCTGGAGAGACAATTCTACT
11695	Control	TGTAGATATAGGGTGTCTAC
11696	Control	GCGAATGCCTGAAAGTATAA
11697	Control	ATACTCTCACAGGTACATAA
11698	Control	ATGGAAGAGCGTCATGACTT
11699	Control	TTGCAATGCTGCTATAGAAG
11700	Control	AAGGCAATTTACTGGATCCT
11701	Control	CTGCACTGTGGAGACGCCCG
11702	Control	AGGACACAGGATACGCAGCT
11703	Control	TGCTCACTCCACTCCTCAAC
11704	Control	GGAGAGGAAAATCGGCACAG
11705	Control	GCACAAAACCAGCAATGGTC
11706	Control	AGCTTAATGTGCAGGTCAGA
11707	Control	ACTAGGGCAGTGTGTCTGCA
11708	Control	CTCCCAGTACCAGTCAGTTC

11709	Control	TCAGGATCAGGGTGTATGGC
11710	Control	GGGAGGTGGCTTTAGGTTTT
11711	Control	AGGATGGATTGAGCAGCGGT
11712	Control	AACAGGAAACGTGACTAAAG
11713	Control	CTGGCTTATTAGCTATAAAG
11714	Control	GCAAAAGTGGCATAAAACCG
11715	Control	ACAAAAAACTACGGTGATTA
11716	Control	TGACACATTGGCTGGGTGTT
11717	Control	ATGATATCTGACATGCAGCG
11718	Control	AGAATGCCATCTAGTTAGTA
11719	Control	TTGGCCTGGAATTTAGGATC
11720	Control	TCTGAAAAATAGGCCCAACC
11721	Control	GTGGGGTAGTCTGTTGCTCG
11722	Control	GAGCAGCTGTCAGGTCTTGT
11723	Control	CATGGCATAAGTATAAGACA
11724	Control	GCCTGGGTTTTGGTGCATAC
11725	Control	GCACTTTGTTTGGCCTACTG
11726	Control	TAGATCGAGTTTATTTTCCT
11727	Control	CTCTTTTGAGATTGACAAGT
11728	Control	TTGCAGCCACTCCTGCAATA
11729	Control	GAACTCAACCAGAGGGCCAA
11730	Control	TATCTCTGCATAAAAGTGCC
11731	Control	CAAATGCCATTTAGGTTATC
11732	Control	GCACGAGGTGAACAGCCGCT
11733	Control	GTGAGATGCACCTTTTGGCC
11734	Control	TGGCAGTGGAAAATCTGCGG
11735	Control	TCATGCTTGCTTGGGCAAAA
11736	Control	TGACCTCTGAGGAATTCACA
11737	Control	AATTTATCGTCAACATTAAT
11738	Control	AAACTGTAGTGCAGGGTCAG
11739	Control	AAGTGTGTGCATAGCAGGGT
11740	Control	GCAATGCAATCGCAGGAGCA
11741	Control	GGTCCCTCTGGCTGGGTTAA
11742	Control	GAGCTTAGCAAAGGGTTGGG
11743	Control	AACTACAAGTAAAAGTATCG
11744	Control	TTATATGGTTTTAAACGGCT
11745	Control	GAGGACCTTAAGGTGACATG
11746	Control	CTTAAGTCATGAGCAAAGAT
11747	Control	AAACGAGATCGAGAAAGGTA
11748	Control	ATCTGCCATGGCGTCCTGGC
11749	Control	AGTGCTACTGAAACTTGCCT
11750	Control	AGTGTTTGAAAAAAGGGCGG
11751	Control	CCAGAAAGCCTCTCCATTAC
11752	Control	TCCTGCCAAGAAACACCCTT
11753	Control	GACCTTCATTGAAGAAAAGC
11754	Control	GAGATGATAACTTAATTTGT
11755	Control	AAGAGATCACATCTAGGCCA
11756	Control	GTTAGGAATAAAAGCTTTGA
11757	Control	ACTTCCCACTTCTTAGGTTG

11758	Control	CCATTCACAATCCCACTACA
11759	Control	TGTTTTGCATGTTGCATAGG
11760	Control	TAAGCCTCATGAAGGAGGGG
11761	Control	GGTACTGGAAGTCCGAAACC
11762	Control	AAATAATATGCATCTCTCGA
11763	Control	AGCTGGACTCTGTAGAAATC
11764	Control	CCTAACCCCTGGCCAGGAAG
11765	Control	AACCACGGCATTGAGAGGTG
11766	Control	AGTACCCCTAGGTATGGGGA
11767	Control	CCTCTGGAAGGACACTTCTG
11768	Control	TCTACCTATTGTGGAATTTG
11769	Control	TACATGGTATAGTGTTTATT
11770	Control	TGCAACCTTCCTTTTCAGGA
11771	Control	GGGCAGAAGTTGCTGTCCTG
11772	Control	AGGAGTGTTATGAAAAGGCT
11773	Control	ACAGGTTCTTATTCATTGAC
11774	Control	ATCCATGTGGTTACAATATT
11775	Control	GGGGAAACAAGTAGGCTTTG
11776	Control	TCAAGGAGCCTTATTTCTTG
11777	Control	AGAGGGATTGGGAGCTTGAC
11778	Control	ACTGCTGCTGACATCTCTTA
11779	Control	TGGAGTGCACGCGAAAAGAA
11780	Control	ATCTACAATCCAGCCCTCTA
11781	Control	TTTTACCTTGTTCACATGGA
11782	Control	GTAAATTAATGTAACTACCG
11783	Control	ATTAGGCCTTTTTCTTAACT
11784	Control	TCCCTGCATTCATGGTTTTA
11785	Control	TGTGAAAGTTTATGGTGTTA
11786	Control	GGAGTATAAAAAAGTGGTGT
11787	Control	GCTTTCAATTGCAAAAATAC
11788	Control	ACTCAGCCATTTTATTAGAA
11789	Control	AAAGACTTGCTCCAAAACAC
11790	Control	GGCTCTGGGGCTCACGGACG
11791	Control	ACCCATGAGTTAAGTTTTCT
11792	Control	TAAATTCAGACCACAGCTAA
11793	Control	GGAATGTCCTAGGTTACTGA
11794	Control	CTCTCTCCTACCTGTCCACC
11795	Control	CCTTGAAATCAAATCAAACC
11796	Control	AGGGTCAGTCTGTCCTTTTT
11797	Control	CCTCTCCCAATCTACAGCTC
11798	Control	GCAGCGAGATAACTTGACTC
11799	Control	ATCAAAGTGTCTGACTTATT
11800	Control	CAAATACAATTACTTATAGC
11801	Control	CAAAATCTAGCAATTTTTGG
11802	Control	CTAAAATTTTTGCGTGTTTG
11803	Control	GCAAGGTCATGAAACCAAGC
11804	Control	CGTGTAAAAATACCTTTCTA
11805	Control	CTGTGTTGCTCCCCTCCCCT
11806	Control	GTCTTCACAGGGTGCAACGA

11807	Control	GCAAATTCAGACAGCTAATT
11808	Control	TTCCATTGGCTGGAATCTGA
11809	Control	CTGAAGGTGTCTGGCAGAGC
11810	Control	GTAATTTTATGAGTTAAGTG
11811	Control	TAGTACATGTGTGGTATTTA
11812	Control	AGTGTGGAAAAGCTAACAGA
11813	Control	TGCCCCACCCCAGTGCTCAG
11814	Control	GATCCTTCTCTTTTTGGTTT
11815	Control	TCGAGAGGAAAAACACACTG
11816	Control	AATTTAAGCTTCGTAACTGG
11817	Control	ACTGCTGCTGTCTTCTAAAT
11818	Control	GATCCAGCAATATTTCTTAA
11819	Control	CCAGCCAAGAATCCAGCCCC
11820	Control	CAGAGACAATGACATGTAGA
11821	Control	GGAACGAGGCAGTGACAGGG
11822	Control	GCTAGTTTCTGTTAGCTCCT
11823	Control	TTTGGCAGTACCTTTTATTA
11824	Control	AAAATAGCAGTAAACTCAAC
11825	Control	TCAGCAAAGGACGAAACAAA
11826	Control	AAGGACTGGAATATGGAGAG
11827	Control	AGTTCCCAGAAATATATTGC
11828	Control	GTTTAAAGAAAGGGGCTAAG
11829	Control	CCAGTTGCTCTGGGGGAACA
11830	Control	GAAAGGCATAGTGAGAATGG
11831	Control	ACTATTTAATATTGGTAAGT
11832	Control	TCATCTTACATCTGGGAGAC
11833	Control	CAAGTTTTCTGAAAGGCAAT
11834	Control	AAAGATATAGCAAATTATGG
11835	Control	ACCTTACAATAAGTTATATT
11836	Control	TGTCATGCGTCACTTAGTGC
11837	Control	AAGTGACAGATGGGCAGGCG
11838	Control	GGTCTCTGTACGGGCCGCCC
11839	Control	TTGGATATTAATTAGACATG
11840	Control	GACTTCTAGAATATAAAAGA
11841	Control	TTCCTAAGATTTTTAAGACT
11842	Control	TAGTGGAAAATGAATATTGA
11843	Control	TTTTTAATACAAGGTAATCT
11844	Control	CGGGATGCAGCTGGAGAGGA
11845	Control	TATAACGGTAATGGGATTCC
11846	Control	AAGAAGAATTGGGGATGATG
11847	Control	CAAAGGGGGAAGTGCAGGGA
11848	Control	TAAAGCAGAAGAATATACAG
11849	Control	ATTTAGTAGACATTGGGTCT
11850	Control	GAGAAGGATGGAAATTAGAA
11851	Control	GTGTAGTCCTAGCCATGGGG
11852	Control	GCCCCGCCGCCCTCCCCTCC
11853	Control	AAAGAAAGAGGAATAGTAGC
11854	Control	GAGAAGTGGGGAGCCATTGG
11855	Control	AGAAGAAAAAAATGTCTACG

11856	Control	GACTGAAATCCAAGGACTGT
11857	Control	ATATTTTATGACATAAAAAT
11858	Control	TAAACAAAAAGGAAATAGTT
11859	Control	TTTCCCATGATCATTTAGTG
11860	Control	CTGAGTGAAAAATAAAAGTT
11861	Control	GACAATCATGGTGAAAGCGG

Supplementary Table2 shRNA and siRNA Sequences

shRNA	Sequences (5'>3')
shRNA-MAP2K2	CAACATCCTCGTGAACTCTAG
shNC（negative control）	TTCTCCGAACGTGTCACGT
**siRNA**	**Sequences (5'>3')**
1-MAP2K2(HUMAN)-sense	CAAAGACGAUGACUUCGAATT
1-MAP2K2(HUMAN)- antisense	UUCGAAGUCAUCGUCUUUGTT
2-MAP2K2(HUMAN)-sense	GCAUUUGCAUGGAACACAUTT
2-MAP2K2(HUMAN)- antisense	AUGUGUUCCAUGCAAAUGCTT
3-MAP2K2(HUMAN)-sense	CCAUCUUUGAACUCCUGGATT
3-MAP2K2(HUMAN)- antisense	UCCAGGAGUUCAAAGAUGGTT
1-MAP2K1(HUMAN)-sense	GAGGUUCUCUGGAUCAAGUTT
1-MAP2K1(HUMAN)- antisense	ACUUGAUCCAGAGAACCUCTT
2-MAP2K1(HUMAN)-sense	GGACUCAUUACUCUGUGCATT
2-MAP2K1(HUMAN)- antisense	UGCACAGAGUAAUGAGUCCTT
3-MAP2K1(HUMAN)-sense	GAGCAGAUUUGAAGCAACUTT
3-MAP2K1(HUMAN)- antisense	AGUUGCUUCAAAUCUGCUCTT
1-ERK(HUMAN)-sense	CAGGGUUCCUGACAGAAUATT
1-ERK(HUMAN)- antisense	UAUUCUGUCAGGAACCCUGTT
2-ERK(HUMAN)-sense	GCUGCAUUCUGGCAGAAAUTT
2-ERK(HUMAN)- antisense	AUUUCUGCCAGAAUGCAGCTT
3-ERK(HUMAN)-sense	CCAGGAUACAGAUCUUAAATT
3-ERK(HUMAN)- antisense	UUUAAGAUCUGUAUCCUGGTT
1-SP1(HUMAN)-sense	GCUCUGAACAUCCAGCAAATT
1-SP1(HUMAN)- antisense	UUUGCUGGAUGUUCAGAGCTT
2-SP1(HUMAN)-sense	GCAGACCUUUACAACUCAATT
2-SP1(HUMAN)- antisense	UUGAGUUGUAAAGGUCUGCTT
3-SP1(HUMAN)-sense	GGGAAAGUGUAUGGCAAGATT
3-SP1(HUMAN)- antisense	UCUUGCCAUACACUUUCCCTT
Negative control-sense	UUCUCCGAACGUGUCACGUTT
Negative control- antisense	ACGUGACACGUUCGGAGAATT

Supplementary Table3 Primer Sequences

Gene	Sequences (5’>3’)
CRISPR Library-F	TGGACTATCATATGCTTACCGTAAC
CRISPR Library-R	TGAATACTGCCATTTGTCTCAAGATCTAG
Human MAP2K2-F	GGAACTTGACGAGCAGCAGA
Human MAP2K2-R	AAGGTGGATCAGCTTCCTGG
Human MAP2K1-F	TGAAGCTGGAGAGGACCAAC
Human MAP2K1-R	CTCCCACCTTCTGCTTCTGG
Human ERK-F	GCCGAAGCACCATTCAAGTT
Human ERK-R	CCTCTGAGCCCTTGTCCTGA
Human SP1-F	CCACCATGAGCGACCAAGAT
Human SP1-R	TGAAAAGGCACCACCACCAT
Human MAP2K2-promoter-F1	GTGGTAAGGCAAGCGAGGGCG
Human MAP2K2-promoter-R1	AGGGGAGGGGCGGCCACAAG
Human MAP2K2-promoter-F2	GGTTCTCTCAGCCCCAGCCTG
Human MAP2K2-promoter-R2	GGCGCCCTCGCTTGCCTTAC
Human MAP2K2-promoter-F3	CCATCCTGGCTAACACGGTG
Human MAP2K2-promoter-R3	GGAGTGCAGTGGTGCGATCTC
Human GAPDH-F	ACCCACTCCTCCACCTTTG
Human GAPDH-R	CTCTTGTGCTCTTGCTGGG
